# Allelic expression patterns of imprinted and non-imprinted genes in cancer cell lines from multiple histologies

**DOI:** 10.1186/s13148-025-01883-3

**Published:** 2025-05-25

**Authors:** Julia Krushkal, Travis L. Jensen, George Wright, Yingdong Zhao

**Affiliations:** 1 Division of Cancer Treatment and Diagnosis, Biometric Research Program, National Cancer Institute, 9609 Medical Center Dr., Rockville, MD 20850 USA; 2 The Emmes Company, LLC, Rockville, MD 20850 USA

**Keywords:** Imprinting, Monoallelic expression, Cancer, Single nucleotide variant, Gene expression

## Abstract

**Background:**

Imprinted genes are epigenetically regulated in normal tissues to follow monoallelic expression according to the parent of origin of each allele. Some of these patterns are dysregulated in cancer.

**Results:**

We developed a novel computational multi-omic pipeline to evaluate monoallelic and biallelic expression patterns based on matched RNA-seq expression data, whole-exome sequencing information, and copy number data. We analyzed allelic expression of the entire genes, individual isoforms, and each exon of 59,283 autosomal protein-coding and ncRNA genes, with a focus on 94 genes previously reported to be imprinted. We analyzed 108 cell lines from 9 different tumor histologies using molecular data from the DepMap Portal for the Cancer Cell Line Encyclopedia. Allelic expression patterns of imprinted genes and isoforms in tumor cells were variable. We also identified additional genes and isoforms with predominantly monoallelic expression due to a variety of potential mechanisms. We provide a novel public dataset of transcriptome-wide allelic expression patterns in cell lines from diverse tumor categories, which can serve as a resource for future cancer studies. We examined associations of in vitro cell line response to antitumor agents and repurposed drugs with allelic patterns and overall levels of isoform expression of imprinted genes and of additional genes with predominantly monoallelic expression. Drug response was associated with isoform expression patterns of multiple imprinted genes including *CPA4, DGCR6, DNMT1, GNAS, GRB10, H19, NAA60, OSBPL5, PHACTR2,* and *ZFAT,* predominantly monoallelically expressed *MAP2K5* and *BCLAF1,* and additional predominantly monoallelically expressed genes. Multiple associations may be related to mechanisms of drug activity, including associations between the response to the DNA damaging agents and allelic expression of *ZFAT, CDC27,* and *BCLAF1* isoforms*,* and the response to inhibitors of multiple signaling pathways with expression patterns of *GNAS* isoforms.

**Conclusions:**

Tumor cells have a range of monoallelic and biallelic expression patterns in both imprinted and non-imprinted genes and are likely affected by the complex interplay among changes in allelic expression, sequence variants, copy number changes, and expression changes of biologically important genes. Multiple isoform-specific patterns of allelic expression were associated with drug response, indicating complex mechanisms of cancer chemoresistance.

**Supplementary Information:**

The online version contains supplementary material available at 10.1186/s13148-025-01883-3.

## Background

Imprinted genes are monoallelically expressed according to the parental origin of each allele. They play important roles in embryonic and postnatal development, and many of them promote cell proliferation and body growth [1–7]. In healthy individuals, the imprinting patterns in the germline and somatic tissues are controlled via epigenetic mechanisms, which involve DNA methylation of imprinting control regions (ICRs, which harbor differentially methylated regions, or DMRs), histone modifications, chromatin regulation, silencing by long noncoding RNA (lncRNA), and CCCTC-binding factor (CTCF) binding [2, 6, 8–16]. Imprinted genome loci frequently contain multiple isoforms with different patterns of parent-of-origin monoallelic or biallelic expression in the normal tissues, and some imprinting patterns are also tissue specific or transiently appear at specific developmental stages [2, 3, 9, 14, 17–21].

Disruption of germline patterns of many imprinted genome loci, e.g., those at 11p15, 14q32, 15q11-q13, and 20q13, results in congenital imprinting disorders [12, 22–24]. It may involve changes in the number of maternal and/or paternal allele copies, genomic and chromosomal alterations, or epigenetic dysregulation [12, 23]. However, imprinting of some genes, e.g., *HM13*, *GABRG3, IGF2R,* and *nc886/VTRNA2-1,* is polymorphic in healthy individuals, whereas *RB1* expression is skewed in favor of one parental allele rather than adhering to strict monoallelic patterns [21, 25–31]. Such skewed allele expression according to parental origin (noncanonical imprinting) may be tissue specific [32].

Malignant cells acquire multiple epigenetic changes, some of which include dysregulation of imprinting [2, 8, 18, 22, 33–47]. Some imprinted loci gain or lose imprinting or undergo epigenetic switches of allelic expression [18, 33–38, 45, 48]. In addition to changes in their imprinted status, imprinted genes may also undergo other molecular alterations in cancer, e.g., genetic mutations, copy number gain or loss, copy-neutral loss of heterozygocity (cnLOH, also known as uniparental disomy, or UPD), amplified loss of heterozygocity (aLOH), fusions, DNA methylation changes, transcriptional up- or downregulation, and post-transcriptional regulatory changes [2, 8, 18, 33, 34, 37, 39–45, 49–52]. Dysregulation of many imprinted genes, e.g., *IGF2, H19, PLAGL1* (*ZAC1*), *CDKN1C, SLC22A18, MEG3, DLK1, PEG3* (*ZIM2*), and *GNAS*, has been associated with diverse protumorigenic roles including activation of oncogenic transformation, increased cell growth, metabolism, and proliferation, chromatin instability, and metastases [1, 2, 4, 8, 53, 54]. For example, the loss of maternal imprinting of the insulin-like growth factor 2 gene, *IGF2*, accompanied by its increased expression, is the most common genetic or epigenetic alteration in Wilms tumors, an early driver event in the development of ileal neuroendocrine tumors (I-NETs), and has protumorigenic effects in many other cancers [53–57]. The loss of *IGF2* imprinting or dysregulation of its expression through other mechanisms have been associated with increased chemoresistance, radiotherapy resistance, and immune evasion in diverse cancer models in vitro and in vivo; however, *IGF2* imprinting was also associated with improved disease-free survival of esophageal cancer patients [53, 58–60]. Alterations of other imprinted genes have also been associated with effects on malignancy. For example, differential imprinting of certain single nucleotide variants (SNVs) in *ZDBF2* was associated with survival of breast cancer patients [45], whereas loss of imprinting (LOI) and overexpression of *HM13* were associated with decreased overall patient survival and with tumor progression in clear cell renal cell carcinoma [61]. In contrast, transcriptional upregulation of *H19* in neuroendocrine prostate cancer and its contribution to treatment resistance have been attributed to non-imprinting mechanisms [62]. Due to complexity of imprinting regulation and the magnitude of molecular changes in cancer, there is a very limited understanding of the effects of allelic expression patterns of imprinted genes on treatment resistance and on patient outcomes.

In our earlier study, we reported that drug response of cancer cell lines and patient acute myeloid leukemia (AML) specimens was associated with variation in the copy number, DNA methylation, and gene-averaged expression levels of imprinted genes, with a particularly notable association of the copy number of multiple imprinted genes at 20q11-q13.32 with drug resistance [63]. That analysis was restricted to whole gene associations and did not distinguish between monoallelically and biallelically expressed transcripts. While associations of these molecular features of imprinted genes in tumor cells with treatment response may have important clinical implications, potential effects of changes in their imprinting status or the effects of individual isoforms on sensitivity or resistance to drug treatment remain an open question requiring further investigation. In the current study, we tested whether cancer drug response may be mediated by allelic patterns of expression of individual isoforms. We conducted a high-resolution analysis of allelic expression patterns of imprinted and non-imprinted genes, their isoforms, and individual exons. We further examined associations of cancer drug response with allelic expression patterns of individual isoforms of imprinted genes and of other predominantly monoallelically expressed genes.

A number of studies of normal tissues used DNA methylation levels or the absence of heterozygous SNVs in expressed transcripts to assess the imprinting or monoallelic status of multiple genes [11, 64–66]. Some studies of imprinting in normal tissues had the added parental information [67], thereby increasing the accuracy of inference. A broad overview of the use of different omics datasets in imprinting studies has been provided by Hubert and Demars [9]. In addition to dysregulation of imprinting in tumors, many cancer cell lines lack both parental information and the data from the matched normal tissue, which complicates the inference of the parent of origin-based allelic expression patterns. Several earlier studies were able to make broad indirect assessments of the imprinting status of imprinted genes in tumor samples and cancer cell lines by evaluating their DMR methylation, with subsequent validation of selected genes by evaluating expression of heterozygous SNV sites (e.g., [33, 34]). Another study identified imprinted genes as those containing SNVs in Hardy–Weinberg disequilibrium in the RNA-seq data of breast tumor and control samples and examined differential DNA methylation and differential expression in the follow-up analysis [45]. Additional methods utilizing differential allelic expression of imprinted genes in tumors have also been employed, e.g., quantitative imaging-based methodology of selected imprinted genes, which was suggested as a potential diagnostic tool for cancer biomarkers [38].

With the progress of next-generation sequencing, detailed transcriptome-wide RNA expression data and whole-exome sequencing (WES) or whole-genome DNA sequencing data are increasingly becoming available for tumor samples and cancer cell lines. This wealth of data provides novel opportunities for distinguishing between biallelically and monoallelically expressed transcripts based on the presence or absence of both alleles of heterozygous SNV sites in RNA expression data. In this study, we analyzed variation in allelic expression patterns of imprinted genes in cancer cell lines and examined their association with in vitro drug response. To achieve this goal, we used publicly available data generated by the Cancer Cell Line Encyclopedia (CCLE), Cancer Dependency Map (DepMap), and the Genomics of Drug Sensitivity in Cancer (GDSC) projects [68–74]. These cell line collections were derived from a variety of tumor histologies with distinct etiologies and molecular profiles. They provided resources for examination of variation among allelic expression patterns in cancer cell lines from distinct lineages. Such lineages included adult cancers that accumulated genetic and environmental influences over the life course, as well as pediatric tumors, where allelic expression patterns may be of special interest due to involvement of many imprinted genes in early embryonic and childhood development and frequent molecular alterations of imprinted genes in childhood cancers [57, 75, 76].

## Methods

### Generation of the list of 94 imprinted genes and the list of genes used in comparison

Additional file 1: Fig. S1 provides an overview of our workflow of molecular data collection and processing. We analyzed 59,283 autosomal genes for which WES, RNA-seq, and copy number data were available according to GENCODE release 38 annotation, with the primary focus on 94 imprinted genes for which matching molecular data were available based on the GENCODE annotation (Additional file 2: Table S1). Detailed information about each gene was collected from the Catalogue of Imprinted Genes [77–79], Geneimprint [80, 81], and biomedical publications [2, 3, 10, 19, 26, 28, 30, 31, 39, 42, 49, 50, 65–67, 81–97] during our earlier analysis [63].

All genes were manually reviewed for the concordance of their imprinted status across different sources. We included genes that had been previously reported as imprinted in embryonic or adult somatic tissues, placenta, embryonic stem cells, or induced pluripotent stem cells (iPSCs). Those genes whose imprinted status had been reported as conflicted among different sources were included if at least two references suggested their imprinting in any human tissue. Gene name synonyms were obtained from GeneCards [98, 99]. Gene chromosomal locations were reported according to GeneCards [98, 99], the Catalog of Imprinted Genes [77–79], and Bonaldi et al. [65].

We refer to the remaining 59,189 genes, which were not included in the original list of the imprinted genes, as the remaining genes or other genes. We compared allelic expression patterns between the 94 imprinted genes and the remaining 59,189 genes. We also compared the expression patterns between the 94 imprinted genes and the average values of 94 genes subsampled, with 1000 replications, from the list of remaining 59,189 genes, while stratifying within each tumor histology. We refer to the latter gene list as subsampled genes.

### Selection of non-imprinted controls

While we inferred allelic expression patterns for the entire transcriptome, for comparison purposes we also noted allelic expression patterns of several control genes that are not imprinted in normal tissues. Housekeeping genes *HMBS, GAPDH, GUSB, TBP,* and *ACTB* were used as positive controls [100]. We used *HBG1* (fetal hemoglobin F subunit gamma 1) and the neuroendocrine marker *CHGA* (chromogranin A) as negative controls, as both genes, which are not imprinted in normal tissues, are expressed only in subsets of tumors and are not expected to be expressed across all cell lines [101, 102].

### Acquisition of molecular data for CCLE cancer cell lines

We analyzed 108 CCLE cell lines from 9 pediatric and adult cancer categories, for which matching WES and RNA-seq expression data were available (Additional file 3: Table S2, Additional file 4: Table S3). The 9 tumor histologies included AML (the number of cell lines n = 17; 15.7%), bladder (n = 10; 9.26%), breast (n = 6; 5.6%), colorectal (n = 13; 12.04%), and head and neck (n = 16; 14.81%) cancers, neuroblastoma (n = 4; 3.7%), ovarian cancer (n = 14; 12.96%), pancreatic ductal adenocarcinoma (PDAC; n = 16; 14.81%), and small cell lung cancer (SCLC; n = 12; 11.11%). The tumor category of each cell line was assigned according to DepMap project r. 22Q2 data [69–71]. Cell line identities were verified using DepMap project information [103] to ensure the absence of duplicate cell lines. Additional information about the cell lines was obtained from Cellosaurus [104, 105] and original publications.

For each CCLE cell line, RNA-seq and WES BAM files were downloaded from the National Cancer Institute (NCI) Genomic Data Commons (GDC) CCLE HG19 Legacy Archive [106], which was publicly available at the time of the download. The GDC Legacy Archive was later retired; however, the BAM sequencing data for CCLE cell lines are currently a part of the DepMap project at the Broad Institute [69–71]) and are available from the National Cancer for Biotechnology Information Sequence Read Archive (NCBI SRA; accession PRJNA523380) [107] and via the Terra genomics cloud workspace [108]. RNA and DNA library construction, sequencing, and processing of CCLE data including variant calling, filtering, and validation have been previously described in the publications and data portals of the CCLE and DepMap projects [69, 109, 110].

Gene-level copy number data for the 108 CCLE cell lines were obtained from the DepMap portal (22Q2 release) [69–71]. These copy number data, generated by the DepMap project using WGS, WES, or SNP array data, were available as log_2_-transformed pseudocount of 1 [69, 70, 110]. To assess allelic patterns of expression of individual genes, these continuous values were transformed to gene-level discreet copy number estimates and rounded to the nearest integer (Additional file 1: Fig. S1).

### Molecular data processing and data filtering

Analysis of sequencing reads utilized their mapping to the human genome assembly hg19 according to the CCLE HG19 Legacy Archive information. Analysis was restricted to autosomal sites, and any reads mapping to sex chromosomes or mitochondrial genomes were excluded. Gene, isoform, and exon annotation was performed according to GENCODE, using lifted annotations from V38lift37 (Ensembl 104) mapped to hg19 (gencode.v38lift37). The overall composition of the 60,649 genes in the GENCODE 38 annotation, including different categories of protein-coding genes, ncRNA genes and pseudogenes, is available from the GENCODE project portal [111]. After restricting the data to autosomal sites, our dataset included 59,283 autosomal genes.

SNVs were annotated using gnomAD v. 2.1.1. Analyses were performed using Python v. 2.7 and R v. 3.4.1. Read counts were computed within each exon separately using HTSeq v. 2.0.4 and summarized for individual isoforms and genes based on their exon structure. Mapping of SNVs to individual alleles was performed using phASER v. 1.1.1, with recommended parameters (paired_end 1, mapq 255, baseq 10, threads 4, pass_only 0) [112, 113].

### Genome and feature annotations

The GRCh37/hg19 genome [114] was downloaded from UCSC and converted to fasta format using twobittofa utility from KentUtils v. 302.1 [115]. The genome data were limited to autosomes (chromosomes 1:22) in order to reduce gender-specific effects of chromosomes X and Y. Here and below, a feature indicates an entire gene, an isoform, or an exon. A GRCh38 to GRCh37/hg19 lifted version of GENCODE 38 feature annotations was downloaded from the EBI website [116]. Feature annotations were also reduced to chromosomes 1:22. Analyses of allelic expression patterns were performed at all three feature levels (whole gene, isoform, and exon levels). Additional information about biological interpretation of features of interest was obtained from Ensembl release 112 [117].

### Sample processing workflow

For each of the 216 samples (108 paired RNA-seq and WES samples), BAM files derived from paired-end sequencing experiments were downloaded from the NCI GDC Legacy Archive (Additional file 1: Figure S1). Samples were then indexed using Samtools (v. 1.17), and VCF formatted variants were called and sorted using bcftools (v. 1.17). The VCF formatted file was then indexed using GATK IndexFeatureFile (v. 4.4.0.0) and tabix (v. 1.17). Each sorted VCF file was passed through phASER (v. 1.1.1) with options paired_end = 1, mapq = 255, baseq = 10 and pass_only = 0, which attempted to phase variants in the VCF file. The htseq-count (v. 2.0.4) software with options "-a 0", "-s no", "–nonunique = all", "-i exon_id", and "-t exon" was also utilized to count mapped reads to each feature on the exon level.

### Preparation of the analysis dataset

On both the gene and isoform level, many summary metrics were captured from the phASER VCF formatted variant files, HTSEQ read counts on the exon level, and copy number on the gene level. For each cell line and feature (gene and isoform level), the number of phased SNVs, the number of heterozygous SNVs, and log_10_ length normalized HTSEQ counts (sum across exons of the feature) were assessed for RNA-seq and WES, and copy number on the gene level was tabulated. Only exonic SNVs and read counts were captured, whereas intronic SNVs were excluded from analysis.

### Identification of monoallelic and biallelic expression patterns

**Fig. 1:**
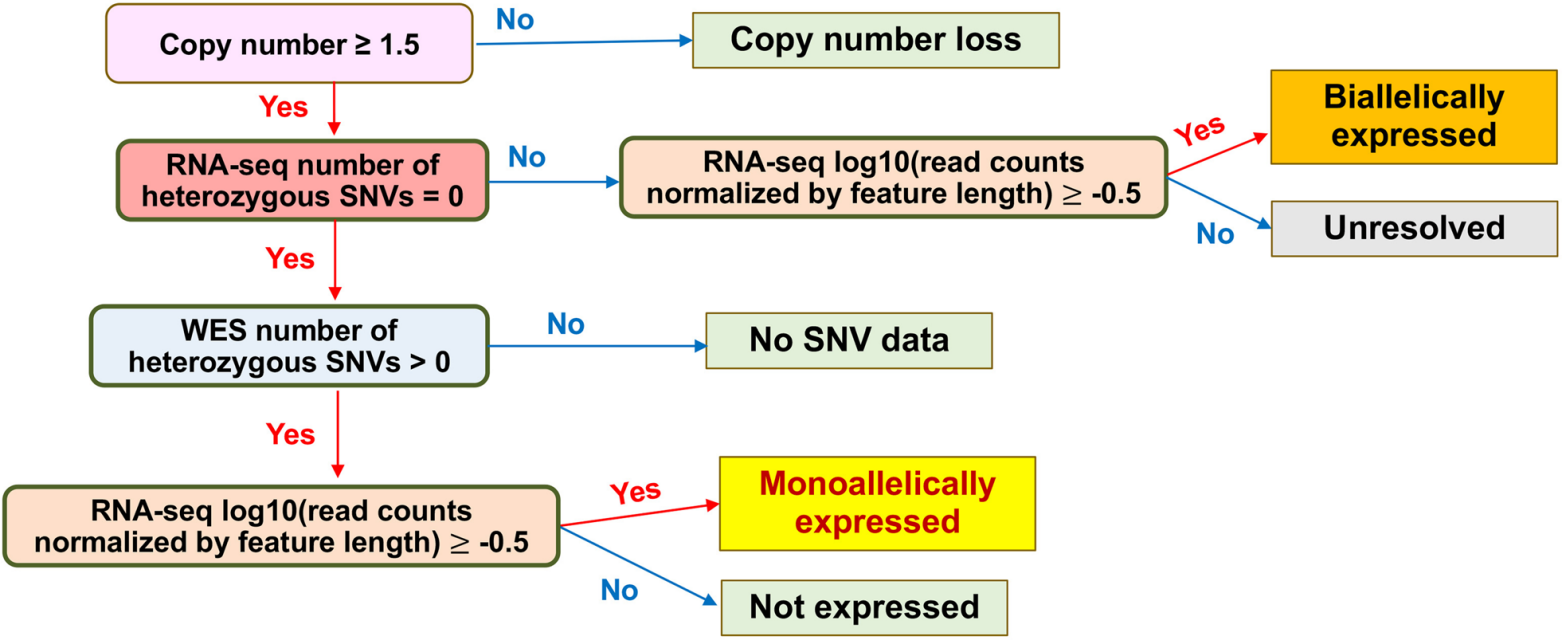
An algorithm for determination of allelic expression patterns. **Copy number loss** was assessed using gene-level data and indicates the loss of at least one allelic copy of the gene. All other inferences were specific to the **feature** type (exon, isoform, or gene), depending on the type of analysis. Additional quality checks examined for discrepancies when heterozygous SNVs were absent in WES data but were present in RNA-seq data, and both data types had sufficient sequencing coverage (RNA-seq log_10_(read counts normalized by feature length) ≥ -0.5, WES RNA-seq log_10_(read counts normalized by feature length) ≥ -0.5, number of heterozygous RNA-seq SNVs > 0, and number of heterozygous WES SNVs = 0)

We developed an algorithm for identifying monallelic and biallelic patterns of expression of each gene, isoform and exon (Fig. 1). This algorithm uses the presence of more than one allele in heterozygous SNVs as evidence for biallelic expression. In this study, we refer to expression of more than one allele as biallelic expression, with an understanding that expression of some of such features may be multi-allelic (> 2 alleles expressed) in case of copy number gain.

The algorithm uses matching data on heterozygous SNVs in RNA-seq files as compared to WES files and accounted for copy number loss or lack of expression (Fig. 1). All inference of allelic expression was performed independently for each cell line, without combining the information from other cell lines. Genes with copy number values < 1.5 were considered to have a copy number loss. After excluding copy number loss, among the features with available heterozygous SNV data, only those with log_10_ length normalized HTSEQ counts ≥ -0.5 were considered to be expressed. This cutoff was selected to separate noise from signal in heterozygous SNV detection from the sequencing data. The cutoff was determined based on examination of the inflection point for the LOESS regression line of the proportion of genes and isoforms with no detected heterozygous SNVs relative to log_10_ length normalized HTSEQ counts in RNA-seq and WES data (Additional file 5: Fig. S2).

A separate quality check noted discrepancies when heterozygous SNVs were absent in WES data but were present in RNA-seq data, while both data types had sufficient sequencing coverage. Such discrepancies could be due to a variety of reasons, including, e.g., sequencing errors or post-transcriptional RNA editing [118, 119]. Additionally, 116 genes (0.196% of all genes analyzed), including predominantly ncRNA, were flagged due to their feature annotation assignment to more than one chromosome, which could affect the accuracy of inference of their copy number and allelic expression patterns.

Tissue-specific differences between monoallelic and biallelic expression of the *PLAGL1* isoforms were analyzed using the Chi-square test among those tumor histologies where at least one isoform had one of such patterns.

### Validation of allelic expression patterns using previously published reports

Allelic expression patterns, overall expression, and copy number loss of individual isoforms of imprinted genes in CCLE cell lines were compared to previously reported gene-level results of Martin-Trujillo et al. [34], who examined molecular data in cell lines from 4 tumor categories from the Catalogue of Somatic Mutations in Cancer (COSMIC) project at the Sanger Institute [120]. Their study included 3 breast cancer cell lines (HCC1143, HCC1954, and MDAMB436) and 1 colorectal cell cancer line (HT55), which were also analyzed in our project using independently generated CCLE data [69–71].

After establishing transcriptome-wide expression patterns, we searched biomedical publications for the top genes which showed predominantly monoallelic expression in our data and were not included in our initial list of 94 imprinted genes. Our literature search confirmed the imprinting status of several such genes [121, 122] and suggested other explanations for monoallelic expression of additional genes [123].

### Cell line drug response data

In order to examine whether allelic expression patterns in cancer cell lines were associated with drug response, we analyzed those of the 108 cell lines (Additional file 3: Table S2, Additional file 4: Table S3) that had drug response data (IC50, representing the total drug concentration that reduced cell activity by 50%) in either secondary PRISM drug repurposing dataset or the GDSC datasets [68, 72, 124, 125]. The PRISM dataset included repurposed compounds which had been screened in DepMap cell lines [124, 125]. The GDSC drug response data included two batches, GDSC1 and GDSC2, screened in the COSMIC project cell lines [120]. These batches were analyzed separately in our study due to the differences between their drug screening protocols [72]. Information about cell lines in each tumor category which had matching drug response data is provided in Additional file 3: Table S2 and Additional file 4: Table S3. All drug response measures were transformed to the log_10_(IC50) scale. We refer to these values as log(IC50). Response measures for those agents which were screened in multiple datasets were analyzed separately for each dataset. For those agents in the GDSC1 dataset that had duplicate measurements [68], we used the combined average of their drug response measures from separate experiments. The concordance of drug response measures between the GDSC and PRISM datasets was reported previously [125].

#### Association analysis of drug response with allelic expression of isoforms of imprinted genes and aditional genes with predominantly monoallelic expression

To account for isoform-specific variation of imprinting and expression patterns, analysis of associations of allelic patterns or expression measures with drug response was performed at the isoform level. Isoforms from the same gene that had identical allelic expression patterns were combined into isoform groups. Each isoform group was used in the comparisons only once, instead of repeating the tests for individual isoforms in the group. Due to small numbers of available cell lines in individual cancer categories with matching drug response data (Additional file 3: Table S2), association analyses were performed in the combined pancancer dataset of all cell lines from all cancer categories. Associations with drug response were examined for allelic expression status and for overall expression (log_10_(HTseq counts normalized by length)) of isoforms of the 94 imprinted genes (Additional file 2: Table S1) and 60 additional genes with predominantly monoallelic expression (Additional file 6: Table S4). Those additional genes had > 50 monoallelic counts and ≥ 50% of monoallelic calls out of all calls for 108 cell lines, in gene-level data. We used Student’s *t*-test to compare log(IC50) between groups of cell lines with monoallelic vs biallelic calls for each isoform group. We restricted our analysis to the pairs of isoform groups and agents with ≥ 3 cell lines each in both biallelic and monoallelic expression categories with available log(IC50) data. Association of the mean overall expression of each isoform group with log(IC50) was examined using Spearman correlation for comparisons with ≥ 10 cell lines with both drug response and gene expression data. We also examined differences of the mean expression levels of each isoform group between cell lines with monallelic and bialleic expression using Student's *t-*test, for those isoform groups which had ≥ 3 cell lines with data in both monoallelic and biallelic expression categories. Additional file 7: Table S5 provides numbers of drugs in the PRISM, GDSC1, and GDSC2 datasets which were included in each analysis based on the sample size requirements.

Significance of the associations was evaluated using the Benjamini–Hochberg adjustment for false discovery rate (FDR) [126]. Separate FDR adjustments were performed for each analysis of isoform groups (allelic expression patterns vs drug response, allelic expression vs mean overall expression, and overall expression vs drug response). We refer to the *p-*values prior to FDR adjustment as *p*_0_ and FDR adjusted *p*-values as *p*_FDR_. We report the results satisfying the stringent cutoff of *p*_FDR_ < 0.05 to identify the strongest associations and also provide the list of associations satisfying the more relaxed cutoff of *p*_FDR_ < 0.125. The relaxed *p*_FDR_ cutoff of 0.125 was chosen to provide comparable numbers of top associations of drug response with either allelic expression patterns or overall expression levels.

#### Elucidation of possible mechanisms of monoallelic expression in 94 imprinted genes and additional 60 top monoallelically expressed genes

To explore potential mechanisms of monoallelic expression of the 94 imprinted genes (Additional file 2: Table S1) and 60 additional top monoallelically expressed genes (Additional file 6: Table S4), we examined whether SNVs in these 154 genes had the bias for monoallelic expression of a single nucleotide base (A, T, G, or C) across the cell lines. Predominant expression of a single base may suggest monoallelic mechanisms other than imprinting, e.g., RNA editing [127–129] or allele-specific expression (ASE) with selective advantage of a particular base [130]. In contrast, monoallelic expression without a bias toward any base across the cell lines would be consistent with imprinting, when expression would be dependent on the paternal origin of the allele but not on the type of the nucleotide in the transcribed allele.

In the examination of a potential preference for expression of specific allelic variants in monoallelically expressed genes and isoforms, the first step involved the capture of information incorporated in VCF formatted SNVs for each cell line and for RNA-seq/WES using the vcfR package v. 1.14.0 (Additional file 8: Fig. S3). After limiting input features to the 154-gene set, all SNVs expressing a single base RNA-seq and all heterozygous SNVs for WES were captured for each cell line. Single base expressing SNVs in the RNA-seq data may include those SNVs which monoallelically express a single base and also homozygous SNVs that biallelically express two copies of the same base. The latter were filtered out in subsequent steps, as described below. The primary alternative nucleotide base (relative to human hg19 genome reference sequence) was captured for RNA-seq, and the reference nucleotide base and primary alternative nucleotide base were captured for WES. Next, only those SNVs that belong to monoallelic features (based on algorithm assignment, Fig. 1) were retained and RNA-seq reference SNVs were inferred if WES identified a heterozygous SNV and no paired alternative single base expressing SNVs was recorded in the RNA-seq SNV data for a specific cell line. Finally, single base expressing RNA-seq SNVs were removed if there was no supporting paired WES heterozygous SNV associated with it across all cell lines (these are either missing or homozygous in WES SNV data).

For individual SNVs that were located within the genes determined to be monoallelically expressed in specific cell lines, the bias for expression of one allele over the other was tested in a manner analogous to the "flip test" used by an earlier study of Baran et al. [66], by comparing the number of cell lines for which the reference allele was monoallelically expressed, versus the number for which the alternative allele was monoallelically expressed, with a two-sided binomial test, under the assumption that under the null hypothesis either allele was equally likely. SNVs that were monoallelically expressed in < 6 cell lines were excluded from this analysis since the sample size would be insufficient to achieve statistical significance (*p* < 0.05). Based on this criterion, 474 out of 1067 heterozygous SNV loci were tested. They were located within 130 genes that included 70 previously reported imprinted genes and 60 other predominantly monoallelically expressed genes.

## Results

### Transcriptome-wide allelic expression patterns of CCLE cell lines

We inferred transcriptome-wide allelic expression patterns for each of the 59,283 autosomal genes in each of the 108 CCLE cell lines. Detailed information about these expression patterns in each cell line and their summary within each tumor category and in the pancancer dataset is provided as part of our comprehensive dataset of results (available at 10.6084/m9.figshare.27037978). Additional file 9: Fig. S4 shows overall numbers of reads mapped to exons in cell lines from each tumor category. In total, 29,621 genes either had a copy number loss, had no SNV data, or were not expressed in any of the 108 cell lines, and therefore, their allelic expression patterns could not be investigated. Without a consideration of the copy number loss status or SNV status, 25,161 genes (42% of all autosomal genes) had log_10_ length normalized HTSEQ counts < -0.5 and did not satisfy the threshold for expression.

The inference of allelic patterns was limited to features containing heterozygous SNV data. Among 6,402,564 gene-level counts across 108 cell lines, only 10.93% (699,865) of the genes had heterozygous SNVs counts > 0 in the RNA-seq data, and 16.58% (1,061,790) has such counts in the WES data, demonstrating that allelic expression could be inferred only for a relatively small subset of features. Among all genes and across all cell lines, 81.32% (5,206,616) of the genes had equal counts of heterozygous SNVs in RNA-seq and WES data. When restricting the comparison to genes with SNV counts > 0 in both RNA-seq and WES data, only 36.14% of the genes (150,200 out of 415,507) had equal counts of heterozygous SNV in both data types. While many differences in heterozygous SNV detection could be due to biological mechanisms including monoallelic expression, RNA editing, or other mechanisms, some of the differences could be for technical reasons, due to limitations in SNV detection.

**Fig. 2:**
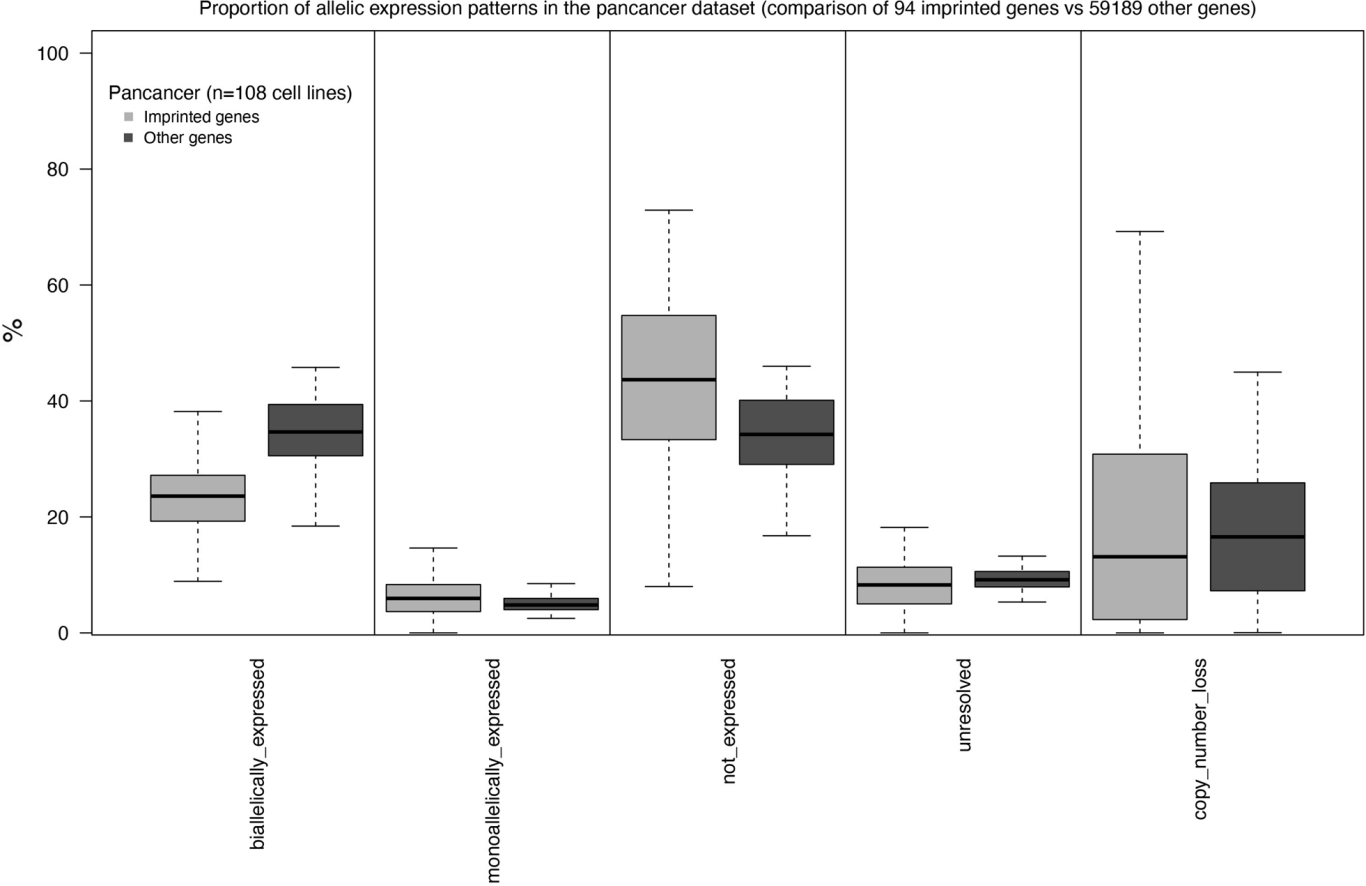
Transcriptome-wide allelic expression patterns of genes with heterozygous SNVs in CCLE data among 108 CCLE cell lines. Shown are proportions of allelic expression patterns for the genes and cell lines with available heterozygous SNV data allowing to make the inference. Boxplots of the 94 imprinted genes listed in Additional file 2: Table S1 are represented by a lighter shade of gray (left). Boxplots of the other genes representing the remaining 59,189 genes not included in the original list of 94 imprinted genes are shown by a darker shade of gray (right)

Figure 2 provides a comparison, across the 108 pancancer cell lines, of the gene-level summary counts of allelic expression patterns in the 94 imprinted genes vs 59,189 remaining genes, using only the informative heterozygous SNV data (Fig. 1). Additional file 10: Fig. S5 provides additional detailed comparisons for annotated transcript features (genes, isoforms, and individual exons) of the 94 imprinted genes vs all remaining genes within individual tumor types, whereas Additional file 11: Fig. S6 shows comparisons, at the gene level, of allelic expression patterns of the 94 imprinted genes vs the average values of 94 subsampled genes.

Detailed information about gene-level allelic expression patterns of the 94 imprinted genes is provided in Additional file 12: Table S6, with select examples listed in Table 1. Additional file 13: Fig. S7 shows the heatmap of tumor histology-specific proportions of monoallelically expressed imprinted genes. All features (genes, isoforms, and individual exons) of the 94 imprinted genes had a higher proportion of monoallelic and a lower proportion of biallelic expression patterns than the remaining 59,189 genes (Fig. 2; Additional file 10: Fig. S5; Additional file 14: Table S7). Based on Additional file 14: Table S7, when comparing counts of genes with monoallelic vs those with biallelic expression patterns (after excluding genes with no SNV data, copy number loss, lack of expression, or unresolved cases), 21.9% of the 94 imprinted genes had monoallelic expression across the 108 cell lines, and 78.1% had biallelic expression. In contrast, among the genes other than the 94 imprinted genes, for which mono- or biallelic expression could be identified, 13.5% were monoallelically expressed, and 86.5% were biallelically expressed. Higher prevalence of monoallelic expression and lower prevalence of bialleic expression of the 94 imprinted genes as compared to the remaining genes were observed not only across the 9 tumor histologies, but also within individual cancer types, even though both the 94 imprinted gene loci and the remaining 59,189 transcripts represent biologically heterogeneous groups. The 94 genes that are imprinted in the normal tissues include genes with isoform-specific (e.g., the *GNAS* locus), tissue-specific (e.g., *BLCAP, DNMT1,* and *PHLDA2*), and polymorphic (e.g., *IGF2R* and *HM13*) imprinting [2, 17–20, 25, 27, 28, 47, 81, 82, 131]. Such genes showed a lower proportion of monoallelic vs biallelic expression in cancer cell lines than some other imprinted genes, e.g., *SNRPN, SGCE, NLRP2, H19,* and *ANO1* (Table 1).

**Table 1:** Gene-level allelic expression patterns for select examples of 94 imprinted genes in 108 CCLE cell lines

Gene	Chr	Cell line counts of allelic expression patterns							% monoallelically expressed
		CN loss	No SNV data	Not expressed	Biallelically expressed	Monoallelically expressed	Total biallelically or monoallelically expressed	Unresolved	
*SNRPN*	15	15	44	15	0	32	32	2	100.00
*SGCE*	7	5	42	20	14	27	41	0	65.85
*NLRP2*	19	6	19	44	12	23	35	4	65.71
*H19*	11	15	23	18	20	14	34	18	41.18
*ANO1*	11	1	41	29	20	11	31	6	35.48
*MEST*	7	11	54	5	22	12	34	4	35.29
*GRB10*	7	6	16	22	41	18	59	5	30.51
*PHACTR2*	6	18	23	10	36	12	48	9	25.00
*OSBPL5*	11	13	19	5	51	16	67	4	23.88
*HM13*	20	1	58	0	41	8	49	0	16.33
*DGCR6*	22	14	40	7	36	7	43	4	16.28
*RBP5*	12	7	36	17	29	4	33	15	12.12
*GNAS*	20	0	11	0	86	11	97	0	11.34
*DNMT1*	19	13	21	0	67	7	74	0	9.46
*UBE3A*	15	15	36	0	53	4	57	0	7.02
*BLCAP*	20	3	42	0	58	4	62	1	6.45
*PHLDA2*	11	14	54	3	33	2	35	2	5.71
*ZNF331*	19	6	32	17	39	2	41	12	4.88
*ZFAT*	8	6	17	9	43	2	45	31	4.44
*INPP5F*	10	13	41	2	44	2	46	6	4.35
*SLC22A18*	11	14	19	4	54	2	56	15	3.57
*NAA60*	16	13	36	0	57	2	59	0	3.39
*ZC3H12C*	11	16	31	8	36	1	37	16	2.70
*IGF2R*	6	19	16	2	69	1	70	1	1.43

Shown are cell line counts of gene-level expression patterns for those of the 94 imprinted genes which had > 30 combined monoallelic or biallelic calls in 108 cell lines. Cell line counts of gene-level expression patterns for all 94 imprinted genes in 108 cell lines are provided in Additional file 12: Table S6 at the gene level and in Additional file 17: Table S9 at the isoform level

Chr, chromosome; CN, copy number; % monoallelically expressed indicates the proportion (%) of monoallelic calls out of Total monoallelically or biallelically expressed, which represents the combined counts of monoallelic and biallelic calls across 108 cell lines

The 59,189 remaining genes are also heterogeneous, and some of them were predominantly monoallelially expressed. Additional file 6: Table S4 shows gene-level allelic expression patterns of the top 60 additional genes with predominantly monallelic expression that were not a part of the initial list of 94 imprinted genes. Additional file 16: Fig. S8 provides tissue-specific comparisons of gene, isoform, and exon expression of the 60 predominantly monoallelically expressed genes listed in Additional file 6: Table S4. For comparison, Additional file 15: Table S8 shows gene-level allelic expression of the 7 control genes, none of which are imprinted in the normal tissues.

A number of imprinted tumor suppressor genes such as *RB1, TP73,* and *CDKN1C* showed copy number loss or the loss of expression in many tumor cell lines (Additional file 12: Table S6). At least one copy of these genes was lost in 21, 20, and 14 cell lines, respectively. In the cell lines which had both gene copies and in which heterozygous SNVs were present for allelic analysis, *TP73* expression was lost in 27 cell lines, and *CDKN1C* expression was lost in 4 cell lines. These findings are consistent both with previously documented frequent copy number loss of *RB1, TP73,* and *CDKN1C* in tumors and with disruption of their expression through a variety of epigenetic regulatory mechanisms including, e.g., changes in promoter methylation, LOI, histone modulation, and regulation by ncRNA [47, 48, 52, 76].

Detailed information about allelic patterns of expression of each isoform of the imprinted genes in each tumor category and in the pancancer dataset is provided in Additional file 17: Table S9. Some imprinted genes and isoforms showed tumor type-specific allelic expression patterns. For example, tumor-specific and isoform-specific allelic expression patterns were observed for *PLAGL1* (*ZAC1*, Additional file 17: Table S9; Additional file 18: Fig. S9) which encodes an important transcriptional factor and nuclear receptor co-activator, which is involved in cancer progression and resistance to treatment [1, 2, 8, 132]*. PLAGL1* isoforms were biallelically expressed in multiple AML cell lines (18 isoforms) and monoallelically expressed in colorectal cell lines (22 isoforms), whereas this gene had isoform-specific allelic patterns in head and neck tumor cell lines, with 5 monoallelically and 10 biallelically expressed isoforms (Additional file 17: Table S9; Additional file 19: Table S10). These differences in isoform expression patterns, among the tissues where such patterns could be identified, were statistically significant (*p* = 8.978 × 10^–10^; Additional file 19: Table S10). While each *PLAGL1* isoform with established allelic expression was either monoallelically or biallelically expressed within each of these tumor histologies, 18 isoforms had both monoallelic and biallelic expression patterns across all tumor categories combined (Additional file 19: Table S10). These results suggest isoform-specific regulation of *PLAGL1* expression in different tumor histologies. They are consistent with reports of different promoters regulating *PLAGL1* isoform-specific expression in the normal human tissues, of biallelic *PLAGL1* expression in peripheral blood leukocytes and pancreatic tissues, and its low level of biallelic expression in other adult tissues [133]. In addition, multiple cell lines from several tumor categories showed *PLAGL1* copy number loss or the loss of expression of its multiple isoforms in our dataset (Additional file 17: Table S9)*.* These findings are consistent with previously reported changes in *PLAGL1* expression in malignant cells, including the loss of *PLAGL1* expression in adult tumors and upregulation of its expression in embryonic tumors [1, 8]. In contrast with cancer cells, this gene has been reported to be imprinted in many normal tissues [66].

Consistent with the complexity of imprinting regulation and molecular aberrations in cancer, each imprinted gene had its own unique patterns of isoform expression in cell lines from different tumor histologies. For example, the imprinted genes *L3MBTL1* and *SGK2* are both located at 20q13.12 and are frequently co-deleted in myeloid malignancies [63, 97, 134–136]. Many *SGK2* isoforms were not expressed in a large number of AML, breast, ovarian, PDAC, and SCLC cell lines, as compared to a much smaller number of *L3MBTL1* isoforms that were not expressed in these tumor histologies (Additional file 12: Table S9). The number of cell lines with monoallelic and biallelic expression isoform expression was also specific to each gene. Both *L3MBTL1* and *SGK2* had only monoallically expressed isoforms in AML (Additional file 12: Table S9B); however, the *SGK2* isoforms with informative data that were expressed in SCLC showed only monoallelic expression, whereas *L3MBTL1* had isoform-specific expression in SCLC cell lines (Additional file 12: Table S9J). Some *L3MBTL1* isoforms with informative data were only monoallelically expressed in SCLC, others were only biallelically expressed, and additional *L3MBTL1* isoforms were monoallelically or biallelically expressed in different SCLC cell lines.

*IGF2* and *H19,* which belong to an imprinted gene cluster at 11p15.5, were also monoallelically expressed in some cell lines and biallelically expressed in others, both at the isoform and whole gene levels (Additional file 12: Table S6, Additional file 17: Table S9). This is consistent with frequent loss of imprinting of *IGF2* and occasional LOI of *H19* in tumors, with the resulting biallelic expression leading to increased cell growth, proliferation, and metabolic activity [1, 2, 4, 8, 53, 137].

The *GNAS* locus encodes multiple transcripts, some of which are maternally or paternally imprinted in normal tissues, in a tissue-specific imprinting manner [2, 17–19]. Accordingly, we observed different expression patterns of different *GNAS* isoforms, including isoforms that had only monoallelic, only biallelic, predominantly biallelic expression, or cell line-specific monoallelic or biallelic expression patterns (Additional file 17: Table S9).

### Validation of expression patterns of imprinted genes using previously published information

Our findings of allelic expression patterns of imprinted isoforms in CCLE cell lines confirmed the RT-PCR results of Martin-Trujillo et al. [34], who analyzed gene-level molecular data from COSMIC cell lines. Three breast (HCC1143, HCC1954, and MDAMB436) and one colorectal (HT55) cell lines were analyzed in that study and were also analyzed in our project using independently generated CCLE/DepMap data [69–71]. Table 2 shows the concordance of gene-level expression patterns between both studies. Detailed information about allelic expression of each of the 139 individual isoforms of the genes profiled by both studies is shown in Additional file 20: Table S11. Among all genes and cell lines that were profiled by both studies, only one non-protein-coding isoform (ENST00000475188.1 of *MEST* in HT55, annotated as MEST-215 in Ensembl; Table 2 and Additional file 20: Table S11) showed monoallelic expression in CCLE, as opposed to biallelic expression at the gene level in that cell line in COSMIC [34]. All other *MEST* isoforms with informative data were biallelically expressed in the CCLE cell line HT55, indicating isoform-specific imprinting of *MEST* in cancer*,* consistent with an earlier report by Pedersen et al. [138]. These findings demonstrate the stability of allelic expression patterns of imprinted genes between the CCLE and COSMIC stocks of the 4 cell lines that were independently profiled.

**Table 2:** Examples of genes not included in the initial list of 94 imprinted genes which had predominantly monoallelic expression in CCLE cell lines

Gene	Chr	Cell line counts of allelic expression patterns						% monoallelically expressed	Previous reports of MAE mechanism	References
		CN loss	No SNV data	Not expressed	Biallelically expressed	Monoallelically expressed	Unresolved			
*SEC22B*	1	11	0	0	4	93	0	95.88	Likely imprinted	[122, 142]
*AP3S1*	5	13	4	0	12	79	0	86.81	RMAE under MeCP2 control in mouse, unresolved in human	[140]
*GCSHP5*	1	0	6	0	23	79	0	77.45	Near imprinting control region	[11]
*PRIM2*	6	11	0	2	26	69	0	72.63	Conflicting	[80, 81, 94, 96, 122, 139, 140, 143]
*BCLAF1*	6	17	1	0	25	65	0	72.22	Likely imprinted	[121, 141]
*SF3B5*	6	18	18	0	8	64	0	88.89	Conflicting; located immediately near human imprinted genes *PLAGL1* and* HYMAI*	[32, 144, 145]
*MAP2K3*	17	26	0	0	26	56	0	68.29	Likely imprinted or technical artifact	[66, 122, 140]
*CDC27*	17	4	0	0	52	52	0	50.00	RMAE under MeCP2 control	[140]
*GRK2*	11	1	19	0	37	51	0	57.95	Skewed MAE due to a promoter SNV	[123]

Shown are gene-level expression calls for selected genes with > 50 monoallelic counts and ≥ 50% of monoallelic calls out of all calls 108 cell lines. Additional examples of genes with predominantly monoallelic expression in CCLE cell lines are provided in Additional file 6: Table S4. Chr, chromosome; CN, copy number; MAE, monoallelic expression; RMAE, random monoallelic expression; % monoallelic indicates the proportion (%) of monoallelic calls out of total monoallelic or biallelic, which represents the combined counts of monoallelic and biallelic calls across 108 cell lines

### Additional genes with predominantly monoallelic expression

Multiple genes not included in the initial list of 94 imprinted genes demonstrated a strong preference for monoallelic expression (Additional file 6: Table S4; Additional file 18: Fig. S8). Several of them, e.g., *BCLAF1, MAP2K3, SEC22B,* and *PRIM2,* had been reported in multiple studies as likely to be imprinted (Table 3) [80, 81, 121, 122, 139–142]. However, the imprinting status of *PRIM2* has not been resolved. Some studies reported that this gene was not imprinted in the normal placenta or white blood cells [94, 143], whereas another study suggested its allelic expression in fibroblasts and lymphoblastoid cell lines was due to mechanisms other than imprinting [96]. Another study reported monoallelic expression of *MAP2K3* in lymphoblastoid cell lines but excluded it from imprinted genes after genotype filtering [66]. In our analysis, *PRIM2* and *MAP2K3* had monoallelic expression patterns in the majority of the cancer cell lines from 9 tumor categories.

**Table 3:** Concordance among gene-level allelic expression patterns of imprinted genes in CCLE cell lines analyzed in our study with previously reported results in matching COSMIC cell lines [34]

Gene	Breast			Colorectal
	HCC1143	HCC1954	MDAMB436	HT55
*H19*		Biallelic		
*MEST*				Biallelic expression of all isoforms except one monoallelic isoform ENSG00000106484.16
*PEG10*		Monoallelic		
*PEG3*	Not expressed	Not expressed	Not expressed	Not expressed

Listed are imprinted genes and cell lines which were profiled in both studies, based on the computational analysis of CCLE cell lines in our study, and on RT-PCR results of matching COSMIC cell lines in the study of Martin-Trujillo et al. [34]

A methylome-based study of human germ layer cells and gametic cells found an ICR with gametic origin of methylation near *GCSHP5* [11], which was predominantly monoallelically expressed in our data. Interestingly, *SF3B5,* which was also predominantly monoallelically expressed in our analysis, is located at 6q24 in close proximity to two imprinted genes, *PLAGL1* and *HYMAI* [144, 145]*.* In contrast with these genes, *SF3B5* was reported to be biallelically expressed in the first trimester embryonic and placental tissues [145]. However, *SF3B5* was also found to have noncanonical imprinting with a preference for maternal allele expression in the arcuate nucleus cells of the mouse brain [32]. Our results demonstrate that *SF3B5* is monoallelically expressed in many tumor cell lines, suggesting that it may be imprinted or monoallelically expressed due to other mechanisms in these cell lines.

Some genes in Additional file 6: Table S4 are likely predominantly monoallelically expressed due to mechanisms other than imprinting. Such mechanisms may include, e.g., the presence of regulatory SNV variants in promoters or other regulatory regions, resulting in uneven transcription of both alleles or in complete abrogation of expression of one of the alleles [146]. Consistent with this mechanism, one of the top monoallelically expressed genes in our study, *GRK2* was previously reported to be monoallelically expressed due to an SNV polymorphism (rs182084609) in the *GRK2* promoter, which affects the binding of the EGR-1 transcription factor [123]. Random monoallelic expression (RMAE) can provide another mechanism of monoallelic expression [147]. Brousseau et al. reported RMAE under the control of MeCP2 for *CDC27* in human and mouse cells and for *AP3S1* in the mouse, with human *AP3S1* expression patterns reported as undetermined [140]. Both *CDC27* and *APS31* were predominantly monoallelically expressed in our dataset (Additional file 6: Table S4).

### *Association among isoform allelic expression patterns, expression levels, and *in vitro* drug response*

We analyzed associations of in vitro drug response with allelic expression patterns of isoforms of the 94 imprinted genes from the initial list (Additional file 2: Table S1) and 60 additional genes with predominantly monoallelic expression (Additional file 6: Table S4). Isoforms with identical allelic expression patterns were grouped. Table 4 summarizes the results for those genes whose isoform groups had the strongest associations with drug response, with *p*_FDR_ < 0.05. Violin plots showing associations between agents and expression patterns of the isoform groups satisfying *p*_FDR_ < 0.05 are provided in Additional file 21: Fig. S10 for the 94 imprinted genes and in Additional file 22: Fig. S11 for 60 additional predominantly monoallelic genes. Detailed information about the association of each individual isoform group using a more relaxed threshold of *p*_FDR_ < 0.125 is provided in Additional file 23: Table S12. Drug response was associated with allelic expression patterns of multiple isoform groups, e.g., those in imprinted *GNAS, GRB10, H19, NAA60, OSBPL5, DGCR6, PHACTR2, ZFAT, DNMT1, CPA4,* predominantly monoallelically expressed *MAP2K5* and *BCLAF1,* and other genes. The direction of these associations was specific to individual genes and agents. For example, sensitivity to the mitotic disruptors alisertib (an Aurora kinase A inhibitor) and vindesine was associated with biallelic expression of isoforms of *GTPBP2,* a non-imprinted, predominantly monoallelically expressed gene (Additional file 6: Table S4). Such associations may be due to GTPBP2 role in the regulation of Wnt signaling [148]. Sensitivity to the DNA damaging agent (DDA) oxaliplatin was associated with biallelic expression of the imprinted gene *ZFAT,* whose overall expression had been reported to be a part of a prognostic gene signature for another platinum compound, cisplatin [149]. Biallelic expression of several isoforms of *CDC27,* which encodes a core component of the Anaphase-promoting complex/cyclosome regulating the cell cycle, was associated with sensitivity to the PARP inhibitor PARP_9495, the topoisomerase I inhibitor SN-38, and the Aurora kinase inhibitor SNS-314, consistent with earlier reports of regulation of the CDC27 gene or protein by PARP inhibitors and by SN-38, of direct interactions between CDC27 and Aurora kinase B, and of CDC27 importance in cancer and in response to treatment, notably to DNA damage and mitotic checkpoint inhibition [150–154]. Interestingly, sensitivity to the DDA topotecan was associated with monoallelic expression of the ENST00000392348.6, ENST00000529826.5, and ENST00000628517.2 isoforms of the predominantly monoallelically expressed gene *BCLAF1,* whereas sensitivity to another DDA, gemcitabine, was associated with biallelic expression of its ENST00000533422.5 isoform (Table 4 and Additional file 23: Table S12). BCLAF1 is directly involved in DNA damage response, and its loss has been linked to DDA sensitivity [155]. It remains to be investigated whether the opposite directions of associations of *BCLAF1* isoforms may be due to differences in the regulation of isoform expression, or whether they could be due to different mechanisms of DNA damage by gemcitabine and topotecan. Other notable associations with drug response included *GNAS* isoforms*,* whose allelic patterns were associated with response to multiple inhibitors of signaling pathways, e.g., with resistance to the BRAF inhibitor HG6-64–1, XAV939 which inhibits the Wnt signaling pathway component tankyrase and stimulates β-catenin degradation, and the TGFβ receptor 1 (ALK5) inhibitor SB52334, and with sensitivity to the EGFR family inhibitors afatinib and PD-153035. The *GNAS* locus encodes several maternally and paternally imprinted isoforms, along with biallelically expressed isoforms [2, 19, 64, 156]. *GNAS,* which frequently acquires activating mutations in cancer, encodes the G-protein Gα subunit that participates in G-protein signaling and activates downstream signaling pathways, including direct interactions with MAPK signaling [156, 157].

**Table 4:** Imprinted and other genes with predominantly monoallelic expression genes whose isoform groups had the strongest associations of allelic expression with in vitro drug response with *p*_FDR_ < 0.05

Gene	Category	Associated agents
*Associations where cells with monoallelic expression were more sensitive*		
*DNMT1*	Imprinted	AZD2014, PP242
*GNAS*	Imprinted	50,869, afatinib, combretastatin-A-4, N24798-49-A1, PD-153035
*GRB10*	Imprinted	Repsox
*H19*	Imprinted	PP242
*NAA60*	Imprinted	SDZ-WAG-994
*OSBPL5*	Imprinted	N22899-6-C1
*AC009245.3*	Other monoallelic	BMS-387032, CX-5461, YM-155
*AC024592.12*	Other monoallelic	WZ-3146
*BCLAF1*	Other monoallelic	3-deazaneplanocin-A, BAY-11–7082, fosbretabulin, genz-644282, lestaurtinib, mefexamide, PHA-793887, PP-1, SB-939, SNX-2112, topotecan, VER-49009
*C1D*	Other monoallelic	LFM-A13, linsitinib, VX-11e
*CDC27*	Other monoallelic	CHIR-124, sirolimus
*COMMD6*	Other monoallelic	AS605240, CX-5461, masitinib, MIM1, NSC-207895, pelitinib, PHA-793887, QL-XI-92, TPCA-1
*GRK2*	Other monoallelic	Triapine
*GTPBP2*	Other monoallelic	FMK
*MAP2K3*	Other monoallelic	AS605240, AT7867, irinotecan, QS11, talazoparib, VS-4718
*MROH8*	Other monoallelic	Poziotinib
*PRIM2*	Other monoallelic	GSK269962A
*RP11-152F13.10*	Other monoallelic	Embelin
*RP11-680G24.6*	Other monoallelic	Niraparib
*RPL7AP31*	Other monoallelic	Mexiletine
*SUOX*	Other monoallelic	AP1903
*Associations where cells with biallelic expression were more sensitive*		
*CPA4*	Imprinted	TAS-103
*DGCR6*	Imprinted	VE-822
*GNAS*	Imprinted	HG6-64–1, JQ1, KIN001-270, luminespib, NSC-207895, SB52334, SNX-2112, XAV939
*IGF2R*	Imprinted	CGS-15943
*NAA60*	Imprinted	Cyclovalone
*OSBPL5*	Imprinted	Osimertinib
*PHACTR2*	Imprinted	Plinabulin
*ZFAT*	Imprinted	Oxaliplatin
*AC009245.3*	Other monoallelic	Osimertinib, P276-00
*BCLAF1*	Other monoallelic	ARRY-334543, BIBF-1120, dasatinib, fluvastatin, FTI-277, gemcitabine, sapitinib, torin 2, WEE1 inhibitor
*CBX4*	Other monoallelic	NVP-TAE684
*CDC27*	Other monoallelic	FY026, LY456236, NVP-TAE684, PARP_9495, SN-38, SNS-314
*GCSHP5*	Other monoallelic	N22899-6-C1
*GRK2*	Other monoallelic	I-BET-762
*GTPBP2*	Other monoallelic	Alisertib, captamine, vindesine
*NDUFV2P1*	Other monoallelic	FY026
*RPL12P4*	Other monoallelic	AZD7969
*RPL7AP31*	Other monoallelic	Ispinesib mesylate, oxaliplatin, SB-216641, ZM447439

Shown are genes whose isoform groups were associated with drug response with *p*_FDR_ < 0.05 (corresponding to *p*_0_ < 3.4 × 10^–5^). Gene category indicates whether a gene was included in the initial list of 94 imprinted genes (Imprinted, Additional file 2: Table S1 and Additional file 12: Table S6), or if that gene was one of the top 60 additional genes with predominantly monoallelic expression (Other monoallelic, Additional file 6: Table S4)

Detailed information about associations of each individual isoform group is provided in Additional file 23: Table S12

Additional file 24: Table S13 shows the strongest Spearman correlations between mean overall expression levels of isoform groups and drug response with *p*_FDR_ < 0.125 (corresponding to the absolute value of the correlation coefficient between 0.6485 and 0.9648). These associations of expression levels of isoform groups were consistent our previous finding of strong associations of drug response with whole gene-level associations of multiple imprinted genes, e.g., *HM13, PHLDA2, CPA4, DNMT1, DDC,* and *SLC22A18* [63]*.* Among examples of consistent associations at both isoform and whole gene levels, elevated expression of *HM13* was associated with axitinib resistance, and that of *CPA4,* whose product is involved in the histone deacetylation pathway, with resistance to HDAC inhibitors. Our isoform analysis also showed strong associations of drug response with expression of additional imprinted genes e.g., *KCNK9, CDKN1C, SGCE, INPP5F, WT1,* and *LRRTM1.* In our previous study of gene-level expression [63], many of them were weakly associated with drug response but did not reach statistical significance (data not shown), suggesting an improved resolution when using isoform level data. We also observed multiple associations of drug response with isoform expression of predominantly monoallelically expressed genes, e.g., *MAP2K3, SEC22B*, and *CDC27* (Additional file 24: Table S13).

Previously we reported associations of cancer drug response with copy number, DNA methylation, and overall expression at the whole gene level for multiple imprinted and non-imprinted genes at 20q11-q13.32 [63]. In our current analysis at a higher resolution using isoform data, cell line response to drug treatment was associated not only with the overall isoform expression levels of the imprinted genes *HM13* and *GNAS-AS1* (Additional file 24: Table S13), but also with allelic patterns of isoform expression of several genes in that chromosomal region, including the complex *GNAS* locus at 20q13.32, *MROH8* at 20q11.23, and the pseudogenes *FRG1BP* at 20q11.1 or 20q11.1-q11.21 and *RPL12P4* at 20q13.2 (Table 4; Additional file 23: Table S12)*.* These associations support the likely importance of complex tumor molecular alterations involving the 20q11-q13.32 region in drug response.

Only a few isoform groups of imprinted and predominantly monoallelically expressed genes showed significant associations between their allelic expression patterns and overall expression levels (Additional file 25: Table S14, *p*_FDR_ < 0.125). Such associations were observed for the isoform groups of the imprinted genes *DGCR6, DNMT1, H19, IGF2R, ZFAT,* and *ZNF331,* and other predominantly monoallelically expressed genes: *BRD8, C1D, FADS2, GRK2, MAP2K3,* and *MAD2L2.* Most of them were expressed at significantly lower levels in those cell lines where they were monoallelically expressed, suggesting allele dosage effects. However, one isoform of *C1D* and three isoforms of the imprinted gene *H19* had higher expression in the cell lines where they were monoallelically expressed. The opposite direction of *H19* association is consistent with an earlier report of transcriptional upregulation of *H19* in neuroendocrine prostate cancer via a mechanism other than imprinting [62].

### Analysis of base expression preference of heterozygous SNVs in monoallelically expressed features

Additional file 26: Table S15A and S15B provides counts of monoallelic base expression for heterozygous SNVs in the 154 imprinted or predominantly monoallelically expressed genes and their isoforms. Shown is the number of cell lines with monoallelic expression of each base (A, C, T, or G) for the SNVs that were determined to be heterozygous from WES data. Additional file 27: Table S16A provides the results of the two-sided binomial statistical testing for preferential monoallelic expression of the hg19 reference base vs any non-reference base for each of those 474 SNVs that were expressed in > 5 cell lines, based on an assumption of the 50% probability of expression of the reference allele. These tests are summarized at the gene level in Additional file 27: Table S16B and include 130 genes. The summary of the SNV categories that reached statistical significance for base preferential expression (*p* < 0.05) vs those that were not statistically significant (*p* > 0.05) is shown in Additional file 28: Table S17.

All WES data for the heterozygous SNVs had an equal 50/50 split between the reference and non-reference alleles. 98% of SNVs in the RNA-seq data had only one non-reference type. For the 2% SNVs in monoallelically expressed genes that had more than one non-reference allele in the WES data, one of the non-reference bases had a substantially stronger representation. Additional file 26: Table S15A shows such multi-allelic SNVs in *PRIM2, RP3-401D24.1, EIF4HP1, GRB10, PSPC1P1, CDC27,* and *FRG1BP*. In the RNA-seq data of monoallelically expressed genes, 90% of SNVs showed a preference for monoallelic expression of one or the other base type.

A much smaller proportion (33%) of SNVs imprinted genes showed a statistically significant preference for expression of a single base (16 significant vs 32 non-significant, Additional file 27: Table S16), as opposed to 95% SNVs with significant bias toward a specific base in other monoallelically expressed genes (405 significant SNVs vs 21 non-significant in other genes). The difference between imprinted and other monoallelically expressed genes was highly significant (*p* < 10^–15^), which is consistent with the imprinting mechanism, when an allele is expressed or silenced due to its parental origin without regard to the presence of a specific base. The preference of SNVs in other monoallelically expressed genes for expression of a specific base may indicate that mechanisms other than imprinting drive monoallelic expression of many of them.

Analysis of integrated SNV data at the gene level (Additional file 27: Table S16B) showed that 3 imprinted genes (*NLRP2, SGCE,* and *GRB10*) had multiple SNVs with significant monoallelic expression of a single base, and 6 additional imprinted genes (*GNAS, OSBPL5, DGCR6, MEST, DDC,* and *SGK2*) had 1 such SNV. The finding of SNVs with preferential expression of a specific base in imprinted genes may suggest additional mechanisms of transcriptional and post-transcriptional regulation. For example, the only SNV with significant preferential base expression in *GNAS* was the C > T polymorphism rs7121 (Additional file 27: Table S16A). It is located in the biallelically expressed Gsα transcript of the *GNAS* locus and is not a part of other, imprinted *GNAS* transcripts [158]. The non-reference T allele, the only expressed allele of that SNV in the RNA-seq data in this dataset, may extend the mRNA stability [158–160], suggesting a non-imprinting mechanism for the prevalence of the T allele in the RNA-seq data.

Multiple predominantly monoallelically expressed genes, which were not included in the initial list of the 94 imprinted genes, contained many SNVs with significant preference for expression of a specific base (Additional file 27: Table S16A). Among them, *MAP2K3, PRIM2, RP3-401D24.1, BCLAF1, FRG1BP, CDC27, RP4-592A1.2, SEC22B, SEC22B3P, SEC22B2P, PPIAP22, GCSHP5, NDUFV2P1,* and *RPL23AP2* each had  > 7 significant SNVs, suggesting a likely role of mechanisms other than imprinting in their monoallelic expression. *MAP2K3* had 48 significant SNVs, *PRIM2* had 44, *BCLAF1* had 23, and *GCSHP5* had such 9 SNVs, indicating potential alternative mechanisms for their preferential allele expression bias. Given the prior reports of potential imprinting or conflicting imprinted status of these genes [11, 80, 81, 94, 96, 121, 122, 139–141, 143], such alternative mechanisms may take place instead of or in addition to regulation of their monoallelic expression via imprinting. In contrast, since only one significant SNV was present in *SF3B5,* which also had conflicting reports about its imprinted status [32, 144, 145], its predominantly monoallelic expression may be consistent with imprinting.

## Discussion

We developed a computational pipeline that utilizes transcriptomic, WES, and copy number information to conduct a comprehensive study of allelic expression patterns of imprinted genes and other protein-coding and noncoding transcripts. We observed tissue-specific and isoform-specific expression patterns of multiple imprinted genes in cancer cell lines that were consistent with previous reports of such patterns, e.g., LOI of *H19* [1, 2, 4, 8, 34, 137]. While several earlier studies investigated allelic expression or changes in methylation of imprinted genes in cancer at the whole gene level [33, 34, 40, 41, 45], this is the first study to evaluate transcriptome-wide fine scale allelic expression of all annotated genes, isoforms, and individual exons. We also conducted a novel systemic evaluation of associations of cancer response to drug treatment with allelic expression patterns of isoforms of imprinted genes and other predominantly monoallelically expressed genes.

Allelic expression patterns of genes and individual isoforms observed in our study for breast and colorectal CCLE cancer cell lines were in agreement with the findings of Martin-Trujillo et al. [34] who analyzed cell lines from the COSMIC project. These findings provide a strong support for our algorithm, which is based on RNA-seq and WES data. They also indicate the stability of allelic expression patterns and of expression levels of the cell lines that are included in separate CCLE and COSMIC data collections, with molecular data for the latter now being available from the Cell Line Passports project at the Sanger Institute [161].

We analyzed allelic expression patterns in cell lines from a variety of cancer histologies, some of which have frequent amplifications or deletions of different genome regions containing imprinted genes, e.g., *ANO1* at 11q13, multiple imprinted genes at 20q11-q13.32, *RB1* at 13q14.2, and genes in other regions [34, 63, 76, 97, 134, 135, 162–164]. We utilized whole gene copy number data and distinguished the loss of at least one gene copy from monoallelic expression when both copies of a gene were present. Small germline microdeletions, microinsertions, and chromosomal rearrangements within *GNAS* occur in patients with pseudohypoparathyroidism type 1a and type 1b [165, 166]. Such intragenic events, if they also occur in tumors, may alter the copy number of individual exons and may need to be considered in future investigations. Additionally, some imprinted genes may participate in fusions, e.g., in astroblastomas [51]. Although we did not analyze the fusion events, our results provide a comprehensive catalog of allelic expression patterns of all annotated exons, which may serve as a resource for future studies of allelic expression of gene fusions or intragenic molecular alterations.

Multiple genes, isoforms, and exons that were not a part of the initial list of 94 imprinted genes were predominantly monoallelically expressed across 108 cancer cell lines (Additional file 6: Table S4). While a number of them, e.g., *BCLAF1, MAP2K3, SEC22B,* and *PRIM2,* had been suggested to be imprinted in humans [80, 81, 121, 122, 139, 142], they contained multiple SNVs with a preference for expression of a specific base (Additional file 27: Table S16B), which suggests that their monoallelic expression may be at least in part due to mechanisms other than imprinting. *GCSHP5* and *SF3B5* were also predominantly monoallelically expressed. While their genome co-localization with other imprinted genes and/or ICRs could suggest their imprinting [11, 32, 144, 145], imprinting may be more likely for *SF3B5,* which had only 1 SNV with predominant expression of a specific base, than for *GCSHP5,* which had 9 such SNVs. Monoallelically expression of some other genes, e. g., *GRK2, CDC27,* and *AP3S1,* is likely to be due to non-imprinting mechanisms, e.g., RMAE or the presence of sequence variants in regulatory regions [123, 140]. It is likely, however, that the list of 60 predominantly monoallelically expressed genes in Additional file 6: Table S4 includes some imprinted genes which had not been previously reported. Of note, multiple genes that were predominantly monoallelically expressed in tumor cell lines encode important epigenetic factors (e.g., CBX4 and BRD8), signaling cascade components (MAP2K3), cell cycle regulators (CDC27), and factors involved in DNA damage repair and maintenance of chromosomal stability (e.g., C1D and MAD2L2, or REV7) [151, 152, 167–171].

We focused our analysis on the list of 94 imprinted genes in Additional file 1: Fig. S1. It did not include some additional imprinted genes analyzed in our earlier study at the whole gene level [63]. Among excluded genes were two protein-coding genes *AIM1* (*CRYBG1,* placentally imprinted) [82] and *L3MBTL* (*L3MBTL1*), pseudogenes *TCEB3C* (*ELOA3B*) and *PSIMCT-1* (*MCTS2,* or *MCTS2P*), which were annotated under differing synonym names, and additional ncRNA genes in the *miR-379*/*miR-656* (C14MC) cluster at 14q32.31 (the miRNA genes *MIR134, MIR379, MIR409, MIR410, MIR487B,* and *MIR656*) [91], in the Prader–Willi Syndrome (PWS) region at 15q11.2 (*IPW, PAR1, PAR5,* and multiple C/D small nucleolar RNAs (SNORD) genes) [85], and in other genome regions (*GNAS1-AS, HYMAI, IGF2-AS, MESTIT1, MIR184, MIR296, MIR298, MIR371A, MIR483, MIR517A, MIR675, WT1-AS,* and *RNU5D-1*)*.* No match in GENCODE 38 annotation could be found for 11 of these ncRNA genes. The results for the 92 additional genes (*CRYBG1, L3MBTL1, ELOA3B, MCTS2P,* and additional ncRNA genes) that were matched to the GENCODE 38 annotation under any of the synonym names are available in the supplementary data as part of the whole transcriptome analysis of the 59,283 autosomal genes. None of these 92 genes were among the 60 predominantly monoallelically expressed genes identified in our current study. Furthermore, monoallelic or biallelic expression patterns could not be inferred in any of the 108 cell lines for 85 out of these 92 genes, since they were not expressed, had a copy number loss, or had no SNV data across the cell lines.

Only a small number of imprinted and predominantly monoallelically expressed genes had isoforms whose allelic expression patterns were associated with isoform expression levels (Additional file 25: Table S14), suggesting diverse mechanisms of transcriptional regulation of such genes and the complexity of their downstream effects on drug response. Most genes in Additional file 25: Table S14 had significantly lower expression in cell lines with their monoallelic expression, suggesting potential gene dosage effects on expression. However, 1 isoform of the non-imprinted *C1D* and 3 isoforms of the imprinted *H19* had higher expression in the cell lines where they were monoallelically expressed, suggesting separate regulation of allelic expression and overall expression of *H19* and *C1D*. *H19* is an important negative regulator of *IGF2* expression, with important implications in cancer [1, 2, 4, 8, 137]. It encodes several coding and anticoding transcripts, some of which undergo LOI or transcriptional changes in cancer and have been linked to treatment resistance [62, 137]. Allelic expression of one of these transcripts (ENST00000447298) was also associated with drug response in our study, as were several other *H19* isoforms (Additional file 23: Table S12). Additionally, one *H19* isoform, ENST00000436715, whose monoallelic expression was significantly associated with elevated expression (Additional file 25: Table S14), has been implicated in important developmental and cancer-related processes, as it is upregulated in human bone marrow mesenchymal stem cells during osteogenic differentiation and is among most dysregulated lncRNAs in gallbladder cancer [172, 173]. Our results add to the body of evidence [62, 137] suggesting that expression of different *H19* isoforms in cancer may be regulated by different mechanisms, some of which lead to LOI and biallelic isoform expression, whereas others may upregulate monoallelically expressed isoforms.

We observed significant associations of allelic expression patterns of multiple imprinted genes and other predominantly monoallelically expressed genes with in vitro drug response. Due to small sample sizes in individual cancer categories (Additional file 3: Table S2), significant associations with drug response were identified using pancancer data. Therefore, they most likely represent biological mechanisms that may be common to different cancer types. Our results demonstrate that in addition to overall expression levels, cancer cell line response to different antitumor agents is affected by whether one or both allelic copies of specific isoforms are expressed (Table 4; Additional file 23: Table S12). Many associations of allelic patterns of isoform expression were observed for genes that were relevant to the mechanisms of action of the associated agents. For example, response to DDAs was associated with allelic patterns of *ZFAT, CDC27,* and *BCLAF1* isoforms*,* and response to inhibitors of multiple signaling pathways with expression patterns of *GNAS* isoforms. These results support earlier reports linking dysregulation of imprinting and non-imprinting mechanisms of allelic expression to cancer treatment response [45, 59, 174] and suggest potential mechanisms of drug resistance or therapeutic vulnerabilities. They may also suggest potential future combination therapies, e.g., with epigenetic agents to reactivate or upregulate the expression of isoforms associated with drug sensitivity, or with agents directly targeting products of the genes associated with therapy resistance. Given the high frequency of mutations in *GNAS* [156, 158], association of allelic expression patterns of imprinted and non-imprinted genes with drug response may involve not only allelic dose-specific effects, as suggested, e.g., for *IGF2* [58, 60], but also a potential interplay between the numbers of expressed mutated and non-mutated allele copies of isoforms of *GNAS* and other genes carrying functionally important variants.

While the agreement of the results from our algorithm with previous studies suggests the utility of our computational approach in using heterozygous SNVs to identify monoallelically expressed genes, it has some limitations. The inference of allelic expression patterns in malignant cells is complicated by the complexity of genetic and epigenetic alterations in tumors and potential technical artifacts. Even though the use of cancer cell lines eliminated potential complexities of mixed tissue expression that are present in patient tumor specimens, some epigenomic changes in this dataset may be unique to cancer cell lines and may not represent the biology of clinical specimens. Due to the absence of parental or matched normal tissue information, our algorithm was not designed detect the switching of imprinted alleles, which may occur in cancer [33, 36]. Our study also did not investigate allelic patterns of features expressed at very low levels, which were assigned the "not expressed" status. Following the assignment of allelic expression status, the accuracy of inference of the expressed base in SNVs of monoallelically expressed genes and isoforms may be reduced in the regions with the low depth of sequencing coverage. Additionally, the lack of heterozygocity in RNA-seq data of some genes could be due to cnLOH when a chromosomal segment originating from one parent is duplicated and the corresponding segment from another parent is lost without an overall change in the copy number, or aLOH, when amplification of one allelic copy is accompanied by the loss of another copy. cnLOH is frequent in germline imprinted disorders, affecting imprinted genes, and both cnLOH and aLOH are common in primary tumors and in cancer cell lines [8, 12, 34, 175, 176]. Some expression patterns that were identified as monoallelic by our algorithm (Fig. 1) may represent cnLOH or aLOH. This was documented earlier by Martin-Trujillo et al. [34], who used DNA methylation data to resolve patterns of expression under LOH. Consistent with previous studies of imprinted genes [33, 34], future expansion of our algorithm to incorporate DNA methylation may improve transcriptome-wide detection of allelic switching and cnLOH and would allow resolution of allelic patterns at very low expression levels. In addition, our analysis did not distinguish between biallelic and multi-allelic expression, which may occur in the event of gene amplification and subsequent mutation [34]. However, multi-allelic SNVs were rare in our analysis of imprinted and other predominantly monoallelically expressed genes (Additional file 26: Table S15A). While multi-allelic expression is suggestive of LOI, expression of multiple alleles may have nuanced biological effects. Copy number of imprinted genes was associated with drug response in our earlier investigation [63], suggesting a complex interplay of allelic expression, copy number, and sequence variation of imprinted genes in cancer.

The interplay between epigenetic changes and sequence variants can be very diverse and specific to different genes and isoforms. Some tumor suppressor genes, e.g., *RB1* and *CDKN1C*, can be downregulated or inactivated in cancer via promoter hypermethylation or copy number loss [52, 76, 177, 178], inactivating mutations in *RB1* [177] or imprinting allele switching of *CDKN1C* [52]. In contrast, *IGF2* is upregulated in many tumors through different mechanisms, e.g., LOI and methylation changes of regulatory sites, focal amplification, activation of fetal *IGF2* promoters, and WT1 and p53 loss, and its upregulation contributes to protumorigenic effects and drug resistance [53–55, 57, 179]. Our study did not examine the effects of SNV variants on gene function or expression levels, as our analysis did not distinguish between pathogenic and benign sequence variants. Future studies may incorporate additional effects of pathogenic SNV variants to examine the interplay of allelic expression patterns, copy number loss, and sequence variation of genes and isoforms.

In addition to imprinting, preferential occurrence of a single allele in the RNA-seq data could be caused by other factors, e.g., variation in the *cis-*regulatory elements, nonsense-mediated decay, and other mechanisms [130, 180, 181]. To better understand the potential mechanisms of monoallelic expression in cancer cell lines, we examined whether monoallelically expressed genes and isoforms had a predominant expression of a specific nucleotide. Our results (Additional file 27: Table S16 and Additional file 28: Table S17) indicate that mechanisms leading to observed monoallelic expression may be diverse and transcript specific. For example, we observed 7 copies of the T variant in the RNA-seq data and no expressed copies of the reference C base for the *GNAS* C > T polymorphism rs7121 at position 57,478,807 on chromosome 20 (Additional file 27: Table S16A). It represents a synonymous substitution in exon 5 of the biallelically expressed Gsα transcript of the *GNAS* locus, and it does not overlap with imprinted *GNAS* isoforms [158]. Earlier studies suggested that the presence of the reference C allele or the CC genotype is a biomarker for more favorable cancer outcomes, associated with improved response to chemotherapy and radiotherapy, as compared to worse outcomes in the presence of the alternative T allele or the TT genotype [158–160]. The T variant was predicted to affect mRNA folding and to result in longer mRNA stability than the reference C allele, and accordingly, the TT genotype carriers have elevated mRNA expression of Gsα [158–160]. Therefore, the presence of multiple T alleles and the absence of C alleles for rs7121 in the RNA-seq data are likely to result from faster degradation of mRNA transcripts containing the C base, rather than due to the switch of the *GNAS* Gsα transcript to monoallelic expression*.*

Some cases when heterozygous SNVs were absent in the RNA-seq data of non-imprinted genes or present in the RNA-seq data of imprinted genes, while being accompanied by discrepancies in the presence of heterozygous SNVs between RNA-seq and WES data (Fig. 1), could be due to post-transcriptional RNA editing, which is common both in normal and malignant cells [127–129]. For example, the adenosine-to-inosine (A-to-I) editing in familial esophageal squamous cell carcinoma has been described for *SLC22A3* [128]. This imprinted gene was analyzed in our study in cell lines from other tumor categories (Additional file 3: Table S2; Additional file 12: Table S6). It was biallelically expressed in 14 cell lines and monoallelically in 2 cell lines, and in 3 cell lines heterozygous SNVs were detected in this gene in the RNA-seq but not WES data (Additional file 12: Table S6).

Many imprinted genes influence tumor growth, proliferation, and cancer progression [1, 2, 4, 8, 76]. Our results provide novel information, at high resolution, about regulation of allelic expression of imprinted genes in specific tumor types and about allelic expression of non-imprinted genes with important roles in cancer. For example, numerous studies documented protumorigenic effects of LOI of *IGF2* in many cancer types [53–55, 57]. Consistent with these reports, in the tumor cell lines where allelic expression patterns could be inferred for this gene in our study, *IGF2* was biallelically expressed in 10 cell lines and monoallelically expressed in only 1 cell line (Additional file 12: Table S6). Notably, LOI of *IGF2* is an early initiating event in the development of ileal neuroendocrine tumors [55, 56], and our results show biallelic expression of *IGF2* isoforms in cell lines from other neuroendocrine tumors (neuroblastoma and SCLC; Additional file 17: Table S9 G and J).

Many imprinted genes play roles in embryonic and childhood development, and aberrant imprinting, e.g., LOI at 11p15, occurs in a variety of pediatric embryonic tumors [75]. Our study analyzed several pediatric neuroblastoma and pediatric AML cell lines (Additional file 4: Table S3), including M-07e (M07E), AML-193 (AML193), and CMK (derived from an infant with Down syndrome) [182–184]. However, the dataset analyzed in our study did not include other pediatric tumor categories where alterations in imprinted genes have an early causal impact. Such categories include, e.g., the Wilms tumors, in which LOI of *IGF2* is a common early initiating event [57], or the childhood onset retinoblastoma, in which biallelic loss of *RB1* is an early causal event [76]. In future studies, our approach could be used to evaluate potential causal effects of germline or somatic changes in allelic expression of imprinted genes in pediatric tumor development. This can be achieved by expanding the investigation of allelic expression of imprinted genes in a wide range of pediatric tumors and in matched non-malignant tissues. This may improve the understanding of imprinting dysregulation in pediatric tumors, where LOI or gain of imprinting of specific isoforms, and potential aberrant allelic expression of non-imprinted genes and isoforms may have direct causal effect on tumorigenesis.

Our study generated a novel comprehensive resource of allelic expression patterns of individual exons, isoforms, and whole genes. It also includes data on their overall expression, counts of heterozygous SNVs in RNA and WES data, and gene-level copy number data in 108 cancer cell lines from 9 pediatric and adult tumor types. This dataset provides a resource for future high-resolution studies of molecular changes in cancer. Isoform- and exon-level data provide a better match for underlying transcriptional events than whole gene data currently employed by many studies of imprinted and non-imprinted genes.

While our algorithm was developed to examine transcriptome-wide allelic expression in tumor cells, modifications or extensions of our pipeline may be adapted for additional goals. For example, the use of heterozygous SNVs in expressed transcripts and WES data may assist in identification of neoantigens and to assess neoantigen loss in tumors, to improve cancer immunotherapy outcomes [185]. Another study developed the cis-X framework for using heterozygous SNVs and highly expressed allele-specific transcripts to identify noncoding regulatory elements [146]. Our approach allows for a potential expansion of such an approach to include genes expressed at lower levels. As another potential application, the framework developed by our study could be employed to evaluate biological changes in germline allelic expression patterns in imprinting disorders or other inborn disorders.

## Conclusions

We performed a comprehensive investigation of allelic expression patterns in 108 cancer cell lines from 9 tumor histologies. Our study developed a computational framework for detailed transcriptome-wide allelic expression analysis. We also generated a dataset of allelic expression patterns, which can be utilized by diverse biological investigations of molecular alterations in tumors. We confirmed previously reported expression patterns of multiple imprinted and non-imprinted genes and provided new data on transcriptome-wide allelic expression. Our study identified associations of chemosensitivity of tumor cell lines with allelic expression patterns of imprinted genes and predominantly monoallelically expressed genes, providing novel insights into potential biomarkers for cancer treatment.

## Supplementary Information

**Supplementary Material 1. Figure S1**. An overview of the steps of the analysis of CCLE cancer cell line data. Detailed description of each step is provided in the Methods section. The algorithm for inference of allelic patterns of expression is provided in Fig. 1. EBI, European Bioinformatics institute; UCSC, University of California, Santa Cruz.

**Supplementary Material 2. Table S1**. The initial list 94 imprinted genes compiled from biomedical resources.

**Supplementary Material 3. Table S2**. Cell line data used in analyses of allelic expression status and associations with drug response.

**Supplementary Material 4. Table S3**. List of 108 CCLE cell lines with matching RNA-seq, WES, and copy number data which were included in the analysis of allelic expression patterns, and available drug response information for each cell line.

**Supplementary Material 5. Figure S2**. Proportion of genes and isoforms with no detected heterozygous SNVs relative to log10 length normalized HTSEQ counts in RNA-seq and WES data. (A) genes, RNA-seq expression data; (B) genes, WES data; (C) isoforms, RNA-seq expression data; (D) isoforms, WES data. LOESS regression line is shown in green. Separate plots are provided for each of the 9 cancer categories and for the combined pancancer dataset.

**Supplementary Material 6. Table S4**. Top 60 genes which were not included in the initial list of 94 imprinted genes and were found to have predominantly monoallelic expression in the pancancer analysis of 108 CCLE cell lines.

**Supplementary Material 7. Table S5**. Numbers of agents included in association analyses of in vitro drug response with allelic expression patterns and overall expression levels of isoforms.

**Supplementary Material 8. Figure S3**. A flowchart of inference from VCF files of the expressed allele in monoallelically expressed isoforms and genes in 94 imprinted genes and 60 additional genes with predominantly monoallelic expression. Features, genes or isoforms. An alternative base was reported in the VCF files relative to the human hg19 genome reference sequence.

**Supplementary Material 9. Figure S4**. Number of RNA-seq and WES sequencing reads mapped to exons using HTSeq in each of the 9 tumor histologies of the 108 cell lines.

**Supplementary Material 10. Figure S5**. Comparison of the allelic expression patterns of the 94 imprinted vs 59,189 remaining genes at the (A) gene, (B) isoform, and (C) exon levels in each of the 9 cancer histologies of the 108 cell lines. Boxplots of the 94 imprinted genes listed in Additional file 2:Table S1 are represented by the lighter shades (left). Boxplots of the other genes representing the remaining 59,189 genes not included in the original list of 94 imprinted genes are shown by the darker shades of the same color for each tumor category (right).

**Supplementary Material 11. Figure S6**. Comparison of the gene level allelic expression patterns of the 94 imprinted genes vs average expression patterns in 94 genes that were resampled, using 1000 replications, from 59,189 remaining genes. Boxplots of the 94 imprinted genes listed in Additional file 2:Table S1 are represented by the lighter shades (left). Boxplots of the 94 subsampled genes are shown by the darker shades of the same color for each tumor category (right).

**Supplementary Material 12. Table S6**. Gene level allelic expression patterns of the previously reported 94 imprinted genes among 108 CCLE cell lines.

**Supplementary Material 13. Figure S7**. Heatmap of tumor histology-specific monoallelic expression among the 94 imprinted genes. For each gene and tumor category, shown is the proportion of monoallelically expressed genes among monoallelically or biallelically expressed genes, at the whole gene level. Dendrograms were inferred using Euclidian distances and complete linkage clustering. Biallelic only expression (values of 0 monoallelic and 1 biallelic counts) is presented by light blue color. N/A, cases with 0 monoallelic and 0 biallelic counts, are shown as grey color. Coloring of > 0 to 1 is on a 100 color gradient.

**Supplementary Material 14. Table S7**. Average proportions (%) of transcriptome-wide allelic expression patterns of genes, isoforms, and individual exons with heterozygous SNVs in 108 CCLE cell lines .

**Supplementary Material 15. Table S8**. Gene level allelic expression patterns of non-imprinted control genes.

**Supplementary Material 16. Figure S8**. Comparison of the allelic expression patterns of the 94 imprinted genes vs 60 additional predominantly monoallelically expressed genes at the (A) gene, (B) isoform, and (C) exon levels in each of the 9 cancer histologies of the 108 cell lines. Boxplots of the 94 imprinted genes listed in Additional file 2:Table S1 are represented by the lighter shades (left, marked as Imprinted in the legend). Boxplots of the 60 additional predominantly monoallelically expressed genes listed in Additional file 6:Table S4 are shown by the darker shades of the same color for each tumor category (right, marked as Mono in the legend).

**Supplementary Material 17. Table S9**. Allelic expression patterns of isoforms of the 94 imprinted genes.

**Supplementary Material 18. Figure S9**. Tissue-specific allelic expression patterns of 28 *PLAGL1* isoforms. Shown are proportions (%) of each allelic expression category within the cell lines from each of the 9 tumor categories.

**Supplementary Material 19. Table S10**. Numbers and proportions of monoallelically and biallelically expressed isoforms of *PLAGL1* in different cancer categories.

**Supplementary Material 20. Table S11**. Concordance among isoform level allelic expression of CCLE cell lines in our study with previously reported whole gene level results of Martin-Trujillo et al. (2017) in matching COSMIC cell lines.

**Supplementary Material 21. Figure S10**. Violin plots for associated agents and expression patterns of the isoform groups satisfying *p*_FDR_ < 0.05 for the 94 imprinted genes.

**Supplementary Material 22. Figure S11**. Violin plots for associated agents and expression patterns of the isoform groups satisfying *p*_FDR_ < 0.05 for 60 predominantly monoallelic genes.

**Supplementary Material 23. Table S12**. Associations of allelic expression patterns of isoform groups of imprinted genes and other genes with predominantly monoallelic expression with in vitro drug response satisfying *p*_FDR_ < 0.125.

**Supplementary Material 24. Table S13**. Spearman correlation of log(IC50) and mean allelic expression levels of isoform groups of imprinted genes and other genes with predominantly monoallelic expression with in vitro drug response satisfying *p*_FDR_ < 0.125.

**Supplementary Material 25. Table S14**. Associations of allelic expression patterns of isoform groups of imprinted genes and other genes with predominantly monoallelic expression with isoform expression satisfying *p*_FDR_ < 0.125.

**Supplementary Material 26. Table S15**. Counts of the expressed allele in monoallelically expressed genes and isoforms and of both alleles in the genomic data for heterozygous SNVs located within 94 imprinted genes and 60 additional genes with predominantly monoallelic expression.

**Supplementary Material 27. Table S16**. Results of the two-sided binomial test for preferential monoallelic expression of sequence variants.

**Supplementary Material 28. Table S17**. Distribution of statistically significant (*p* < 0.05) preference for monoallelic expression of a single base among SNVs in imprinted genes, as compared to SNVs in other predominantly monoallelically expressed genes.

**Supplementary Material 29**. Supplementary figure and table legends.

## Data Availability

All CCLE, DepMap, and GDSC molecular data and drug response data used in this project are publicly available online. Information about access to these resources is provided in the Methods section. The source code developed for this study and the study results, including counts of heterozygous SNVs in RNA-seq and WES data, copy number information, numbers of mapped reads, inferred allelic expression of each feature in each cell line, and summary counts of allelic expression patterns of each feature within each tumor category and in the combined pancancer dataset, are available on figshare (10.6084/m9.figshare.27037978).

## Abbreviations

aLOH: Amplified loss of heterozygocity
AML: Acute myeloid leukemia
ANO1: Anoctamin 1
BLADDER: Bladder cancer
BREAST: Breast cancer
BRP: Biometric Research Program
BWA: Burrows-Wheeler Aligner
CCLE: Cancer Cell Line Encyclopedia
cnLOH: Copy number-neutral loss of heterozygocity
CTCF: CCCTC-binding factor (CTCF)
COSMIC: Catalogue Of Somatic Mutations In Cancer
DDA: DNA damaging agent
DepMap: Cancer Dependency Map
DMR: Differentially methylated region
EBI: European Bioinformatics Institute
GDC: Genomic Data Commons
GDSC: Genomics of Drug Sensitivity in Cancer
ICR: Imprinting control region
IGF2: Insulin-like growth factor 2
I-NET: Ileal neuroendocrine tumor
lncRNA: Long noncoding RNA
LOH: Loss of heterozygocity
LOI: Loss of imprinting
NCI: National Cancer Institute
NCBI: National Center for Biotechnology Information
OVARIAN: Ovarian cancer
*p*_0_: *p*-Value prior to FDR adjustment
*p*_FDR_: *p*-Values after FDR adjustment
PDAC: Pancreatic ductal adenocarcinoma
PRISM: Profiling Relative Inhibition Simultaneously in Mixtures
RB1: Retinoblastoma
RMAE: Random monallelic expression
SCLC: Small cell lung cancer
SNV: Single nucleotide variant
SRA: Sequence Read Archive
UCSC: University of California, Santa Cruz
UPD: Uniparental disomy
WES: Whole-exome sequencing
