# Supplementary figures and images for: Allelic expression patterns of imprinted and non-imprinted genes in cancer cell lines from multiple histologies
Source: Clin Epigenetics. 2025 May 25;17:83. doi: 10.1186/s13148-025-01883-3 (PMC12105275; doi:10.1186/s13148-025-01883-3)

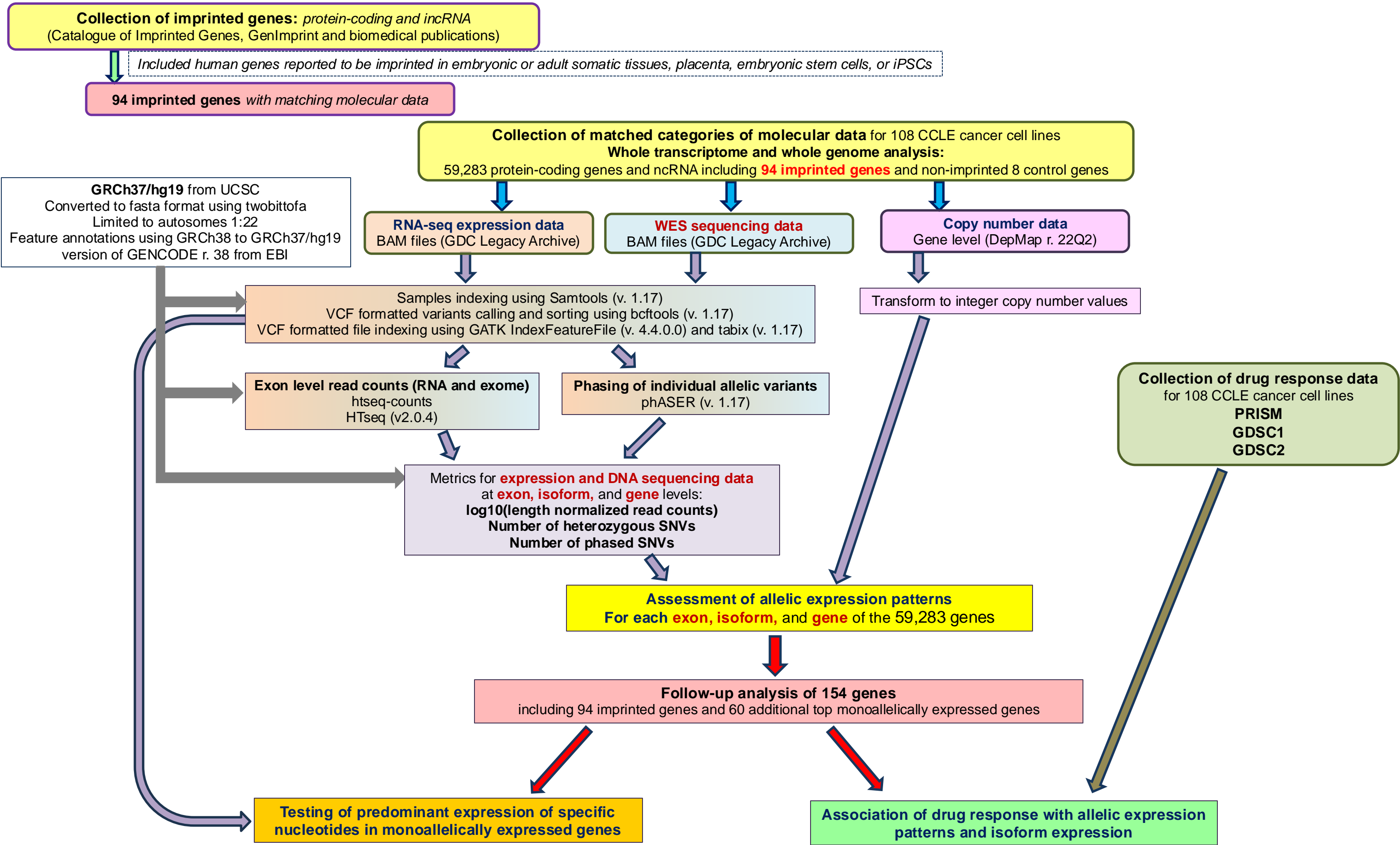

Fig. S1

Supplement: Supplementary file 1 — Supplementary Material 1. Figure S1. An overview of the steps of the analysis of CCLE cancer cell line data. Detailed description of each step is provided in the Methods section. The algorithm for inference of allelic patterns of expression is provided in Fig. 1. EBI, European Bioinformatics institute; UCSC, University of California, Santa Cruz. [file 13148_2025_1883_MOESM1_ESM.pdf]

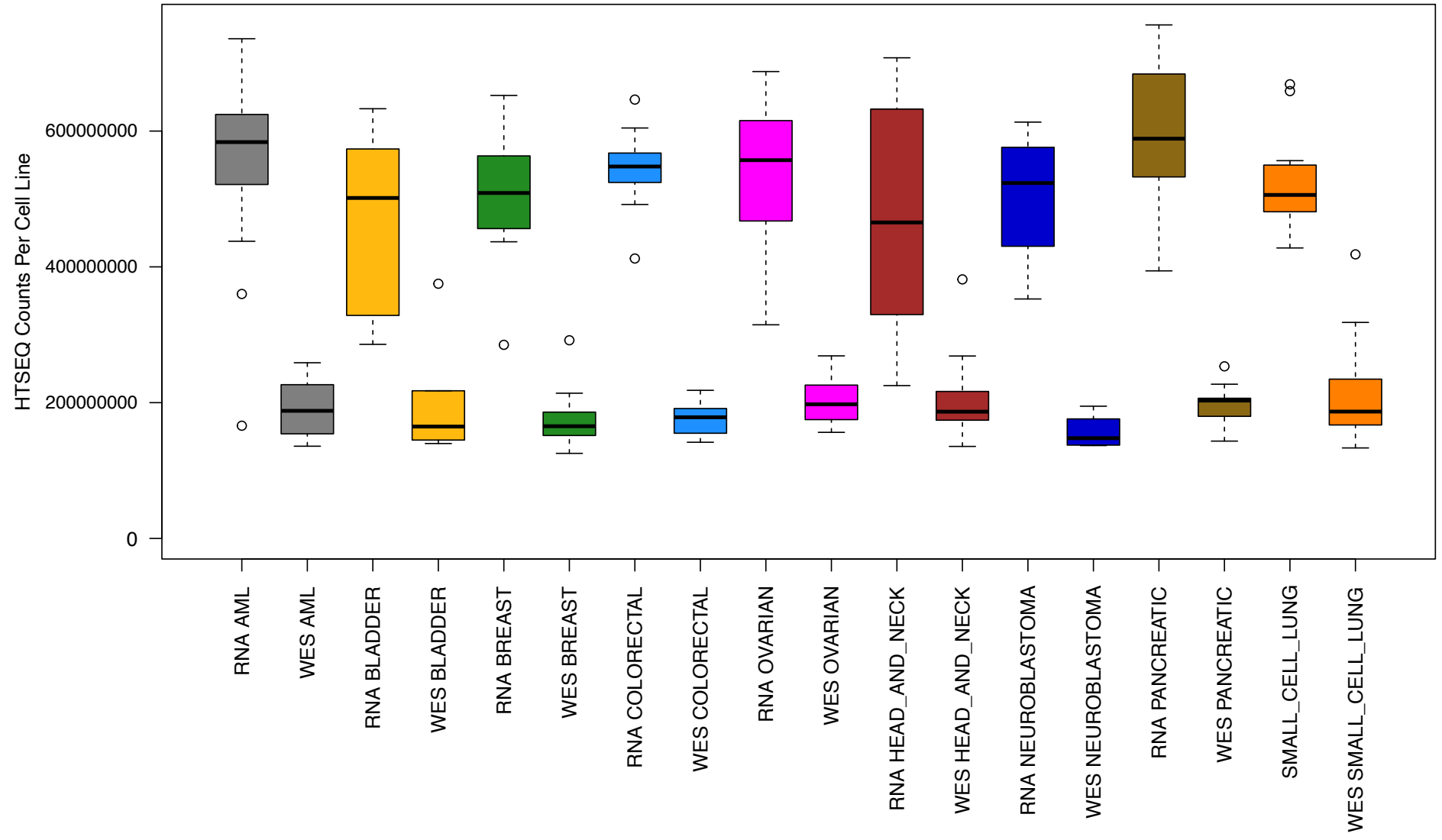

**Fig. S4**

Supplement: Supplementary file 9 — Supplementary Material 9. Figure S4. Number of RNA-seq and WES sequencing reads mapped to exons using HTSeq in each of the 9 tumor histologies of the 108 cell lines. [file 13148_2025_1883_MOESM9_ESM.pdf]

PLAGL1 Status by Cancer Category (n features = 28 isoforms)

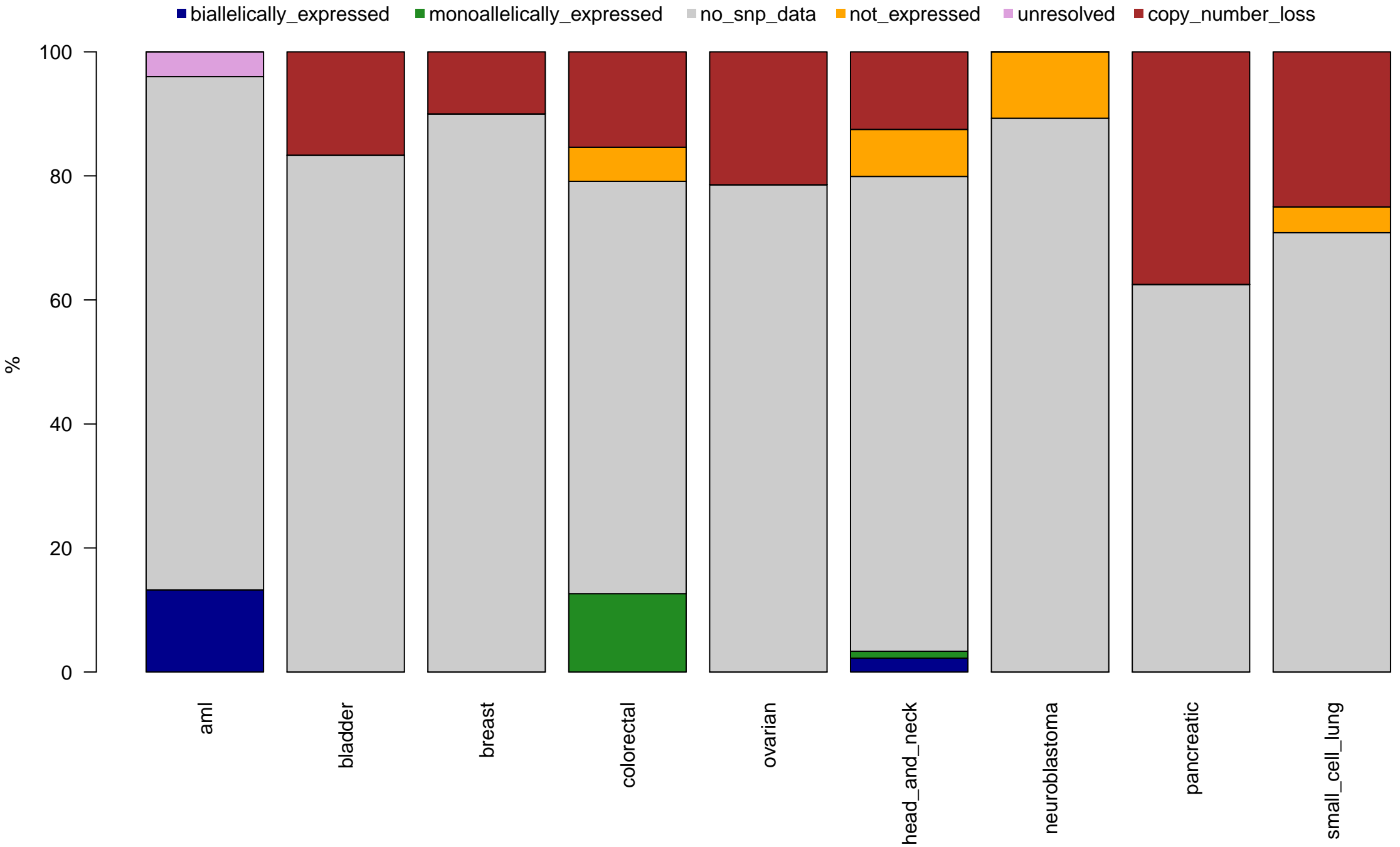

**Fig. S9**

Supplement: Supplementary file 18 — Supplementary Material 18. Figure S9. Tissue-specific allelic expression patterns of 28 PLAGL1 isoforms. Shown are proportions (%) of each allelic expression category within the cell lines from each of the 9 tumor categories. [file 13148_2025_1883_MOESM18_ESM.pdf]
