# Supplementary material for: Allelic expression patterns of imprinted and non-imprinted genes in cancer cell lines from multiple histologies
Source: Clin Epigenetics. 2025 May 25;17:83. doi: 10.1186/s13148-025-01883-3 (PMC12105275; doi:10.1186/s13148-025-01883-3)
Supplement: Supplementary file 2 — Supplementary Material 2. Table S1. The initial list 94 imprinted genes compiled from biomedical resources. [file 13148_2025_1883_MOESM2_ESM.pdf]

Table S1. The initial list 94 imprinted genes compiled from biomedical resources

Gene name	Synonyms	Gene category	Location[a]	References for imprinted status[b]	Gene ID	Gene start	Gene end	Overlapping exon bases	Unique exon bases
AIRN		ncRNA	6q25	[1-4]	ENSG00000268257.2_1	160424322	160428696	5867	4375
ANO1		protein-coding	11q13.3	[1-3, 5-8]	ENSG00000131620.17_1	69924407	70035634	10526	6051
ATP10A		protein-coding	15q12	[1-3, 5-7]	ENSG00000206190.12_1	25922417	26110331	43246	20707
BLCAP		protein-coding	20q11.23	[1-3, 5-7]	ENSG00000166619.15_1	36120873	36156333	14182	7880
CALCR		protein-coding	7q21.3	[1-3, 5, 7]	ENSG00000004948.16_1	93053797	93204042	7492	4239
CDKN1C		protein-coding	11p15.4	[1-7, 9]	ENSG00000129757.15_1	2904442	2907005	7633	2482
CPA4		protein-coding	7q32.2	[1-3, 5-7, 10]	ENSG00000128510.12_1	129932973	129964020	6435	3971
DCN		protein-coding	12q21.33	[1-3, 11]	ENSG00000011465.18_1	91534260	91576994	19350	12956
DDC		protein-coding	7p12.2-p12.1	[1-3, 5-7]	ENSG00000132437.18_1	50526133	50633102	6676	3104
DGCR6		protein-coding	22q11.21	[1-3, 6, 7]	ENSG00000183628.14_1	18893540	18901751	10002	5173
DIO3		protein-coding	14q32.31	[1-3, 5, 11]	ENSG00000197406.7_1	102027687	102029789	2103	2103
DIRAS3		protein-coding	1p31.3	[1-3, 5-7, 9, 10]	ENSG00000162595.7_1	68511568	68517314	3536	2053
DLGAP2		protein-coding	8p23.3	[1-3, 5-7, 12]	ENSG00000198010.13_1	687627	1656642	27969	15158
DLK1		protein-coding	14q32.2	[1-3, 6, 7, 9-11]	ENSG00000185559.16_1	101192041	101204561	6963	5254
DLX5		protein-coding	7q22	[1-3, 6, 7]	ENSG00000105880.7_1	96649707	96654262	3562	1747
DNMT1		protein-coding	19p13.2	[1-3, 6, 13]	ENSG00000130816.17_1	10244017	10341962	45270	15422
FAM50B		protein-coding	6p25.2	[1-3, 6, 7, 10]	ENSG00000145945.7_1	3849606	3851554	3581	1949
GABRA5		protein-coding	15q12	[1-3]	ENSG00000186297.12_1	27112057	27194355	7694	3621
GABRB3		protein-coding	15q12	[1-3, 9, 14]	ENSG00000166206.15_1	26788692	27184686	46365	13920
GABRG3		protein-coding	15q12	[1-3, 15]	ENSG00000182256.13_1	27216327	27787130	14347	12703
GDAP1L1		protein-coding	20q12	[1-3, 6, 16]	ENSG00000124194.17_1	42875738	42909587	7447	3411
GLIS3		protein-coding	9p24.2	[1-3, 6, 7]	ENSG00000107249.24_1	3824126	4348392	43057	15493
GNAS		protein-coding	20q13.32	[1-3, 6, 7, 17]	ENSG00000087460.29_1	57414772	57486247	78380	22238
GNAS-AS1	***GNASAS, GNAS-AS***	ncRNA	20q13.32	[1-3, 6, 7, 11, 17]	ENSG00000235590.7_1	57393973	57425958	4367	3666
GPR1		protein-coding	2q33.3	[1-3, 6, 7]	ENSG00000183671.12_1	207040039	207082771	9092	2840
GRB10		protein-coding	7p12.1	[1-3, 6, 7, 9, 10]	ENSG00000106070.20_1	50657759	50861159	28892	9827
H19		ncRNA	11p15.5	[1-3, 6, 7, 9, 10]	ENSG00000130600.19_1	2016405	2022700	10100	2901
HM13		protein-coding	20q11.21	[1-3, 7]	ENSG00000101294.18_1	30102212	30165726	20891	8784
HTR2A		protein-coding	13q14.2	[1-3]	ENSG00000102468.11_1	47405680	47471211	10621	5632
IGF2		protein-coding	11p15.5	[1-3, 5-7, 9, 10]	ENSG00000167244.21_1	2150341	2162468	14168	6266
IGF2-AS	***IGF2AS***	ncRNA	11p15.5	[1-3, 5, 6, 10]	ENSG00000099869.8_1	2161730	2169896	5579	2871
IGF2R		protein-coding	6q25.3	[1-3, 7]	ENSG00000197081.16_1	160390113	160534539	46919	22665
INPP5F		protein-coding	10q26.11	[1-3, 5-7, 10]	ENSG00000198825.15_1	121485553	121588659	39544	7505
INS		protein-coding	11p15.5	[1-3, 5, 6]	ENSG00000254647.7_1	2181008	2182451	1735	658
KCNK9		protein-coding	8q24.3	[1-3, 5-7, 12]	ENSG00000169427.8_1	140613080	140716352	19073	6793

KCNQ1		protein-coding	11p15.4-p15.5	[1-7, 10]	ENSG00000053918.18_1	2465913	2870335	6748	4442
KCNQ1DN		ncRNA	11p15.4-p15.5	[1-3, 5, 6]	ENSG00000237941.2_1	2891262	2893335	1095	1095
KCNQ1OT1		ncRNA	11p15.5	[1-7, 9, 11]	ENSG00000269821.1_1	2629557	2721224	91668	91668
KLF14		protein-coding	7q32.2	[1-3, 5-7]	ENSG00000266265.4_1	130415524	130418967	148	148
L3MBTL1	***L3MBTL***	protein-coding	20q13.12	[1-3, 5-7, 9, 10, 16]	ENSG00000185513.17_1	42118081	42179594	55663	15569
LIN28B		protein-coding	6q16.3-q21	[1-3, 5-7]	ENSG00000187772.8_1	105384490	105531207	8258	5951
LRRTM1		protein-coding	2p12	[1-3, 5-7]	ENSG00000162951.11_1	80515475	80531877	12077	5052
MAGEL2		protein-coding	15q11.2	[1-3, 5-7, 9, 10, 15]	ENSG00000254585.5_1	23888695	23893014	4320	4320
MAGI2		protein-coding	7q21.11	[1-3, 5-7, 10]	ENSG00000187391.22_1	77646371	79082983	64080	34086
MEG3		ncRNA	14q32.2	[1-3, 5-7, 9-11]	ENSG00000214548.18_1	101245746	101327368	54636	23231
MEG8		ncRNA	14q32.2-q32.31	[1-3, 5, 6, 10]	ENSG00000225746.13_1	101361106	101505196	42971	27570
MEST		protein-coding	7q32.2	[1-3, 5-7, 9, 10]	ENSG00000106484.16_1	130126011	130146306	12768	4785
MIMT1		ncRNA	19q13.43	[1-3, 5-7]	ENSG00000268654.2_1	57352233	57359924	3522	2040
MKRN3		protein-coding	15q11.2	[1-3, 5-7, 9]	ENSG00000179455.10_1	23810820	23875222	18994	7127
NAA60		protein-coding	16p13.3	[1-3, 5-7]	ENSG00000122390.19_1	3493648	3536953	31634	7363
NAP1L5		protein-coding	4q22.1	[1-3, 5-7, 9, 10]	ENSG00000177432.8_1	89617063	89618980	1918	1918
NDN		protein-coding	15q11.2	[1-3, 5-7, 9-11, 15]	ENSG00000182636.8_1	23930546	23932452	1907	1907
NLRP2		protein-coding	19q13.42	[1-3, 5-7, 9]	ENSG00000022556.16_1	55464497	55512510	14534	6333
NNAT		protein-coding	20q11.23	[1-3, 5-7, 9]	ENSG00000053438.11_1	36149607	36152092	3888	1332
NPAP1		protein-coding	15q11.2	[1-3, 5, 6]	ENSG00000185823.5_1	24920921	24928540	7620	7620
NTM		protein-coding	11q25	[1-3, 5-7, 10]	ENSG00000182667.15_1	131240372	132206716	15982	6978
OSBPL5		protein-coding	11p15.4	[1-3, 5-7]	ENSG00000021762.20_1	3108336	3187969	17633	7499
PEG3	***ZIM2***	protein-coding	19q13.43	[1-3, 5, 6, 9, 10]	ENSG00000198300.14_1	57321444	57352096	81983	9783
PEG10		protein-coding	7q21.3	[1-3, 5-7, 9, 10]	ENSG00000242265.6_1	94285636	94299007	26462	6692
PHACTR2		protein-coding	6q24.2	[1-3, 5-7]	ENSG00000112419.14_1	143857981	144152322	17846	11240
PHLDA2		protein-coding	11p15.4	[1-7]	ENSG00000181649.8_1	2949502	2950650	922	922
PLAGL1	***ZAC1***	protein-coding	6q24.2	[1-3, 5-10]	ENSG00000118495.20_1	144261436	144385736	38297	8742
PPP1R9A		protein-coding	7q21.3	[1-3, 5-7]	ENSG00000158528.12_1	94536513	94925727	33455	11218
PWRN1		ncRNA	15q11.2	[1-3, 5, 18]	ENSG00000259905.7_1	24346973	25068512	39458	24331
RASGRF1		protein-coding	15q25.1	[1-3, 19]	ENSG00000058335.16_1	79252247	79383122	18171	10008
RB1		protein-coding	13q14.2	[1-3, 5-7, 9, 20]	ENSG00000139687.16_1	48877879	49173572	12000	6464
RBP5		protein-coding	12p13.31	[1-3, 5-7]	ENSG00000139194.8_1	7268331	7281485	4763	3032
RTL1		protein-coding	14q32.2-q32.31	[1-3, 5-7, 11]	ENSG00000254656.3_1	101346089	101370059	5402	5402
SGCE		protein-coding	7q21.3	[1-3, 5-7]	ENSG00000127990.19_1	94153515	94285884	64099	20892
SGK2		protein-coding	20q13.12	[1-3, 5-7, 16]	ENSG00000101049.17_1	42187607	42216877	10867	5156
SLC22A18		protein-coding	11p15.4	[1-7]	ENSG00000110628.16_1	2920950	2946476	8899	5247
SLC22A18AS	***SLC22A18-AS, SLC22A18-AS1***	ncRNA	11p15.4	[1-4]	ENSG00000254827.6_1	2908573	2924805	3173	2058
SLC22A2		protein-coding	6q25.3	[1-3, 5, 6, 21, 22]	ENSG00000112499.13_1	160592092	160698670	9022	6000

SLC22A3		protein-coding	6q25.3	[1-7, 9, 22]	ENSG00000146477.6_1	160769409	160873609	3245	3245
SNRPN		protein-coding	15q11.2	[1-3, 5-7, 9, 23]	ENSG00000128739.23_1	25068783	25223870	5785	3430
SNURF		protein-coding	15q11.2	[1-3, 5-7, 23]	ENSG00000273173.5_1	25200132	25222997	1445	1183
TFPI2		protein-coding	7q21.3	[1-3, 5-7, 11]	ENSG00000105825.14_1	93514707	93520065	4615	2449
TH		protein-coding	11p15.5	[1-3]	ENSG00000180176.15_1	2185158	2193045	5835	3193
TP73		protein-coding	1p36.32	[1-3, 5-7]	ENSG00000078900.15_1	3569079	3652765	18933	6783
UBE3A		protein-coding	15q11.2	[1-3, 5-7]	ENSG00000114062.22_1	25578874	25684198	42760	19053
USP29		protein-coding	19q13.43	[1-3, 9]	ENSG00000131864.10_1	57630505	57643294	7568	3899
VTRNA2-1		nc RNA	5q31.1	[1-3, 6, 24]	ENSG00000270123.4_1	135416158	135416286	129	129
WIF1		protein-coding	12q14.3	[1-3, 7]	ENSG00000156076.10_1	65444405	65515085	2547	2117
WT1		protein-coding	11p13	[1-3, 5-7]	ENSG00000184937.16_1	32409320	32457110	28026	8146
WT1-AS	***WT1AS***	ncRNA	11p13	[1-3, 6, 25]	ENSG00000183242.12_1	32457063	32480315	7789	5570
ZC3H12C		protein-coding	11q22.3	[1-3, 5-7]	ENSG00000149289.11_1	109964117	110042566	19543	9695
ZDBF2		protein-coding	2q33.3	[1-3, 5, 6, 9]	ENSG00000204186.10_1	207139386	207179151	55561	10910
ZFAT		protein-coding	8q24.22	[1-3, 5-7]	ENSG00000066827.16_1	135490030	135725292	19580	8923
ZFAT-AS1	***ZFAT-AS***	ncRNA	8q24.22	[1-3, 5-7]	ENSG00000248492.1_1	135610313	135612932	941	941
ZIM2		protein-coding	19q13.43	[1-3, 5-7]	ENSG00000269699.6_1	57285914	57352097	8460	3080
ZIM3		protein-coding	19q13.43	[1-3, 9]	ENSG00000141946.1_1	57645463	57656570	2632	2632
ZNF215		protein-coding	11p15.4	[1-3]	ENSG00000149054.16_1	6947634	7022235	12735	5978
ZNF331		protein-coding	19q13.42	[1-3, 5, 7]	ENSG00000130844.19_1	54022780	54083523	25940	8708
ZNF597		protein-coding	16p13.3	[1-3, 5-7]	ENSG00000167981.7_1	3482413	3493504	5487	5487

Listed are the genes with available molecular data which had been reported to be imprinted in embryonic or adult somatic tissues, placenta, embryonic stem cells, or induced pluripotent stem cells (iPSCs).

[a] Chromosomal location is provided according to the data in the Catalog of Imprinted Genes [1-3], Bonaldi et al. [5], and additional publications listed among the references. For those imprinted genes that had minor discrepancies among their reported chromosomal location from different sources, their chromosomal location was reported according to GeneCards [26].

[b] The Geneimprint resource [6] was described in [27].

Gene category is provided according to the information reported by Bonaldi et al. [5] and/or GeneCards [26].

Overlapping exon bases provides the combined length of all isoforms in a given gene, some of which may be overlapping

Unique exon bases provides the total length of unique isoform bases in a given gene, with duplicate positions excluded

Gene and exon annotation is provided according to GENCODE, using lifted annotations from V38lift37 (Ensembl 104) mapped to hg19 (gencode.v38lift37).

The "**_1**" , which is a part of each **Gene ID**, is included in the GENCODE GTF/GFF feature annotations for all genes/transcripts/exons, corresponding to the mapping version "lift" from GRCh38 to GRCh37/hg19 [28].

References for Supplementary Table 1

1. Morison I. Catalogue of Imprinted Genes. 2021. Available from: http://www.otago.ac.nz/IGC. Accessed: 22 July 2021
2. Morison IM, Paton CJ, Cleverley SD. The imprinted gene and parent-of-origin effect database. Nucleic Acids Res. 2001;29:275-6.
3. Morison IM, Ramsay JP, Spencer HG. A census of mammalian imprinting. Trends Genet. 2005;21:457-65.
4. Monk D, Arnaud P, Apostolidou S, Hills FA, Kelsey G, Stanier P et al. Limited evolutionary conservation of imprinting in the human placenta. Proc Natl Acad Sci U S A. 2006;103:6623-8.
5. Bonaldi A, Kashiwabara A, de Araujo ES, Pereira LV, Paschoal AR, Andozia MB et al. Mining novel candidate imprinted genes using genome-wide methylation screening and literature review. Epigenomes. 2017;1.
6. Geneimprint. Available from: http://www.geneimprint.org/. Accessed: 8 April 2019
7. Babak T, DeVeale B, Tsang EK, Zhou Y, Li X, Smith KS et al. Genetic conflict reflected in tissue-specific maps of genomic imprinting in human and mouse. Nat Genet. 2015;47:544-9.
8. Smeester L, Yosim AE, Nye MD, Hoyo C, Murphy SK, Fry RC. Imprinted genes and the environment: links to the toxic metals arsenic, cadmium, lead and mercury. Genes (Basel). 2014;5:477-96.
9. Anwar SL, Krech T, Hasemeier B, Schipper E, Schweitzer N, Vogel A et al. Loss of DNA methylation at imprinted loci is a frequent event in hepatocellular carcinoma and identifies patients with shortened survival. Clin Epigenetics. 2015;7:110.
10. Baran Y, Subramaniam M, Biton A, Tukiainen T, Tsang EK, Rivas MA et al. The landscape of genomic imprinting across diverse adult human tissues. Genome Res. 2015;25:927-36.
11. Ribarska T, Bastian KM, Koch A, Schulz WA. Specific changes in the expression of imprinted genes in prostate cancer--implications for cancer progression and epigenetic regulation. Asian J Androl. 2012;14:436-50.
12. Luedi PP, Dietrich FS, Weidman JR, Bosko JM, Jirtle RL, Hartemink AJ. Computational and experimental identification of novel human imprinted genes. Genome Res. 2007;17:1723-30.
13. Das R, Lee YK, Strogantsev R, Jin S, Lim YC, Ng PY et al. *DNMT1* and *AIM1* Imprinting in human placenta revealed through a genome-wide screen for allele-specific DNA methylation. BMC Genomics. 2013;14:685.
14. Bird LM. Angelman syndrome: review of clinical and molecular aspects. Appl Clin Genet. 2014;7:93-104.
15. Sharp AJ, Migliavacca E, Dupre Y, Stathaki E, Sailani MR, Baumer A et al. Methylation profiling in individuals with uniparental disomy identifies novel differentially methylated regions on chromosome 15. Genome Res. 2010;20:1271-8.
16. Aziz A, Baxter EJ, Edwards C, Cheong CY, Ito M, Bench A et al. Cooperativity of imprinted genes inactivated by acquired chromosome 20q deletions. J Clin Invest. 2013;123:2169-82.
17. Turan S, Bastepe M. The *GNAS* complex locus and human diseases associated with loss-of-function mutations or epimutations within this imprinted gene. Horm Res Paediatr. 2013;80:229-41.
18. Wawrzik M, Spiess AN, Herrmann R, Buiting K, Horsthemke B. Expression of *SNURF-SNRPN* upstream transcripts and epigenetic regulatory genes during human spermatogenesis. Eur J Hum Genet. 2009;17:1463-70.
19. Yuen RK, Jiang R, Penaherrera MS, McFadden DE, Robinson WP. Genome-wide mapping of imprinted differentially methylated regions by DNA methylation profiling of human placentas from triploidies. Epigenetics Chromatin. 2011;4:10.
20. Kanber D, Berulava T, Ammerpohl O, Mitter D, Richter J, Siebert R et al. The human retinoblastoma gene is imprinted. PLoS Genet. 2009;5:e1000790.
21. Frost JM, Moore GE. The importance of imprinting in the human placenta. PLoS Genet. 2010;6:e1001015.
22. Zwart R, Sleutels F, Wutz A, Schinkel AH, Barlow DP. Bidirectional action of the Igf2r imprint control element on upstream and downstream imprinted genes. Genes Dev. 2001;15:2361-6.
23. Hassan M, Butler MG. Prader-Willi syndrome and atypical submicroscopic 15q11-q13 deletions with or without imprinting defects. Eur J Med Genet. 2016;59:584-9.
24. Silver MJ, Kessler NJ, Hennig BJ, Dominguez-Salas P, Laritsky E, Baker MS et al. Independent genomewide screens identify the tumor suppressor *VTRNA2-1* as a human epiallele responsive to periconceptional environment. Genome Biol. 2015;16:118.

25. Haruta M, Arai Y, Sugawara W, Watanabe N, Honda S, Ohshima J et al. Duplication of paternal *IGF2* or loss of maternal *IGF2 i*mprinting occurs in half of Wilms tumors with various structural *WT1* abnormalities. Genes Chromosomes Cancer. 2008;47:712-27.
26. GeneCards®: Human Gene Database. Available from: https://www.genecards.org/. Accessed: 18 November 2021
27. Skaar DA, Li Y, Bernal AJ, Hoyo C, Murphy SK, Jirtle RL. The human imprintome: regulatory mechanisms, methods of ascertainment, and roles in disease susceptibility. ILAR J. 2012;53:341-58.
28. Format description of GENCODE GTF. 2023. Available from: https://www.gencodegenes.org/pages/data_format.html. Accessed: 24 October 2023
