# Supplementary material for: Allelic expression patterns of imprinted and non-imprinted genes in cancer cell lines from multiple histologies
Source: Clin Epigenetics. 2025 May 25;17:83. doi: 10.1186/s13148-025-01883-3 (PMC12105275; doi:10.1186/s13148-025-01883-3)
Supplement: Supplementary file 5 — Supplementary Material 5. Figure S2. Proportion of genes and isoforms with no detected heterozygous SNVs relative to log10 length normalized HTSEQ counts in RNA-seq and WES data. (A) genes, RNA-seq expression data; (B) genes, WES data; (C) isoforms, RNA-seq expression data; (D) isoforms, WES data. LOESS regression line is shown in green. Separate plots are provided for each of the 9 cancer categories and for the combined pancancer dataset. [file 13148_2025_1883_MOESM5_ESM.pdf]

Proportion of genes with no detected heterozygous SNVs vs $\log_{10}$ length normalized HTSEQ counts in RNA-seq data

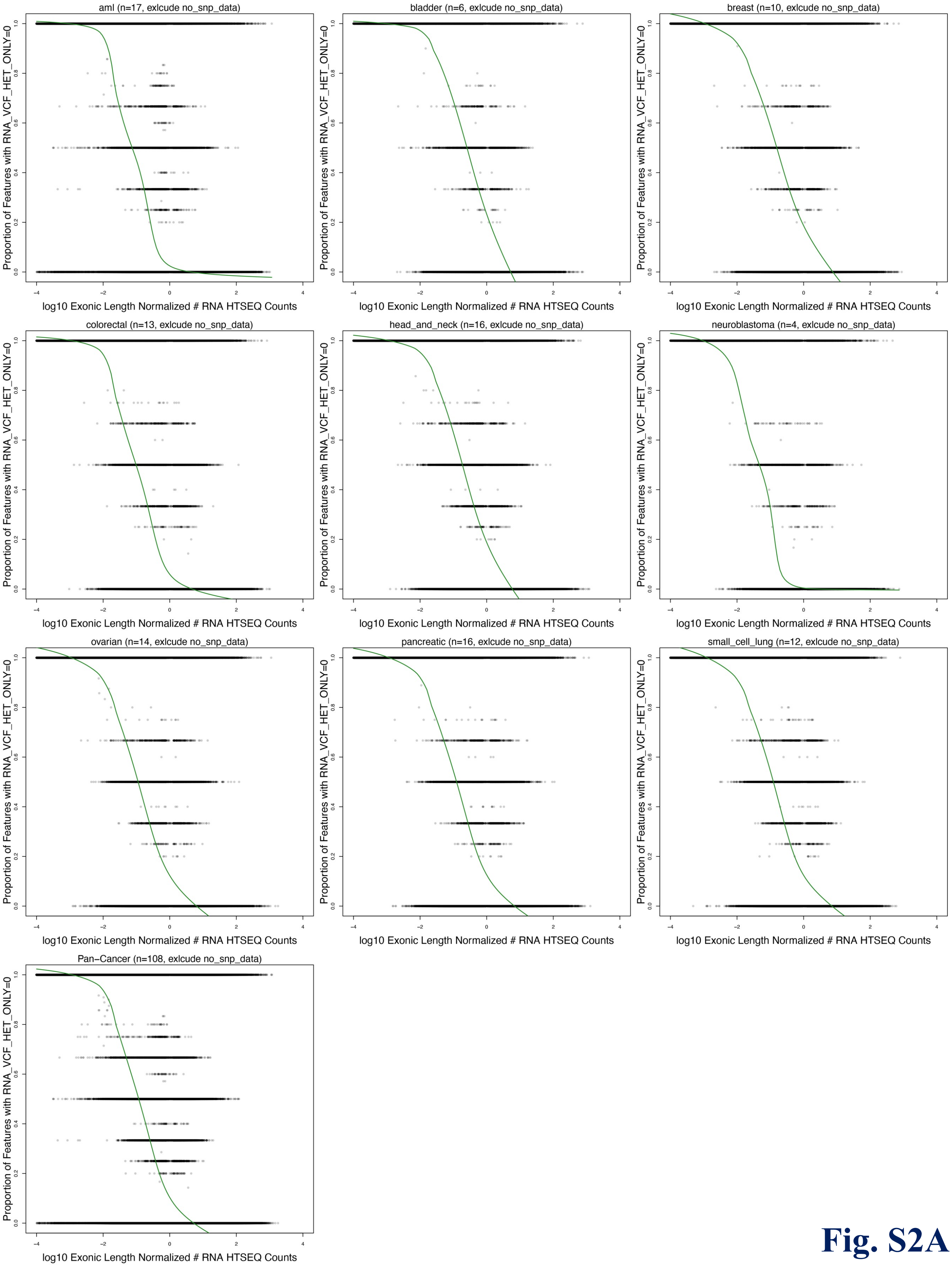

Fig. S2A

Proportion of genes with no detected heterozygous SNVs vs $\log_{10}$ length normalized HTSEQ counts in WES data

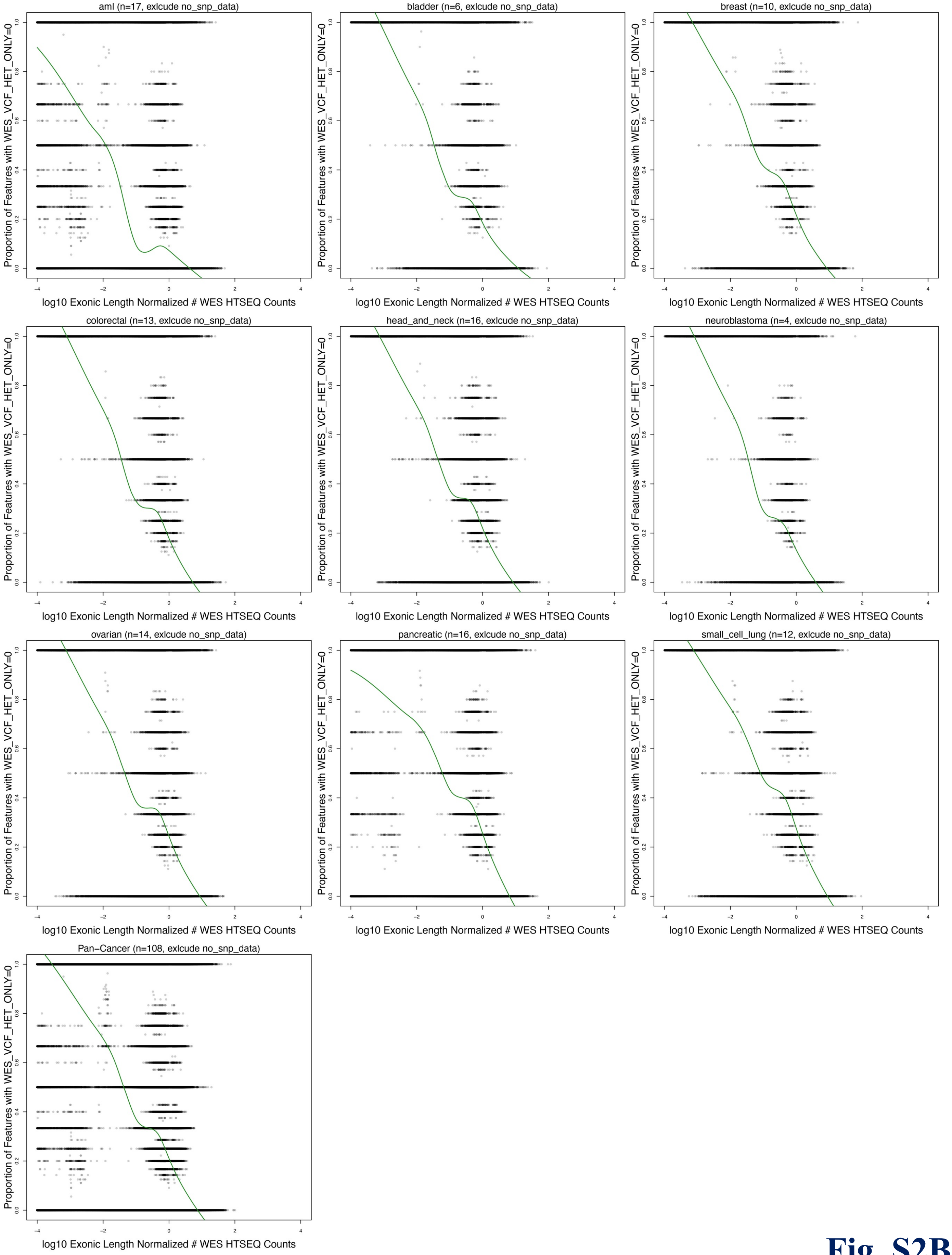

Fig. S2B

Proportion of isoforms with no detected heterozygous SNVs vs $\log_{10}$ length normalized HTSEQ counts in RNA-seq data

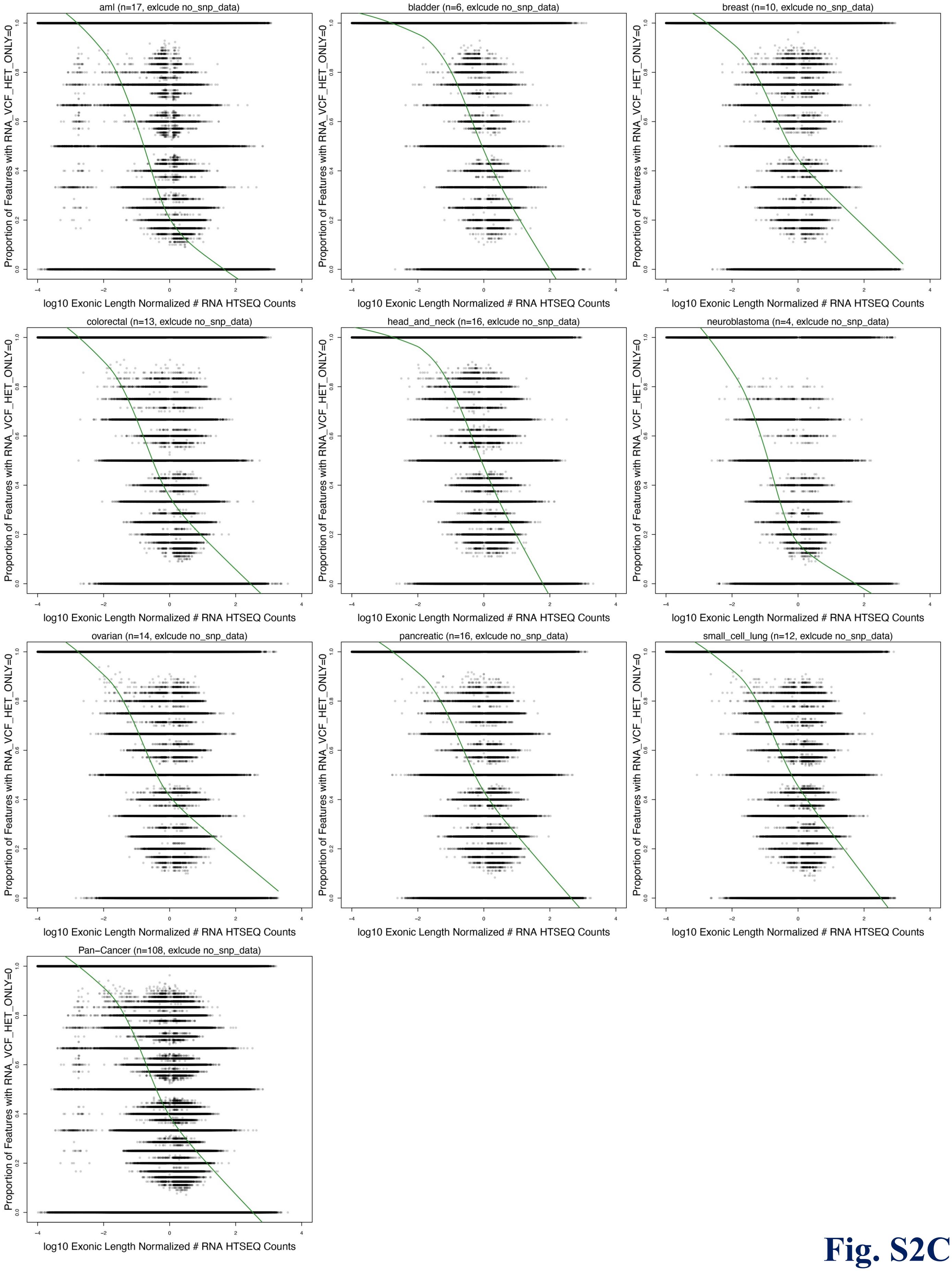

Fig. S2C

Proportion of isoforms with no detected heterozygous SNVs vs $\log_{10}$ length normalized HTSEQ counts in WES data

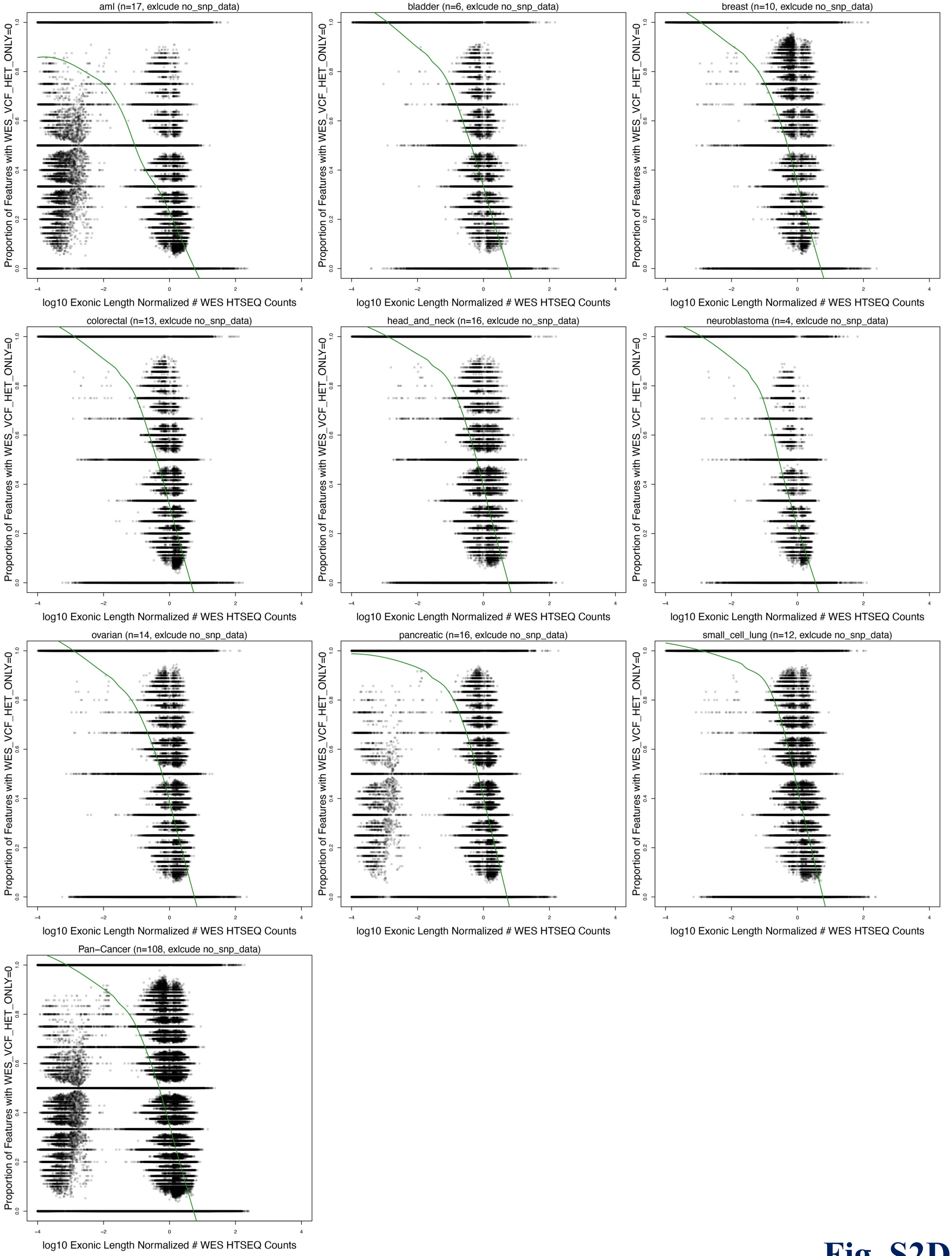

Fig. S2D
