# Supplementary material for: Allelic expression patterns of imprinted and non-imprinted genes in cancer cell lines from multiple histologies
Source: Clin Epigenetics. 2025 May 25;17:83. doi: 10.1186/s13148-025-01883-3 (PMC12105275; doi:10.1186/s13148-025-01883-3)
Supplement: Supplementary file 8 — Supplementary Material 8. Figure S3. A flowchart of inference from VCF files of the expressed allele in monoallelically expressed isoforms and genes in 94 imprinted genes and 60 additional genes with predominantly monoallelic expression. Features, genes or isoforms. An alternative base was reported in the VCF files relative to the human hg19 genome reference sequence. [file 13148_2025_1883_MOESM8_ESM.pdf]

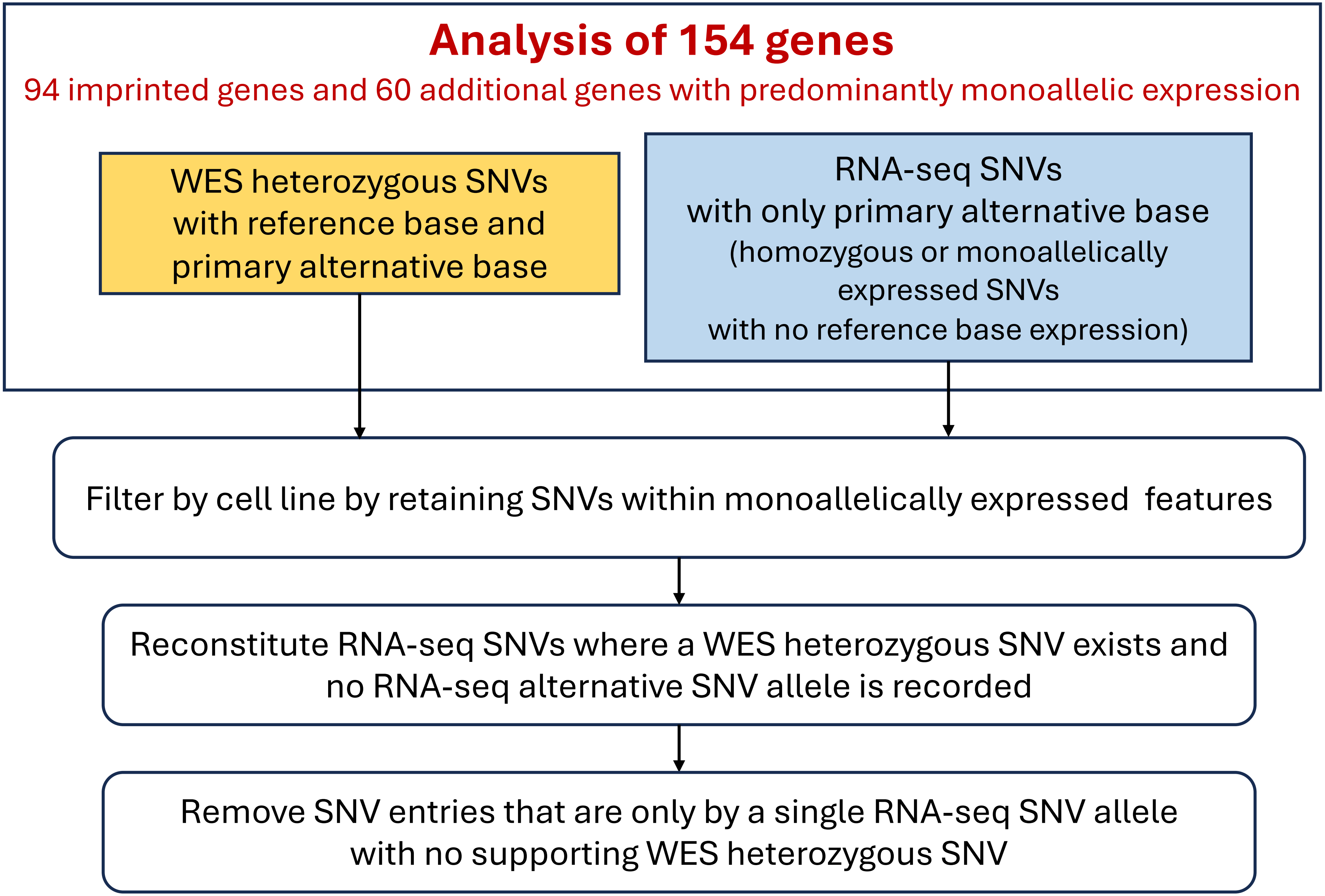
Analysis of 154 genes
94 imprinted genes and 60 additional genes with predominantly monoallelic expression
WES heterozygous SNVs with reference base and primary alternative base
RNA-seq SNVs with only primary alternative base (homozygous or monoallelically expressed SNVs with no reference base expression)
Filter by cell line by retaining SNVs within monoallelically expressed features
Reconstitute RNA-seq SNVs where a WES heterozygous SNV exists and no RNA-seq alternative SNV allele is recorded
Remove SNV entries that are only by a single RNA-seq SNV allele with no supporting WES heterozygous SNV

Fig. S3
