# Supplementary material for: Allelic expression patterns of imprinted and non-imprinted genes in cancer cell lines from multiple histologies
Source: Clin Epigenetics. 2025 May 25;17:83. doi: 10.1186/s13148-025-01883-3 (PMC12105275; doi:10.1186/s13148-025-01883-3)
Supplement: Supplementary file 10 — Supplementary Material 10. Figure S5. Comparison of the allelic expression patterns of the 94 imprinted vs 59,189 remaining genes at the (A) gene, (B) isoform, and (C) exon levels in each of the 9 cancer histologies of the 108 cell lines. Boxplots of the 94 imprinted genes listed in Additional file 2:Table S1 are represented by the lighter shades (left). Boxplots of the other genes representing the remaining 59,189 genes not included in the original list of 94 imprinted genes are shown by the darker shades of the same color for each tumor category (right). [file 13148_2025_1883_MOESM10_ESM.pdf]

Comparison of the whole gene level allelic expression patterns of 94 imprinted genes vs 59189 remaining genes

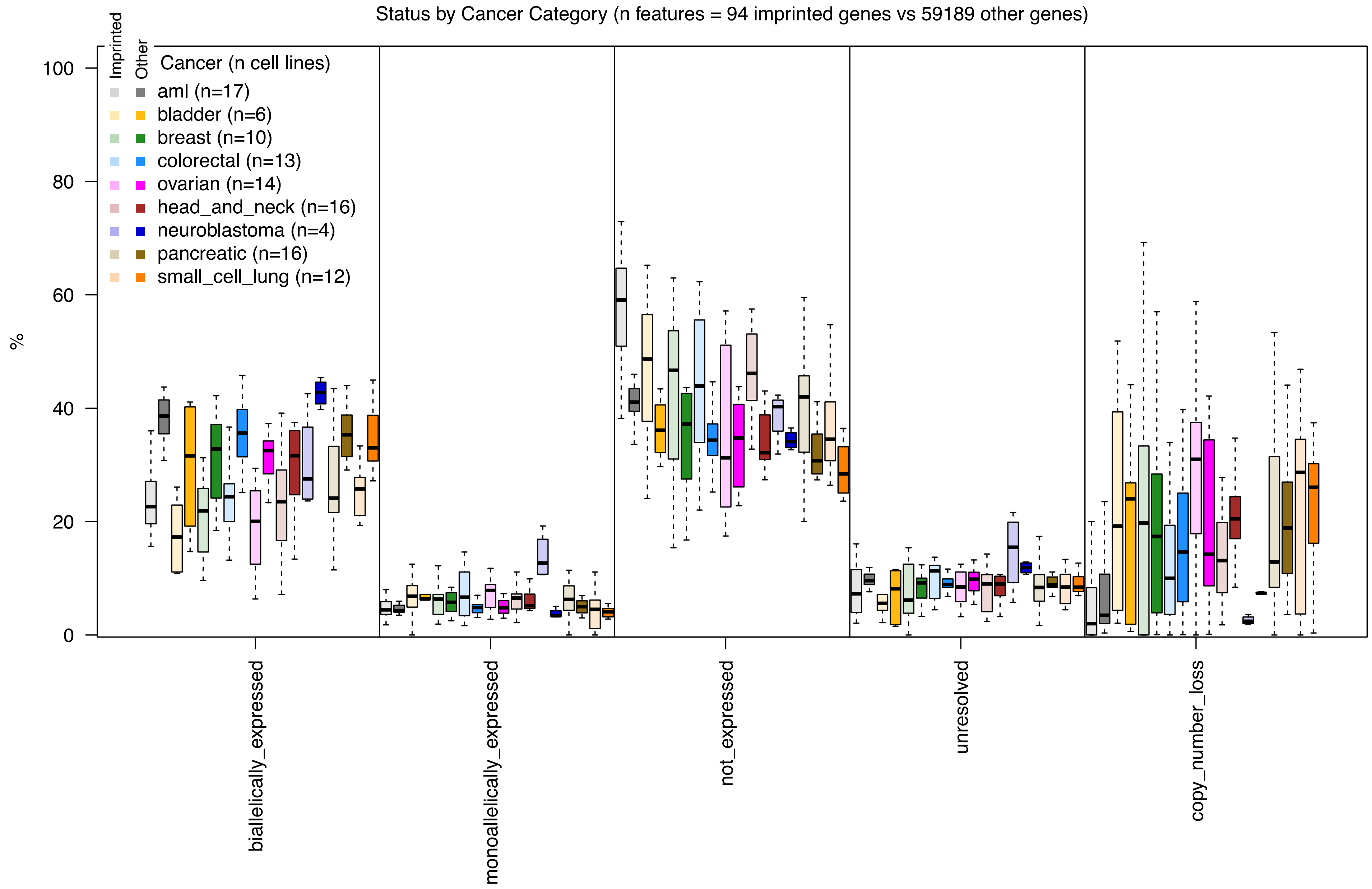

Fig. S5A

Comparison of the isoform level allelic expression patterns of 94 imprinted genes vs 59189 remaining genes

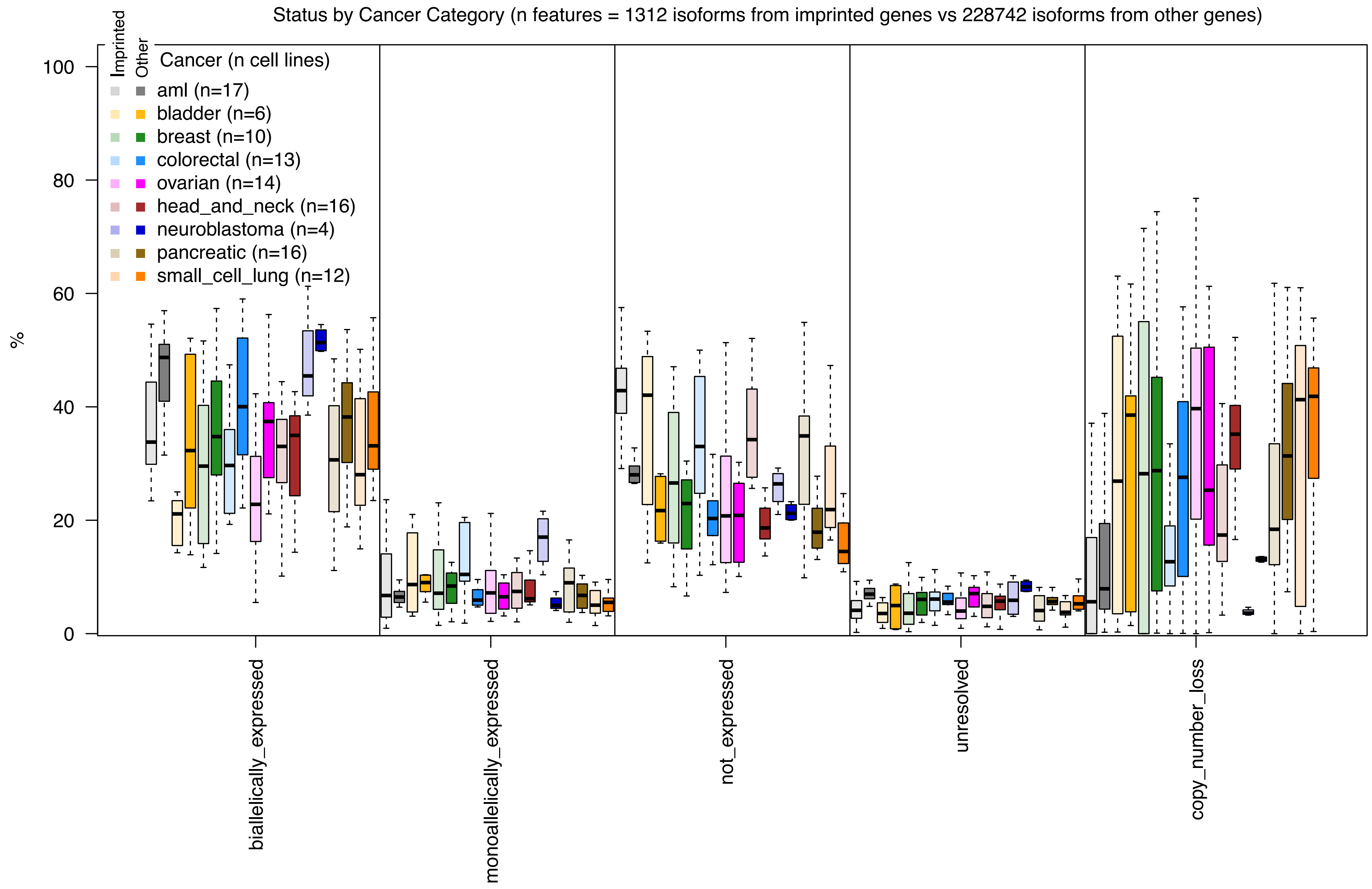

Fig. S5B

Comparison of the exon level allelic expression patterns of 94 imprinted genes vs 59189 remaining genes

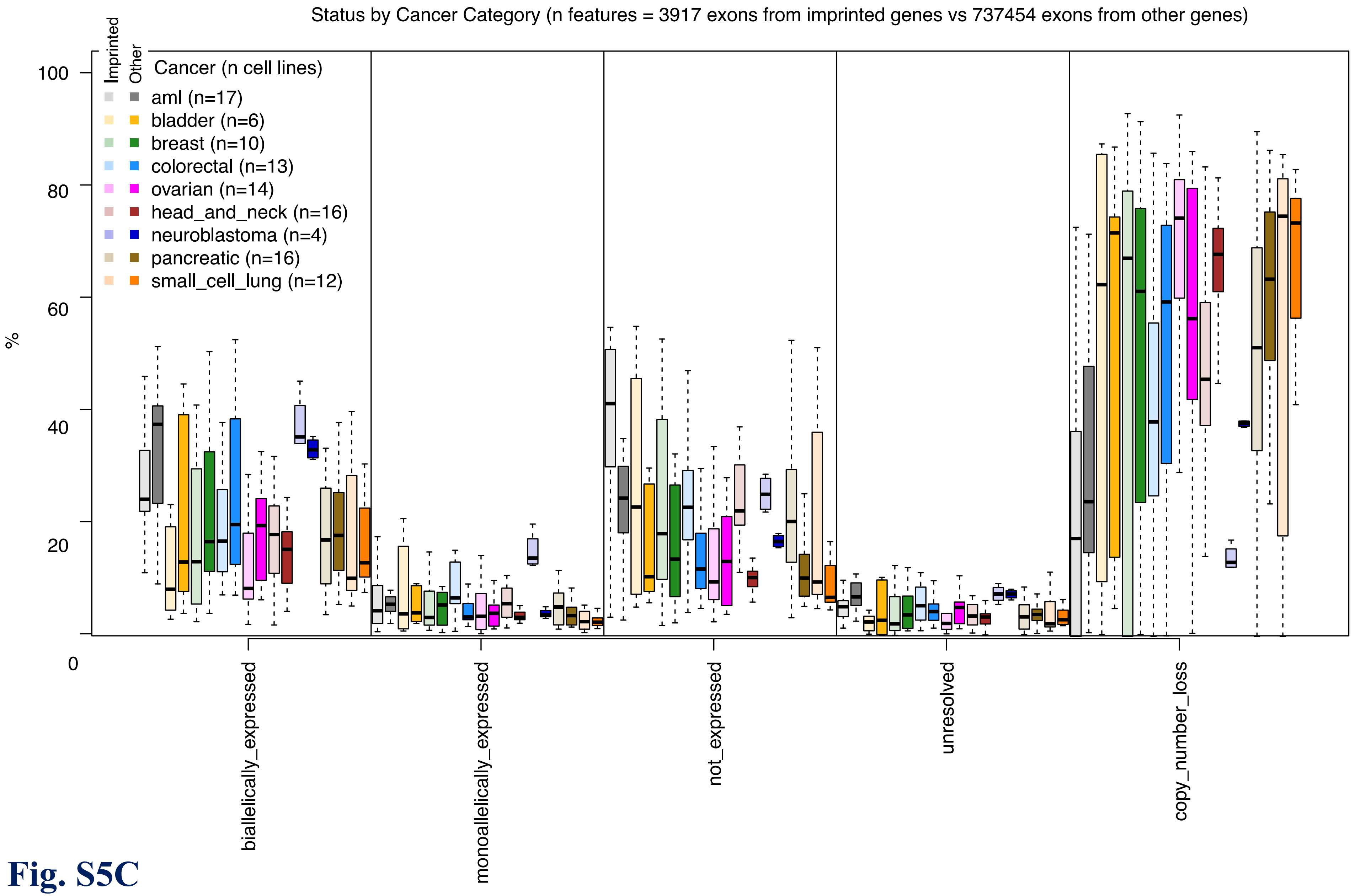

Fig. S5C
