# Supplementary material for: Allelic expression patterns of imprinted and non-imprinted genes in cancer cell lines from multiple histologies
Source: Clin Epigenetics. 2025 May 25;17:83. doi: 10.1186/s13148-025-01883-3 (PMC12105275; doi:10.1186/s13148-025-01883-3)
Supplement: Supplementary file 11 — Supplementary Material 11. Figure S6. Comparison of the gene level allelic expression patterns of the 94 imprinted genes vs average expression patterns in 94 genes that were resampled, using 1000 replications, from 59,189 remaining genes. Boxplots of the 94 imprinted genes listed in Additional file 2:Table S1 are represented by the lighter shades (left). Boxplots of the 94 subsampled genes are shown by the darker shades of the same color for each tumor category (right). [file 13148_2025_1883_MOESM11_ESM.pdf]

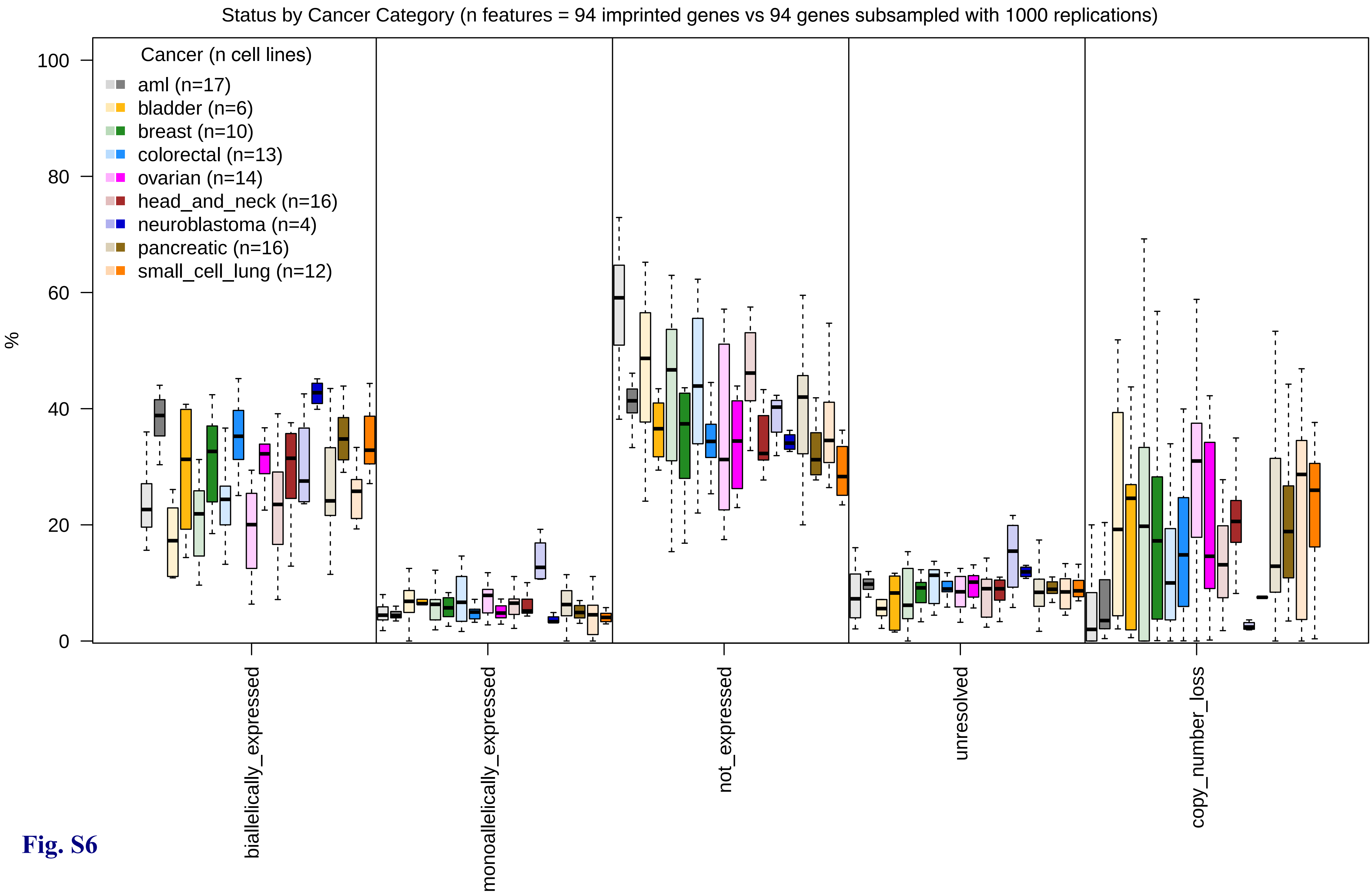
Status by Cancer Category (n features = 94 imprinted genes vs 94 genes subsampled with 1000 replications)
Cancer (n cell lines)
aml (n=17)
bladder (n=6)
breast (n=10)
colorectal (n=13)
ovarian (n=14)
head_and_neck (n=16)
neuroblastoma (n=4)
pancreatic (n=16)
small_cell_lung (n=12)
%
0
20
40
60
80
100
biallelically_expressed
monoallelically_expressed
not_expressed
unresolved
copy_number_loss

Fig. S6
