# Supplementary material for: Allelic expression patterns of imprinted and non-imprinted genes in cancer cell lines from multiple histologies
Source: Clin Epigenetics. 2025 May 25;17:83. doi: 10.1186/s13148-025-01883-3 (PMC12105275; doi:10.1186/s13148-025-01883-3)
Supplement: Supplementary file 13 — Supplementary Material 13. Figure S7. Heatmap of tumor histology-specific monoallelic expression among the 94 imprinted genes. For each gene and tumor category, shown is the proportion of monoallelically expressed genes among monoallelically or biallelically expressed genes, at the whole gene level. Dendrograms were inferred using Euclidian distances and complete linkage clustering. Biallelic only expression (values of 0 monoallelic and 1 biallelic counts) is presented by light blue color. N/A, cases with 0 monoallelic and 0 biallelic counts, are shown as grey color. Coloring of > 0 to 1 is on a 100 color gradient. [file 13148_2025_1883_MOESM13_ESM.pdf]

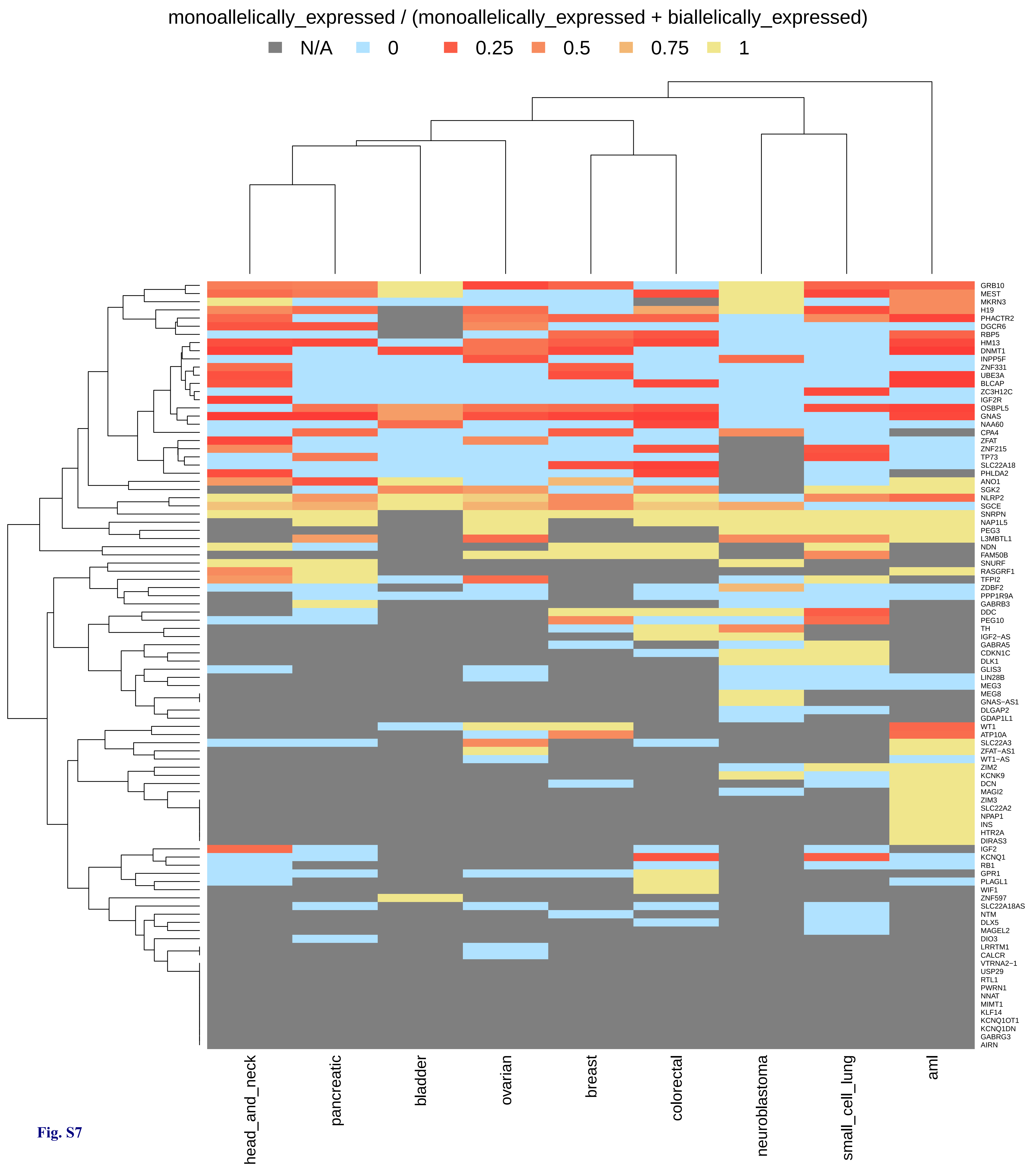

monoallelically_expressed / (monoallelically_expressed + biallelically_expressed)
N/A
0
0.25
0.5
0.75
1
GRB10
MEST
MKRN3
H19
PHACTR2
DGCR6
RBP5
HM13
DNMT1
INPP5F
ZNF331
UBE3A
BLCAP
ZC3H12C
IGF2R
OSBPL5
GNAS
NAA60
CPA4
ZFAT
ZNF215
TP73
SLC22A18
PHLDA2
ANO1
SGK2
NLRP2
SGCE
SNRPN
NAP1L5
PEG3
L3MBTL1
NDN
FAM50B
SNURF
RASGRF1
TFPI2
ZDBF2
PPP1R9A
GABRB3
DDC
PEG10
TH
IGF2−AS
GABRA5
CDKN1C
DLK1
GLIS3
LIN28B
MEG3
MEG8
GNAS−AS1
DLGAP2
GDAP1L1
WT1
ATP10A
SLC22A3
ZFAT−AS1
WT1−AS
ZIM2
KCNK9
DCN
MAGI2
ZIM3
SLC22A2
NPAP1
INS
HTR2A
DIRAS3
IGF2
KCNQ1
RB1
GPR1
PLAGL1
WIF1
ZNF597
SLC22A18AS
NTM
DLX5
MAGEL2
DIO3
LRRTM1
CALCR
VTRNA2−1
USP29
RTL1
PWRN1
NNAT
MIMT1
KLF14
KCNQ1OT1
KCNQ1DN
GABRG3
AIRN
head_and_neck
pancreatic
bladder
ovarian
breast
colorectal
neuroblastoma
small_cell_lung
aml

Fig. S7
