# Supplementary material for: Allelic expression patterns of imprinted and non-imprinted genes in cancer cell lines from multiple histologies
Source: Clin Epigenetics. 2025 May 25;17:83. doi: 10.1186/s13148-025-01883-3 (PMC12105275; doi:10.1186/s13148-025-01883-3)
Supplement: Supplementary file 16 — Supplementary Material 16. Figure S8. Comparison of the allelic expression patterns of the 94 imprinted genes vs 60 additional predominantly monoallelically expressed genes at the (A) gene, (B) isoform, and (C) exon levels in each of the 9 cancer histologies of the 108 cell lines. Boxplots of the 94 imprinted genes listed in Additional file 2:Table S1 are represented by the lighter shades (left, marked as Imprinted in the legend). Boxplots of the 60 additional predominantly monoallelically expressed genes listed in Additional file 6:Table S4 are shown by the darker shades of the same color for each tumor category (right, marked as Mono in the legend). [file 13148_2025_1883_MOESM16_ESM.pdf]

Comparison of the whole gene level allelic expression patterns of 94 imprinted genes vs 60 additional predominantly monoallelically expressed genes

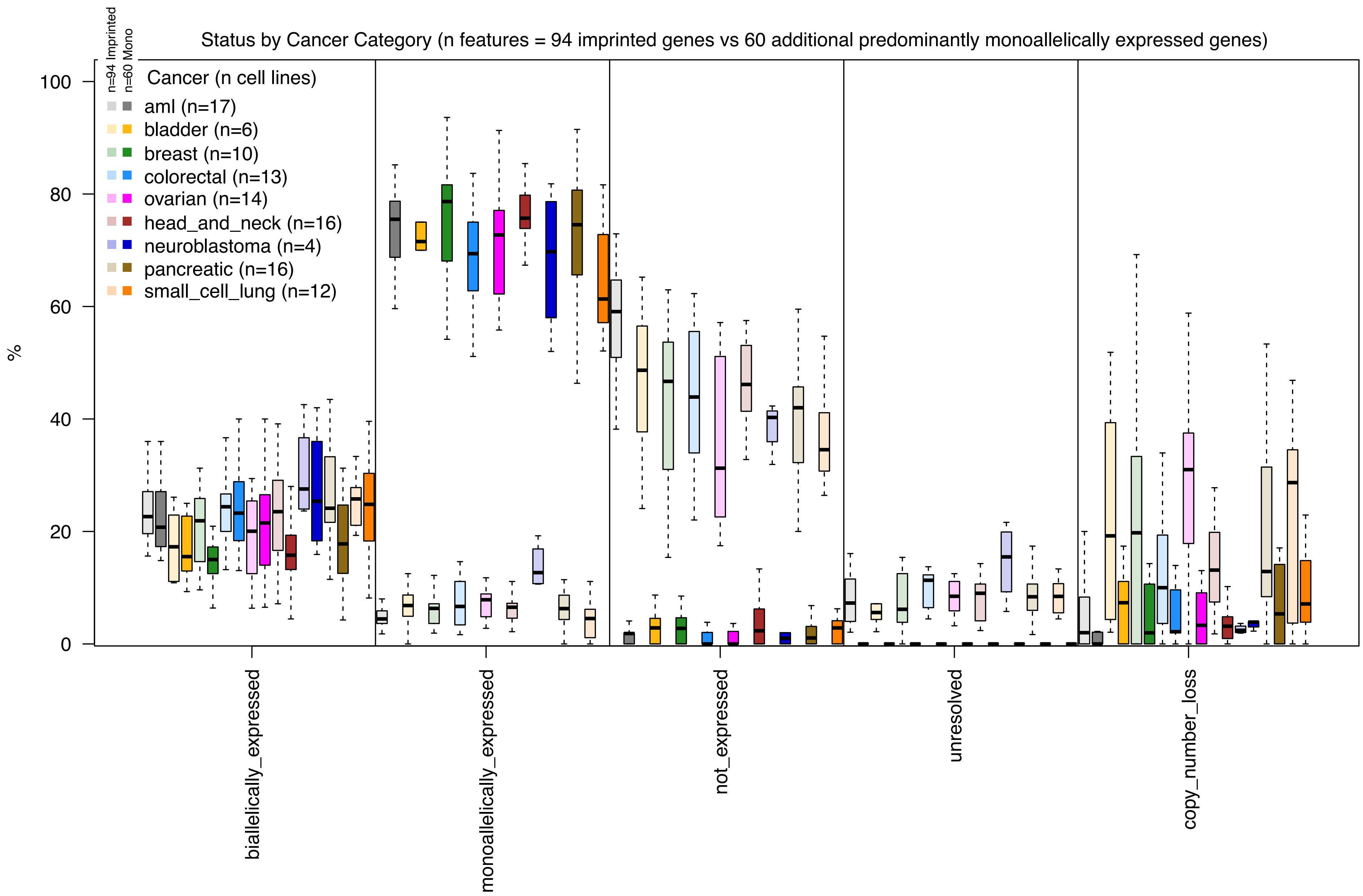

Fig. S8A

Comparison of the isoform level allelic expression patterns of 94 imprinted genes vs 60 additional predominantly monoallelically expressed genes

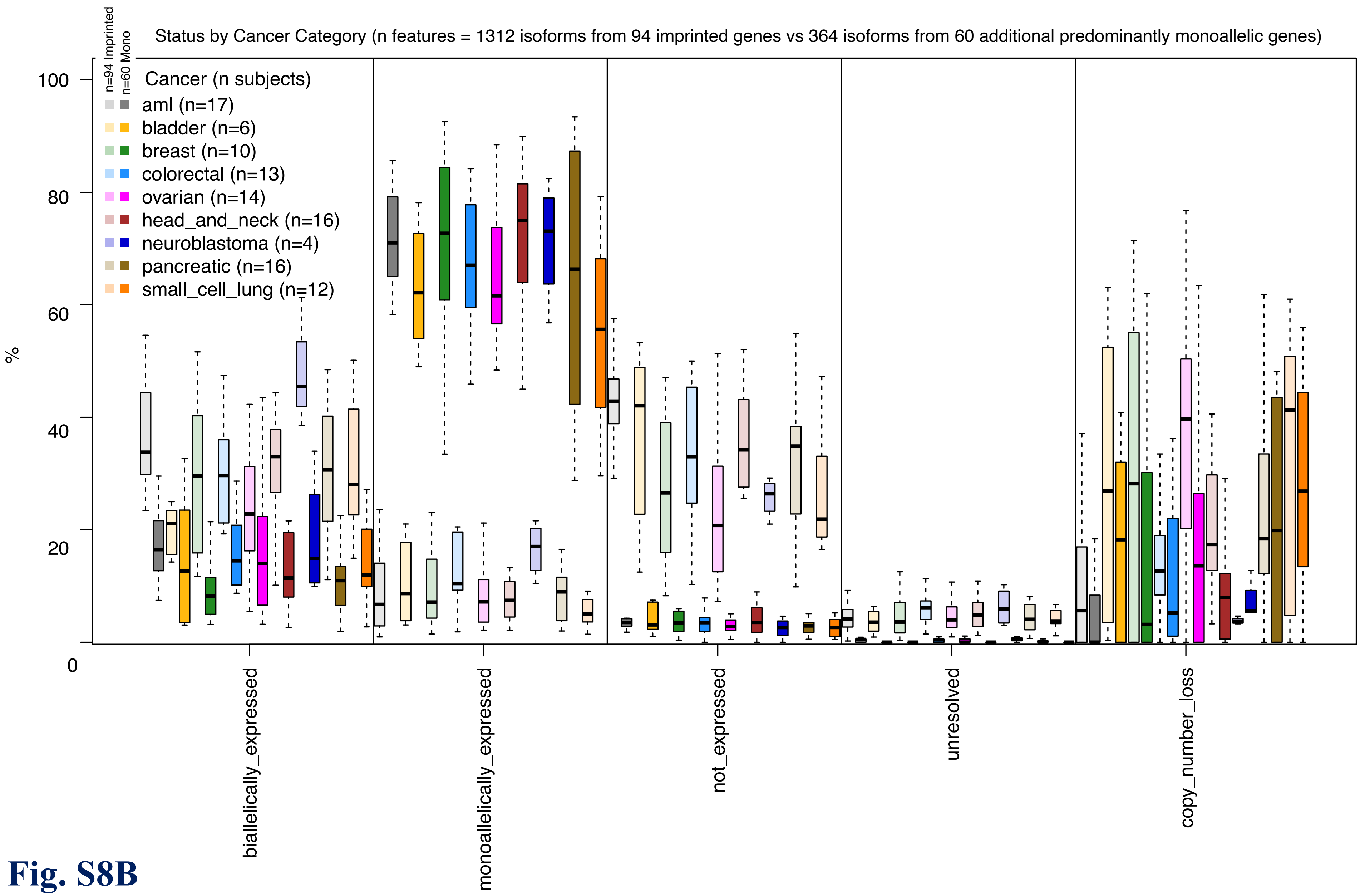

Fig. S8B

Comparison of the exon level allelic expression patterns of 94 imprinted genes vs 60 additional predominantly monoallelically expressed genes

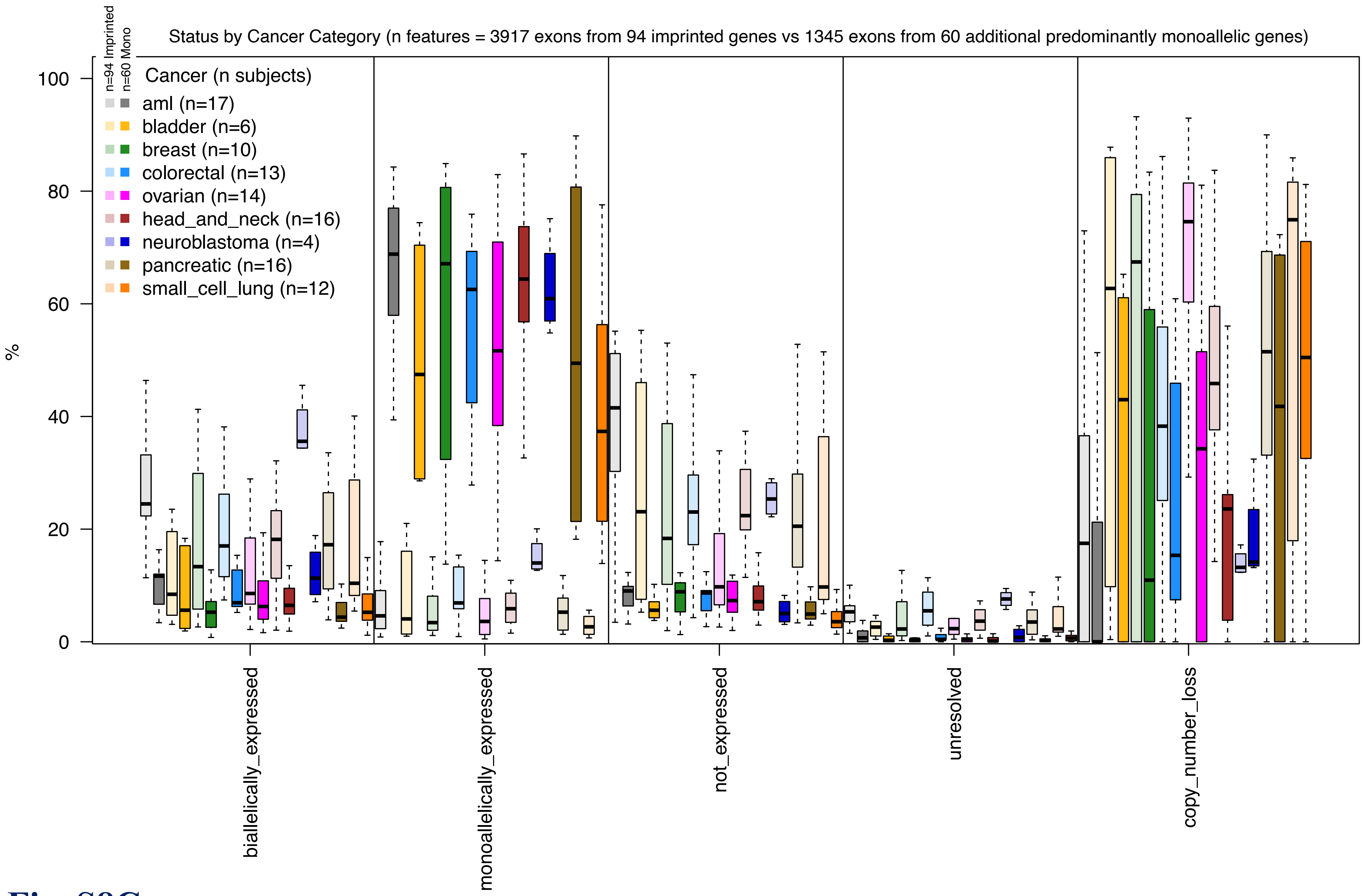

Fig. S8C
