# Supplementary material for: Allelic expression patterns of imprinted and non-imprinted genes in cancer cell lines from multiple histologies
Source: Clin Epigenetics. 2025 May 25;17:83. doi: 10.1186/s13148-025-01883-3 (PMC12105275; doi:10.1186/s13148-025-01883-3)
Supplement: Supplementary file 21 — Supplementary Material 21. Figure S10. Violin plots for associated agents and expression patterns of the isoform groups satisfying pFDR < 0.05 for the 94 imprinted genes. [file 13148_2025_1883_MOESM21_ESM.pdf]

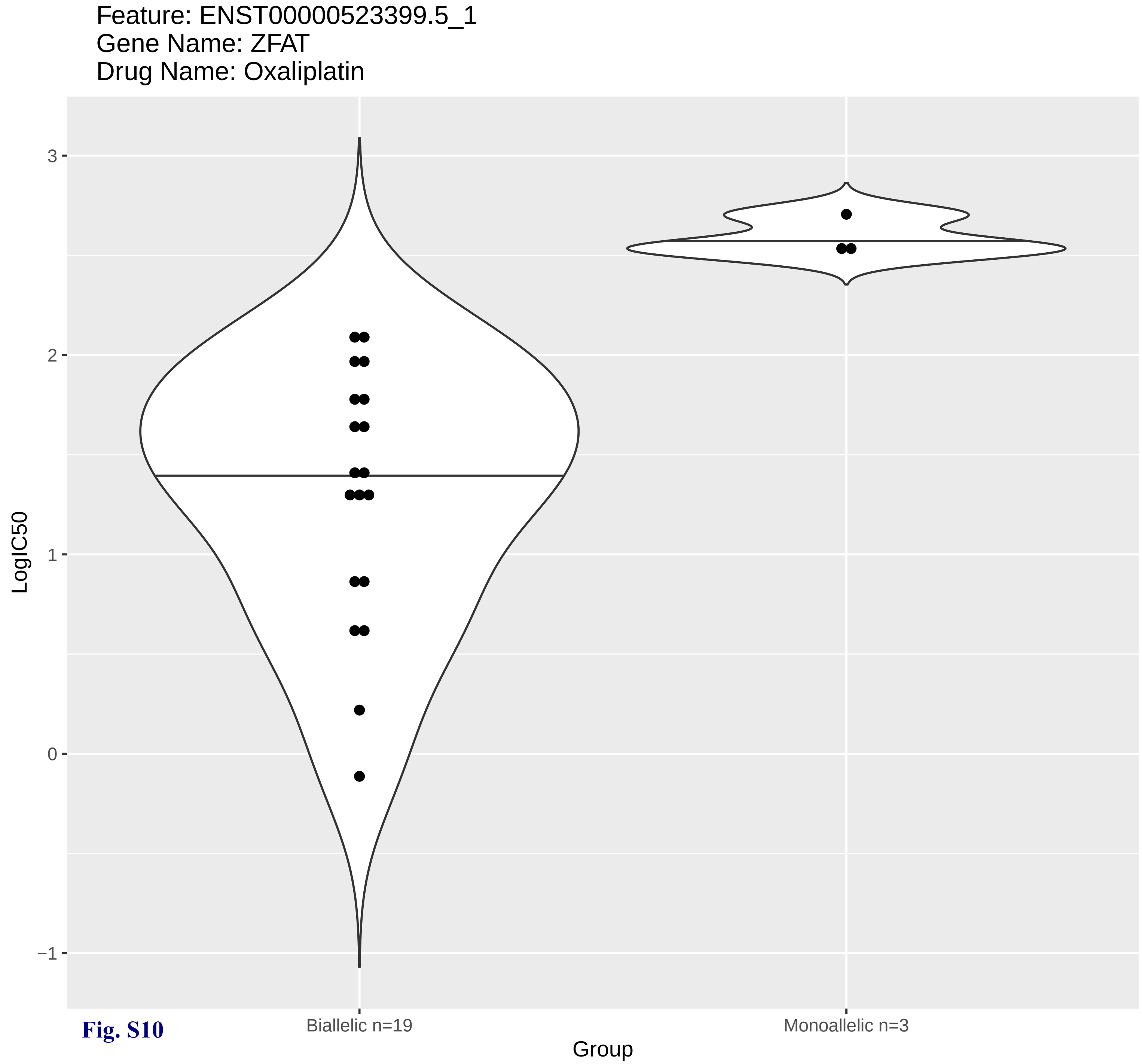
Feature: ENST00000523399.5_1
Gene Name: ZFAT
Drug Name: Oxaliplatin
3
2
1
0
−1
LogIC50
Biallelic n=19
Monoallelic n=3
Group
Fig. S10

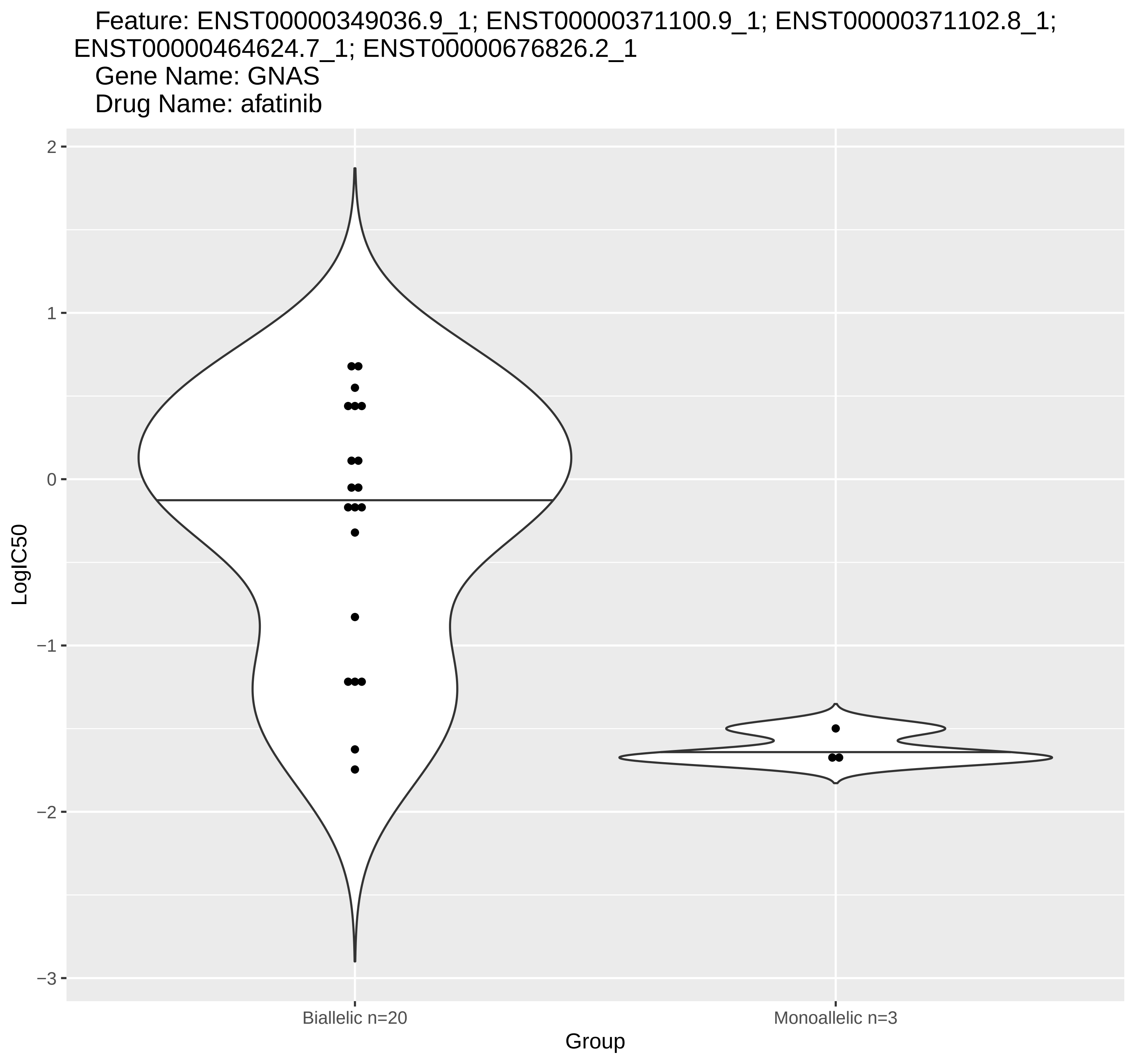

Feature: ENST00000349036.9_1; ENST00000371100.9_1; ENST00000371102.8_1; ENST00000464624.7_1; ENST00000676826.2_1
Gene Name: GNAS
Drug Name: afatinib
LogIC50
2
1
0
−1
−2
−3
Biallelic n=20
Monoallelic n=3
Group

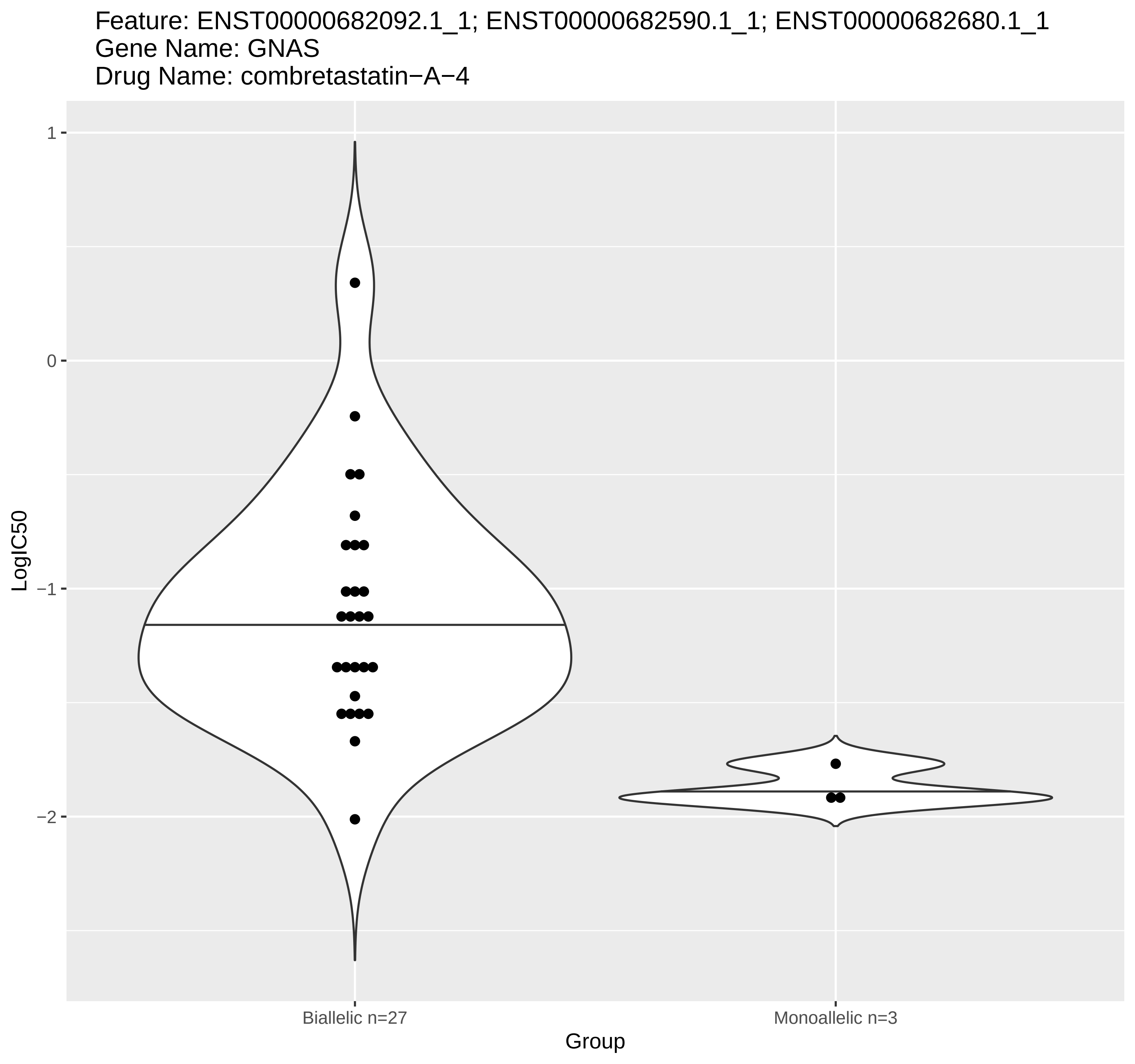
Feature: ENST00000682092.1_1; ENST00000682590.1_1; ENST00000682680.1_1
Gene Name: GNAS
Drug Name: combretastatin−A−4
LogIC50
1
0
−1
−2
Biallelic n=27
Monoallelic n=3
Group

Feature: ENST00000682986.1_1; ENST00000683632.1_1; ENST00000684644.1_1
Gene Name: GNAS
Drug Name: combretastatin−A−4

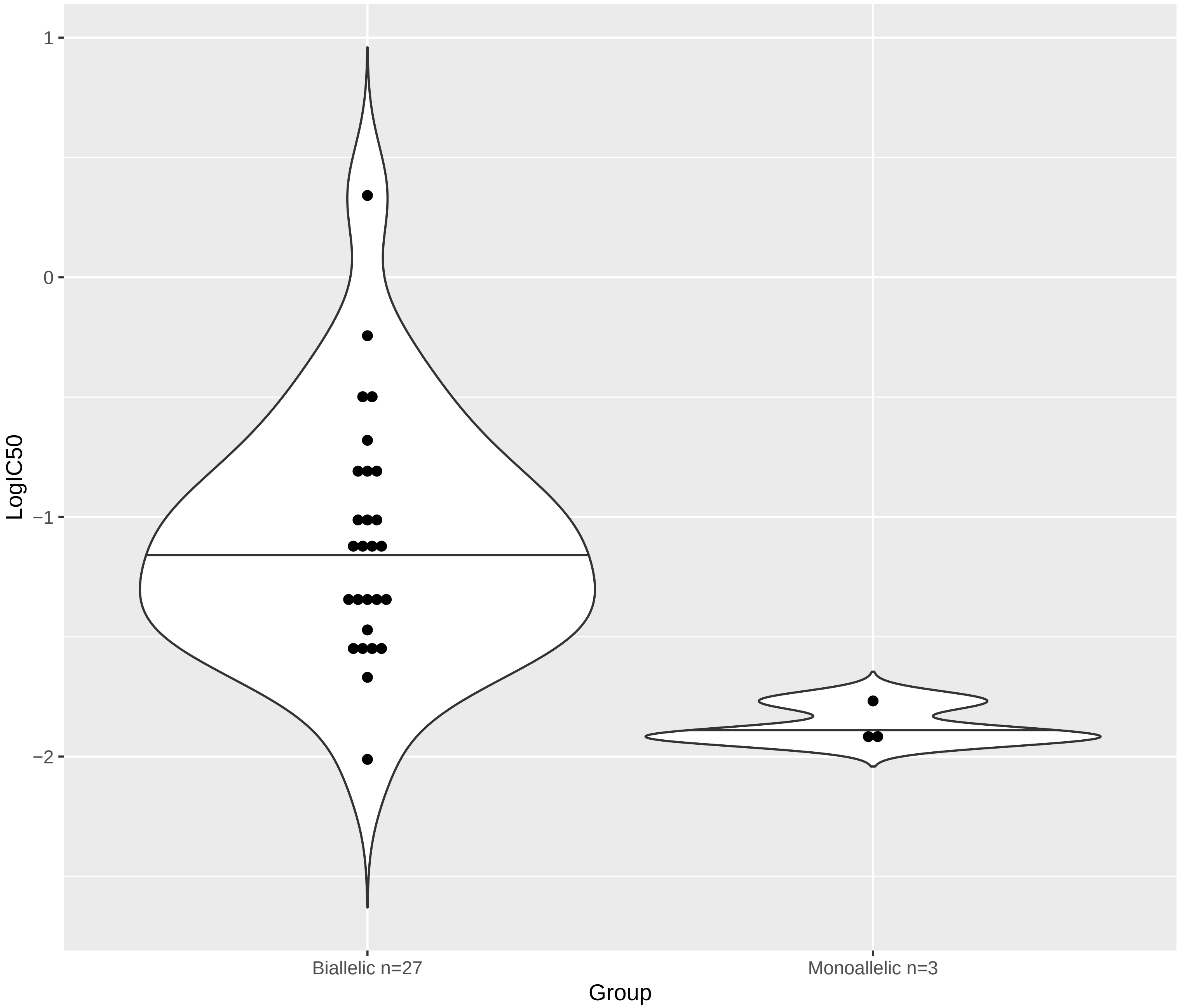

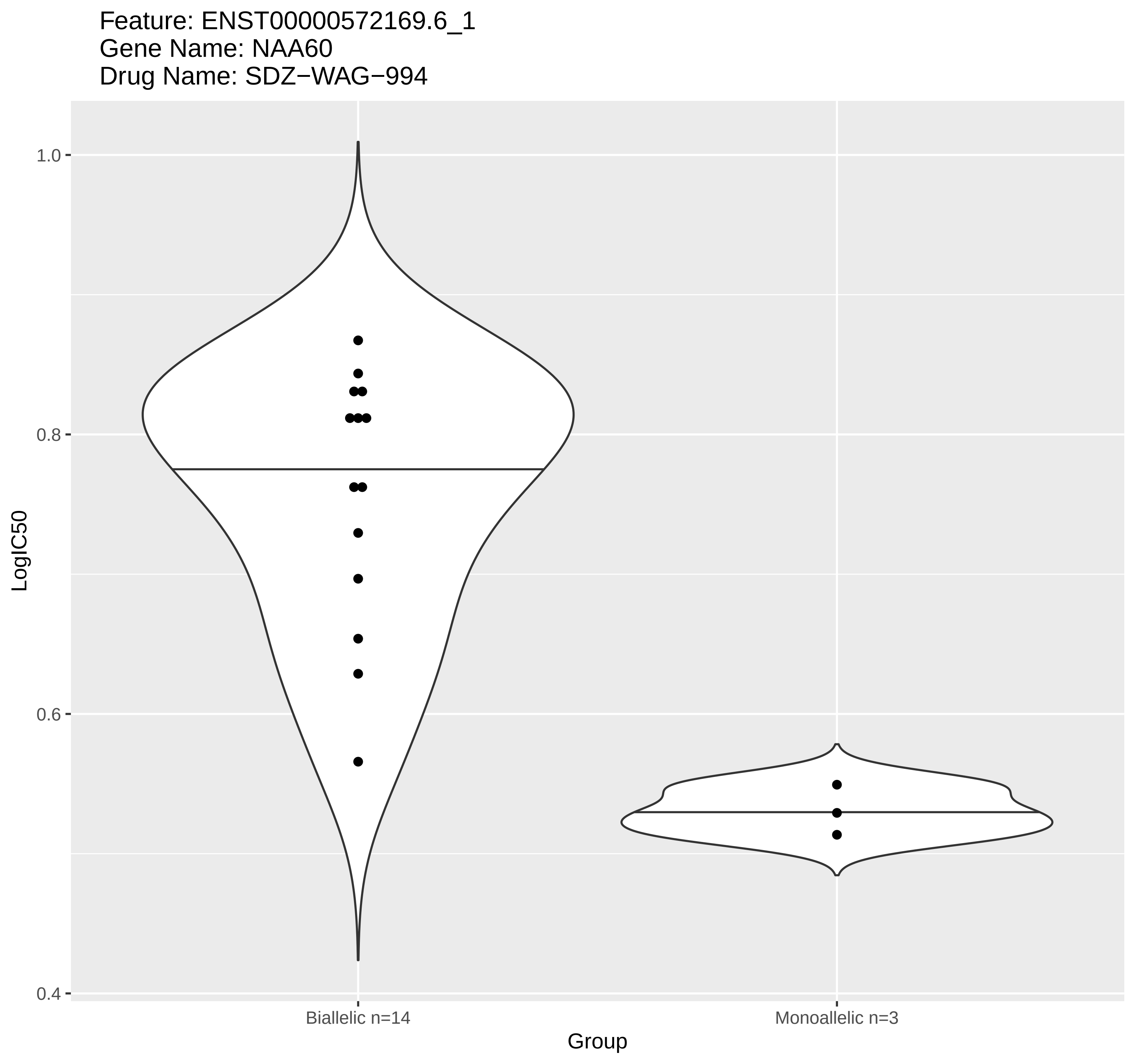
Feature: ENST00000572169.6_1
Gene Name: NAA60
Drug Name: SDZ−WAG−994
LogIC50
1.0
0.8
0.6
0.4
Biallelic n=14
Monoallelic n=3
Group

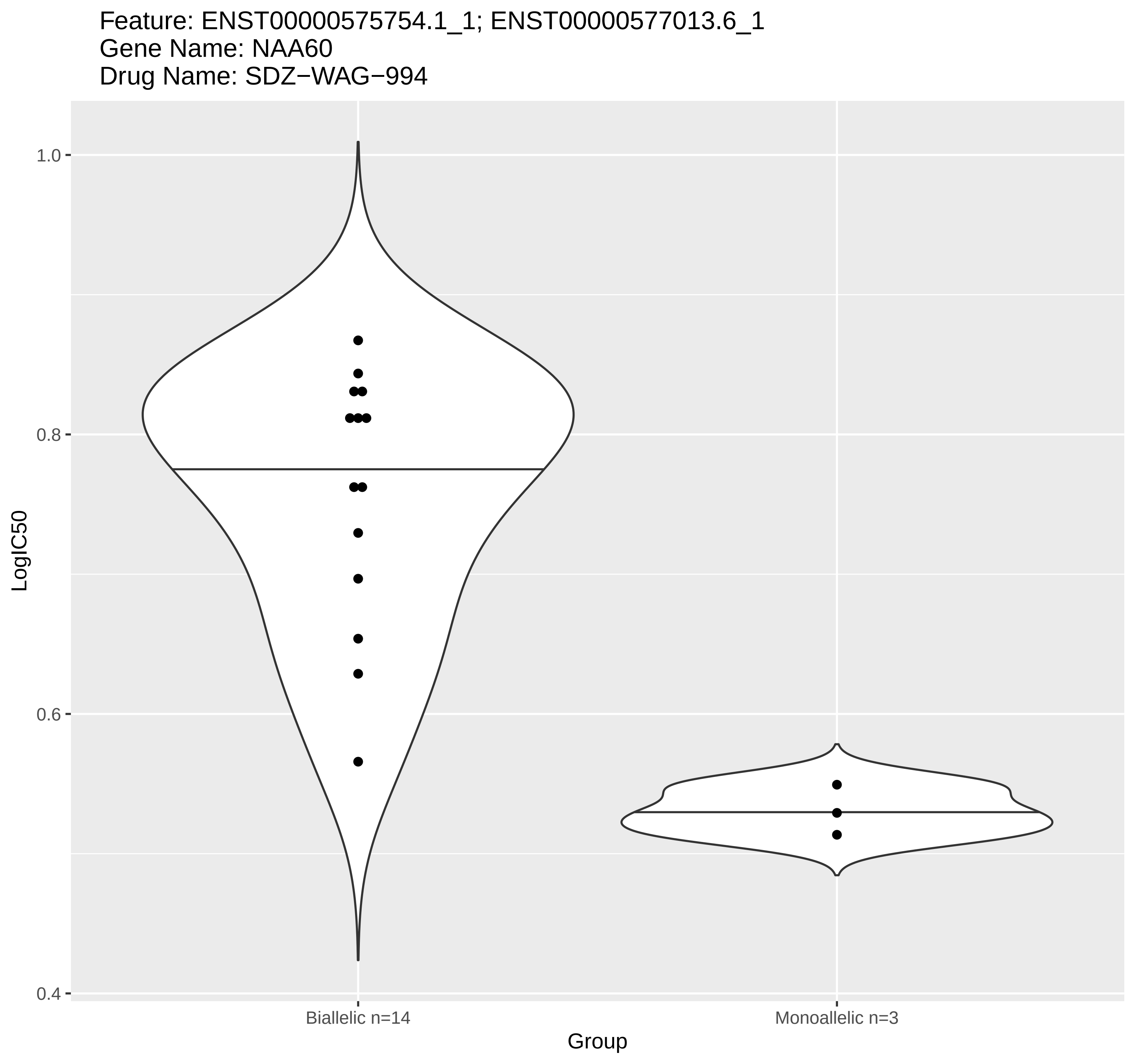

Feature: ENST00000575754.1_1; ENST00000577013.6_1
Gene Name: NAA60
Drug Name: SDZ−WAG−994
1.0
0.8
0.6
0.4
LogIC50
Biallelic n=14
Monoallelic n=3
Group

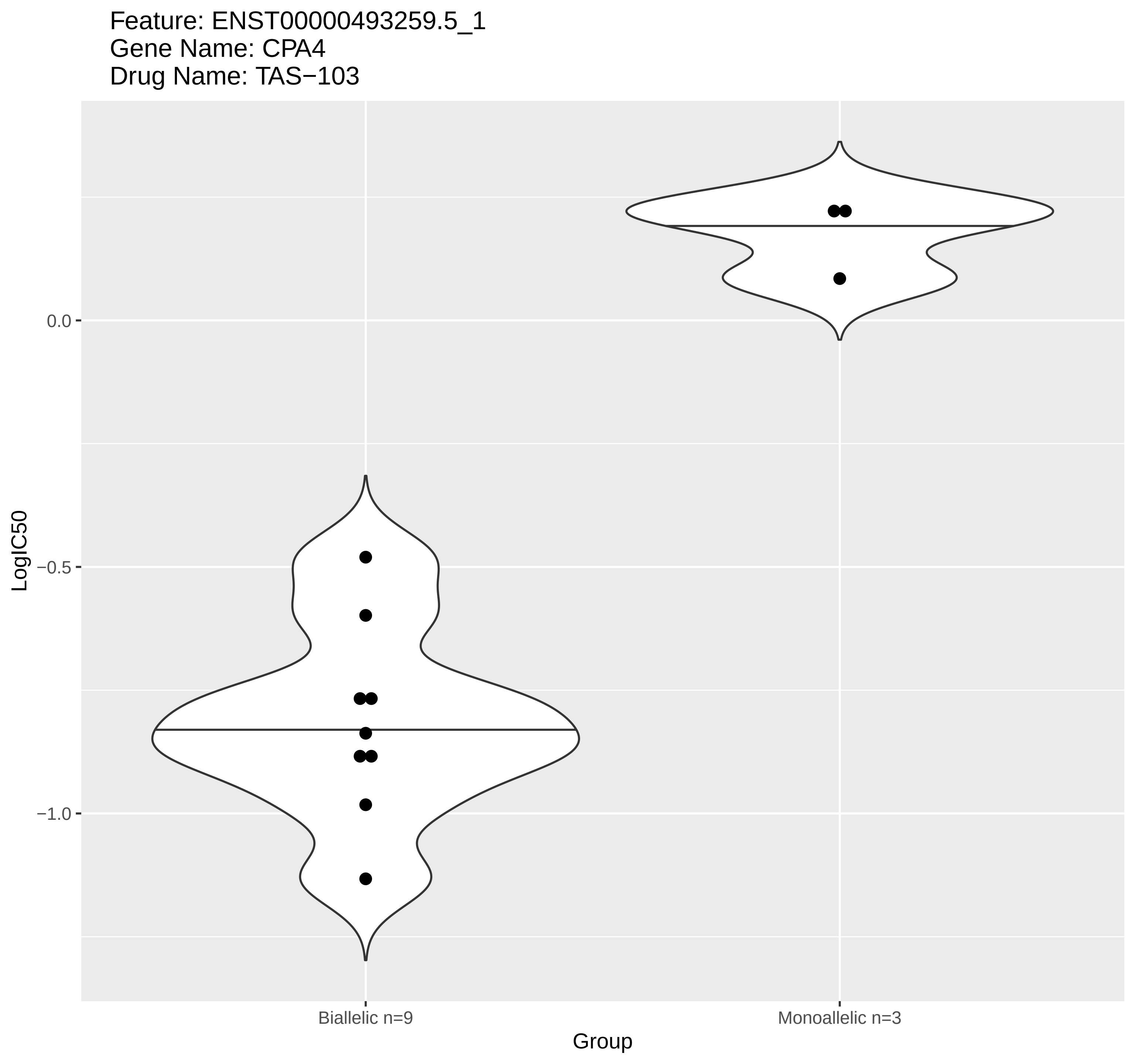
Feature: ENST00000493259.5_1
Gene Name: CPA4
Drug Name: TAS−103
LogIC50
0.0
−0.5
−1.0
Biallelic n=9
Monoallelic n=3
Group

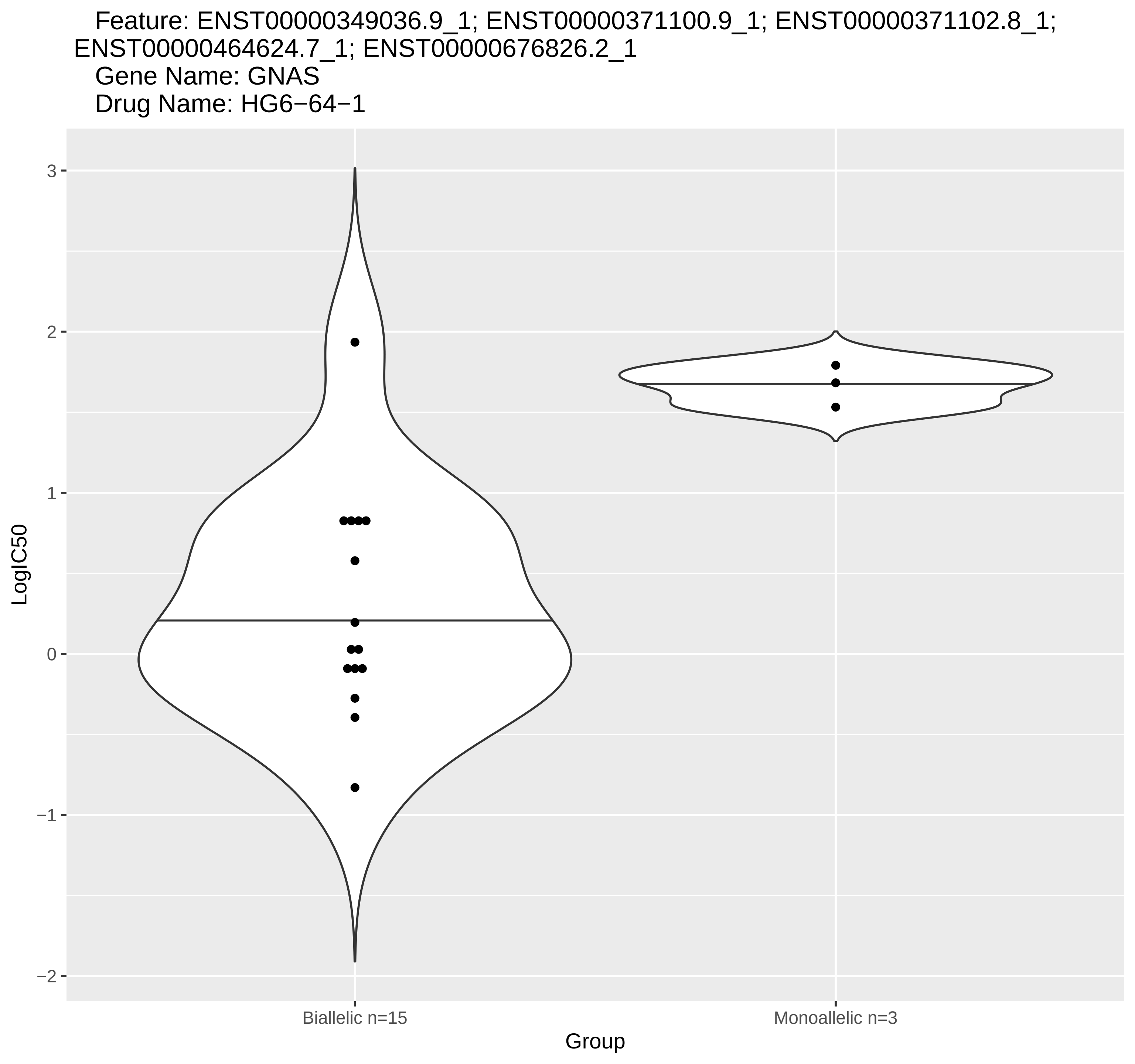

Feature: ENST00000349036.9_1; ENST00000371100.9_1; ENST00000371102.8_1; ENST00000464624.7_1; ENST00000676826.2_1
Gene Name: GNAS
Drug Name: HG6−64−1
LogIC50
3
2
1
0
−1
−2
Biallelic n=15
Monoallelic n=3
Group

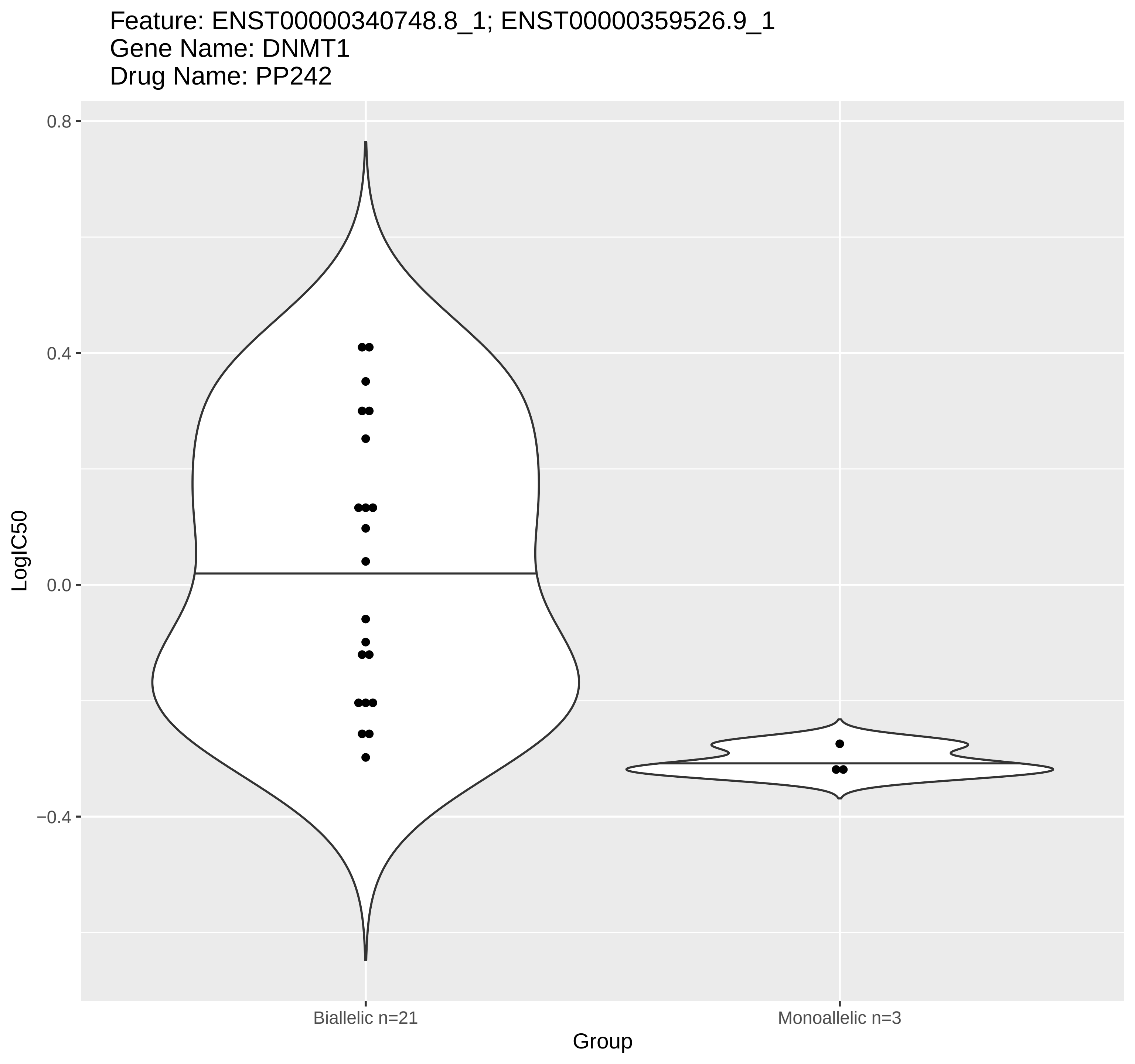
Feature: ENST00000340748.8_1; ENST00000359526.9_1
Gene Name: DNMT1
Drug Name: PP242
LogIC50
0.8
0.4
0.0
−0.4
Biallelic n=21
Monoallelic n=3
Group

Feature: ENST00000676820.1_1
Gene Name: DNMT1
Drug Name: PP242

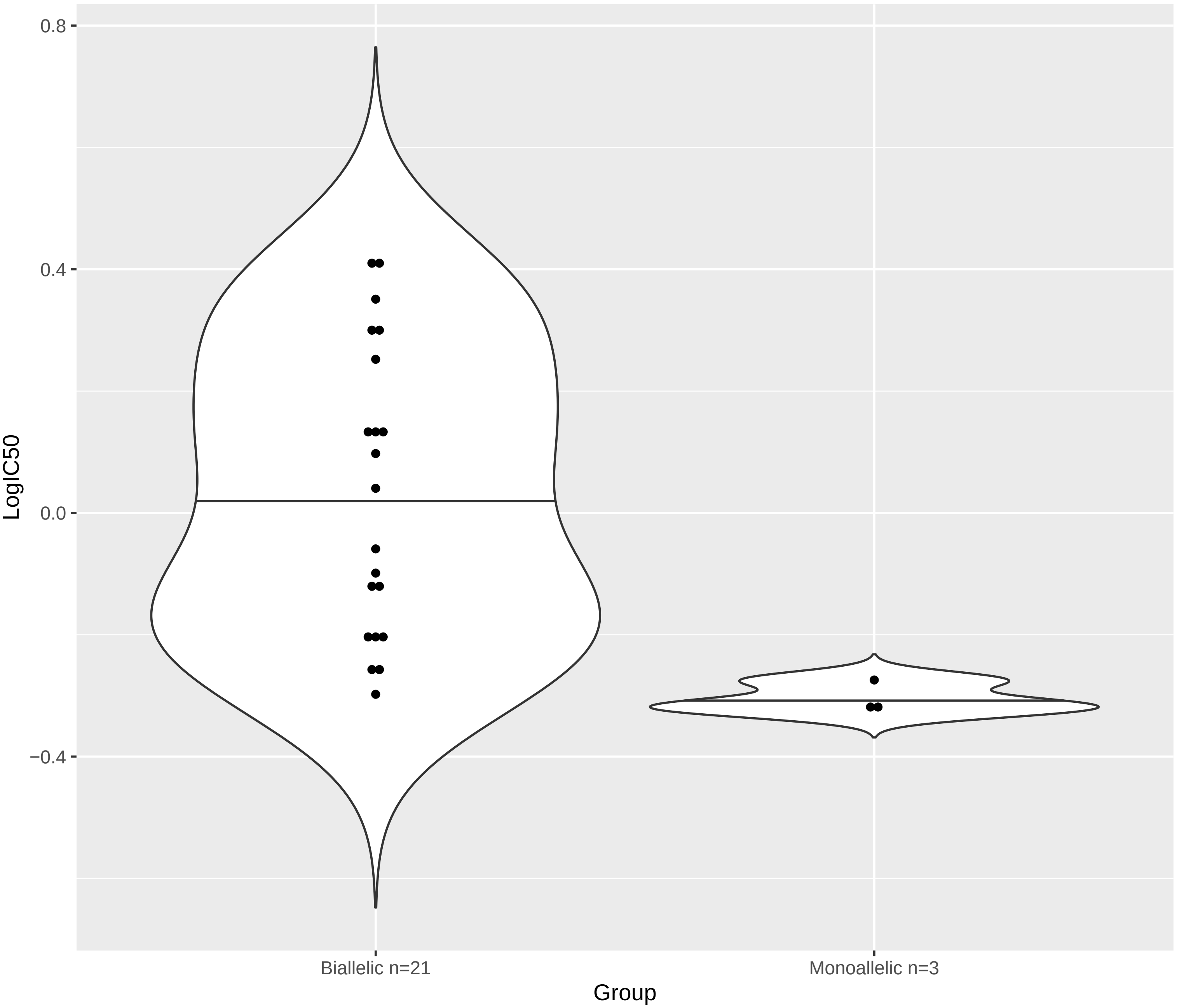

Feature: ENST00000677013.1_1; ENST00000677250.1_1; ENST00000677634.1_1; ENST00000677946.1_1; ENST00000678804.1_1; ENST00000679103.1_1; ENST00000679313.1_1
Gene Name: DNMT1
Drug Name: PP242

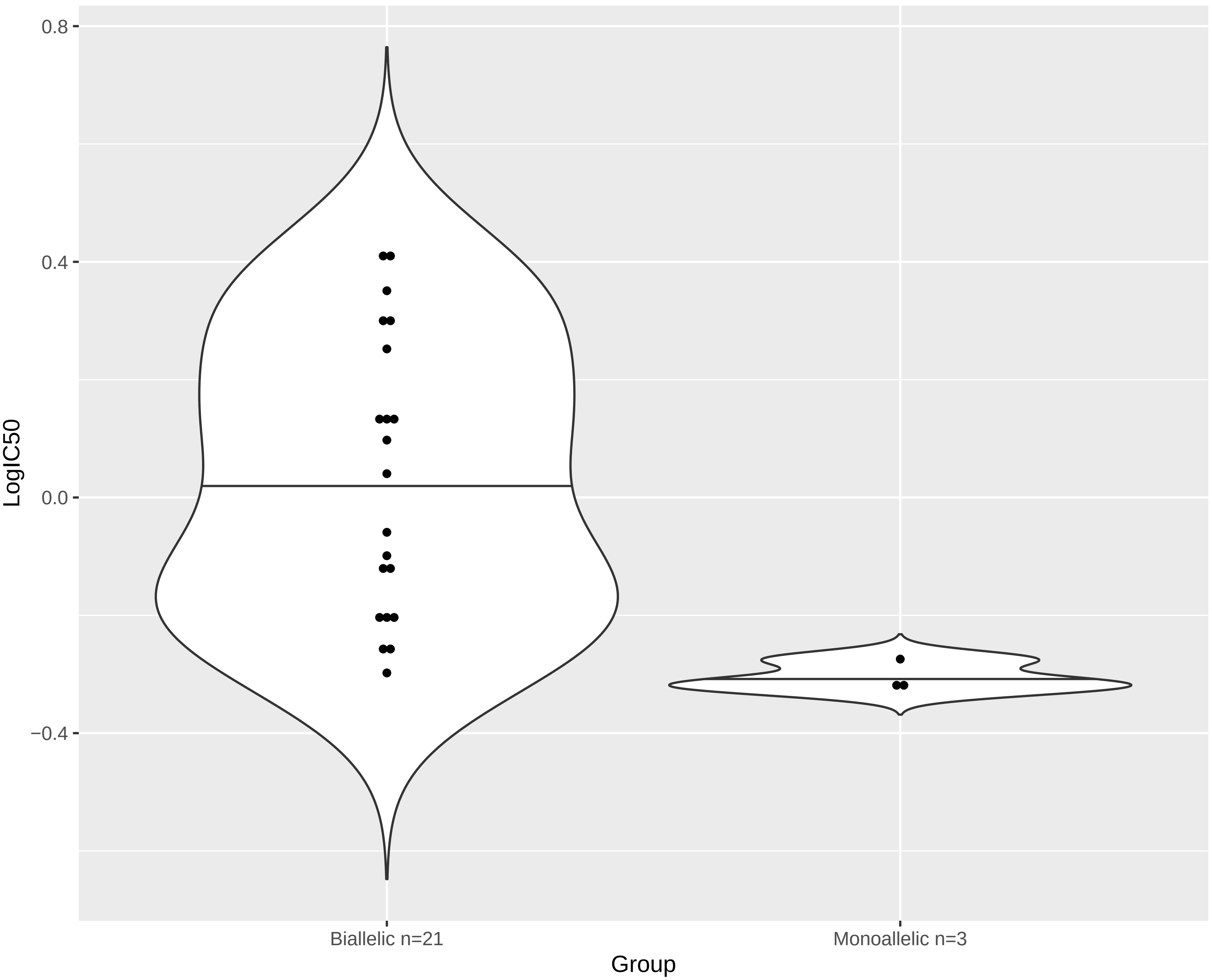

Feature: ENST00000447298.2_1
Gene Name: H19
Drug Name: PP242

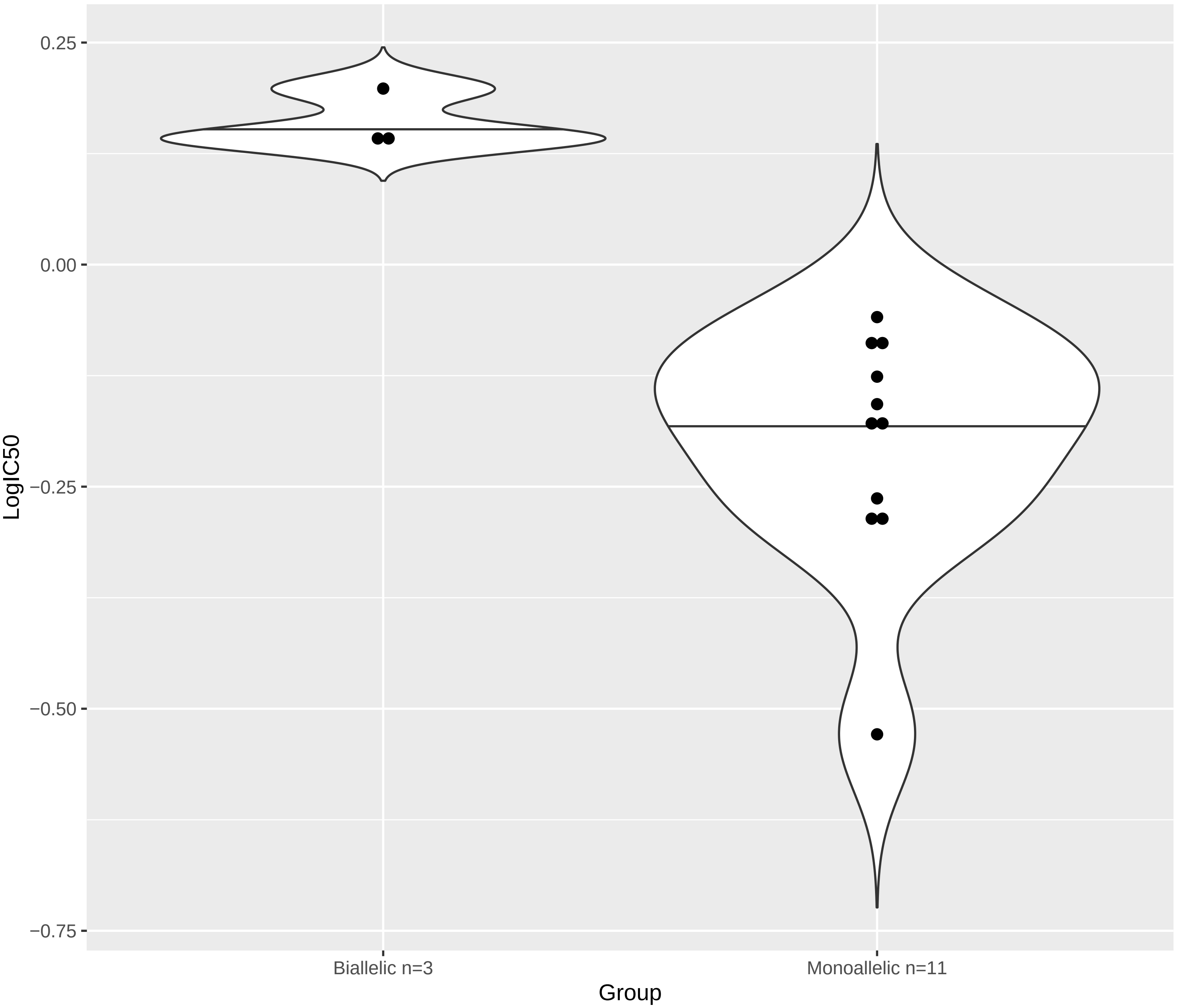

Feature: ENST00000424546.6_1
Gene Name: NAA60
Drug Name: SDZ−WAG−994

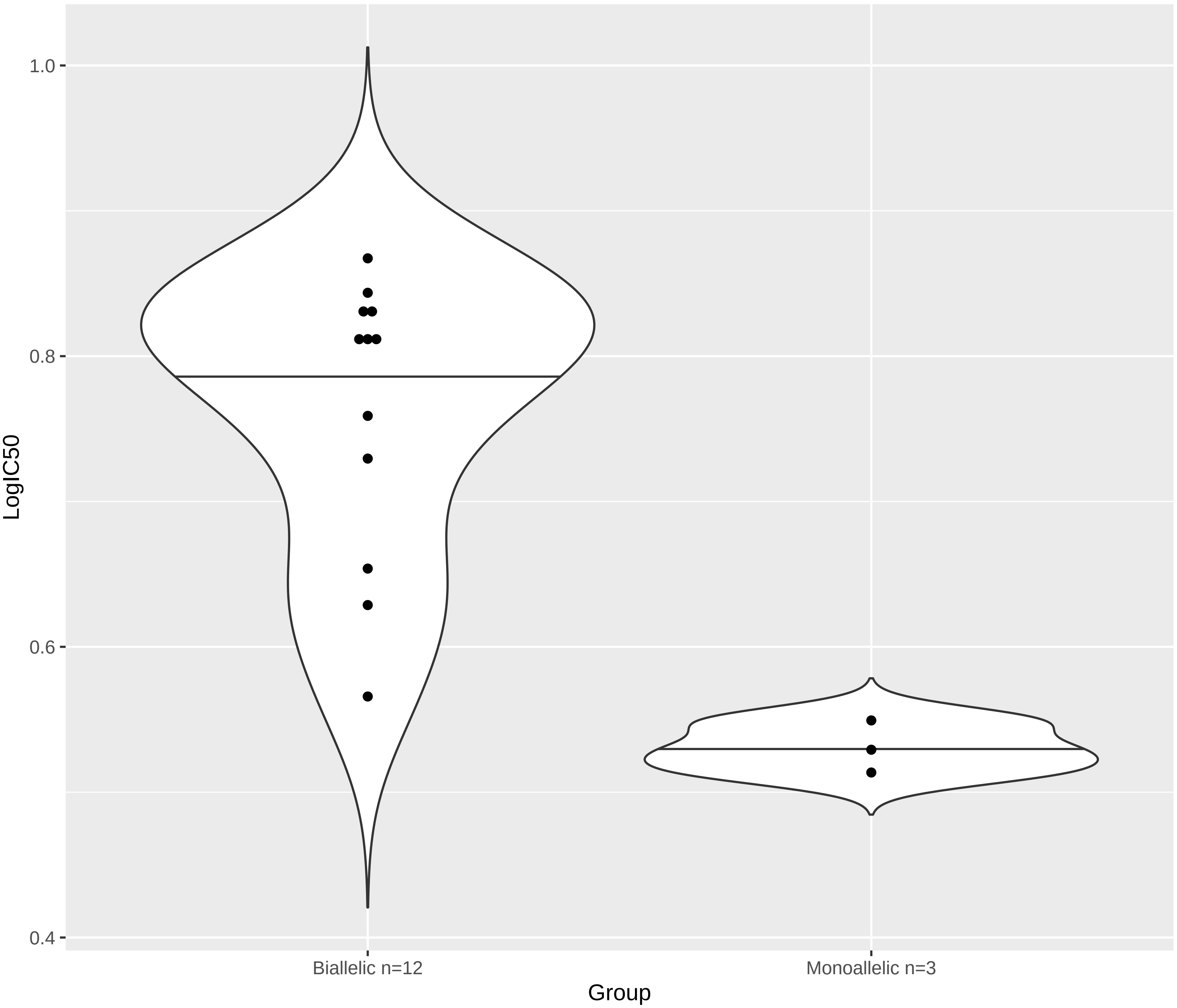

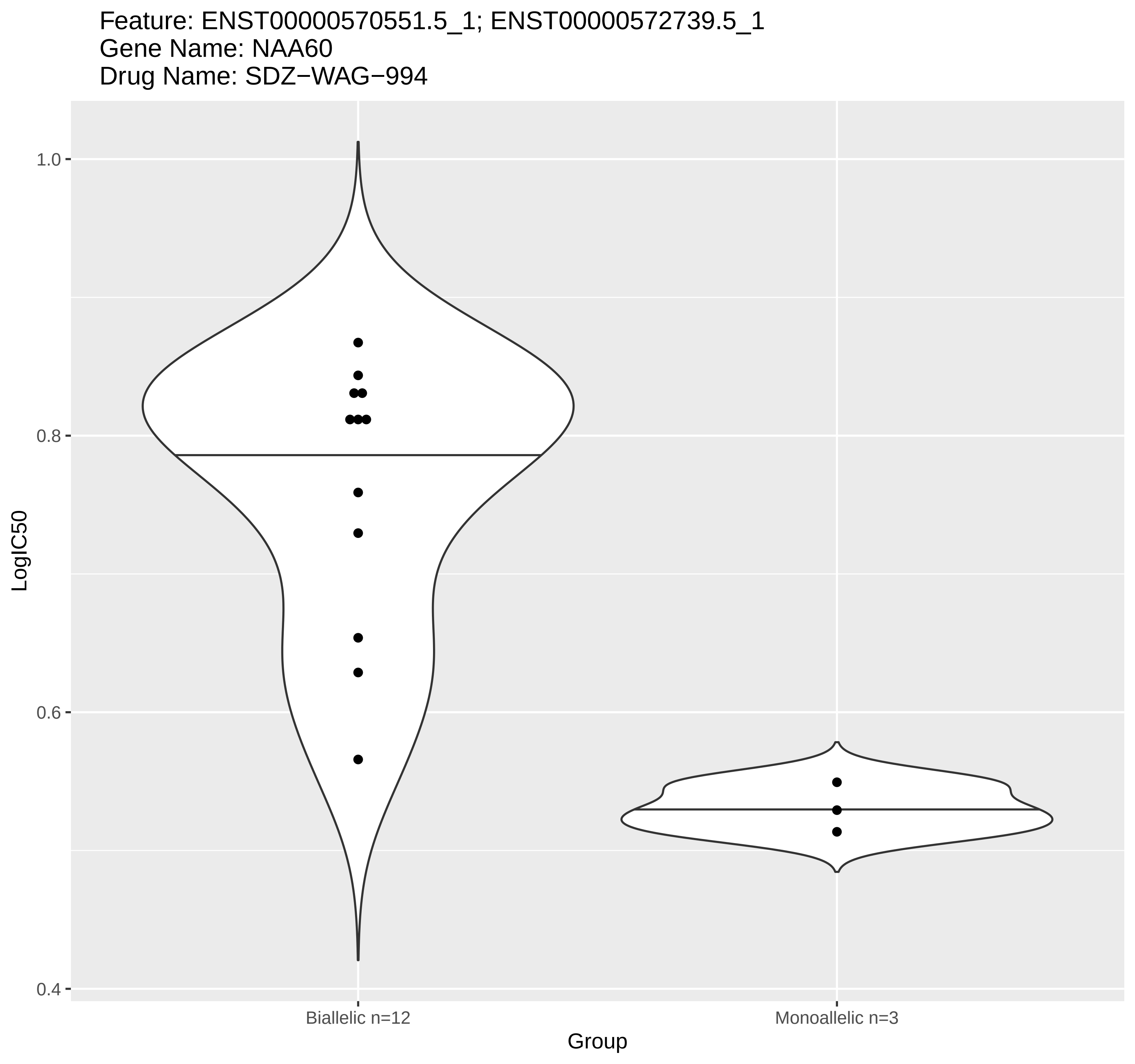

Feature: ENST00000570551.5_1; ENST00000572739.5_1
Gene Name: NAA60
Drug Name: SDZ−WAG−994
LogIC50
1.0
0.8
0.6
0.4
Biallelic n=12
Monoallelic n=3
Group

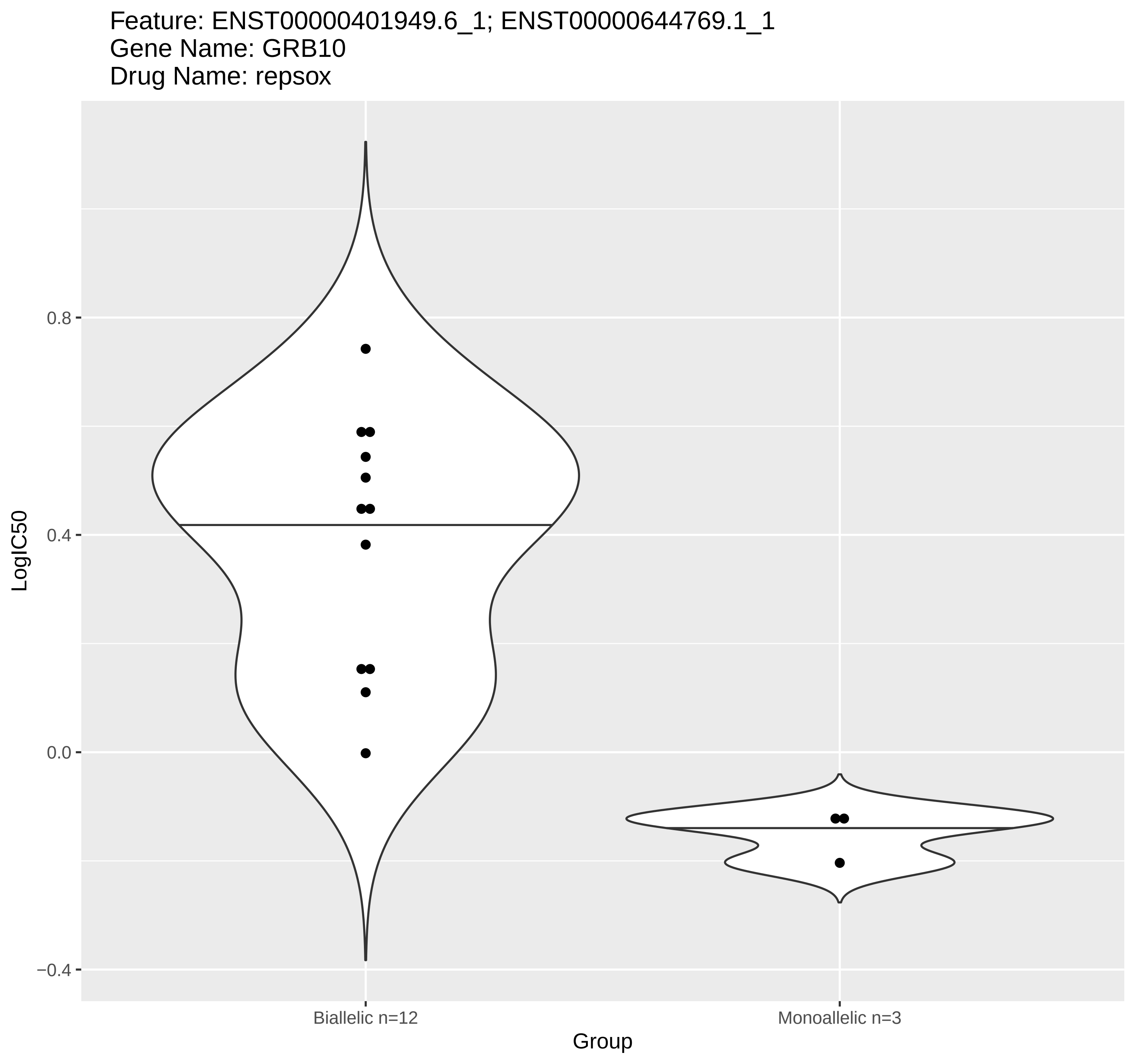
Feature: ENST00000401949.6_1; ENST00000644769.1_1
Gene Name: GRB10
Drug Name: repsox
LogIC50
0.8
0.4
0.0
−0.4
Biallelic n=12
Monoallelic n=3
Group

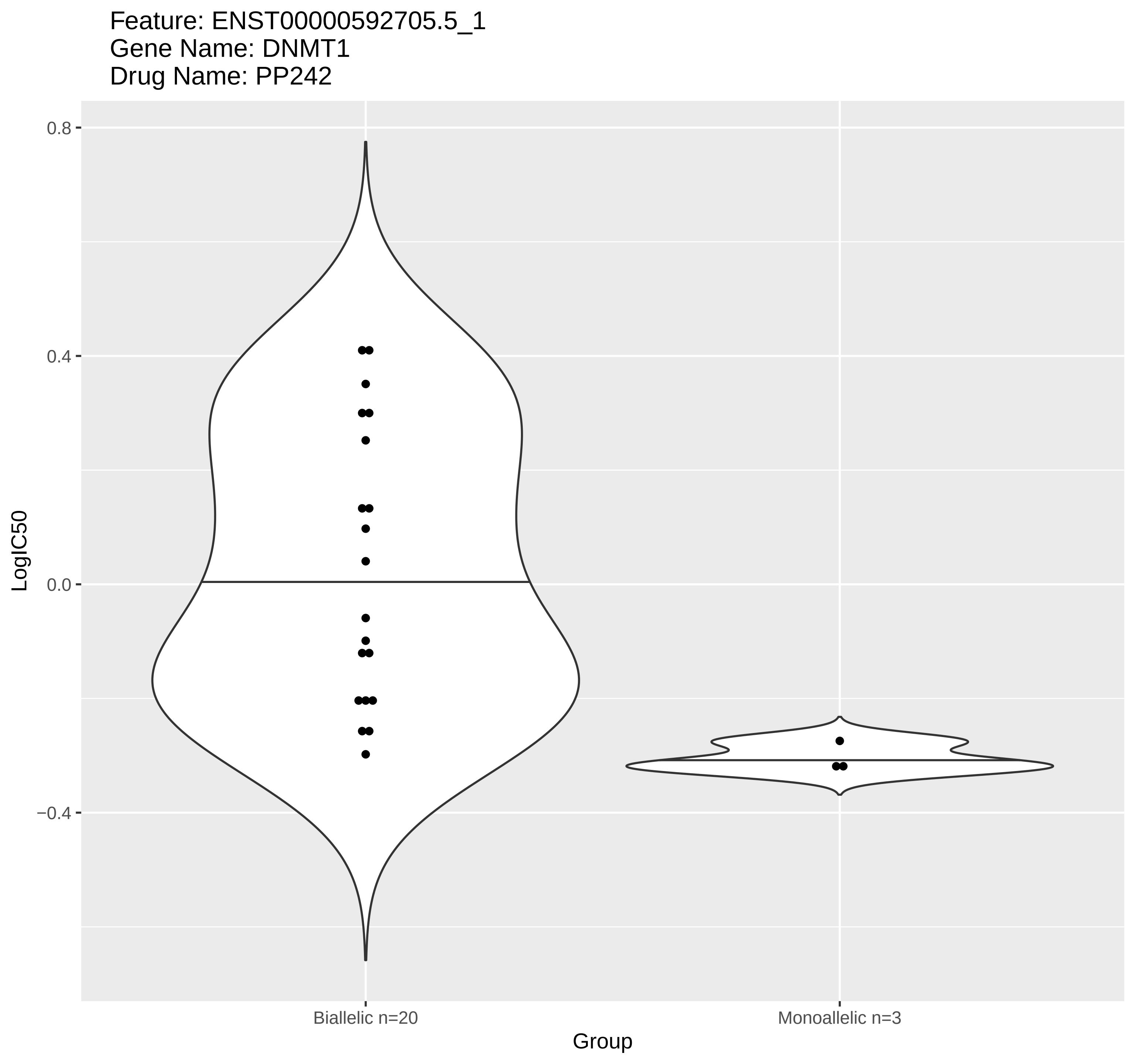
Feature: ENST00000592705.5_1
Gene Name: DNMT1
Drug Name: PP242
0.8
0.4
0.0
−0.4
LogIC50
Biallelic n=20
Monoallelic n=3
Group

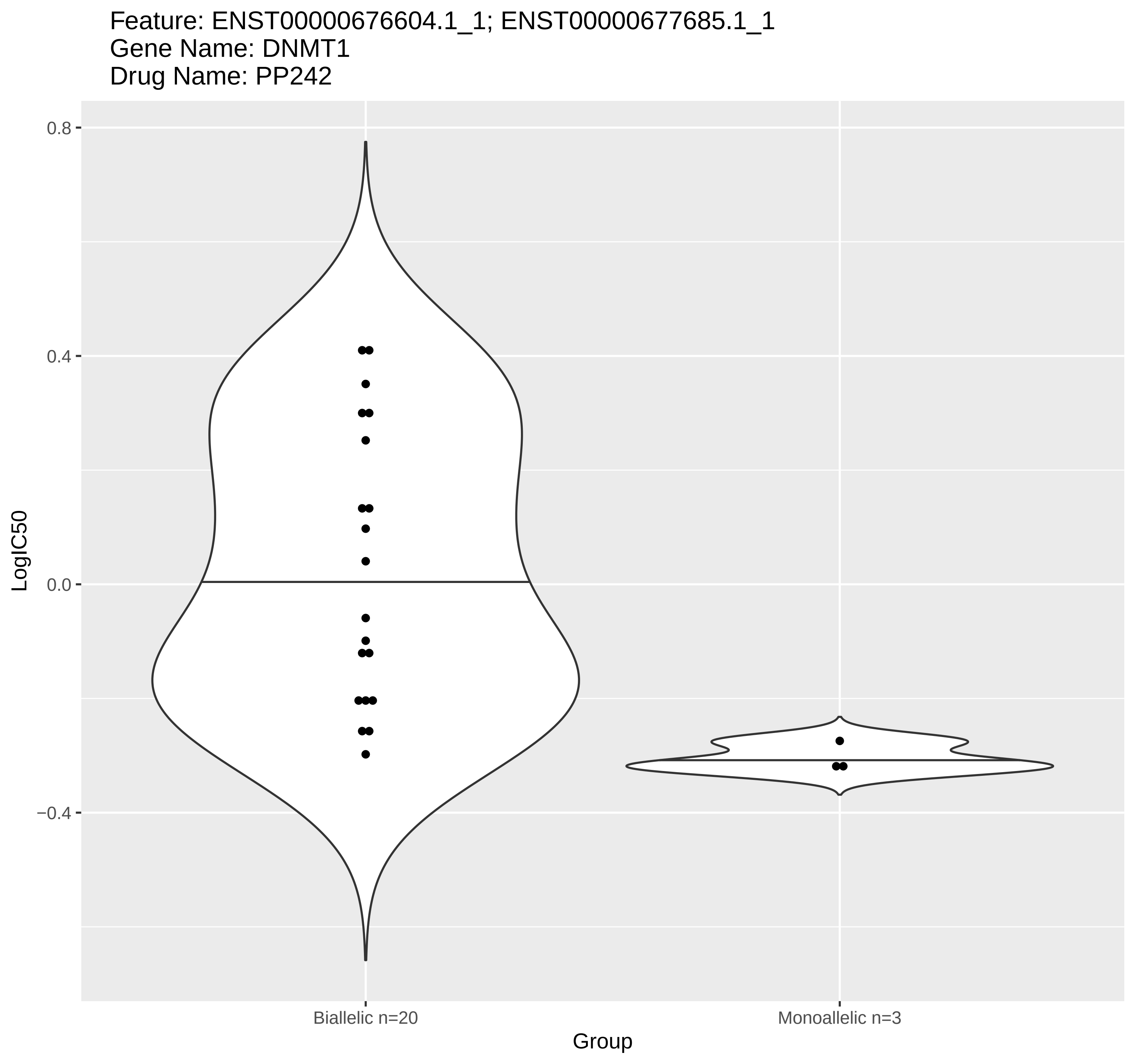
Feature: ENST00000676604.1_1; ENST00000677685.1_1
Gene Name: DNMT1
Drug Name: PP242
0.8
0.4
0.0
−0.4
LogIC50
Biallelic n=20
Monoallelic n=3
Group

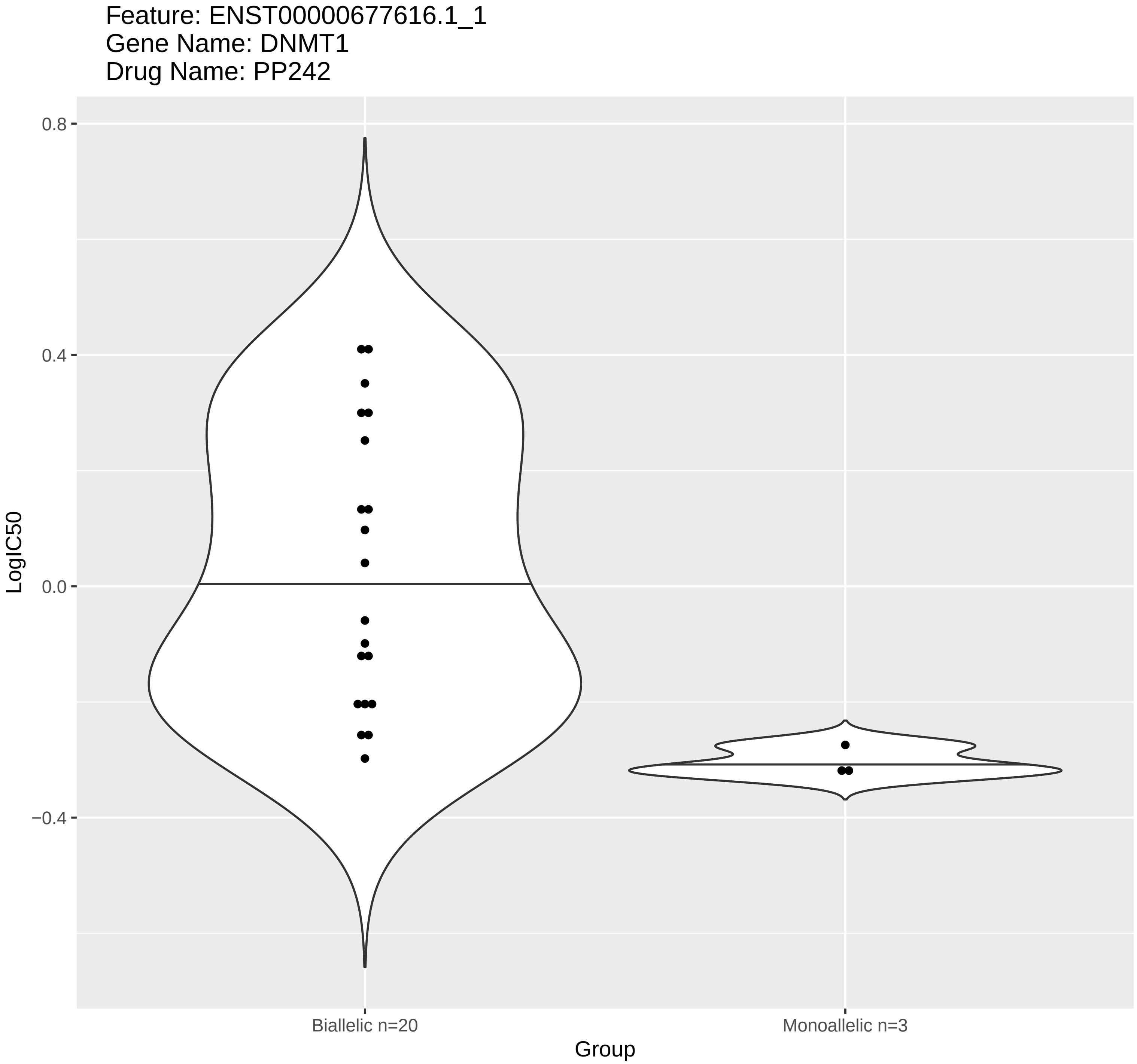
Feature: ENST00000677616.1_1
Gene Name: DNMT1
Drug Name: PP242
0.8
0.4
0.0
−0.4
LogIC50
Biallelic n=20
Monoallelic n=3
Group

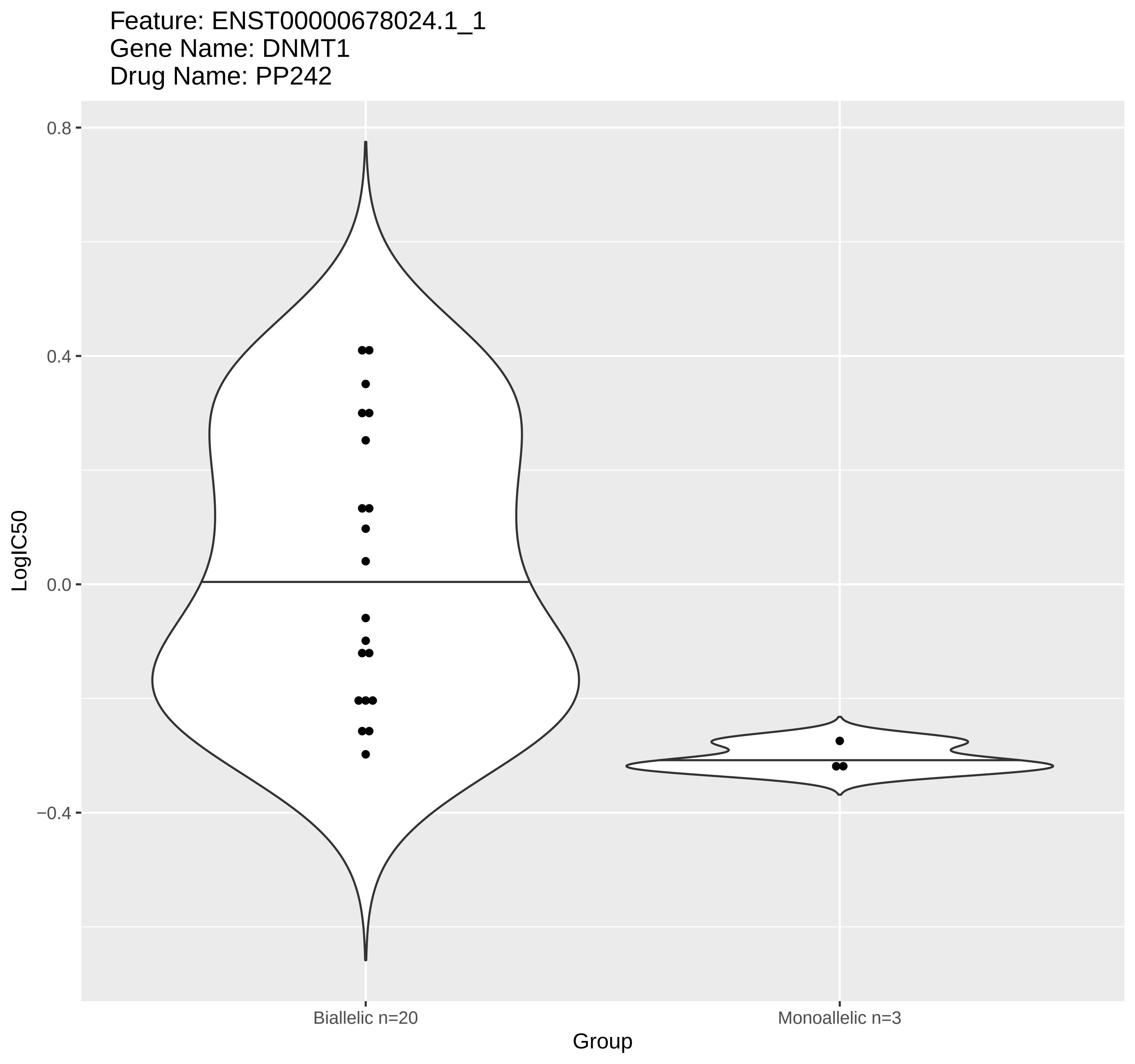
Feature: ENST00000678024.1_1
Gene Name: DNMT1
Drug Name: PP242
LogIC50
0.8
0.4
0.0
−0.4
Biallelic n=20
Monoallelic n=3
Group

Feature: ENST00000678694.1_1
Gene Name: DNMT1
Drug Name: PP242

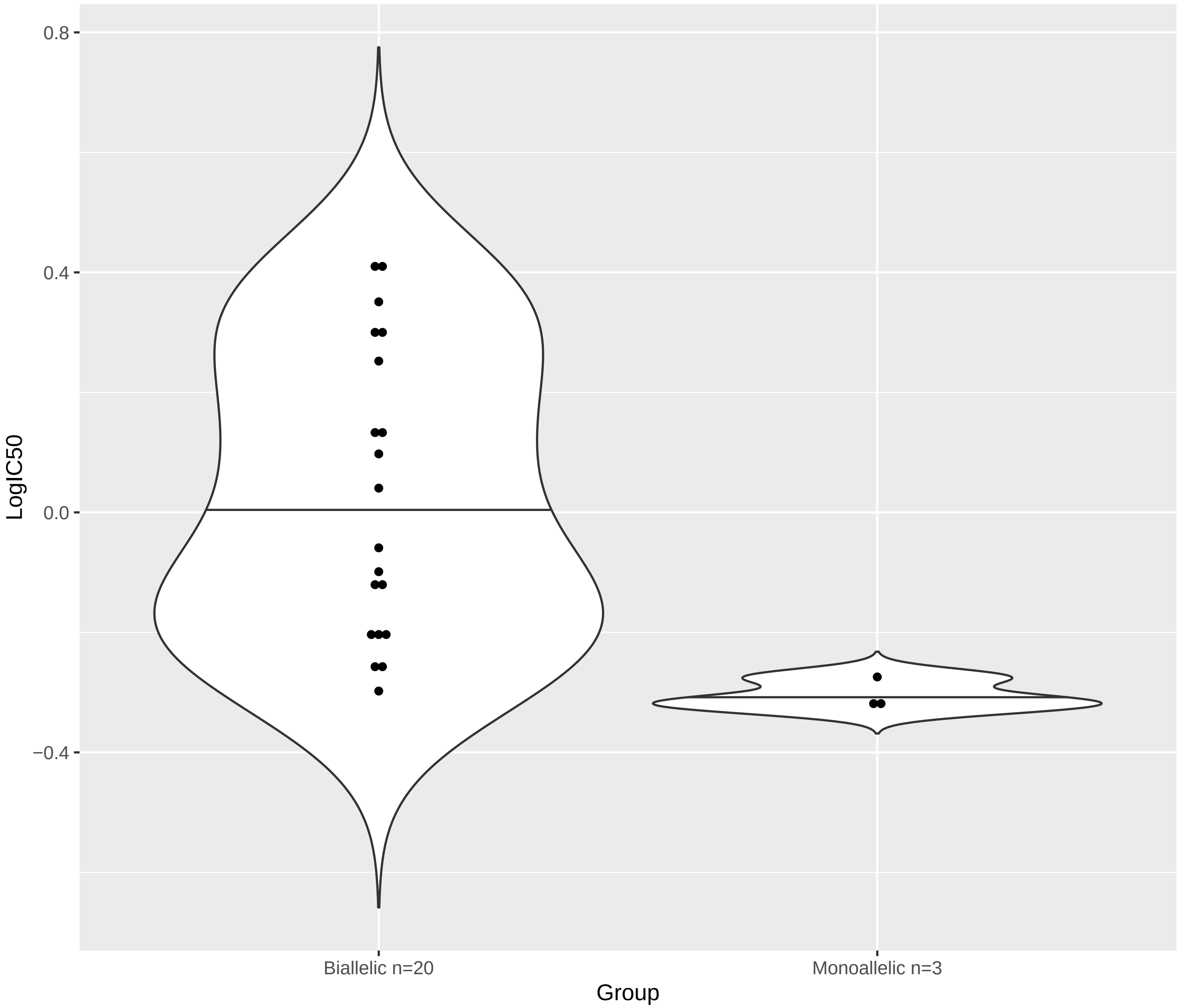

Feature: ENST00000349036.9_1; ENST00000371100.9_1; ENST00000371102.8_1; ENST00000464624.7_1; ENST00000676826.2_1
Gene Name: GNAS
Drug Name: NSC−207895

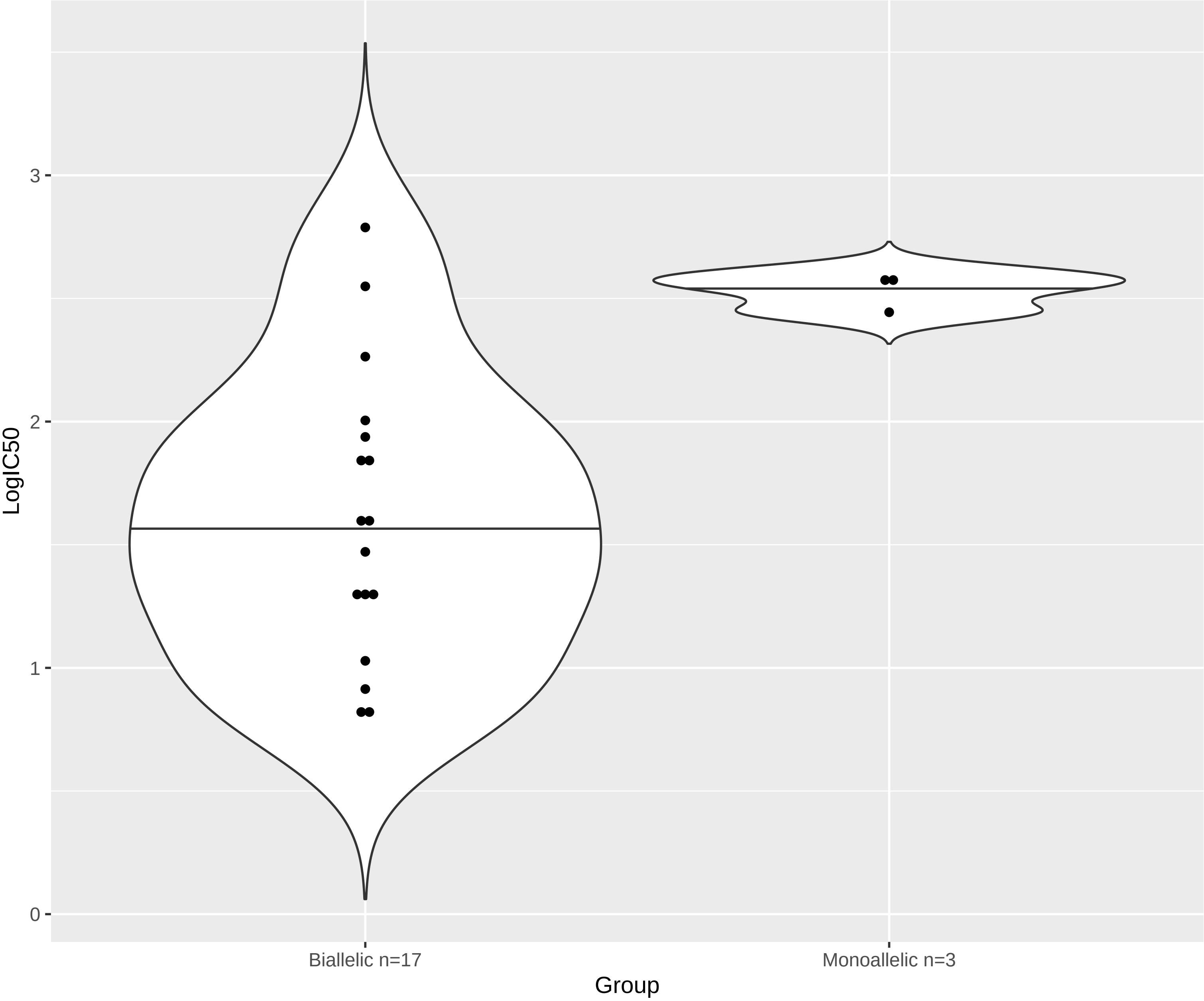

Feature: ENST00000348039.9_1
Gene Name: OSBPL5
Drug Name: N22899−6−C1

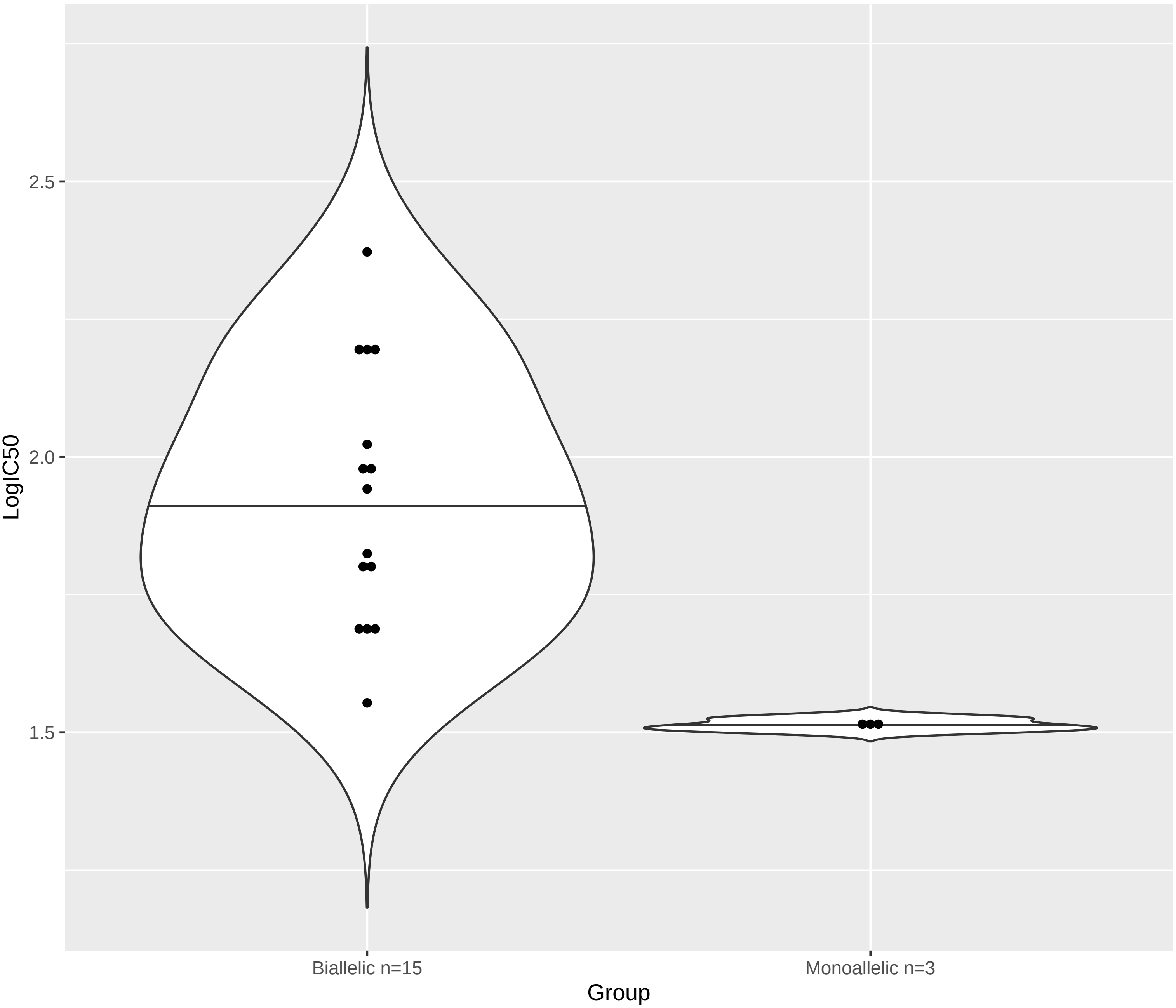

Feature: ENST00000475610.2_1
Gene Name: GNAS
Drug Name: 50869

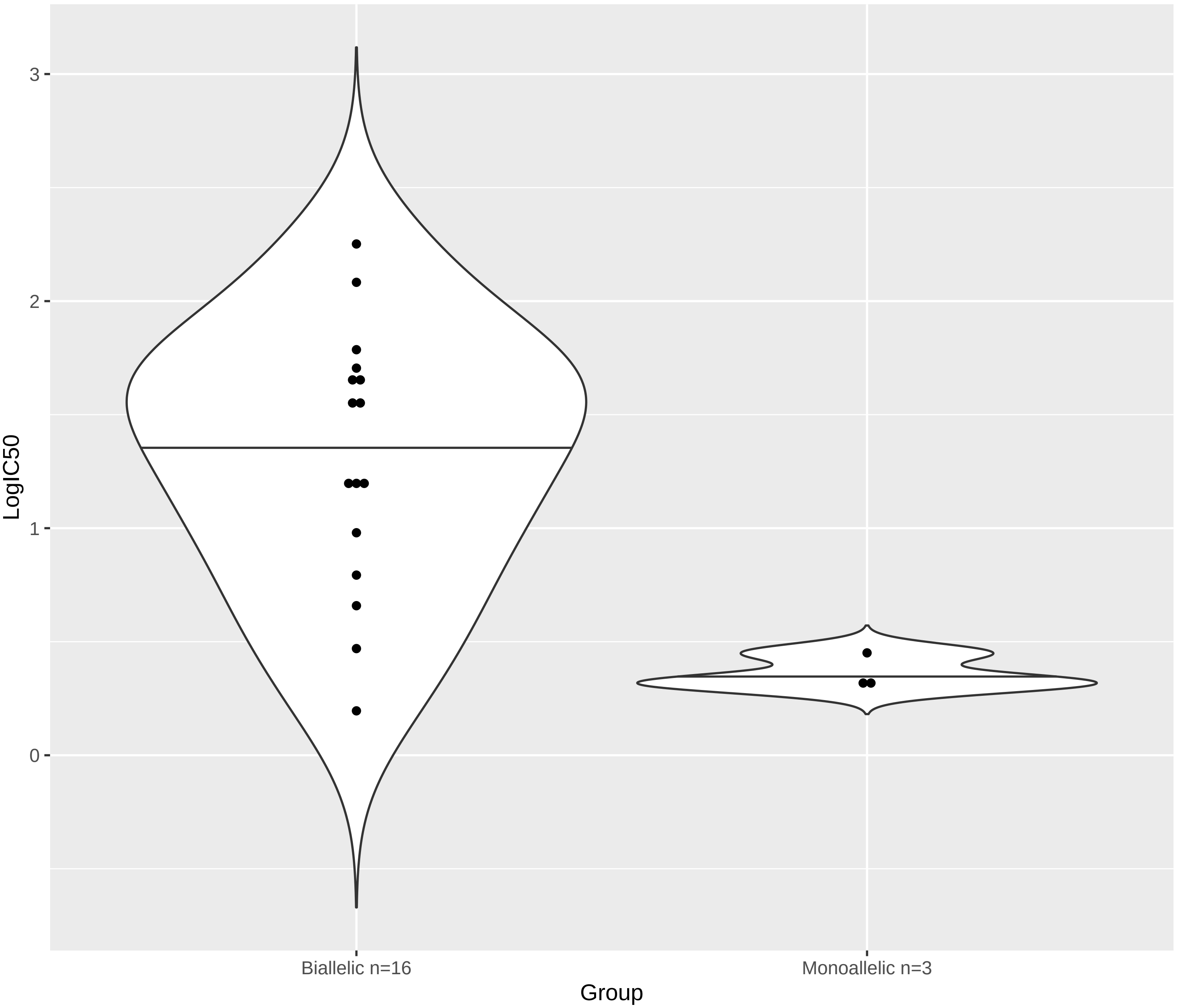

Feature: ENST00000476196.5_1; ENST00000682917.1_1; ENST00000684761.1_1
Gene Name: GNAS
Drug Name: 50869

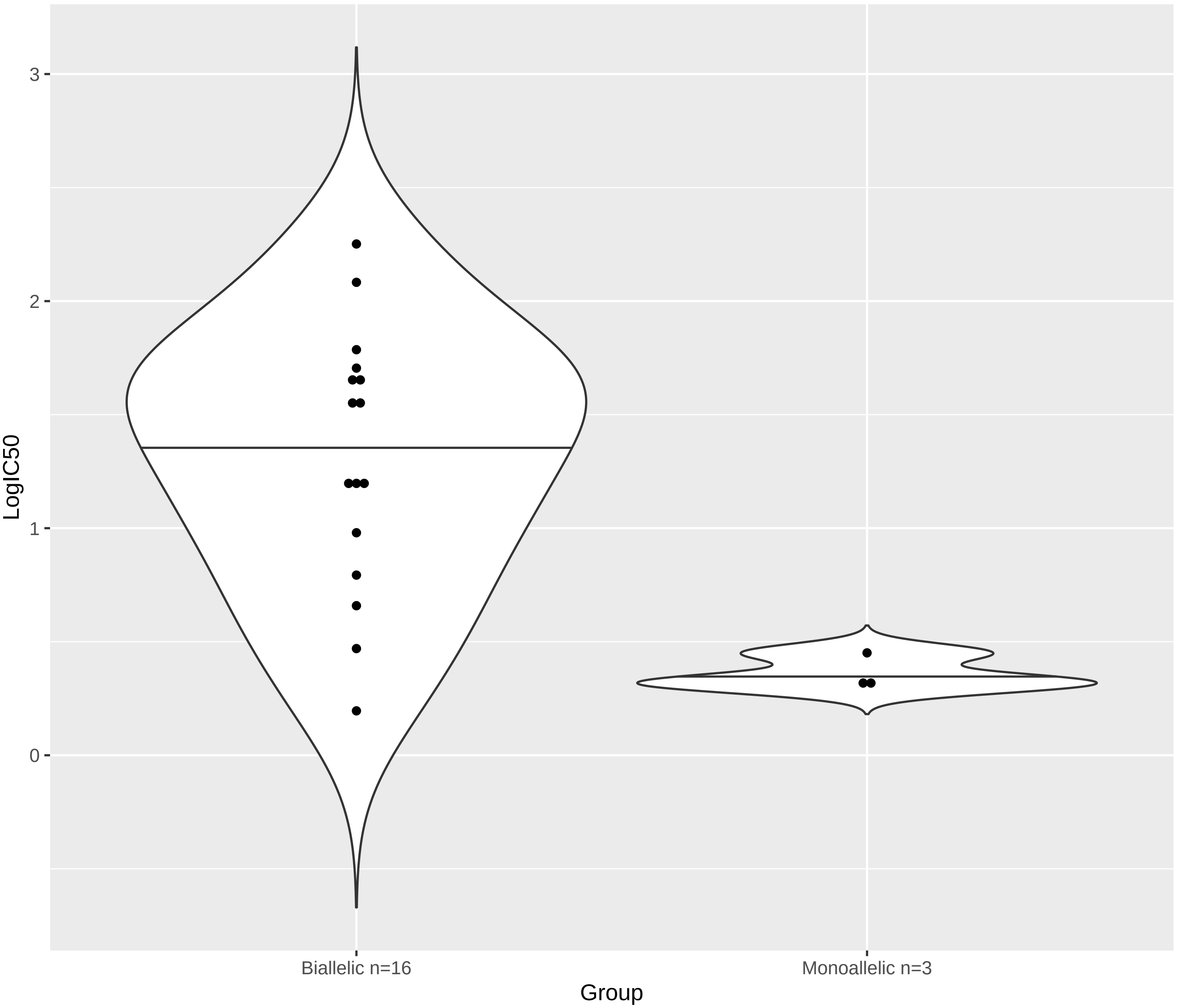

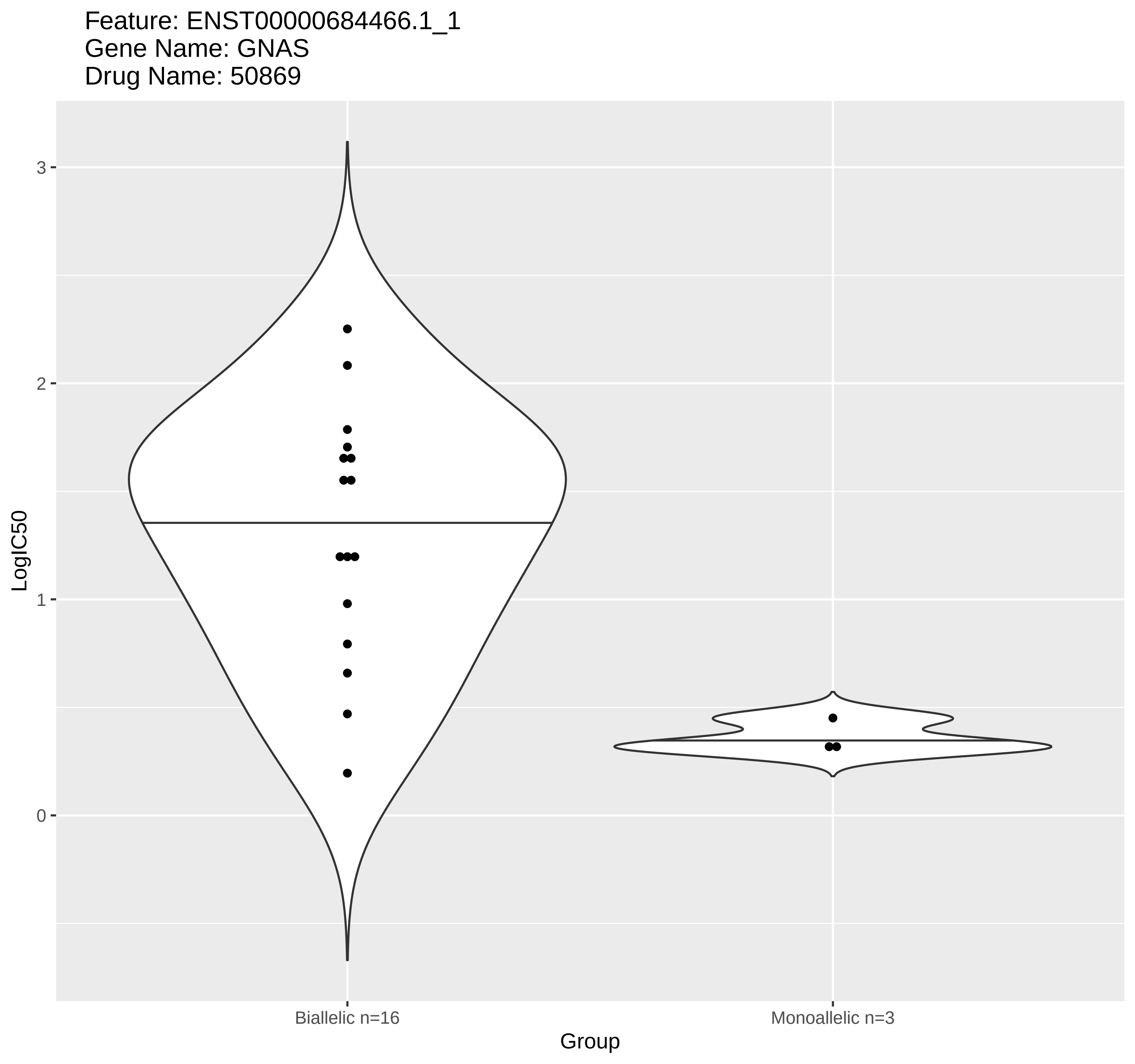
Feature: ENST00000684466.1_1
Gene Name: GNAS
Drug Name: 50869
LogIC50
3
2
1
0
Biallelic n=16
Monoallelic n=3
Group

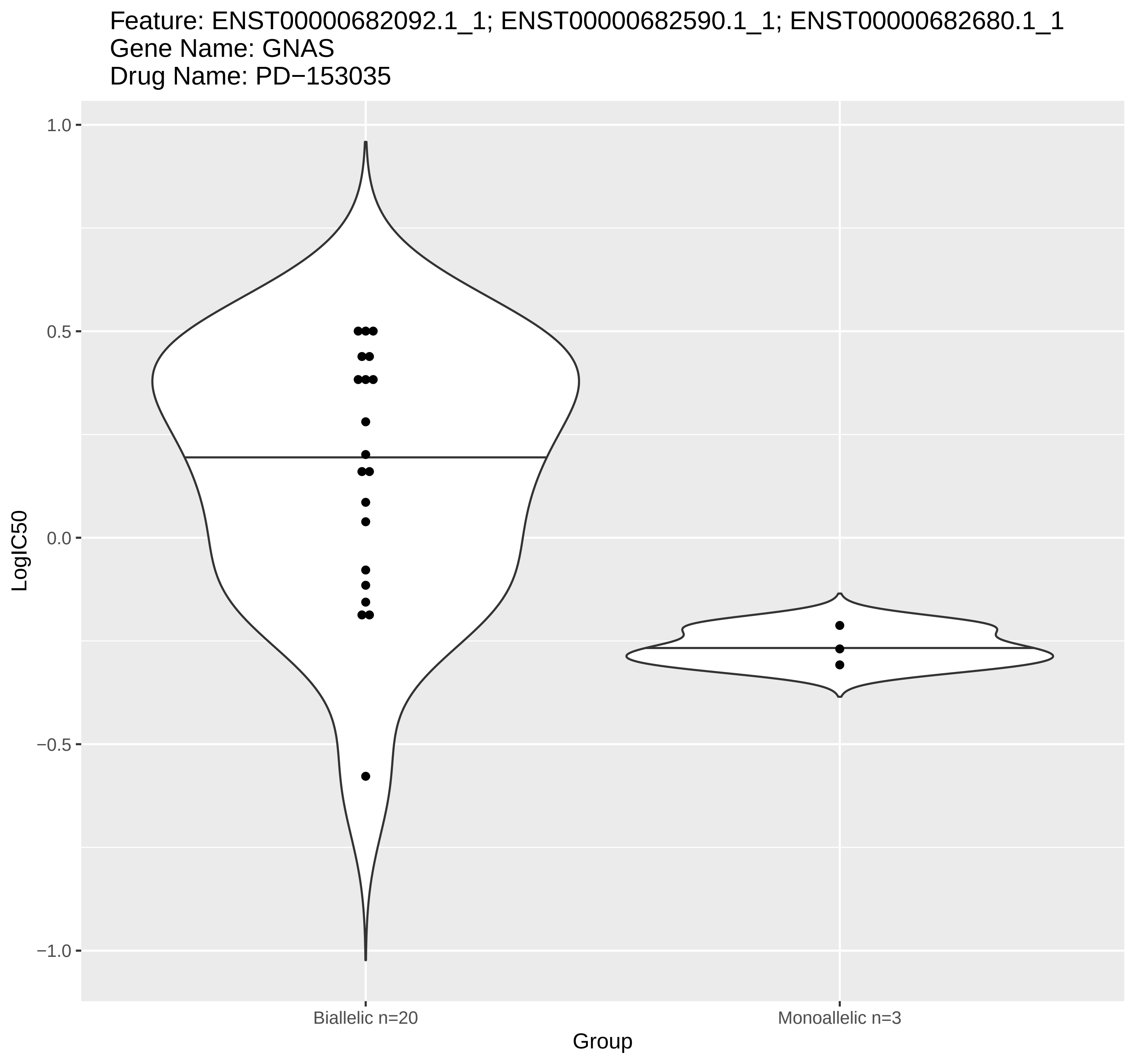

Feature: ENST00000682092.1_1; ENST00000682590.1_1; ENST00000682680.1_1
Gene Name: GNAS
Drug Name: PD−153035
1.0
0.5
0.0
−0.5
−1.0
LogIC50
Biallelic n=20
Monoallelic n=3
Group

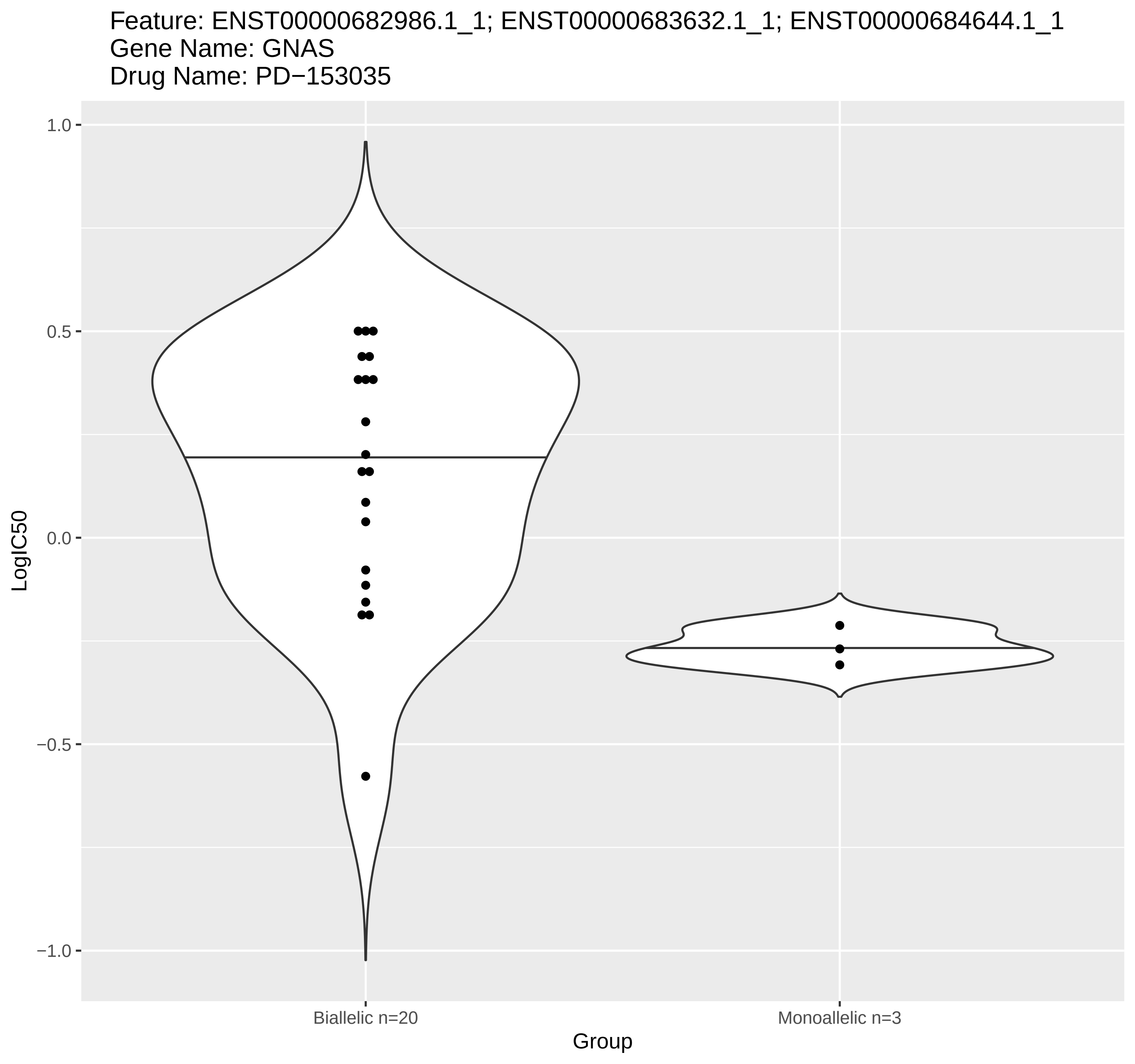

Feature: ENST00000682986.1_1; ENST00000683632.1_1; ENST00000684644.1_1
Gene Name: GNAS
Drug Name: PD−153035
1.0
0.5
0.0
−0.5
−1.0
LogIC50
Biallelic n=20
Monoallelic n=3
Group

Feature: ENST00000525498.5_1
Gene Name: OSBPL5
Drug Name: N22899−6−C1

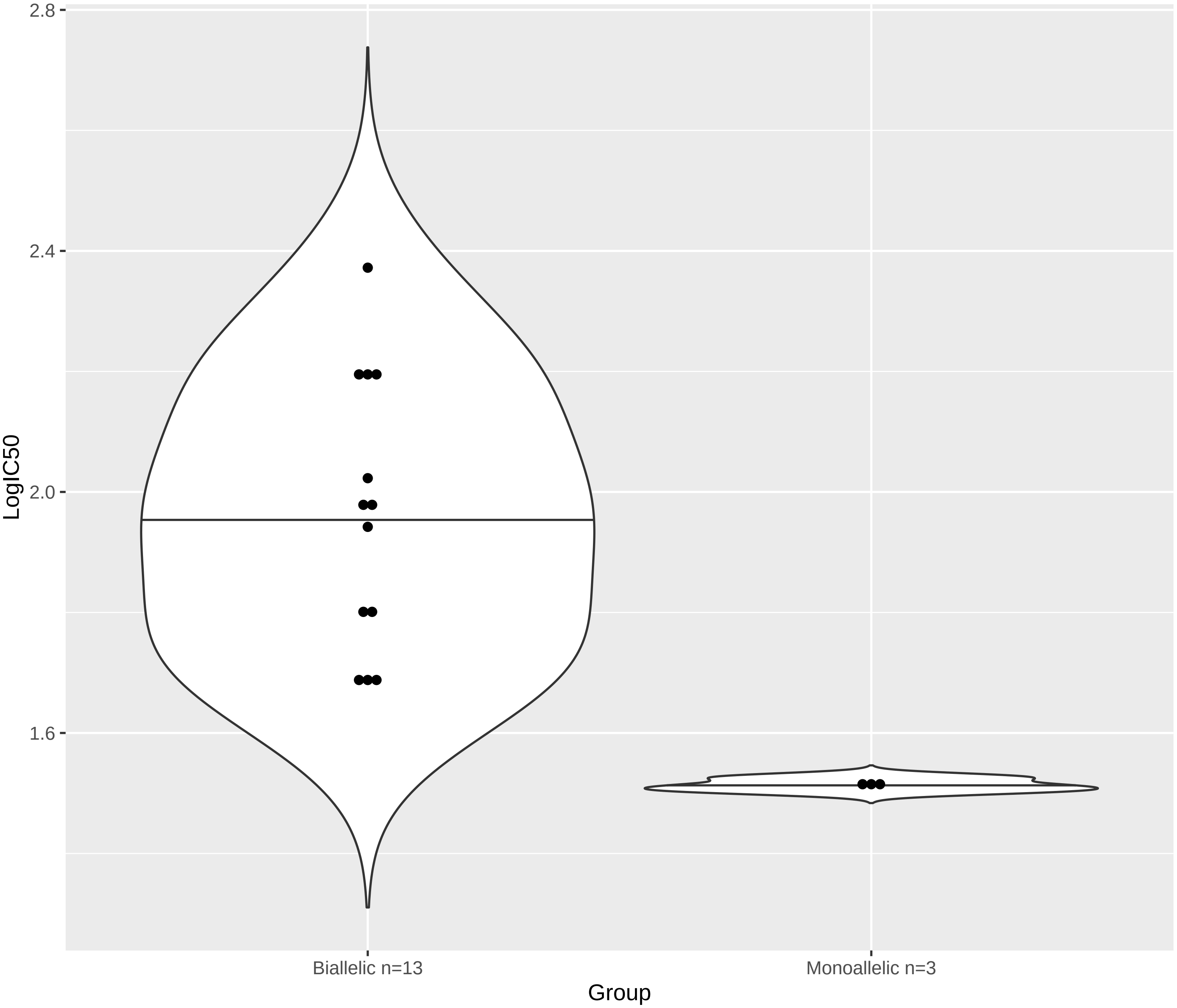

Feature: ENST00000349036.9_1; ENST00000371100.9_1; ENST00000371102.8_1; ENST00000464624.7_1; ENST00000676826.2_1
Gene Name: GNAS
Drug Name: JQ1

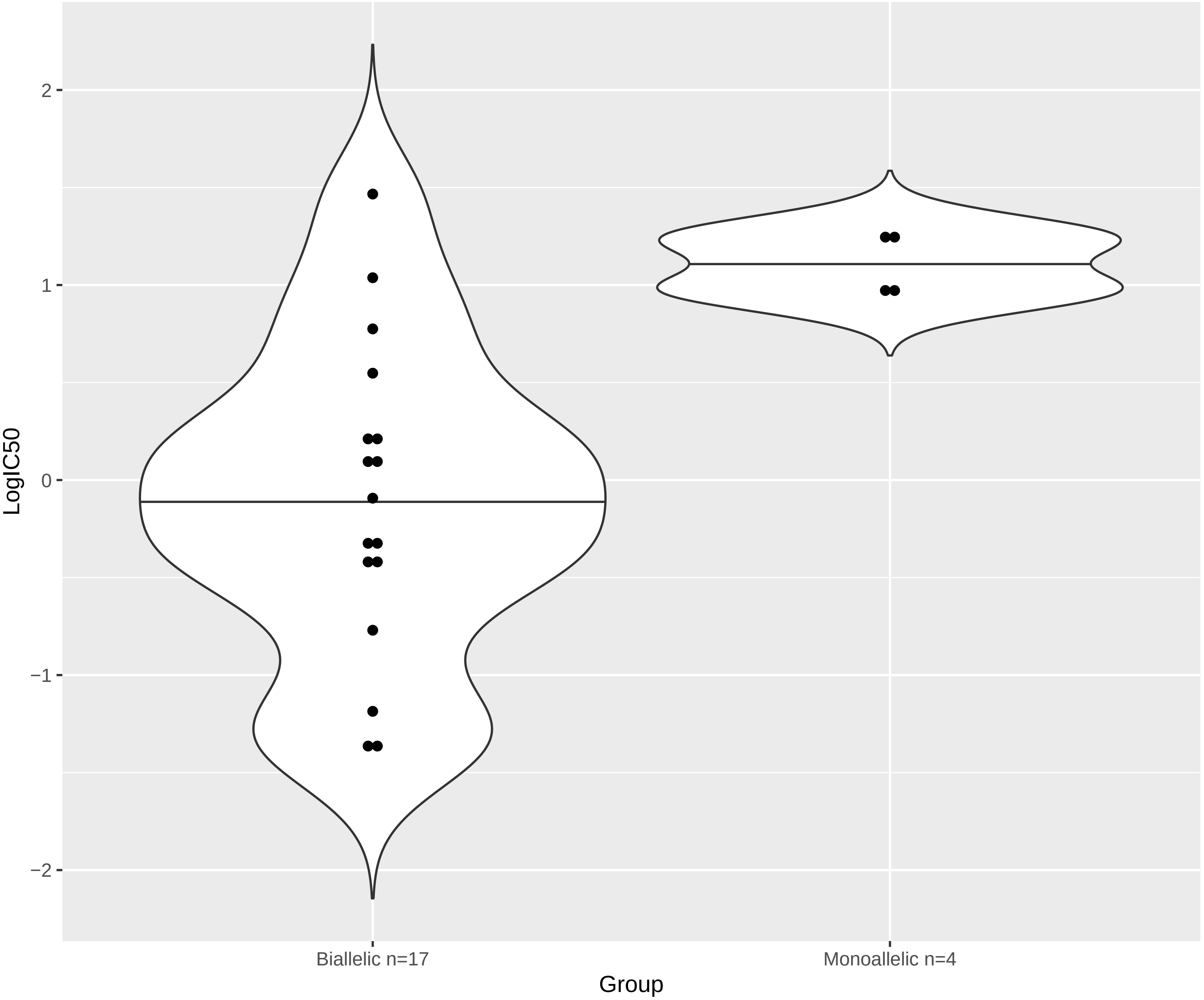

Feature: ENST00000349036.9_1; ENST00000371100.9_1; ENST00000371102.8_1; ENST00000464624.7_1; ENST00000676826.2_1
Gene Name: GNAS
Drug Name: KIN001−270

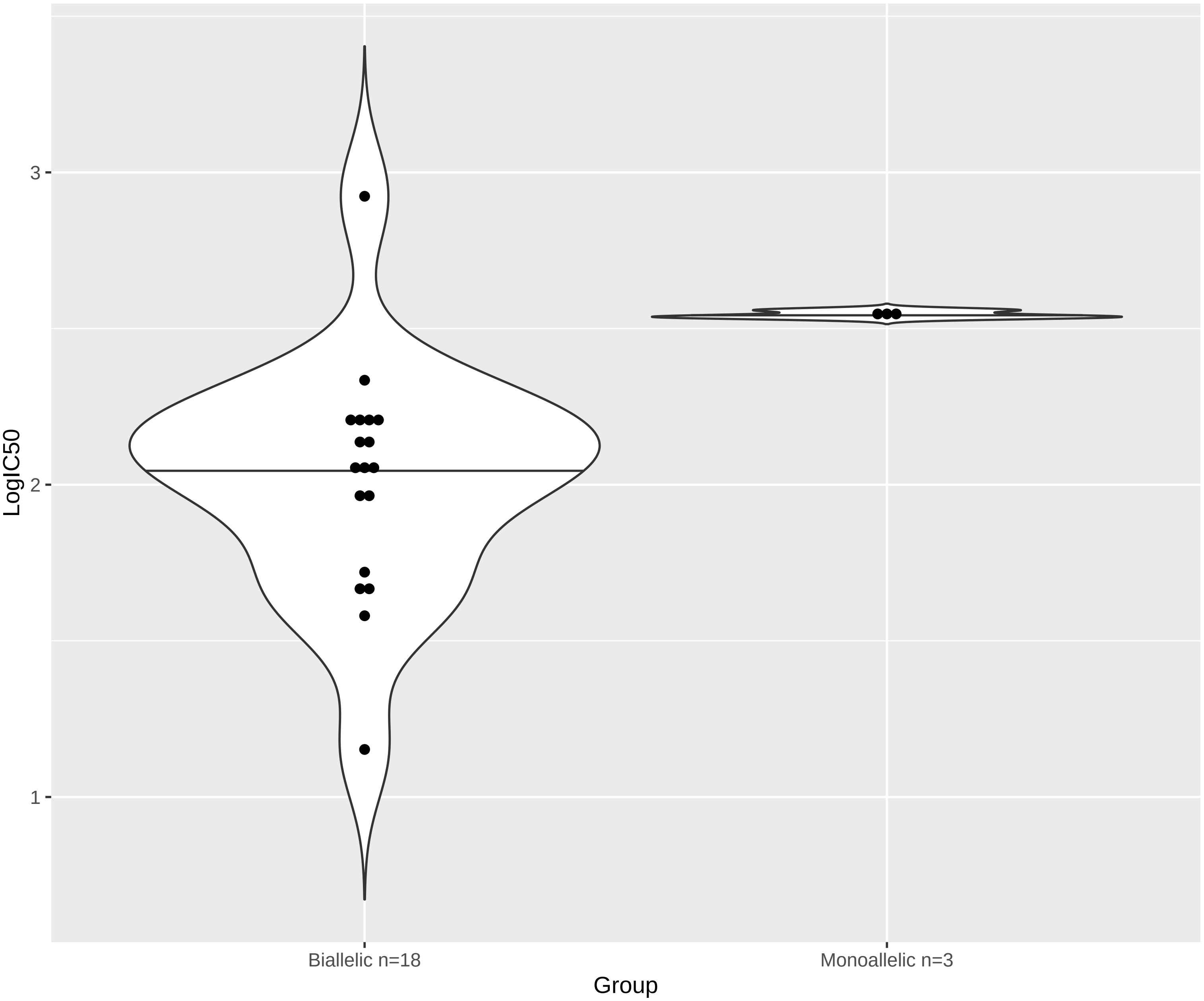

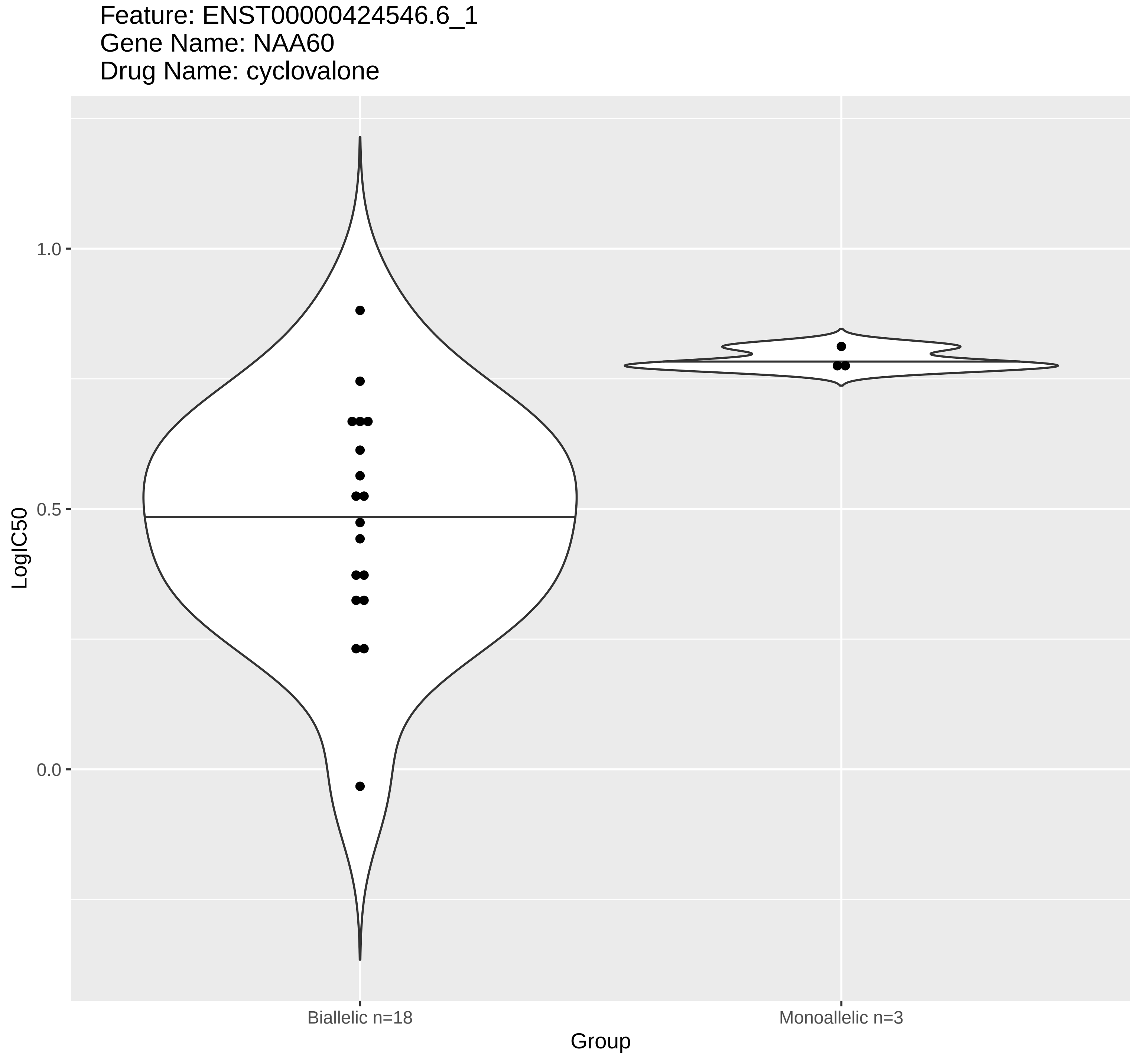
Feature: ENST00000424546.6_1
Gene Name: NAA60
Drug Name: cyclovalone
LogIC50
1.0
0.5
0.0
Biallelic n=18
Monoallelic n=3
Group

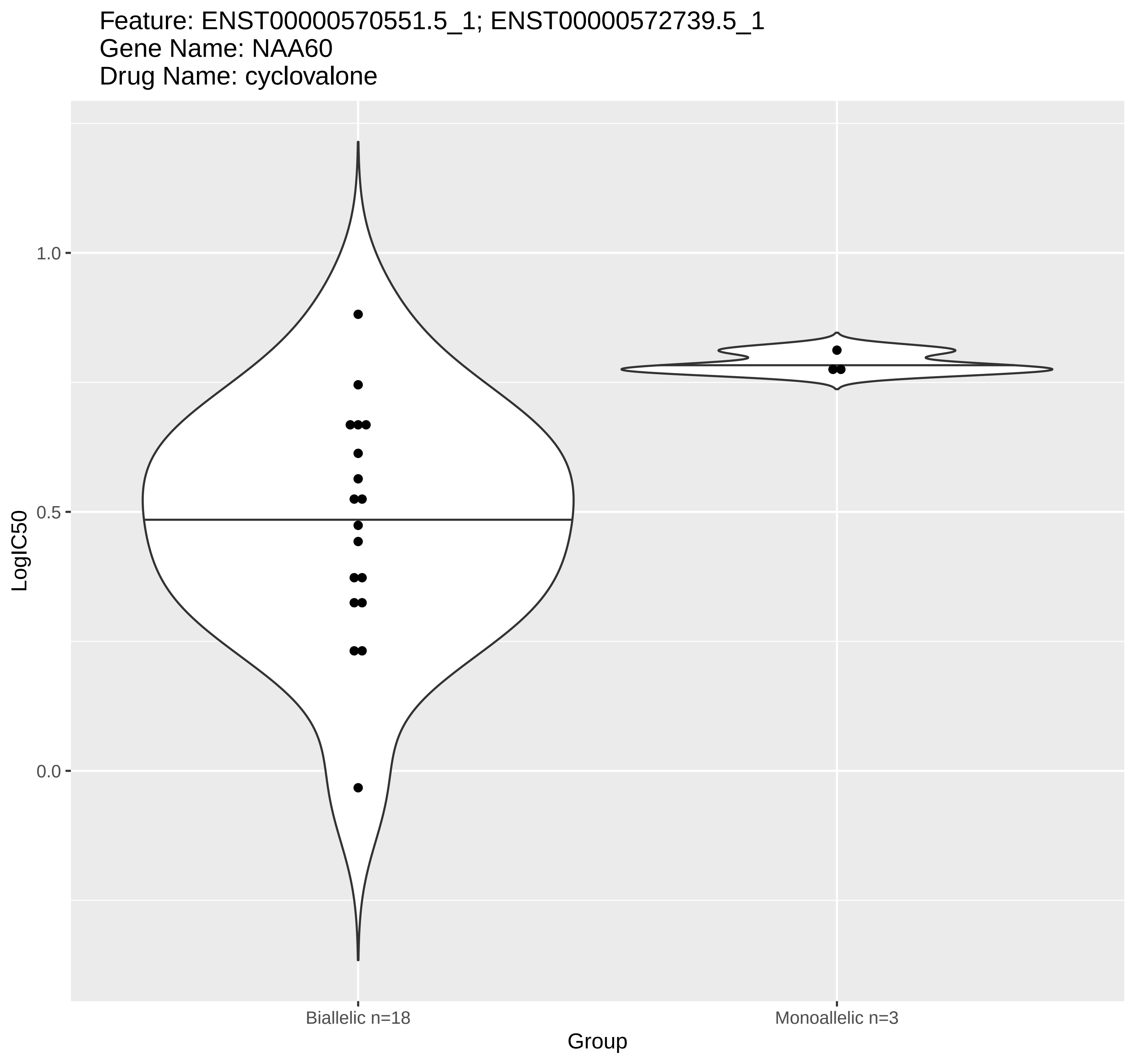
Feature: ENST00000570551.5_1; ENST00000572739.5_1
Gene Name: NAA60
Drug Name: cyclovalone
1.0
0.5
0.0
LogIC50
Biallelic n=18
Monoallelic n=3
Group

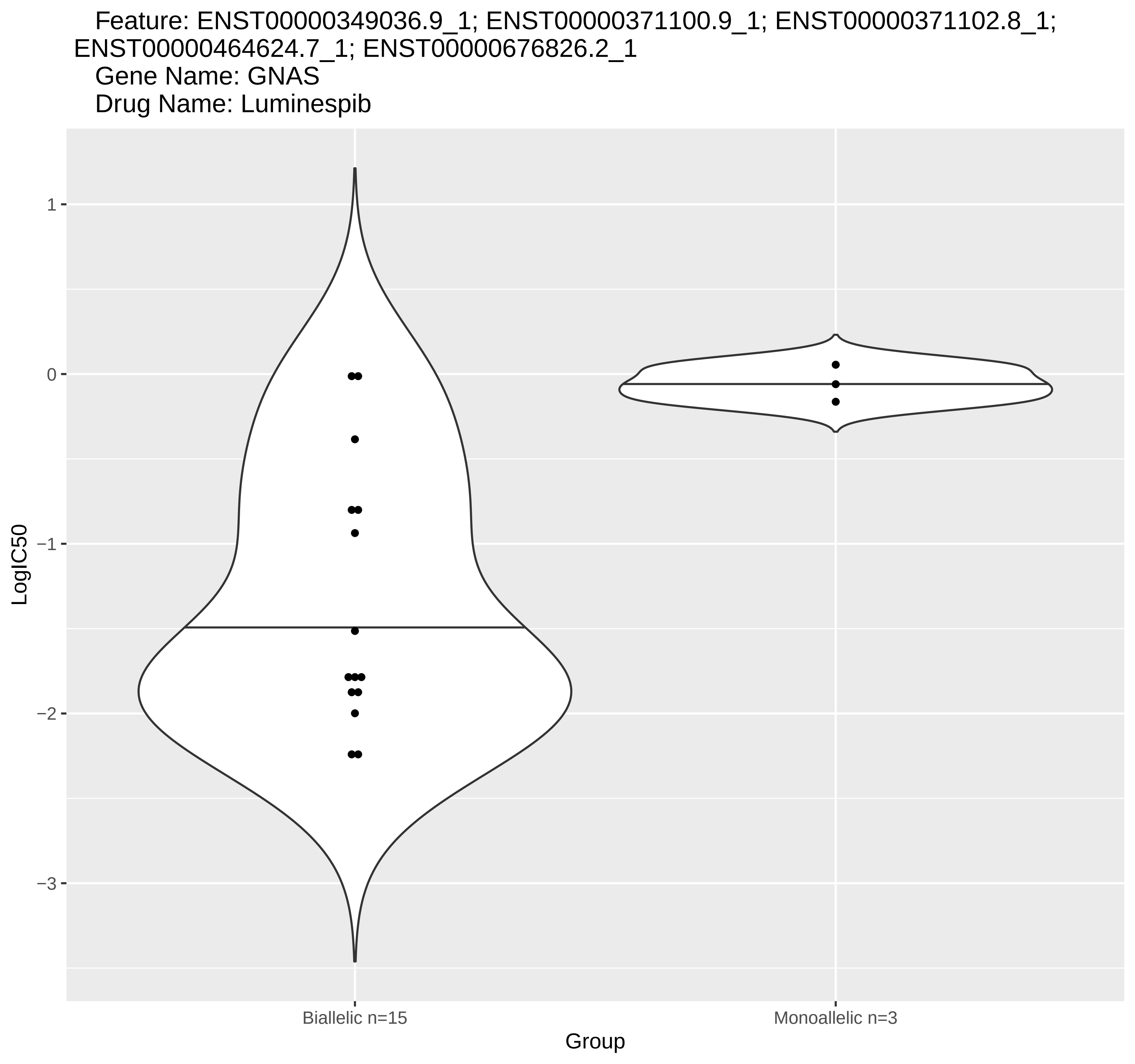

Feature: ENST00000349036.9_1; ENST00000371100.9_1; ENST00000371102.8_1; ENST00000464624.7_1; ENST00000676826.2_1
Gene Name: GNAS
Drug Name: Luminespib
LogIC50
1
0
−1
−2
−3
Biallelic n=15
Monoallelic n=3
Group

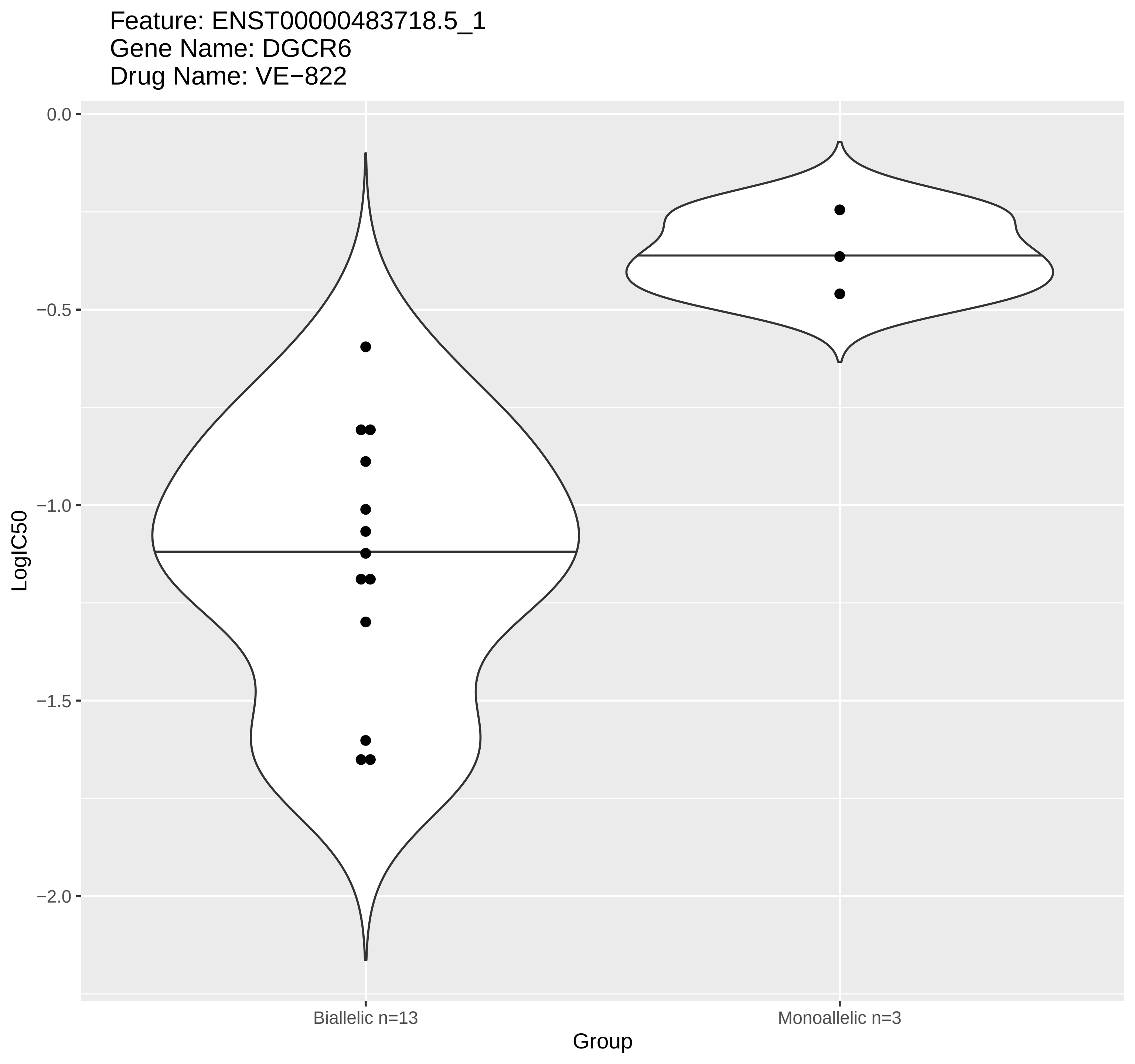

Feature: ENST00000483718.5_1
Gene Name: DGCR6
Drug Name: VE−822
0.0
−0.5
−1.0
−1.5
−2.0
LogIC50
Biallelic n=13
Monoallelic n=3
Group

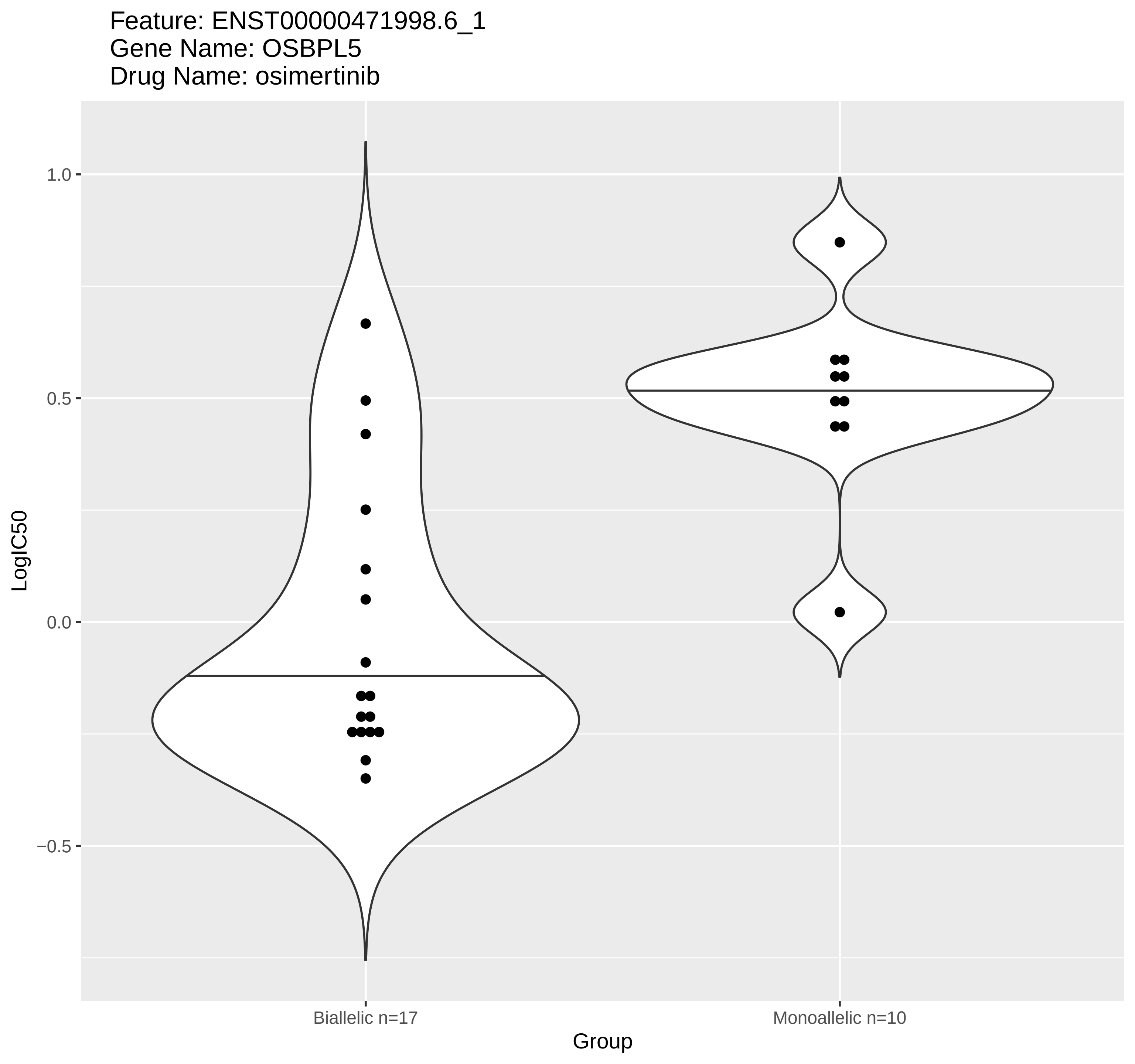

Feature: ENST00000471998.6_1
Gene Name: OSBPL5
Drug Name: osimertinib
1.0
0.5
0.0
−0.5
LogIC50
Biallelic n=17
Monoallelic n=10
Group

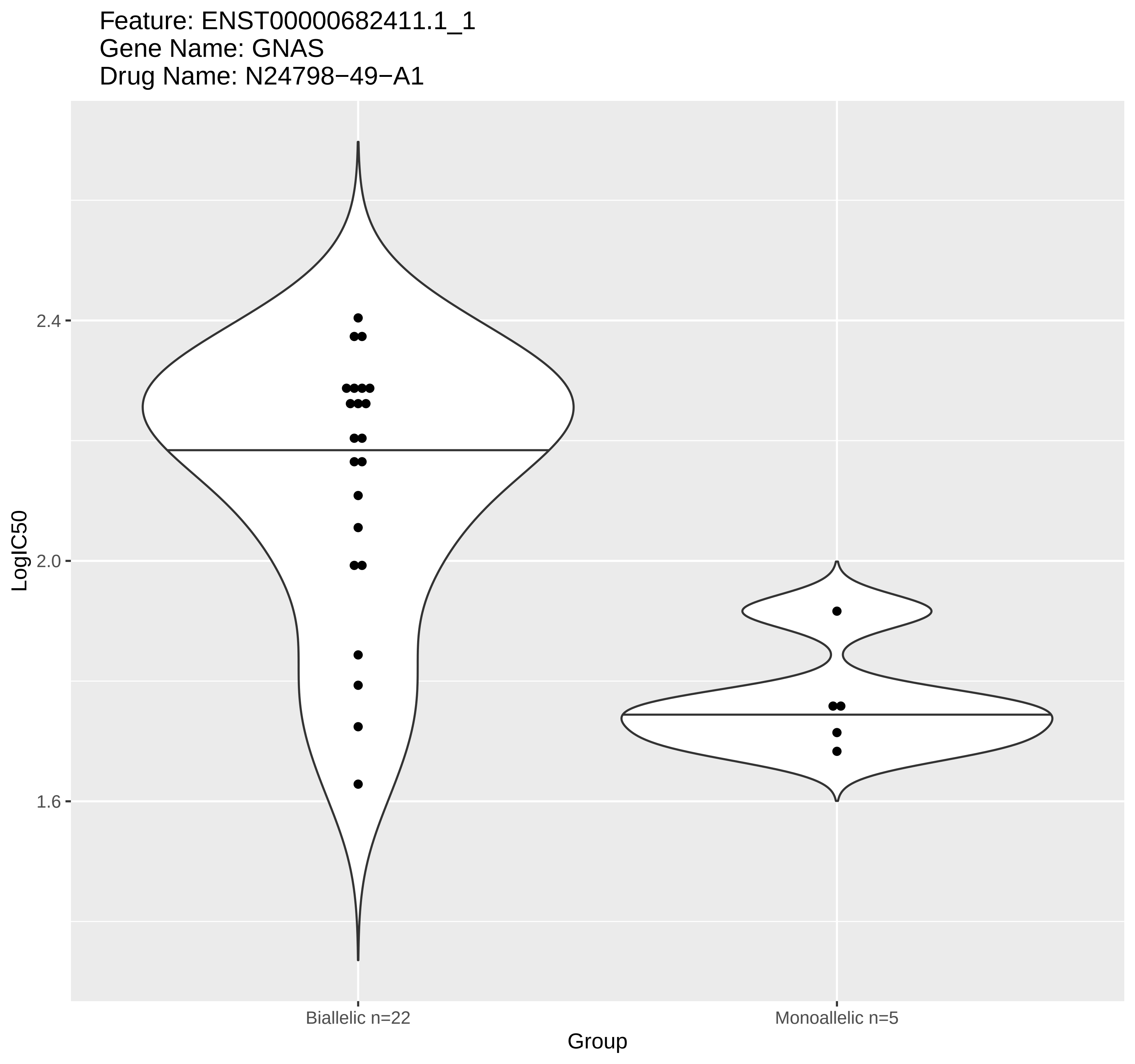
Feature: ENST00000682411.1_1
Gene Name: GNAS
Drug Name: N24798−49−A1
LogIC50
2.4
2.0
1.6
Biallelic n=22
Monoallelic n=5
Group

Feature: ENST00000676868.1_1
Gene Name: DNMT1
Drug Name: AZD2014

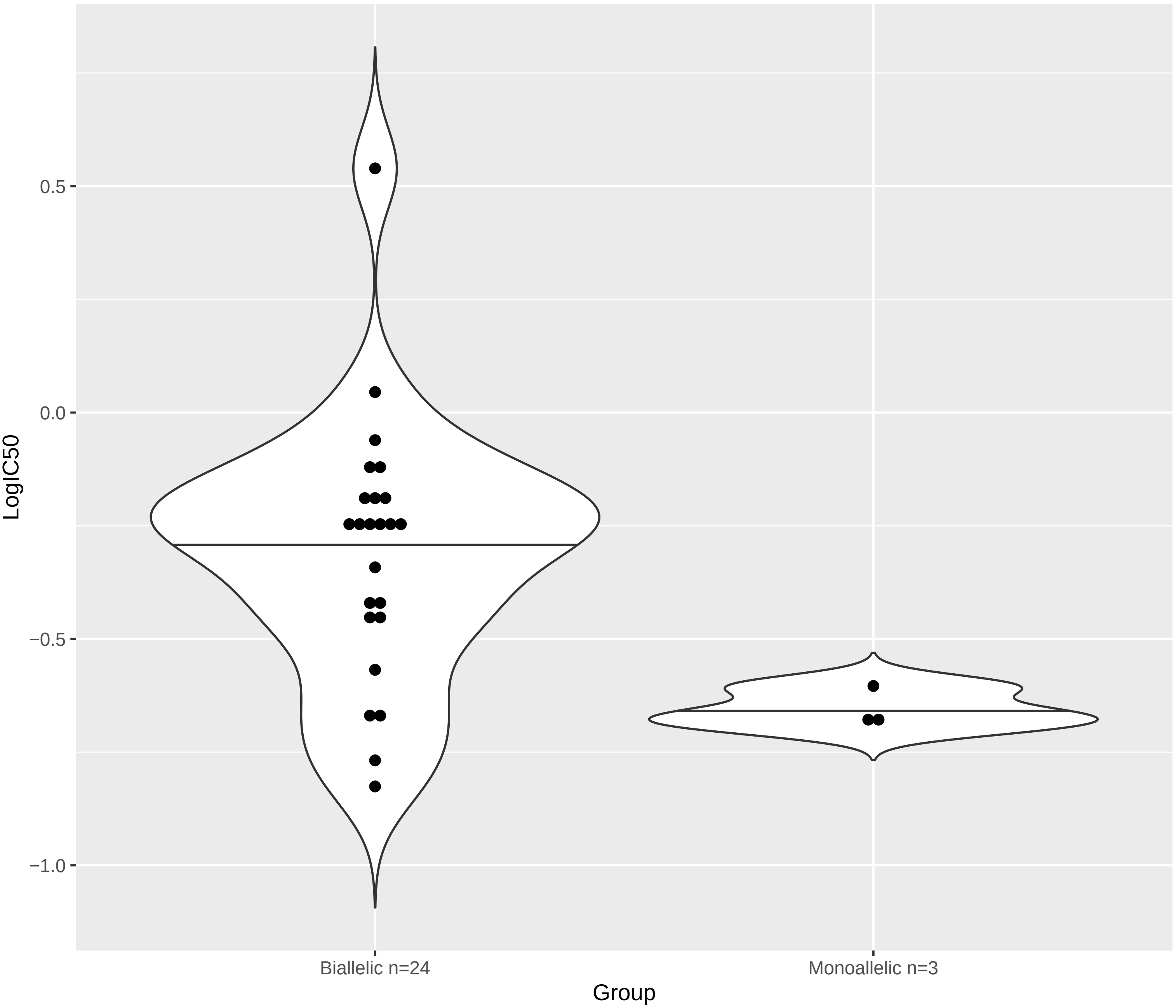

Feature: ENST00000677783.1_1
Gene Name: DNMT1
Drug Name: AZD2014

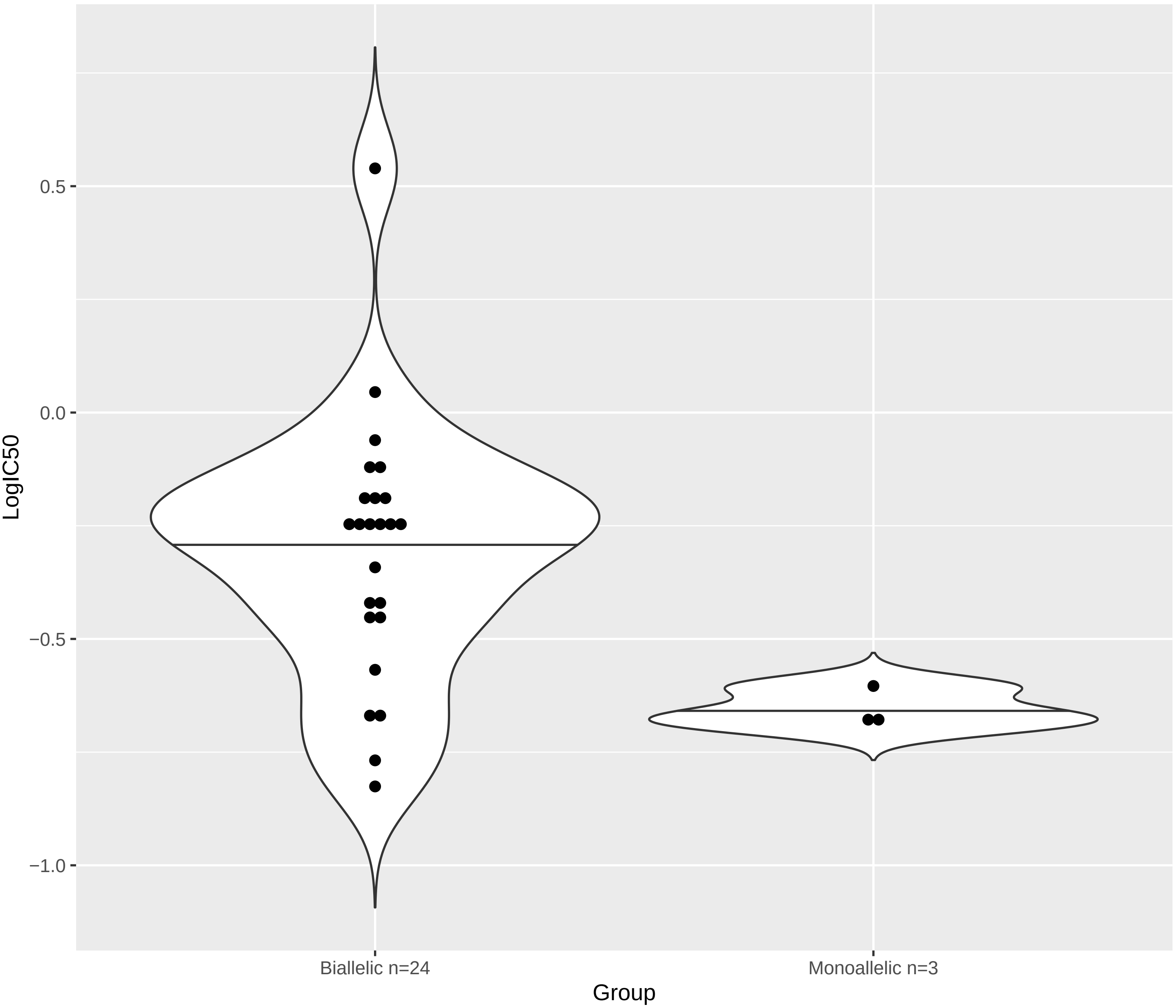

Feature: ENST00000349036.9_1; ENST00000371100.9_1; ENST00000371102.8_1; ENST00000464624.7_1; ENST00000676826.2_1
Gene Name: GNAS
Drug Name: SNX−2112

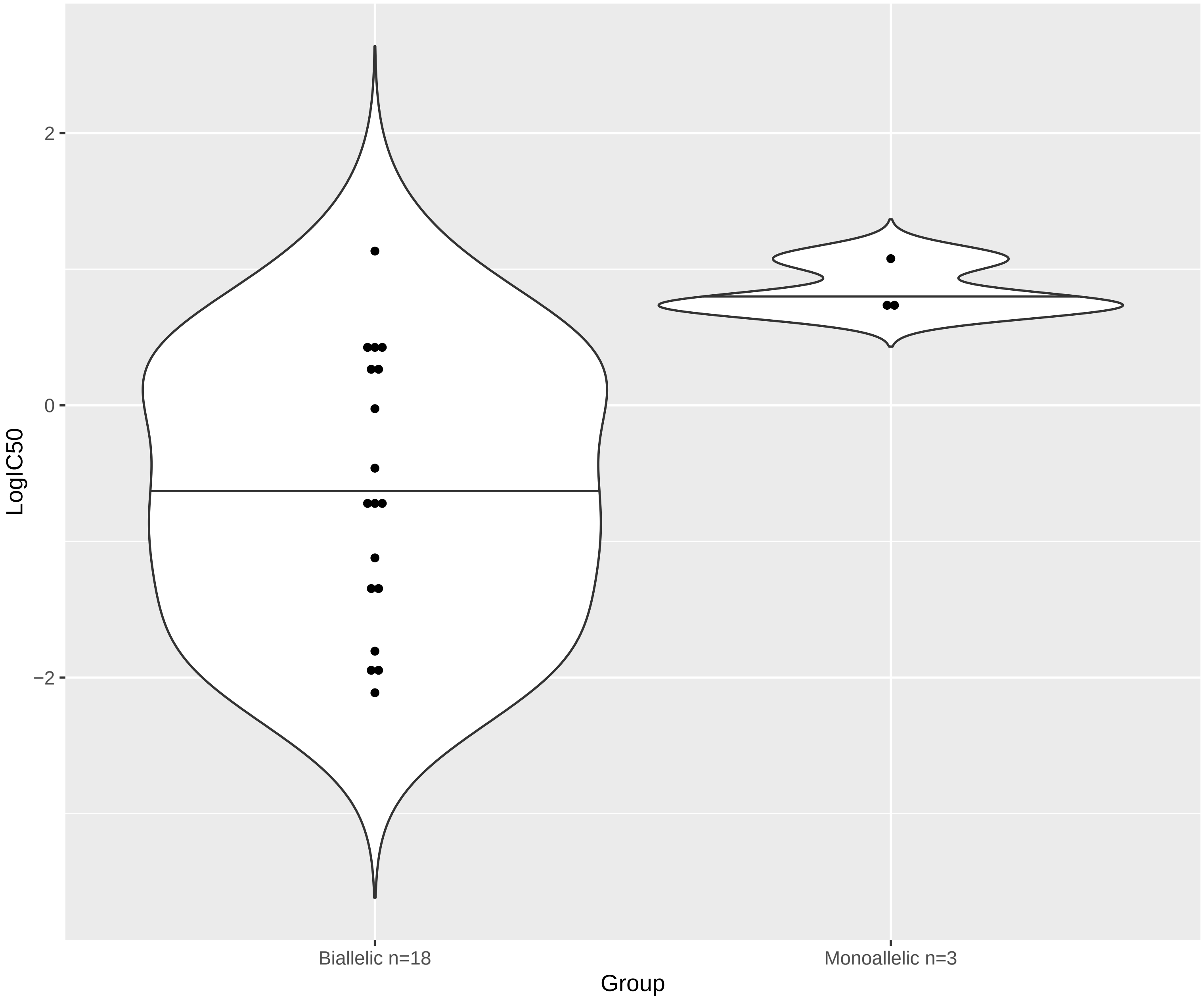

Feature: ENST00000349036.9_1; ENST00000371100.9_1; ENST00000371102.8_1; ENST00000464624.7_1; ENST00000676826.2_1
Gene Name: GNAS
Drug Name: XAV939

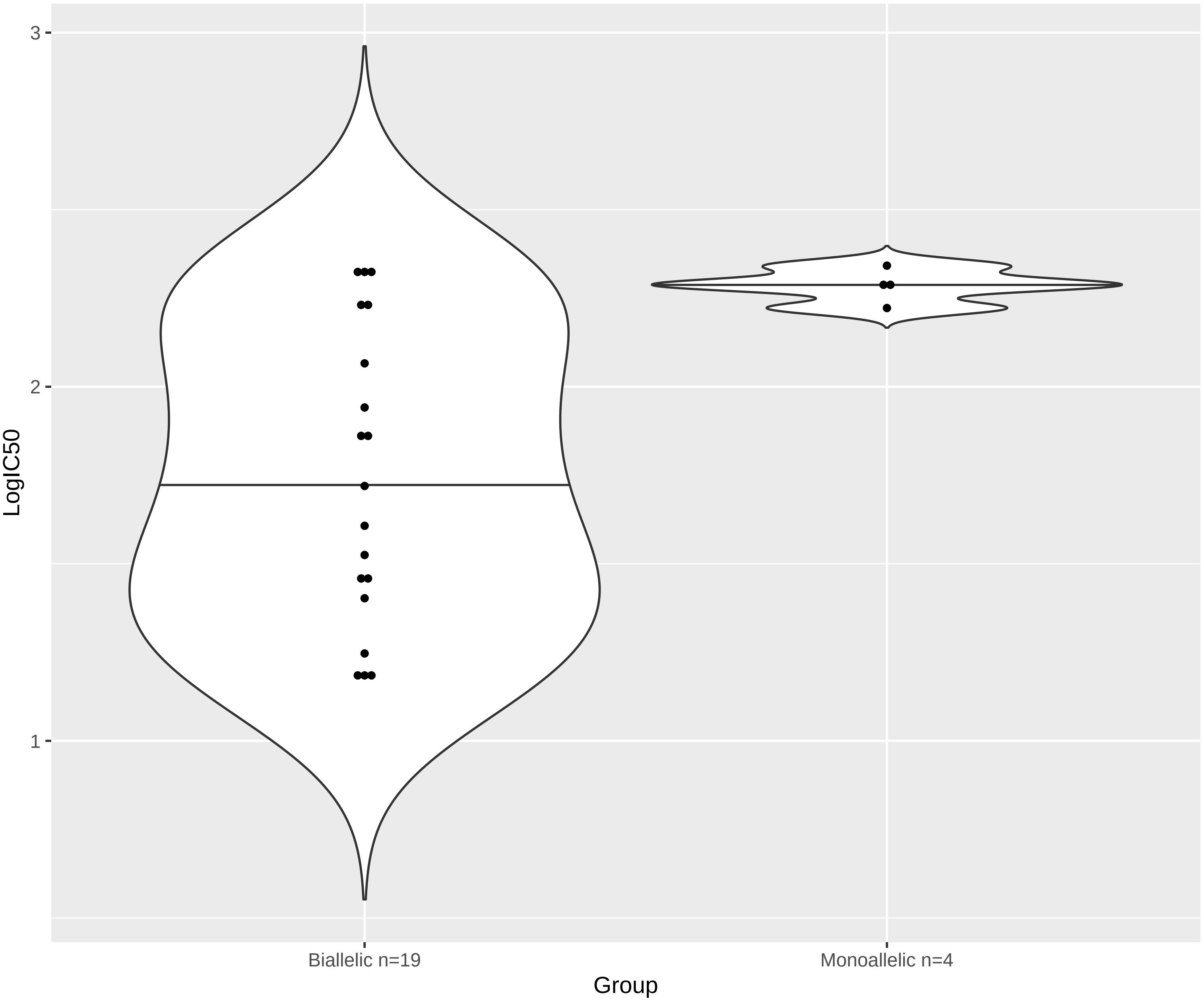

Feature: ENST00000572169.6_1
Gene Name: NAA60
Drug Name: cyclovalone

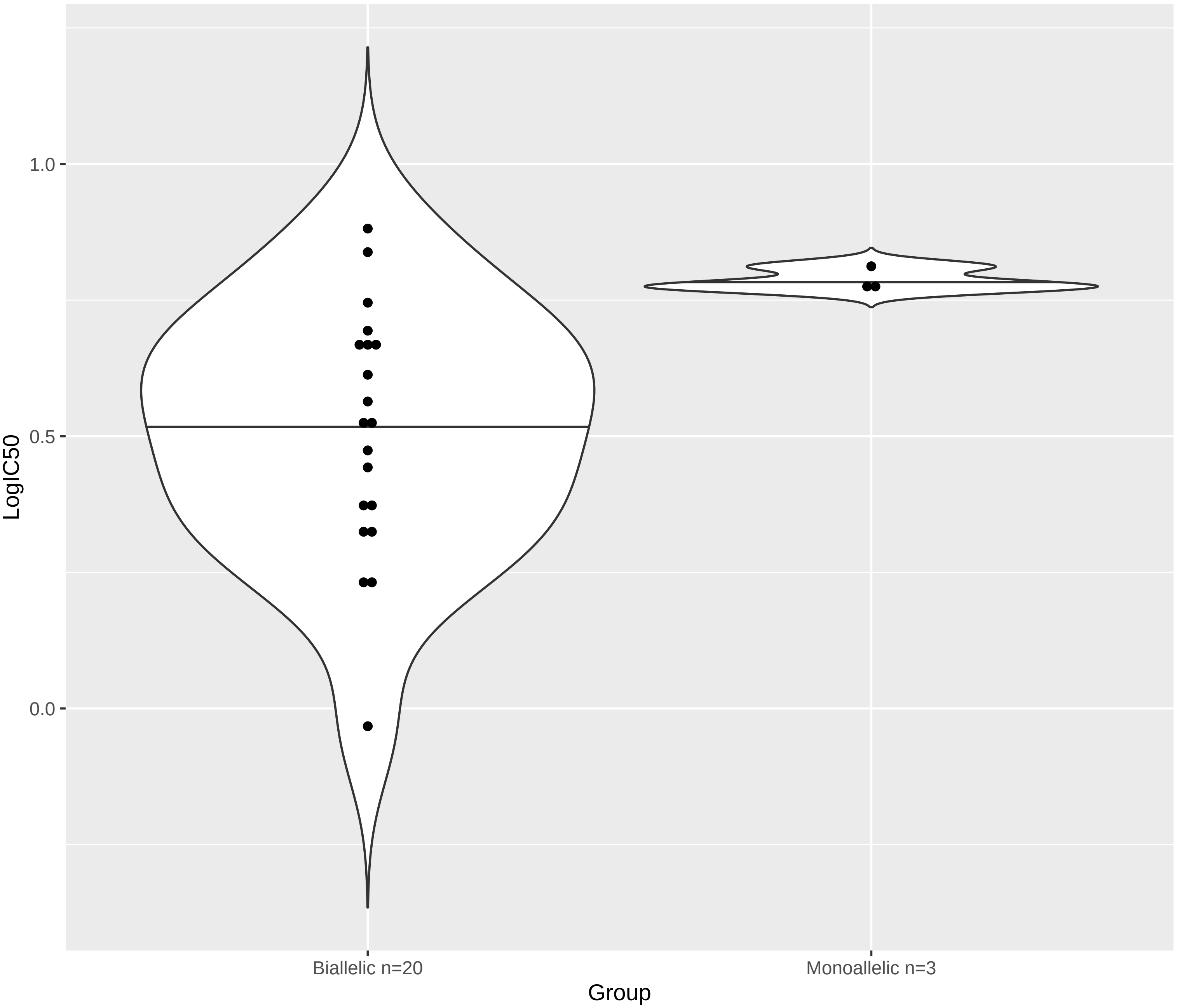

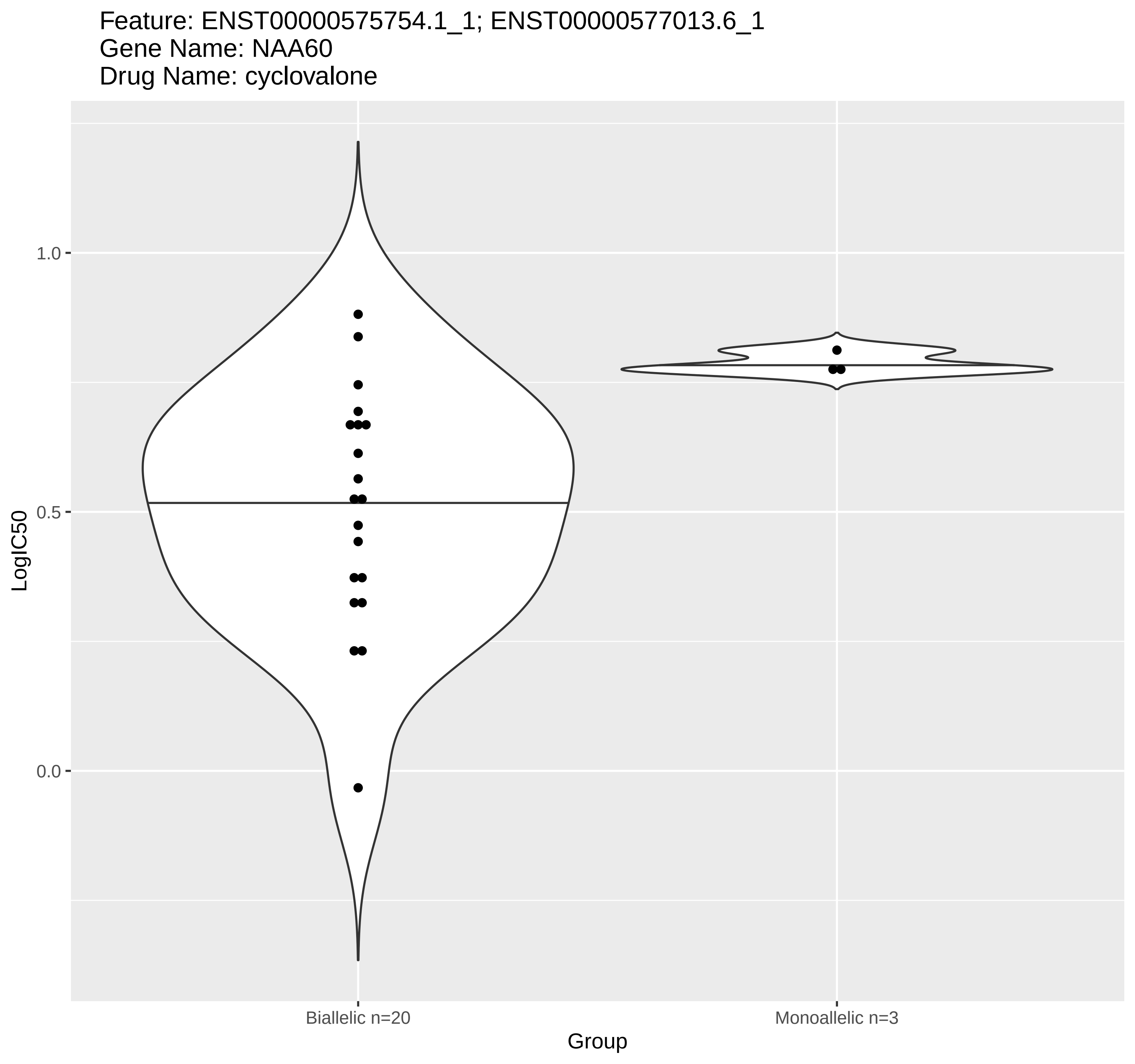

Feature: ENST00000575754.1_1; ENST00000577013.6_1
Gene Name: NAA60
Drug Name: cyclovalone
1.0
0.5
0.0
LogIC50
Biallelic n=20
Monoallelic n=3
Group

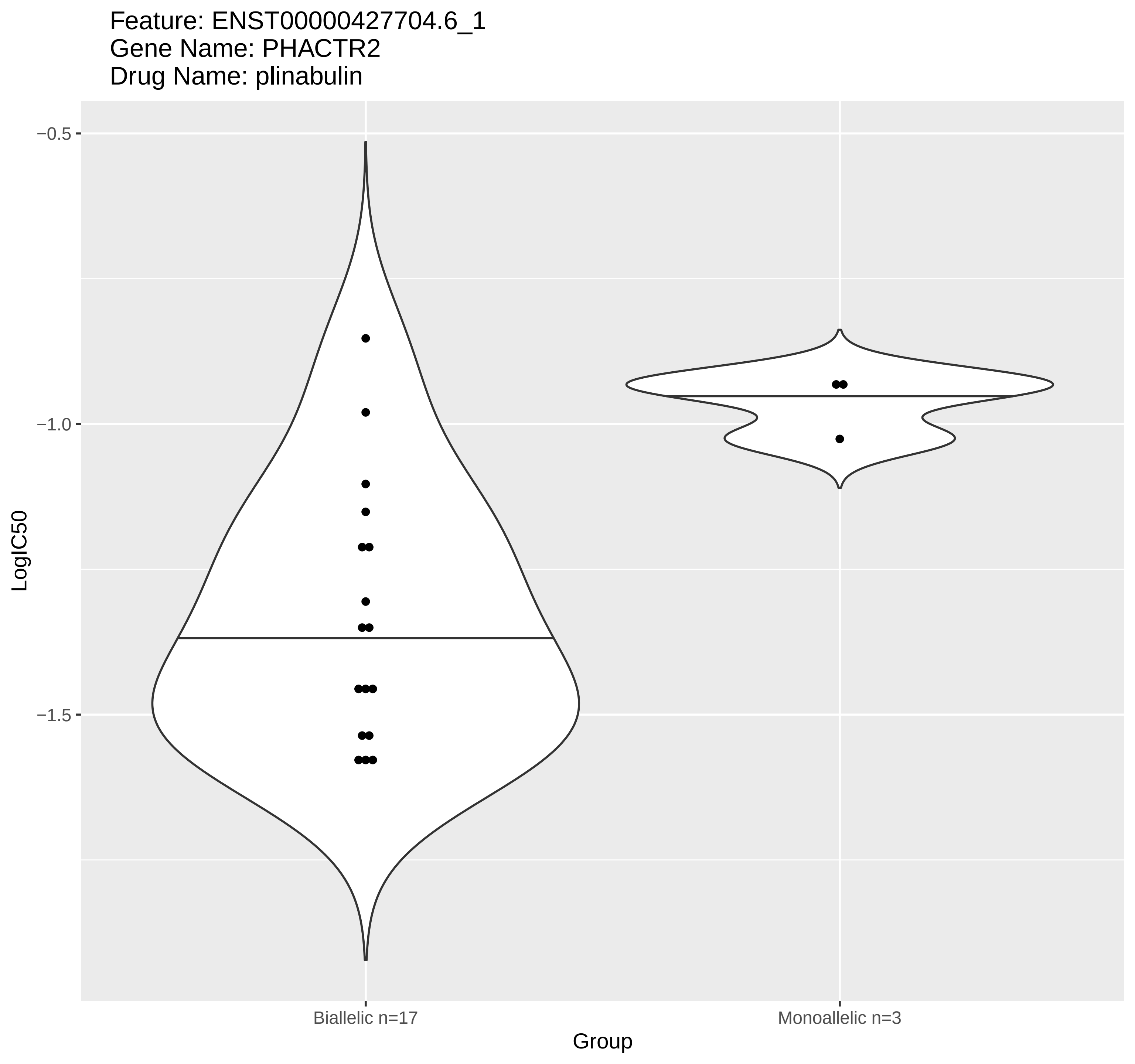
Feature: ENST00000427704.6_1
Gene Name: PHACTR2
Drug Name: plinabulin
LogIC50
−0.5
−1.0
−1.5
Biallelic n=17
Monoallelic n=3
Group

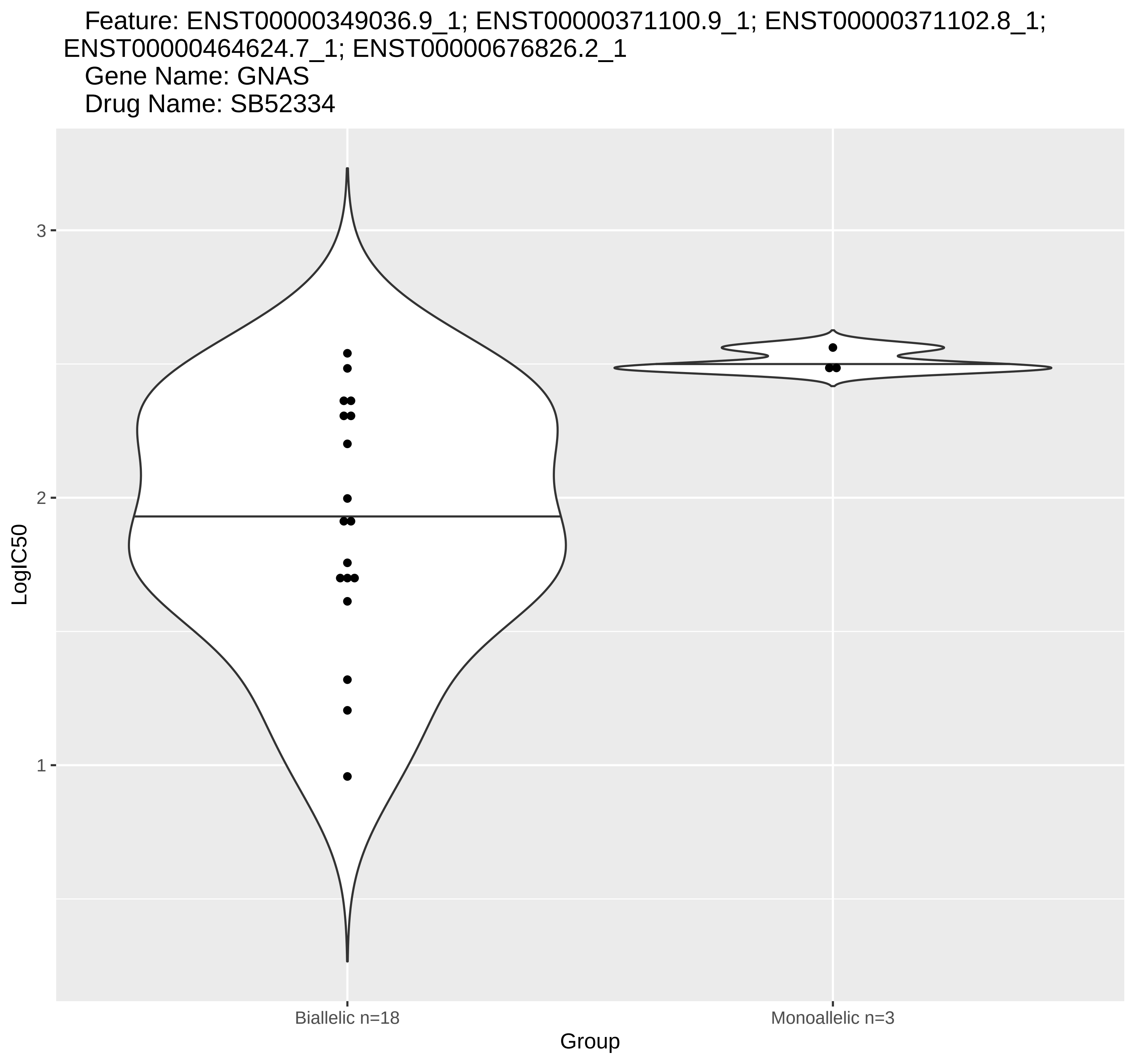

Feature: ENST00000349036.9_1; ENST00000371100.9_1; ENST00000371102.8_1; ENST00000464624.7_1; ENST00000676826.2_1
Gene Name: GNAS
Drug Name: SB52334
LogIC50
3
2
1
Biallelic n=18
Monoallelic n=3
Group

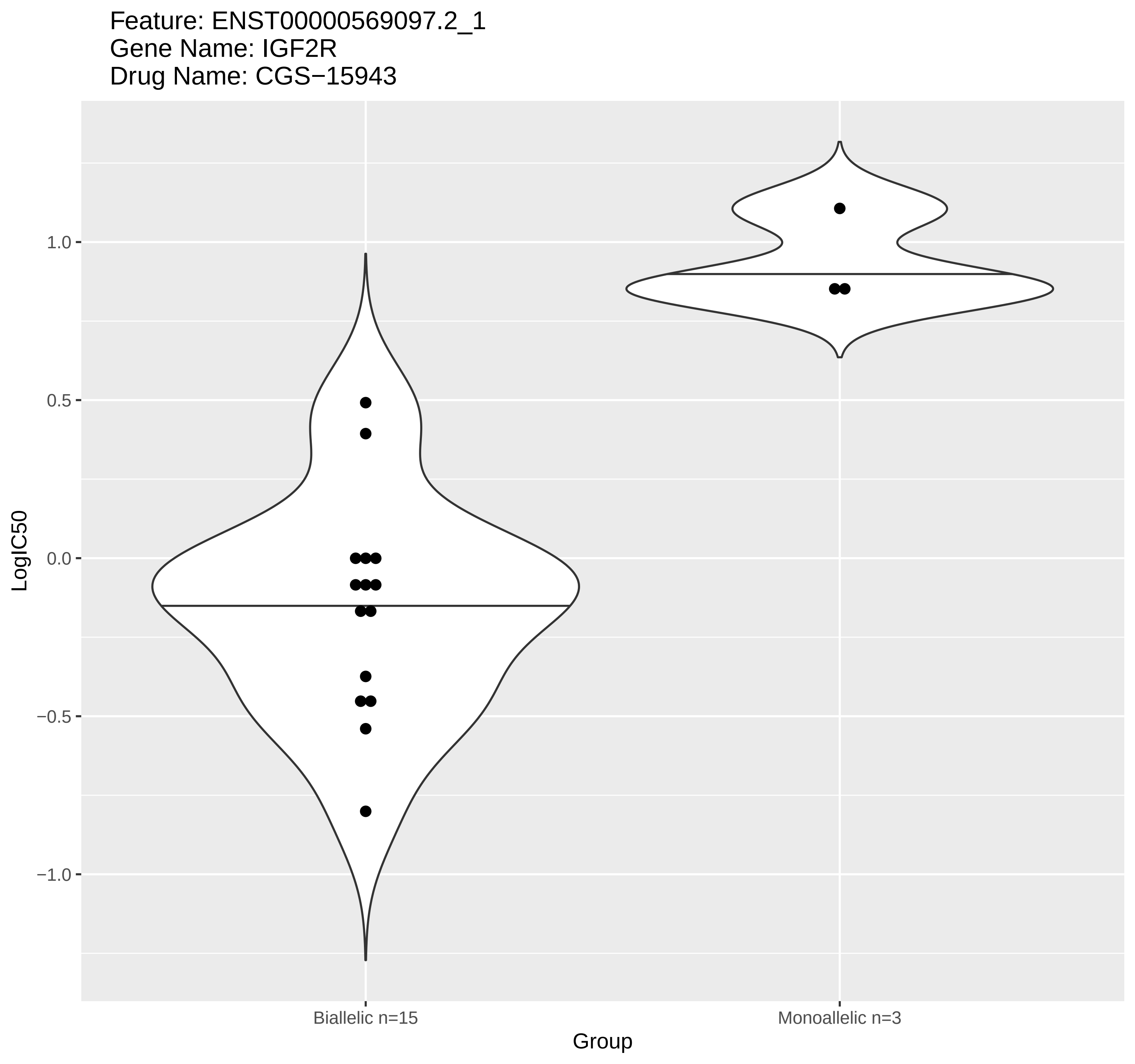
Feature: ENST00000569097.2_1
Gene Name: IGF2R
Drug Name: CGS−15943
LogIC50
1.0
0.5
0.0
−0.5
−1.0
Biallelic n=15
Monoallelic n=3
Group

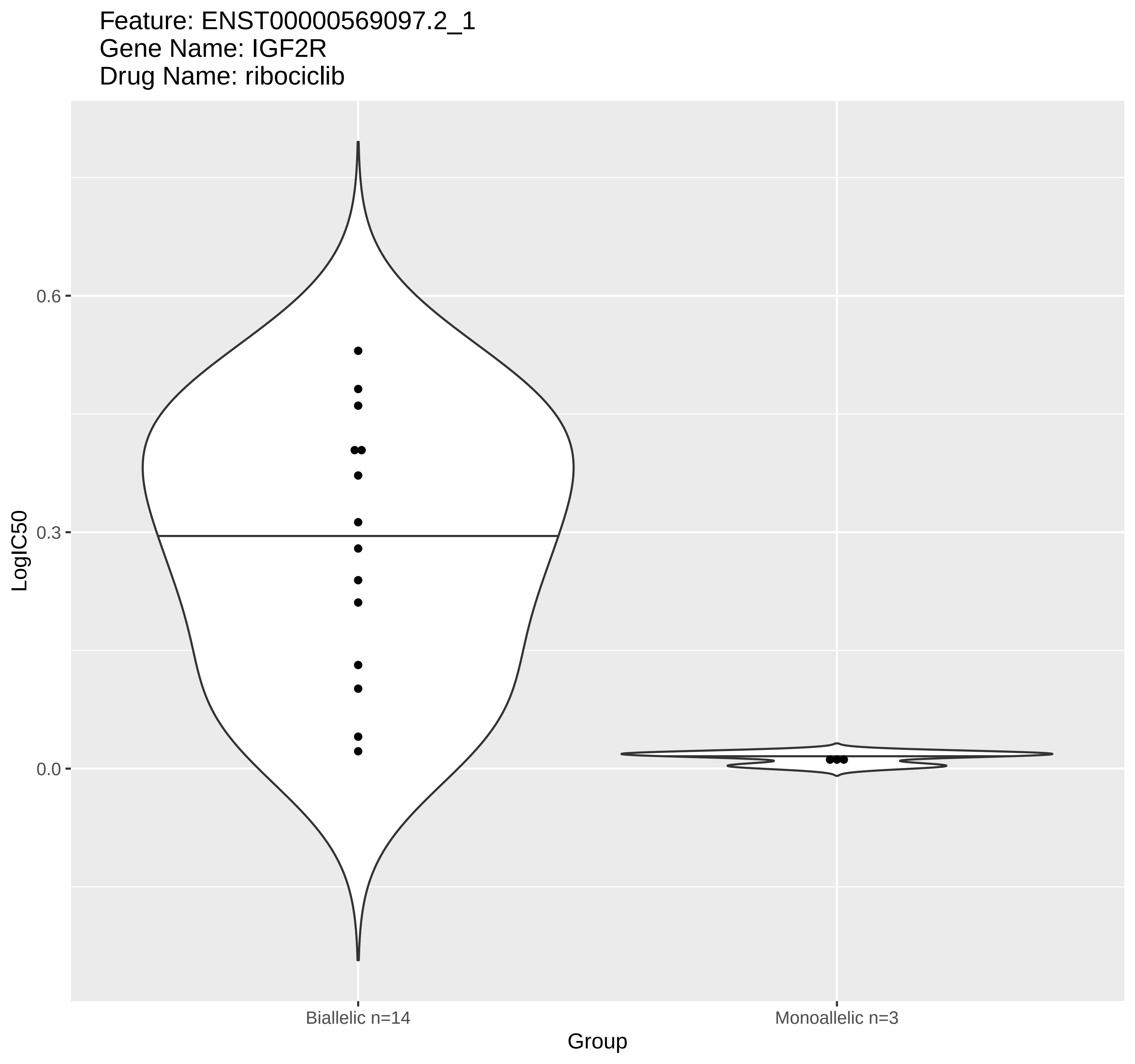

Feature: ENST00000569097.2_1
Gene Name: IGF2R
Drug Name: ribociclib
LogIC50
0.6
0.3
0.0
Biallelic n=14
Monoallelic n=3
Group

Feature: ENST00000493259.5_1
Gene Name: CPA4
Drug Name: danusertib

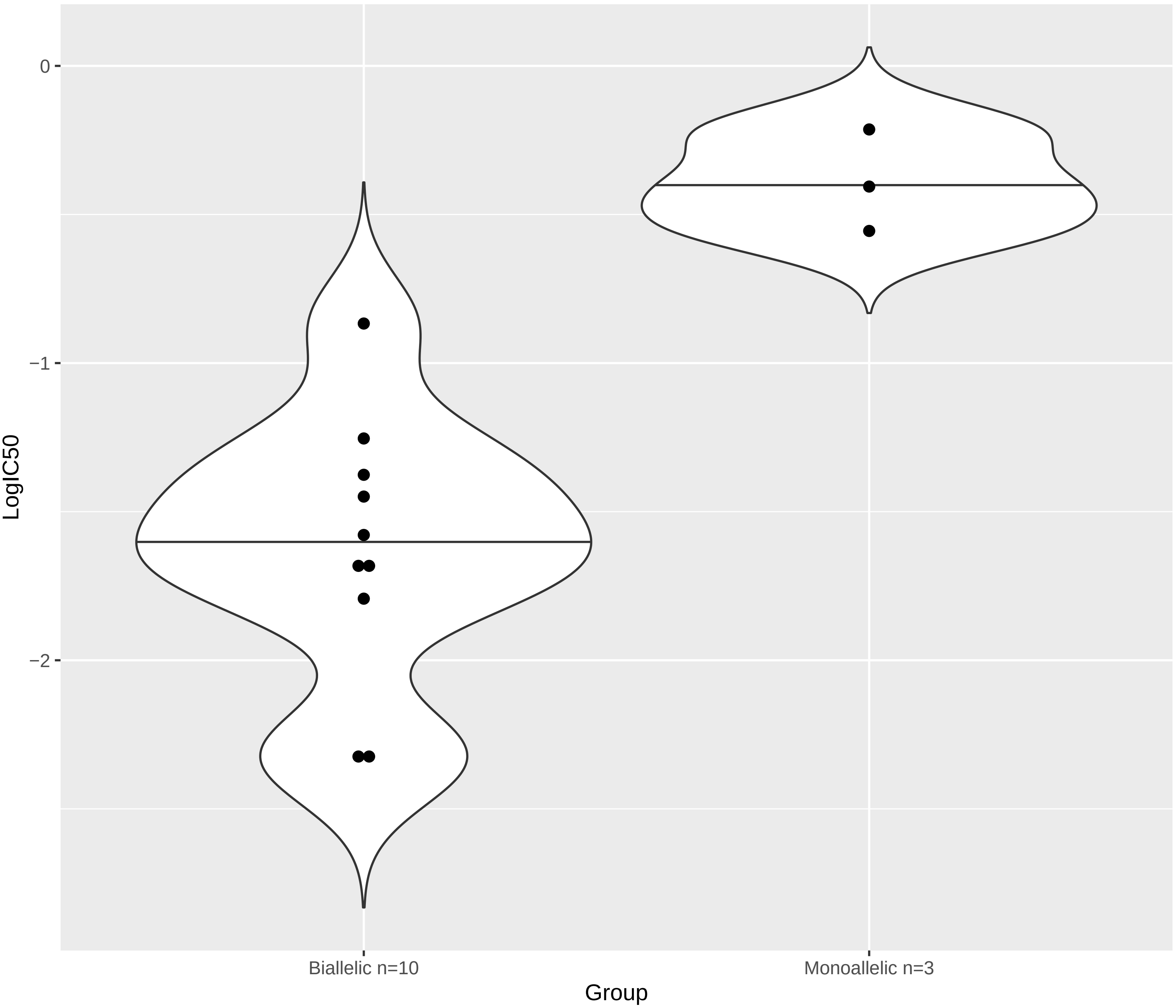

Feature: ENST00000313949.11_1; ENST00000371075.7_1; ENST00000419558.7_1
Gene Name: GNAS
Drug Name: KIN001−270

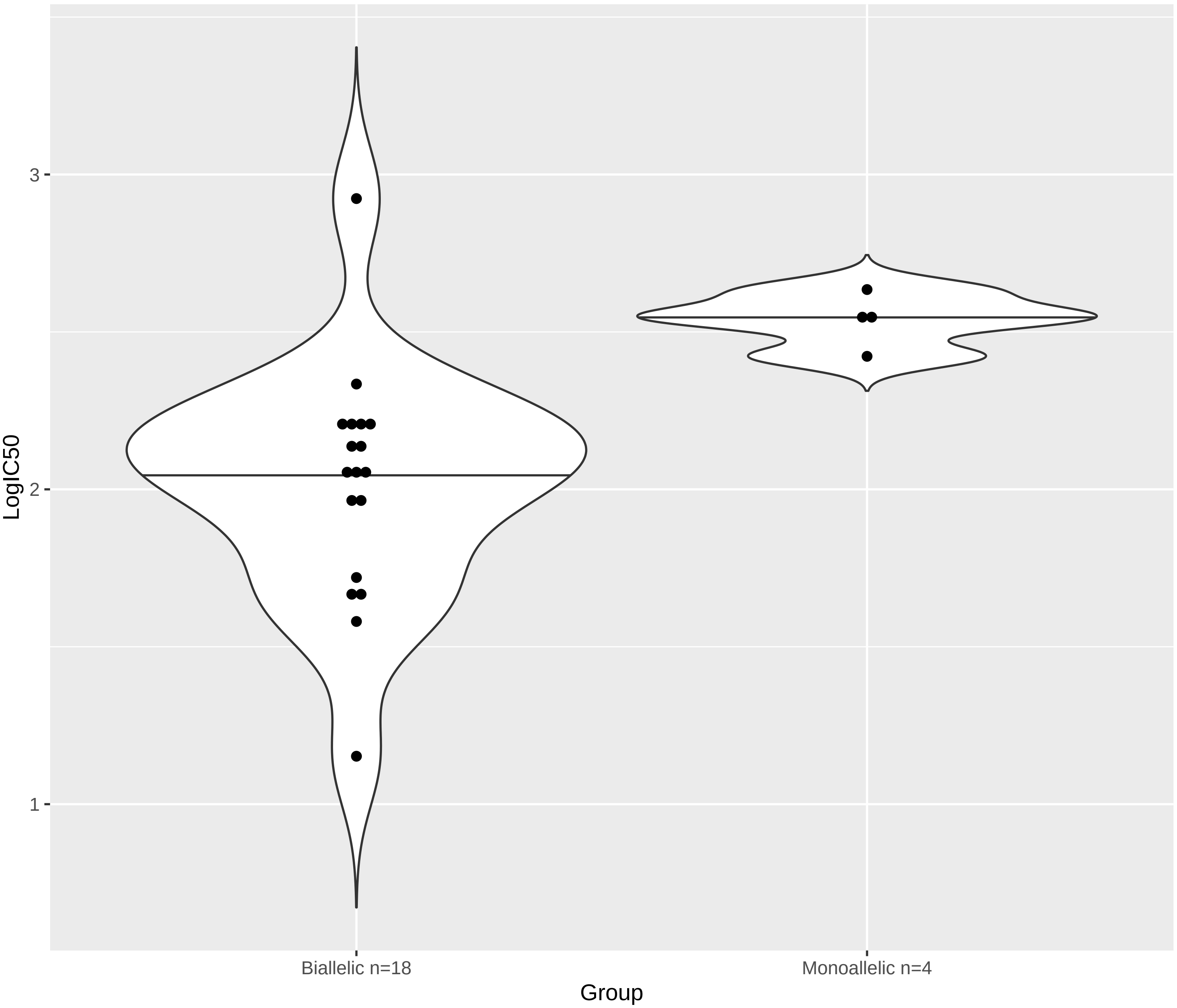

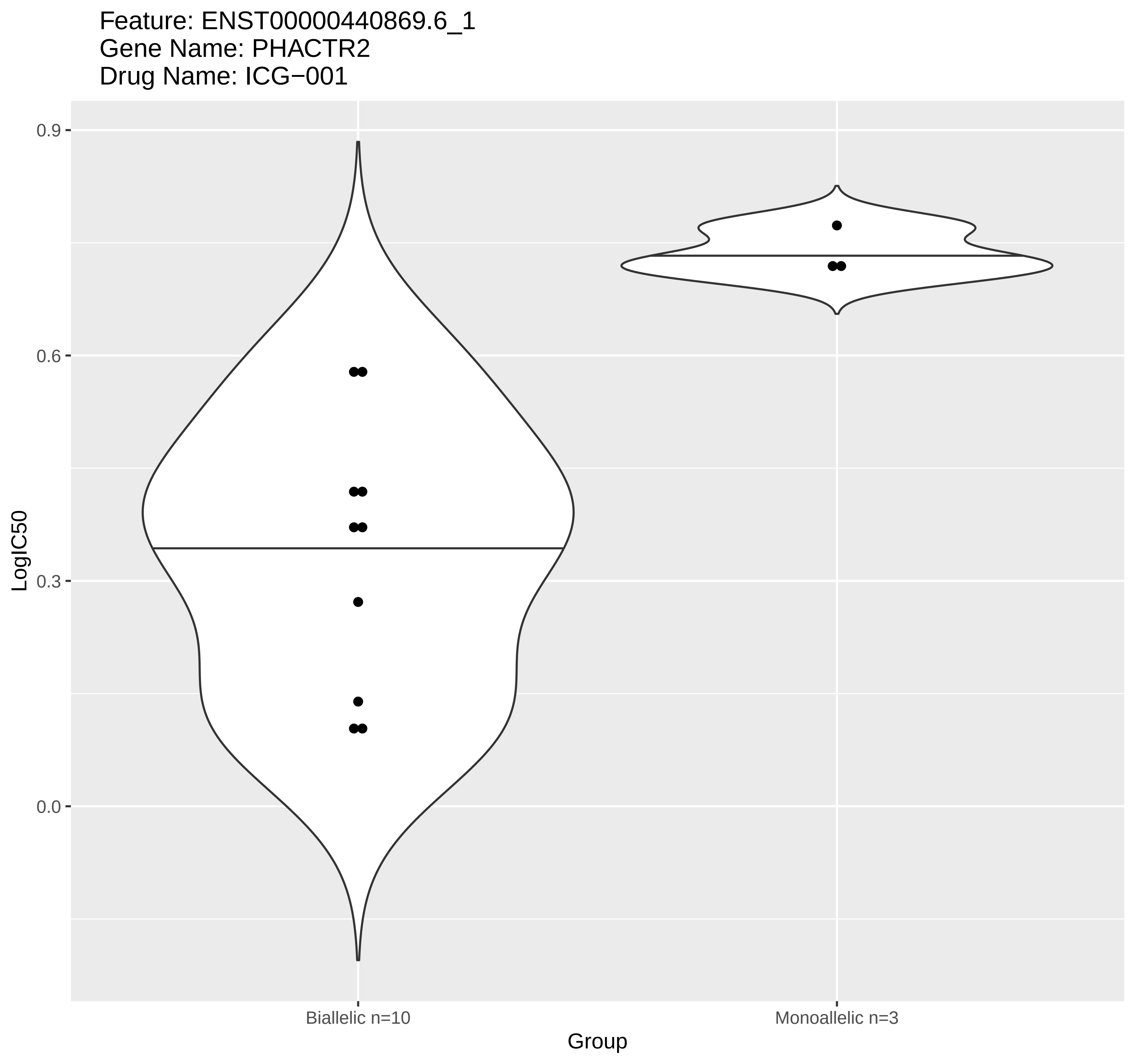

Feature: ENST00000440869.6_1
Gene Name: PHACTR2
Drug Name: ICG−001
0.9
0.6
0.3
0.0
LogIC50
Biallelic n=10
Monoallelic n=3
Group

Feature: ENST00000401949.6_1; ENST00000644769.1_1
Gene Name: GRB10
Drug Name: VER−49009

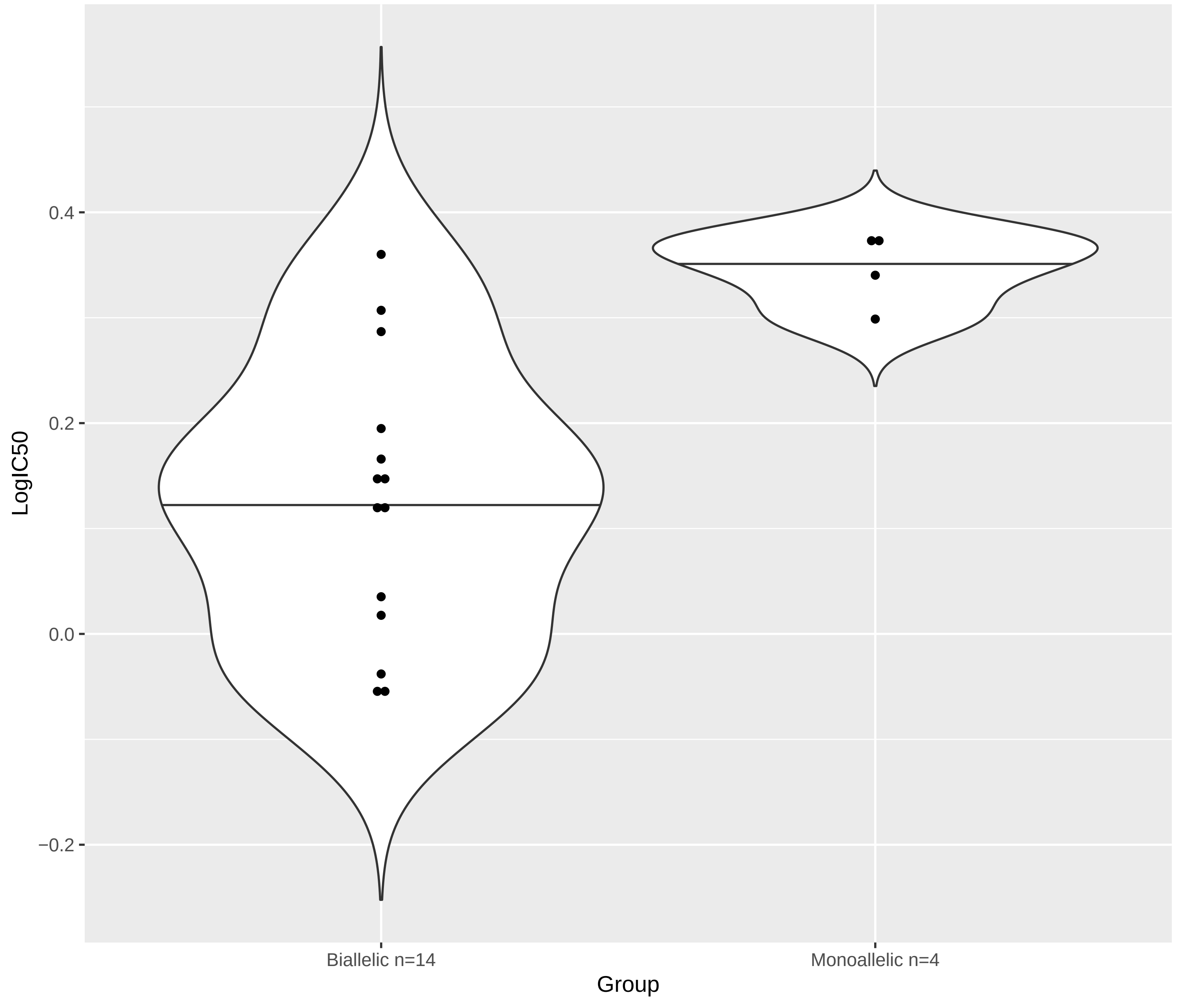

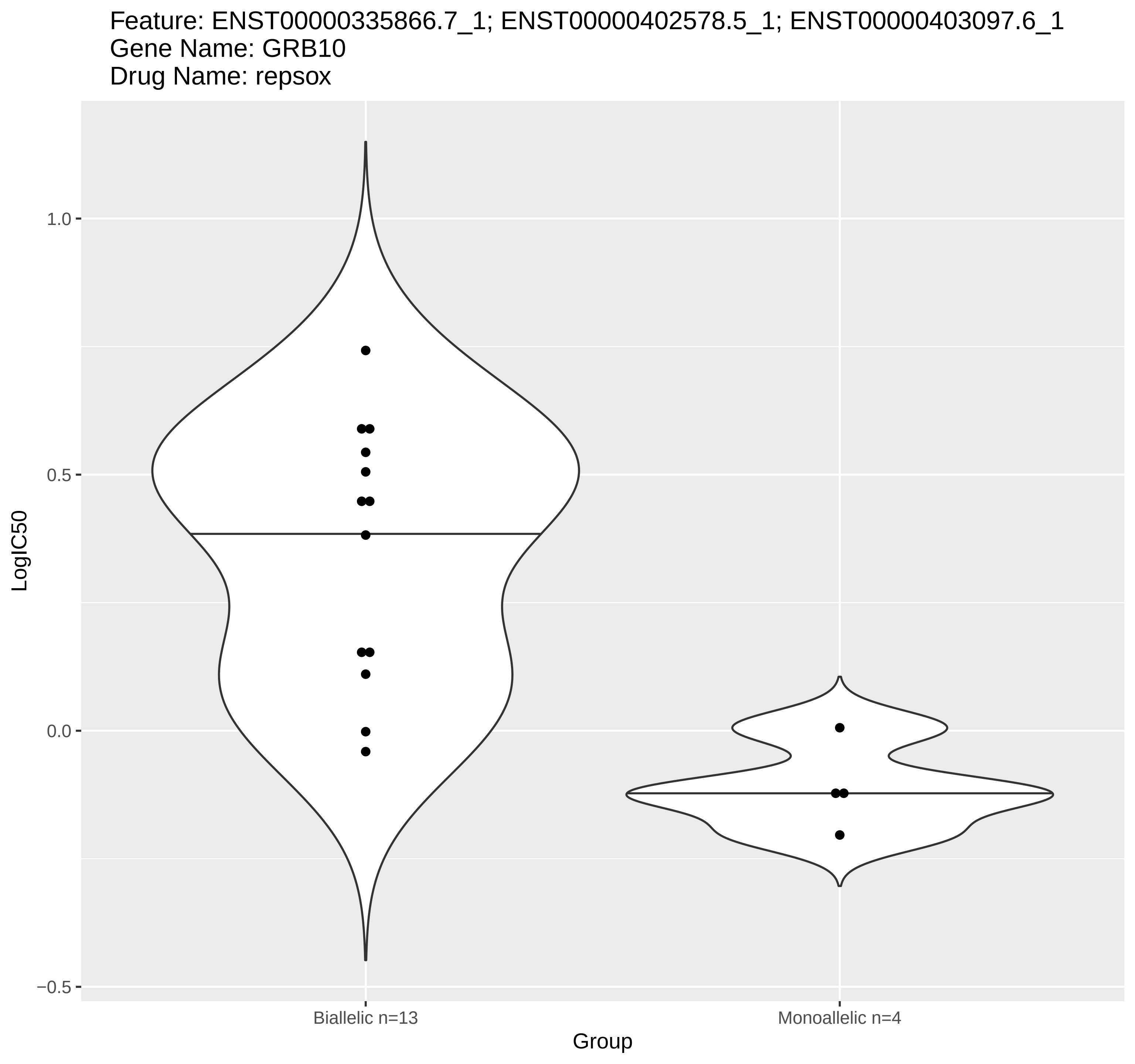
Feature: ENST00000335866.7_1; ENST00000402578.5_1; ENST00000403097.6_1
Gene Name: GRB10
Drug Name: repsox
LogIC50
1.0
0.5
0.0
−0.5
Biallelic n=13
Monoallelic n=4
Group

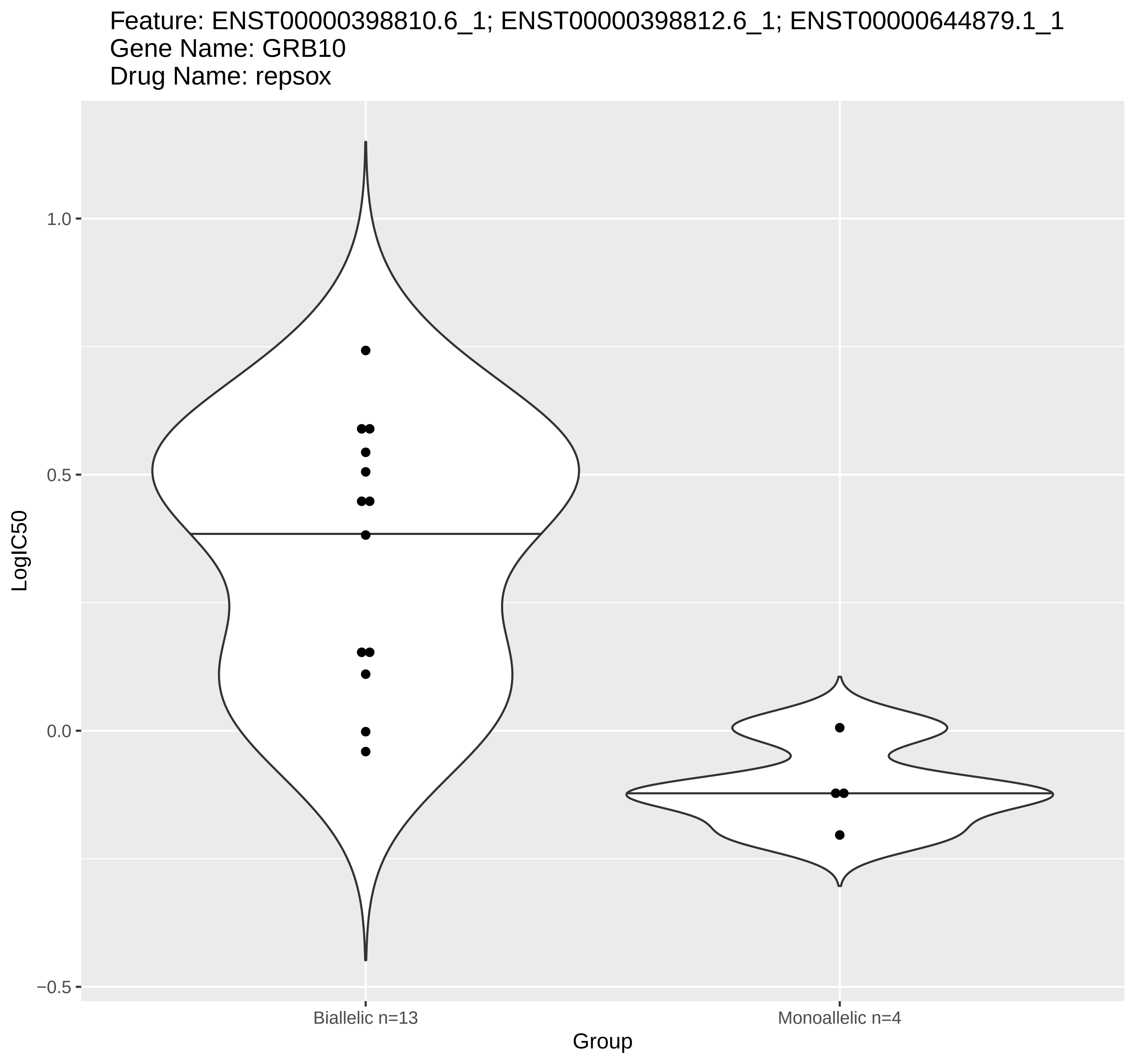

Feature: ENST00000398810.6_1; ENST00000398812.6_1; ENST00000644879.1_1
Gene Name: GRB10
Drug Name: repsox
LogIC50
1.0
0.5
0.0
−0.5
Biallelic n=13
Monoallelic n=4
Group

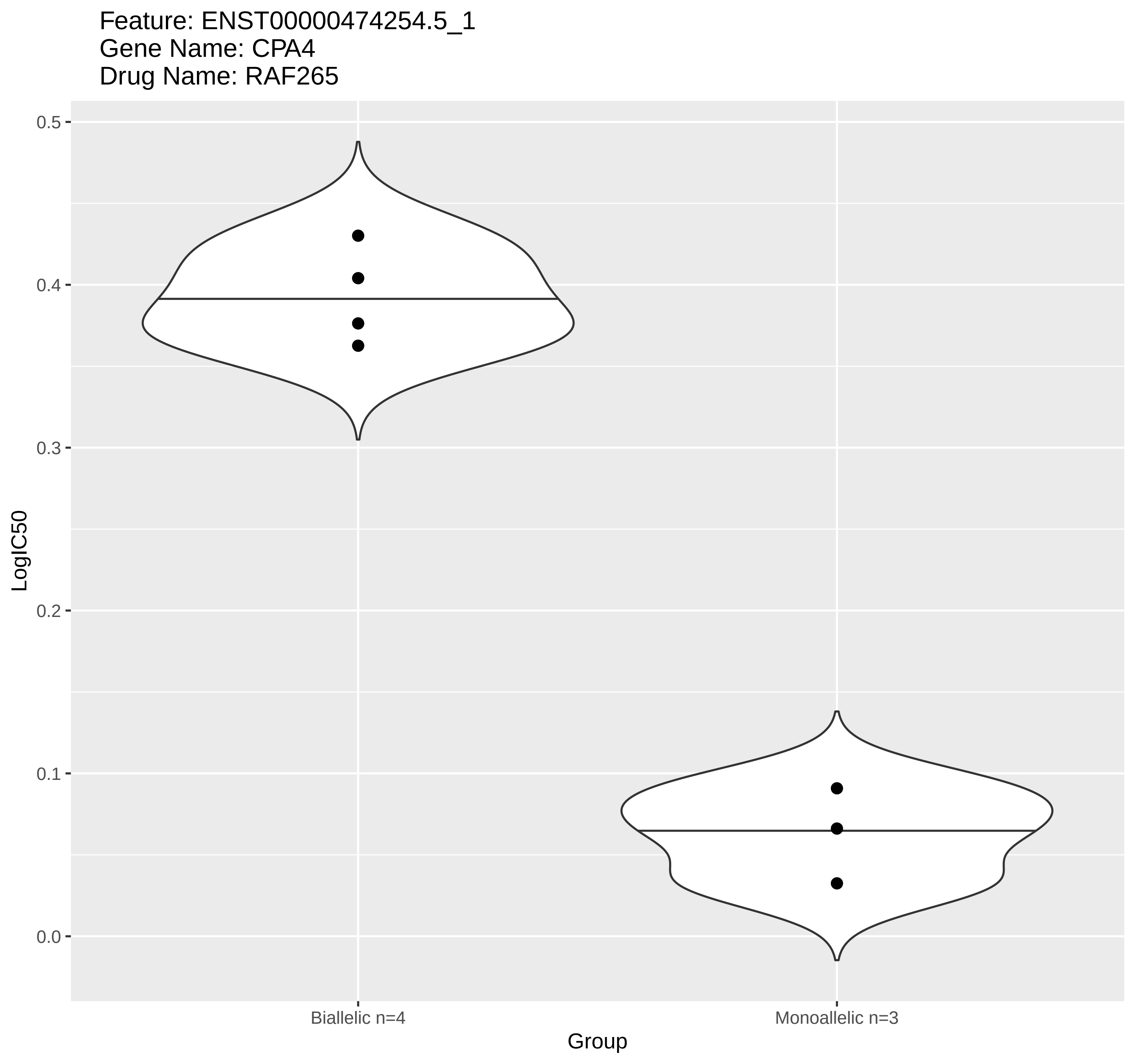

Feature: ENST00000474254.5_1
Gene Name: CPA4
Drug Name: RAF265
LogIC50
0.0
0.1
0.2
0.3
0.4
0.5
Biallelic n=4
Monoallelic n=3
Group

Feature: ENST00000490117.5_1
Gene Name: CPA4
Drug Name: RAF265

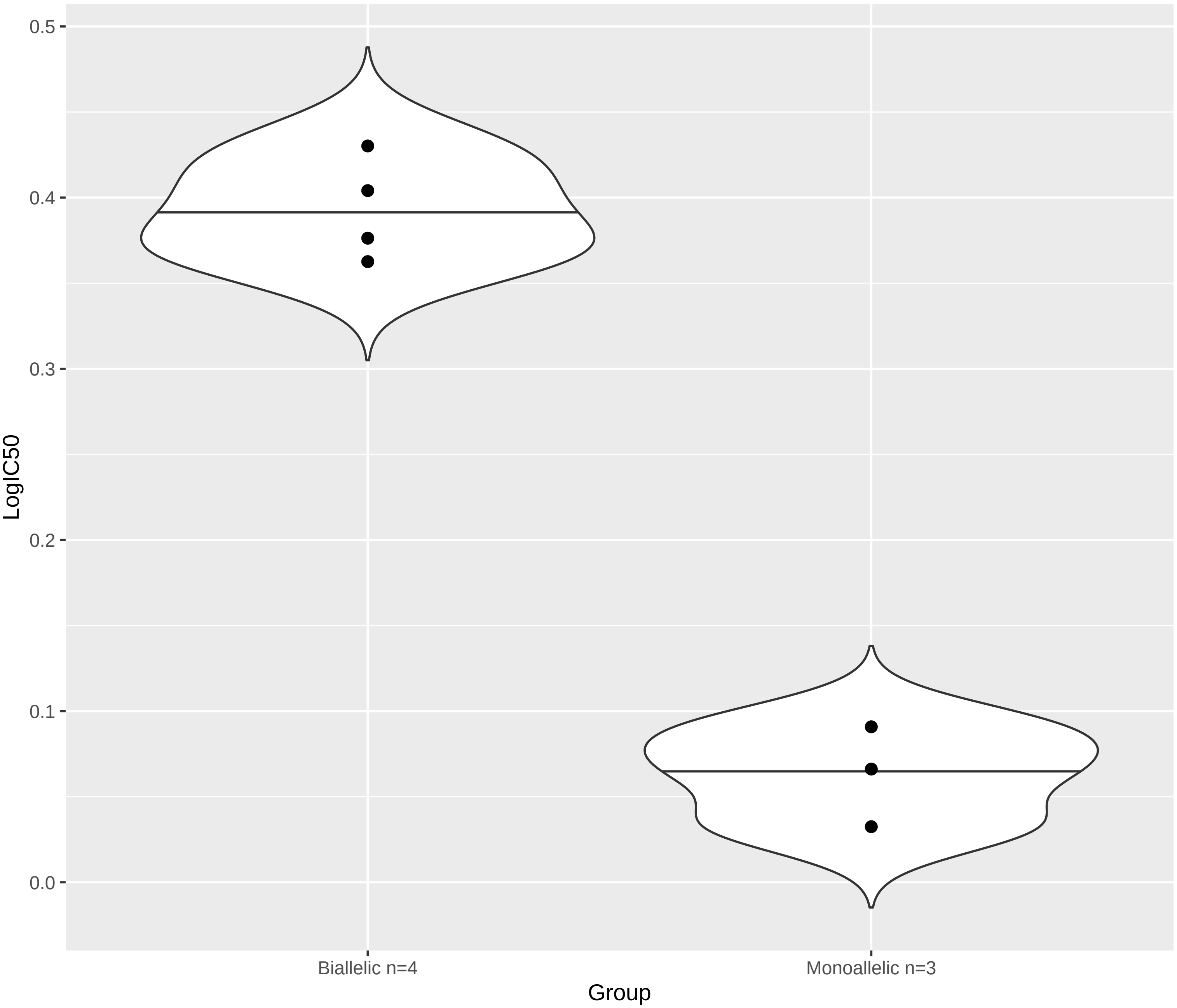

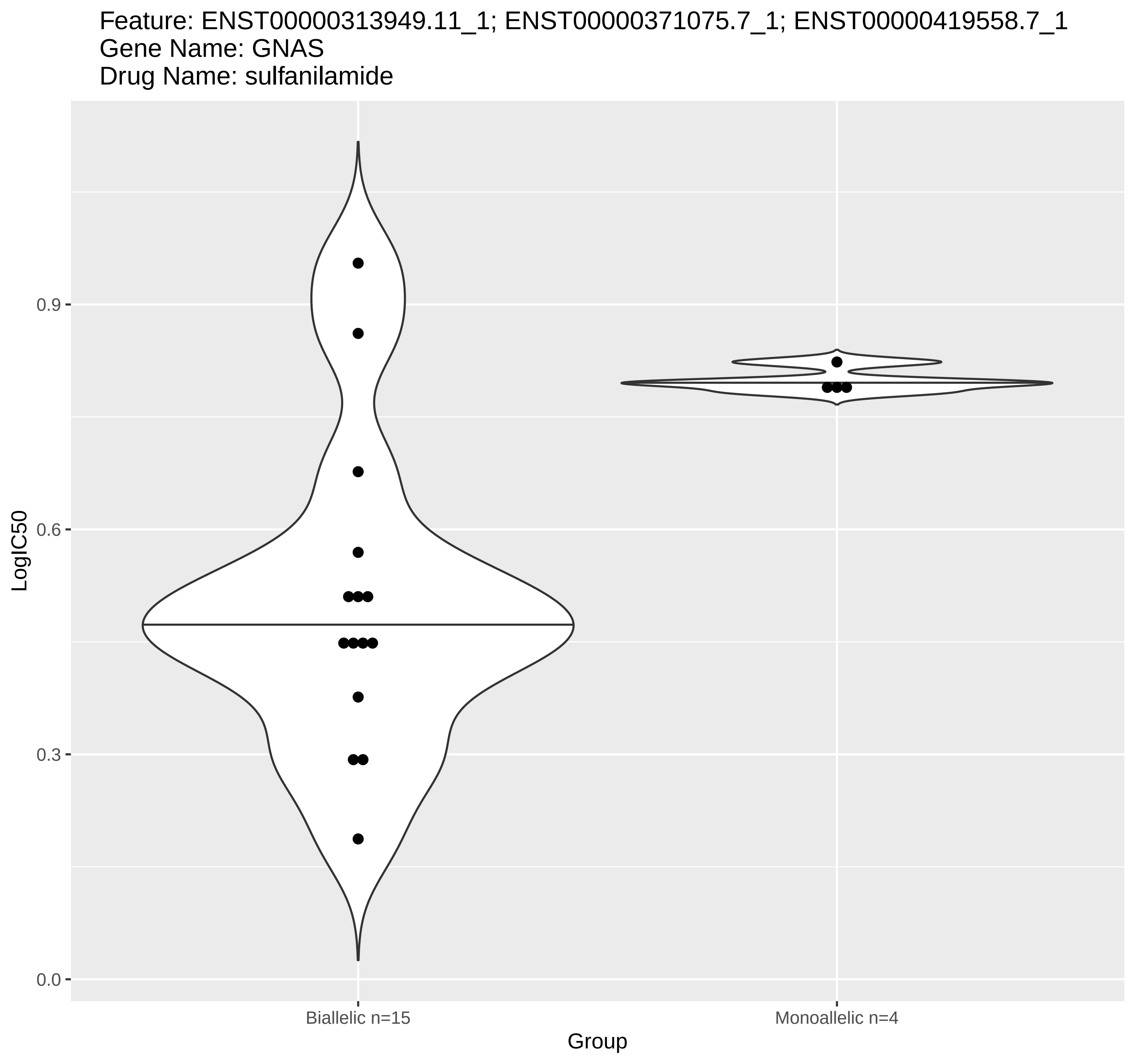

Feature: ENST00000313949.11_1; ENST00000371075.7_1; ENST00000419558.7_1
Gene Name: GNAS
Drug Name: sulfanilamide
LogIC50
0.9
0.6
0.3
0.0
Biallelic n=15
Monoallelic n=4
Group

Feature: ENST00000534491.5_1
Gene Name: OSBPL5
Drug Name: SR−33805

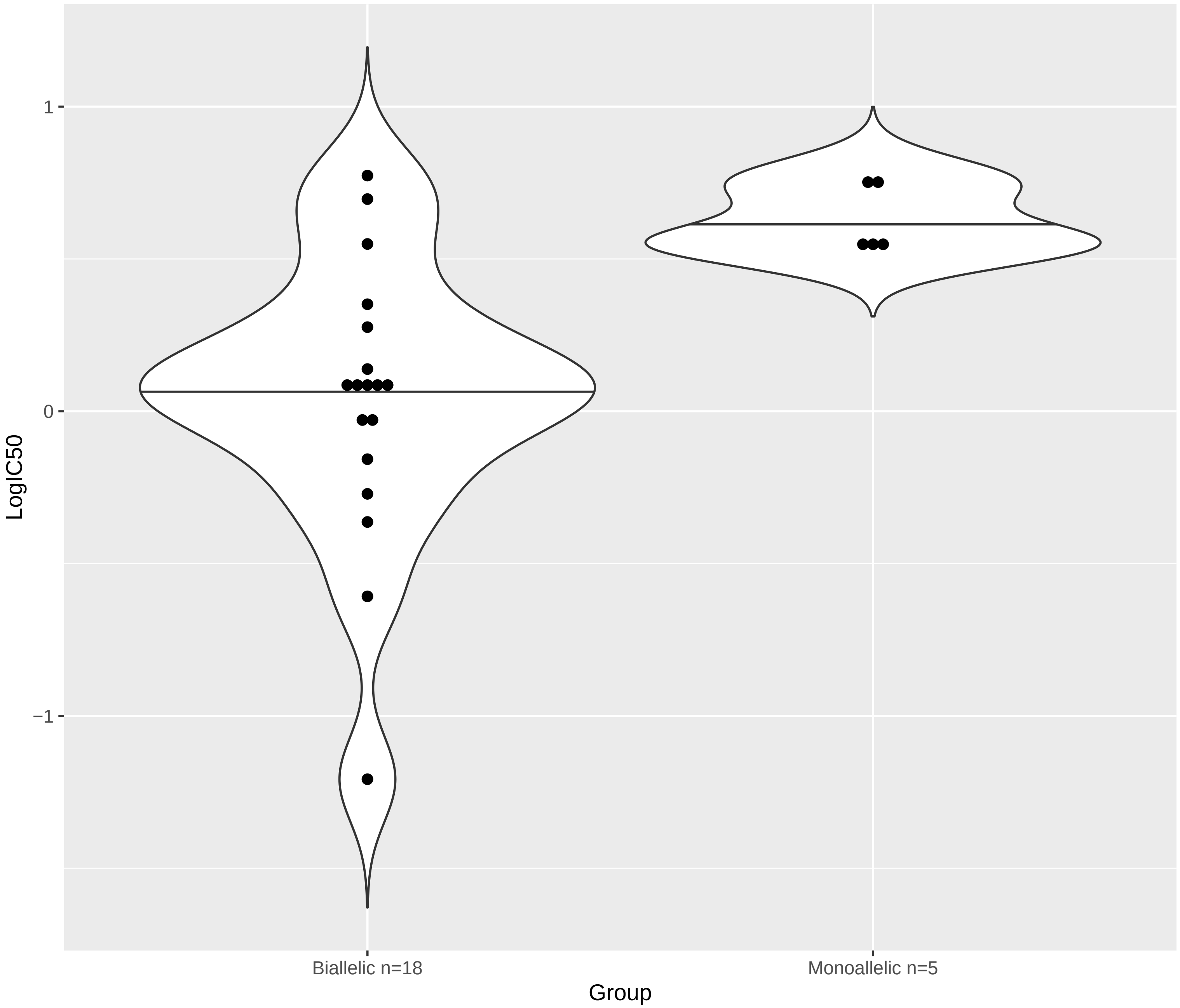

Feature: ENST00000355303.9_1; ENST00000530676.5_1
Gene Name: ANO1
Drug Name: ziprasidone

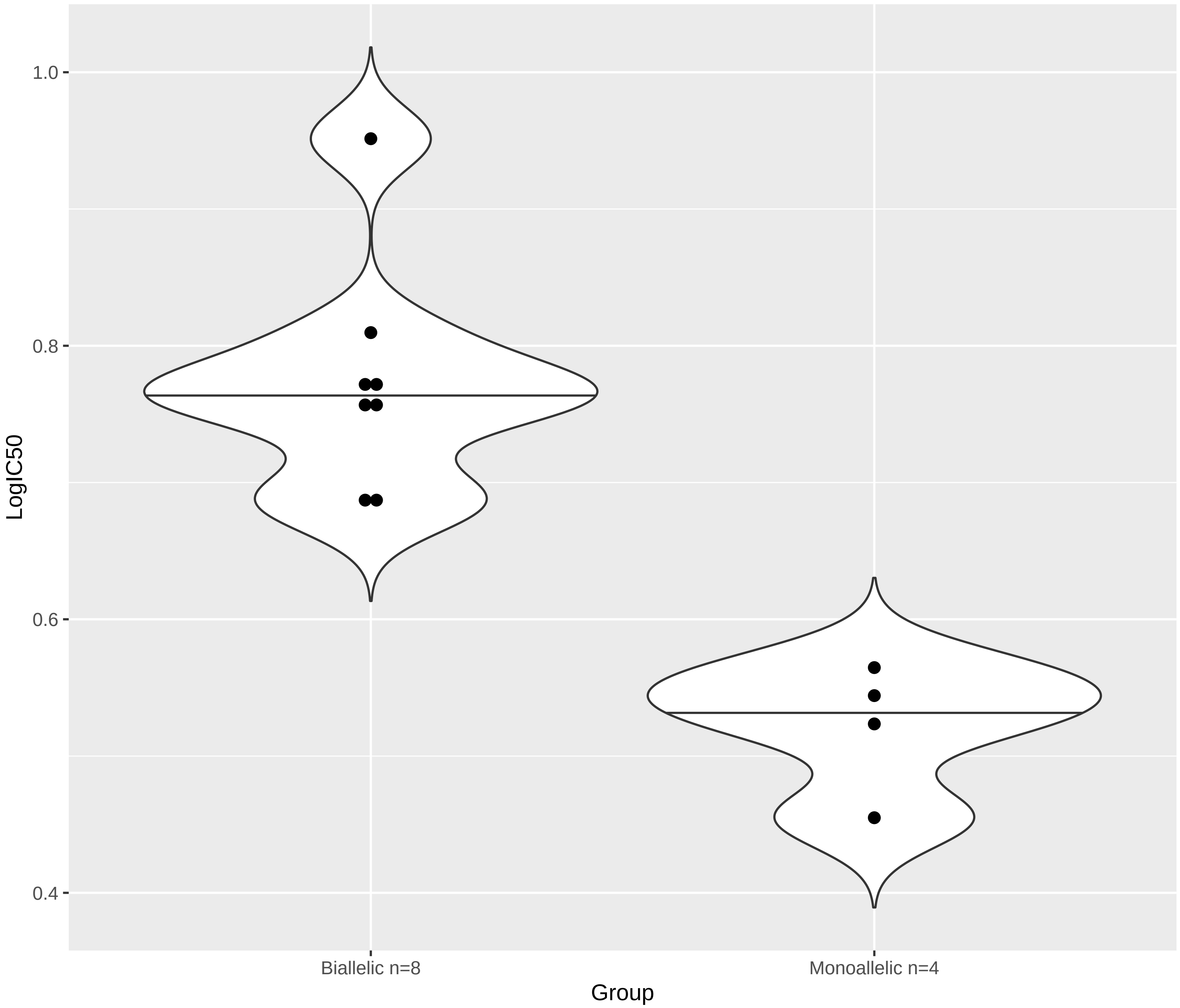

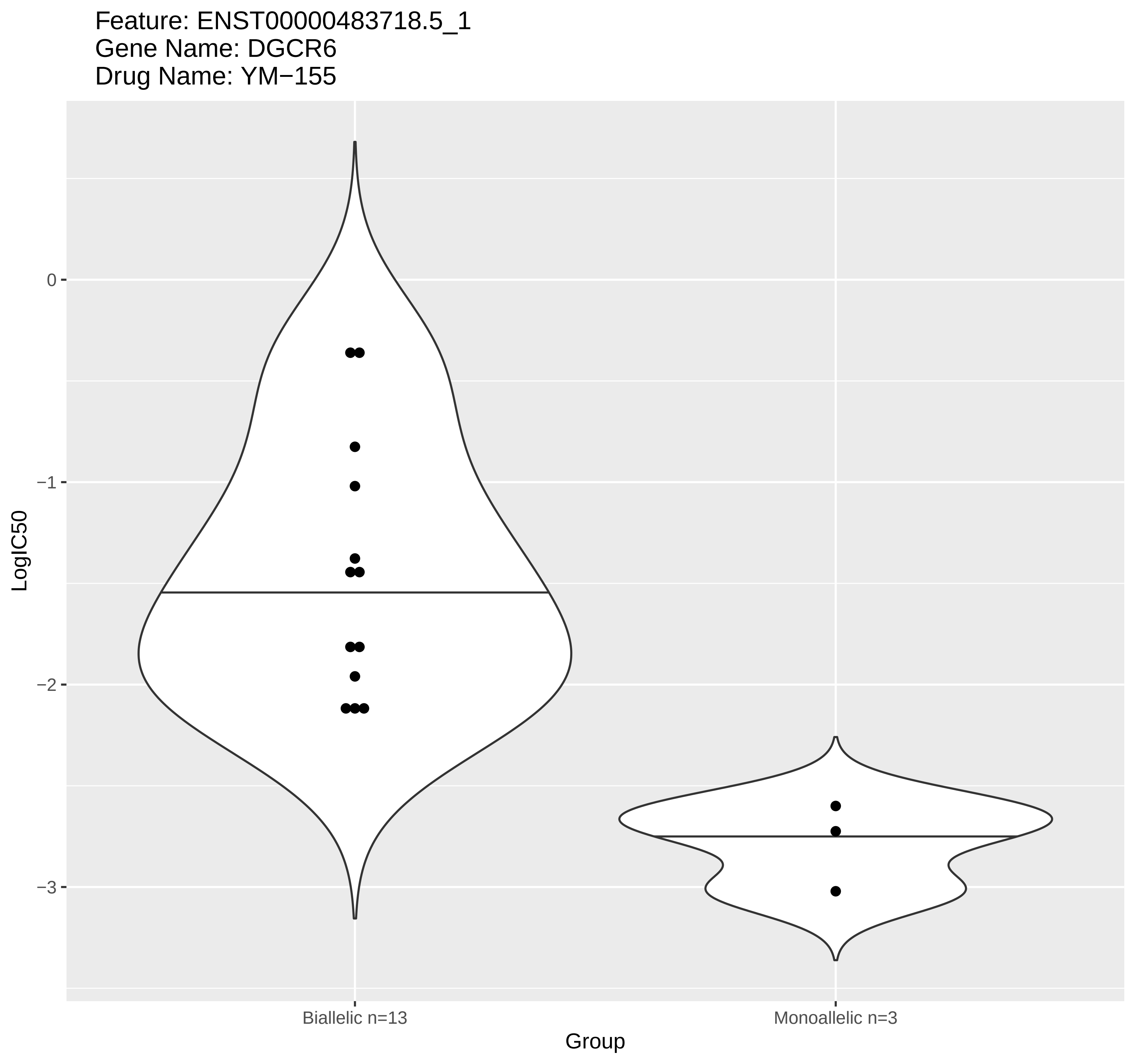
Feature: ENST00000483718.5_1
Gene Name: DGCR6
Drug Name: YM−155
LogIC50
0
−1
−2
−3
Biallelic n=13
Monoallelic n=3
Group

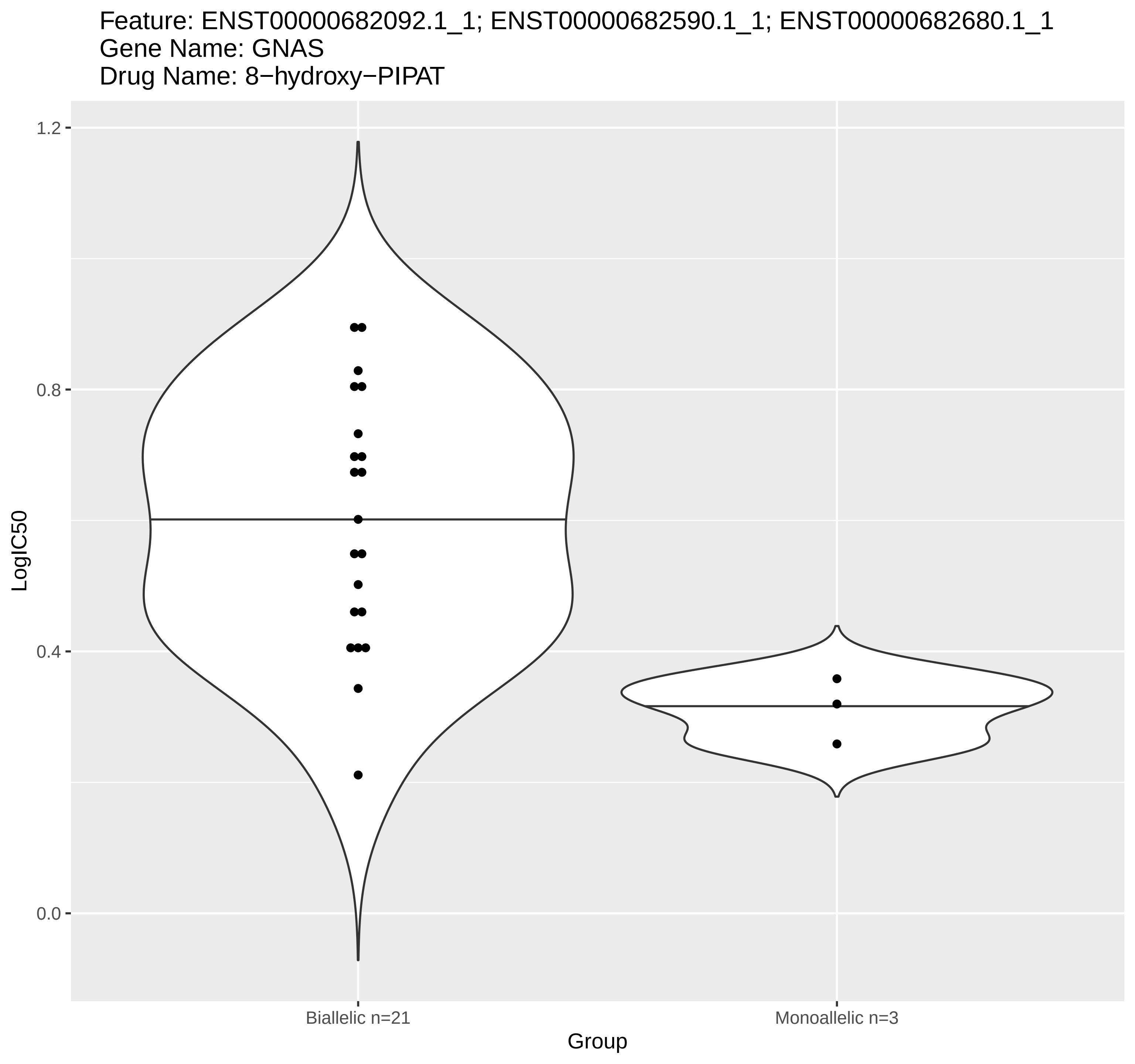
Feature: ENST00000682092.1_1; ENST00000682590.1_1; ENST00000682680.1_1
Gene Name: GNAS
Drug Name: 8−hydroxy−PIPAT
LogIC50
1.2
0.8
0.4
0.0
Biallelic n=21
Monoallelic n=3
Group

Feature: ENST00000682986.1_1; ENST00000683632.1_1; ENST00000684644.1_1
Gene Name: GNAS
Drug Name: 8−hydroxy−PIPAT

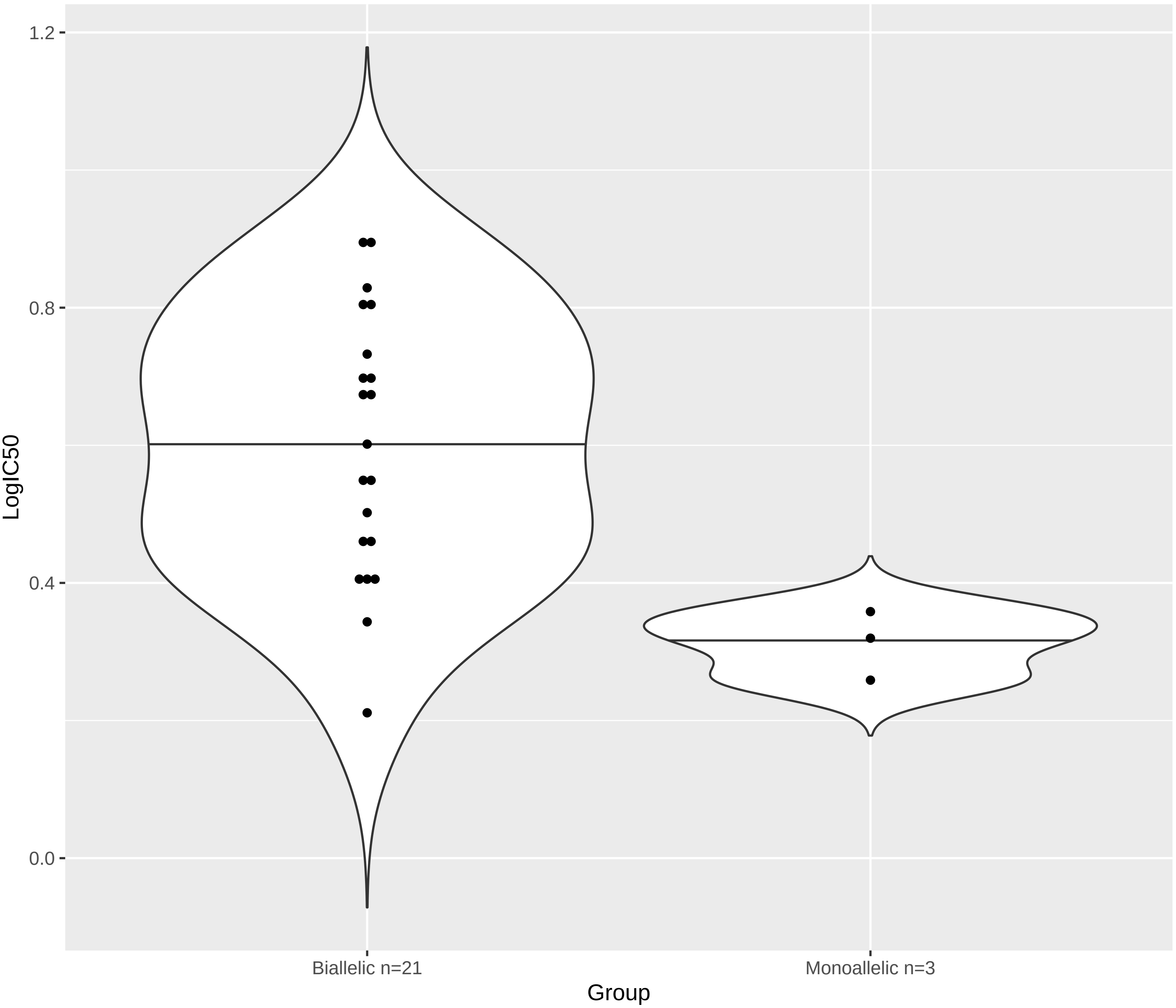

Feature: ENST00000591764.1_1
Gene Name: DNMT1
Drug Name: UNC1215

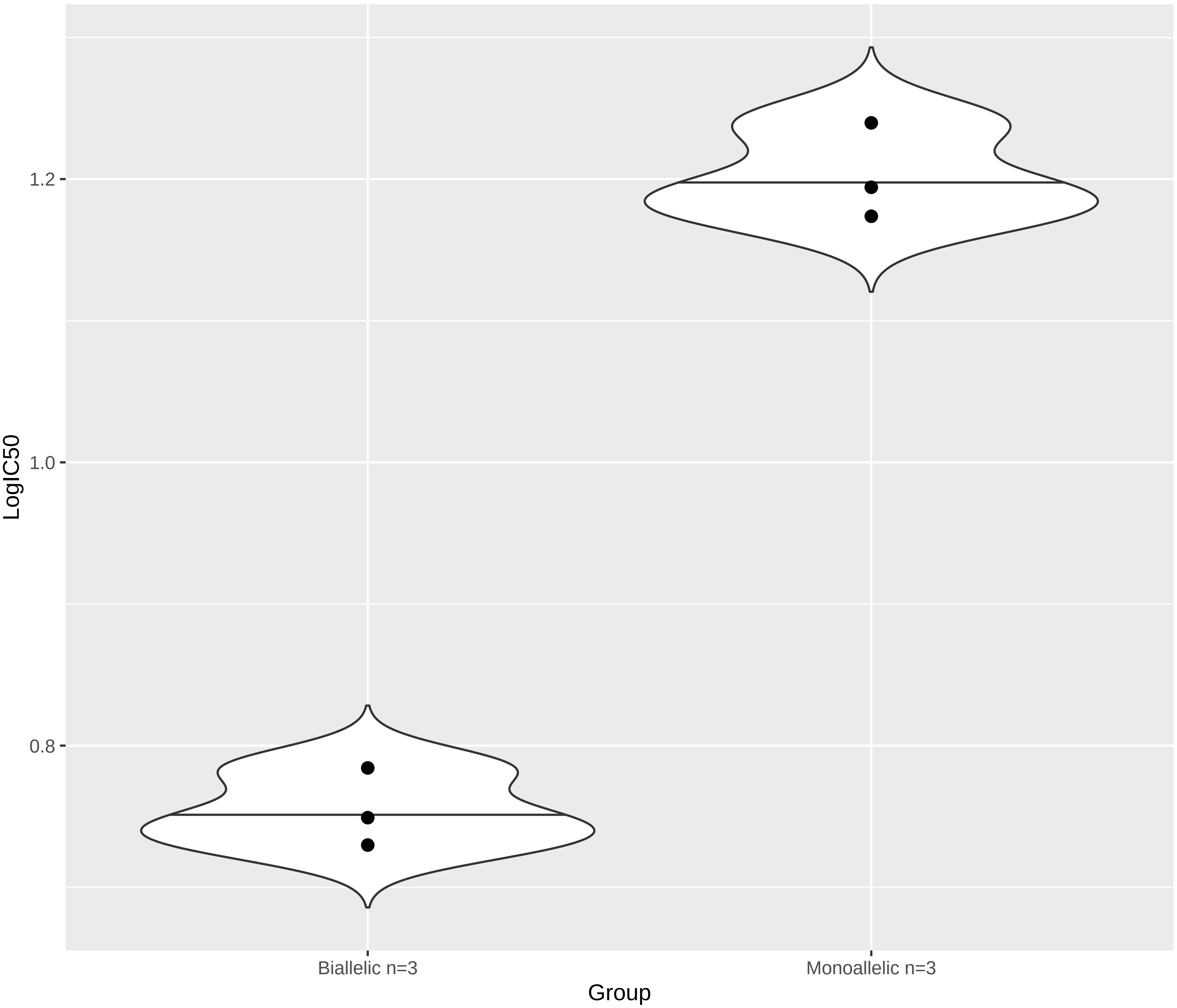

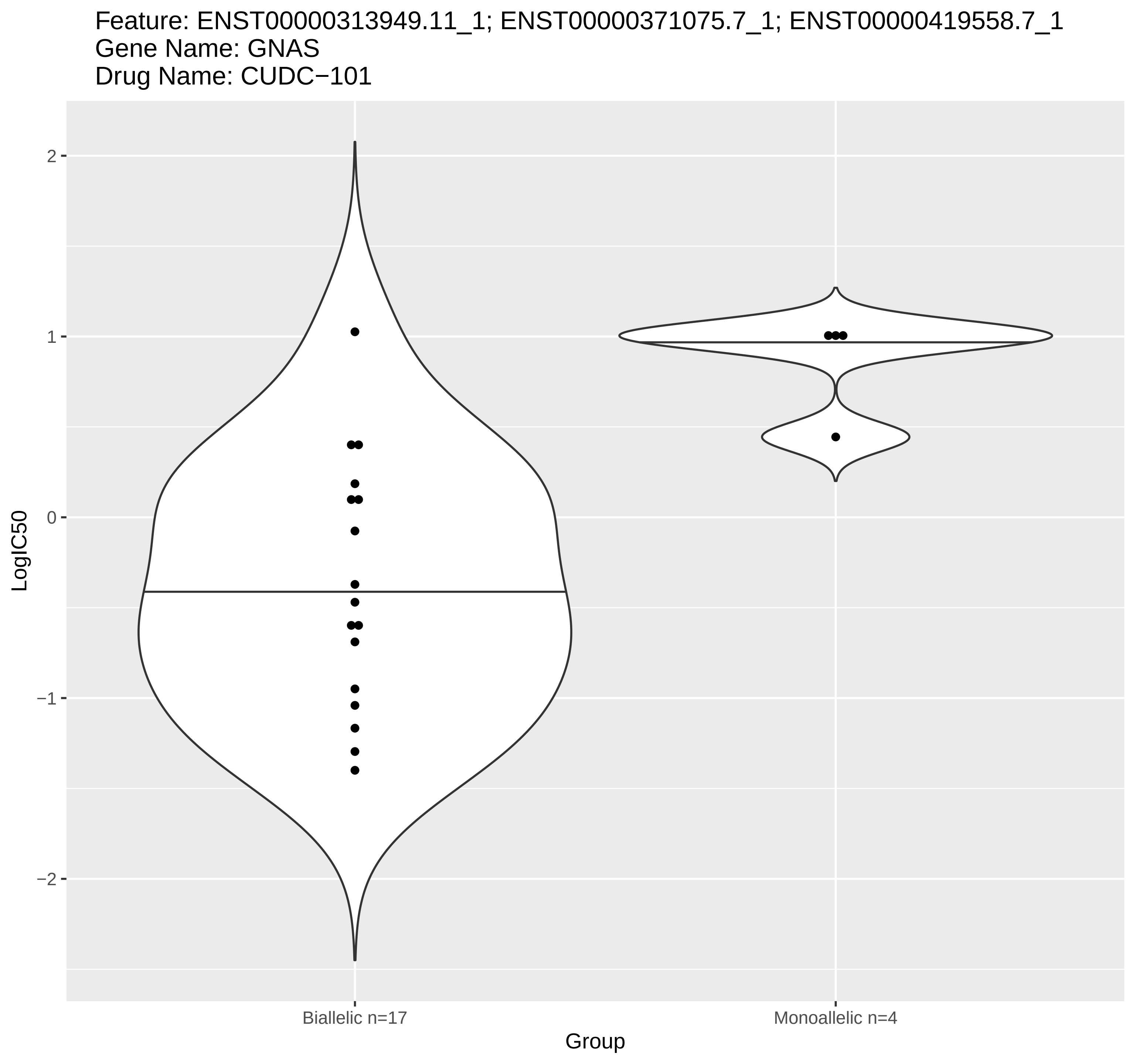

Feature: ENST00000313949.11_1; ENST00000371075.7_1; ENST00000419558.7_1
Gene Name: GNAS
Drug Name: CUDC−101
LogIC50
2
1
0
−1
−2
Biallelic n=17
Monoallelic n=4
Group

Feature: ENST00000682092.1_1; ENST00000682590.1_1; ENST00000682680.1_1
Gene Name: GNAS
Drug Name: resiquimod

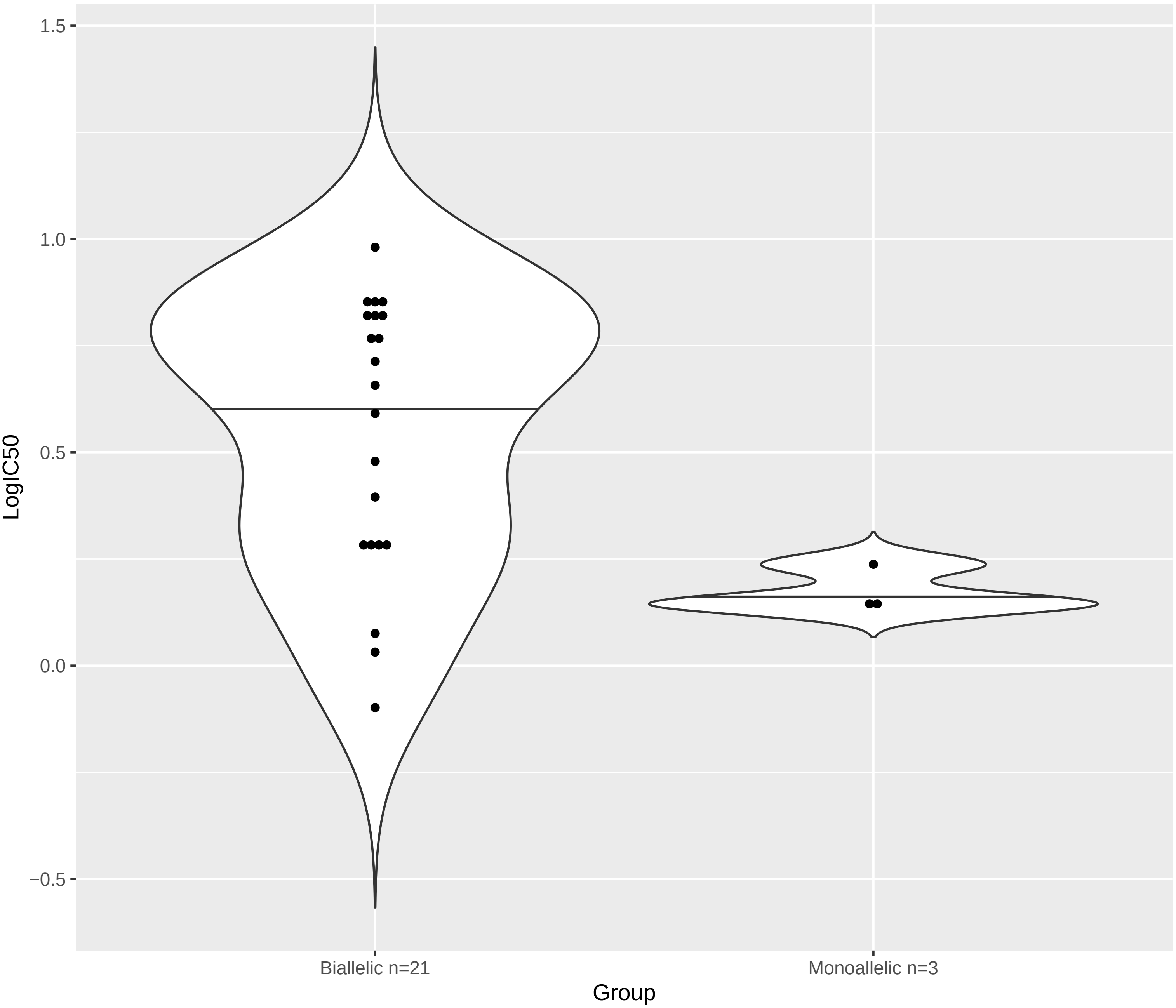

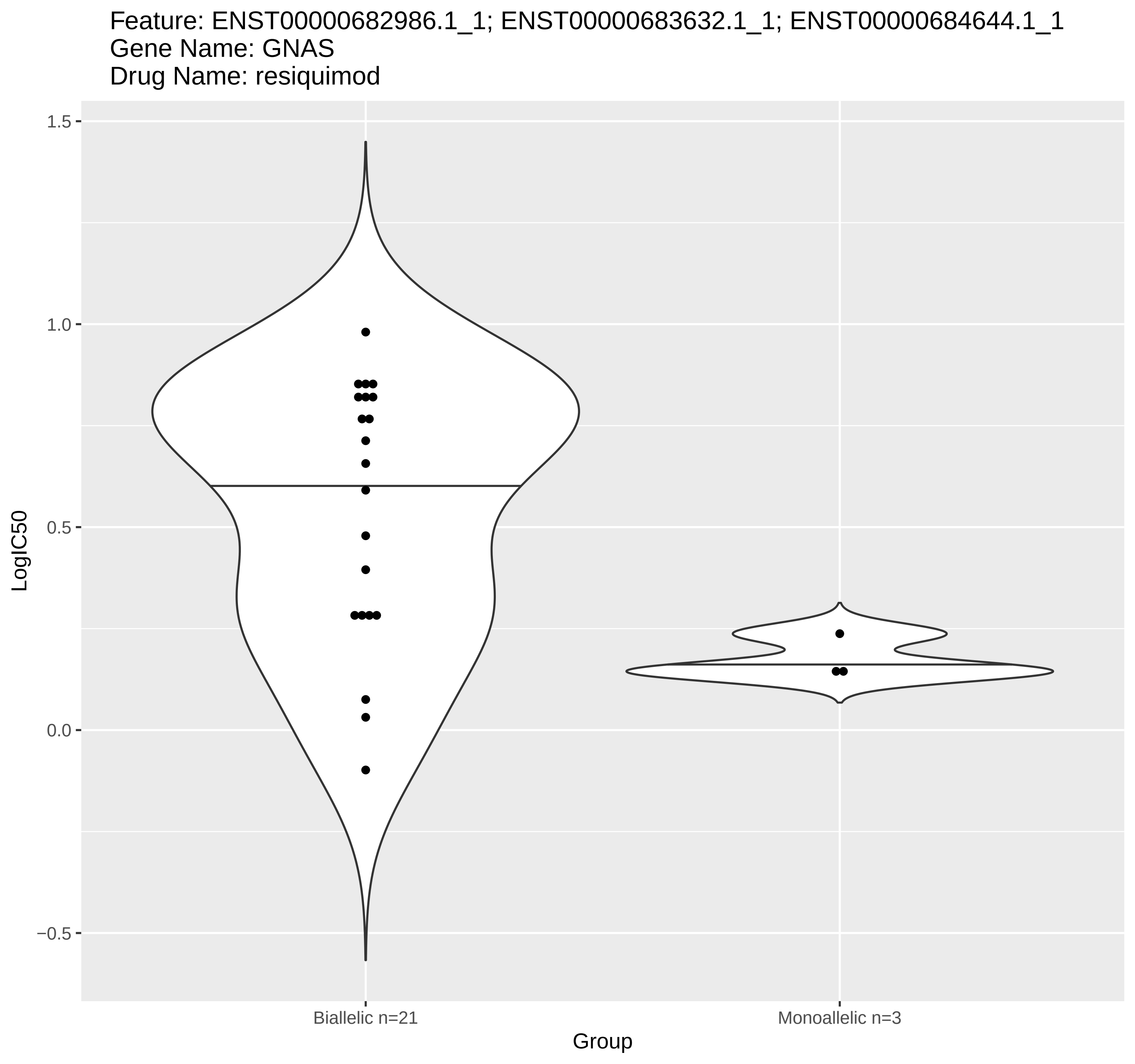
Feature: ENST00000682986.1_1; ENST00000683632.1_1; ENST00000684644.1_1
Gene Name: GNAS
Drug Name: resiquimod
LogIC50
1.5
1.0
0.5
0.0
−0.5
Biallelic n=21
Monoallelic n=3
Group

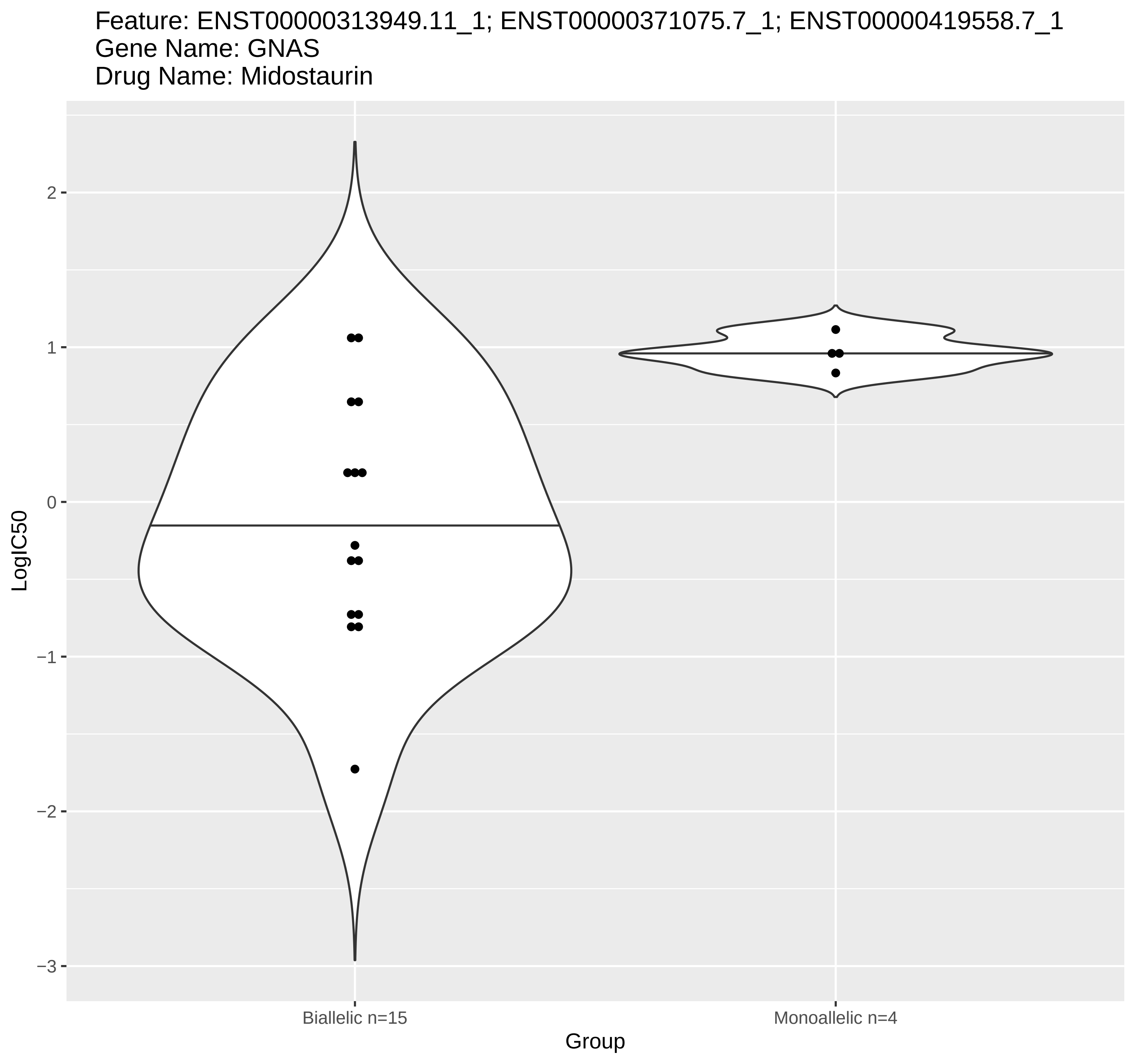
Feature: ENST00000313949.11_1; ENST00000371075.7_1; ENST00000419558.7_1
Gene Name: GNAS
Drug Name: Midostaurin
LogIC50
2
1
0
−1
−2
−3
Biallelic n=15
Monoallelic n=4
Group

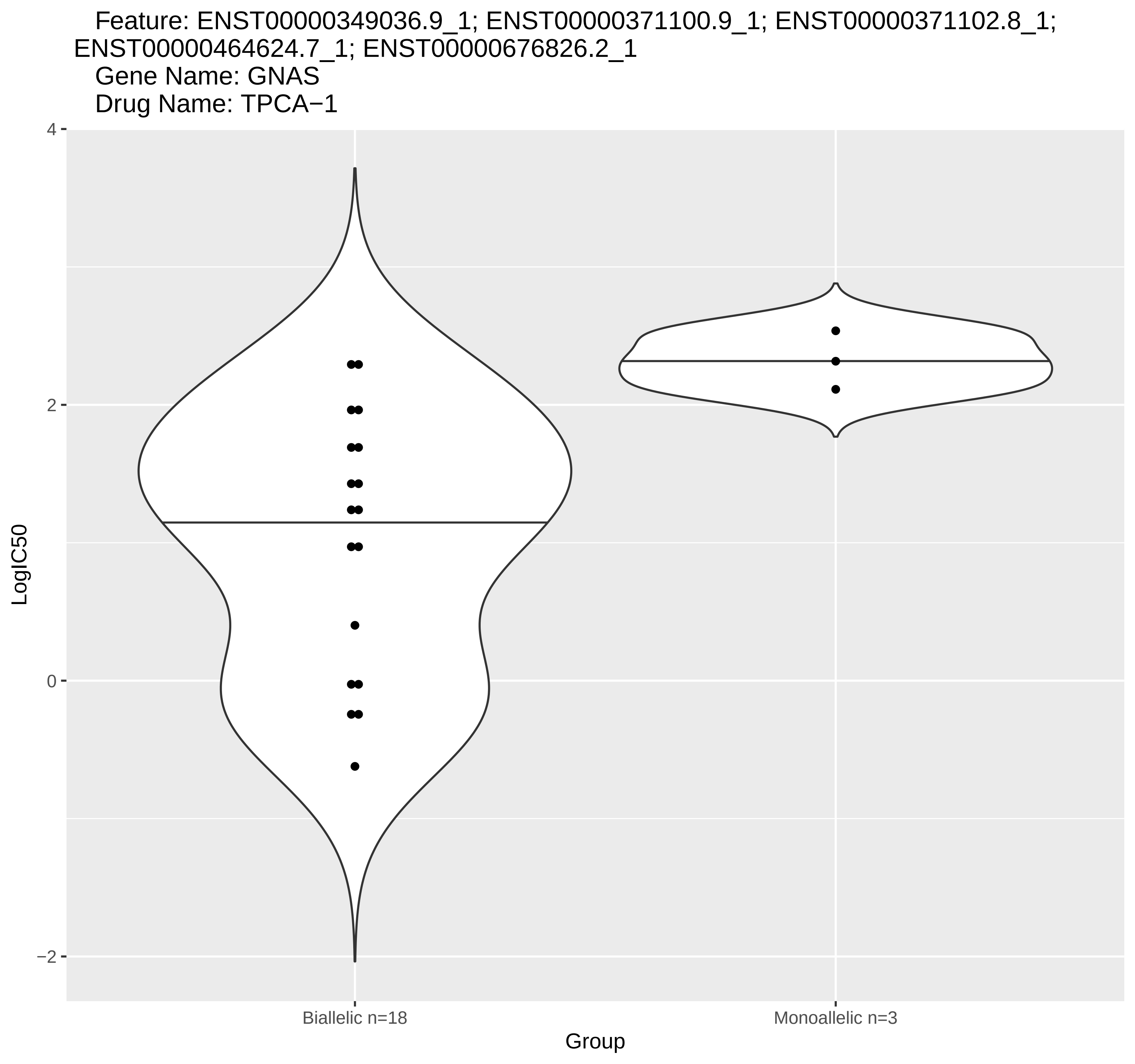

Feature: ENST00000349036.9_1; ENST00000371100.9_1; ENST00000371102.8_1; ENST00000464624.7_1; ENST00000676826.2_1
Gene Name: GNAS
Drug Name: TPCA−1
4
2
0
−2
LogIC50
Biallelic n=18
Monoallelic n=3
Group

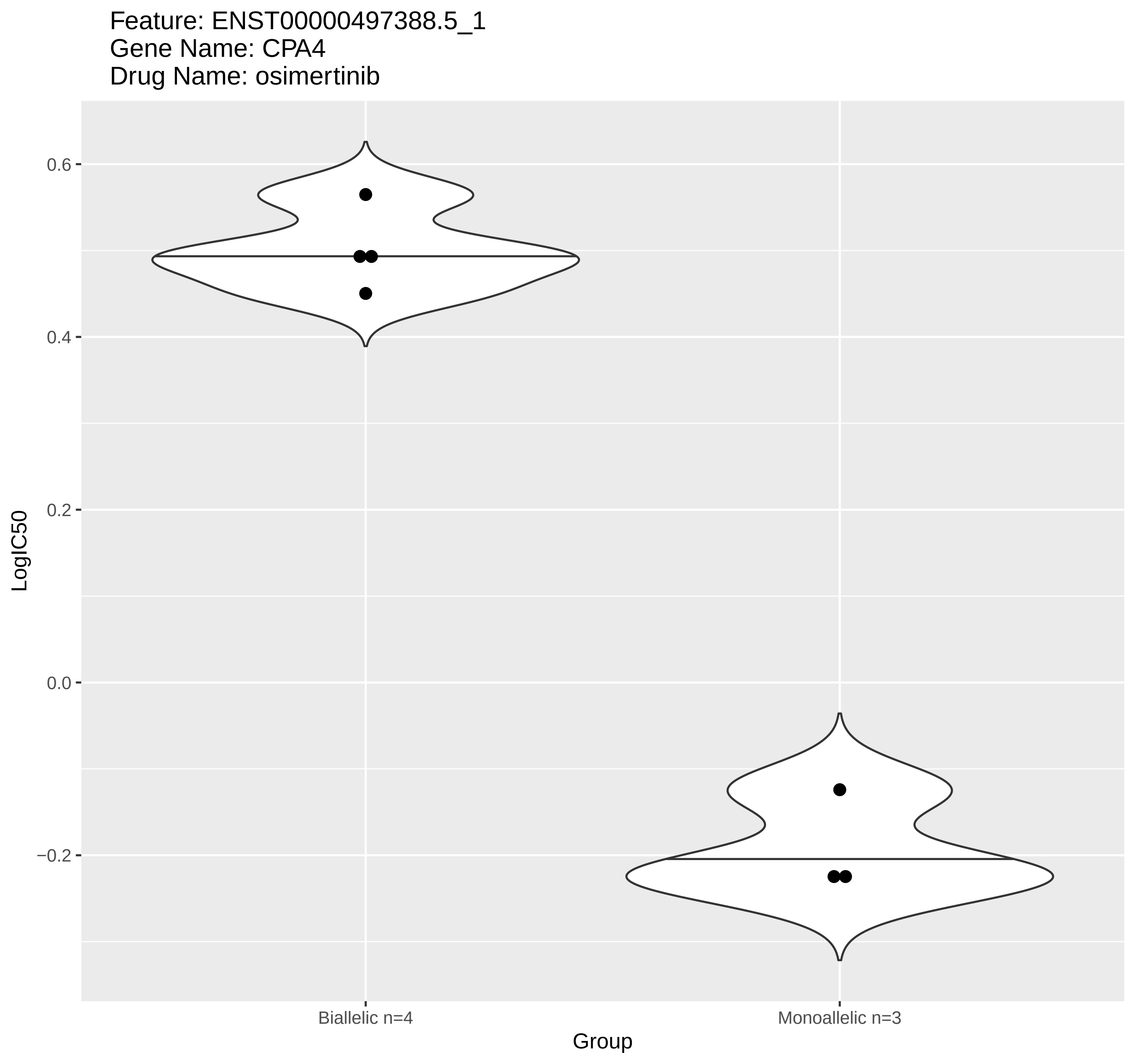

Feature: ENST00000497388.5_1
Gene Name: CPA4
Drug Name: osimertinib
LogIC50
0.6
0.4
0.2
0.0
−0.2
Biallelic n=4
Monoallelic n=3
Group

Feature: ENST00000504493.6_1; ENST00000513999.5_1
Gene Name: ZNF331
Drug Name: tedizolid−phosphate

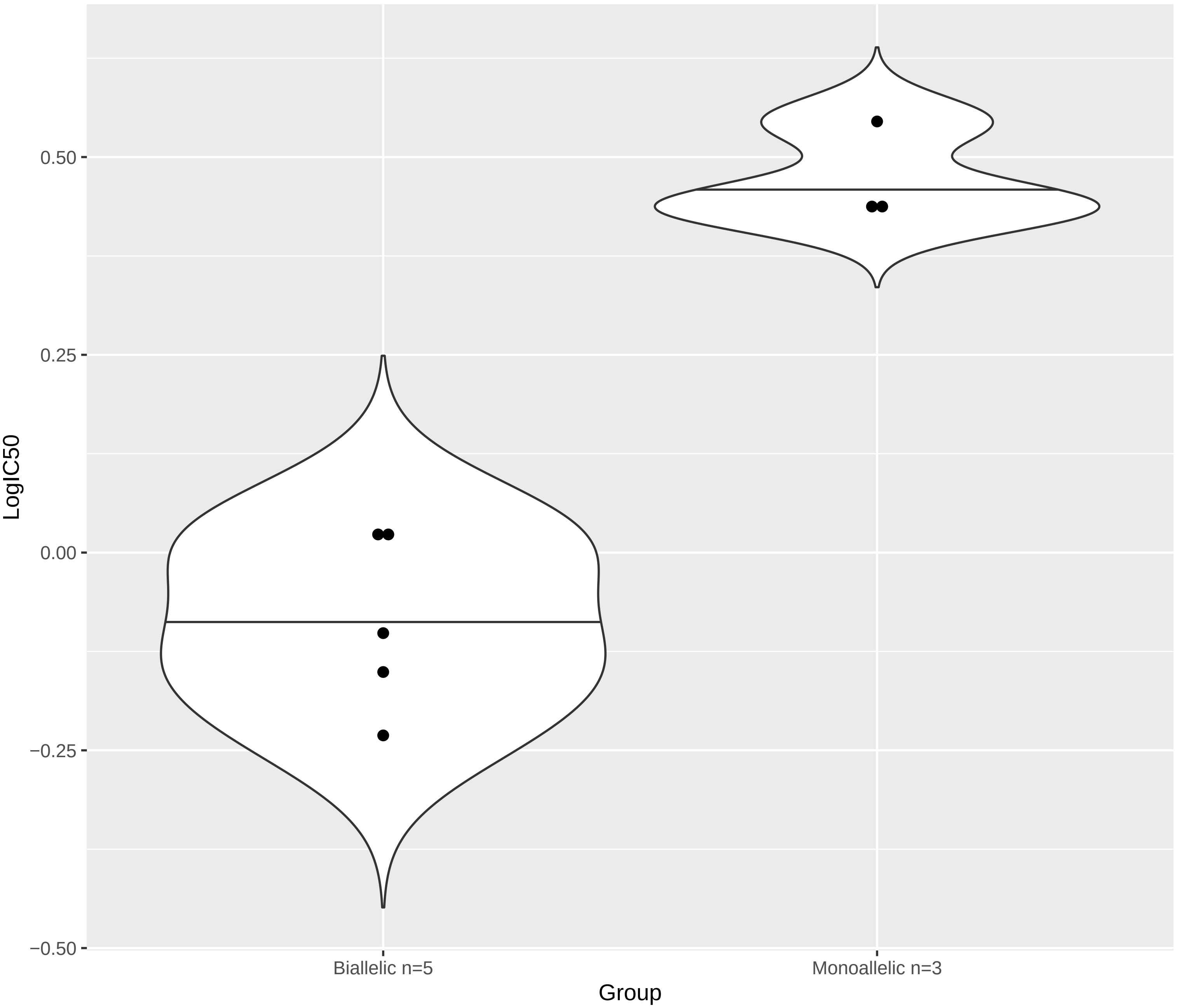

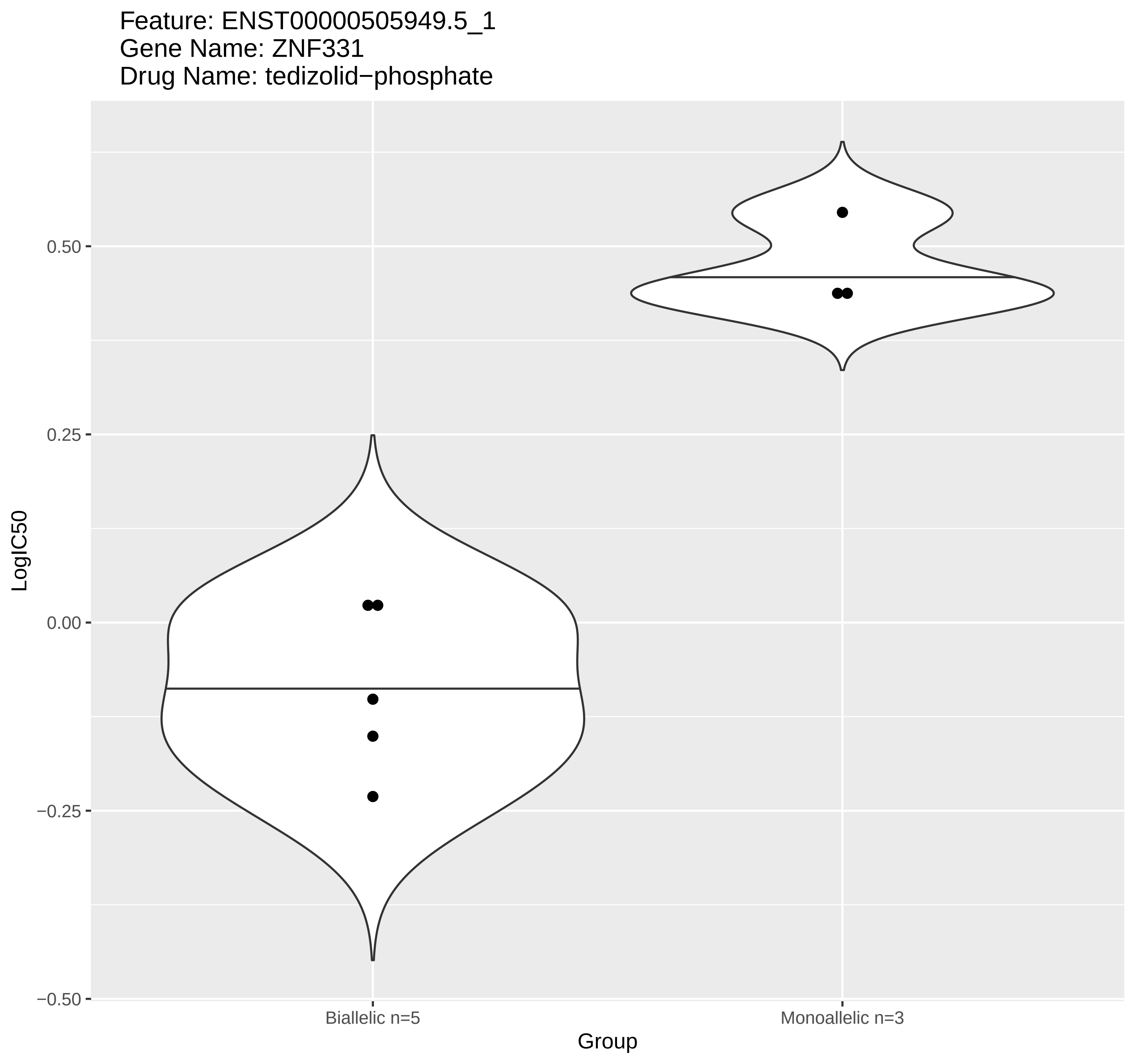
Feature: ENST00000505949.5_1
Gene Name: ZNF331
Drug Name: tedizolid−phosphate
LogIC50
0.50
0.25
0.00
−0.25
−0.50
Biallelic n=5
Monoallelic n=3
Group

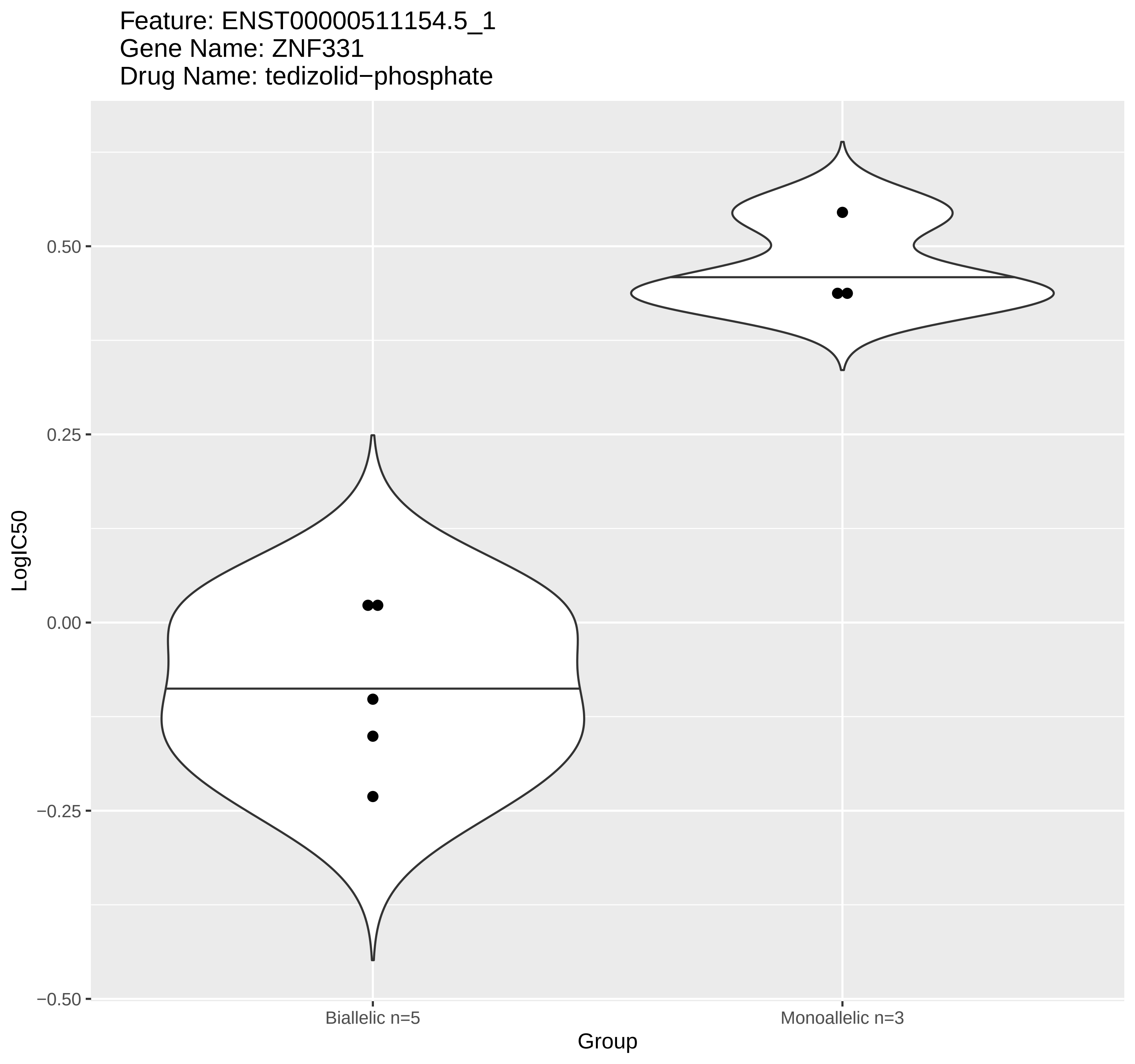
Feature: ENST00000511154.5_1
Gene Name: ZNF331
Drug Name: tedizolid−phosphate
0.50
0.25
0.00
−0.25
−0.50
LogIC50
Biallelic n=5
Monoallelic n=3
Group

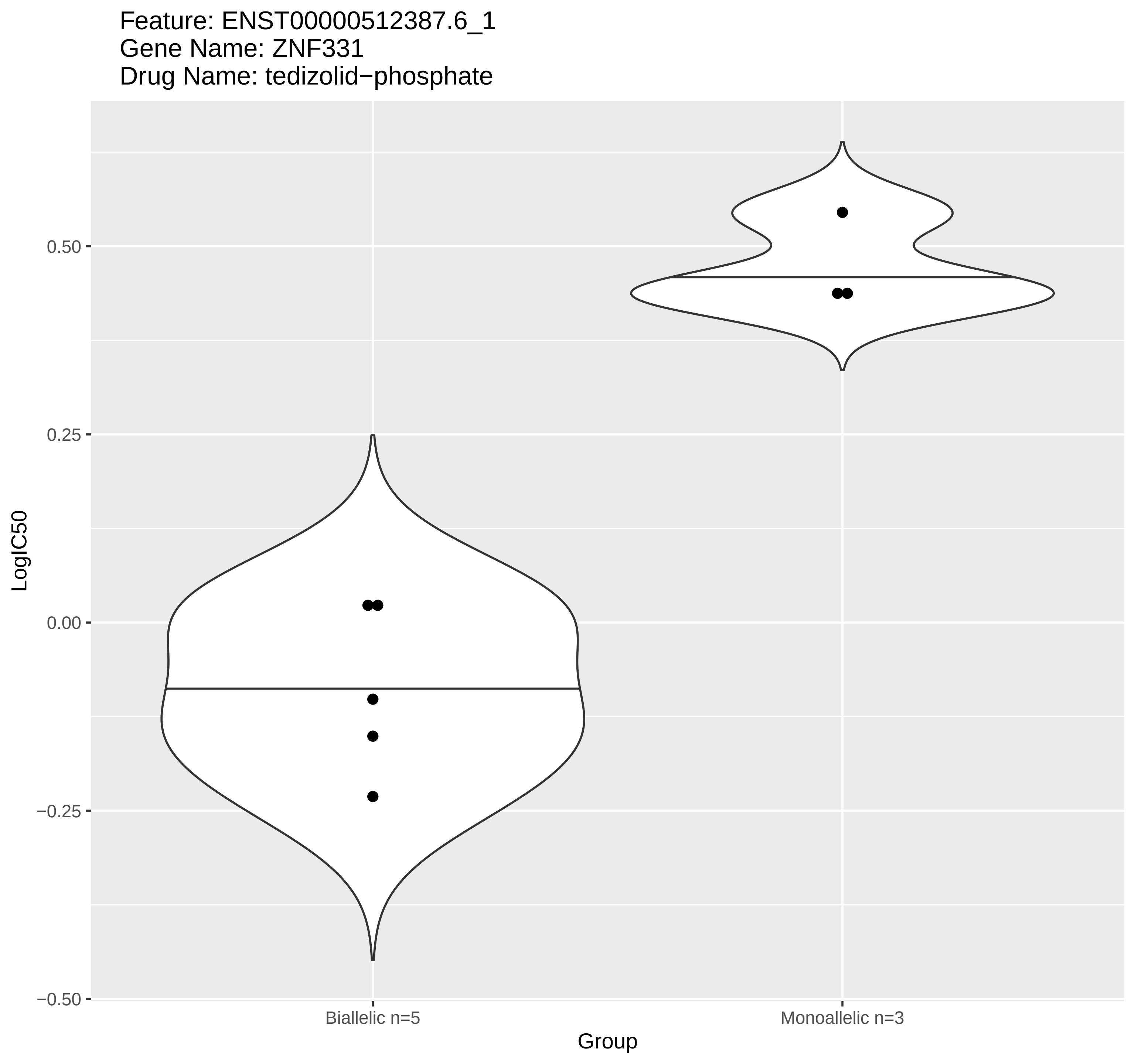

Feature: ENST00000512387.6_1
Gene Name: ZNF331
Drug Name: tedizolid−phosphate
LogIC50
0.50
0.25
0.00
−0.25
−0.50
Biallelic n=5
Monoallelic n=3
Group

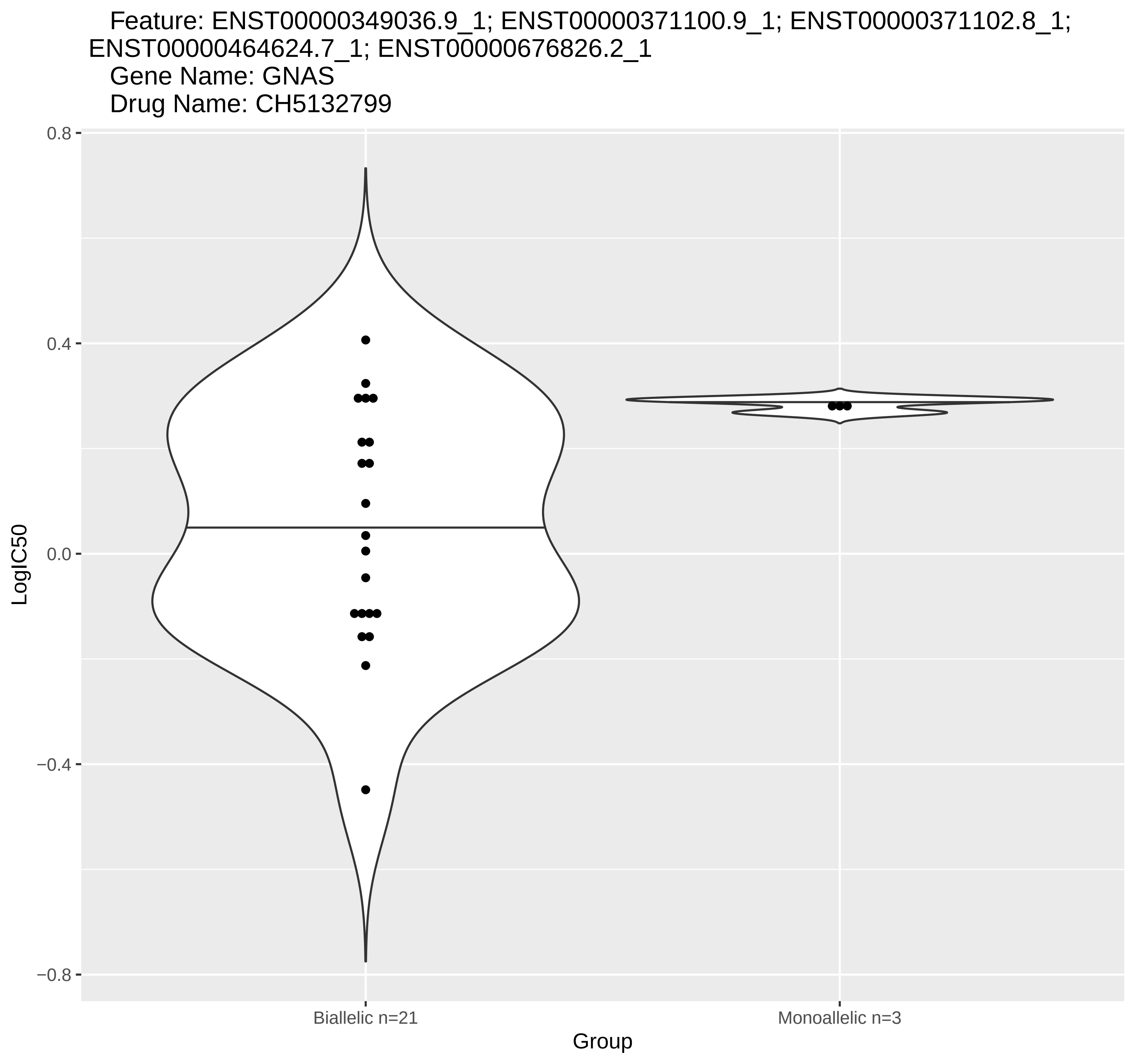
Feature: ENST00000349036.9_1; ENST00000371100.9_1; ENST00000371102.8_1; ENST00000464624.7_1; ENST00000676826.2_1
Gene Name: GNAS
Drug Name: CH5132799
0.8
0.4
0.0
−0.4
−0.8
LogIC50
Biallelic n=21
Monoallelic n=3
Group

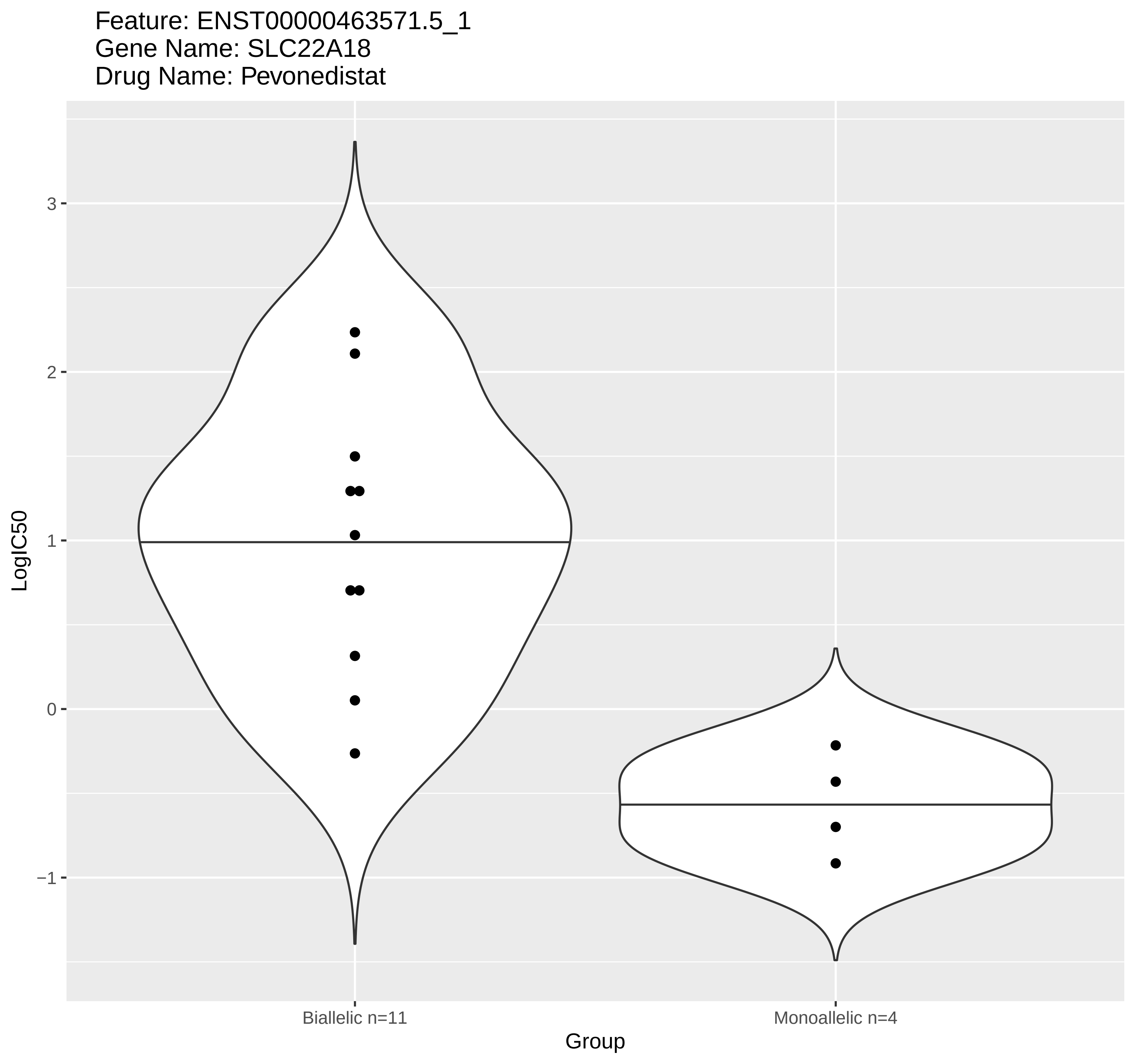
Feature: ENST00000463571.5_1
Gene Name: SLC22A18
Drug Name: Pevonedistat
LogIC50
3
2
1
0
−1
Biallelic n=11
Monoallelic n=4
Group

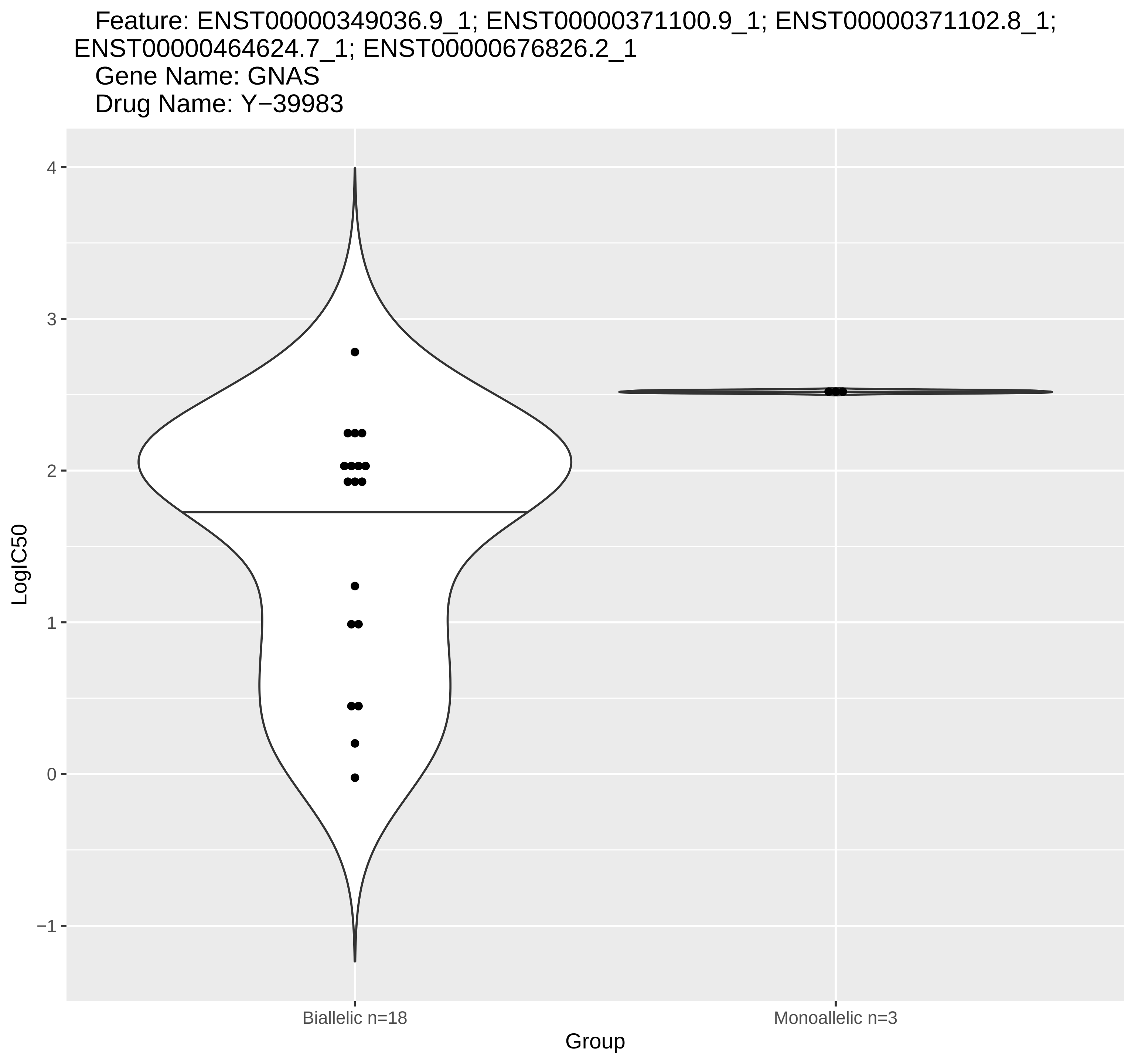

Feature: ENST00000349036.9_1; ENST00000371100.9_1; ENST00000371102.8_1; ENST00000464624.7_1; ENST00000676826.2_1
Gene Name: GNAS
Drug Name: Y−39983
4
3
2
1
0
−1
LogIC50
Biallelic n=18
Monoallelic n=3
Group

Feature: ENST00000523399.5_1
Gene Name: ZFAT
Drug Name: POMHEX

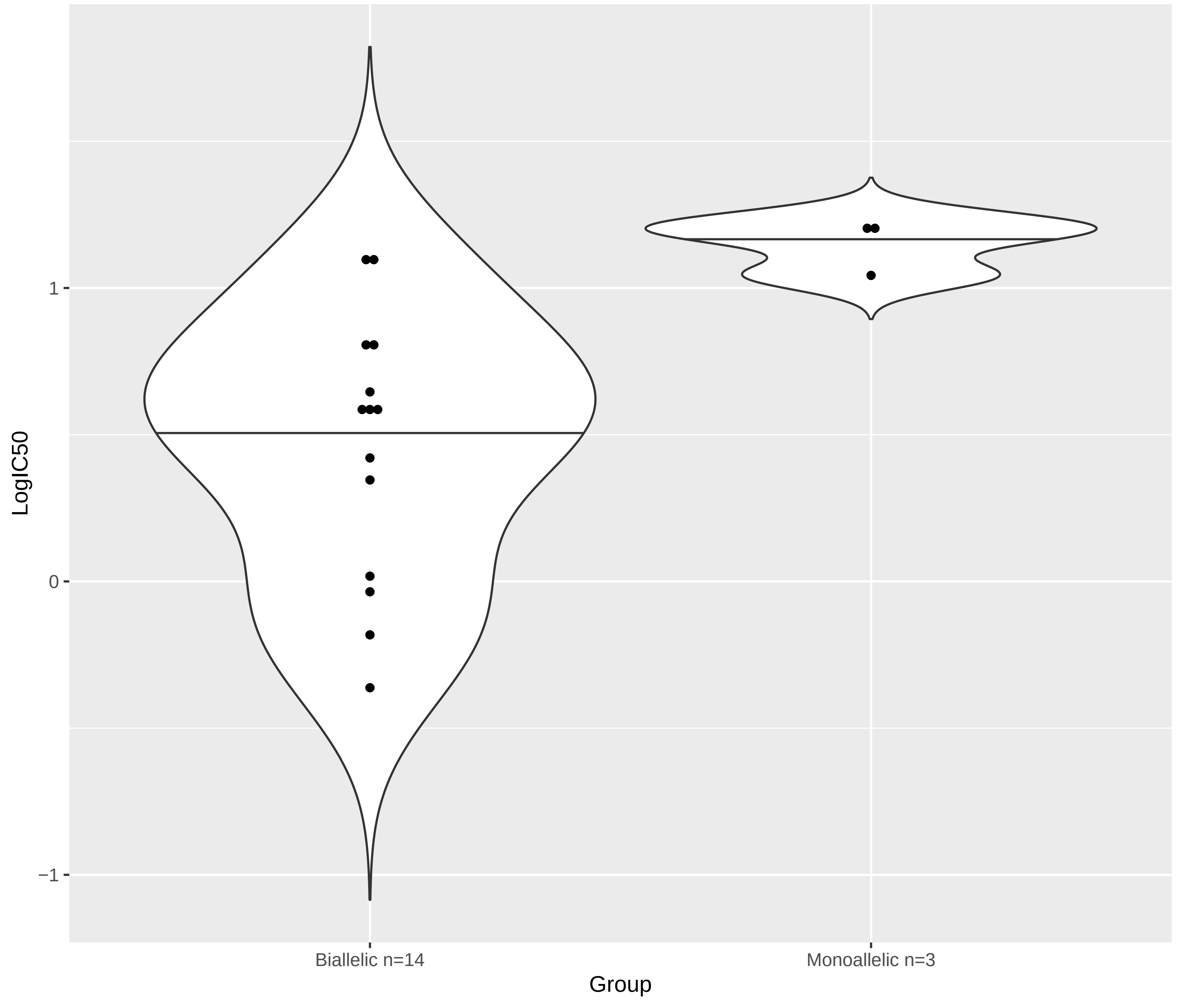

Feature: ENST00000349036.9_1; ENST00000371100.9_1; ENST00000371102.8_1; ENST00000464624.7_1; ENST00000676826.2_1
Gene Name: GNAS
Drug Name: 5−Fluorouracil

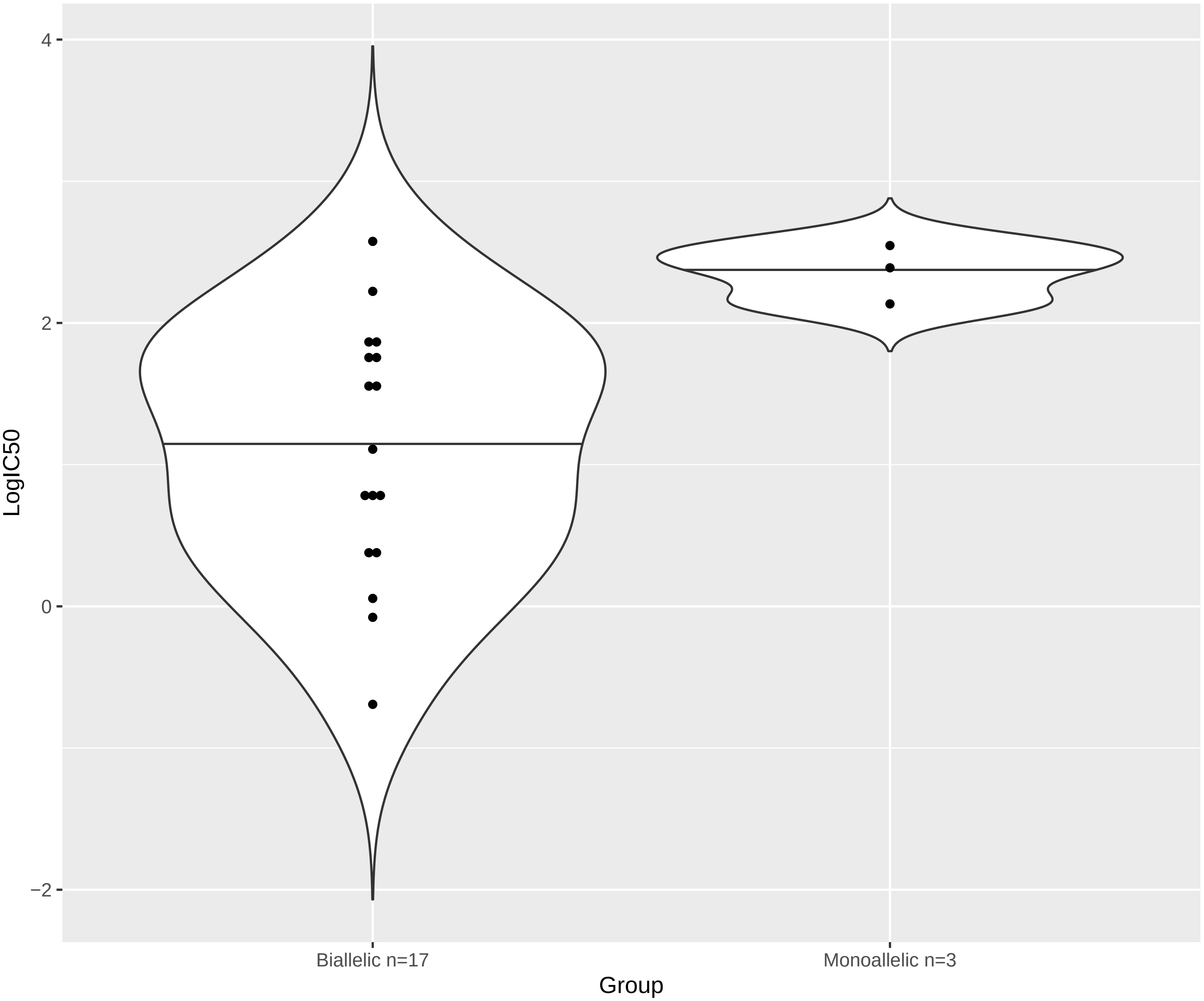

Feature: ENST00000523399.5_1
Gene Name: ZFAT
Drug Name: Niraparib

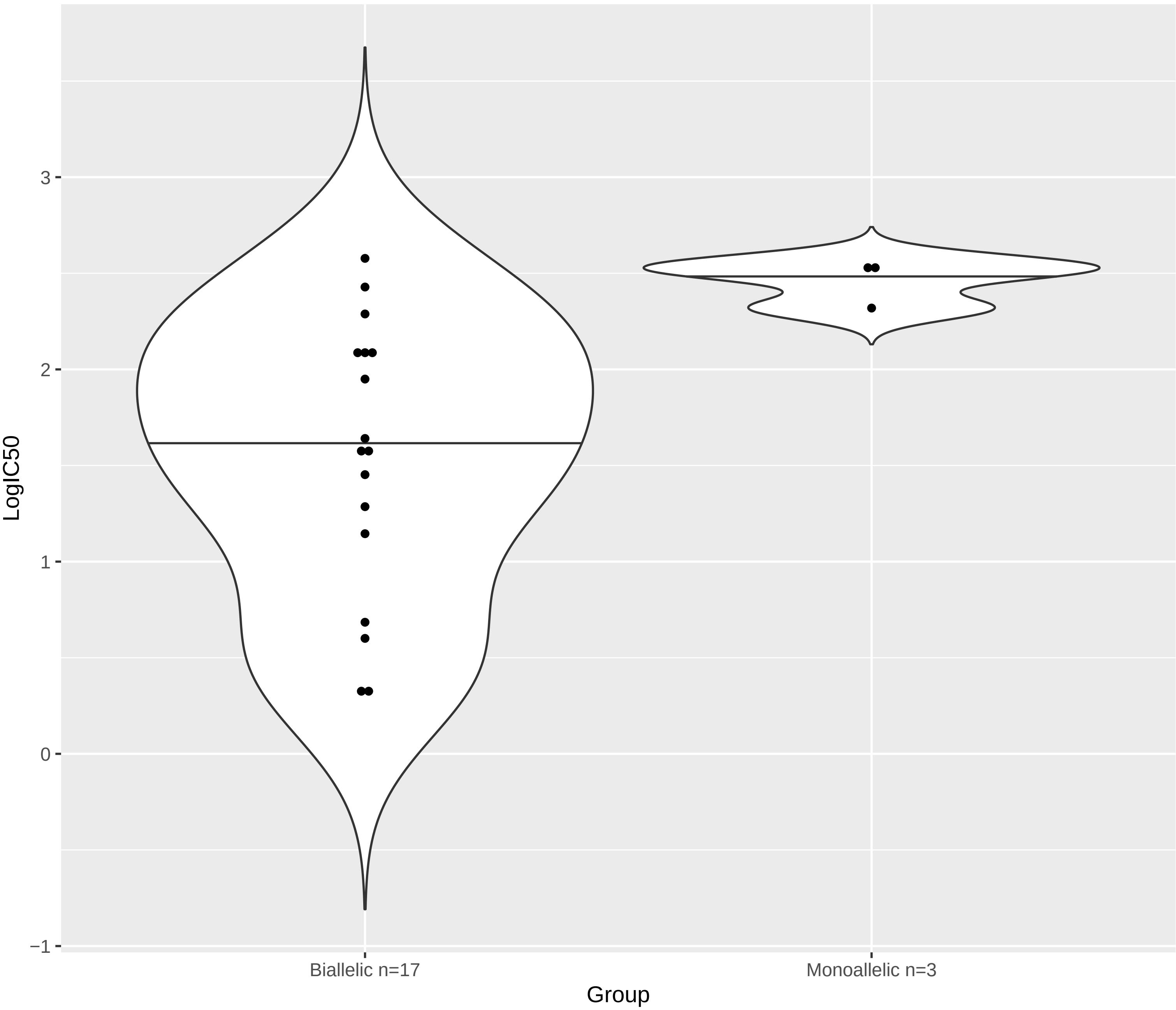

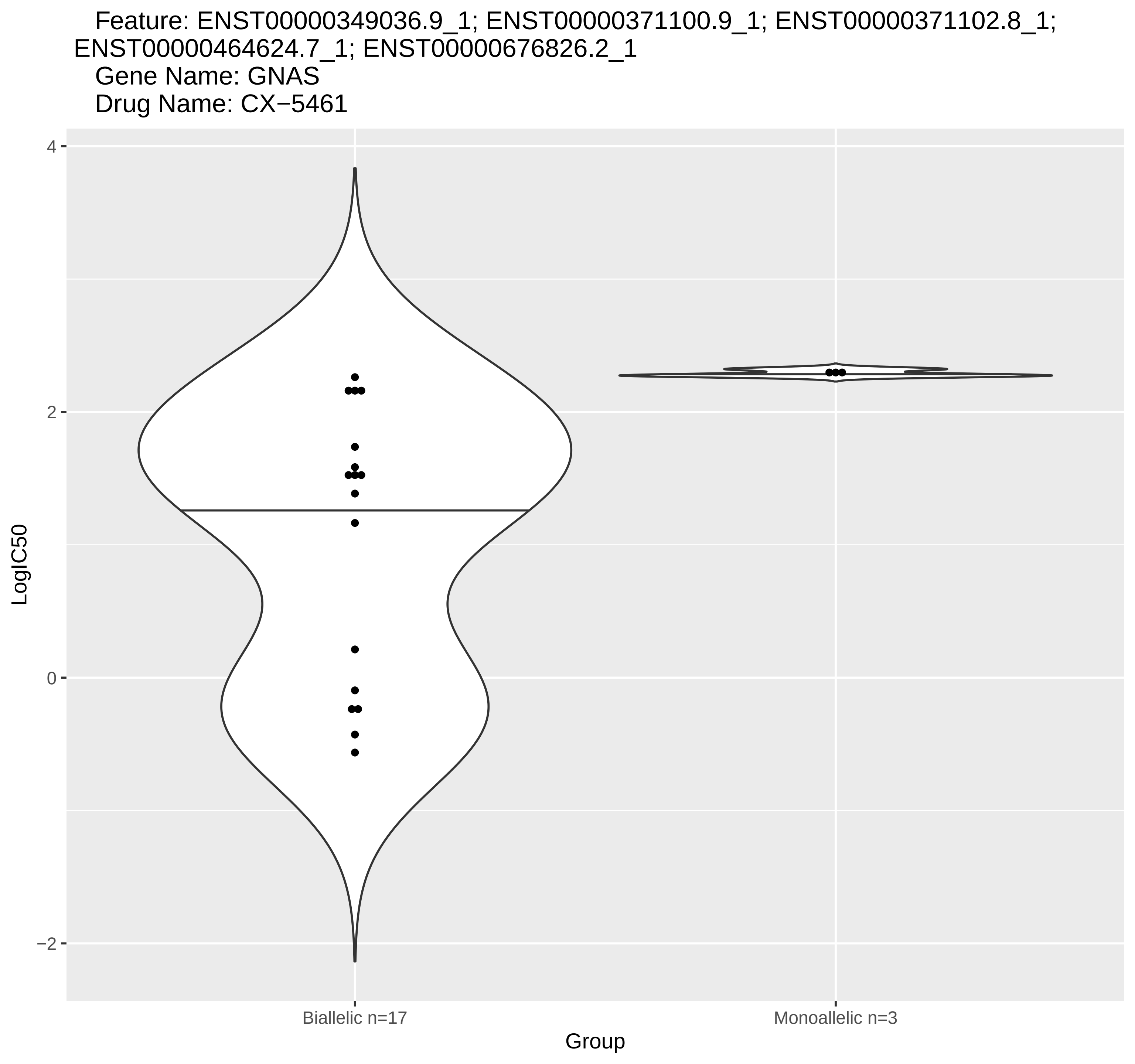

Feature: ENST00000349036.9_1; ENST00000371100.9_1; ENST00000371102.8_1; ENST00000464624.7_1; ENST00000676826.2_1
Gene Name: GNAS
Drug Name: CX−5461
LogIC50
4
2
0
−2
Biallelic n=17
Monoallelic n=3
Group

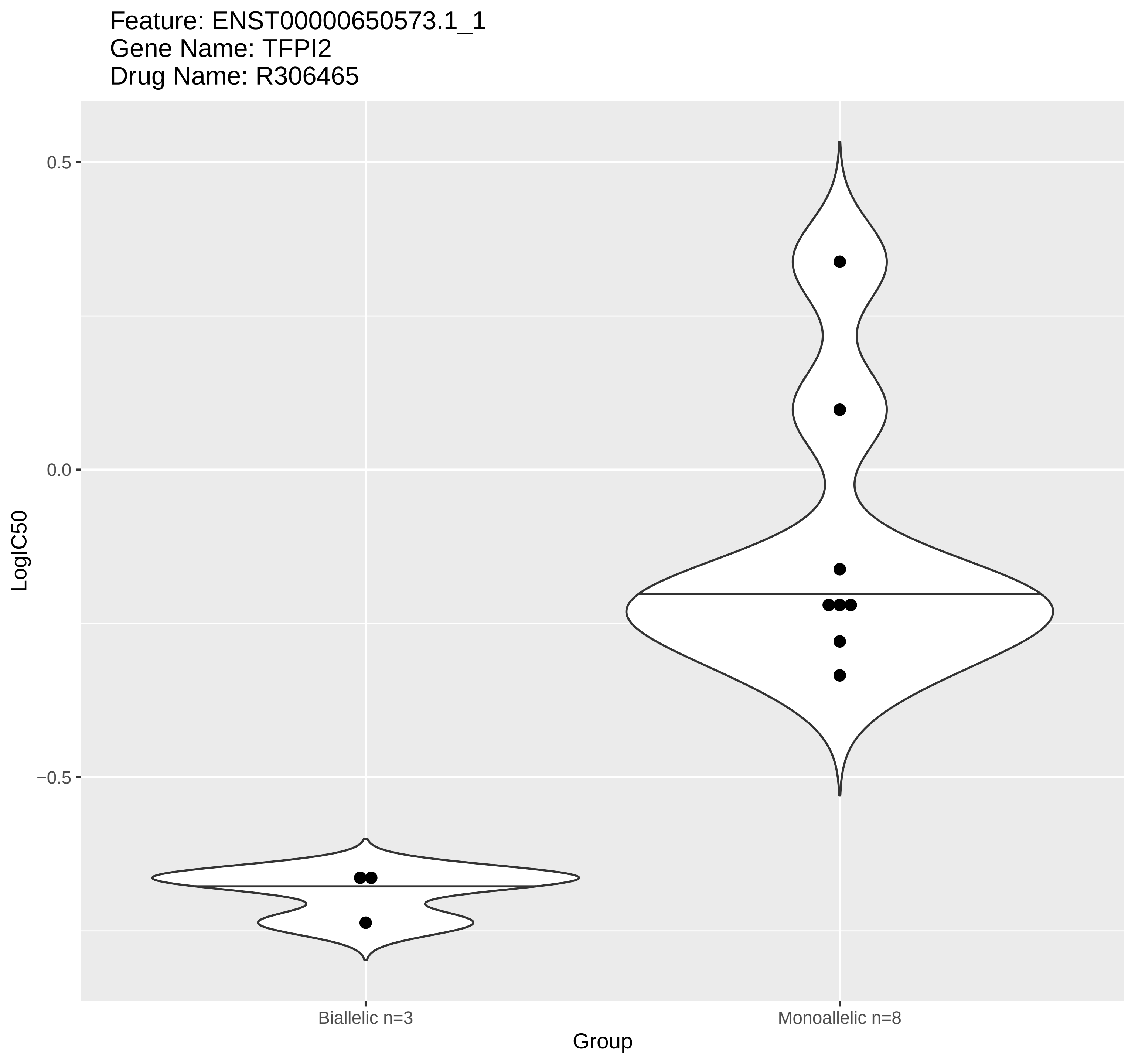

Feature: ENST00000650573.1_1
Gene Name: TFPI2
Drug Name: R306465
LogIC50
0.5
0.0
−0.5
Biallelic n=3
Monoallelic n=8
Group

Feature: ENST00000349036.9_1; ENST00000371100.9_1; ENST00000371102.8_1; ENST00000464624.7_1; ENST00000676826.2_1
Gene Name: GNAS
Drug Name: BIX02189

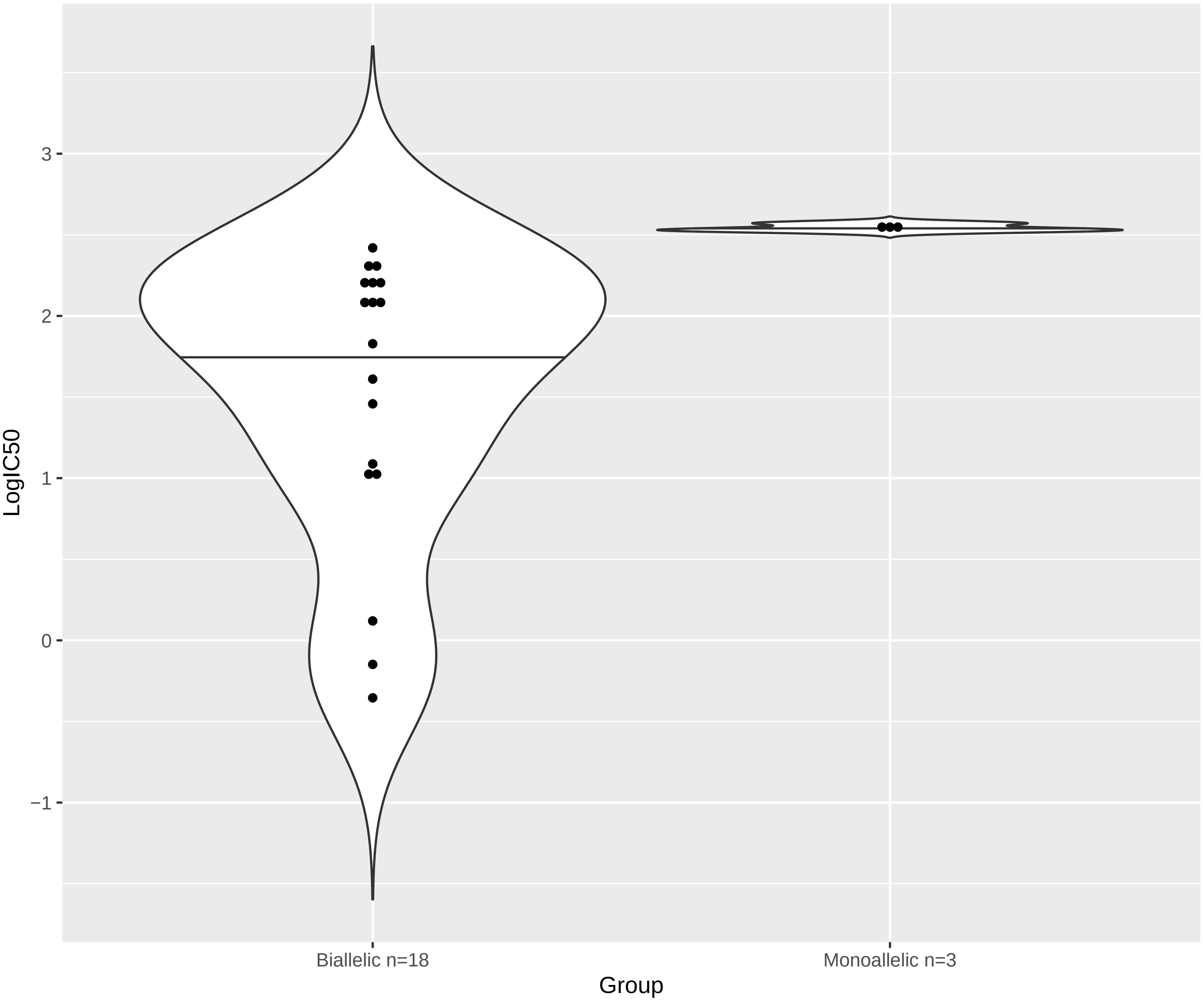

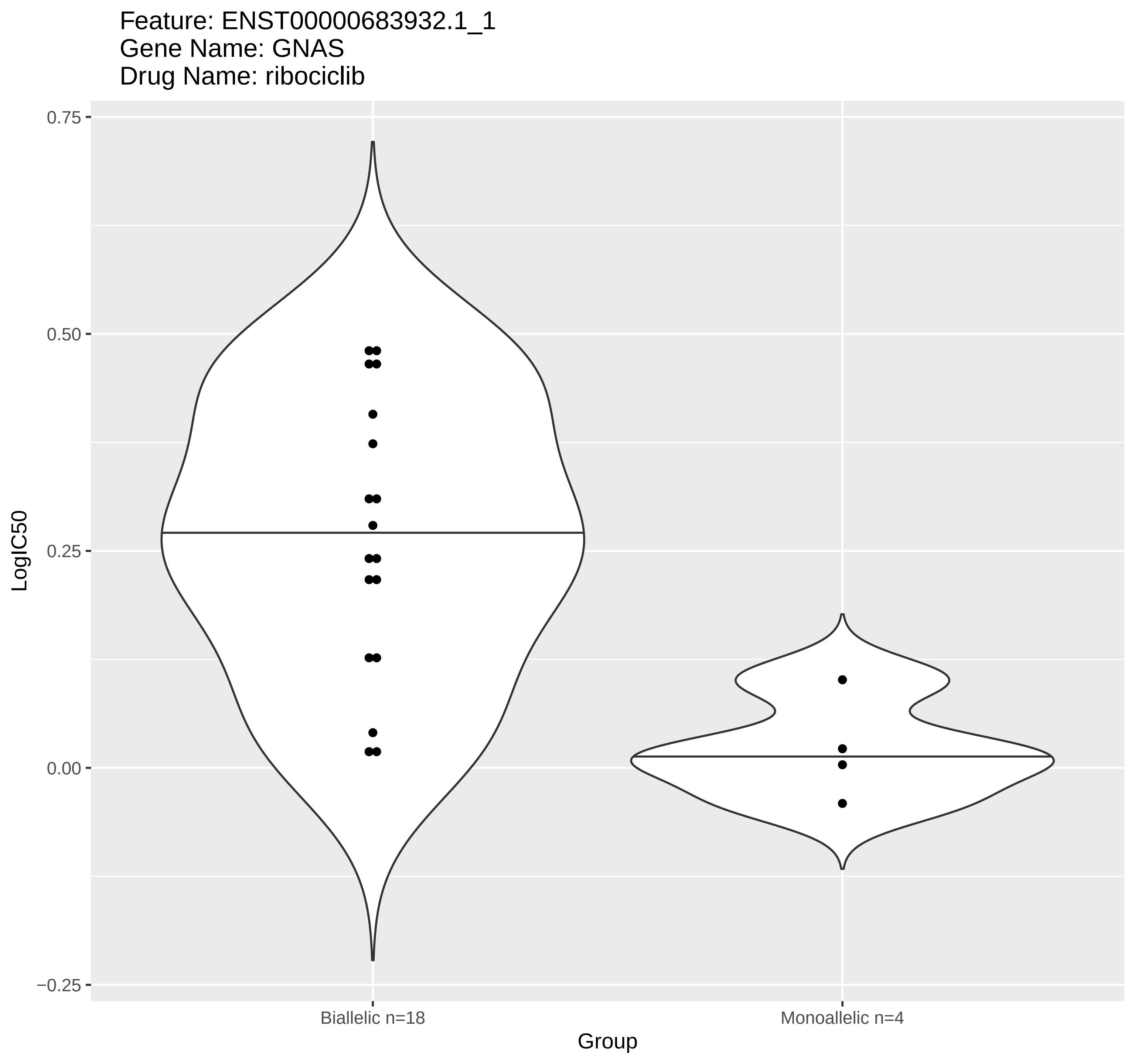
Feature: ENST00000683932.1_1
Gene Name: GNAS
Drug Name: ribociclib
0.75
0.50
0.25
0.00
−0.25
LogIC50
Biallelic n=18
Monoallelic n=4
Group

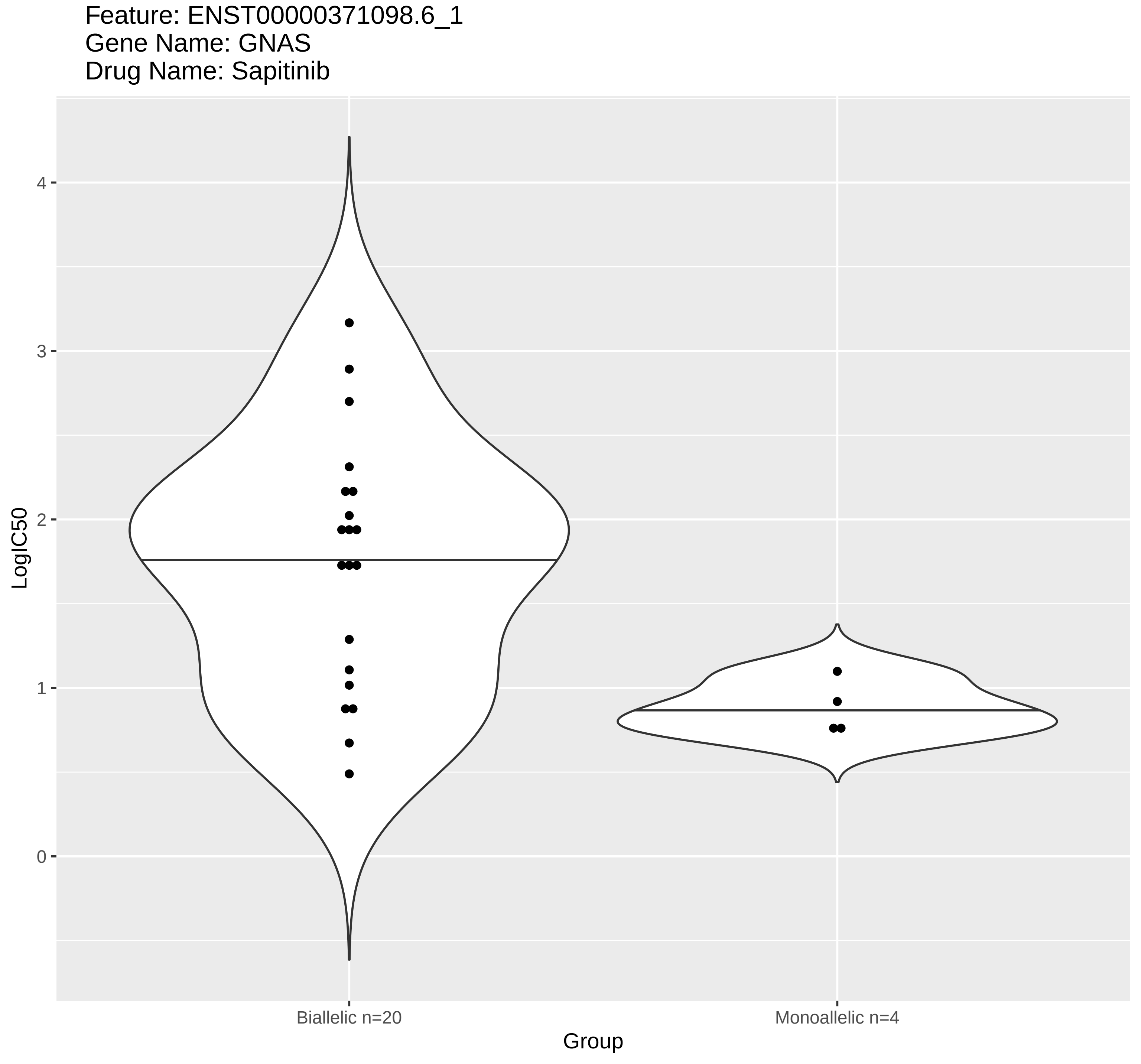

Feature: ENST00000371098.6_1
Gene Name: GNAS
Drug Name: Sapitinib
LogIC50
4
3
2
1
0
Biallelic n=20
Monoallelic n=4
Group

Feature: ENST00000349036.9_1; ENST00000371100.9_1; ENST00000371102.8_1; ENST00000464624.7_1; ENST00000676826.2_1
Gene Name: GNAS
Drug Name: KIN001−260

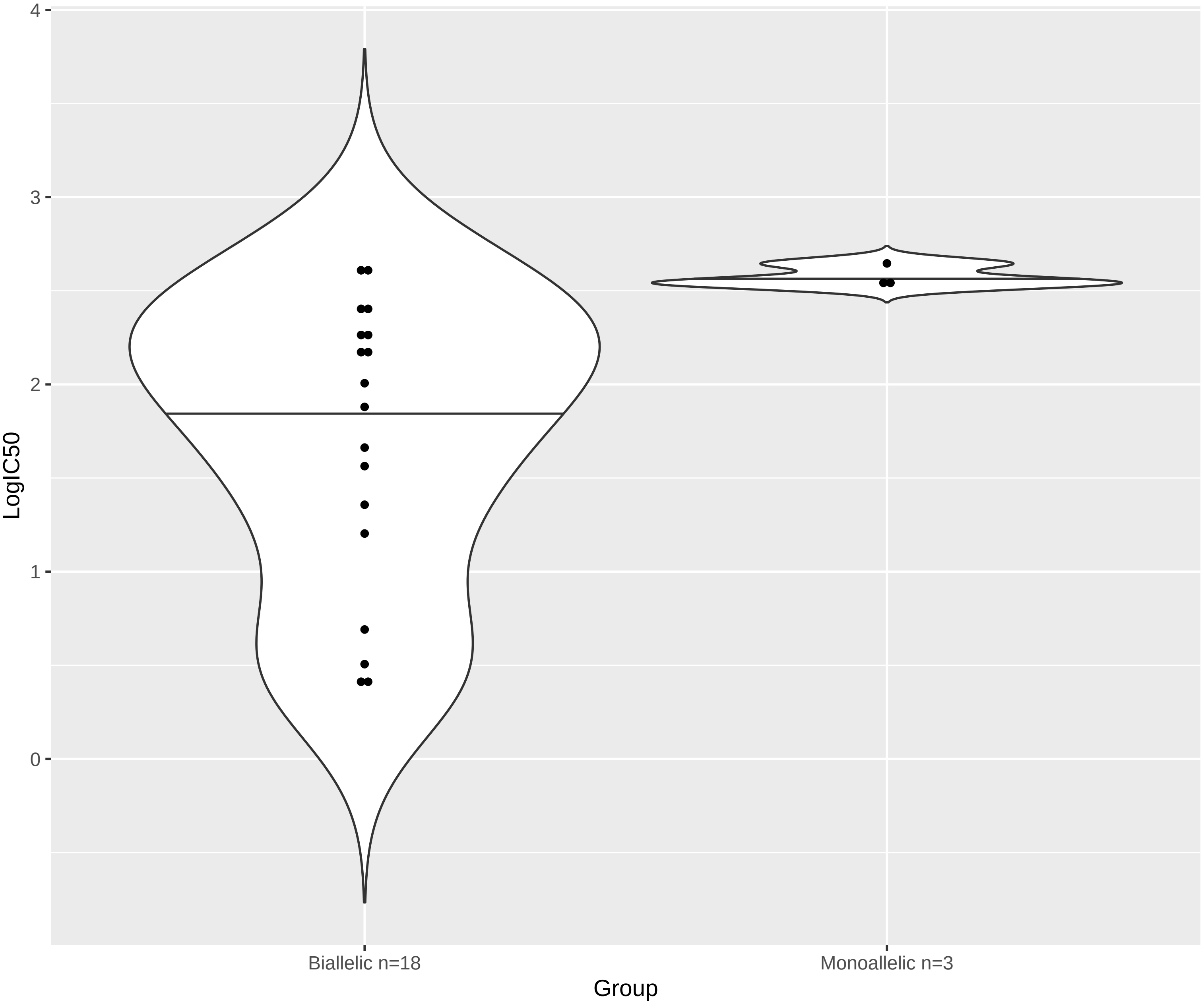

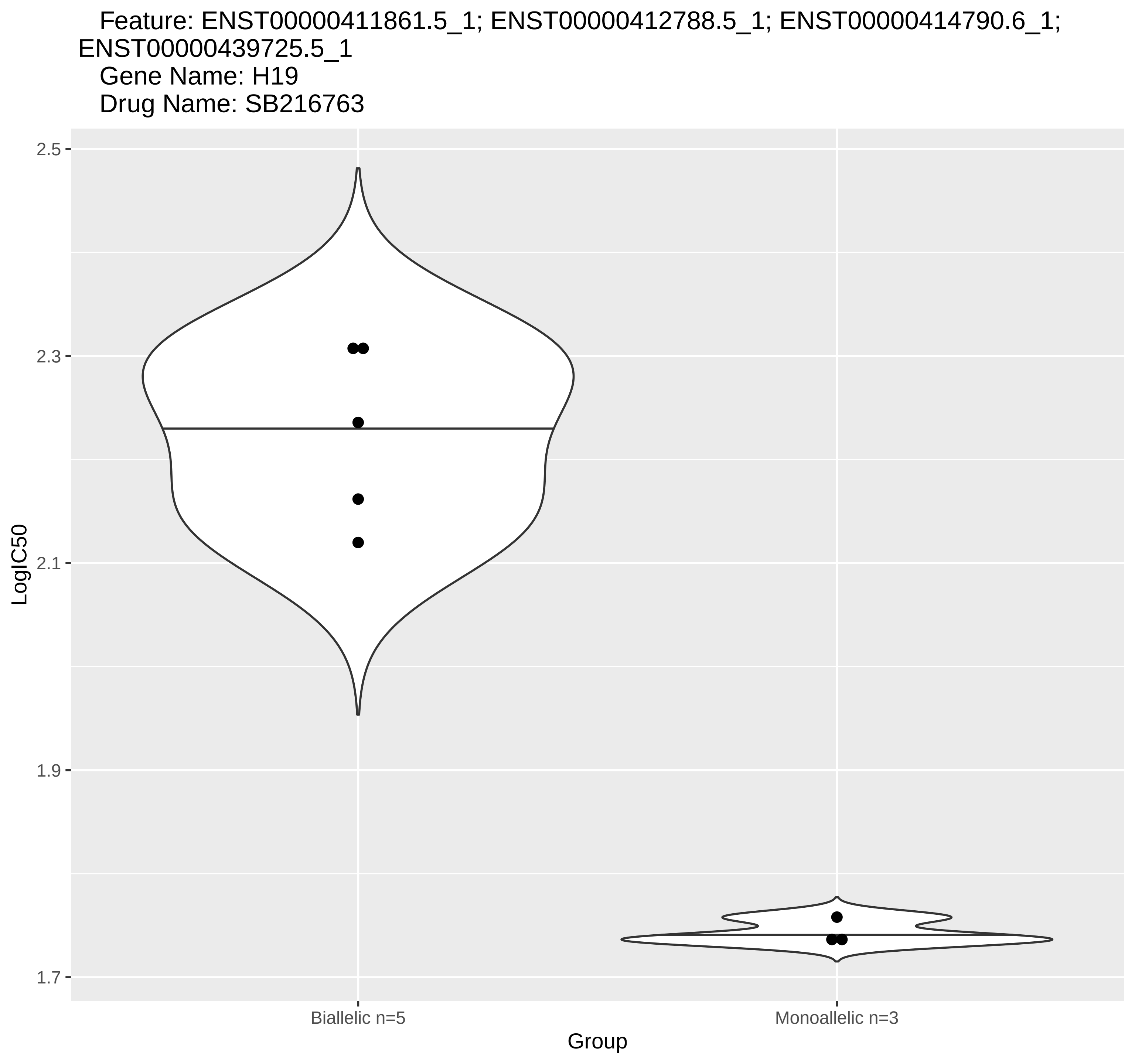

Feature: ENST00000411861.5_1; ENST00000412788.5_1; ENST00000414790.6_1; ENST00000439725.5_1
Gene Name: H19
Drug Name: SB216763
LogIC50
2.5
2.3
2.1
1.9
1.7
Biallelic n=5
Monoallelic n=3
Group

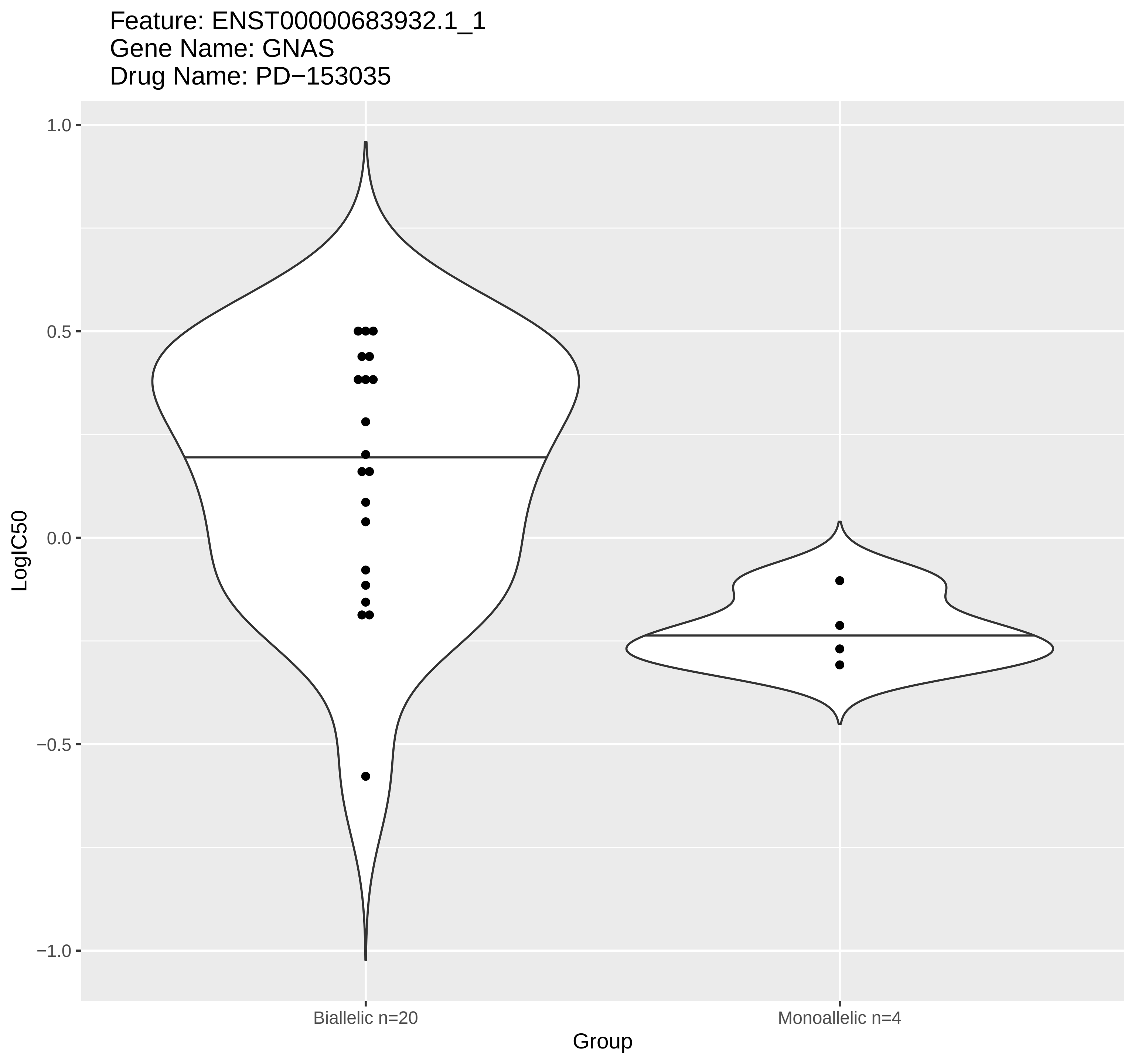

Feature: ENST00000683932.1_1
Gene Name: GNAS
Drug Name: PD−153035
1.0
0.5
0.0
−0.5
−1.0
LogIC50
Biallelic n=20
Monoallelic n=4
Group

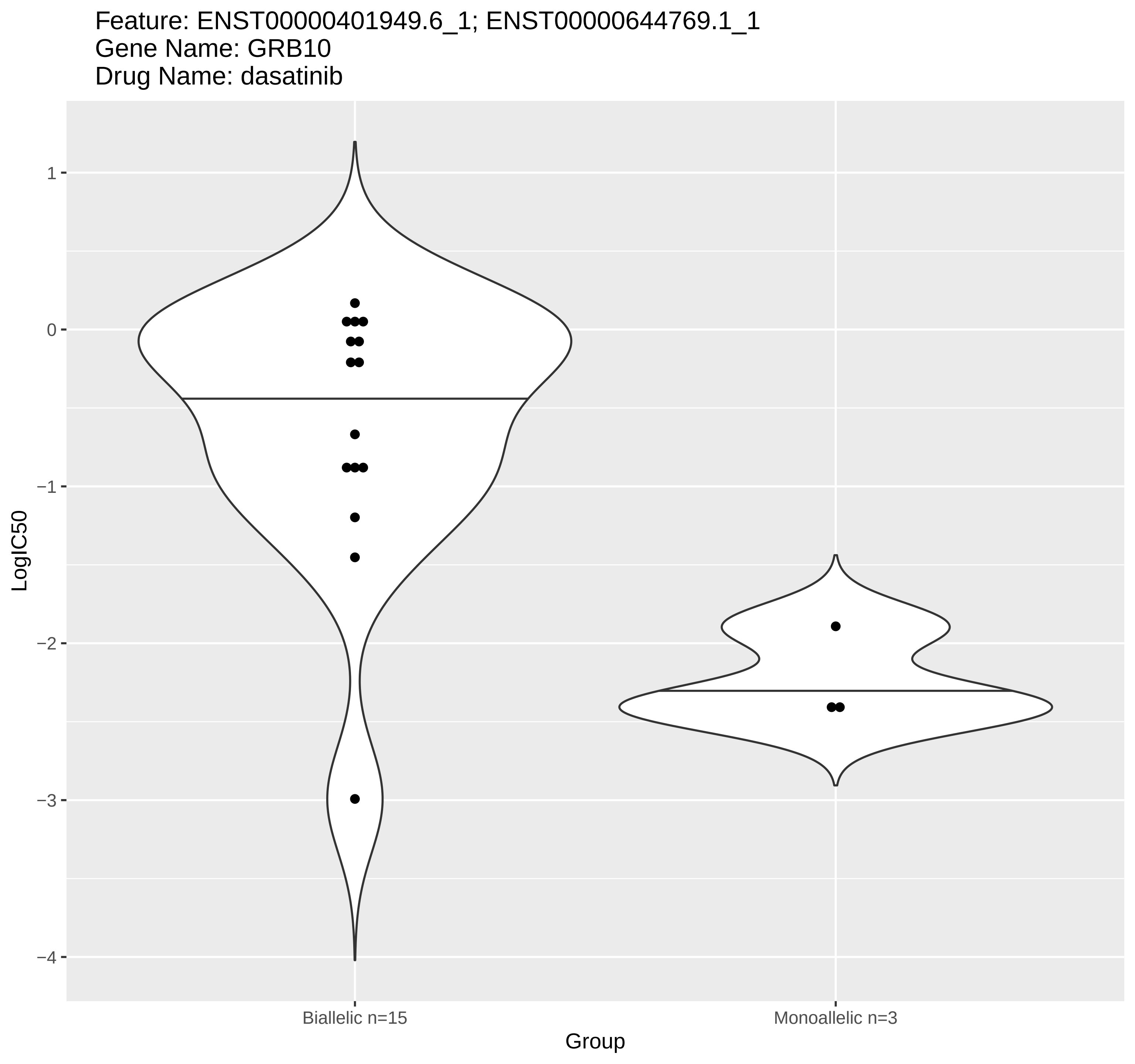
Feature: ENST00000401949.6_1; ENST00000644769.1_1
Gene Name: GRB10
Drug Name: dasatinib
LogIC50
1
0
−1
−2
−3
−4
Biallelic n=15
Monoallelic n=3
Group

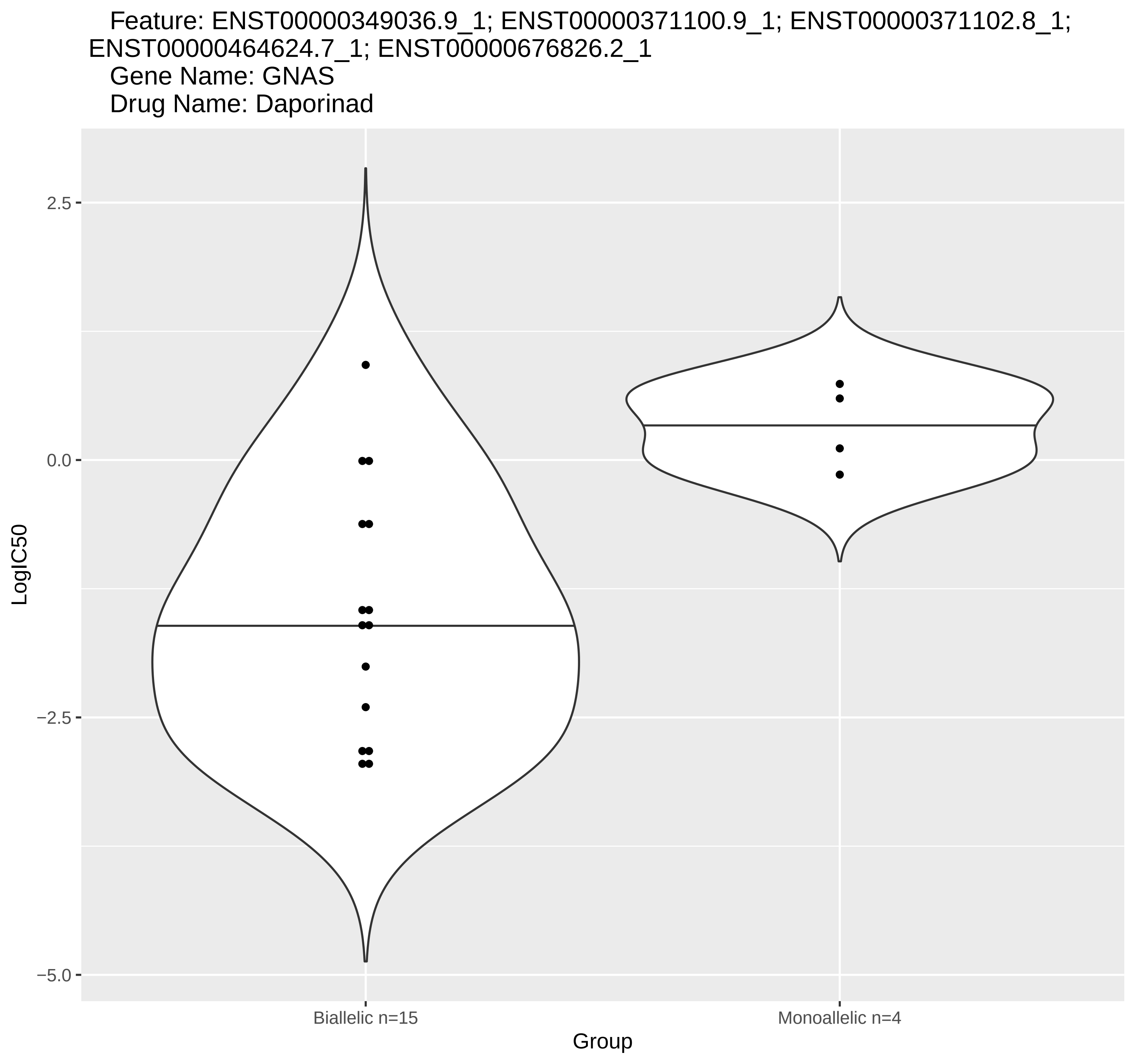

Feature: ENST00000349036.9_1; ENST00000371100.9_1; ENST00000371102.8_1; ENST00000464624.7_1; ENST00000676826.2_1
Gene Name: GNAS
Drug Name: Daporinad
LogIC50
2.5
0.0
−2.5
−5.0
Biallelic n=15
Monoallelic n=4
Group
