# Supplementary material for: Allelic expression patterns of imprinted and non-imprinted genes in cancer cell lines from multiple histologies
Source: Clin Epigenetics. 2025 May 25;17:83. doi: 10.1186/s13148-025-01883-3 (PMC12105275; doi:10.1186/s13148-025-01883-3)
Supplement: Supplementary file 22 — Supplementary Material 22. Figure S11. Violin plots for associated agents and expression patterns of the isoform groups satisfying pFDR < 0.05 for 60 predominantly monoallelic genes. [file 13148_2025_1883_MOESM22_ESM.pdf]

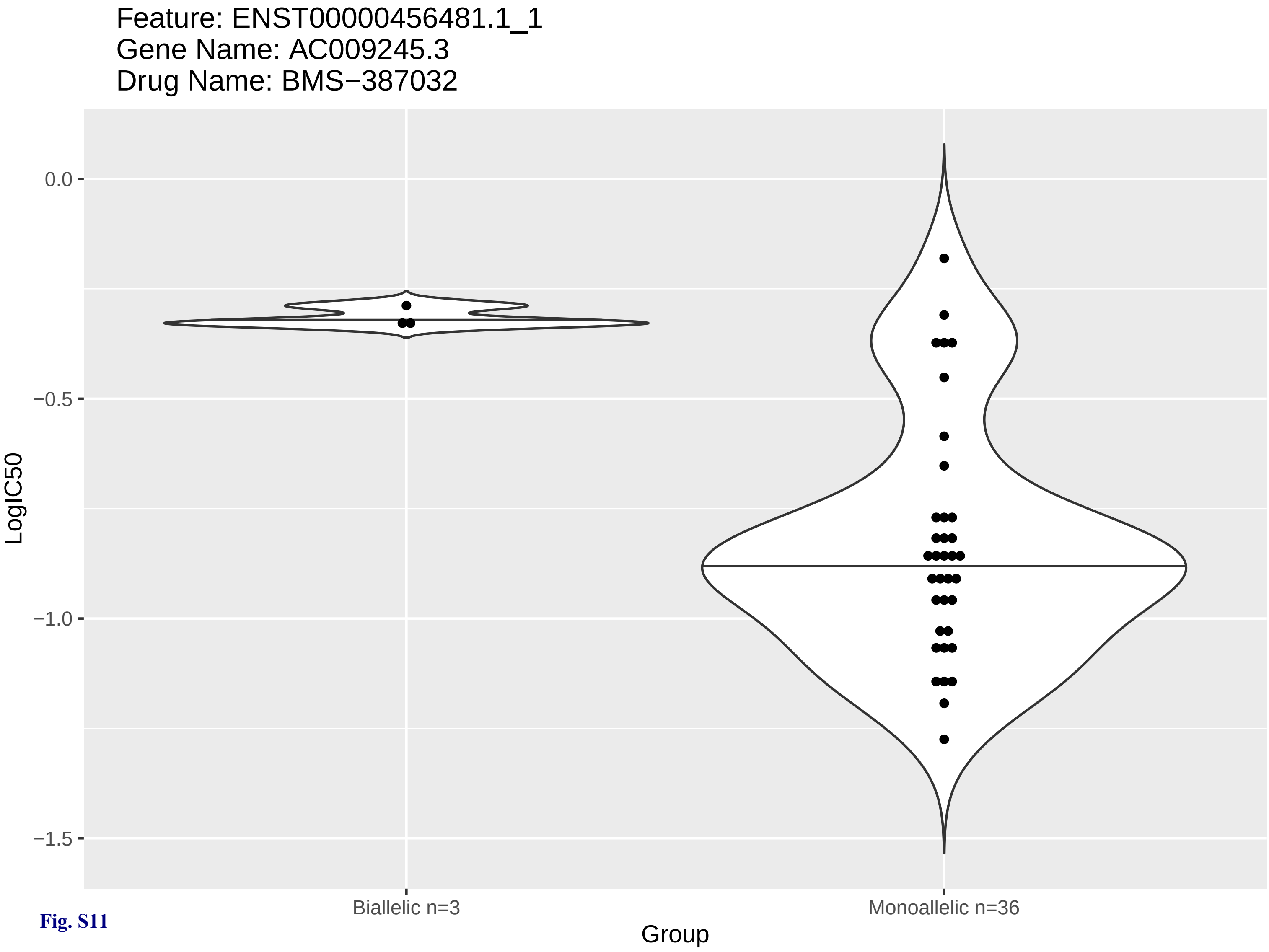
Feature: ENST00000456481.1_1
Gene Name: AC009245.3
Drug Name: BMS−387032
0.0
−0.5
−1.0
−1.5
LogIC50
Biallelic n=3
Monoallelic n=36
Group

Fig. S11

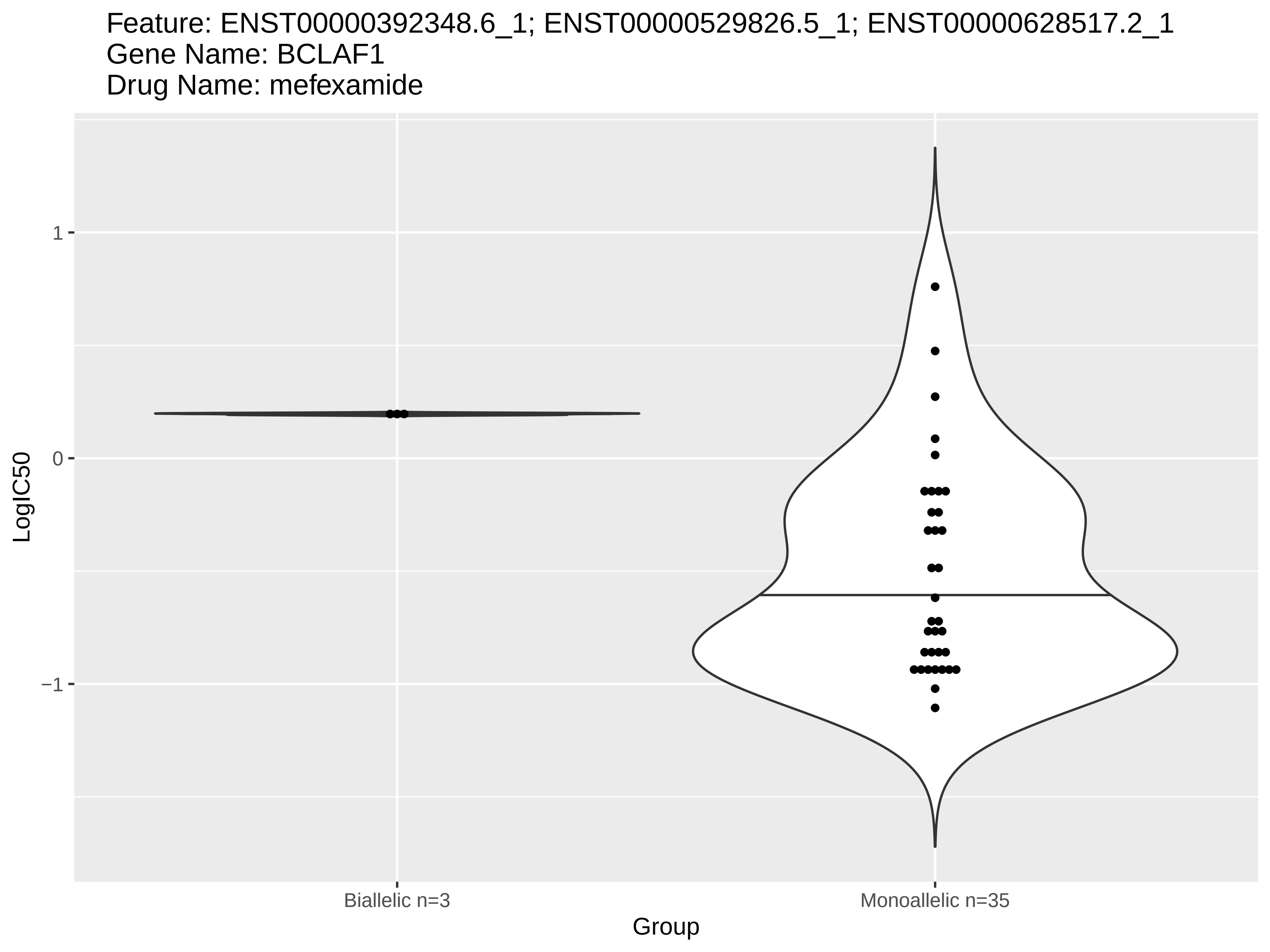

Feature: ENST00000392348.6_1; ENST00000529826.5_1; ENST00000628517.2_1
Gene Name: BCLAF1
Drug Name: mefexamide
1
0
−1
LogIC50
Biallelic n=3
Monoallelic n=35
Group

Feature: ENST00000377619.9_1
Gene Name: COMMD6
Drug Name: Pelitinib

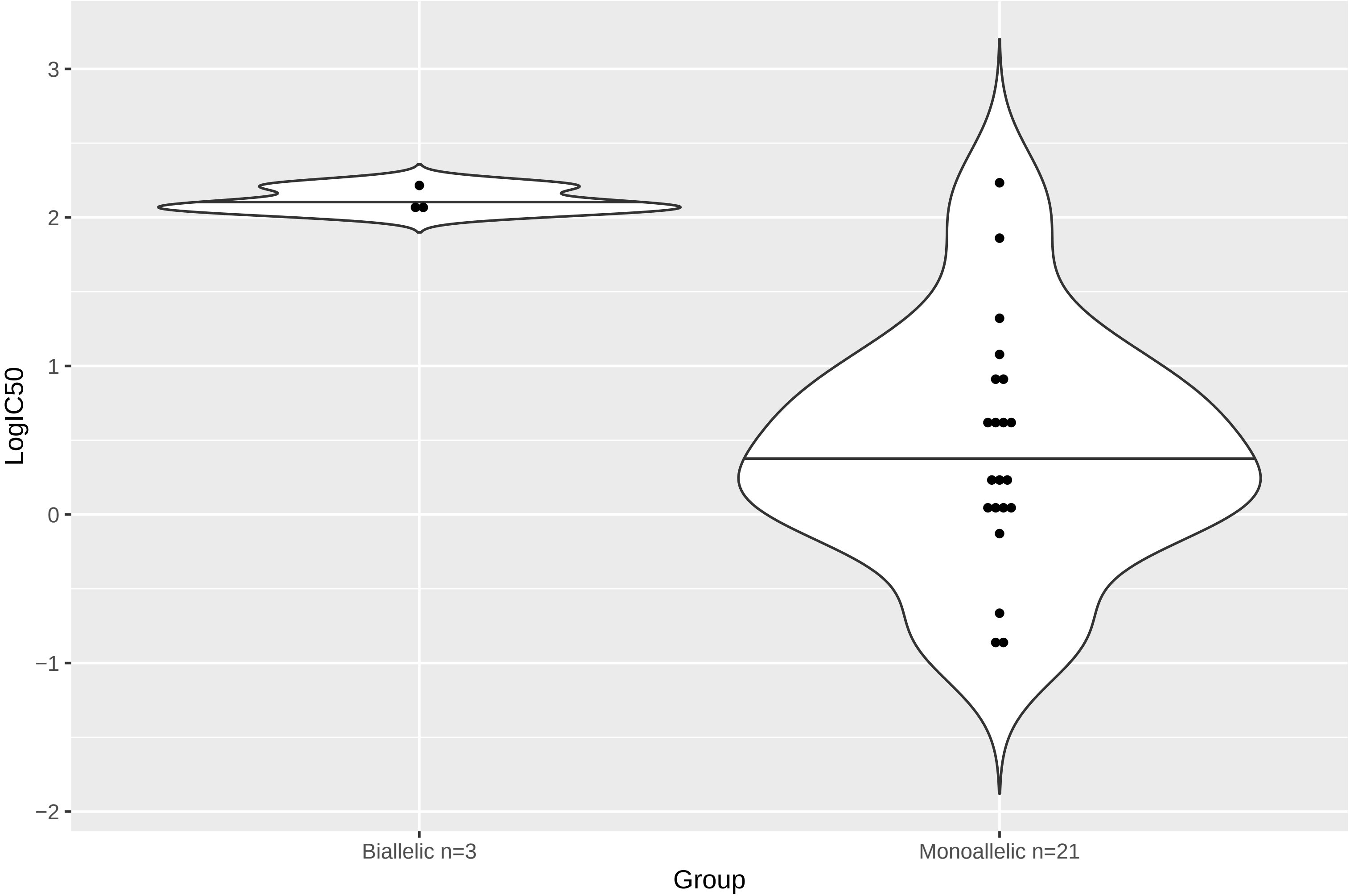

Feature: ENST00000377619.9_1
Gene Name: COMMD6
Drug Name: MIM1

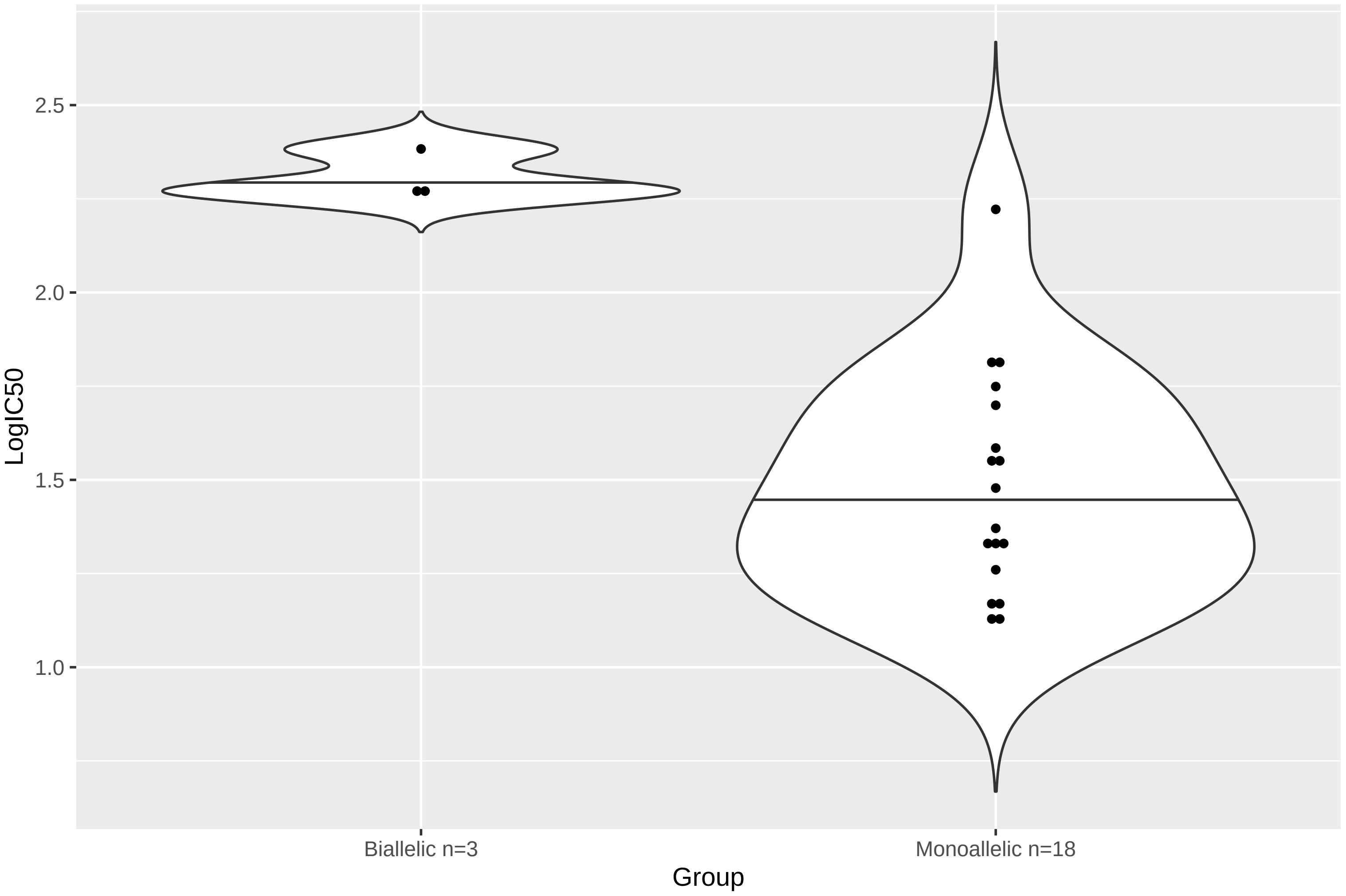

Feature: ENST00000456481.1_1
Gene Name: AC009245.3
Drug Name: CX−5461

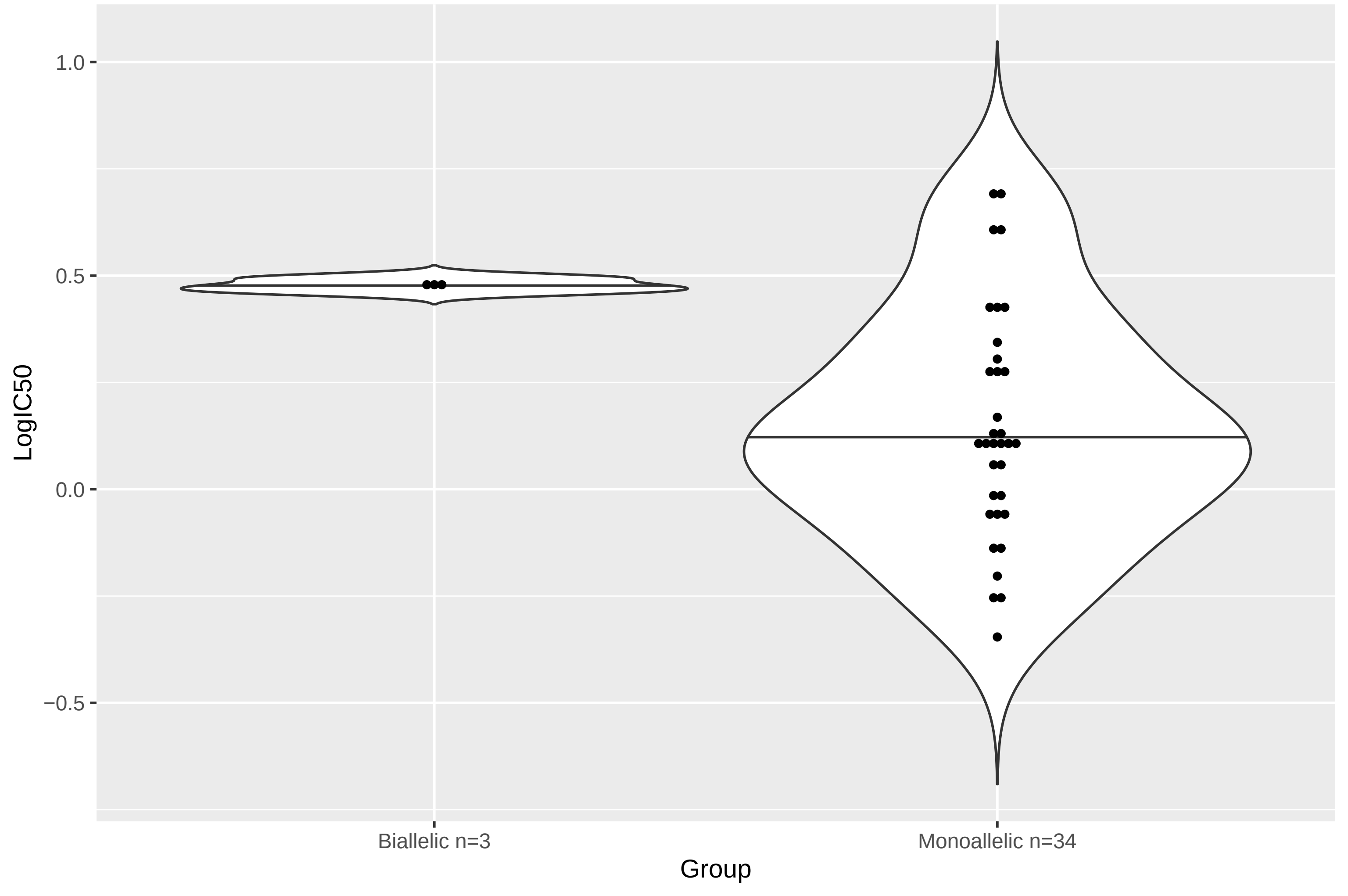

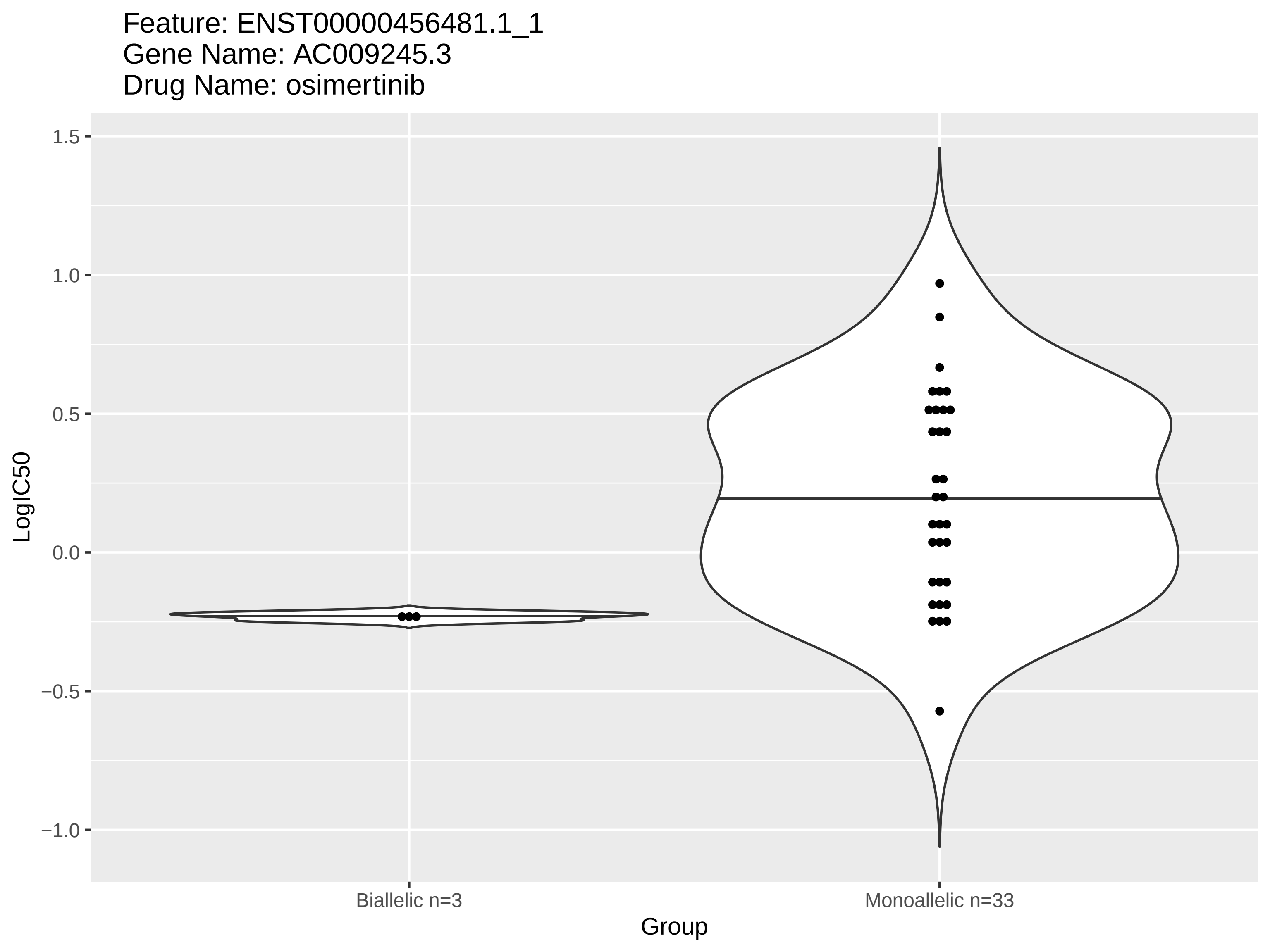

Feature: ENST00000456481.1_1
Gene Name: AC009245.3
Drug Name: osimertinib
1.5
1.0
0.5
0.0
−0.5
−1.0
LogIC50
Biallelic n=3
Monoallelic n=33
Group

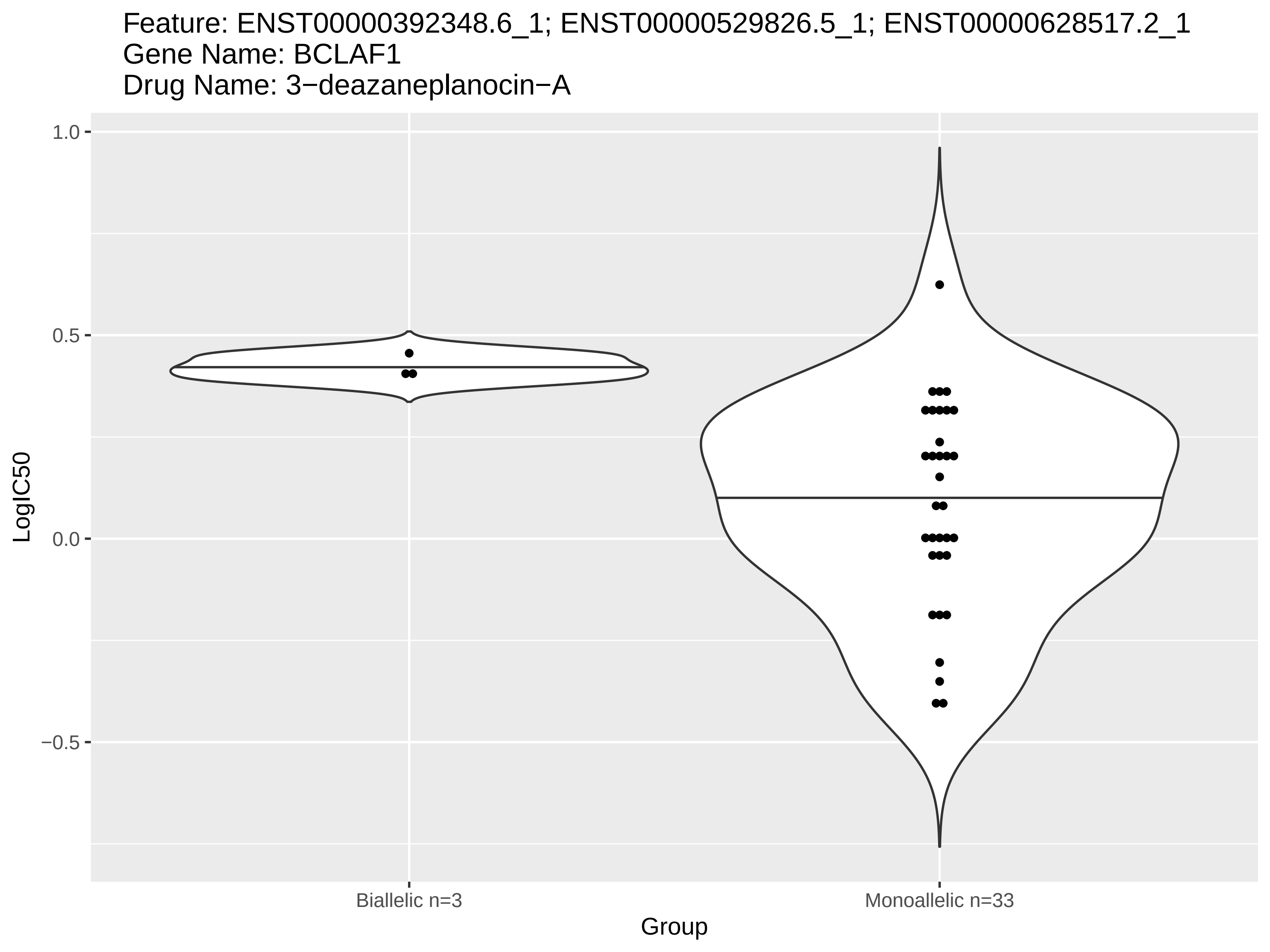

Feature: ENST00000392348.6_1; ENST00000529826.5_1; ENST00000628517.2_1
Gene Name: BCLAF1
Drug Name: 3−deazaneplanocin−A
1.0
0.5
0.0
−0.5
LogIC50
Biallelic n=3
Monoallelic n=33
Group

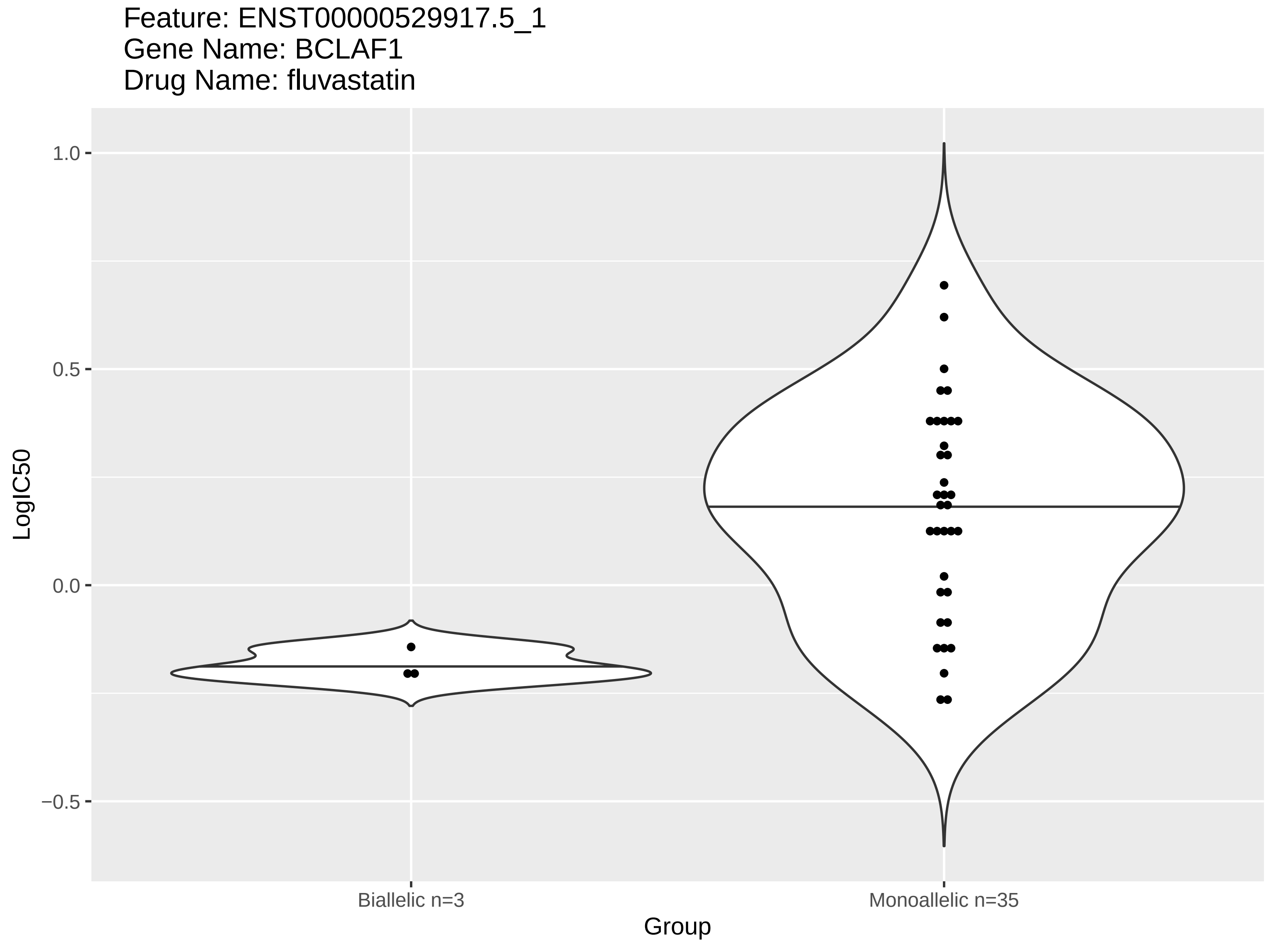
Feature: ENST00000529917.5_1
Gene Name: BCLAF1
Drug Name: fluvastatin
1.0
0.5
0.0
−0.5
LogIC50
Biallelic n=3
Monoallelic n=35
Group

Feature: ENST00000392348.6_1; ENST00000529826.5_1; ENST00000628517.2_1
Gene Name: BCLAF1
Drug Name: topotecan

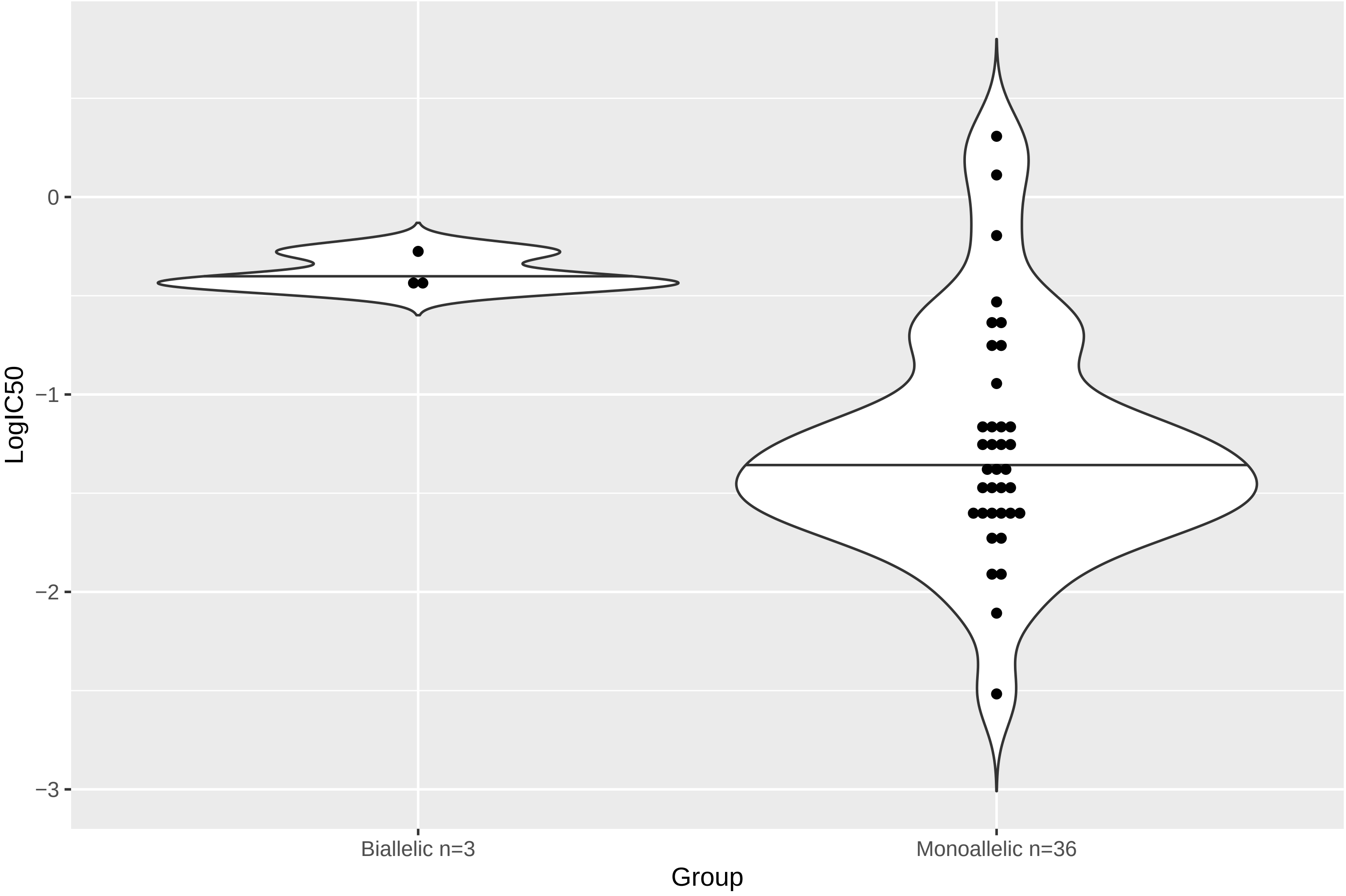

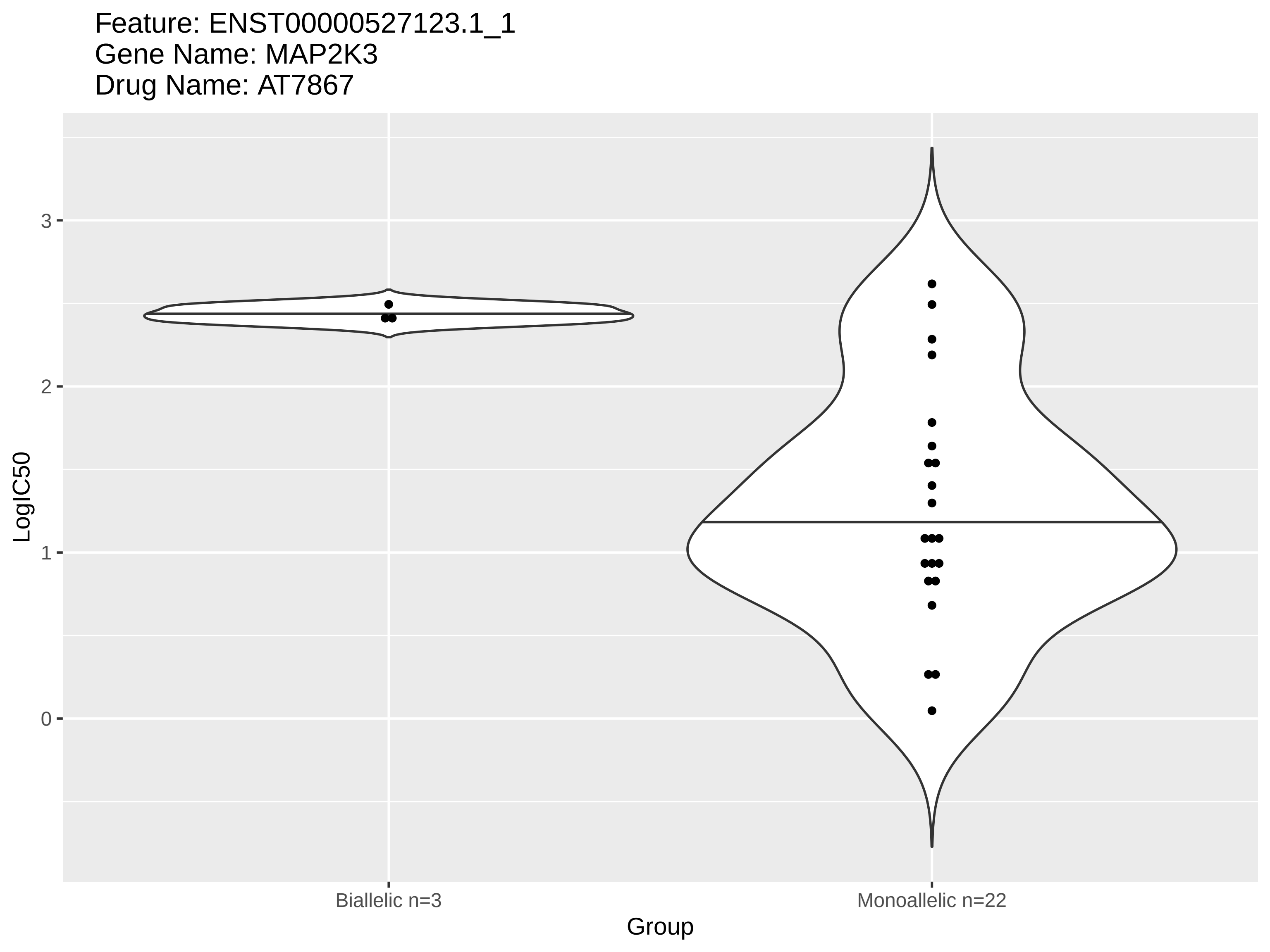
Feature: ENST00000527123.1_1
Gene Name: MAP2K3
Drug Name: AT7867
LogIC50
3
2
1
0
Biallelic n=3
Monoallelic n=22
Group

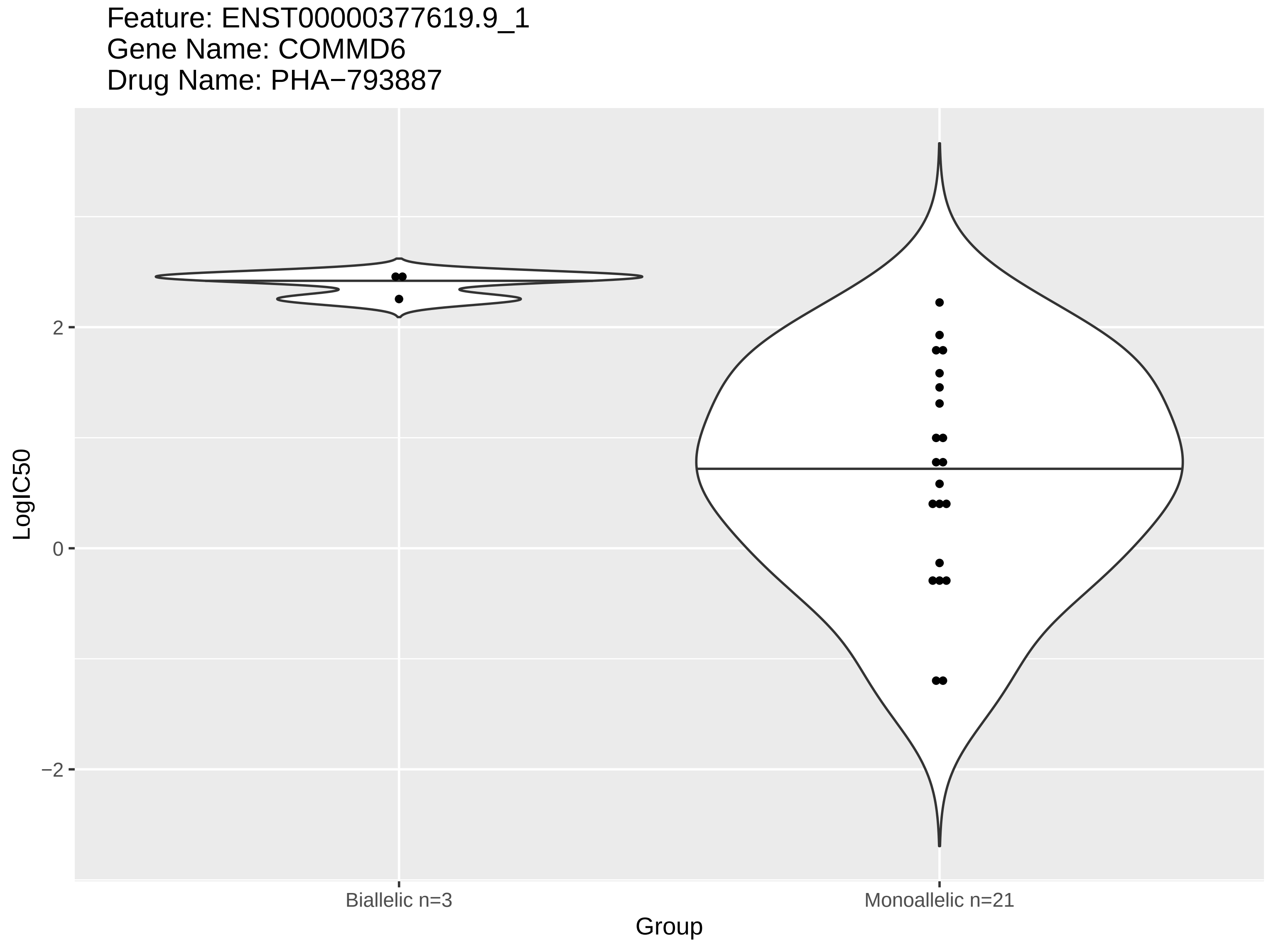
Feature: ENST00000377619.9_1
Gene Name: COMMD6
Drug Name: PHA−793887
LogIC50
2
0
−2
Biallelic n=3
Monoallelic n=21
Group

Feature: ENST00000527536.5_1
Gene Name: BCLAF1
Drug Name: FTI−277

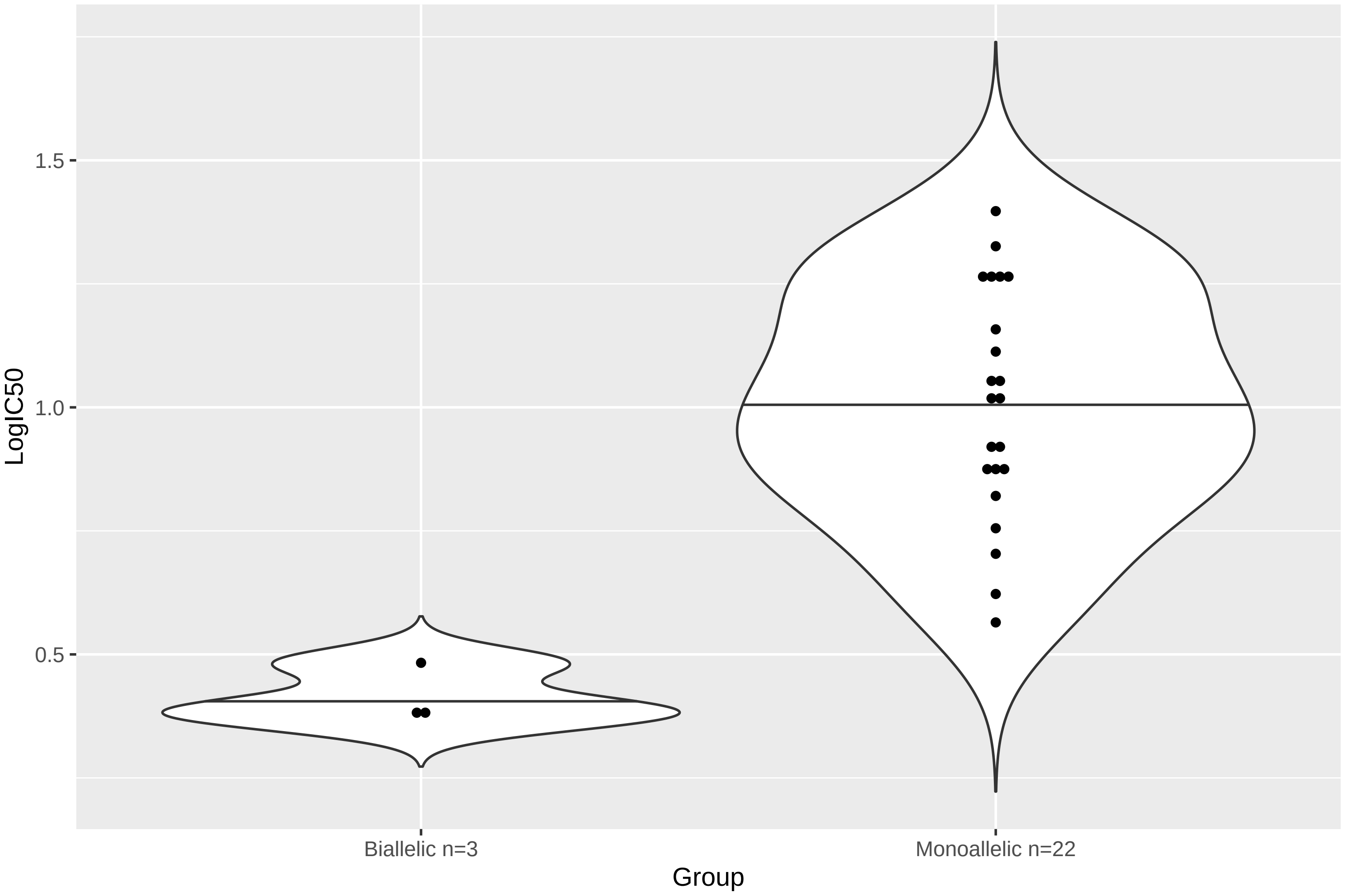

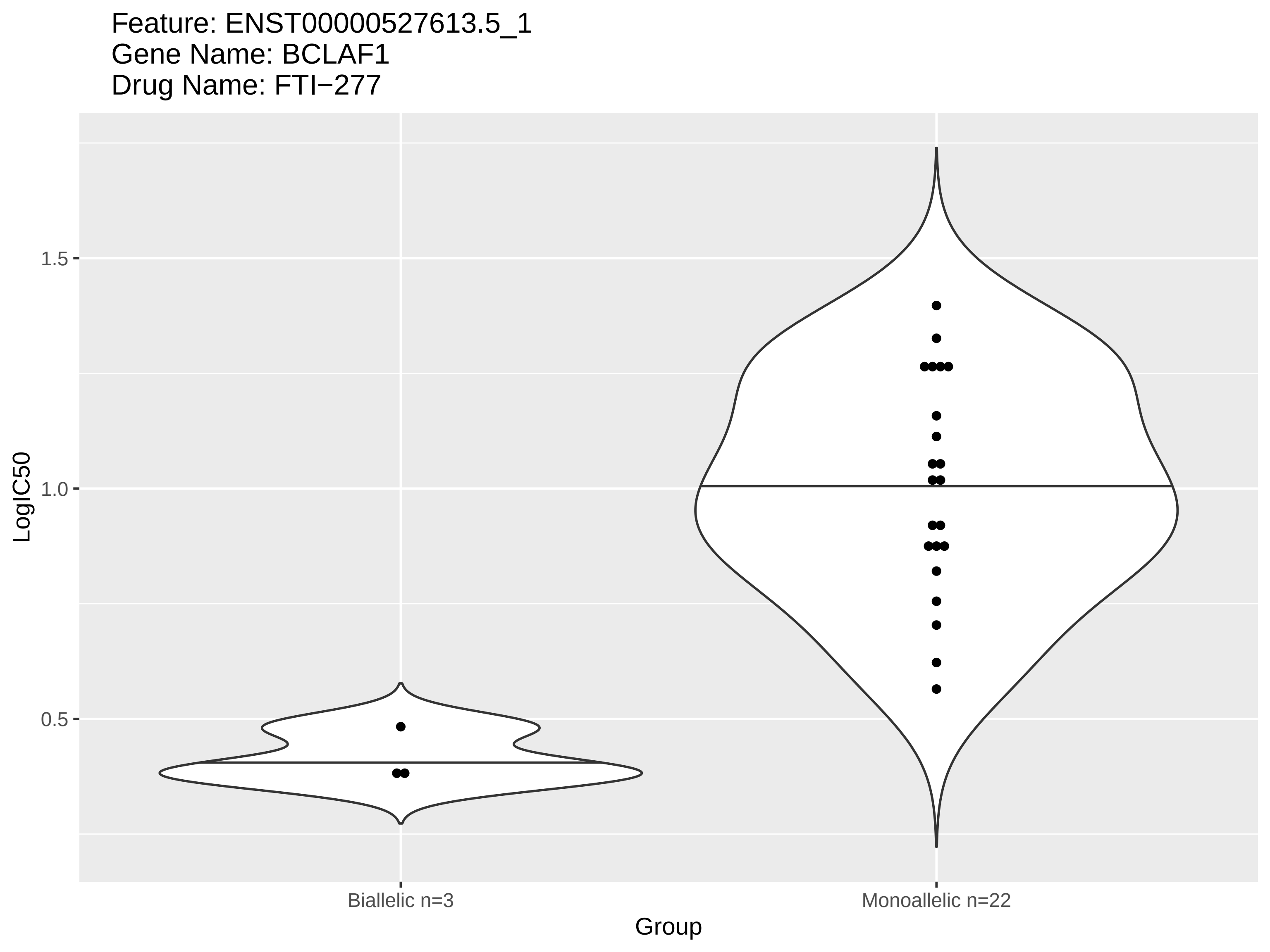
Feature: ENST00000527613.5_1
Gene Name: BCLAF1
Drug Name: FTI−277
LogIC50
1.5
1.0
0.5
Biallelic n=3
Monoallelic n=22
Group

Feature: ENST00000527759.5_1
Gene Name: BCLAF1
Drug Name: FTI−277

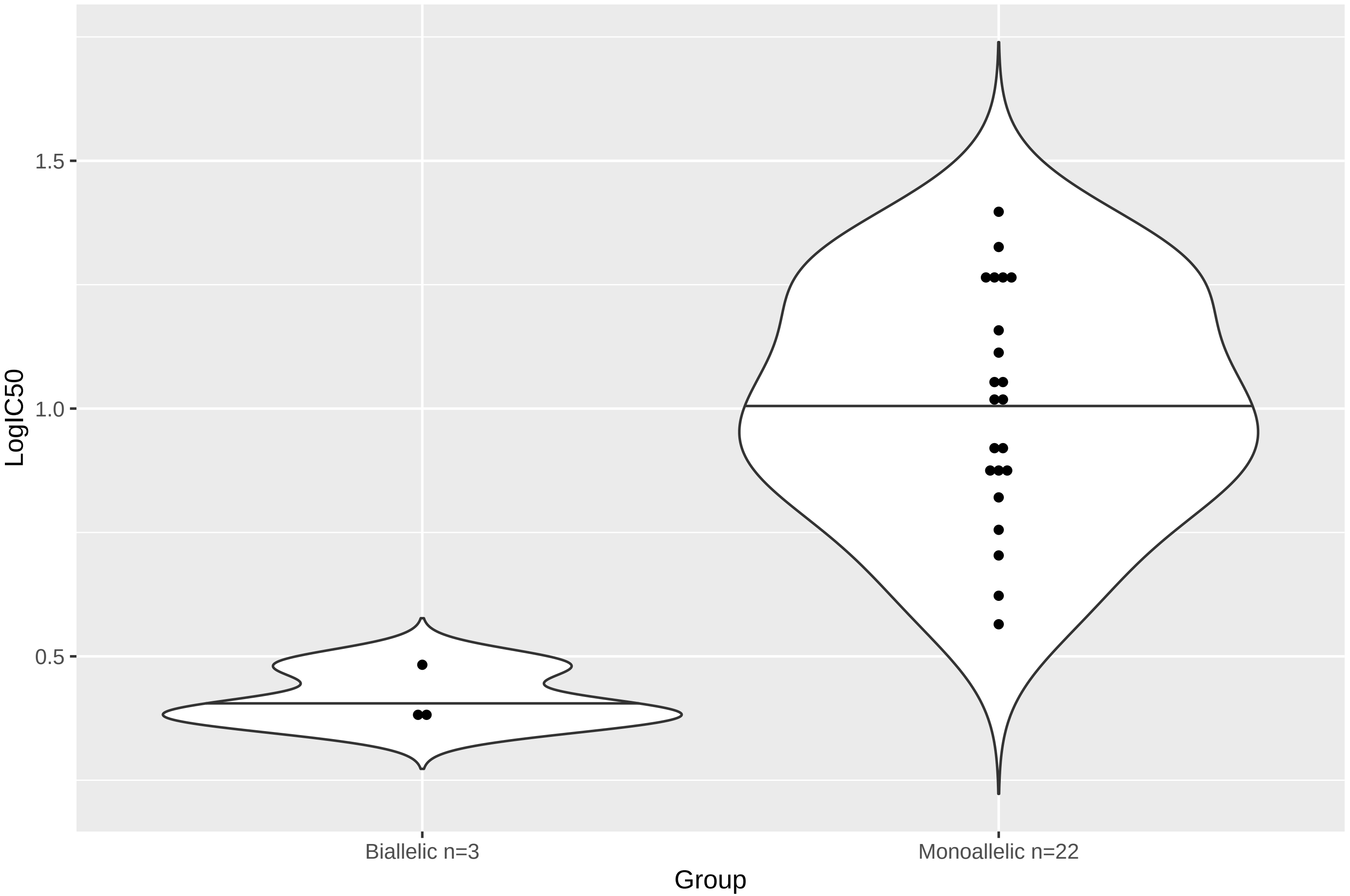

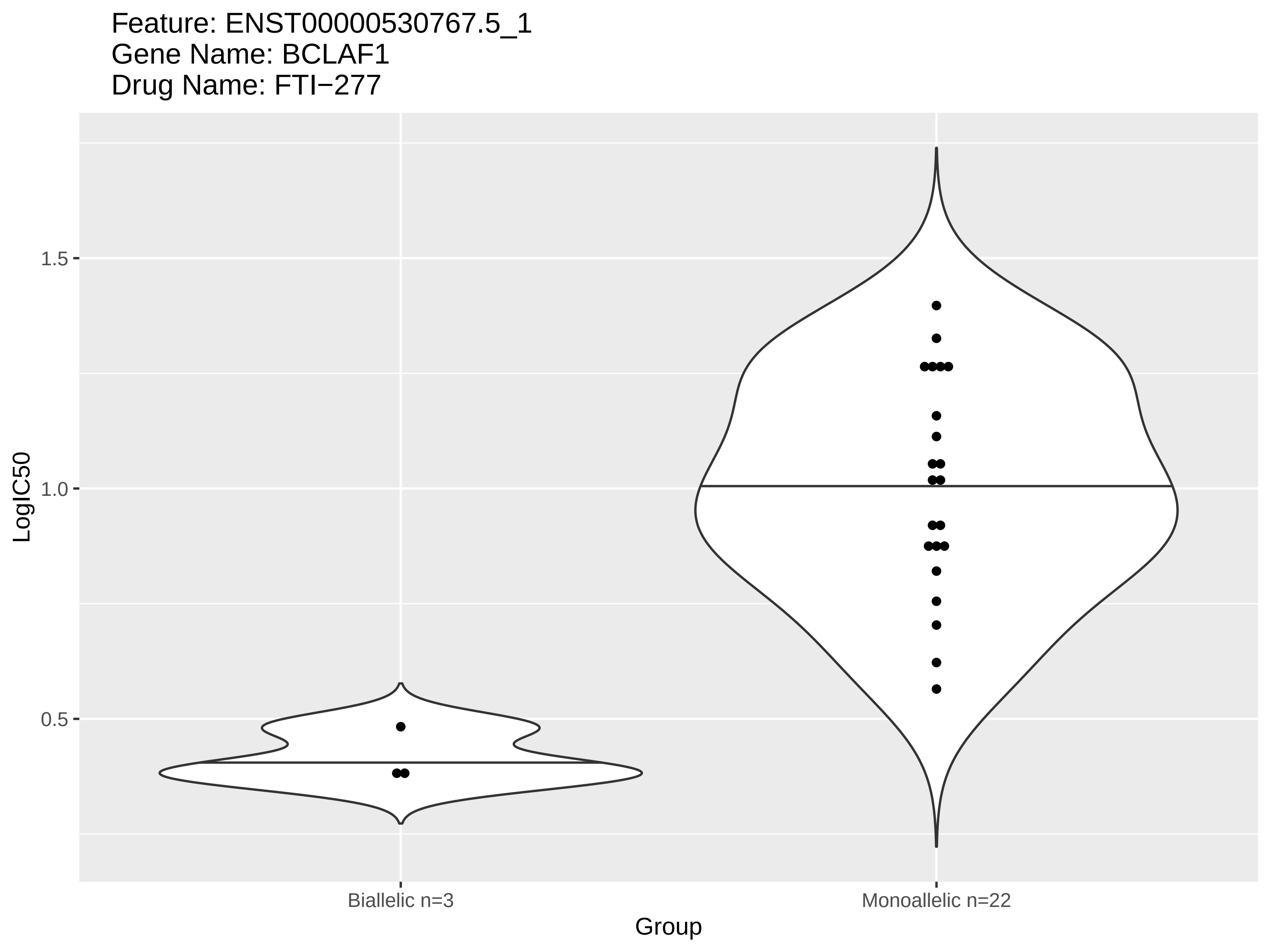
Feature: ENST00000530767.5_1
Gene Name: BCLAF1
Drug Name: FTI−277
LogIC50
1.5
1.0
0.5
Biallelic n=3
Monoallelic n=22
Group

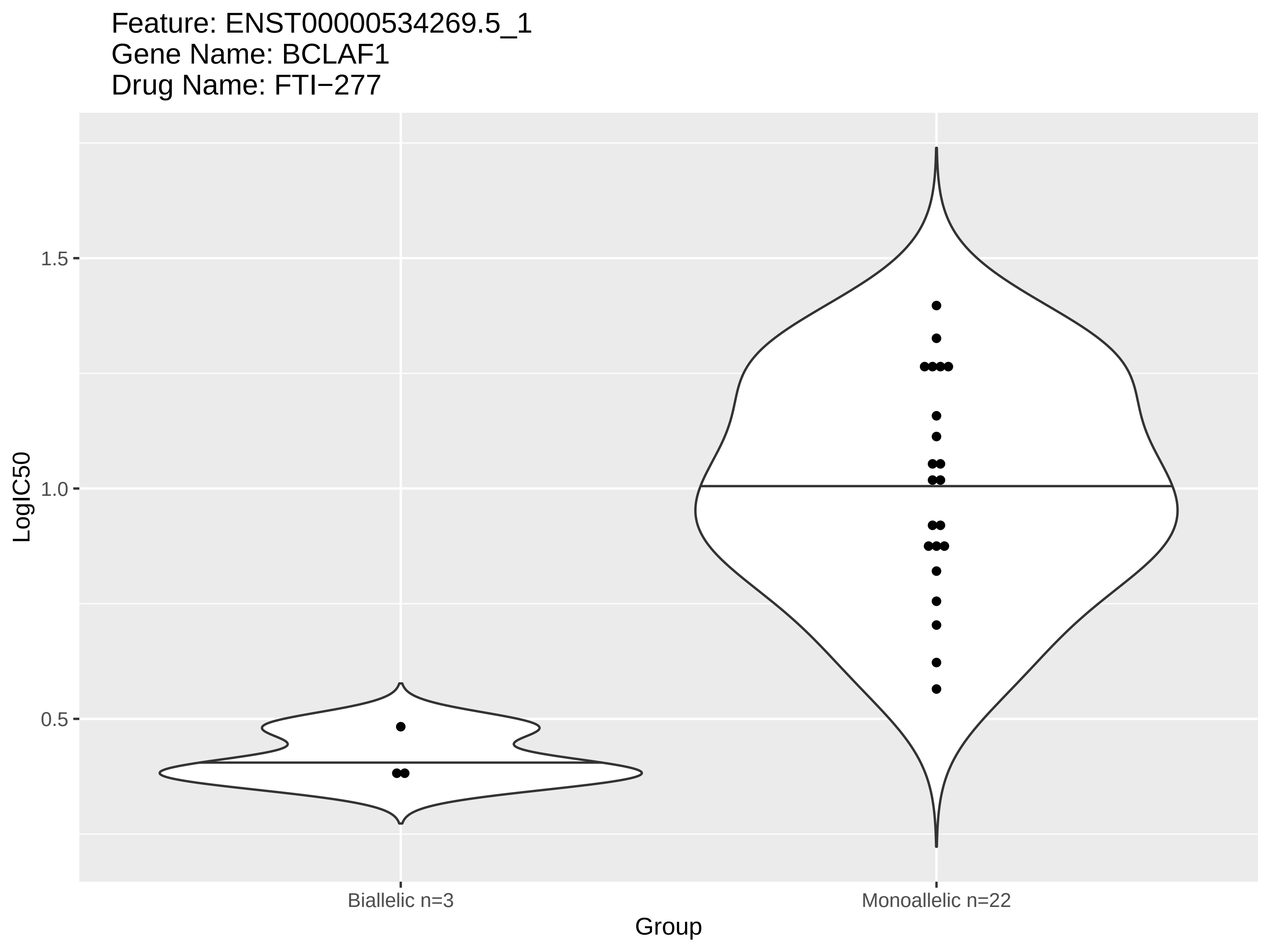

Feature: ENST00000534269.5_1
Gene Name: BCLAF1
Drug Name: FTI−277
LogIC50
1.5
1.0
0.5
Biallelic n=3
Monoallelic n=22
Group

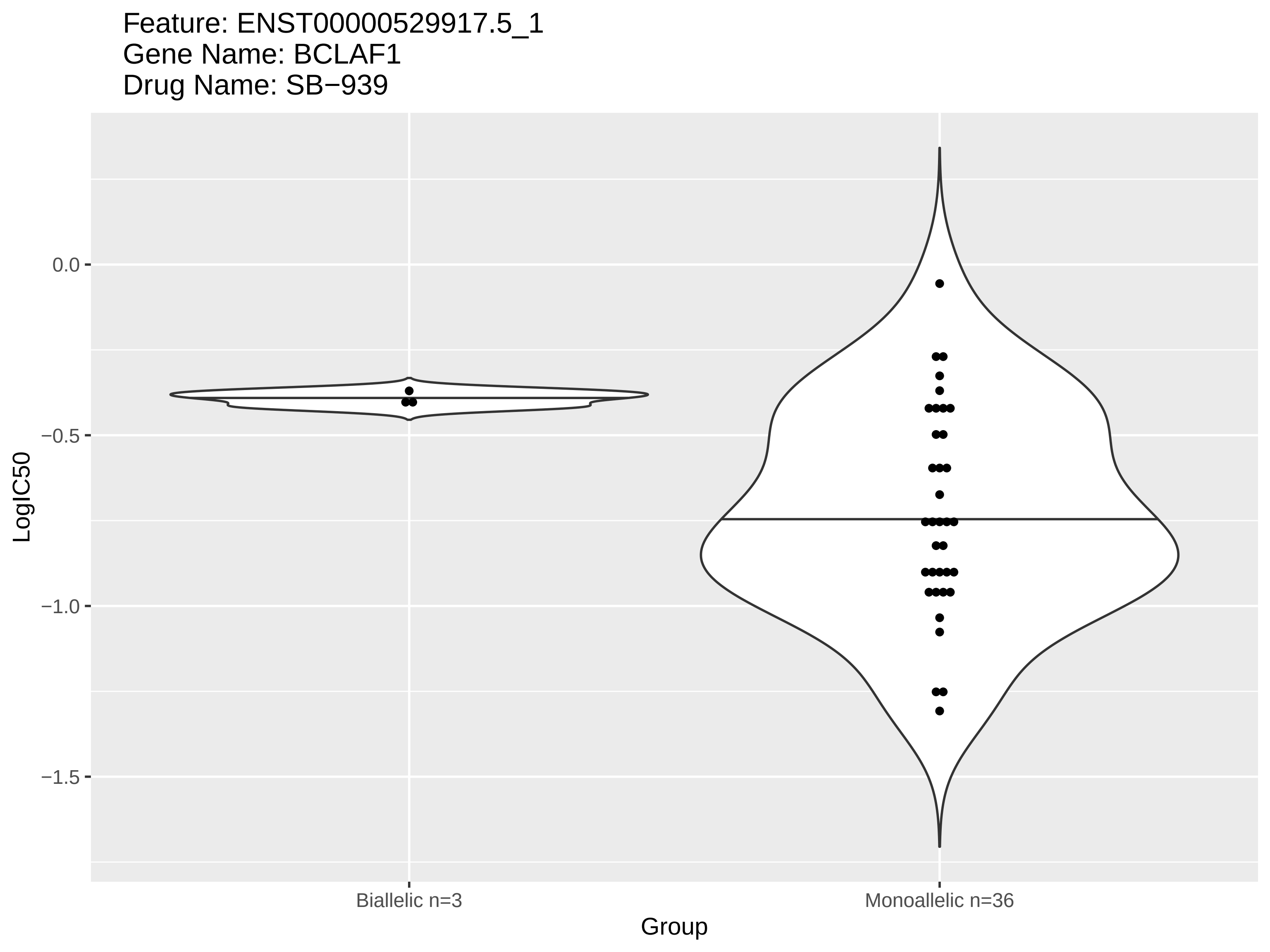
Feature: ENST00000529917.5_1
Gene Name: BCLAF1
Drug Name: SB−939
0.0
−0.5
−1.0
−1.5
LogIC50
Biallelic n=3
Monoallelic n=36
Group

Feature: ENST00000530429.5_1; ENST00000532384.5_1
Gene Name: BCLAF1
Drug Name: VER−49009

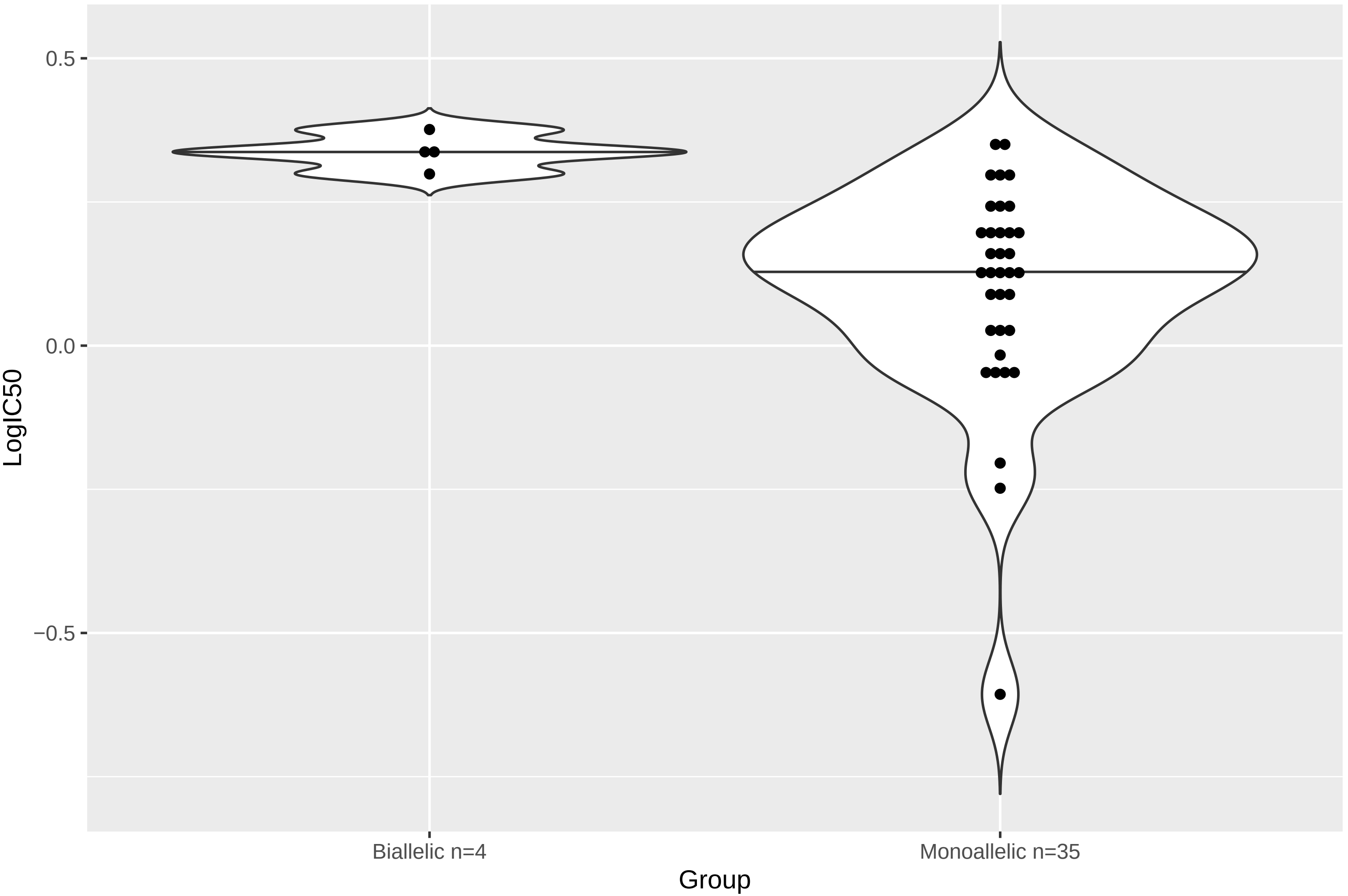

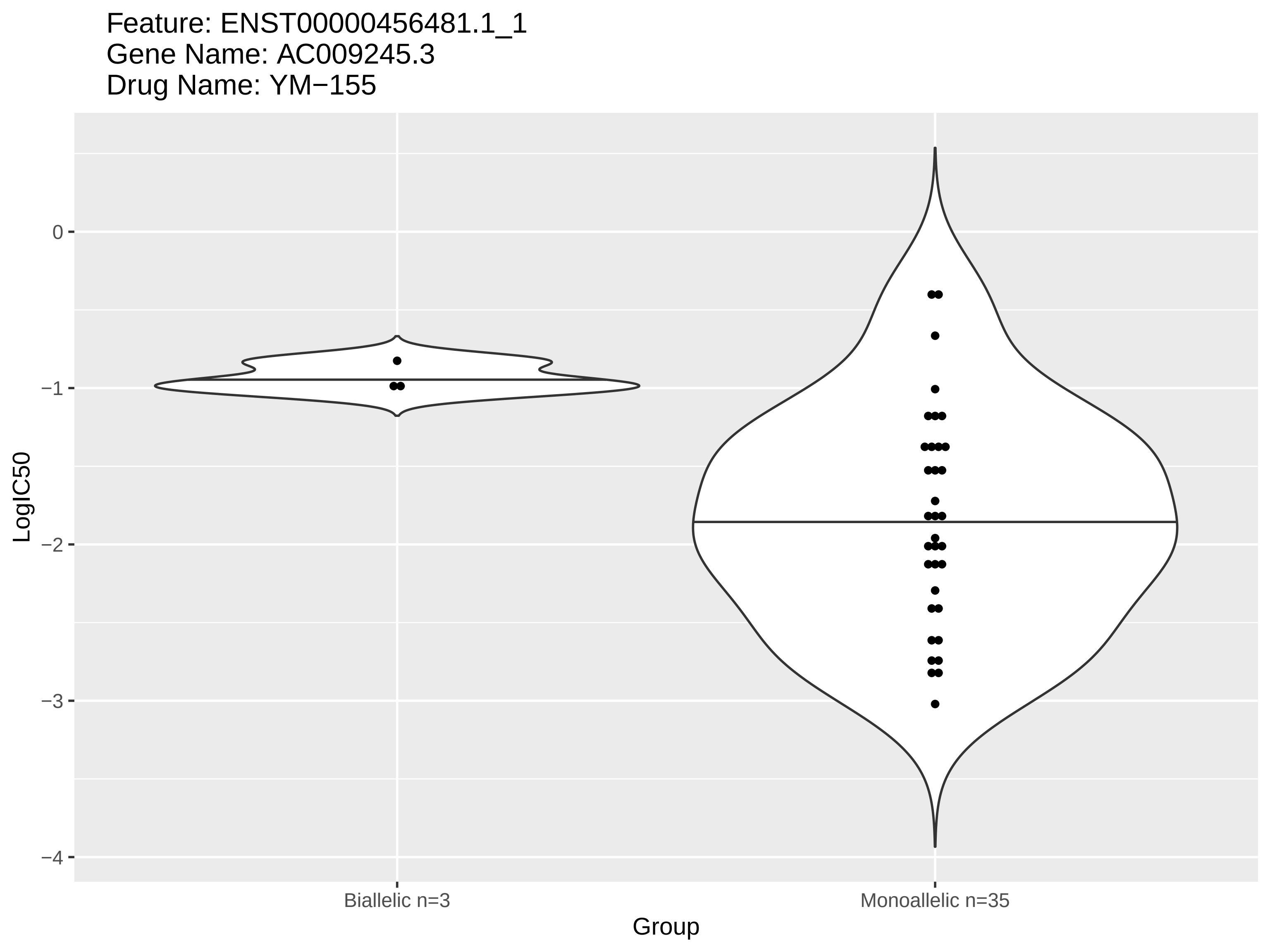
Feature: ENST00000456481.1_1
Gene Name: AC009245.3
Drug Name: YM−155
0
−1
−2
−3
−4
LogIC50
Biallelic n=3
Monoallelic n=35
Group

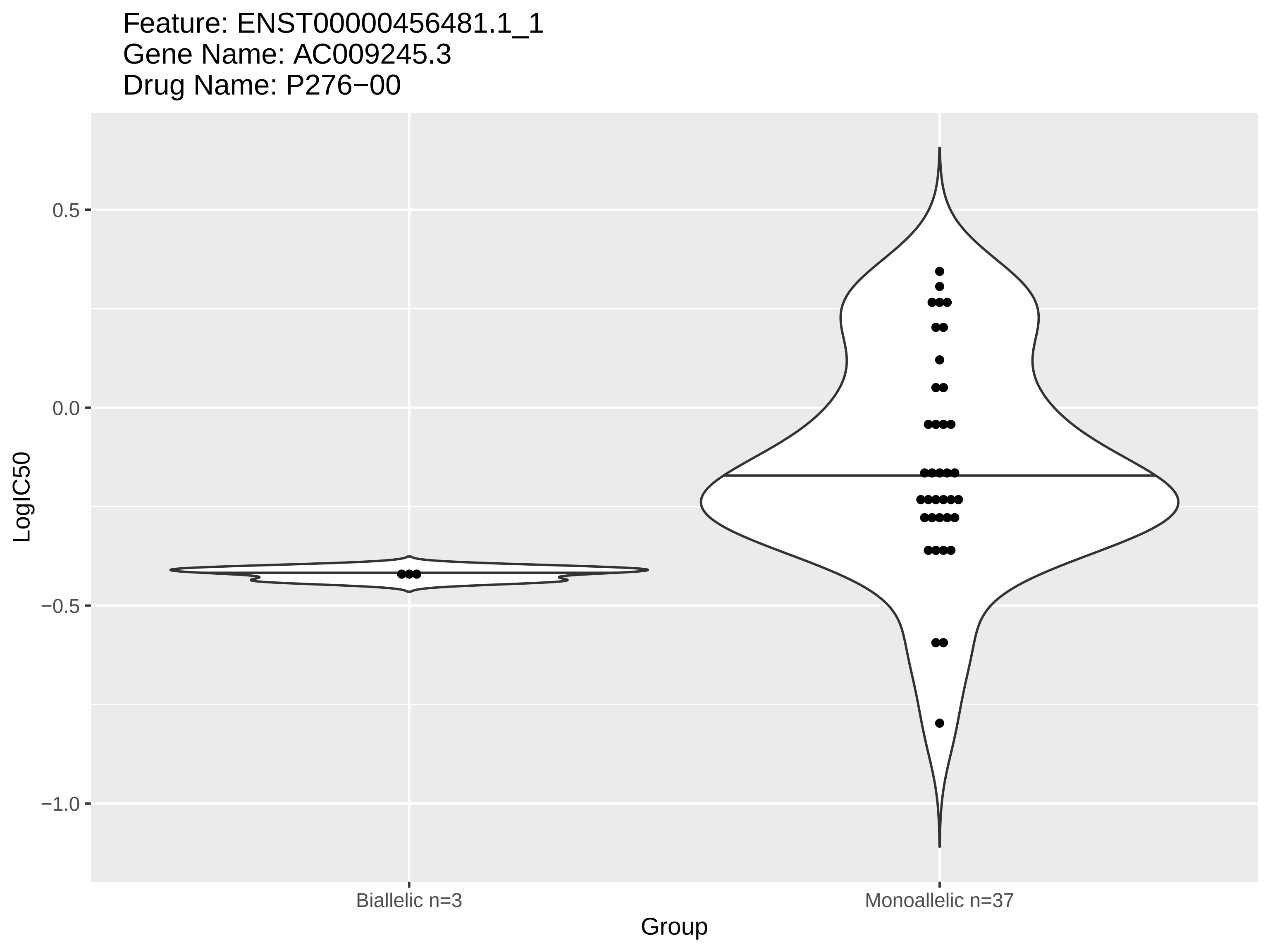

Feature: ENST00000456481.1_1
Gene Name: AC009245.3
Drug Name: P276−00
0.5
0.0
−0.5
−1.0
LogIC50
Biallelic n=3
Monoallelic n=37
Group

Feature: ENST00000526866.5_1; ENST00000532893.5_1
Gene Name: CDC27
Drug Name: CHIR−124

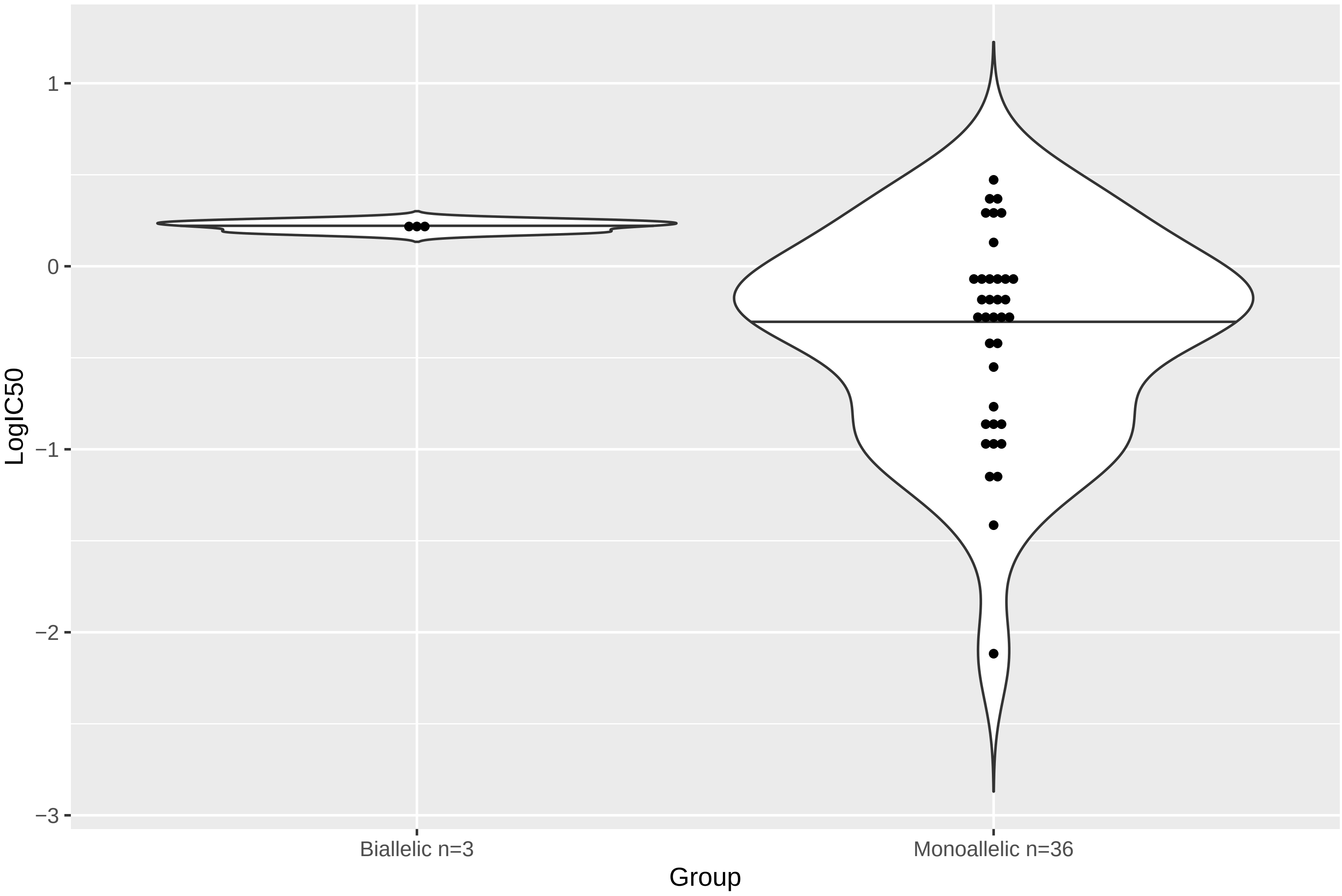

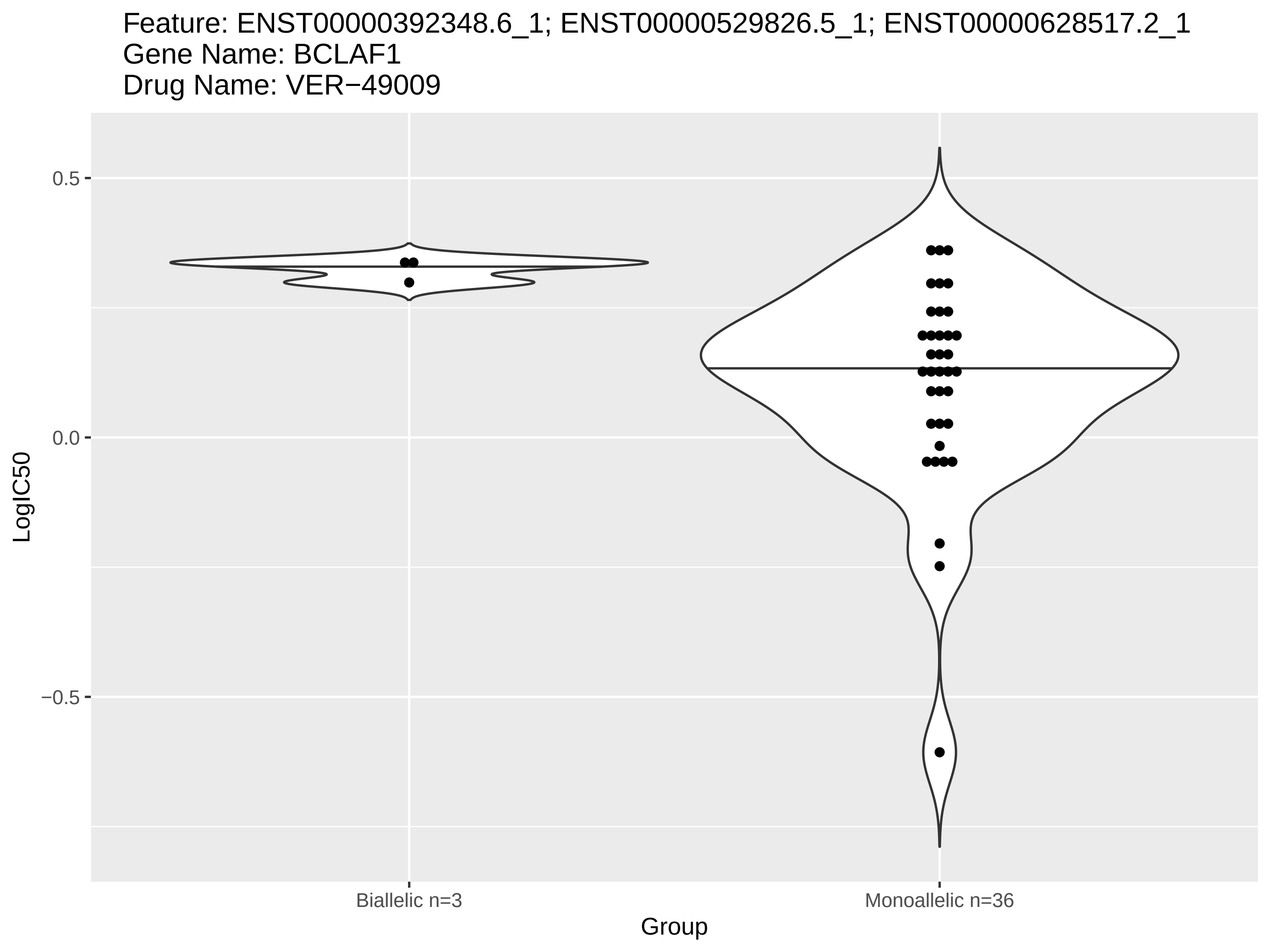

Feature: ENST00000392348.6_1; ENST00000529826.5_1; ENST00000628517.2_1
Gene Name: BCLAF1
Drug Name: VER−49009
0.5
0.0
−0.5
LogIC50
Biallelic n=3
Monoallelic n=36
Group

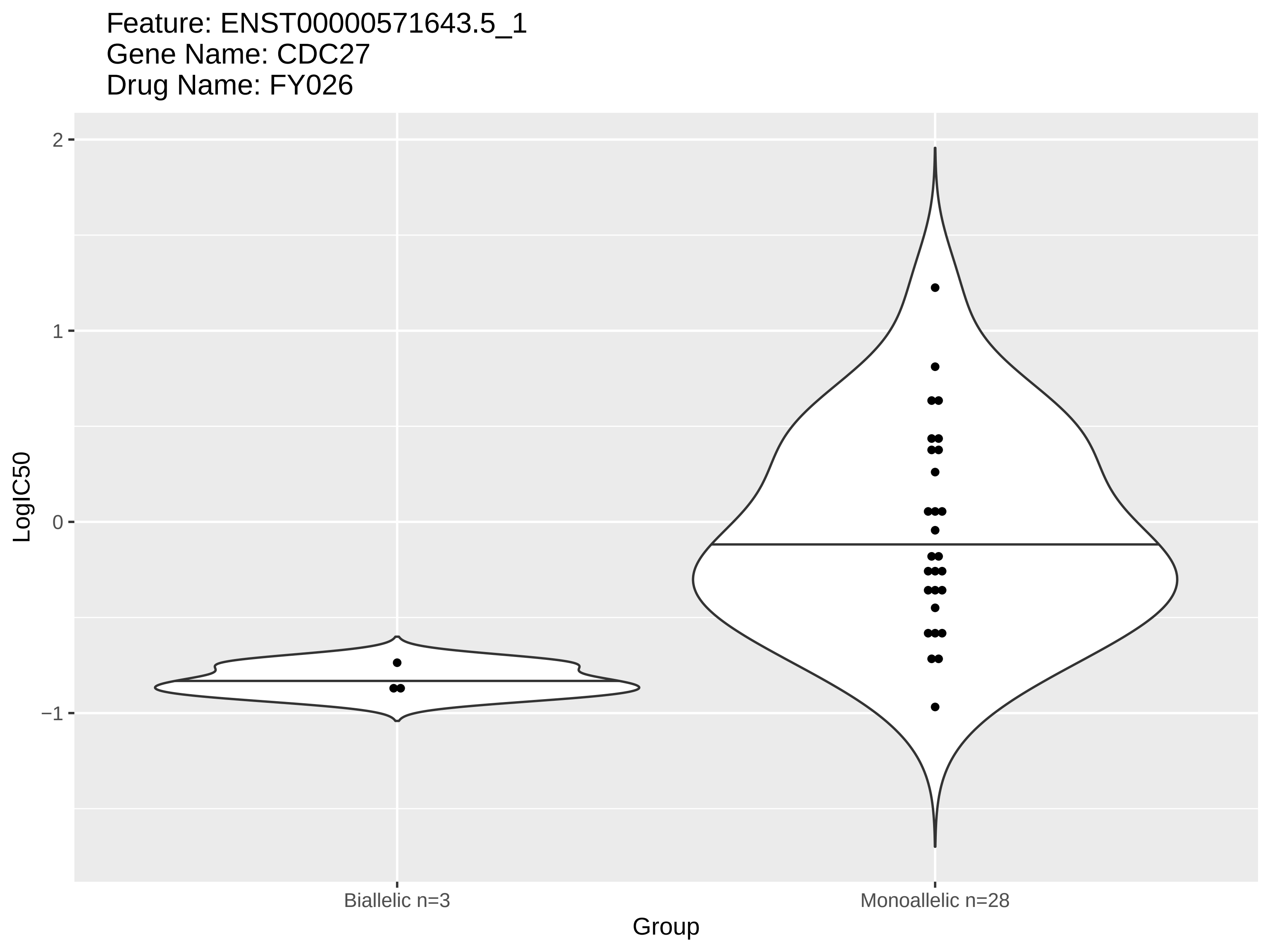

Feature: ENST00000571643.5_1
Gene Name: CDC27
Drug Name: FY026
LogIC50
2
1
0
−1
Biallelic n=3
Monoallelic n=28
Group

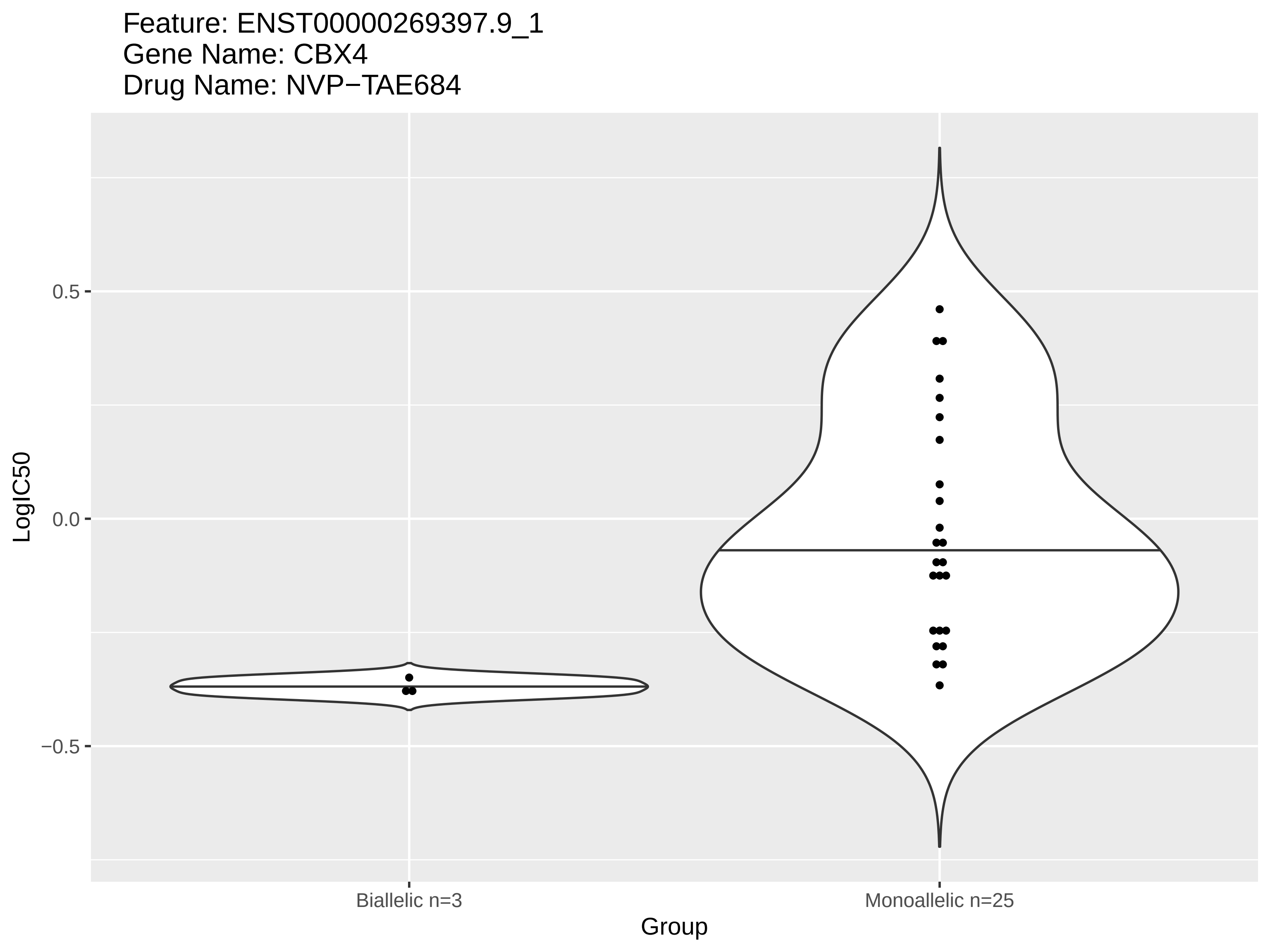
Feature: ENST00000269397.9_1
Gene Name: CBX4
Drug Name: NVP−TAE684
LogIC50
0.5
0.0
−0.5
Biallelic n=3
Monoallelic n=25
Group

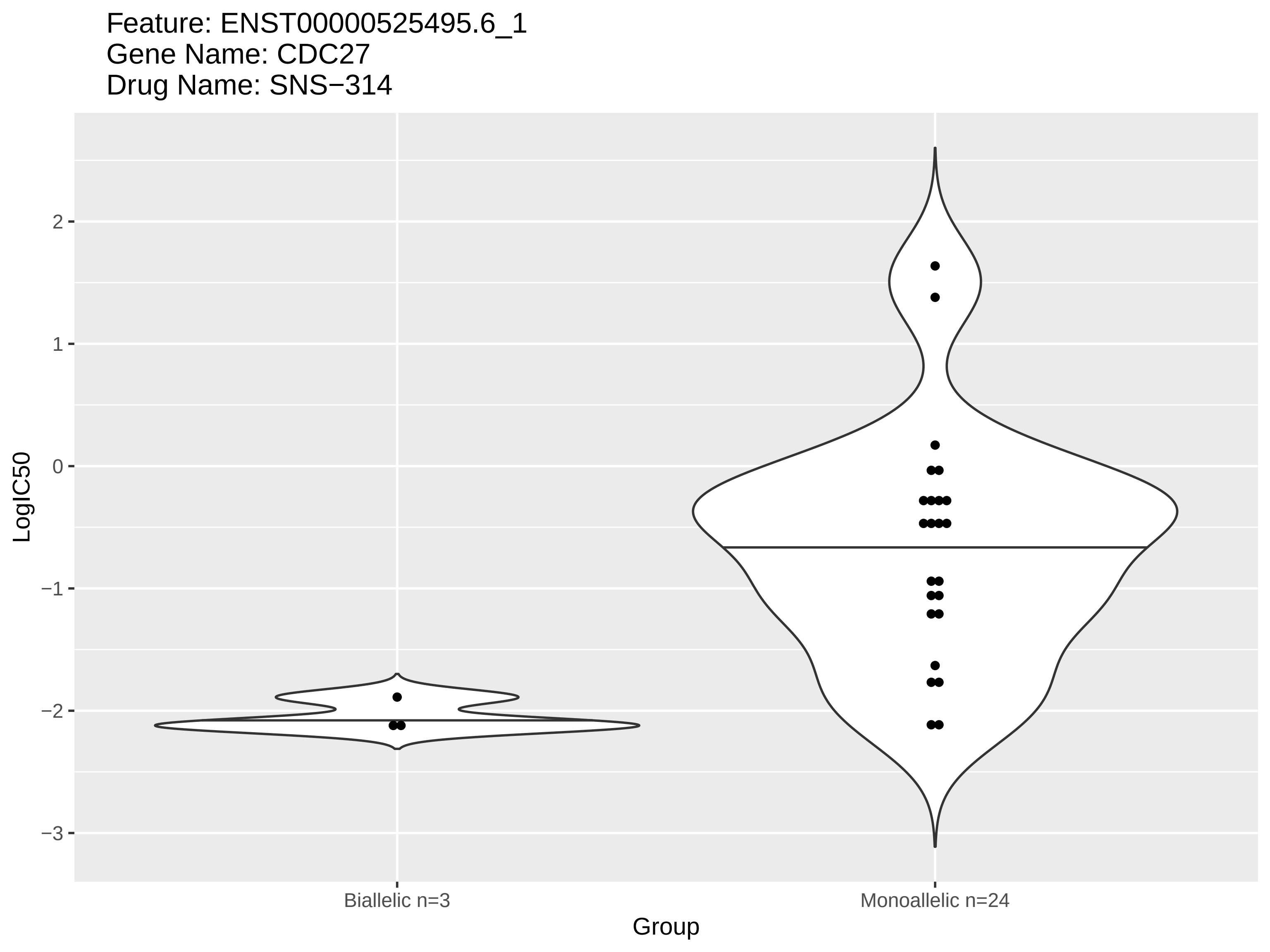
Feature: ENST00000525495.6_1
Gene Name: CDC27
Drug Name: SNS−314
LogIC50
2
1
0
−1
−2
−3
Biallelic n=3
Monoallelic n=24
Group

Feature: ENST00000526866.5_1; ENST00000532893.5_1
Gene Name: CDC27
Drug Name: SNS−314

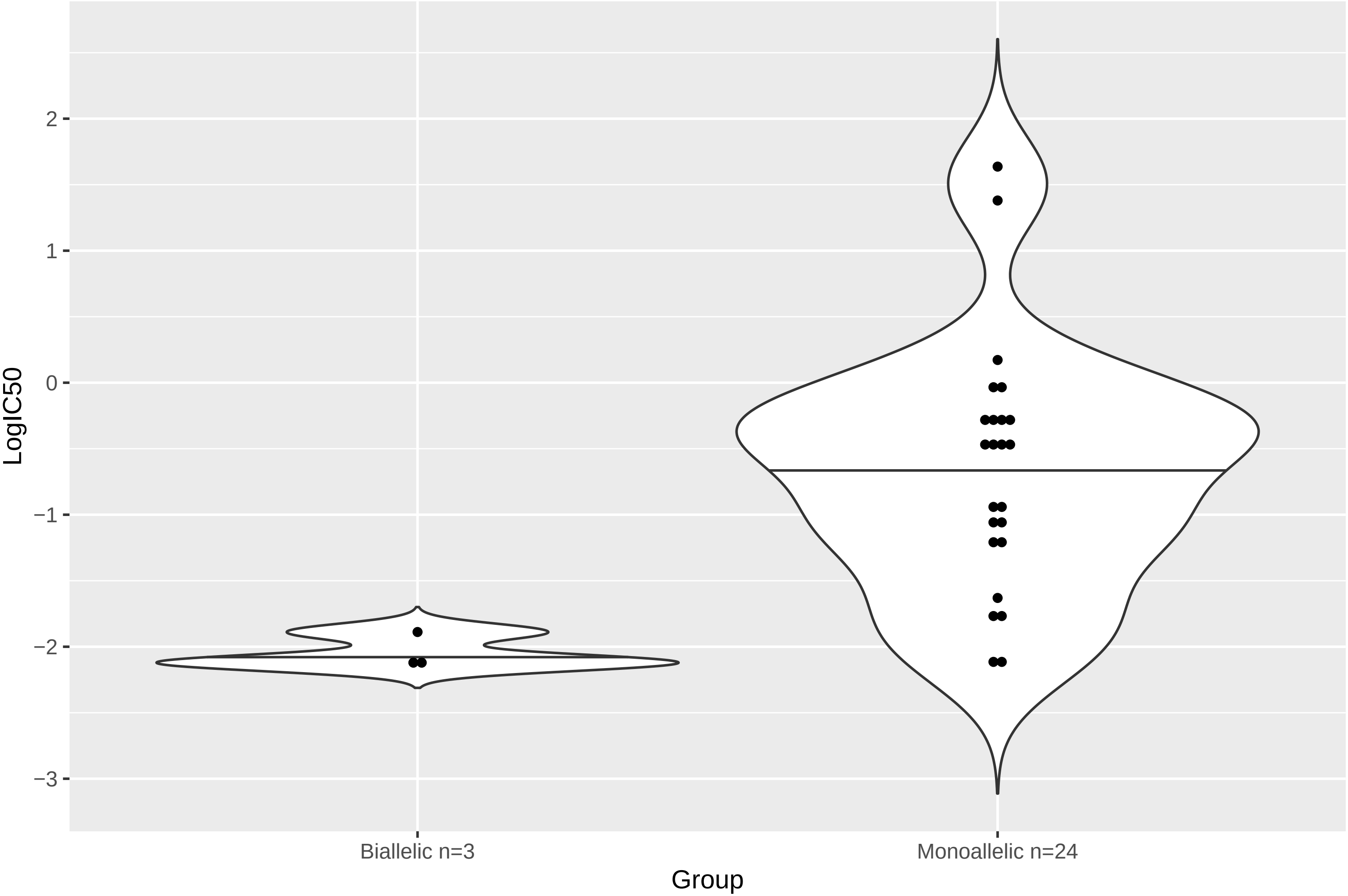

Feature: ENST00000525495.6_1
Gene Name: CDC27
Drug Name: CHIR−124

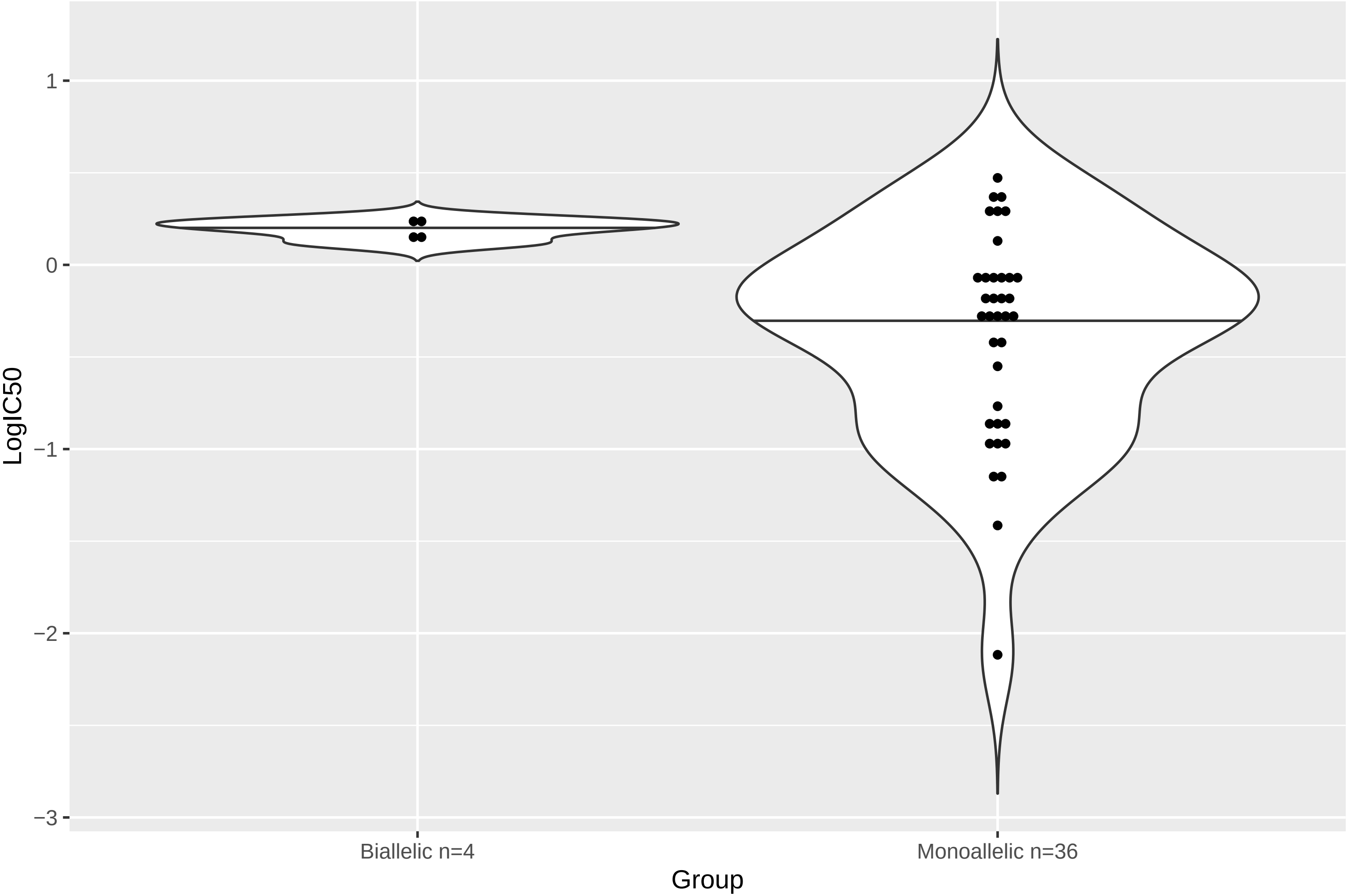

Feature: ENST00000529917.5_1
Gene Name: BCLAF1
Drug Name: SNX−2112

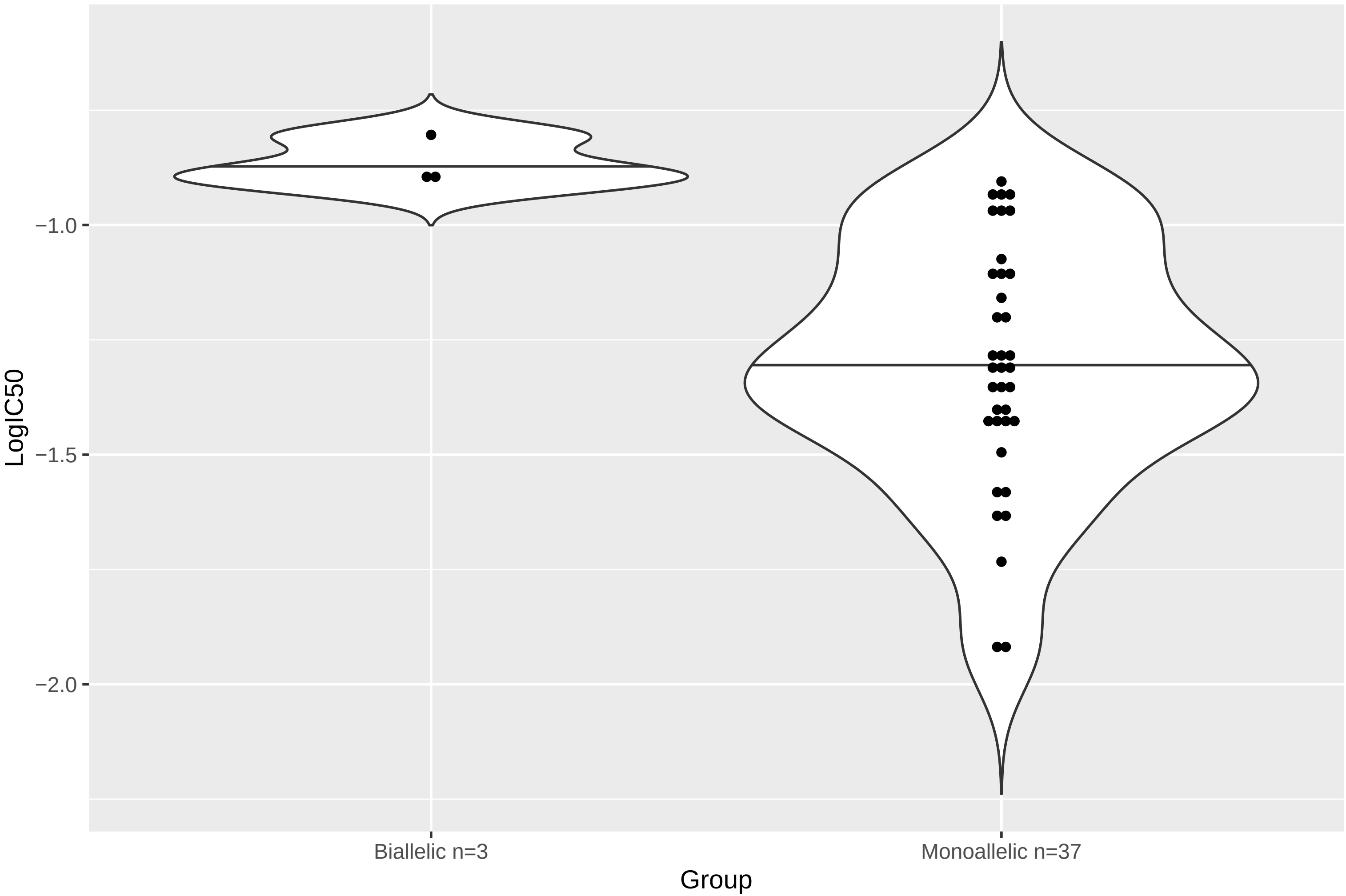

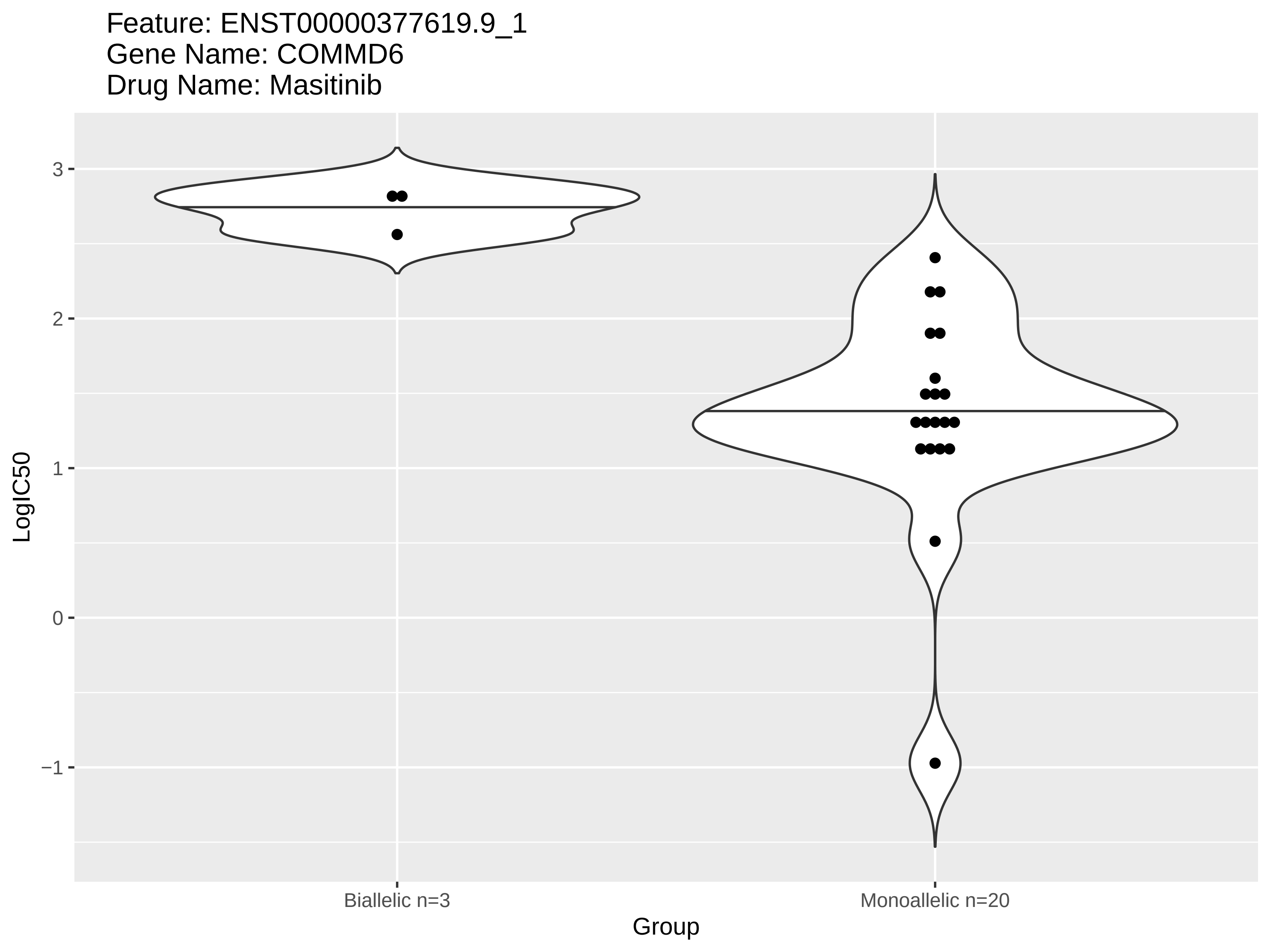
Feature: ENST00000377619.9_1
Gene Name: COMMD6
Drug Name: Masitinib
3
2
1
0
−1
LogIC50
Biallelic n=3
Monoallelic n=20
Group

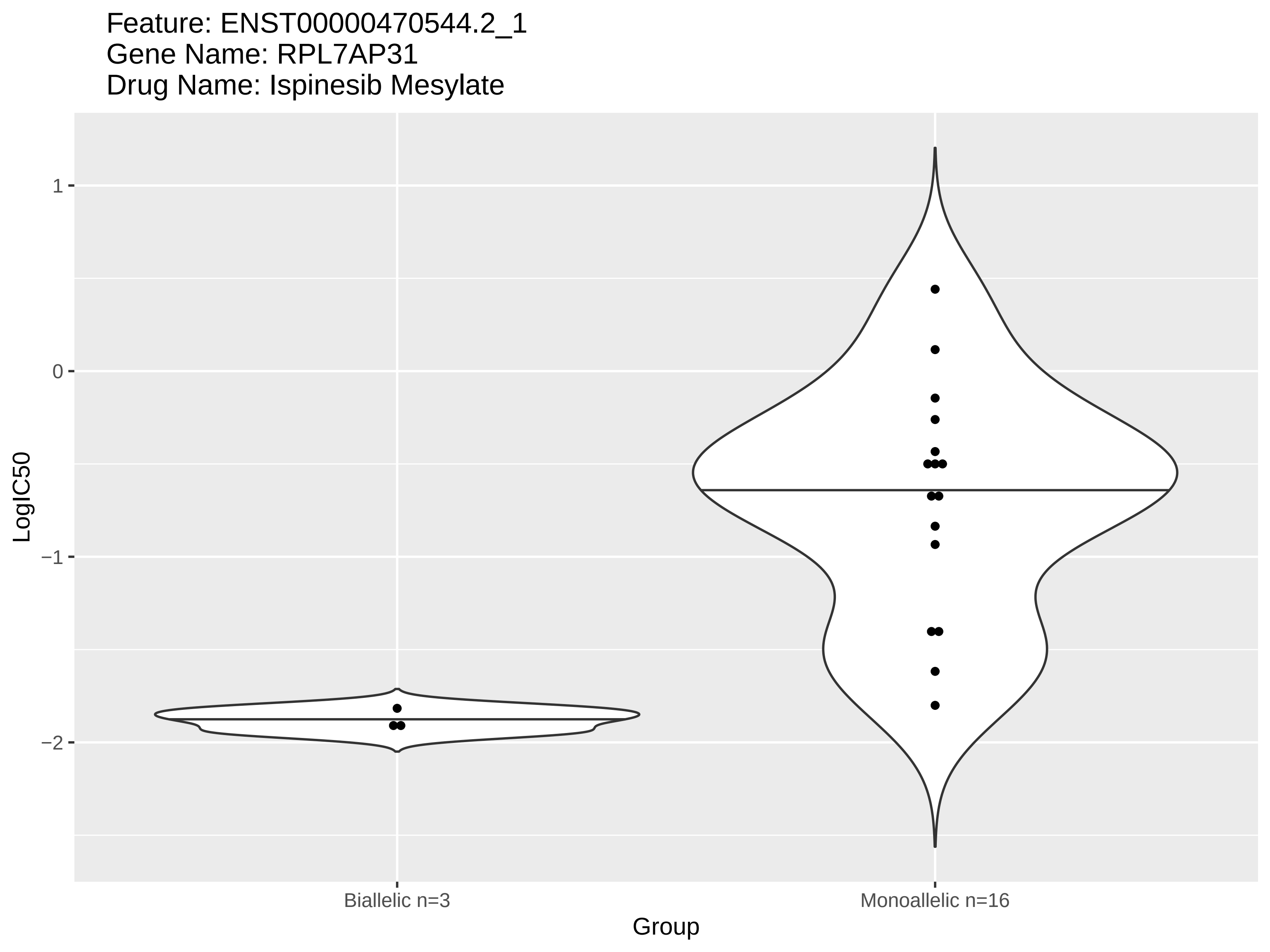
Feature: ENST00000470544.2_1
Gene Name: RPL7AP31
Drug Name: Ispinesib Mesylate
LogIC50
1
0
−1
−2
Biallelic n=3
Monoallelic n=16
Group

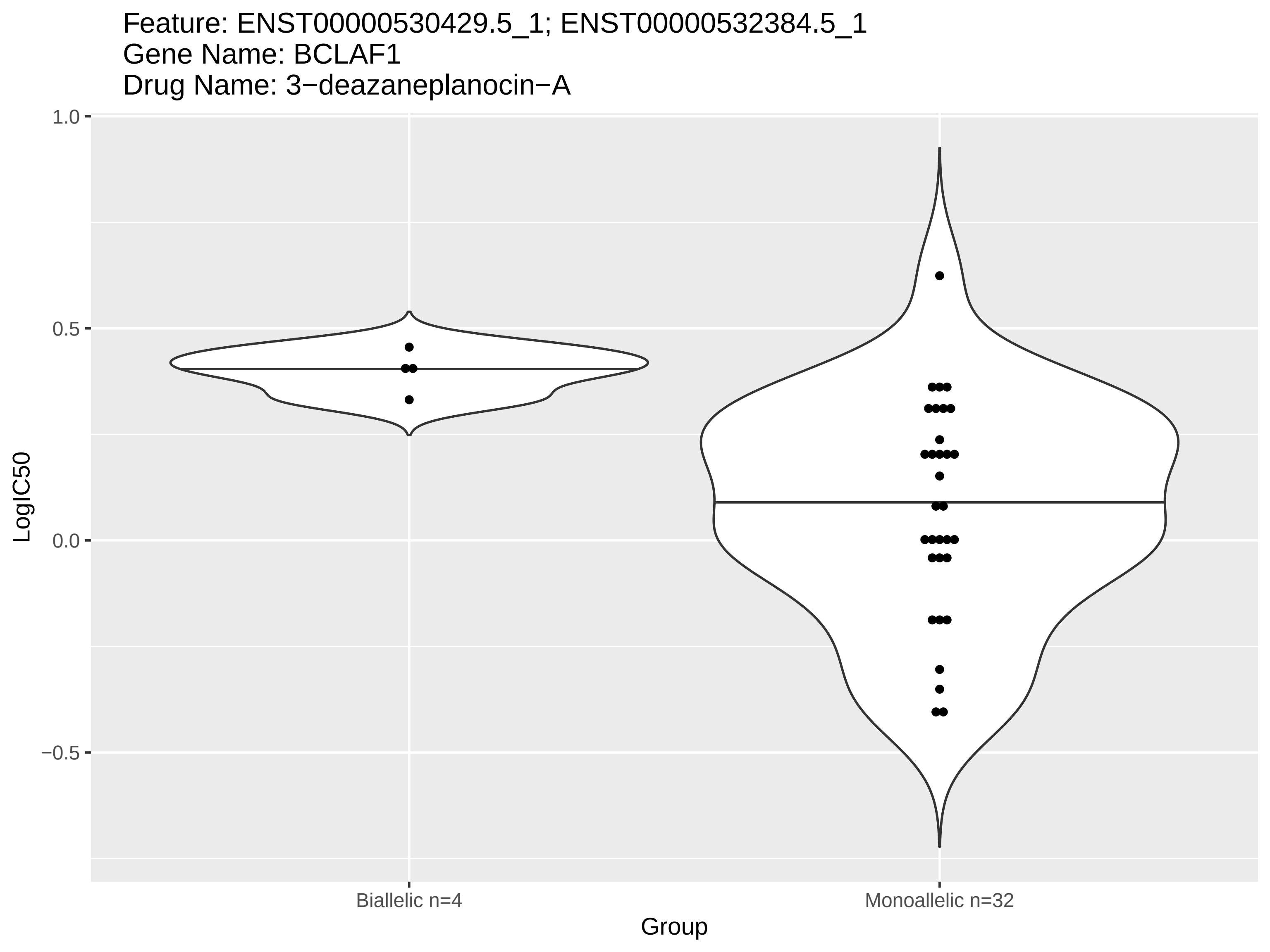

Feature: ENST00000530429.5_1; ENST00000532384.5_1
Gene Name: BCLAF1
Drug Name: 3−deazaneplanocin−A
1.0
0.5
0.0
−0.5
LogIC50
Biallelic n=4
Monoallelic n=32
Group

Feature: ENST00000392348.6_1; ENST00000529826.5_1; ENST00000628517.2_1
Gene Name: BCLAF1
Drug Name: BAY−11−7082

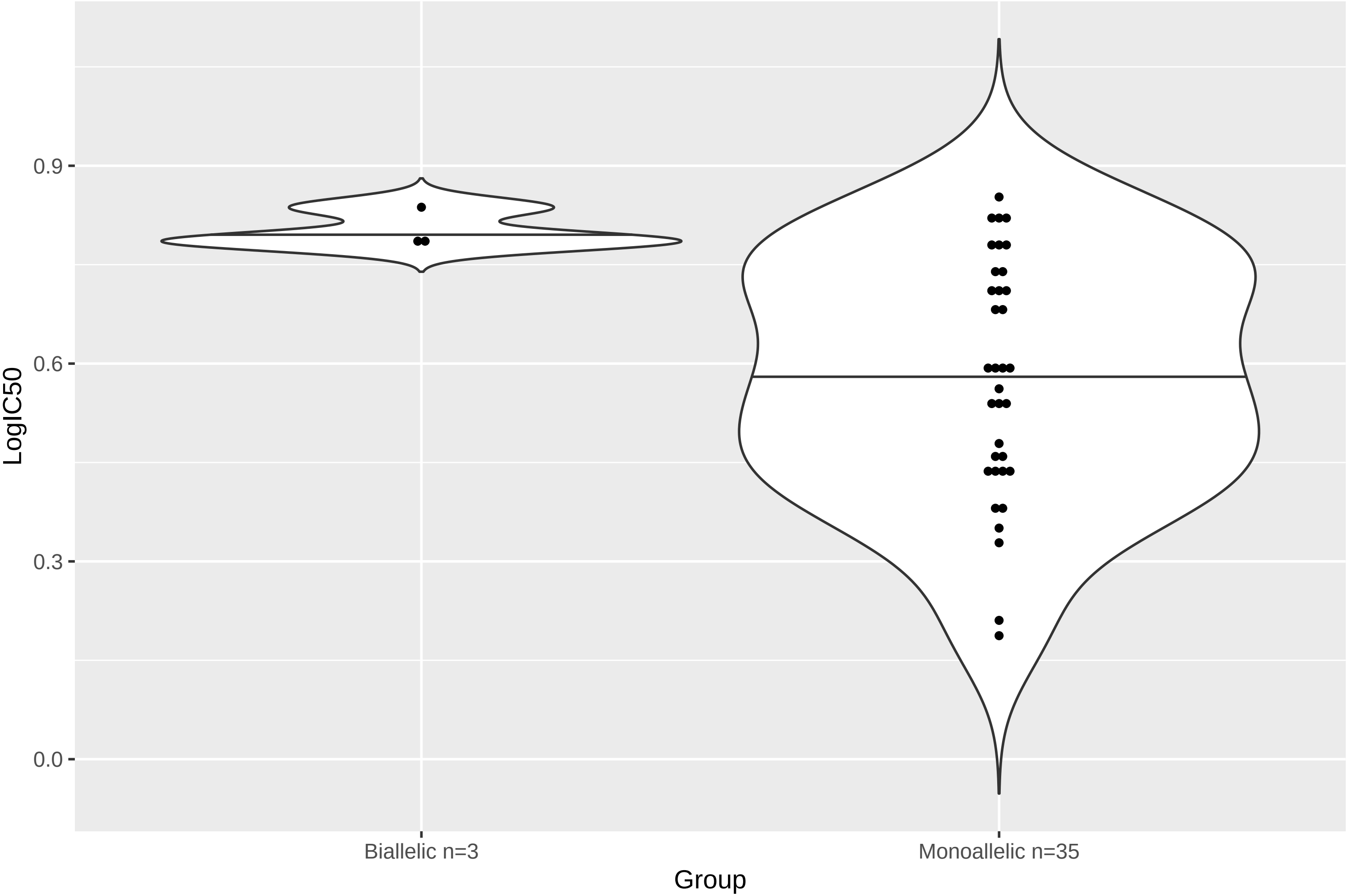

Feature: ENST00000527547.5_1; ENST00000533415.5_1
Gene Name: CDC27
Drug Name: SN−38

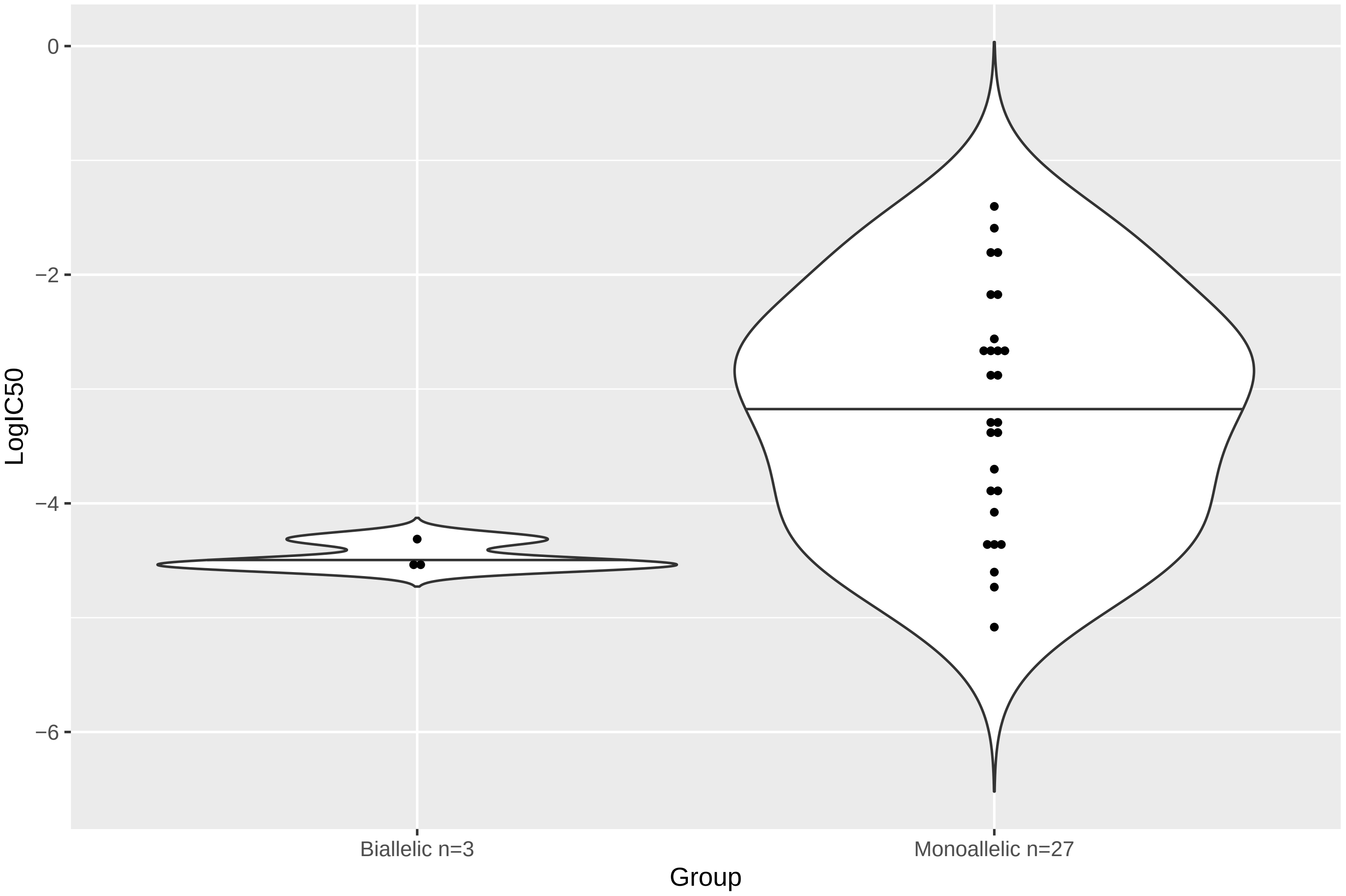

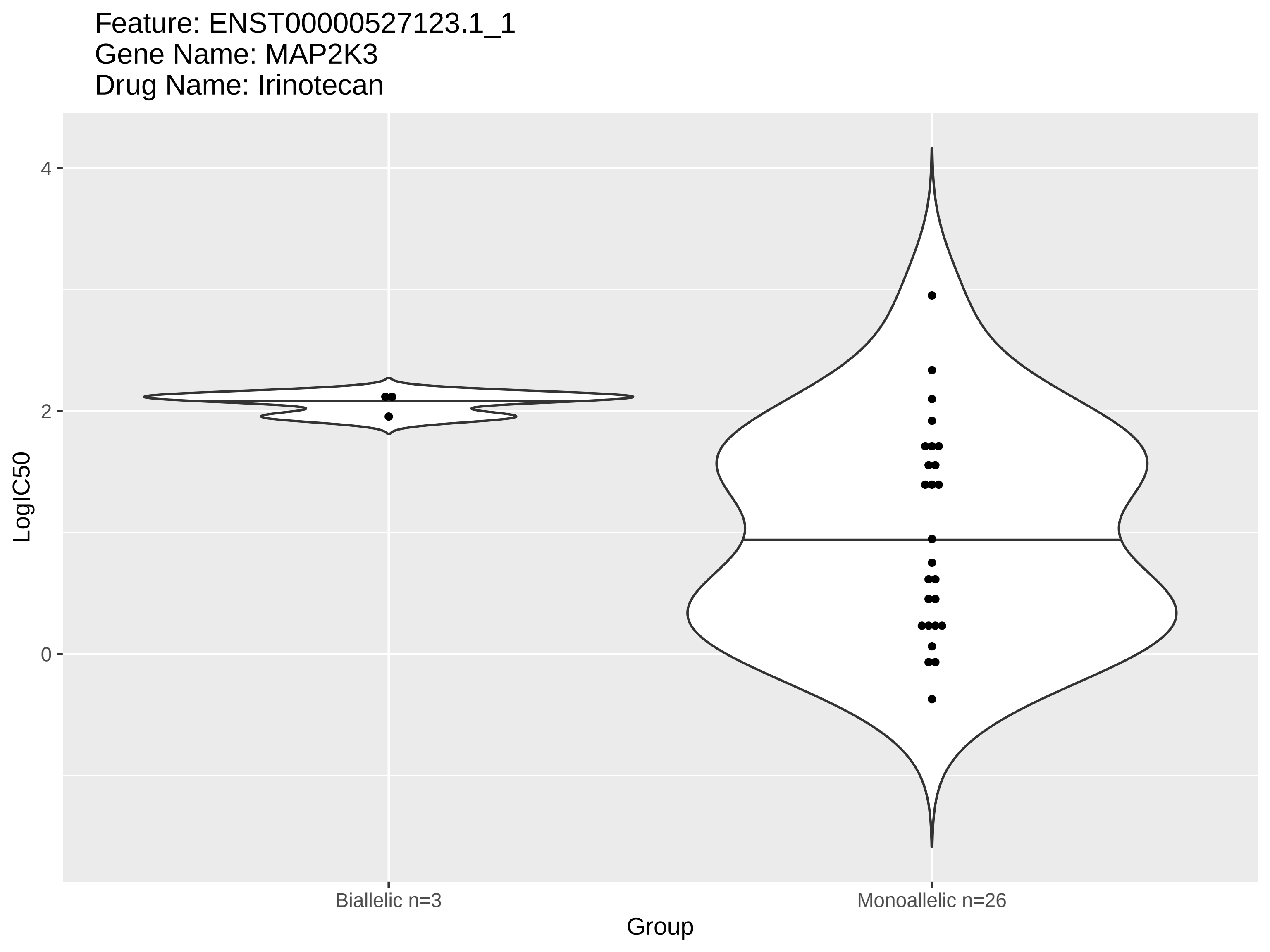
Feature: ENST00000527123.1_1
Gene Name: MAP2K3
Drug Name: Irinotecan
LogIC50
4
2
0
Biallelic n=3
Monoallelic n=26
Group

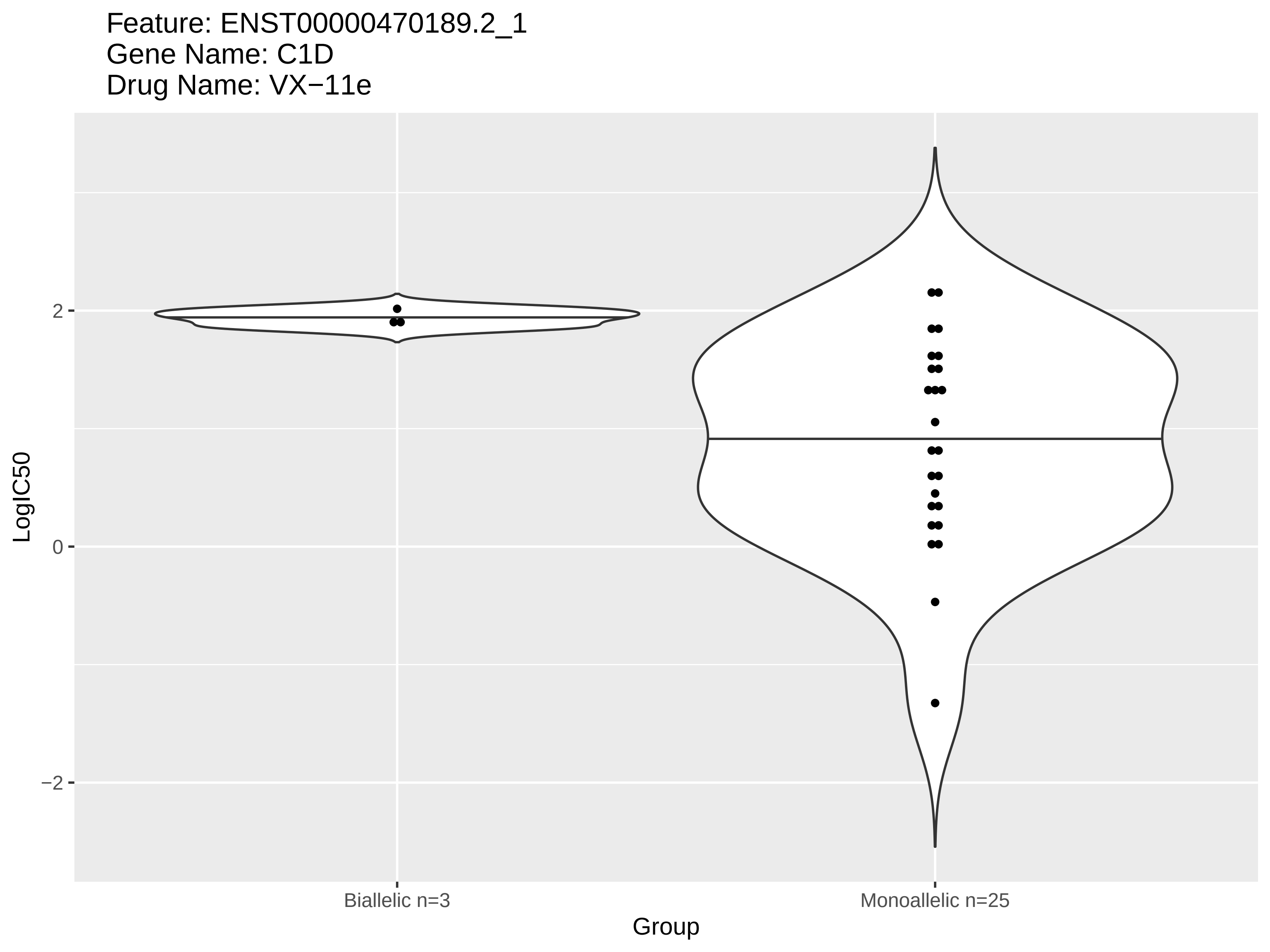

Feature: ENST00000470189.2_1
Gene Name: C1D
Drug Name: VX−11e
LogIC50
2
0
−2
Biallelic n=3
Monoallelic n=25
Group

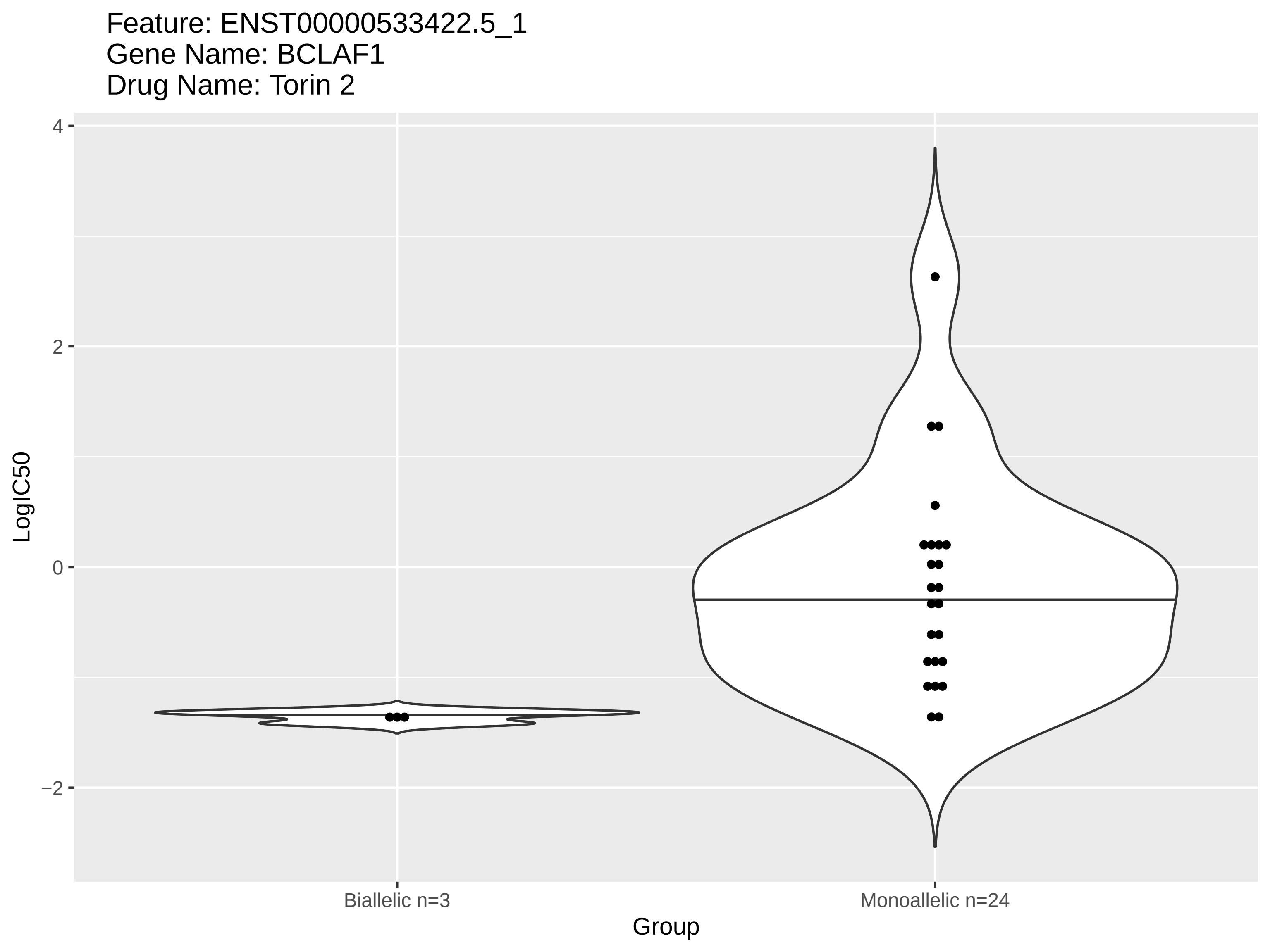
Feature: ENST00000533422.5_1
Gene Name: BCLAF1
Drug Name: Torin 2
LogIC50
4
2
0
−2
Biallelic n=3
Monoallelic n=24
Group

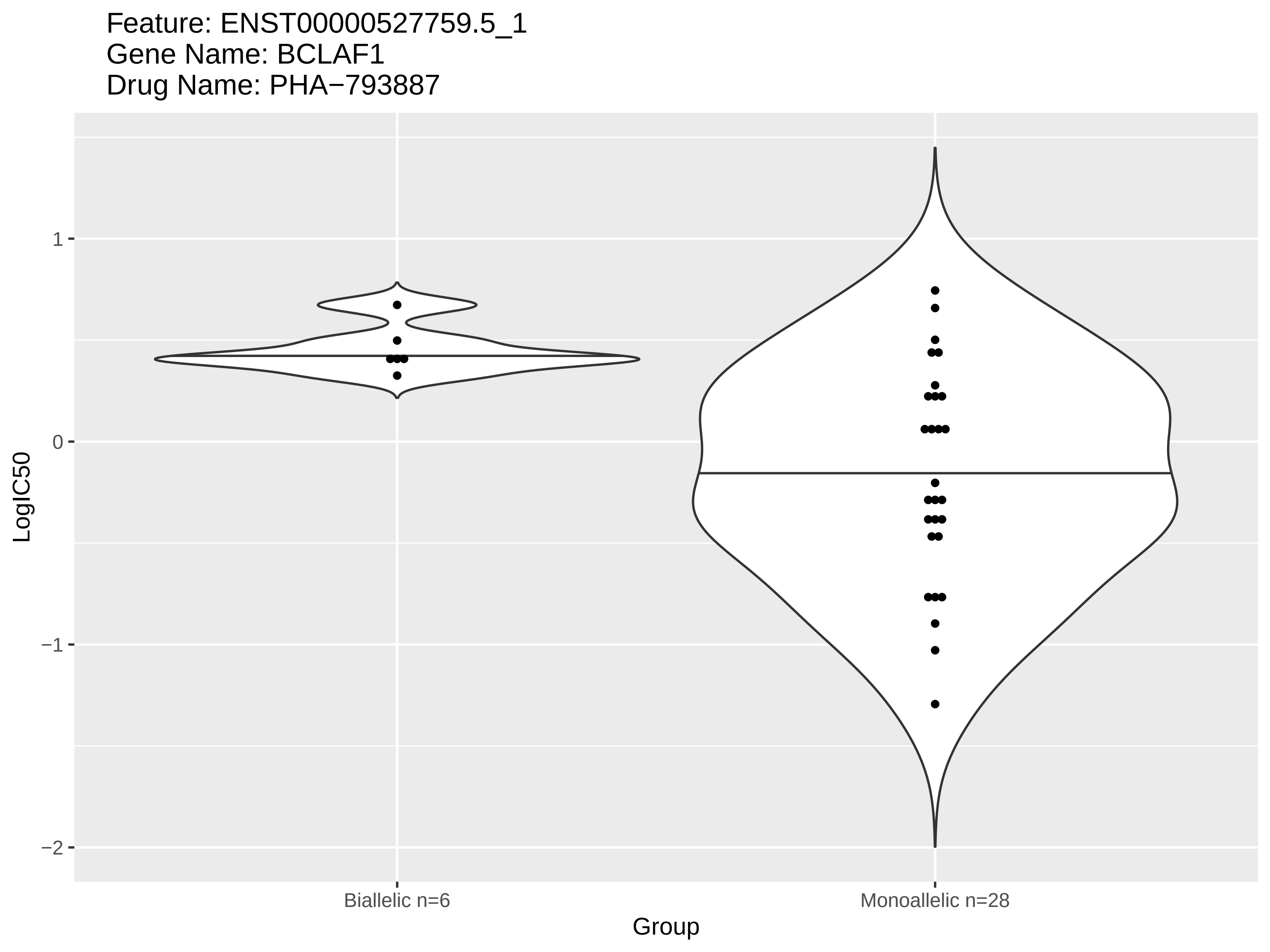
Feature: ENST00000527759.5_1
Gene Name: BCLAF1
Drug Name: PHA−793887
LogIC50
1
0
−1
−2
Biallelic n=6
Monoallelic n=28
Group

Feature: ENST00000392348.6_1; ENST00000529826.5_1; ENST00000628517.2_1
Gene Name: BCLAF1
Drug Name: genz−644282

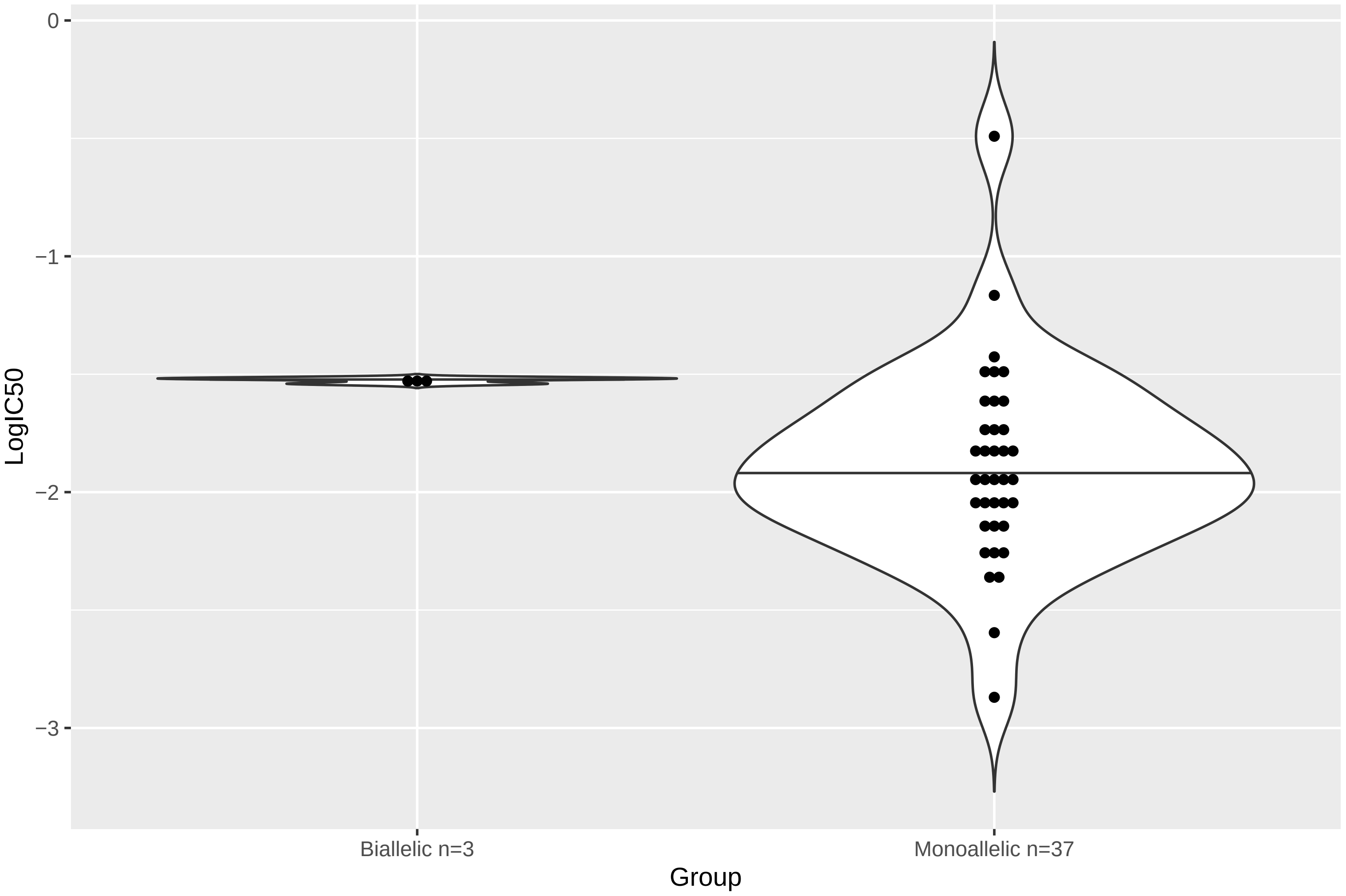

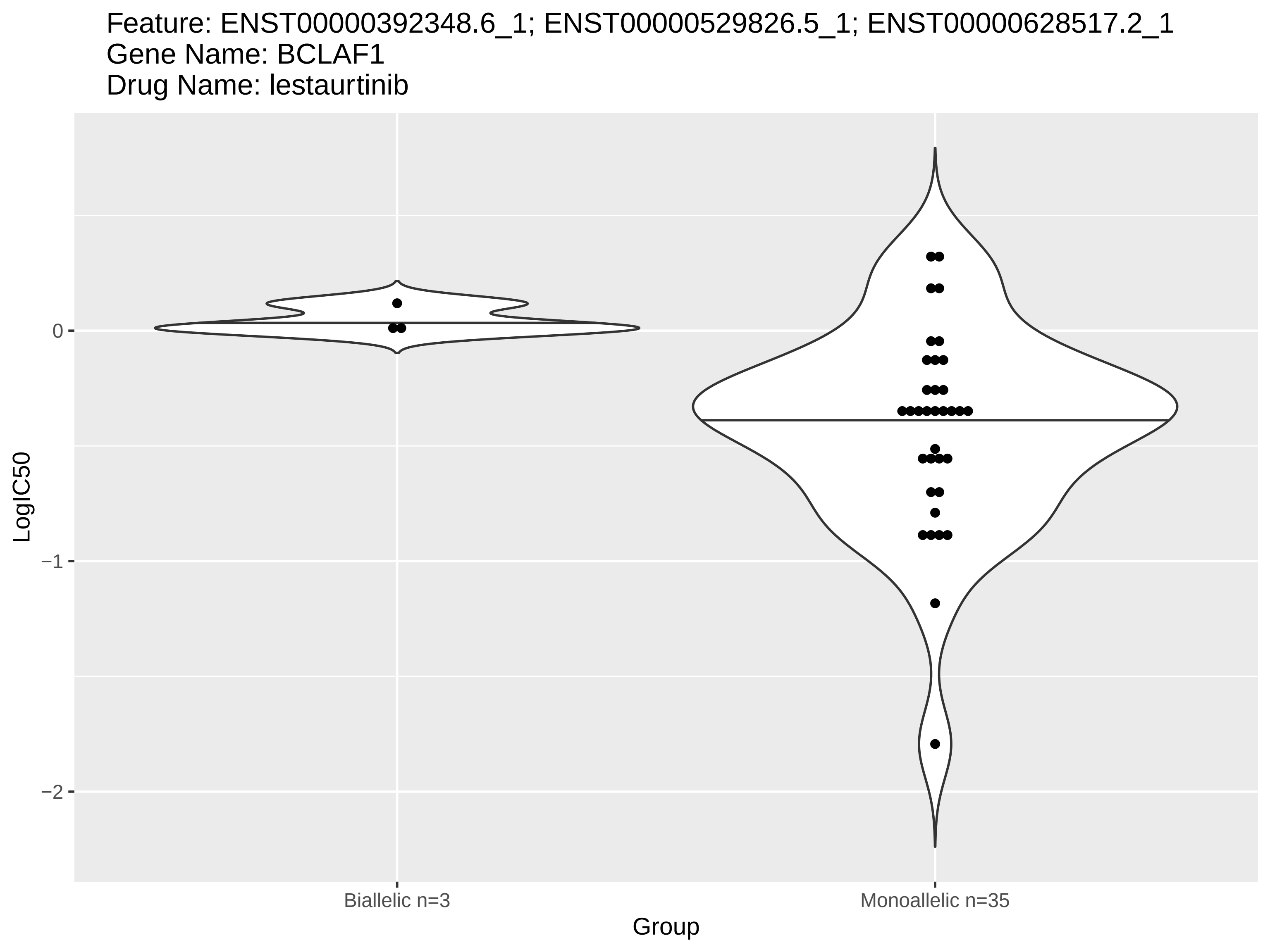

Feature: ENST00000392348.6_1; ENST00000529826.5_1; ENST00000628517.2_1
Gene Name: BCLAF1
Drug Name: lestaurtinib
0
−1
−2
LogIC50
Biallelic n=3
Monoallelic n=35
Group

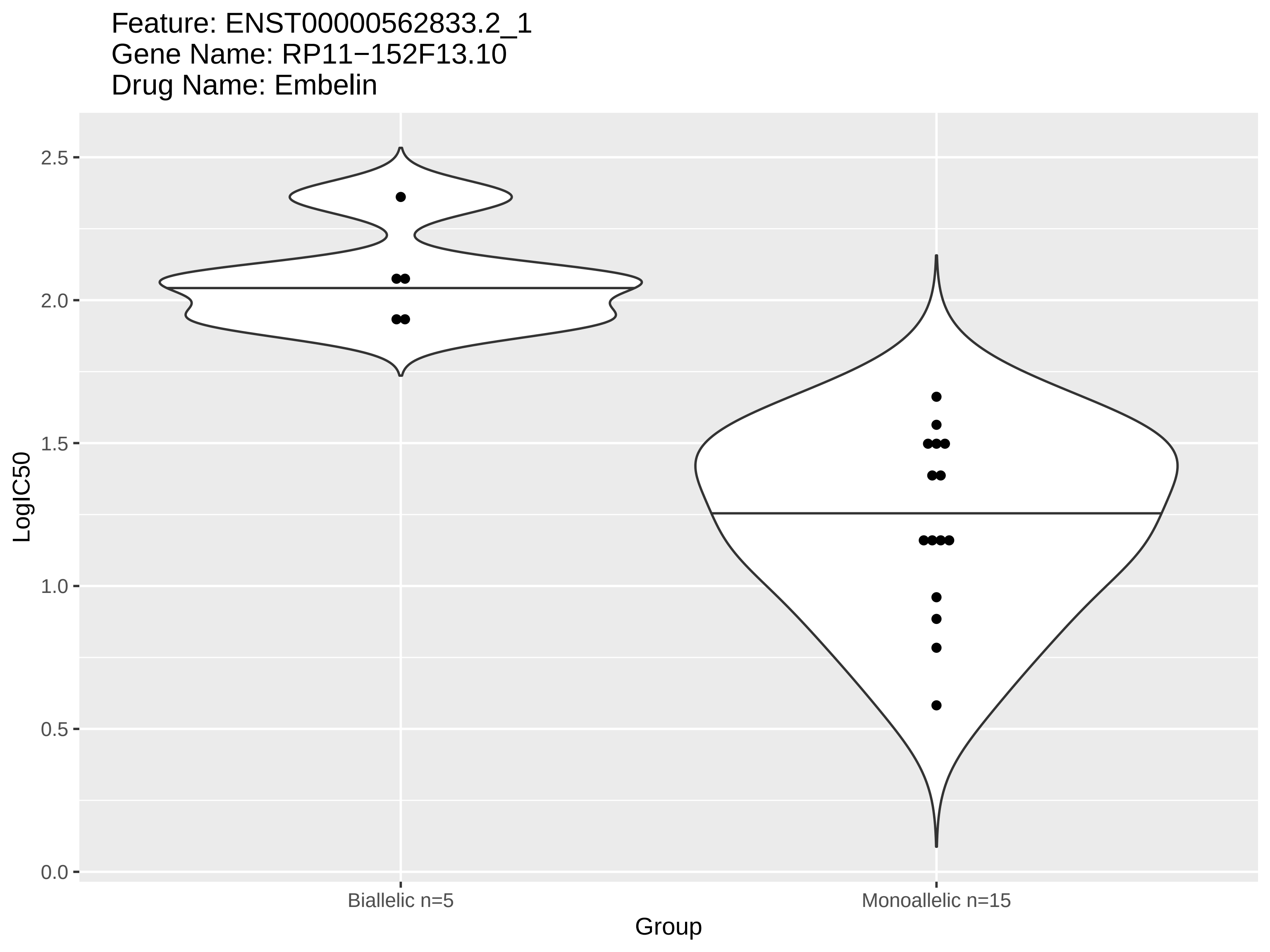
Feature: ENST00000562833.2_1
Gene Name: RP11−152F13.10
Drug Name: Embelin
LogIC50
2.5
2.0
1.5
1.0
0.5
0.0
Biallelic n=5
Monoallelic n=15
Group

Feature: ENST00000533422.5_1
Gene Name: BCLAF1
Drug Name: BIBF−1120

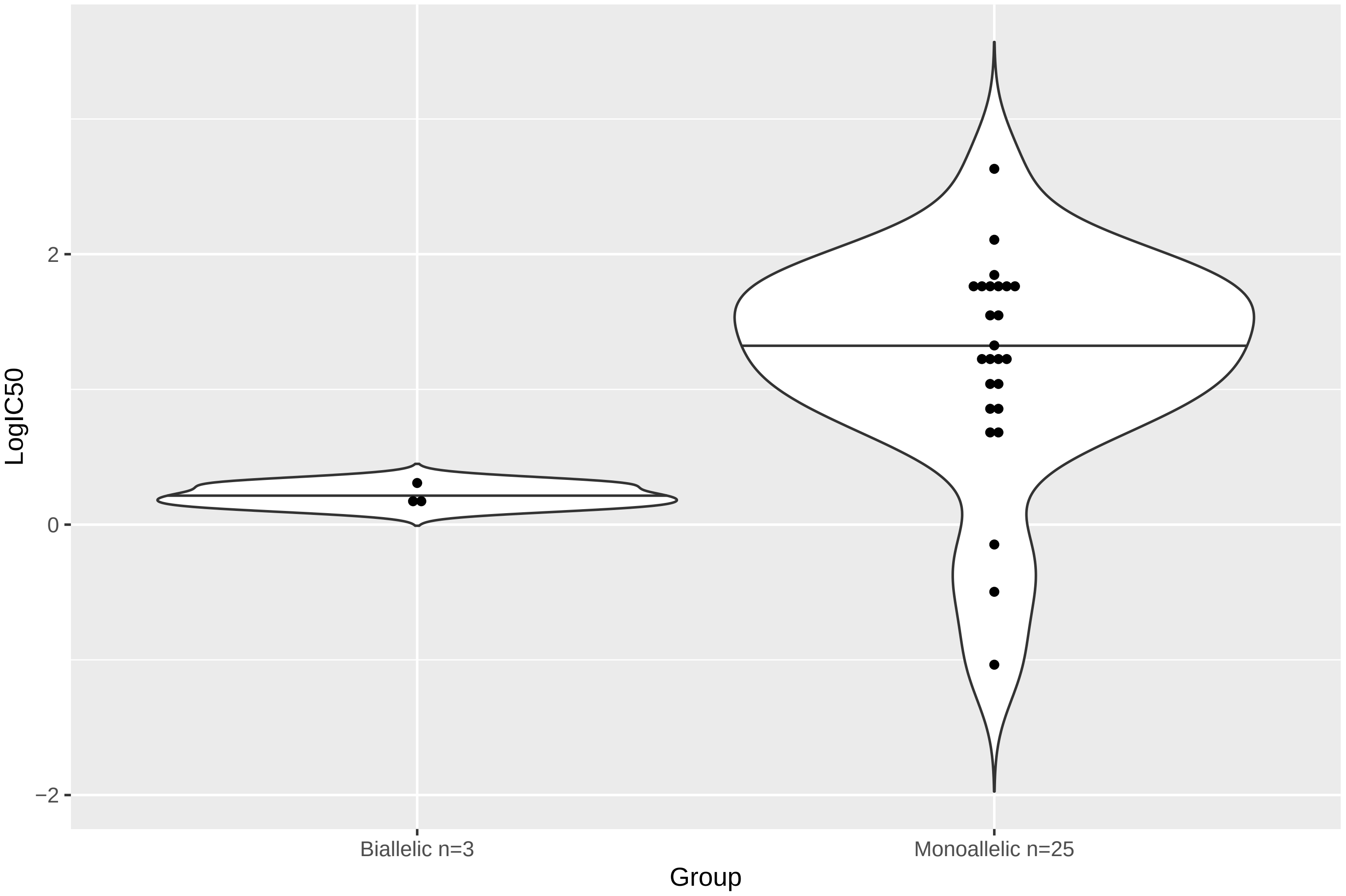

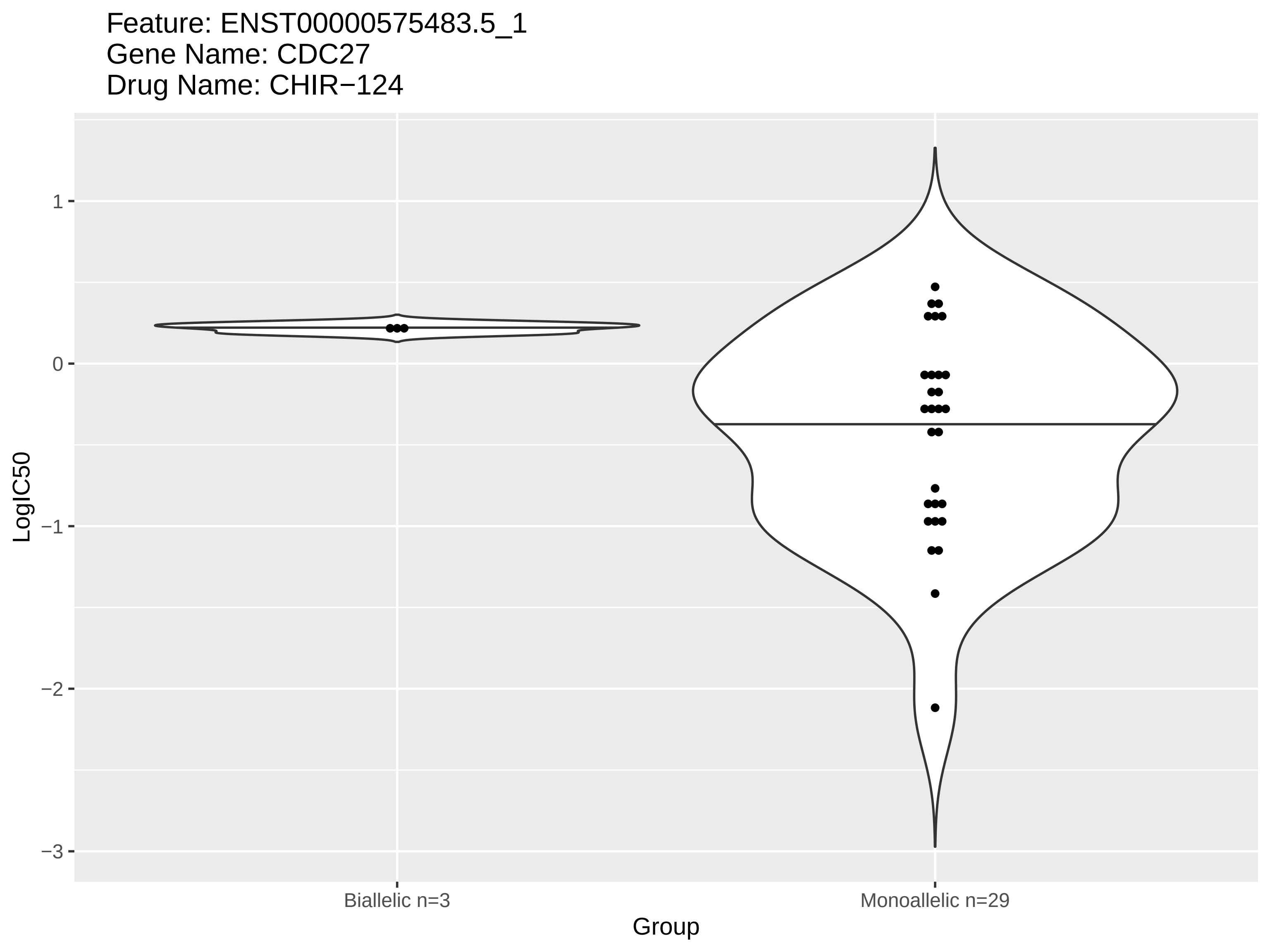
Feature: ENST00000575483.5_1
Gene Name: CDC27
Drug Name: CHIR−124
LogIC50
1
0
−1
−2
−3
Biallelic n=3
Monoallelic n=29
Group

Feature: ENST00000526866.5_1; ENST00000532893.5_1
Gene Name: CDC27
Drug Name: LY456236

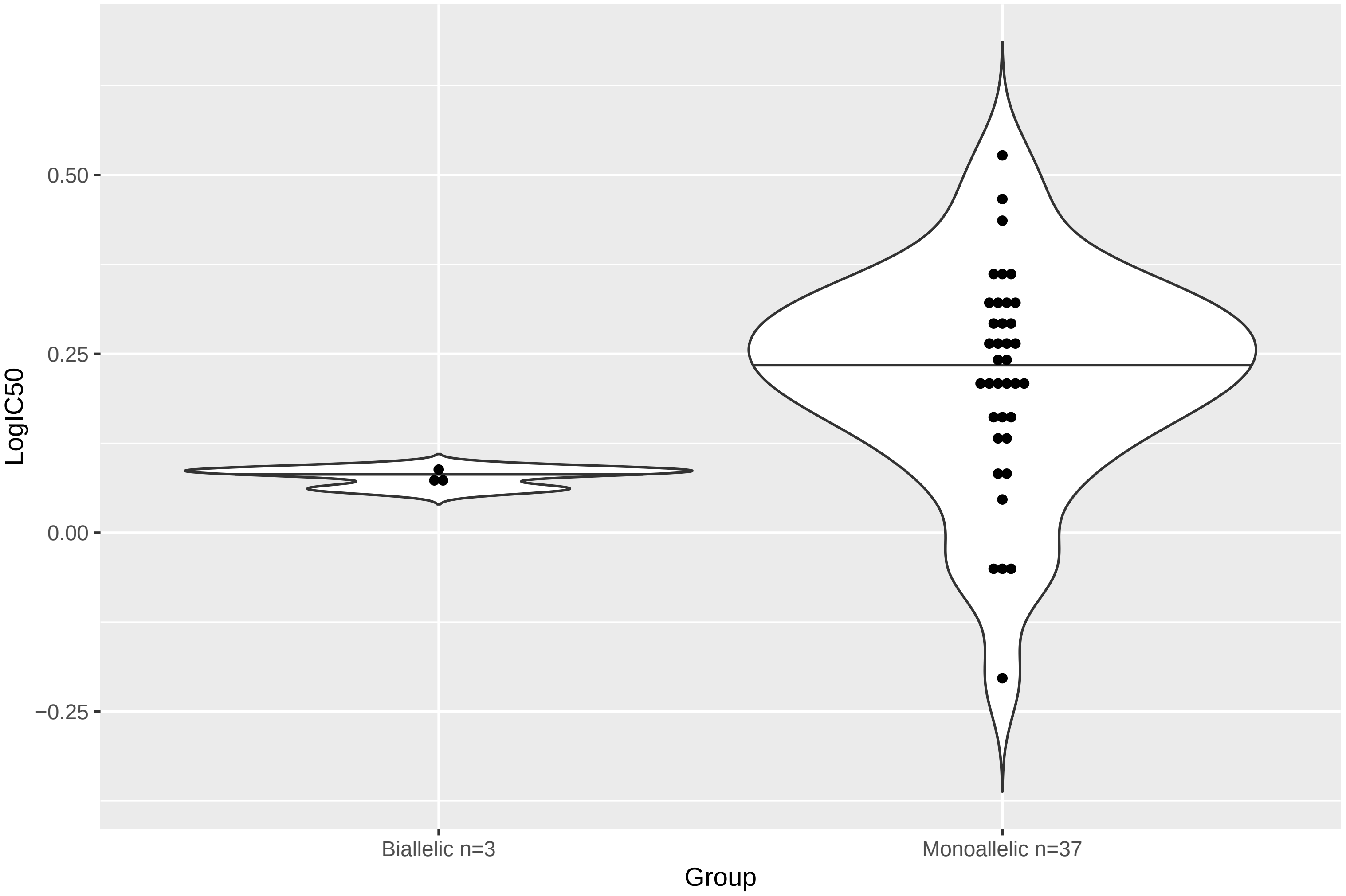

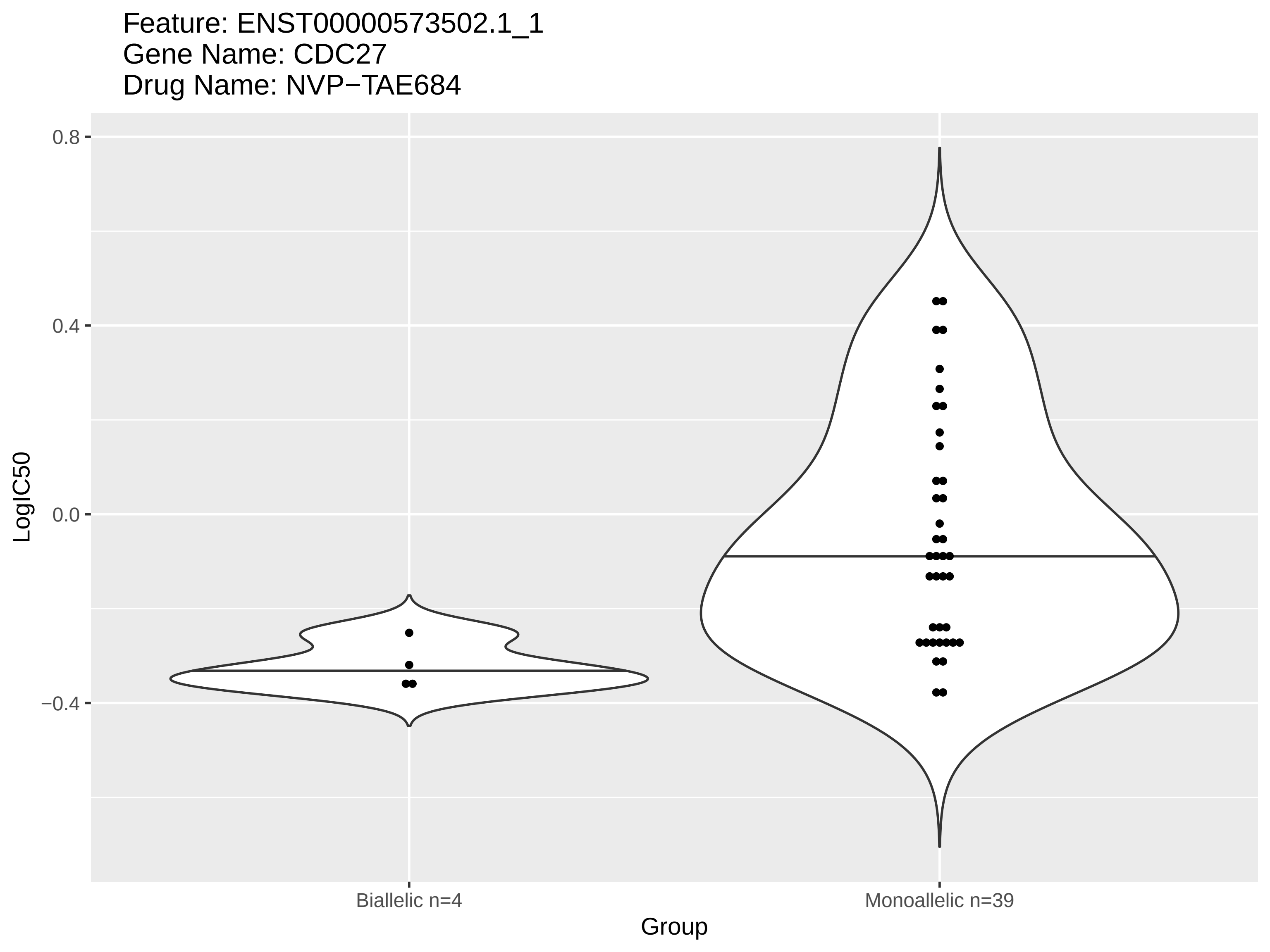

Feature: ENST00000573502.1_1
Gene Name: CDC27
Drug Name: NVP−TAE684
0.8
0.4
0.0
−0.4
LogIC50
Biallelic n=4
Monoallelic n=39
Group

Feature: ENST00000533077.5_1
Gene Name: GRK2
Drug Name: I−BET−762

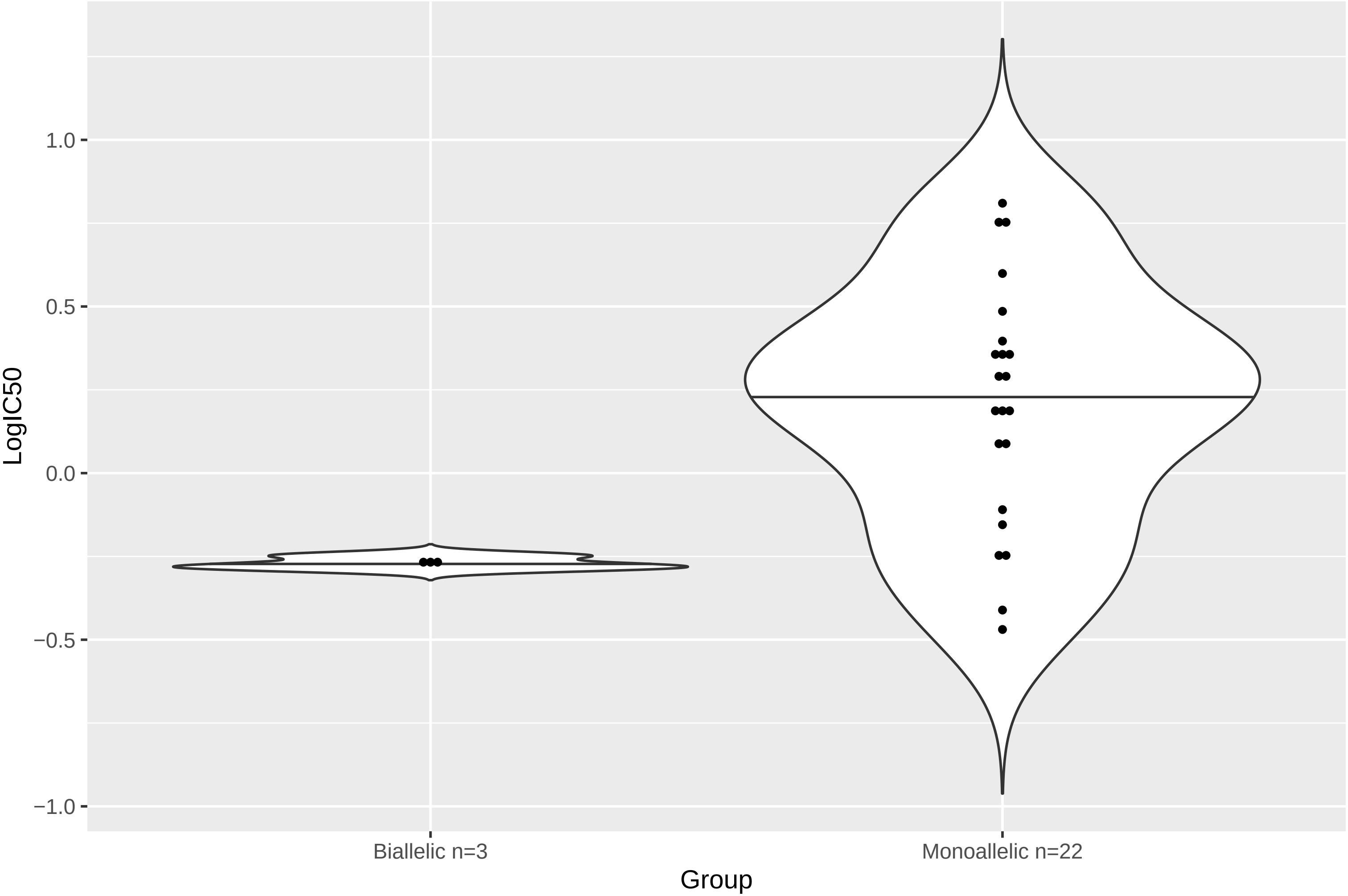

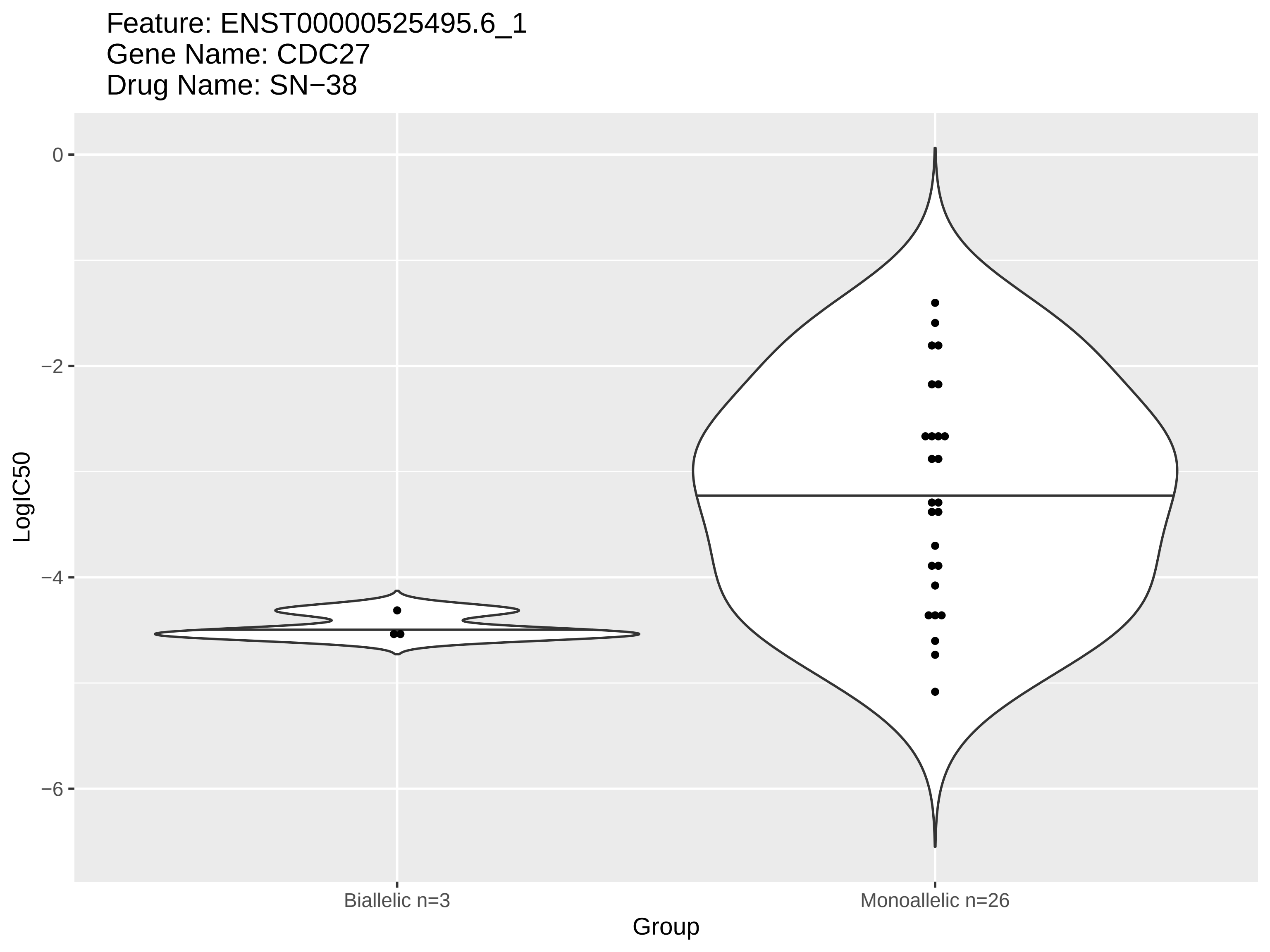
Feature: ENST00000525495.6_1
Gene Name: CDC27
Drug Name: SN−38
0
−2
−4
−6
LogIC50
Biallelic n=3
Monoallelic n=26
Group

Feature: ENST00000343811.9_1
Gene Name: MROH8
Drug Name: poziotinib

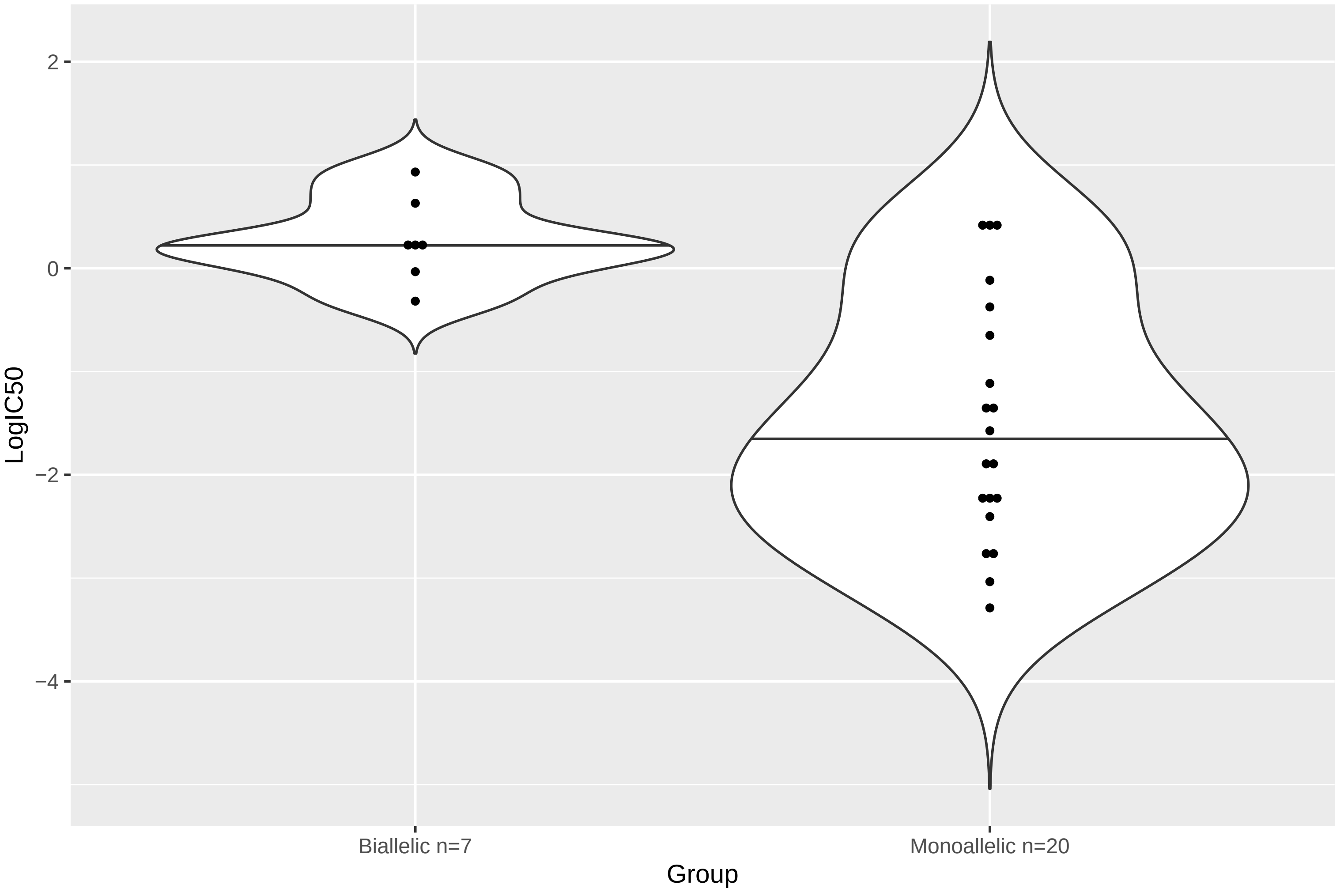

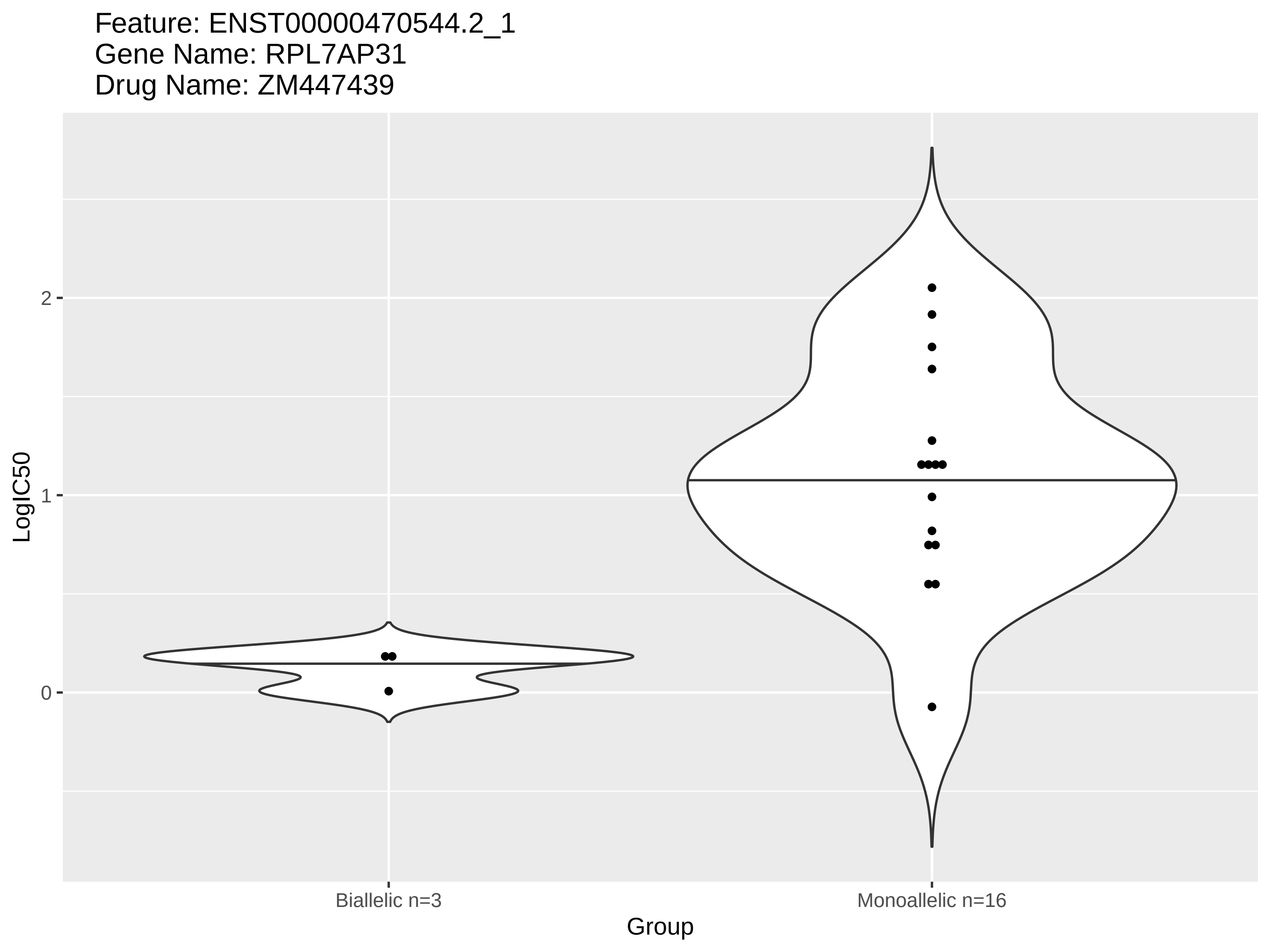

Feature: ENST00000470544.2_1
Gene Name: RPL7AP31
Drug Name: ZM447439
LogIC50
2
1
0
Biallelic n=3
Monoallelic n=16
Group

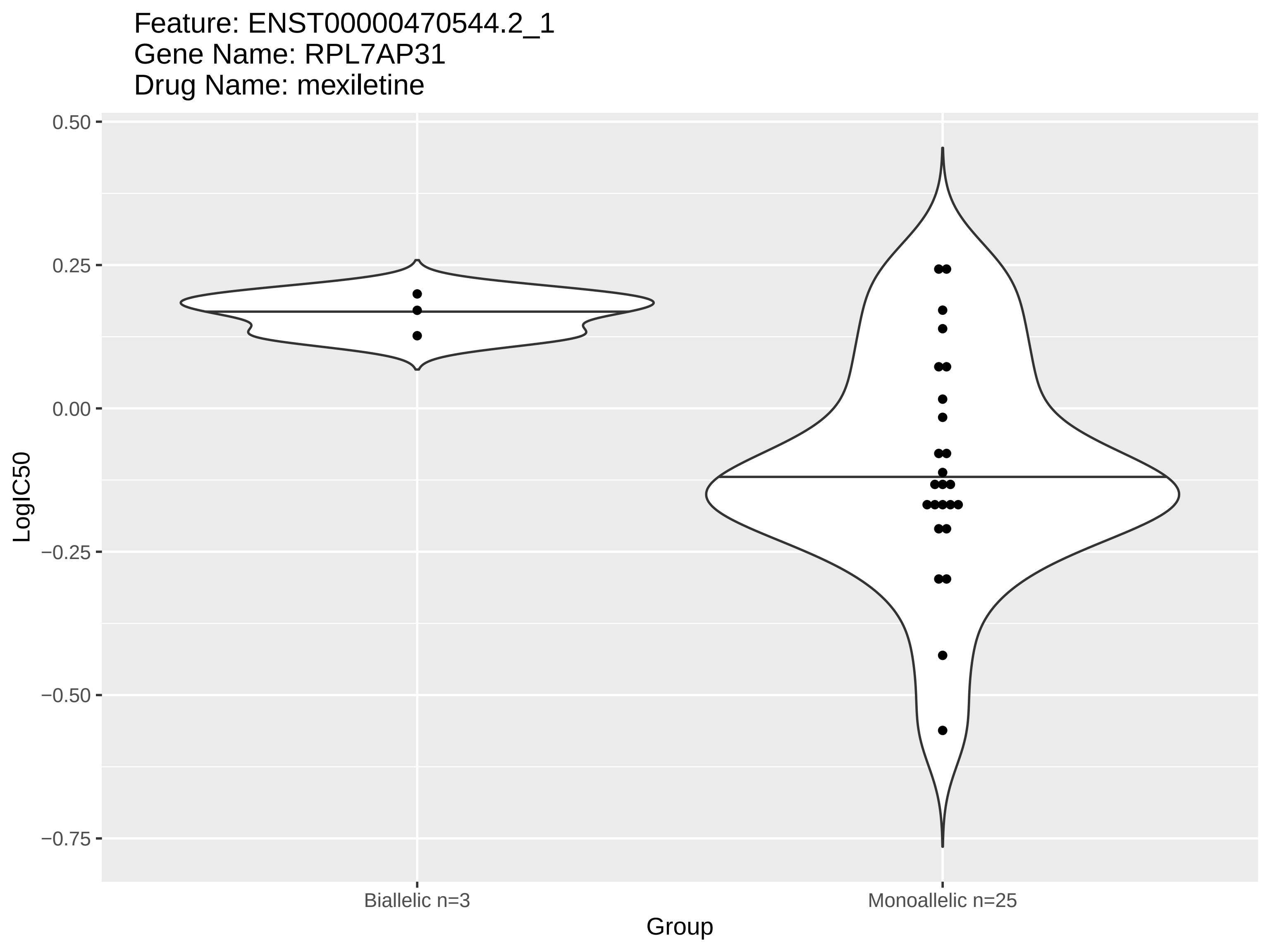

Feature: ENST00000470544.2_1
Gene Name: RPL7AP31
Drug Name: mexiletine
0.50
0.25
0.00
−0.25
−0.50
−0.75
LogIC50
Biallelic n=3
Monoalleli c n=25
Group

Feature: ENST00000392348.6_1; ENST00000529826.5_1; ENST00000628517.2_1
Gene Name: BCLAF1
Drug Name: ARRY−334543

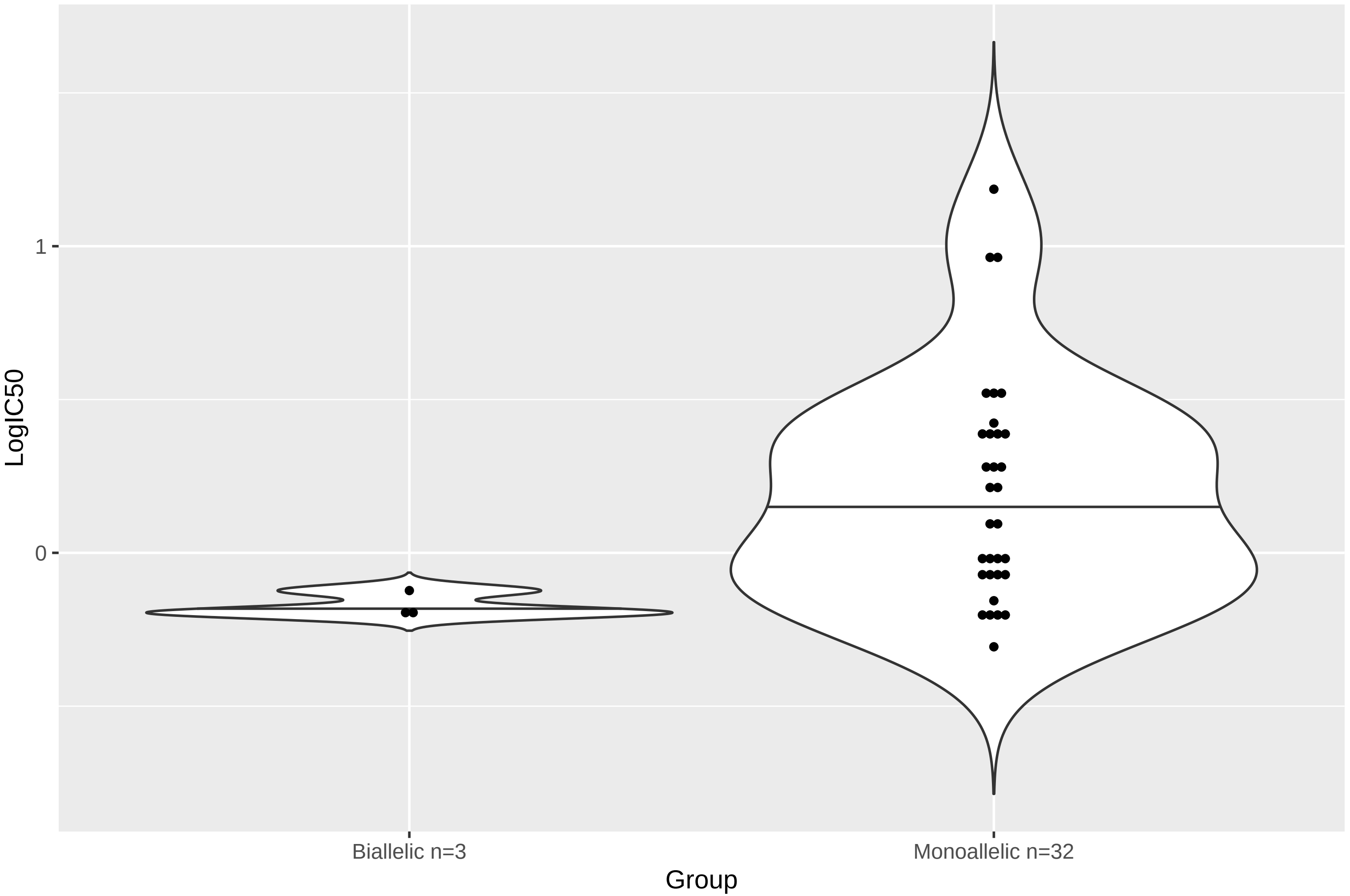

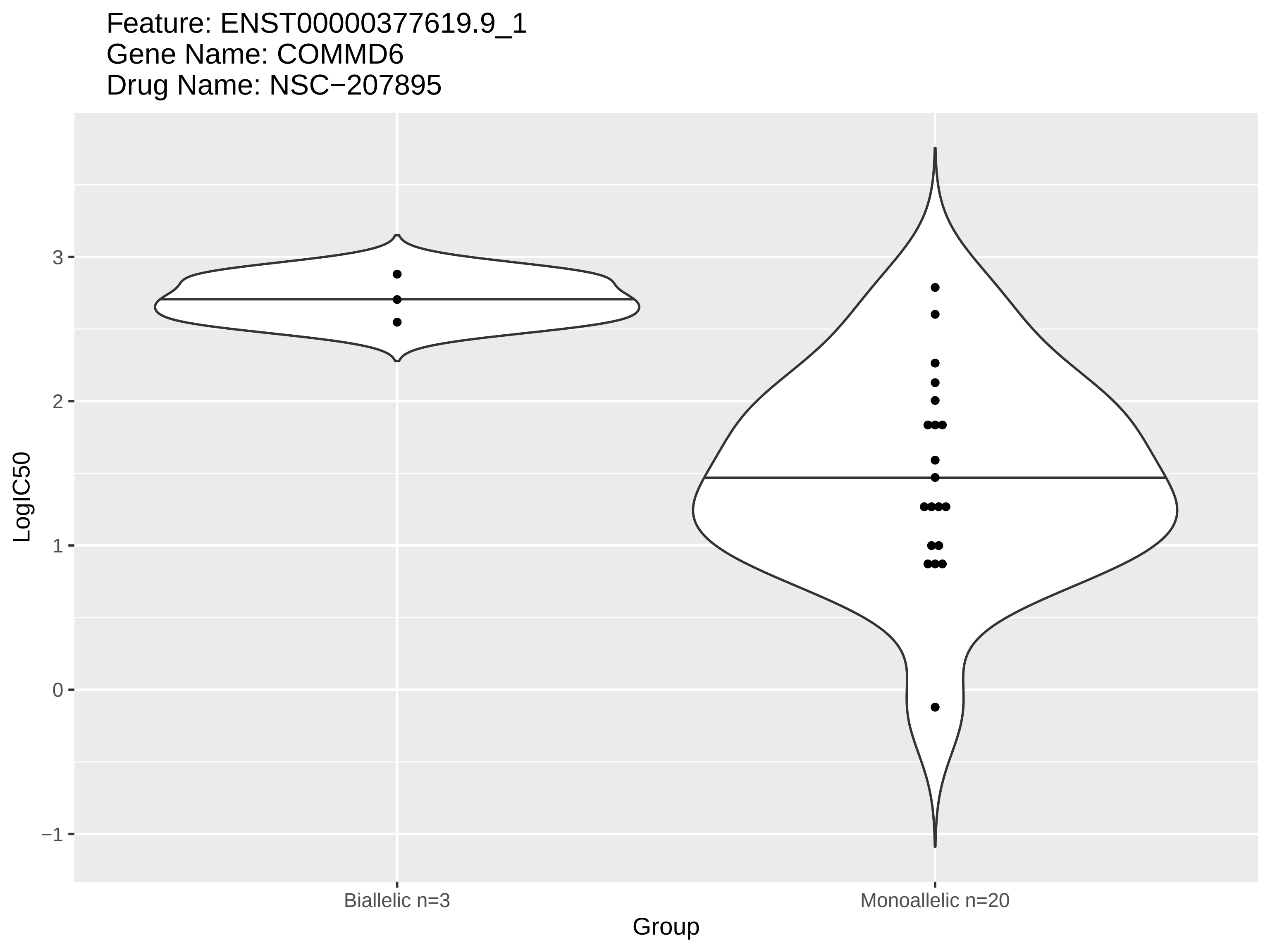
Feature: ENST00000377619.9_1
Gene Name: COMMD6
Drug Name: NSC−207895
LogIC50
3
2
1
0
−1
Biallelic n=3
Monoallelic n=20
Group

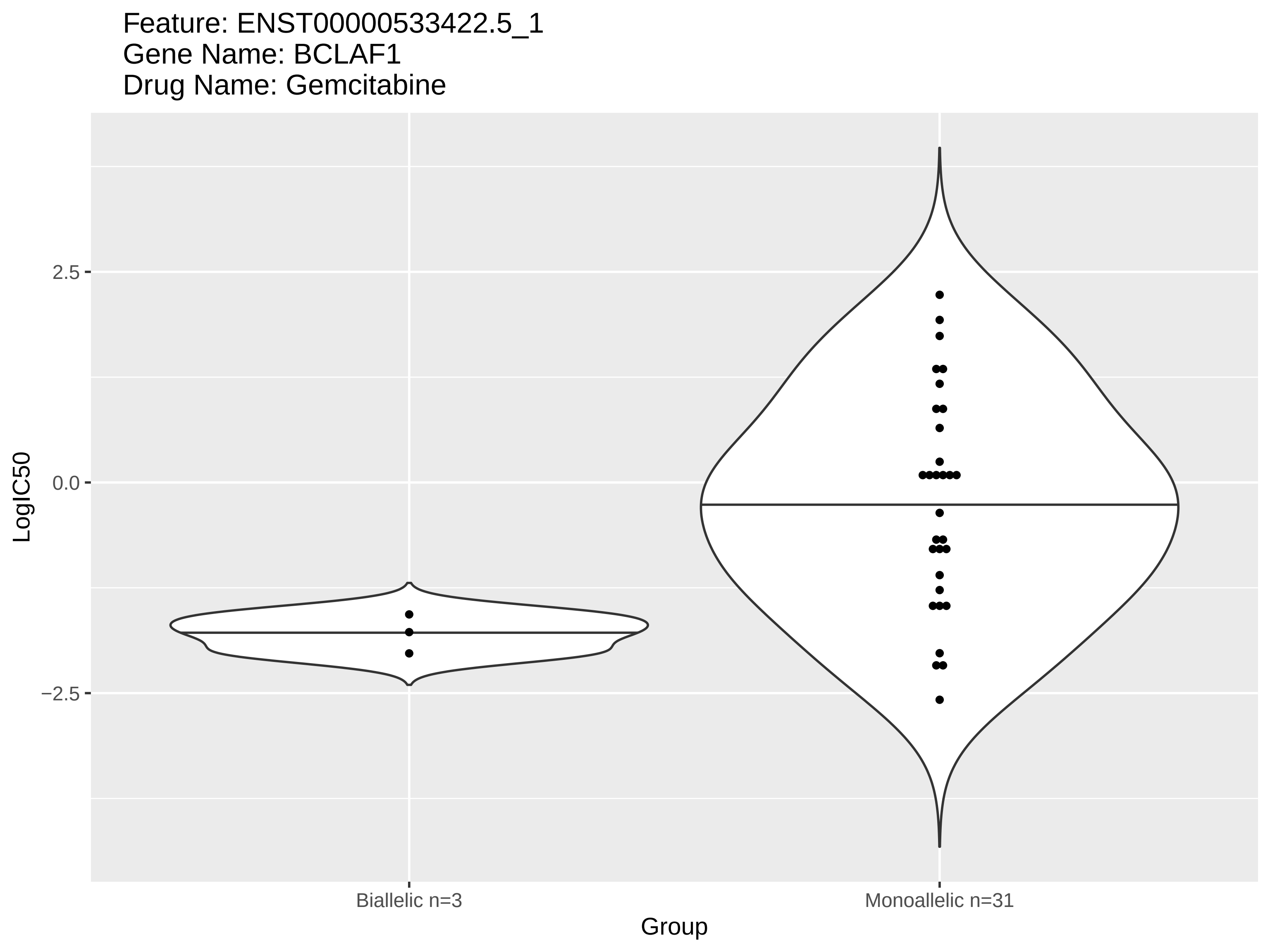

Feature: ENST00000533422.5_1
Gene Name: BCLAF1
Drug Name: Gemcitabine
2.5
0.0
−2.5
LogIC50
Biallelic n=3
Monoallelic n=31
Group

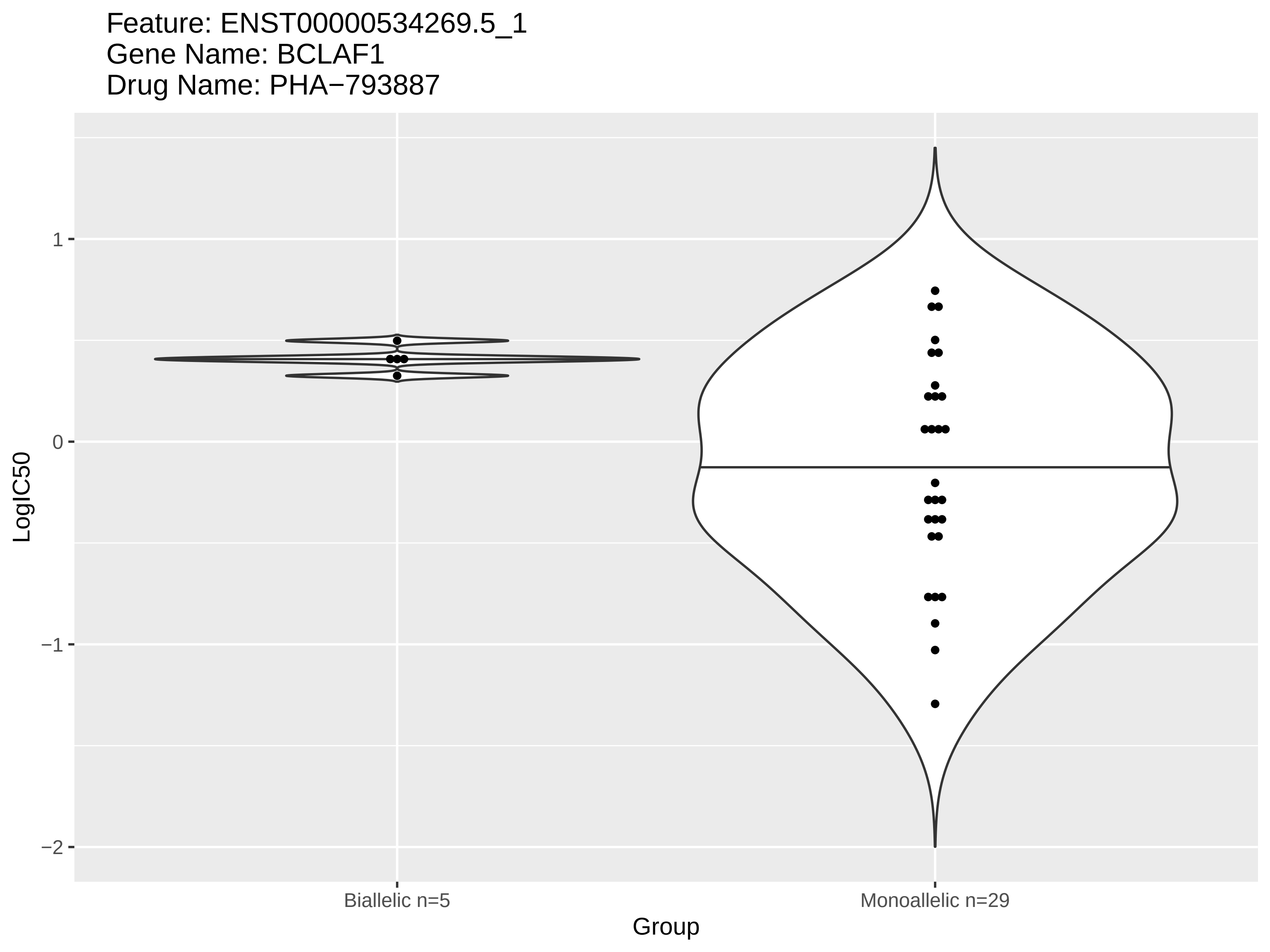
Feature: ENST00000534269.5_1
Gene Name: BCLAF1
Drug Name: PHA−793887
LogIC50
1
0
−1
−2
Biallelic n=5
Monoallelic n=29
Group

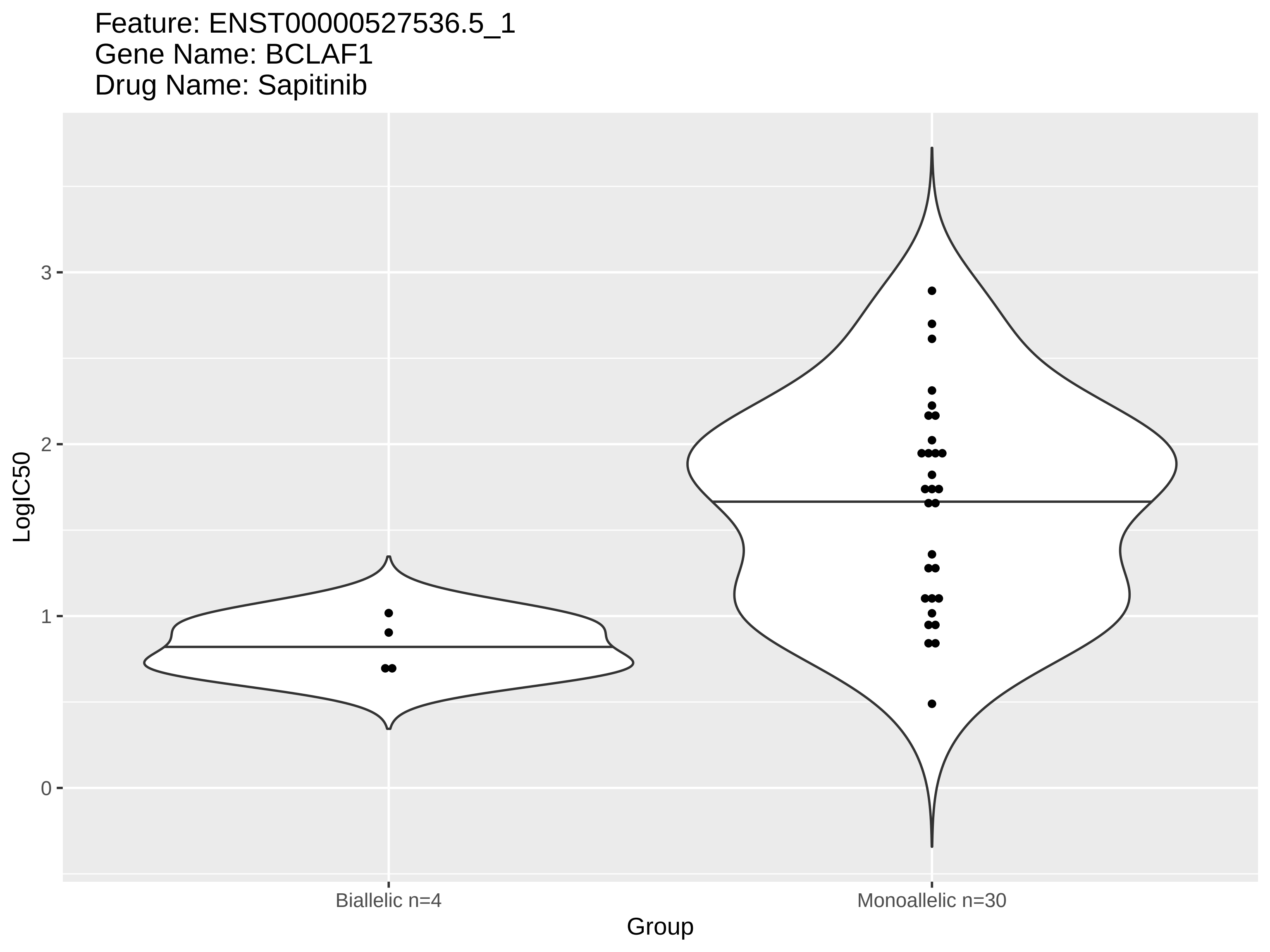
Feature: ENST00000527536.5_1
Gene Name: BCLAF1
Drug Name: Sapitinib
LogIC50
0
1
2
3
Biallelic n=4
Monoallelic n=30
Group

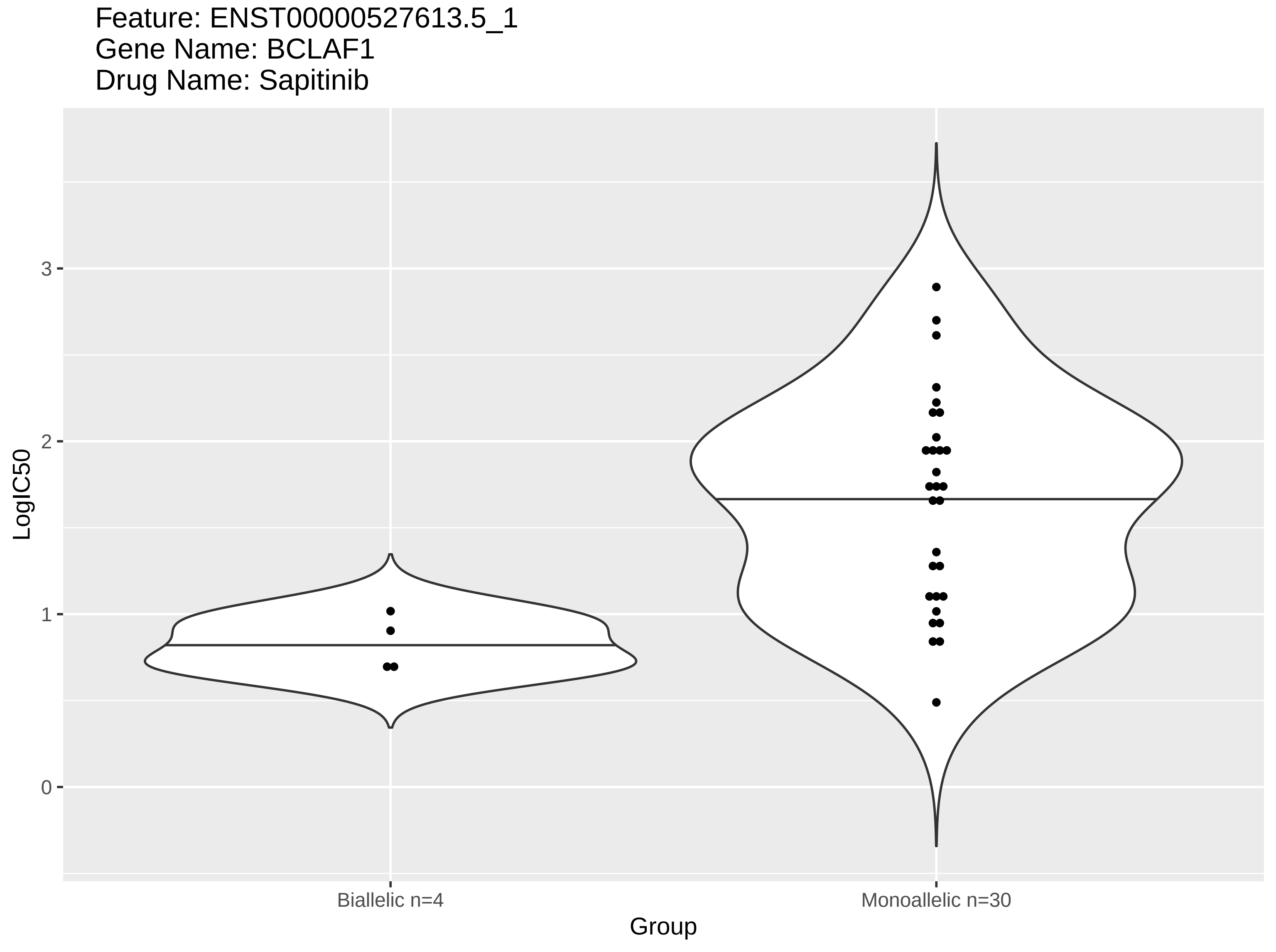
Feature: ENST00000527613.5_1
Gene Name: BCLAF1
Drug Name: Sapitinib
LogIC50
0
1
2
3
Biallelic n=4
Monoallelic n=30
Group

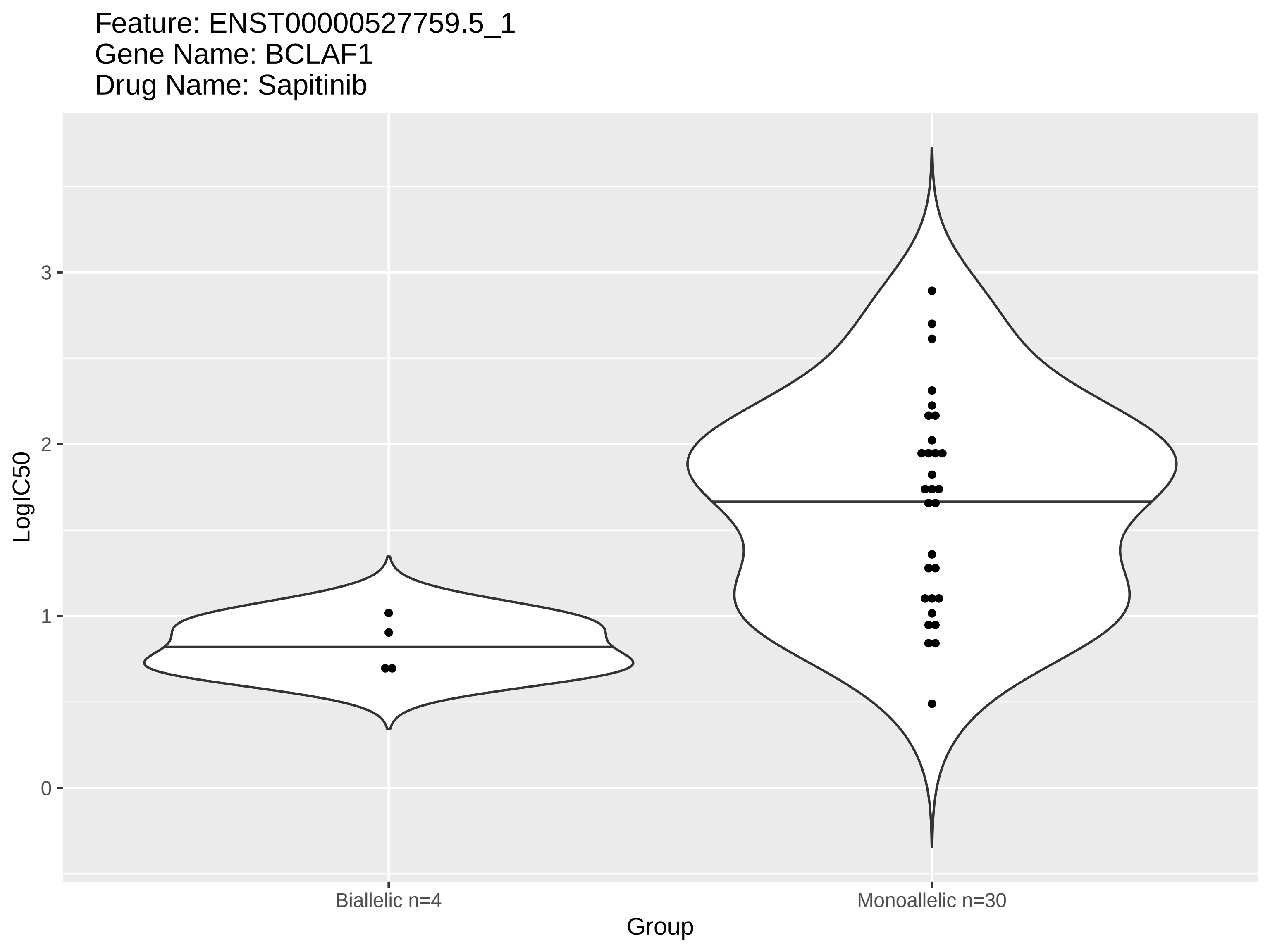
Feature: ENST00000527759.5_1
Gene Name: BCLAF1
Drug Name: Sapitinib
LogIC50
0
1
2
3
Biallelic n=4
Monoallelic n=30
Group

Feature: ENST00000530767.5_1
Gene Name: BCLAF1
Drug Name: Sapitinib

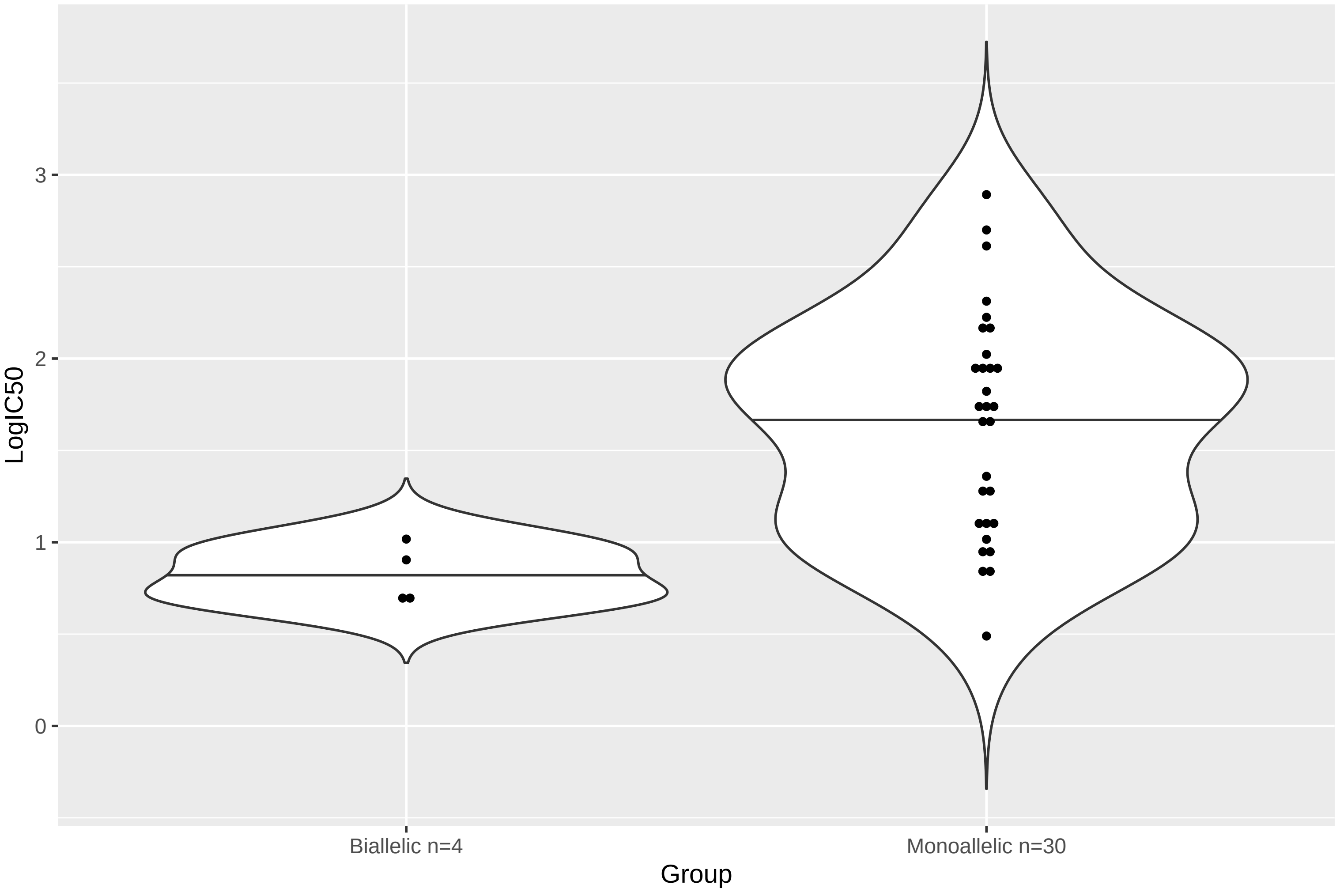

Feature: ENST00000534269.5_1
Gene Name: BCLAF1
Drug Name: Sapitinib

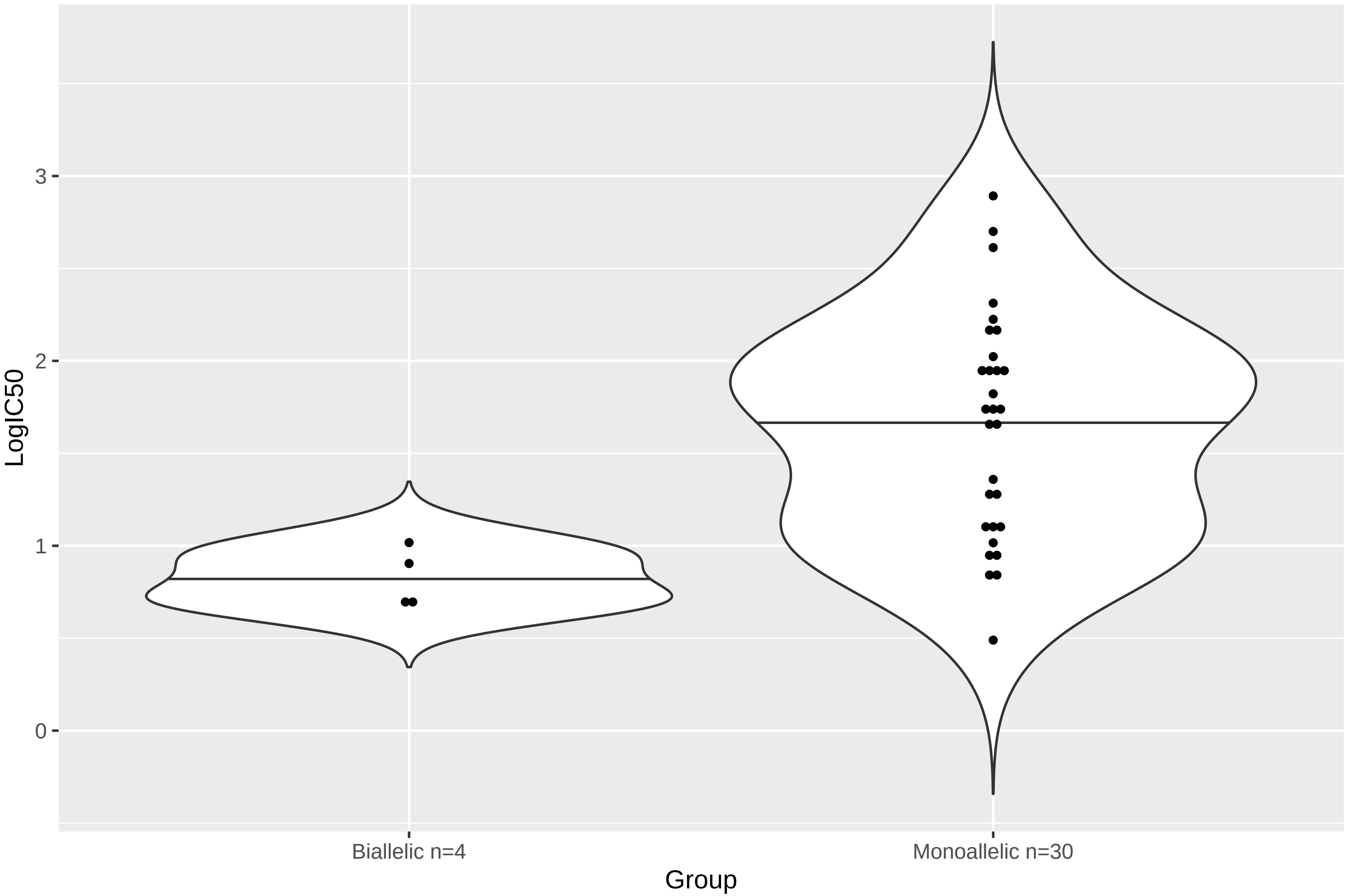

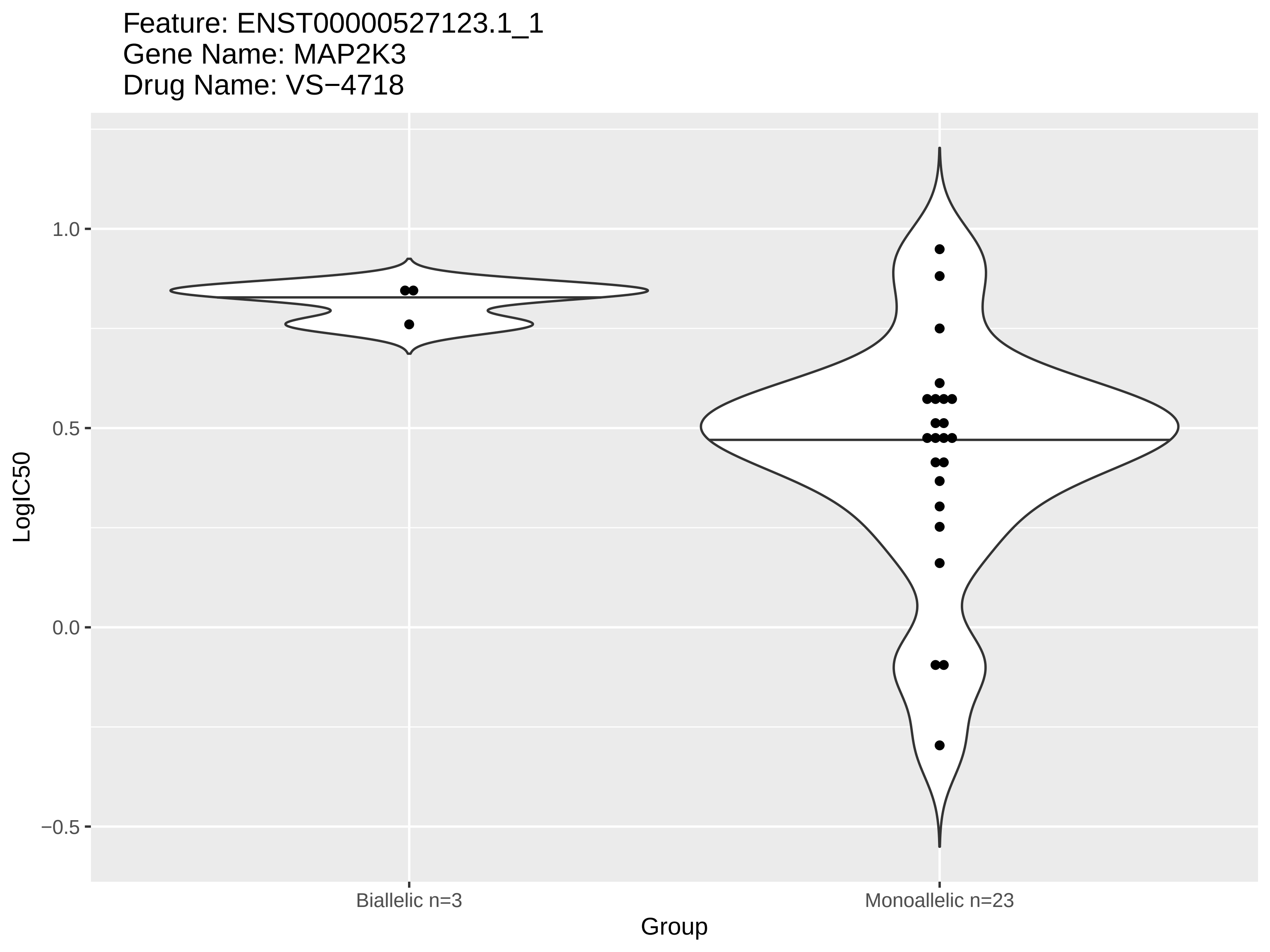
Feature: ENST00000527123.1_1
Gene Name: MAP2K3
Drug Name: VS−4718
1.0
0.5
0.0
−0.5
LogIC50
Biallelic n=3
Monoallelic n=23
Group

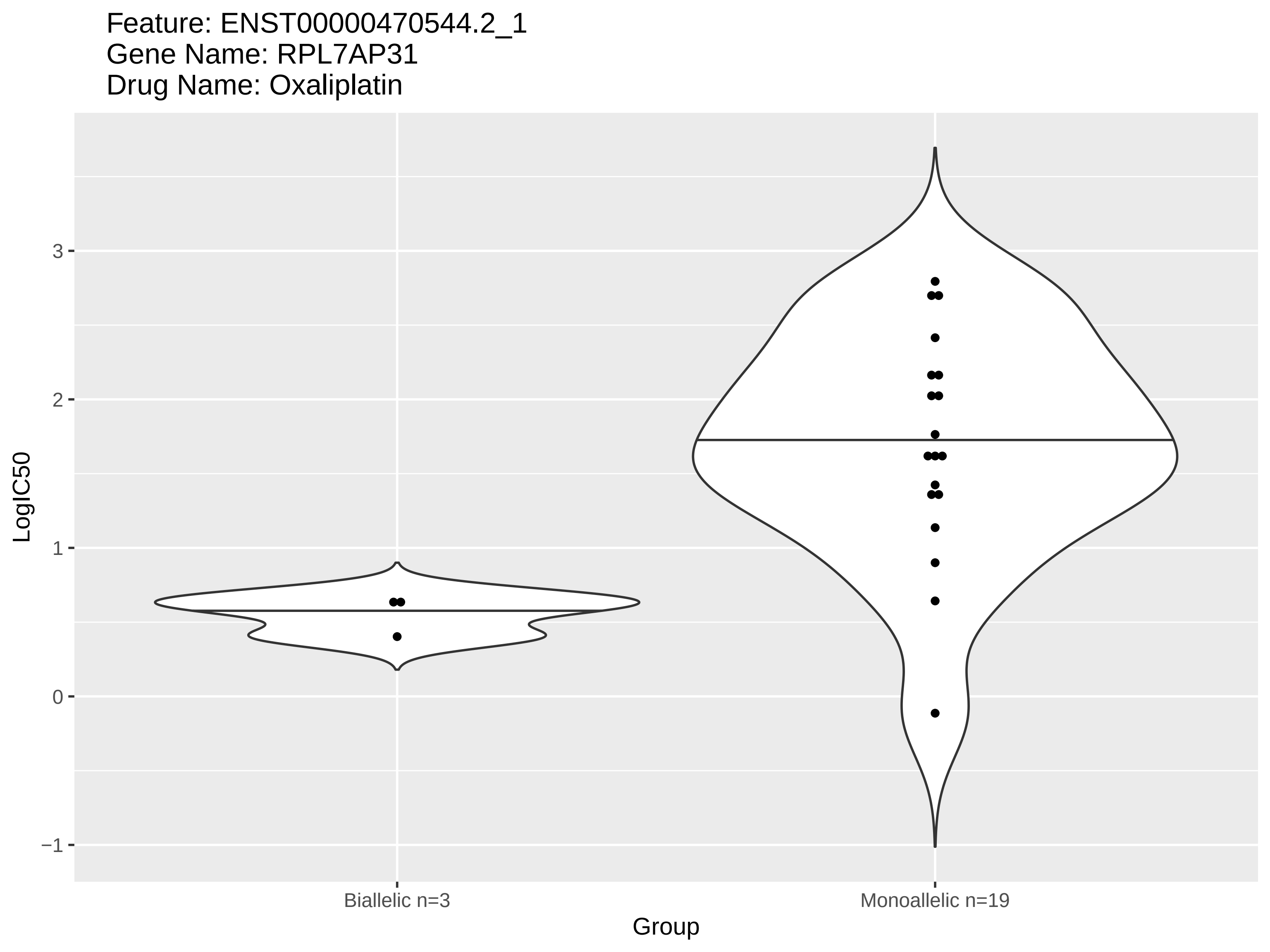
Feature: ENST00000470544.2_1
Gene Name: RPL7AP31
Drug Name: Oxaliplatin
LogIC50
3
2
1
0
−1
Biallelic n=3
Monoallelic n=19
Group

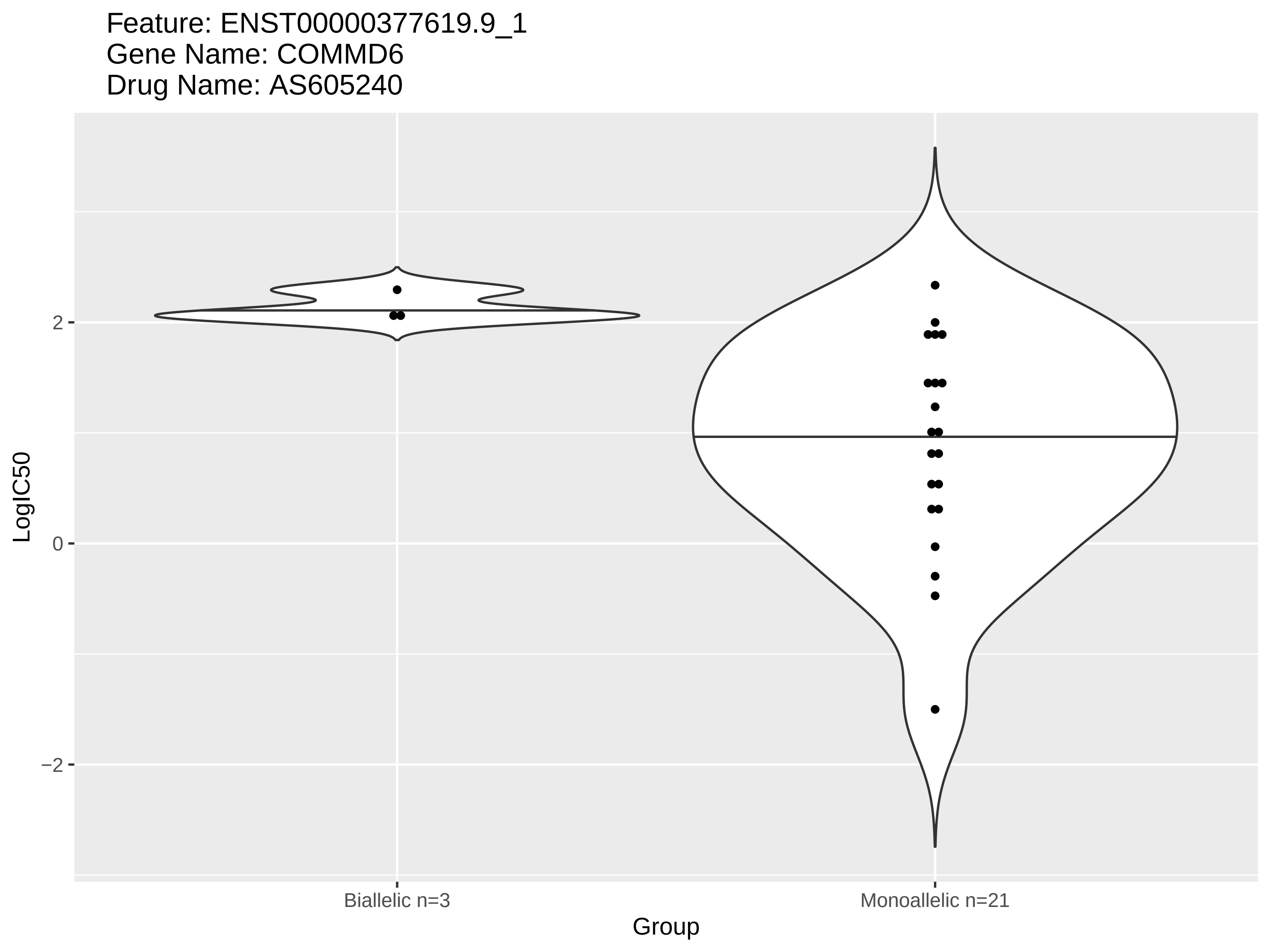
Feature: ENST00000377619.9_1
Gene Name: COMMD6
Drug Name: AS605240
LogIC50
2
0
−2
Biallelic n=3
Monoallelic n=21
Group

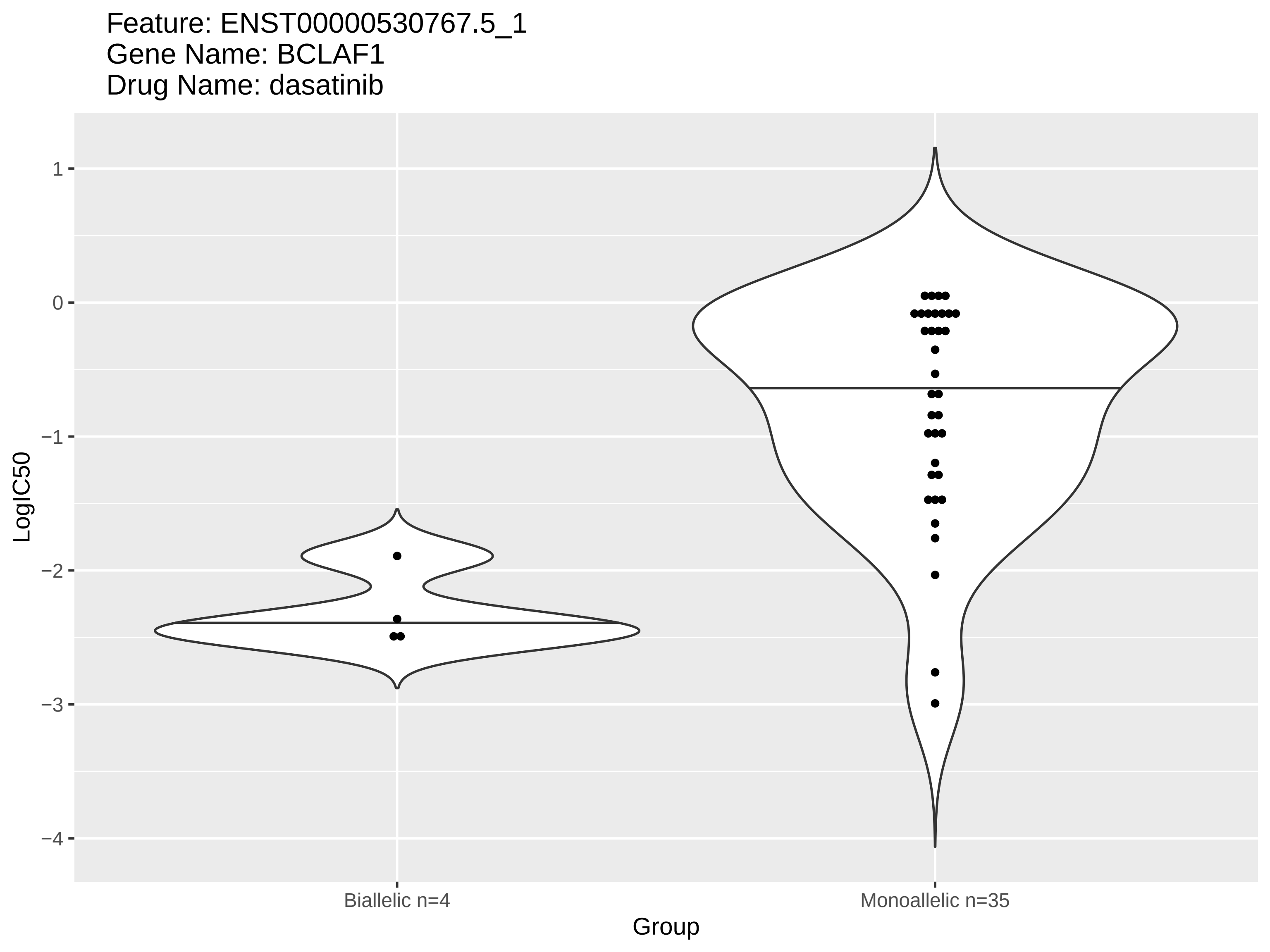
Feature: ENST00000530767.5_1
Gene Name: BCLAF1
Drug Name: dasatinib
LogIC50
1
0
−1
−2
−3
−4
Biallelic n=4
Monoallelic n=35
Group

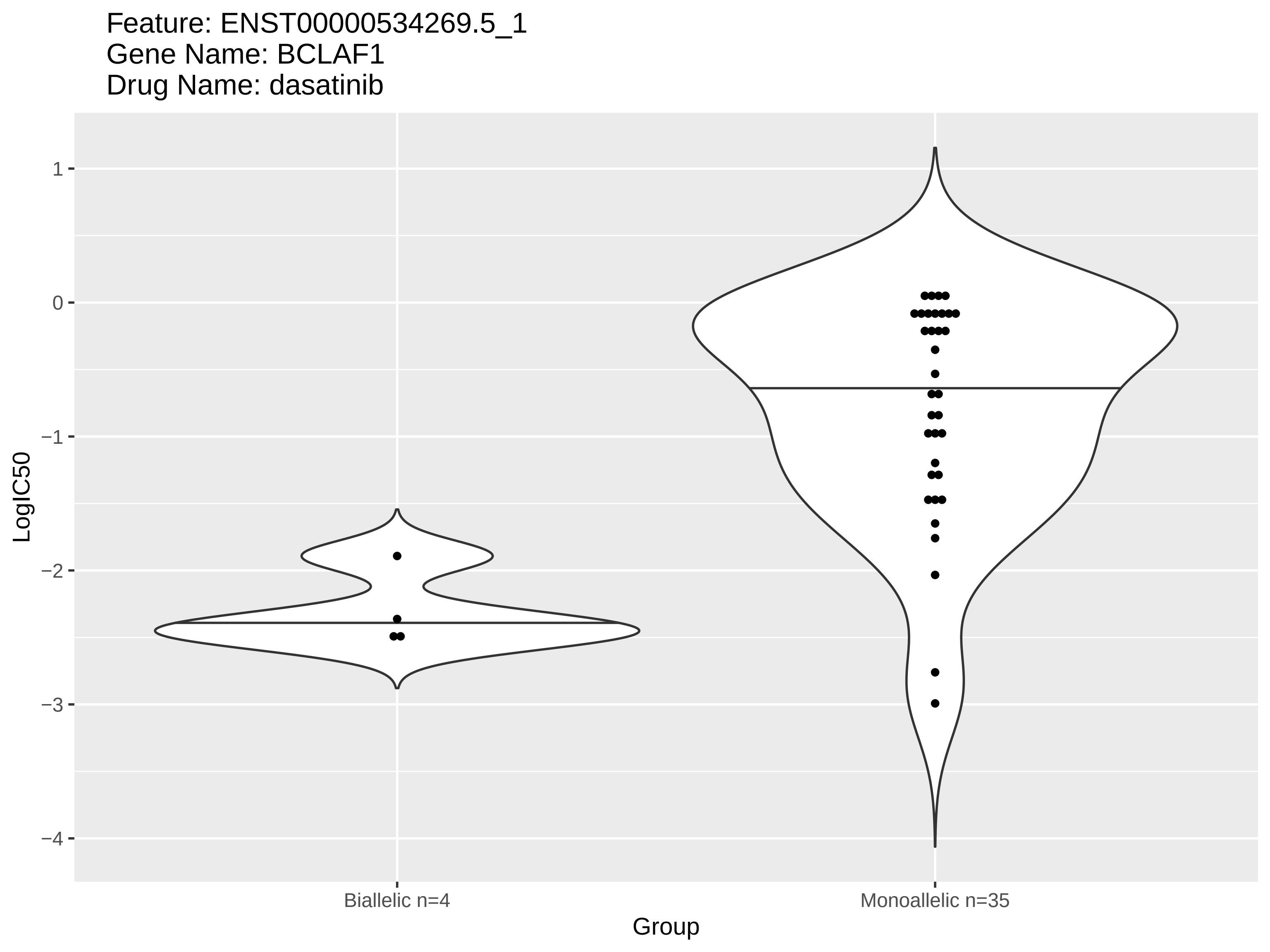
Feature: ENST00000534269.5_1
Gene Name: BCLAF1
Drug Name: dasatinib
1
0
−1
−2
−3
−4
LogIC50
Biallelic n=4
Monoallelic n=35
Group

Feature: ENST00000596415.1_1
Gene Name: NDUFV2P1
Drug Name: FY026

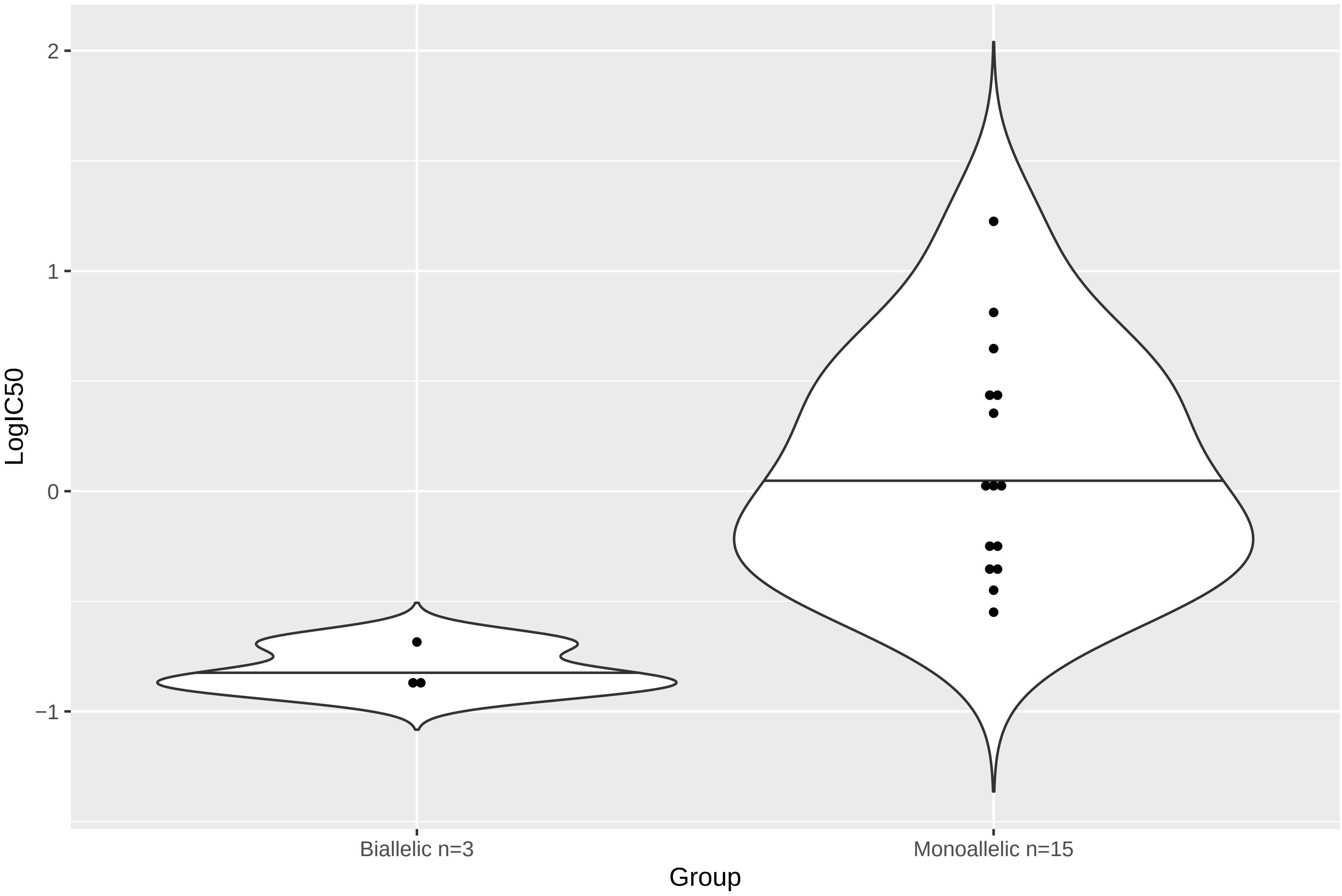

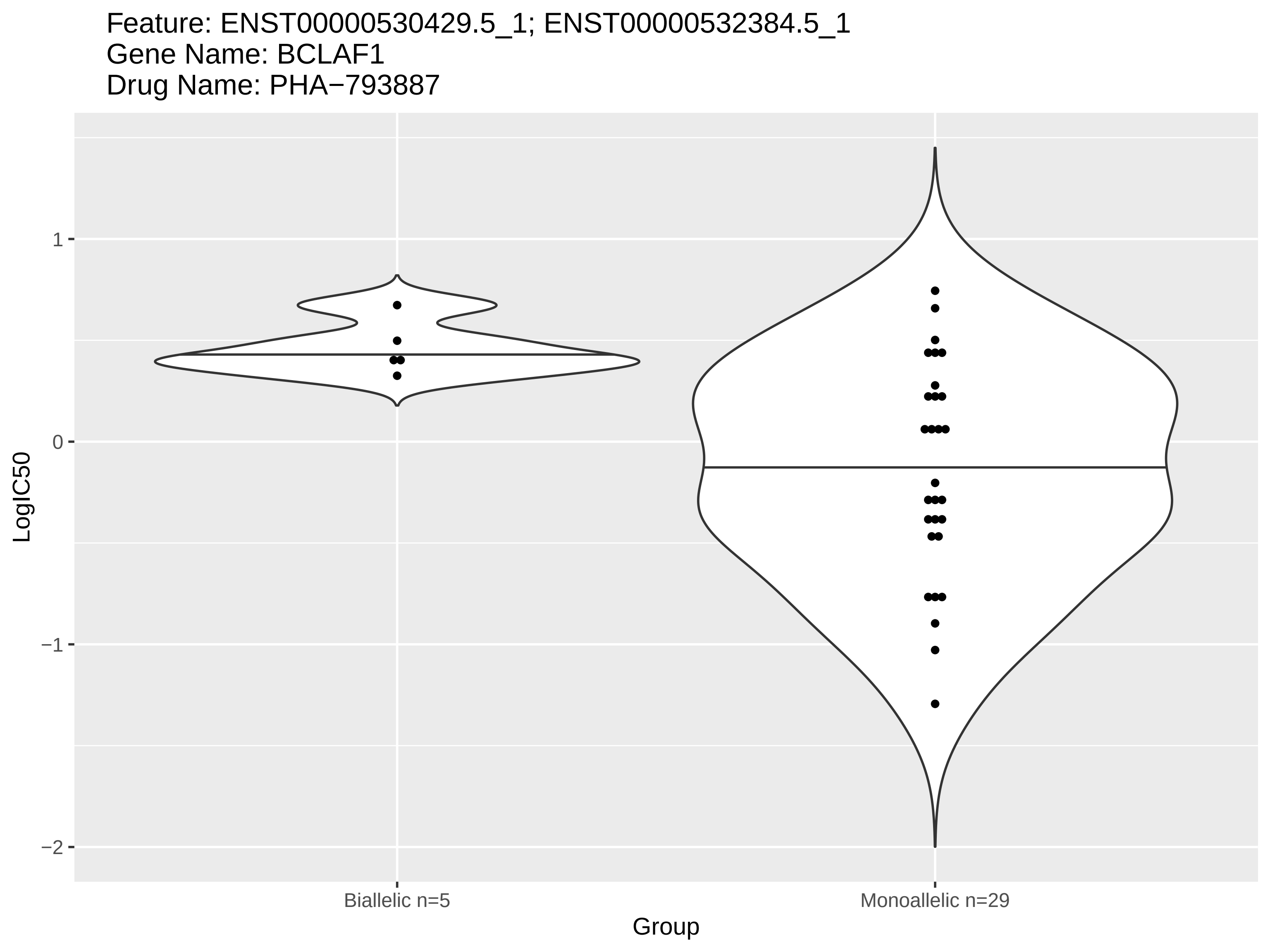

Feature: ENST00000530429.5_1; ENST00000532384.5_1
Gene Name: BCLAF1
Drug Name: PHA−793887
LogIC50
1
0
−1
−2
Biallelic n=5
Monoallelic n=29
Group

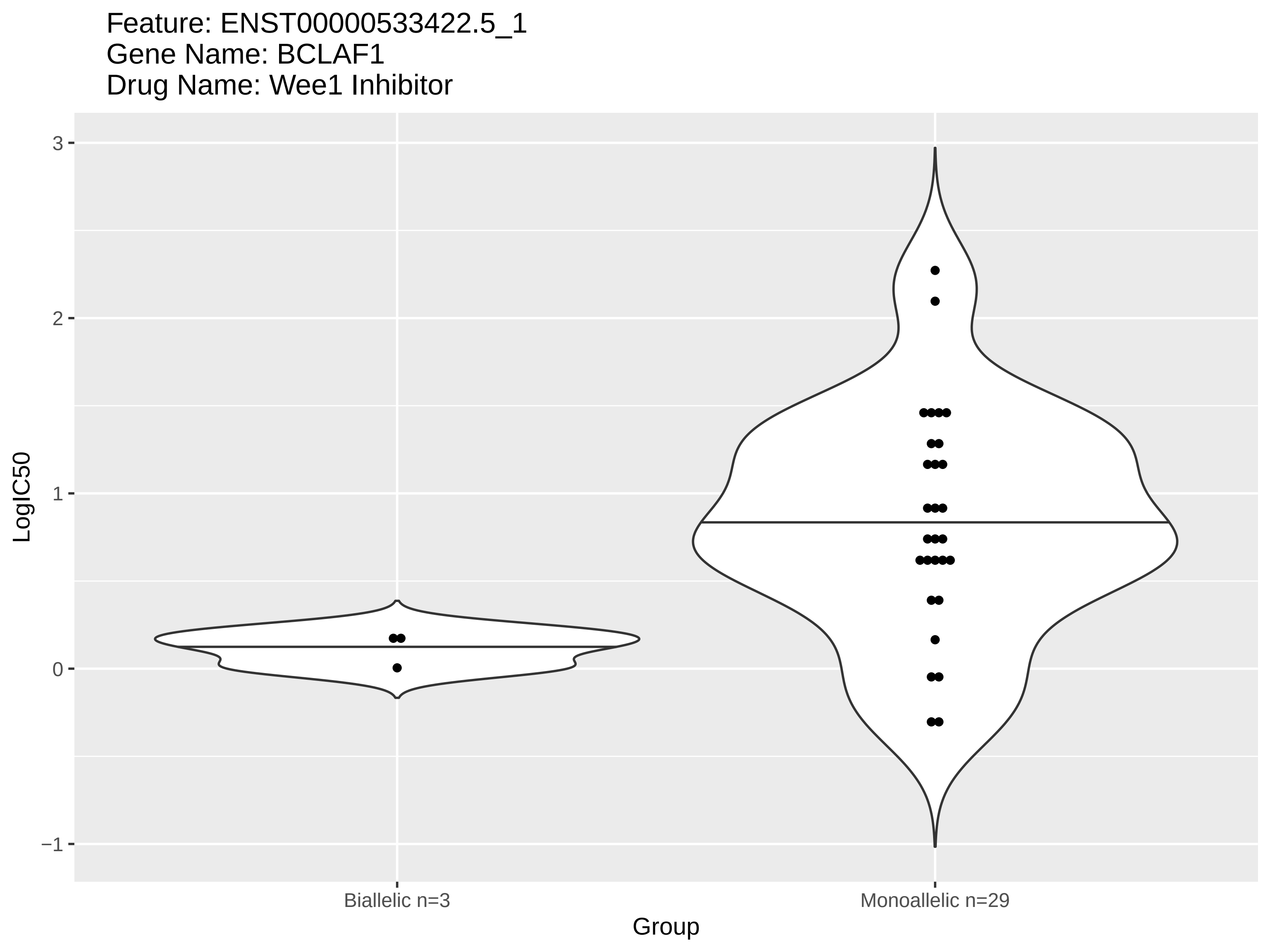
Feature: ENST00000533422.5_1
Gene Name: BCLAF1
Drug Name: Wee1 Inhibitor
LogIC50
3
2
1
0
−1
Biallelic n=3
Monoallelic n=29
Group

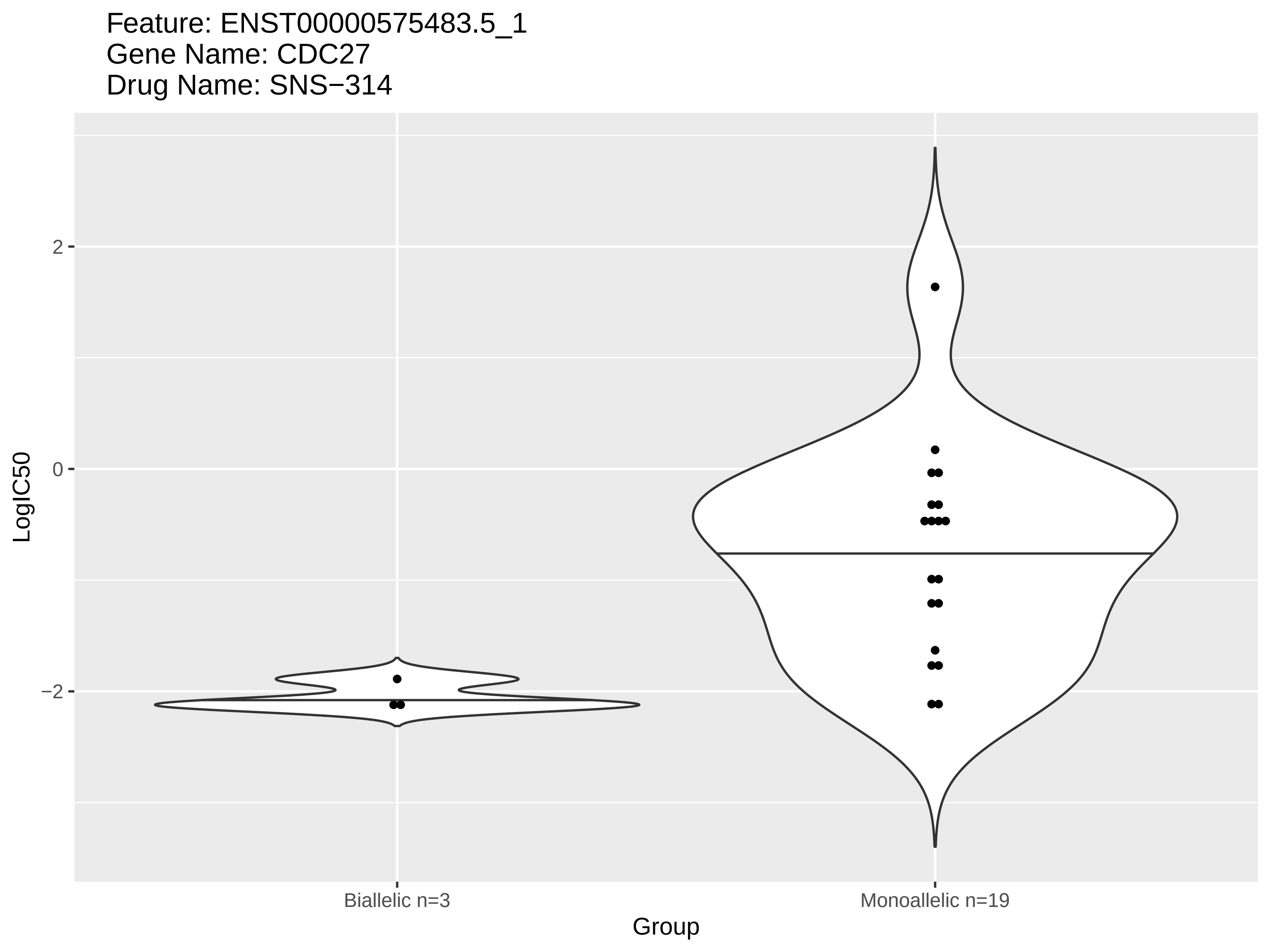

Feature: ENST00000575483.5_1
Gene Name: CDC27
Drug Name: SNS−314
LogIC50
2
0
−2
Biallelic n=3
Monoallelic n=19
Group

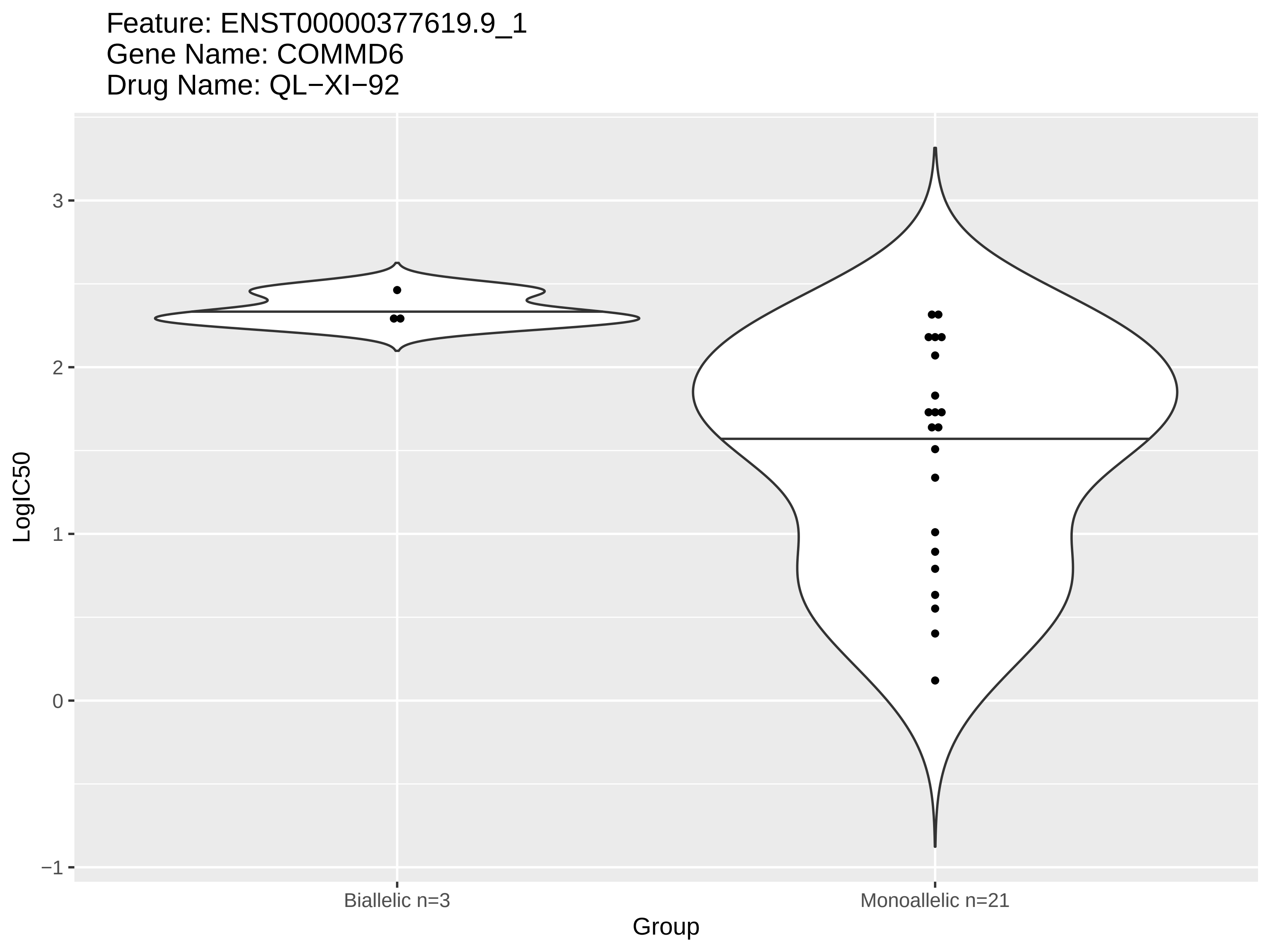

Feature: ENST00000377619.9_1
Gene Name: COMMD6
Drug Name: QL−XI−92
LogIC50
3
2
1
0
−1
Biallelic n=3
Monoallelic n=21
Group

Feature: ENST00000353331.8_1; ENST00000531224.6_1
Gene Name: BCLAF1
Drug Name: PP−1

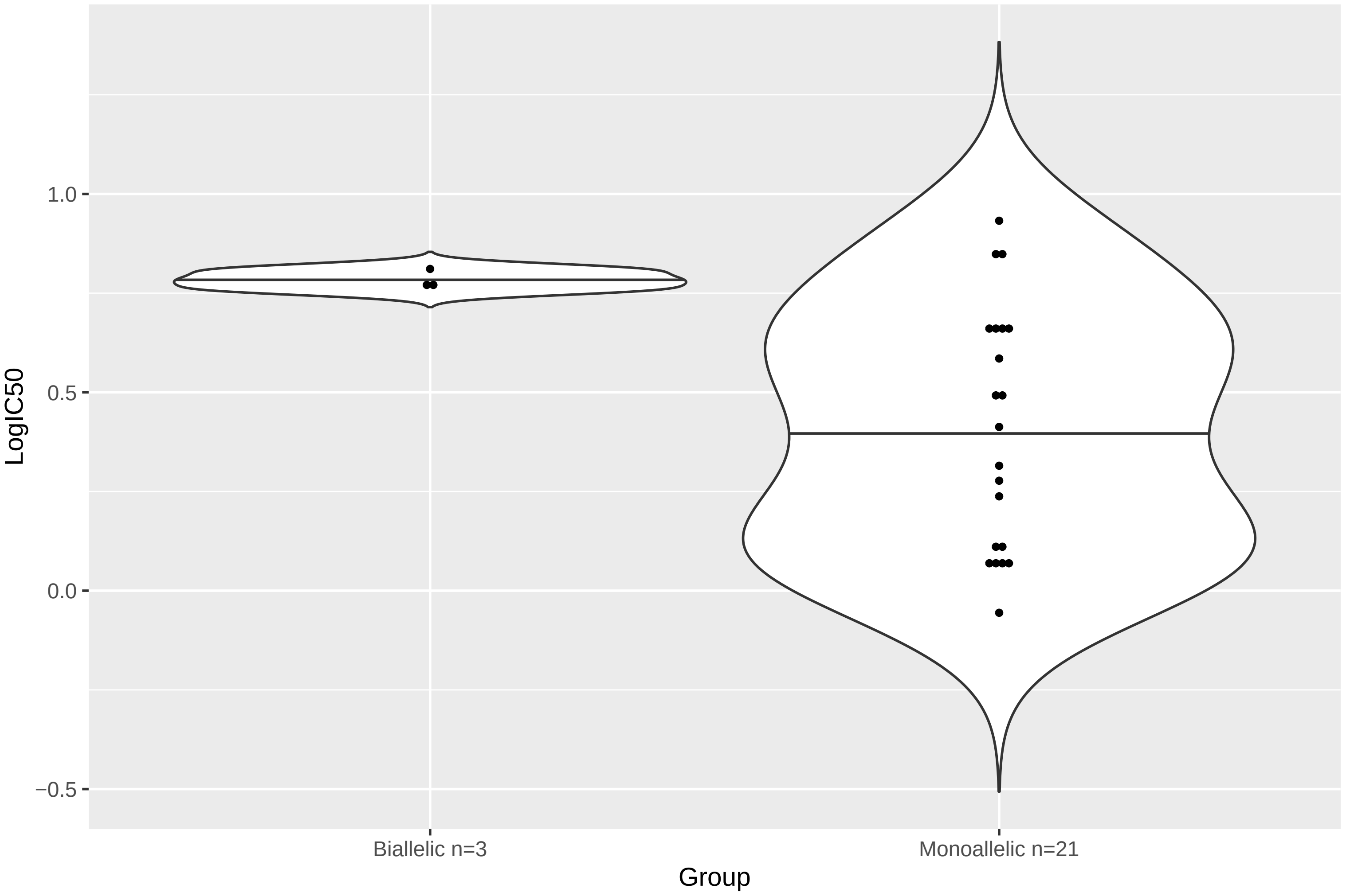

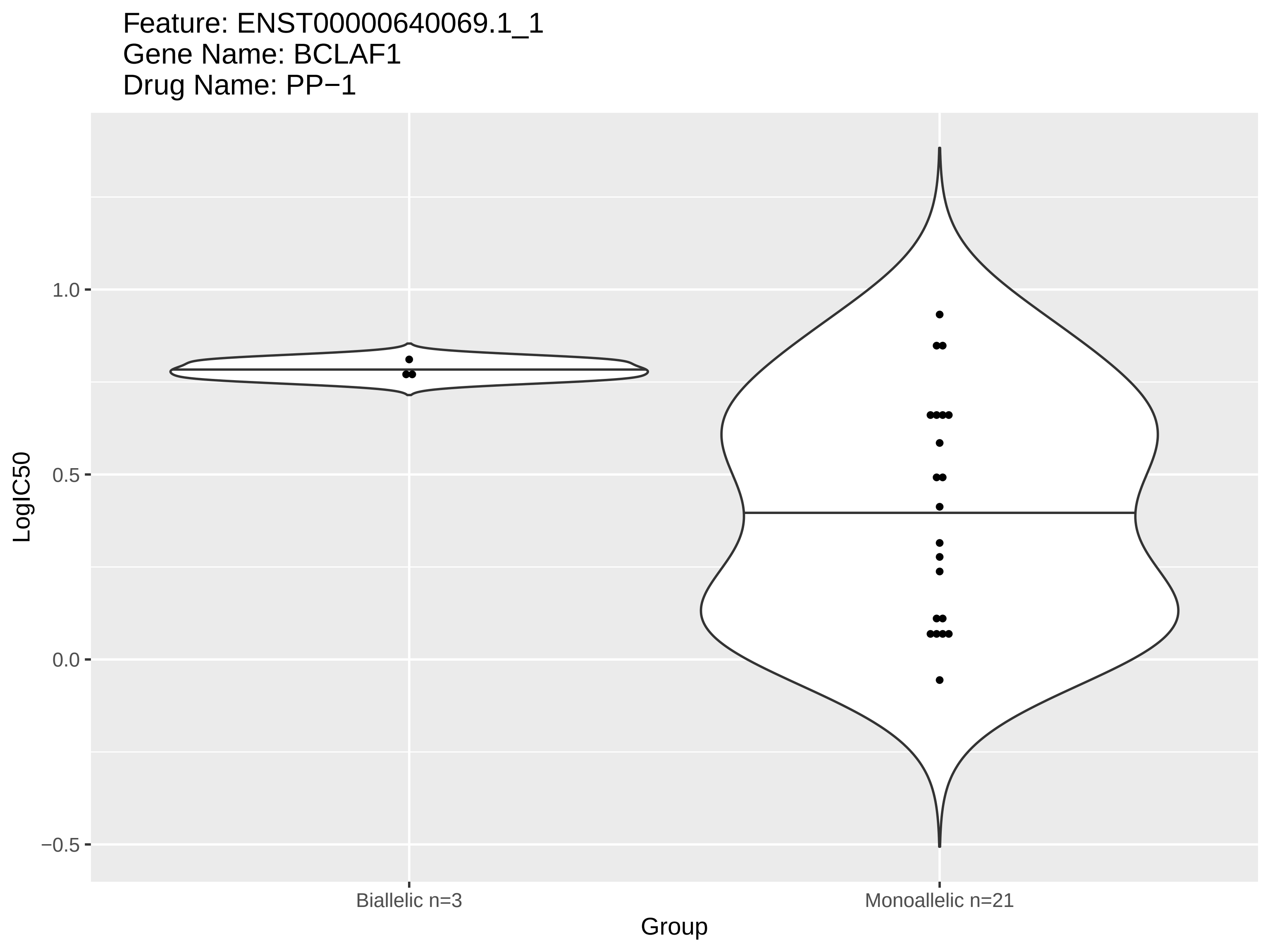
Feature: ENST00000640069.1_1
Gene Name: BCLAF1
Drug Name: PP−1
LogIC50
1.0
0.5
0.0
−0.5
Biallelic n=3
Monoallelic n=21
Group

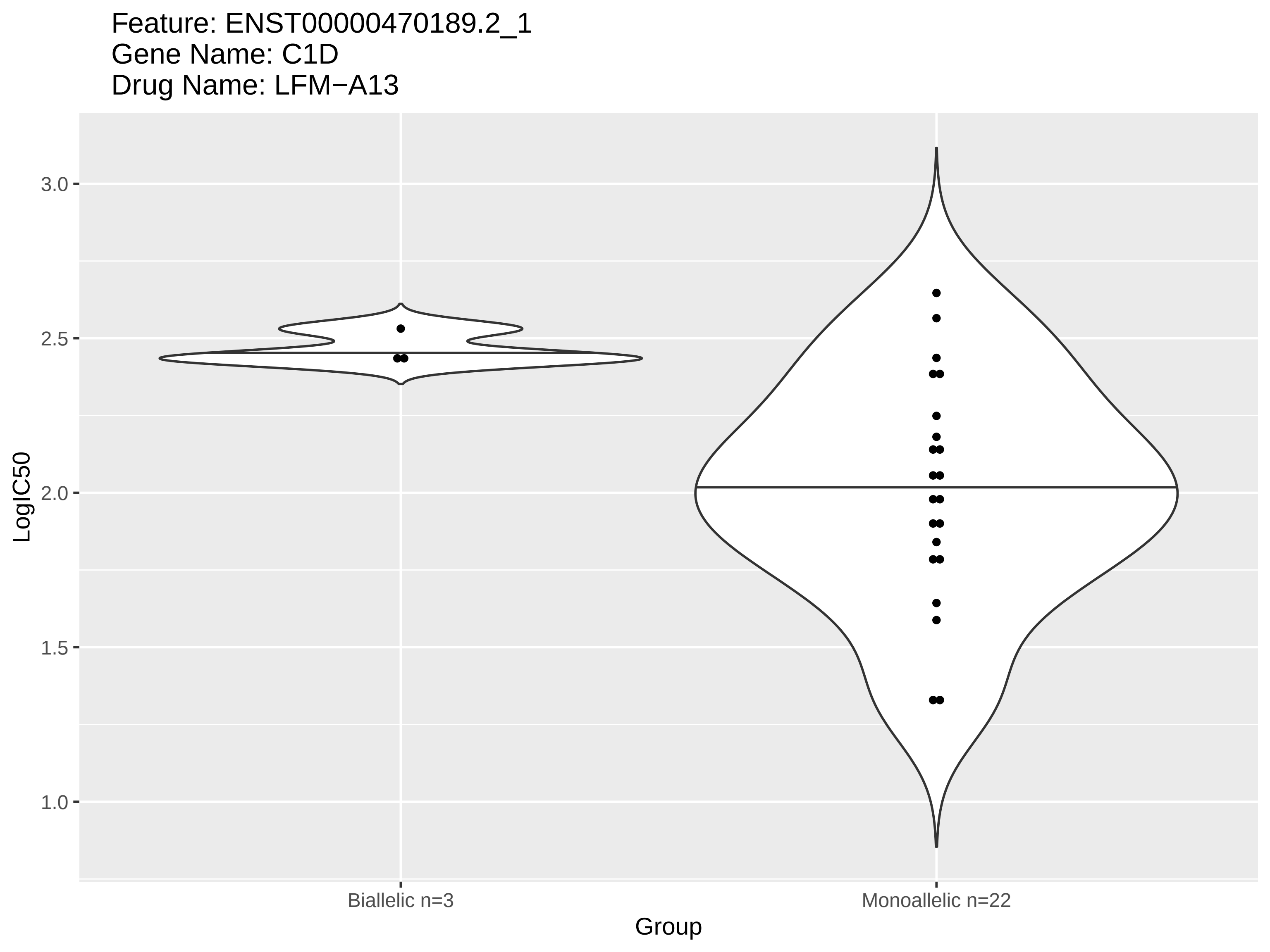
Feature: ENST00000470189.2_1
Gene Name: C1D
Drug Name: LFM−A13
LogIC50
3.0
2.5
2.0
1.5
1.0
Biallelic n=3
Monoallelic n=22
Group

Feature: ENST00000470189.2_1
Gene Name: C1D
Drug Name: Linsitinib

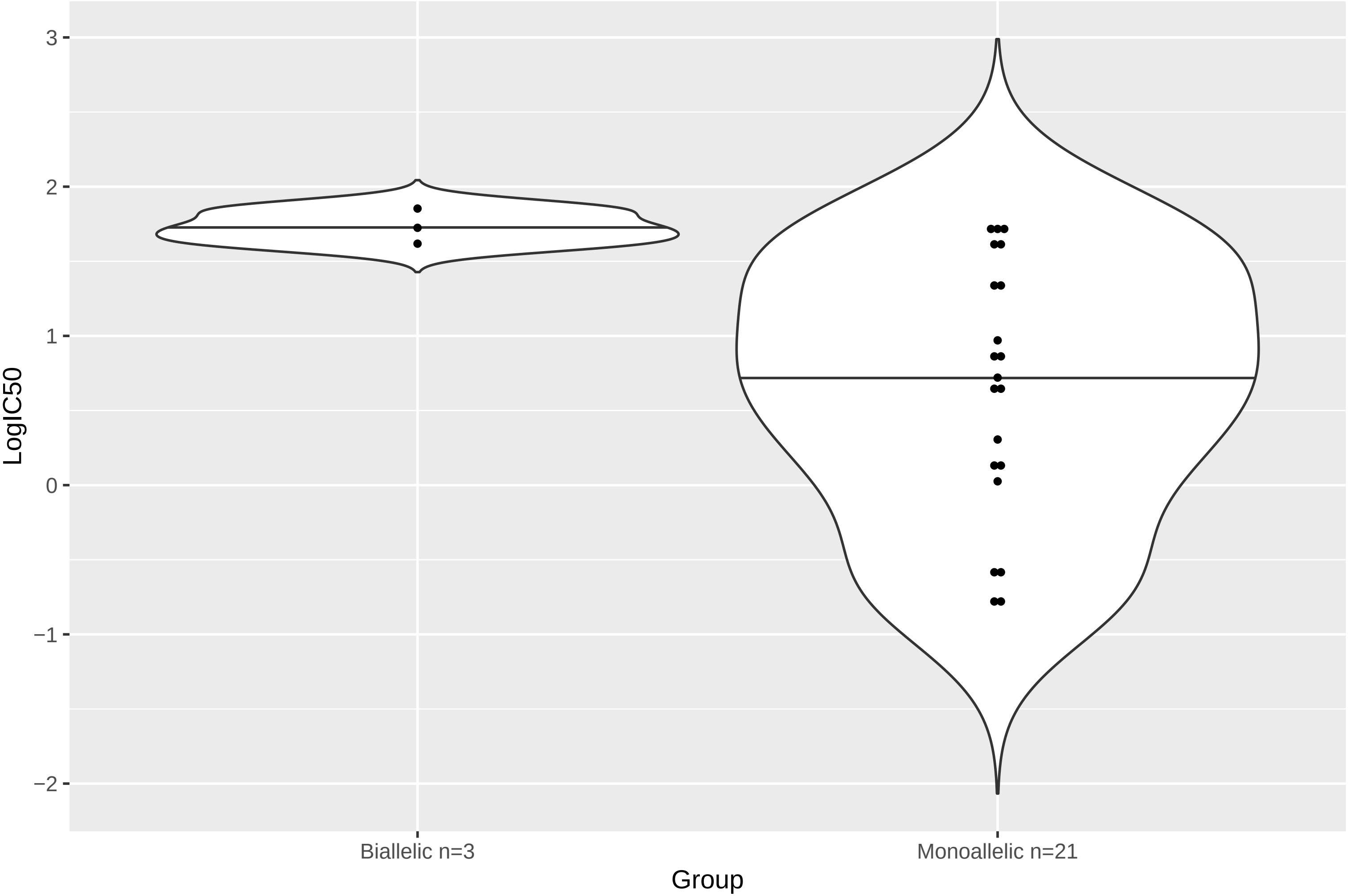

Feature: ENST00000307126.10_1
Gene Name: GTPBP2
Drug Name: FMK

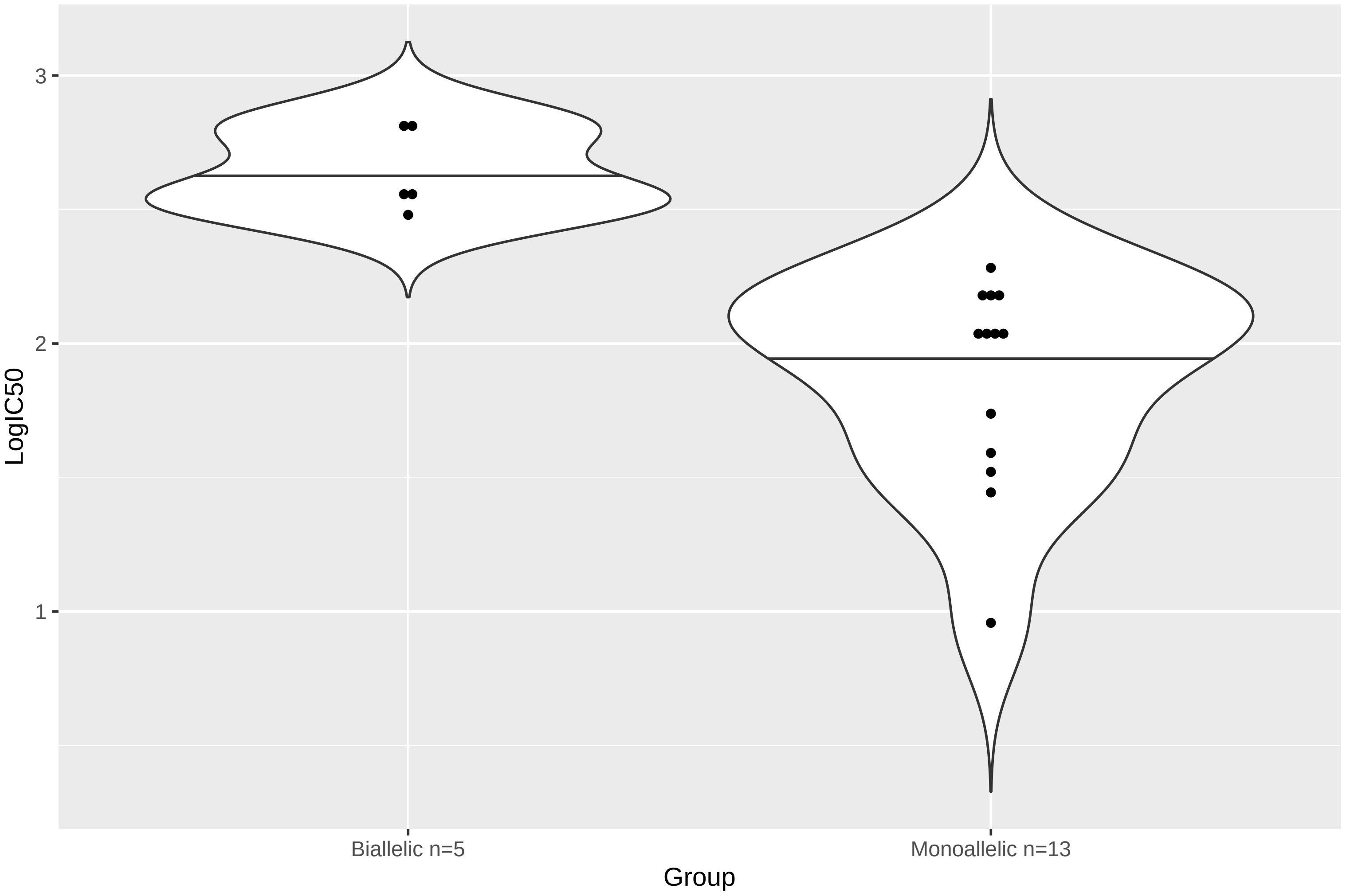

Feature: ENST00000392348.6_1; ENST00000529826.5_1; ENST00000628517.2_1
Gene Name: BCLAF1
Drug Name: fosbretabulin

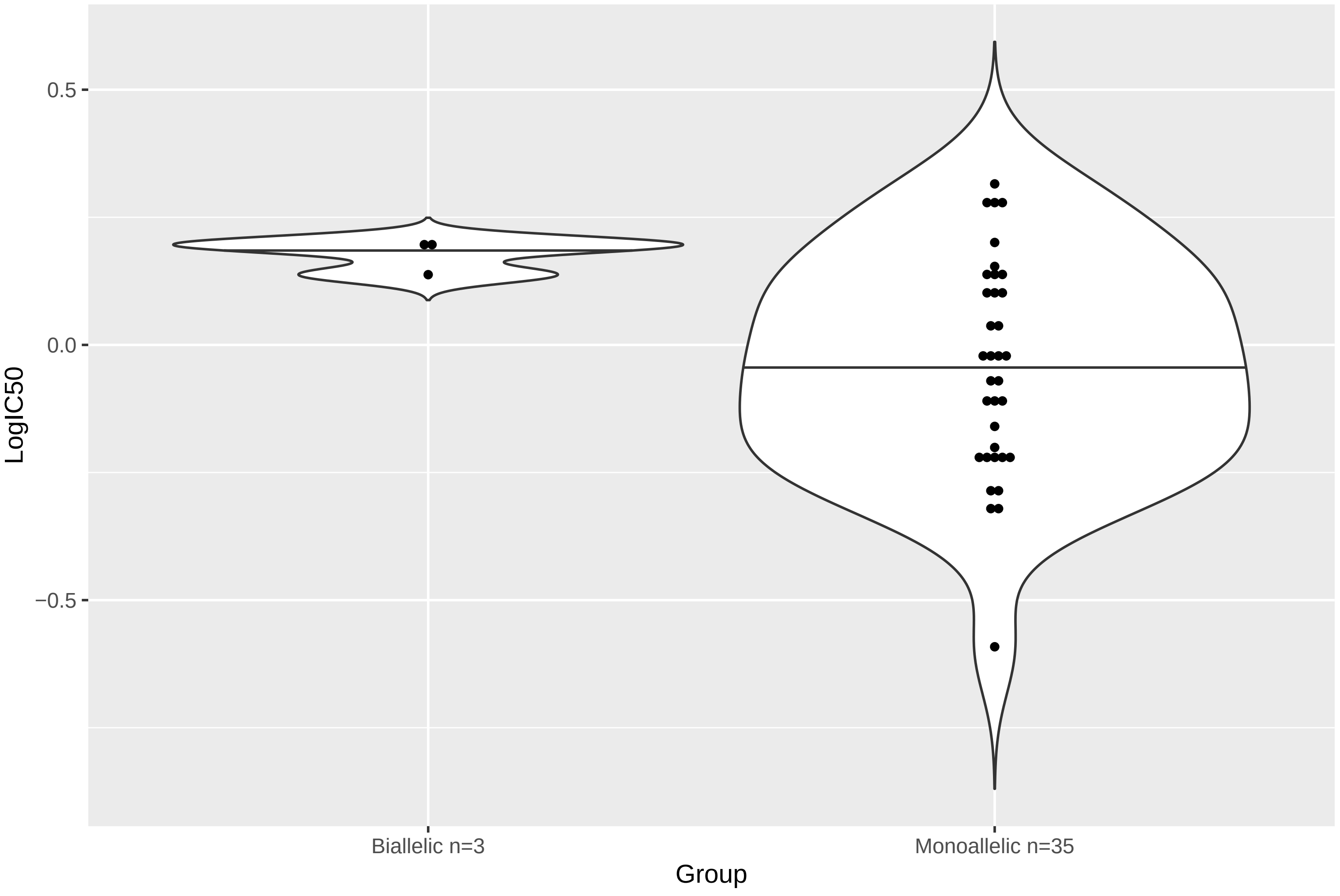

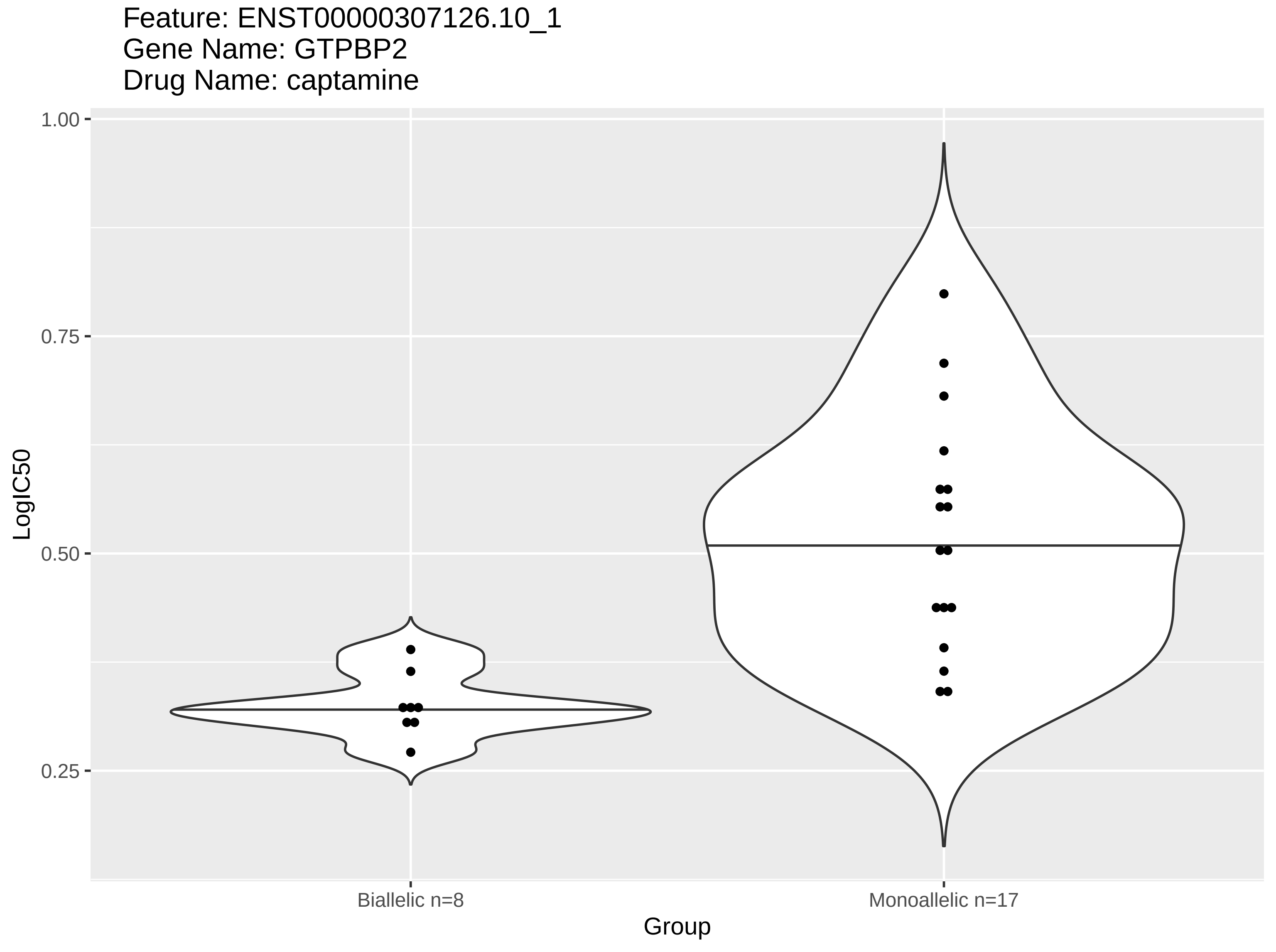

Feature: ENST00000307126.10_1
Gene Name: GTPBP2
Drug Name: captamine
1.00
0.75
0.50
0.25
LogIC50
Biallelic n=8
Monoallelic n=17
Group

Feature: ENST00000573502.1_1
Gene Name: CDC27
Drug Name: sirolimus

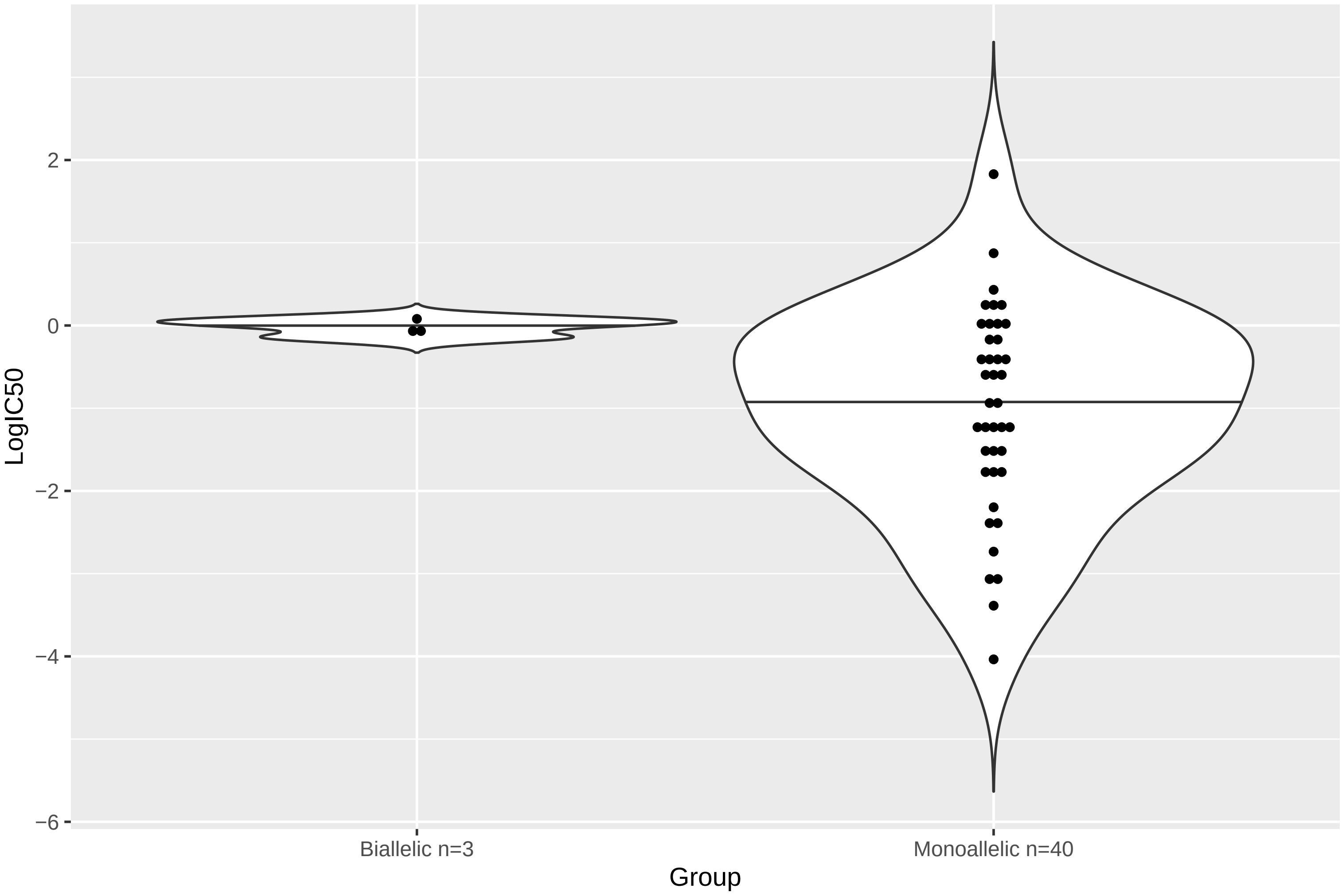

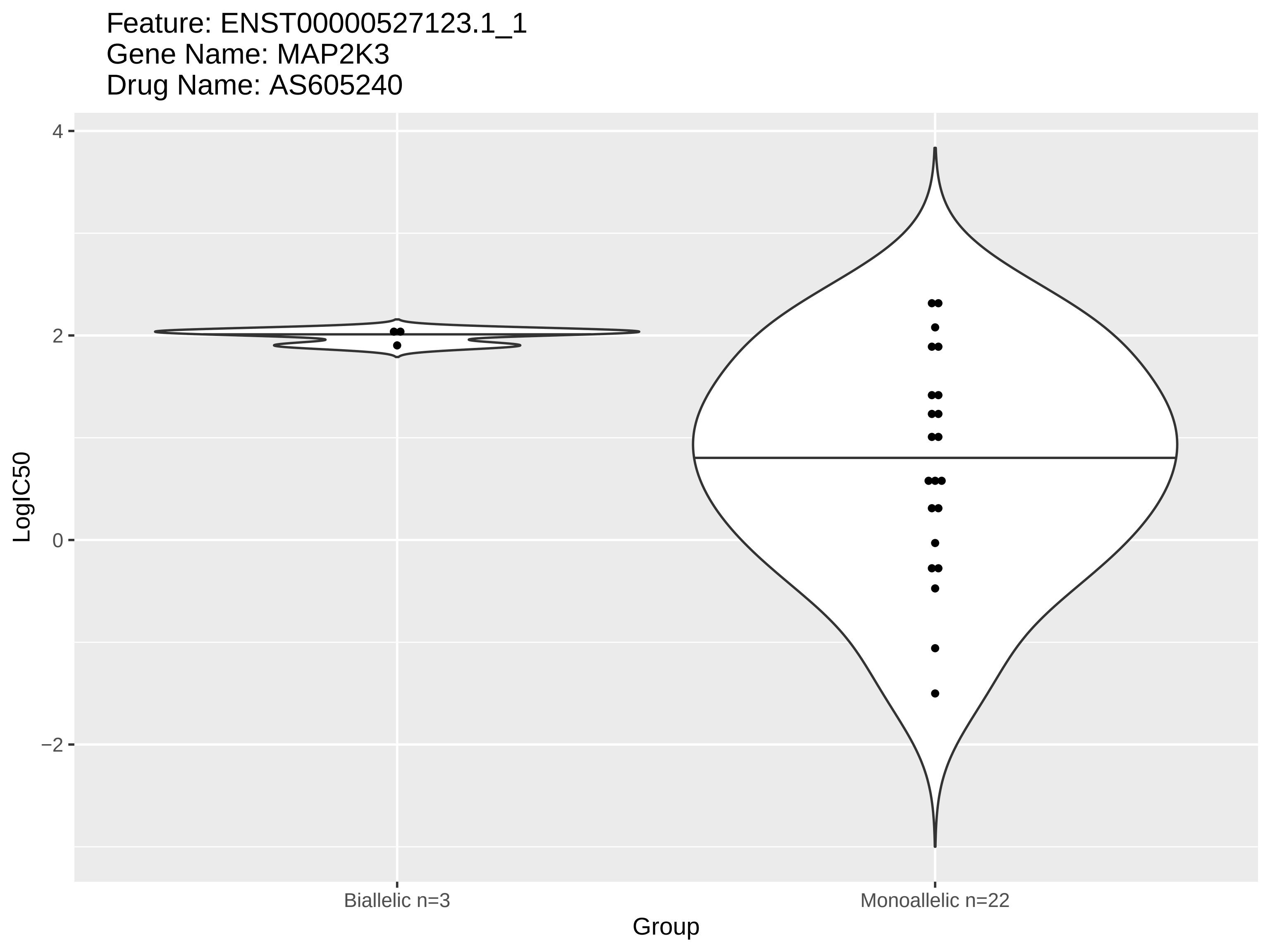
Feature: ENST00000527123.1_1
Gene Name: MAP2K3
Drug Name: AS605240
4
2
0
−2
LogIC50
Biallelic n=3
Monoallelic n=22
Group

Feature: ENST00000490313.1_1
Gene Name: PRIM2
Drug Name: GSK269962A

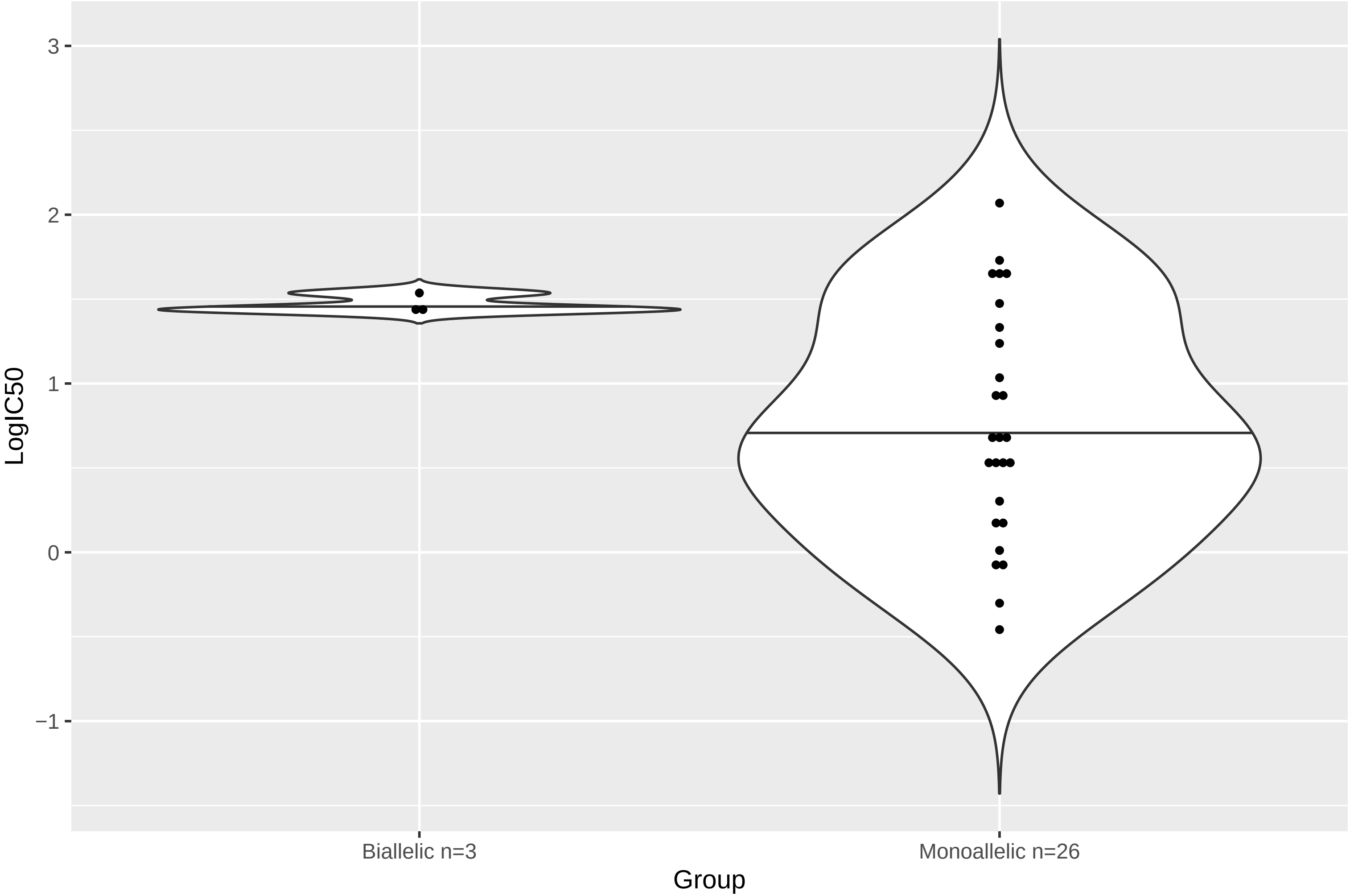

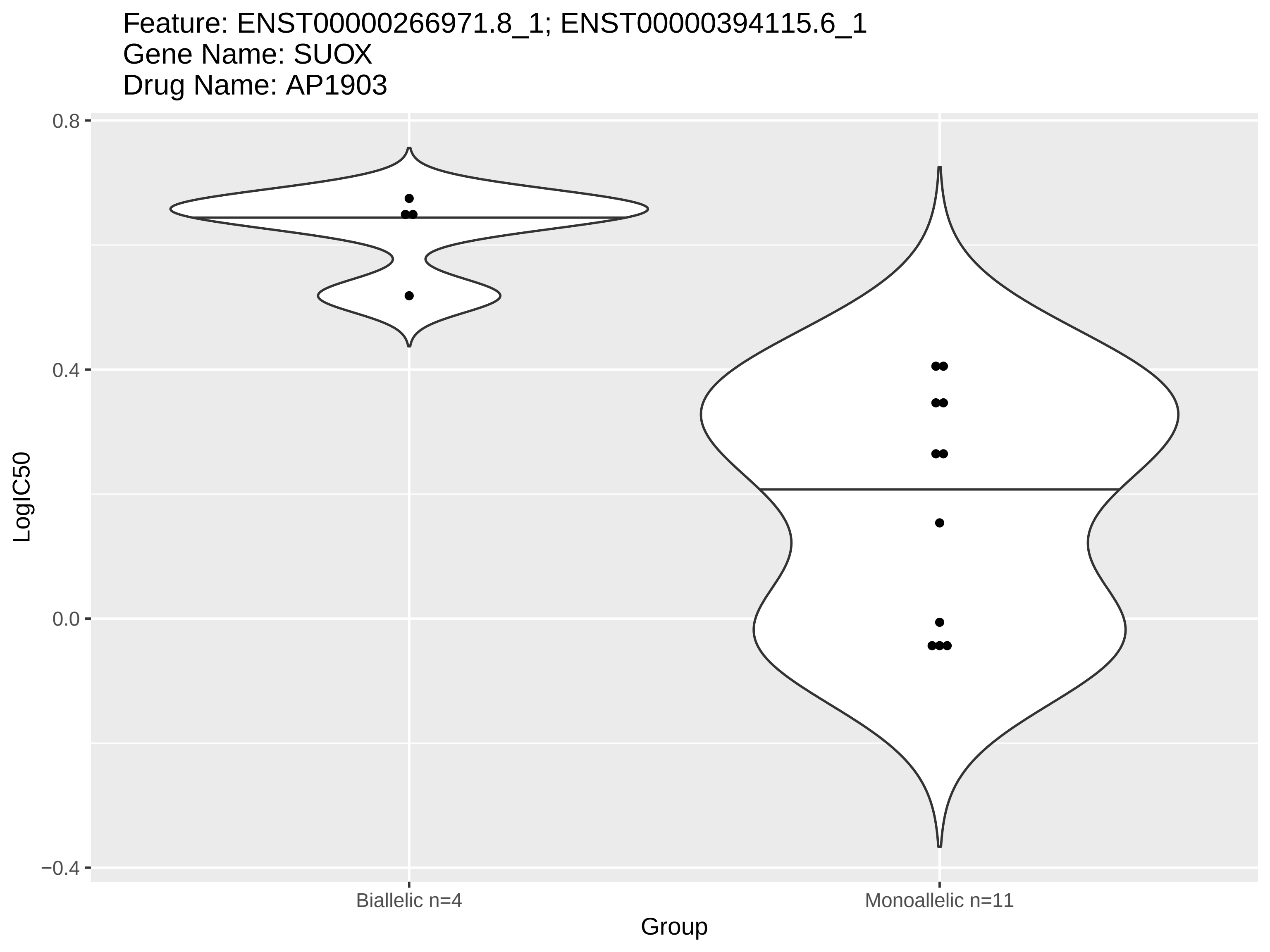

Feature: ENST00000266971.8_1; ENST00000394115.6_1
Gene Name: SUOX
Drug Name: AP1903
0.8
0.4
0.0
−0.4
LogIC50
Biallelic n=4
Monoallelic n=11
Group

Feature: ENST00000356124.8_1; ENST00000548274.5_1; ENST00000550065.1_1
Gene Name: SUOX
Drug Name: AP1903

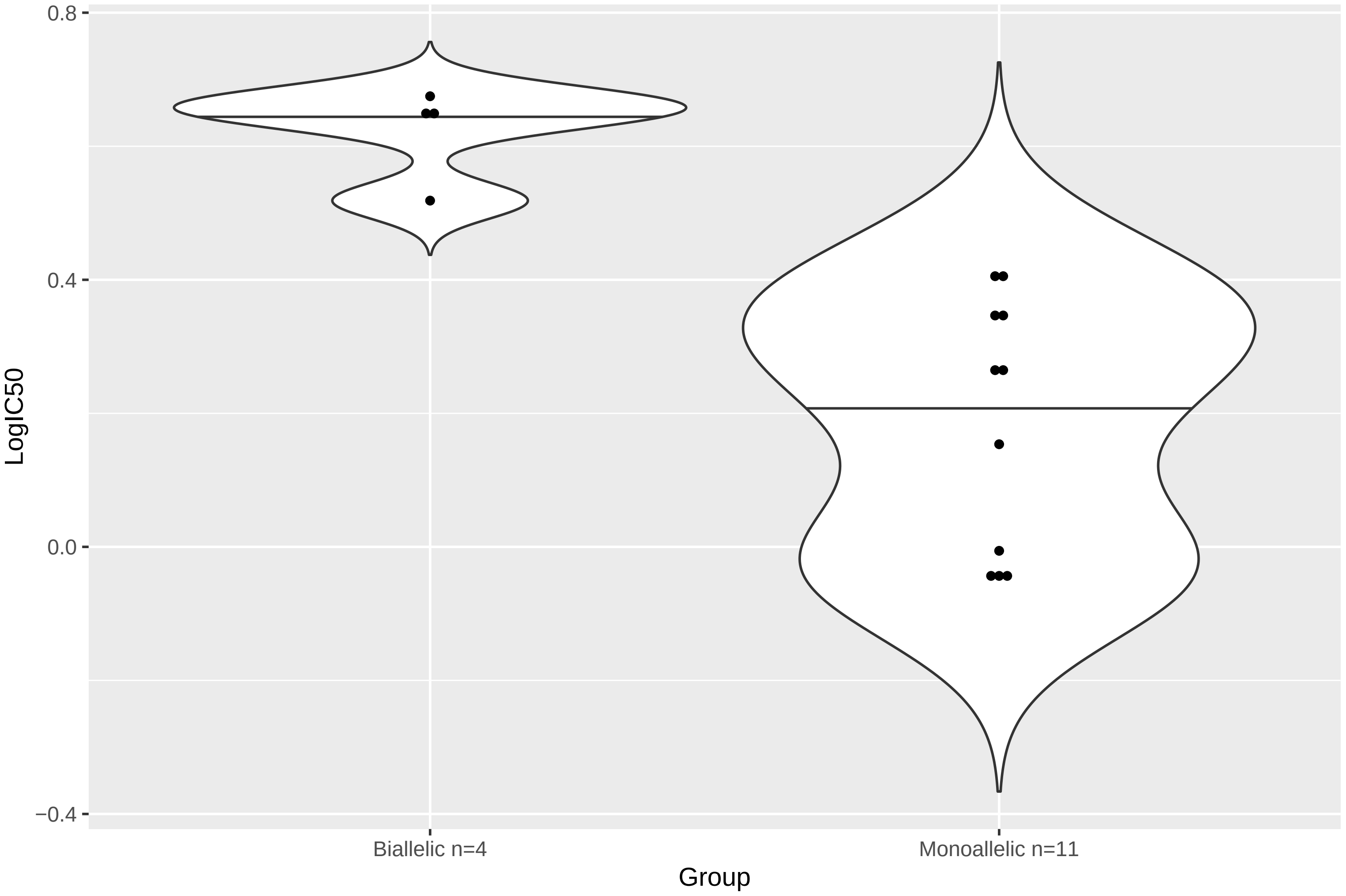

Feature: ENST00000470544.2_1
Gene Name: RPL7AP31
Drug Name: SB−216641

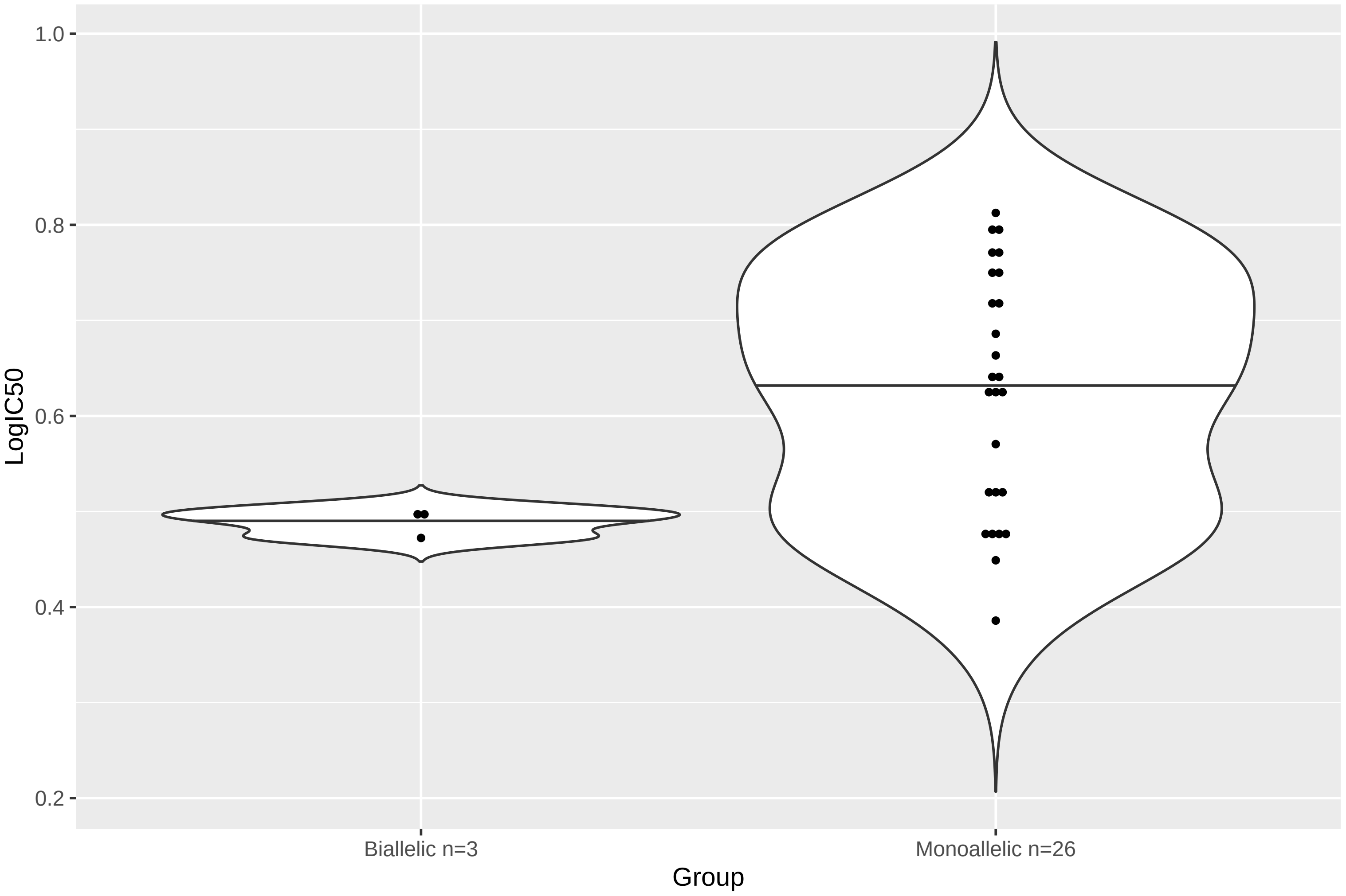

Feature: ENST00000443090.1_1
Gene Name: GCSHP5
Drug Name: N22899−6−C1

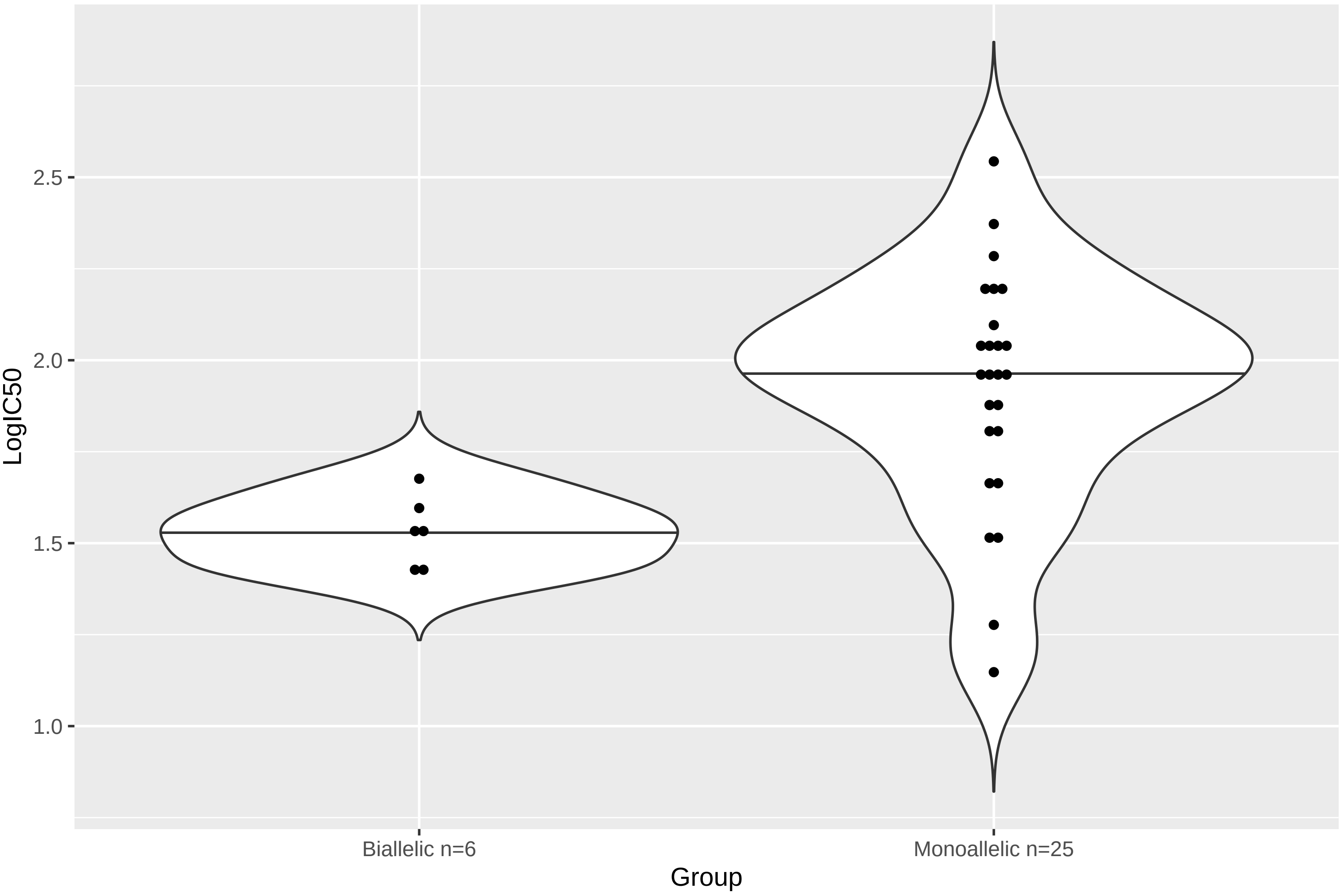

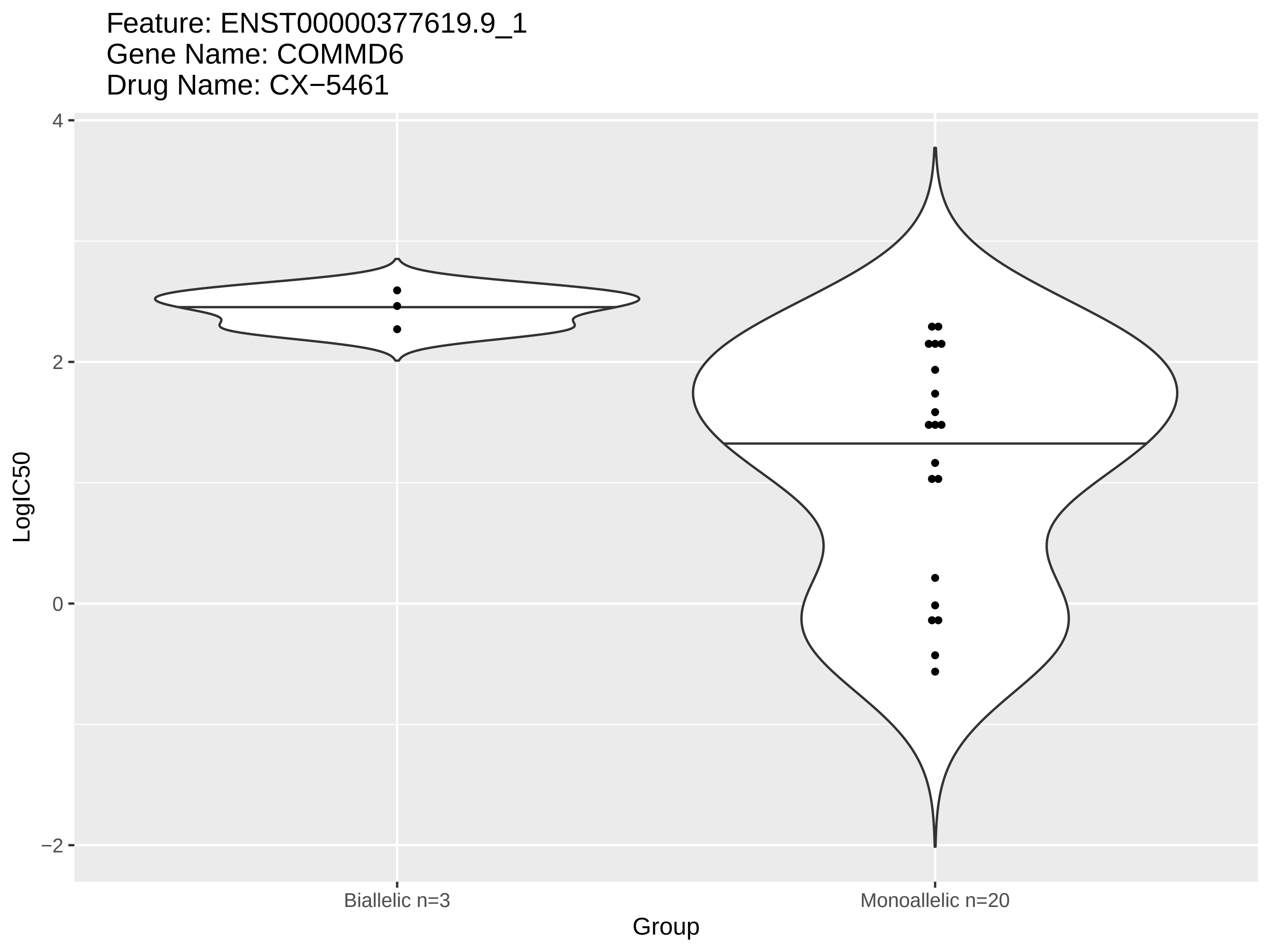
Feature: ENST00000377619.9_1
Gene Name: COMMD6
Drug Name: CX−5461
LogIC50
4
2
0
−2
Biallelic n=3
Monoallelic n=20
Group

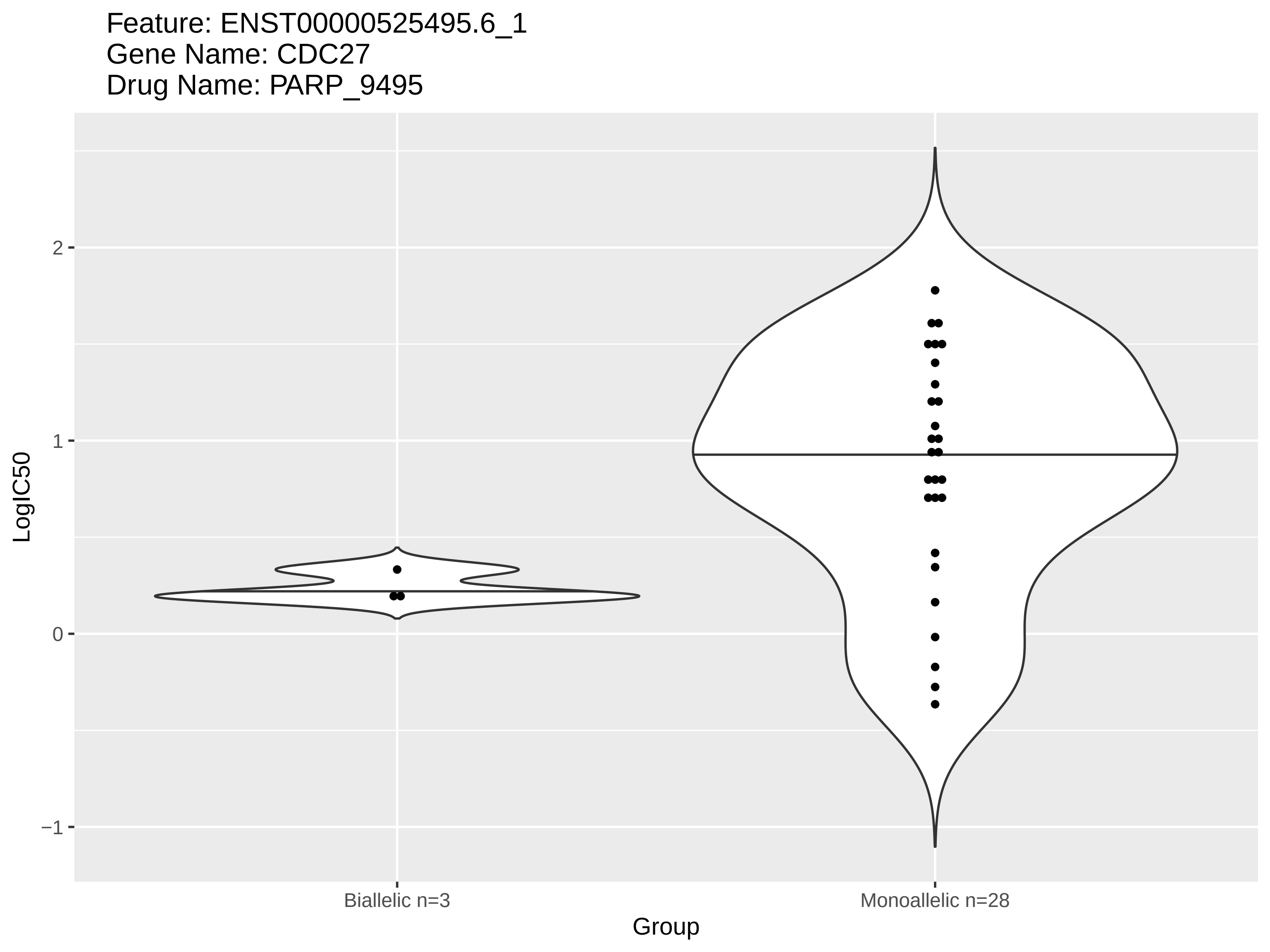
Feature: ENST00000525495.6_1
Gene Name: CDC27
Drug Name: PARP_9495
LogIC50
2
1
0
−1
Biallelic n=3
Monoallelic n=28
Group

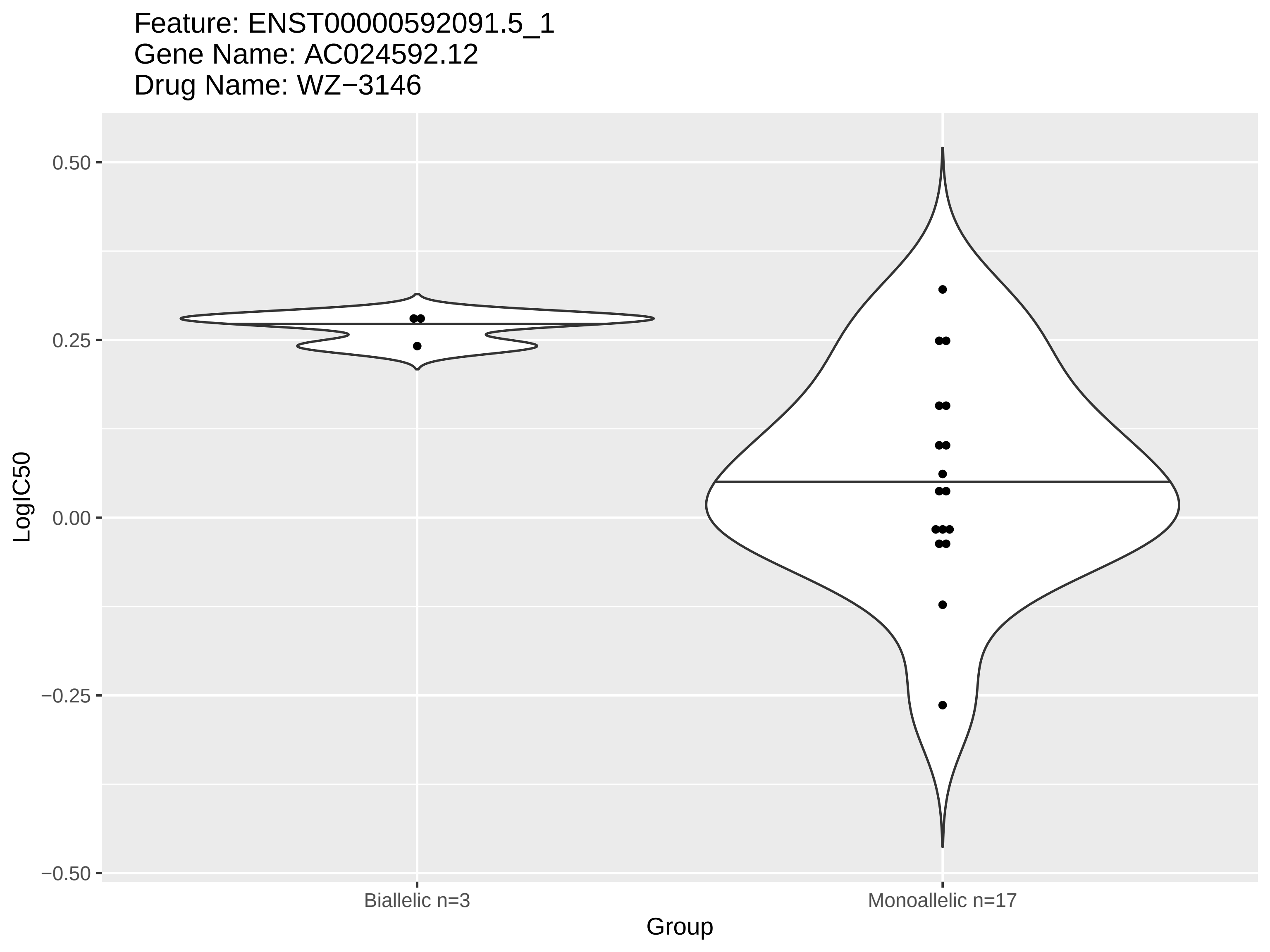

Feature: ENST00000592091.5_1
Gene Name: AC024592.12
Drug Name: WZ−3146
0.50
0.25
0.00
−0.25
−0.50
LogIC50
Biallelic n=3
Monoallelic n=17
Group

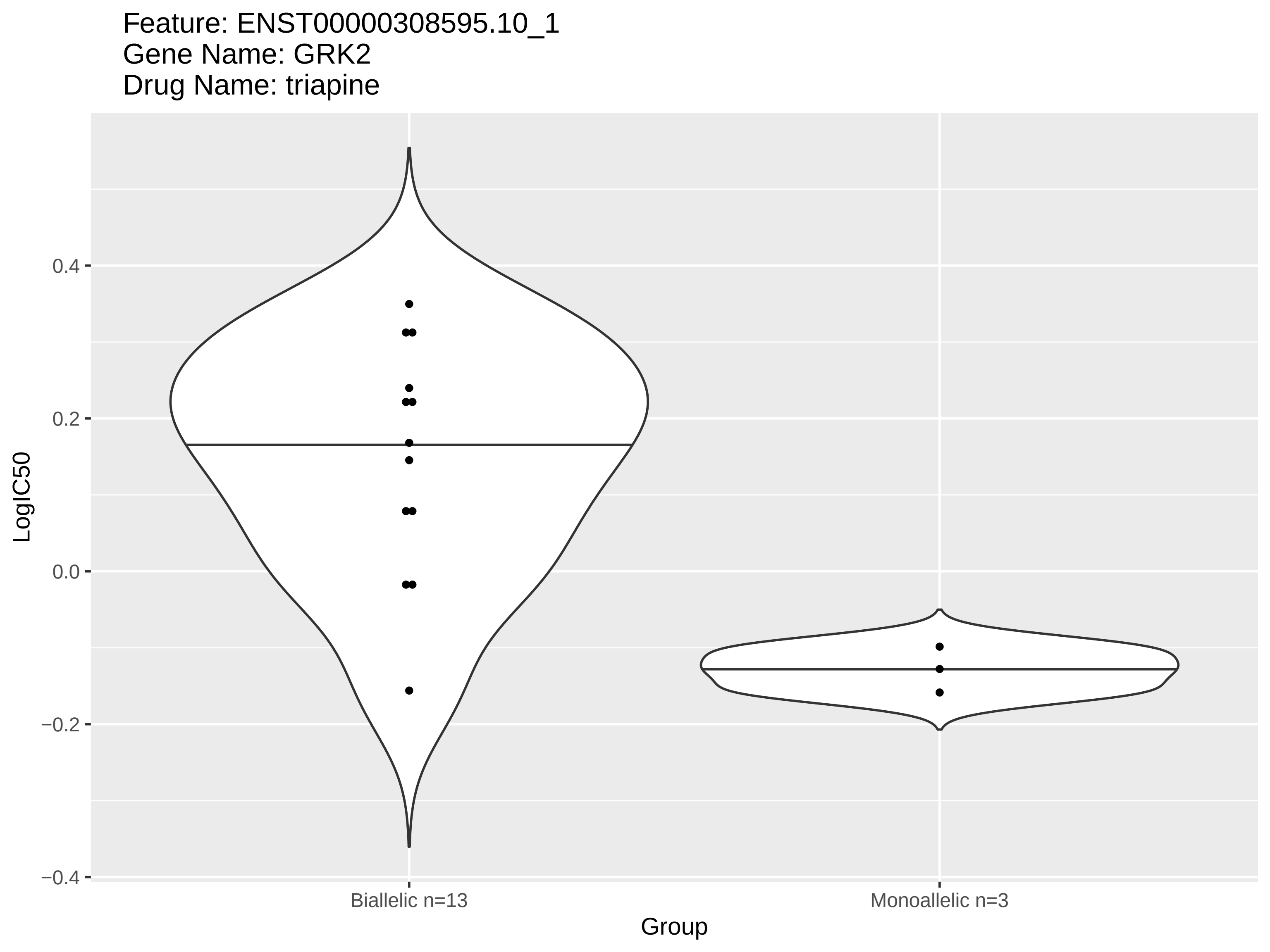
Feature: ENST00000308595.10_1
Gene Name: GRK2
Drug Name: triapine
0.4
0.2
0.0
−0.2
−0.4
LogIC50
Biallelic n=13
Monoallelic n=3
Group

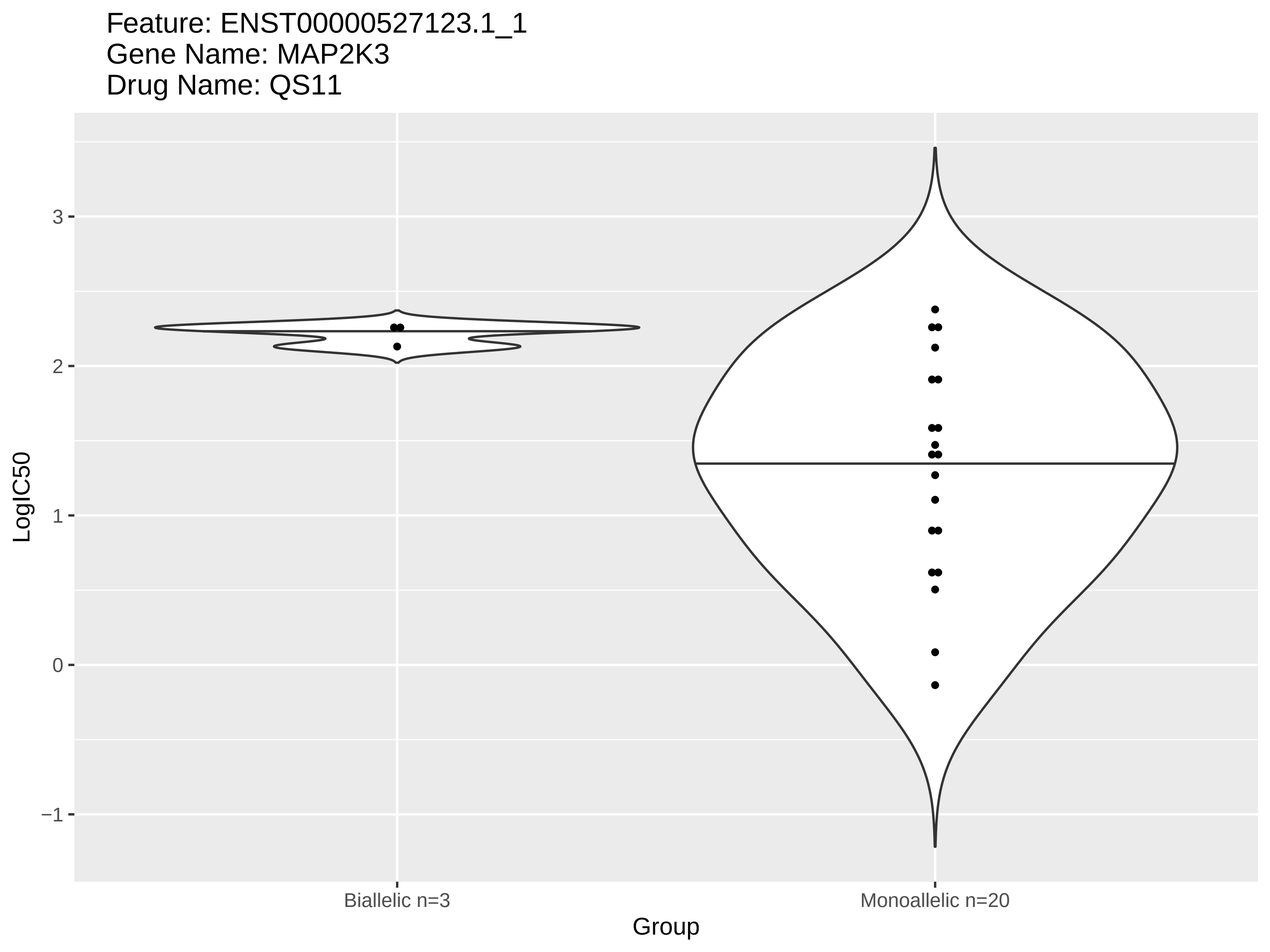

Feature: ENST00000527123.1_1
Gene Name: MAP2K3
Drug Name: QS11
LogIC50
3
2
1
0
−1
Biallelic n=3
Monoalleliс n=20
Group

Feature: ENST00000377619.9_1
Gene Name: COMMD6
Drug Name: TPCA−1

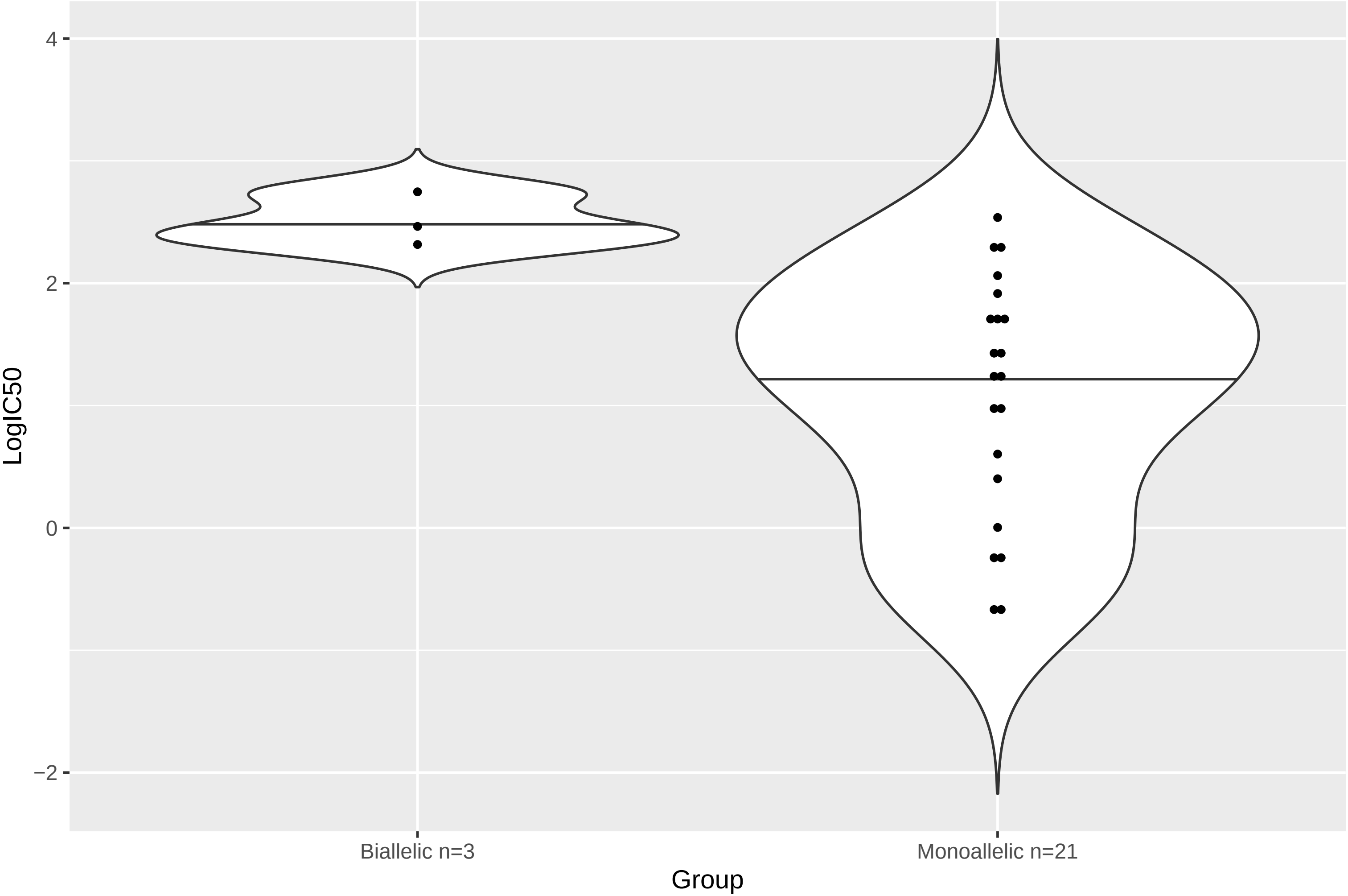

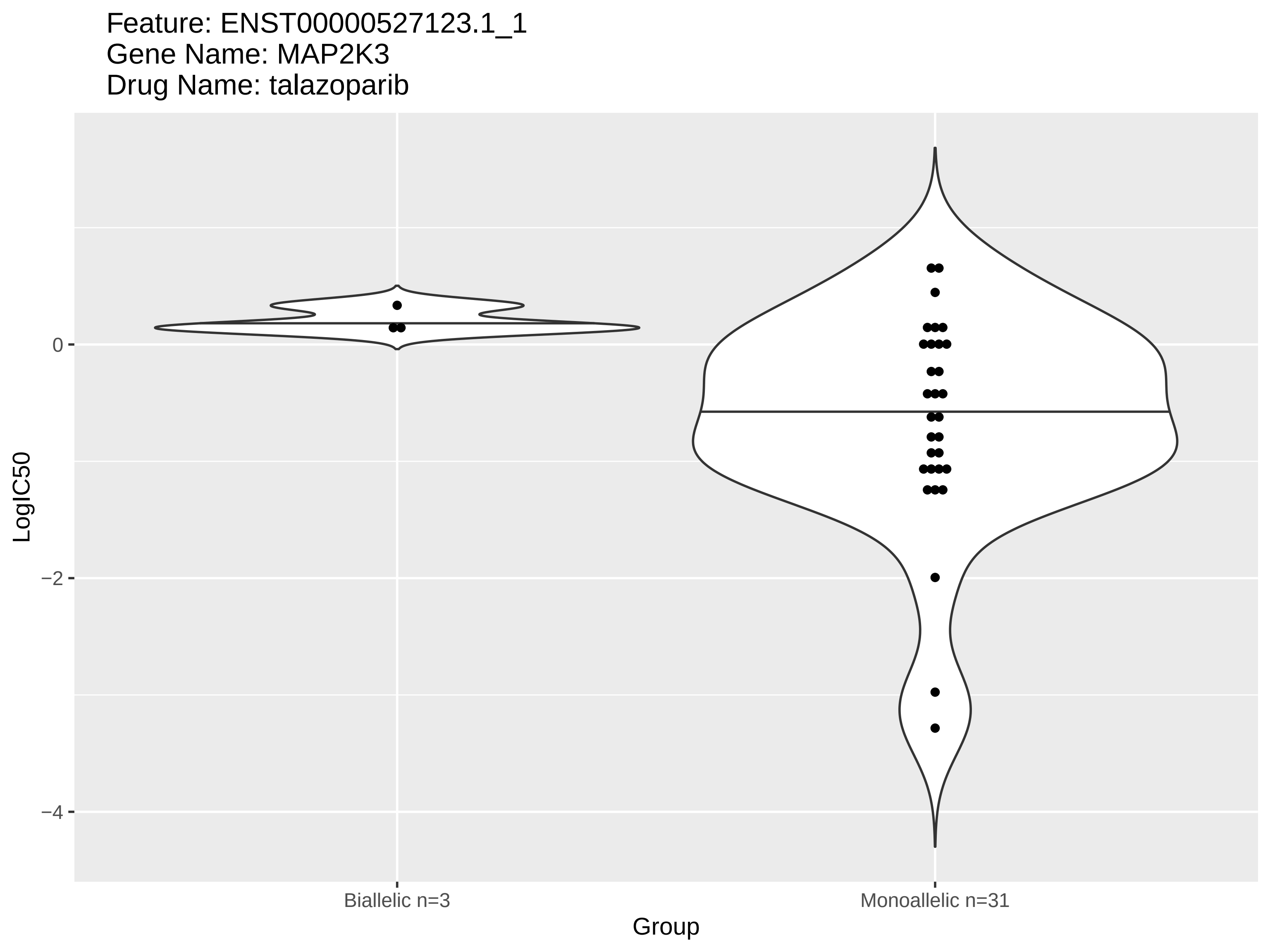

Feature: ENST00000527123.1_1
Gene Name: MAP2K3
Drug Name: talazoparib
LogIC50
0
−2
−4
Biallelic n=3
Monoallelic n=31
Group

Feature: ENST00000432629.1_1
Gene Name: RPL12P4
Drug Name: AZD7969

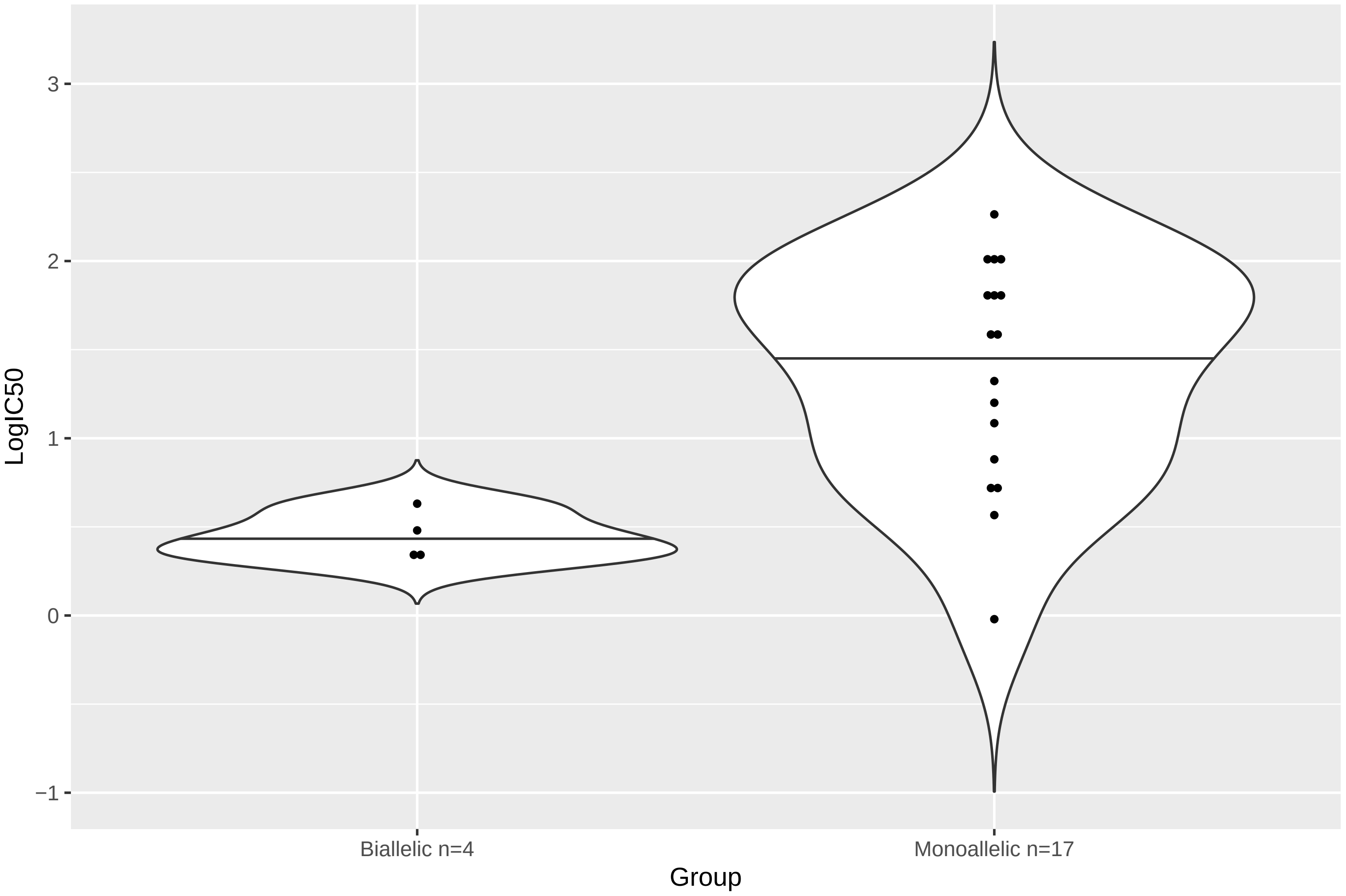

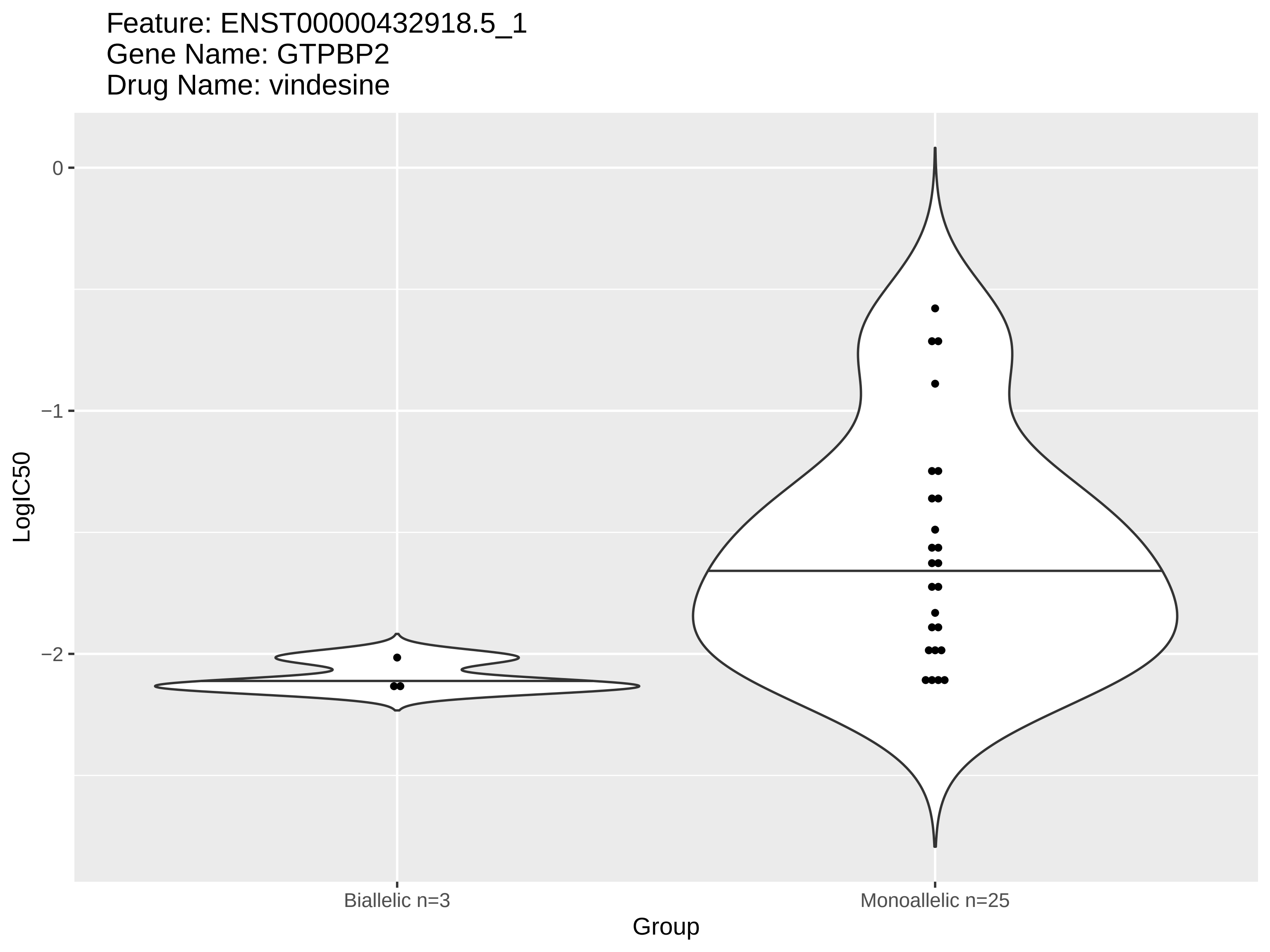
Feature: ENST00000432918.5_1
Gene Name: GTPBP2
Drug Name: vindesine
0
−1
−2
LogIC50
Biallelic n=3
Monoallelic n=25
Group

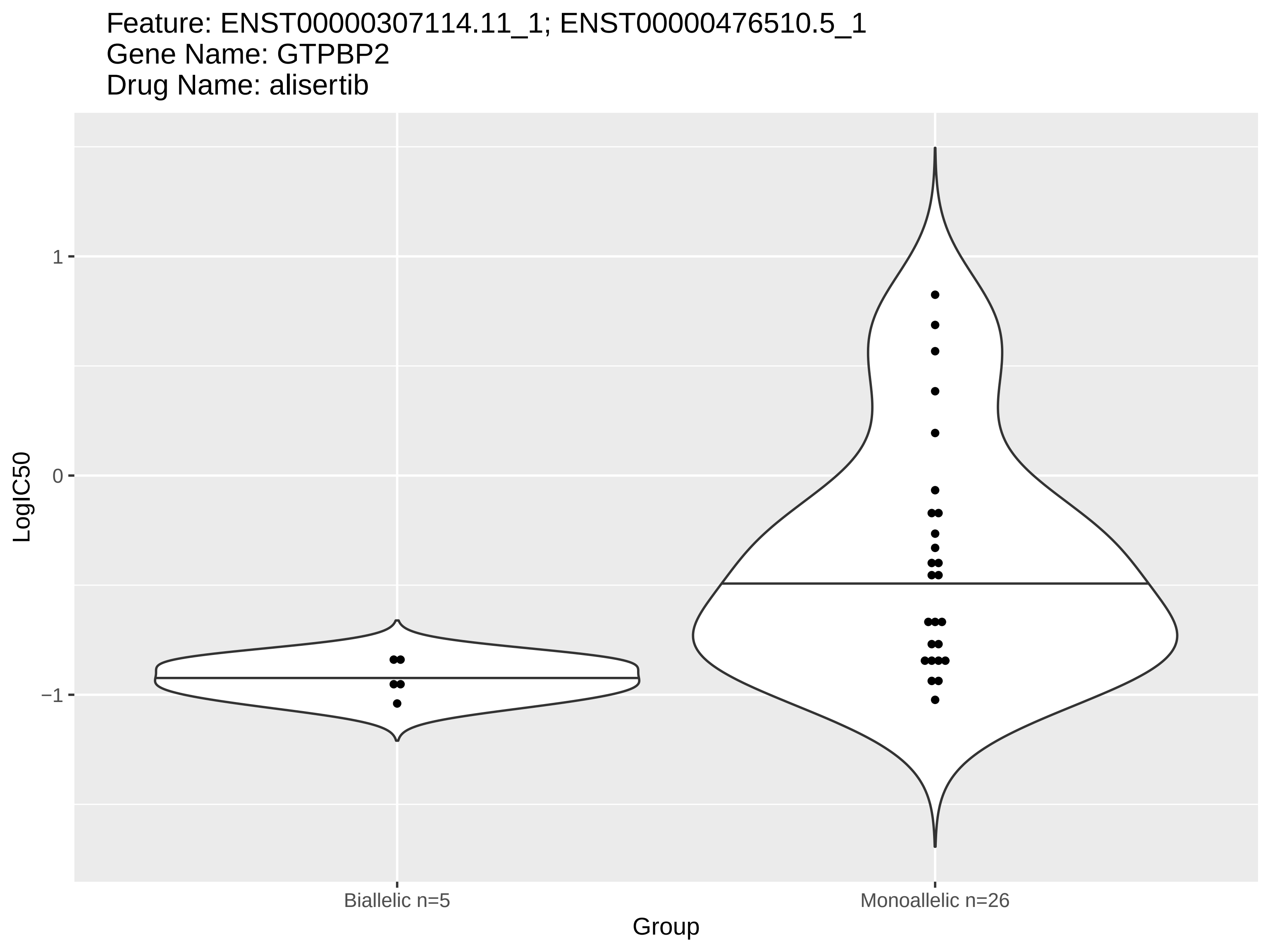

Feature: ENST00000307114.11_1; ENST00000476510.5_1
Gene Name: GTPBP2
Drug Name: alisertib
LogIC50
1
0
−1
Biallelic n=5
Monoallelic n=26
Group

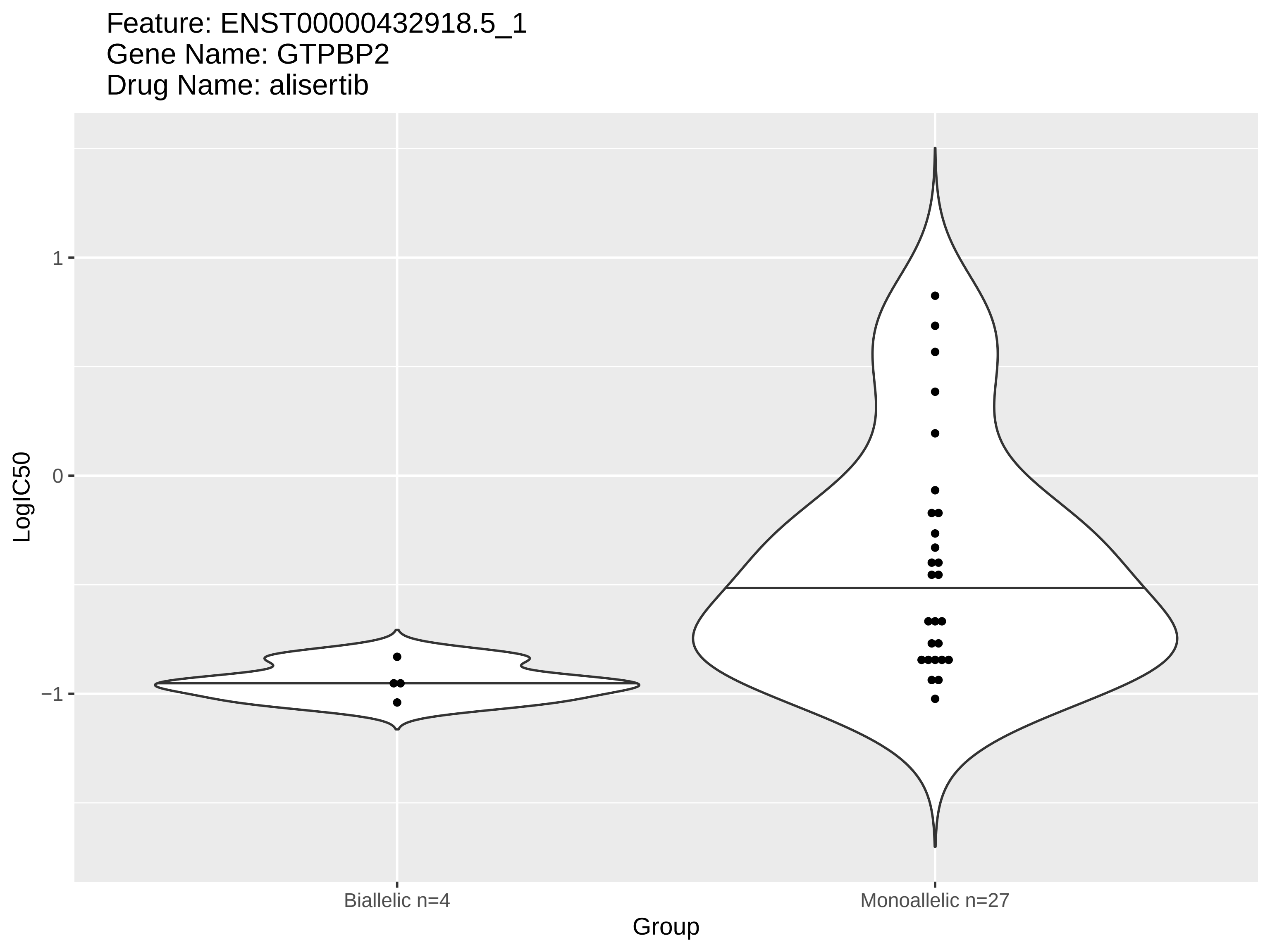
Feature: ENST00000432918.5_1
Gene Name: GTPBP2
Drug Name: alisertib
LogIC50
1
0
−1
Biallelic n=4
Monoallelic n=27
Group

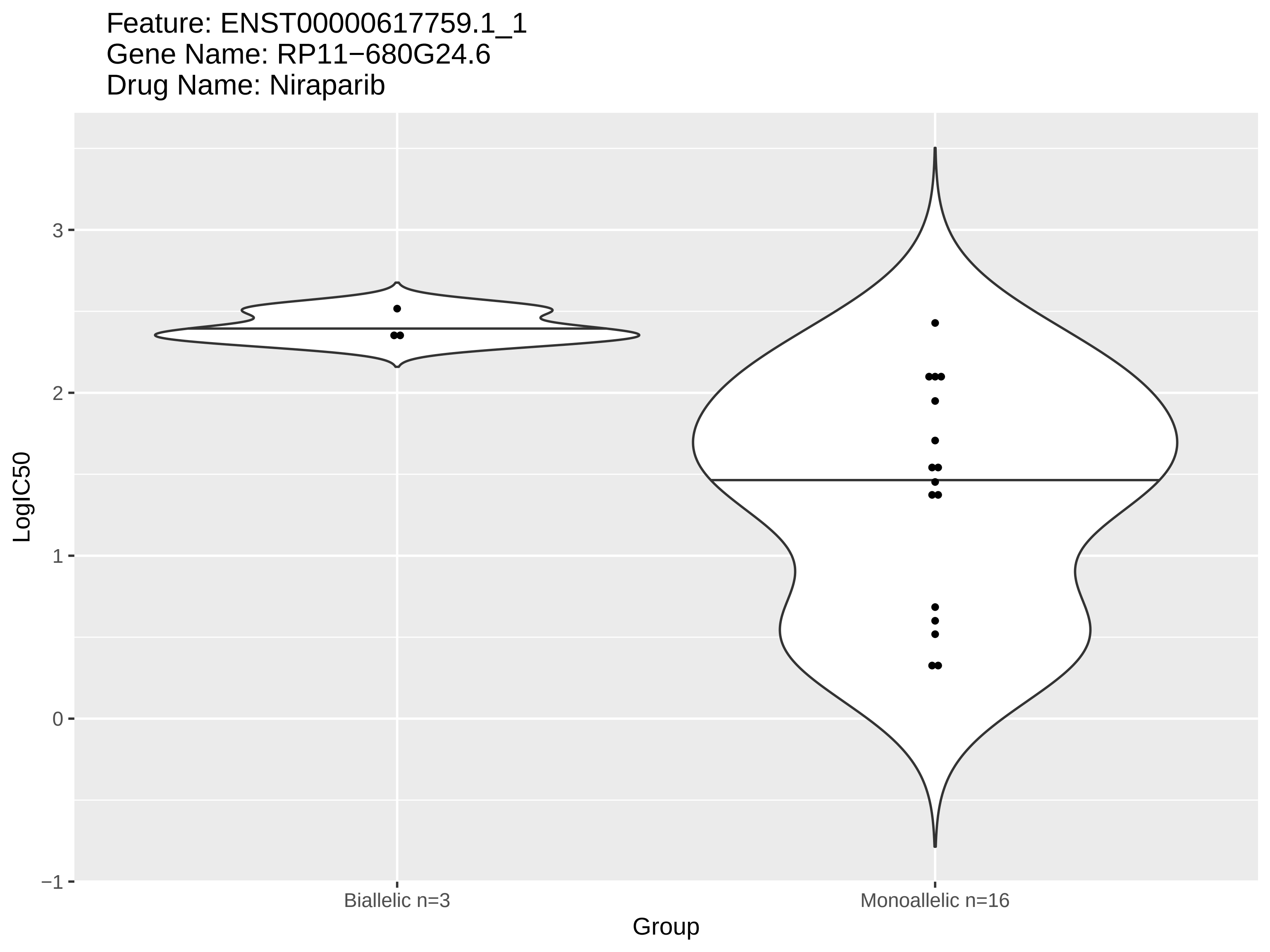

Feature: ENST00000617759.1_1
Gene Name: RP11−680G24.6
Drug Name: Niraparib
LogIC50
3
2
1
0
−1
Biallelic n=3
Monoallelic n=16
Group

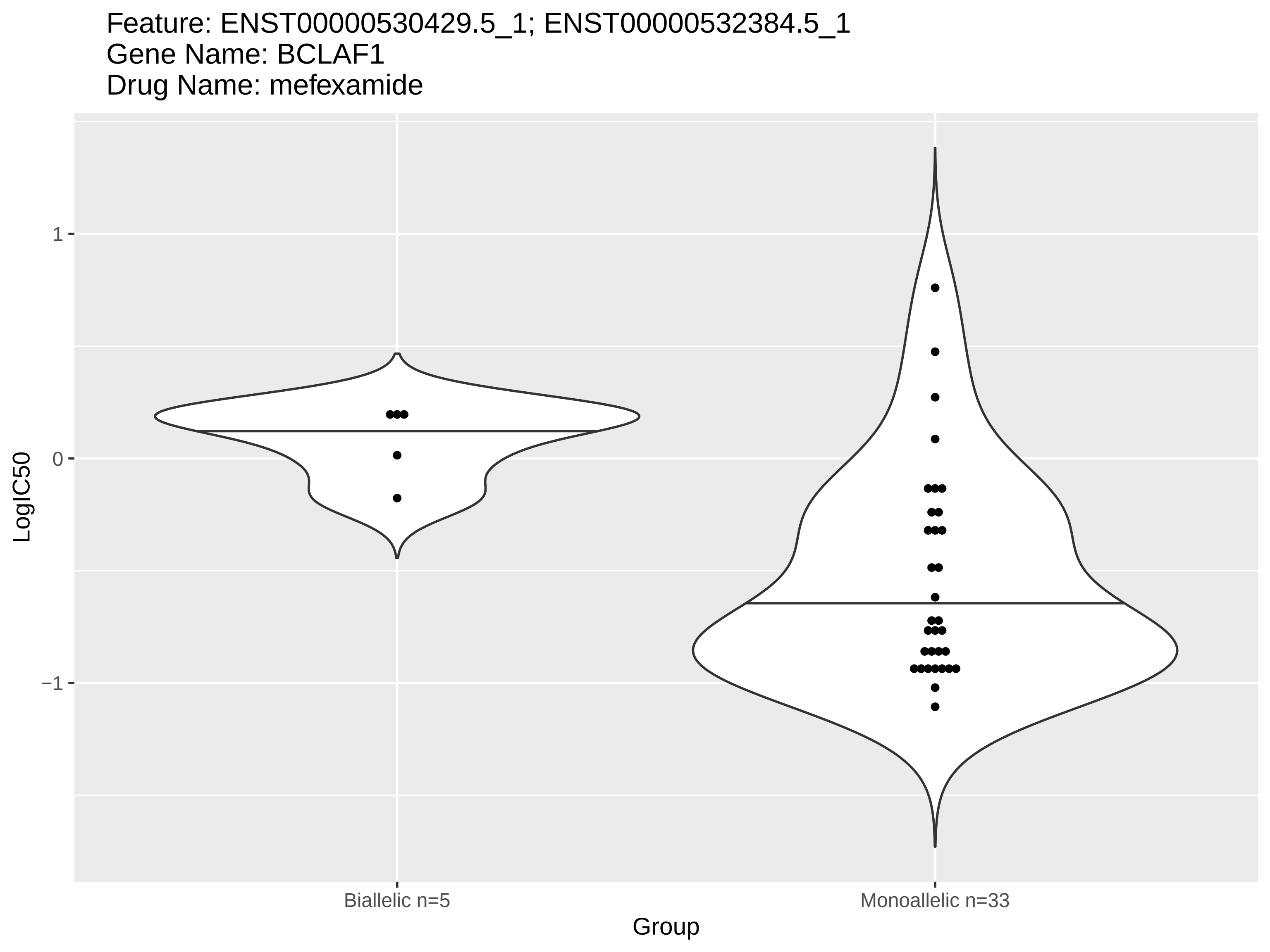

Feature: ENST00000530429.5_1; ENST00000532384.5_1
Gene Name: BCLAF1
Drug Name: mefexamide
LogIC50
1
0
−1
Biallelic n=5
Monoallelic n=33
Group

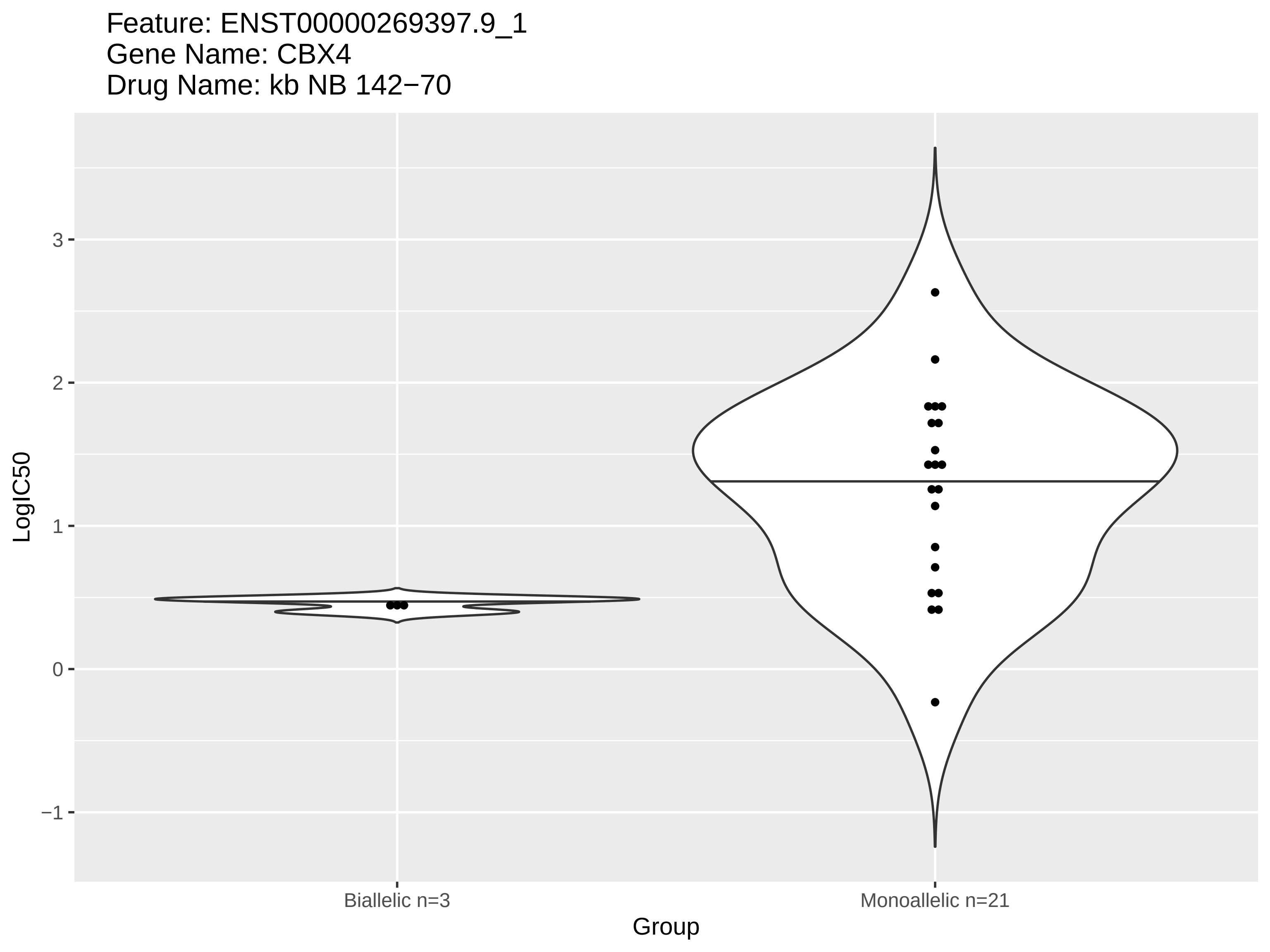

Feature: ENST00000269397.9_1
Gene Name: CBX4
Drug Name: kb NB 142−70
LogIC50
3
2
1
0
−1
Biallelic n=3
Monoallelic n=21
Group

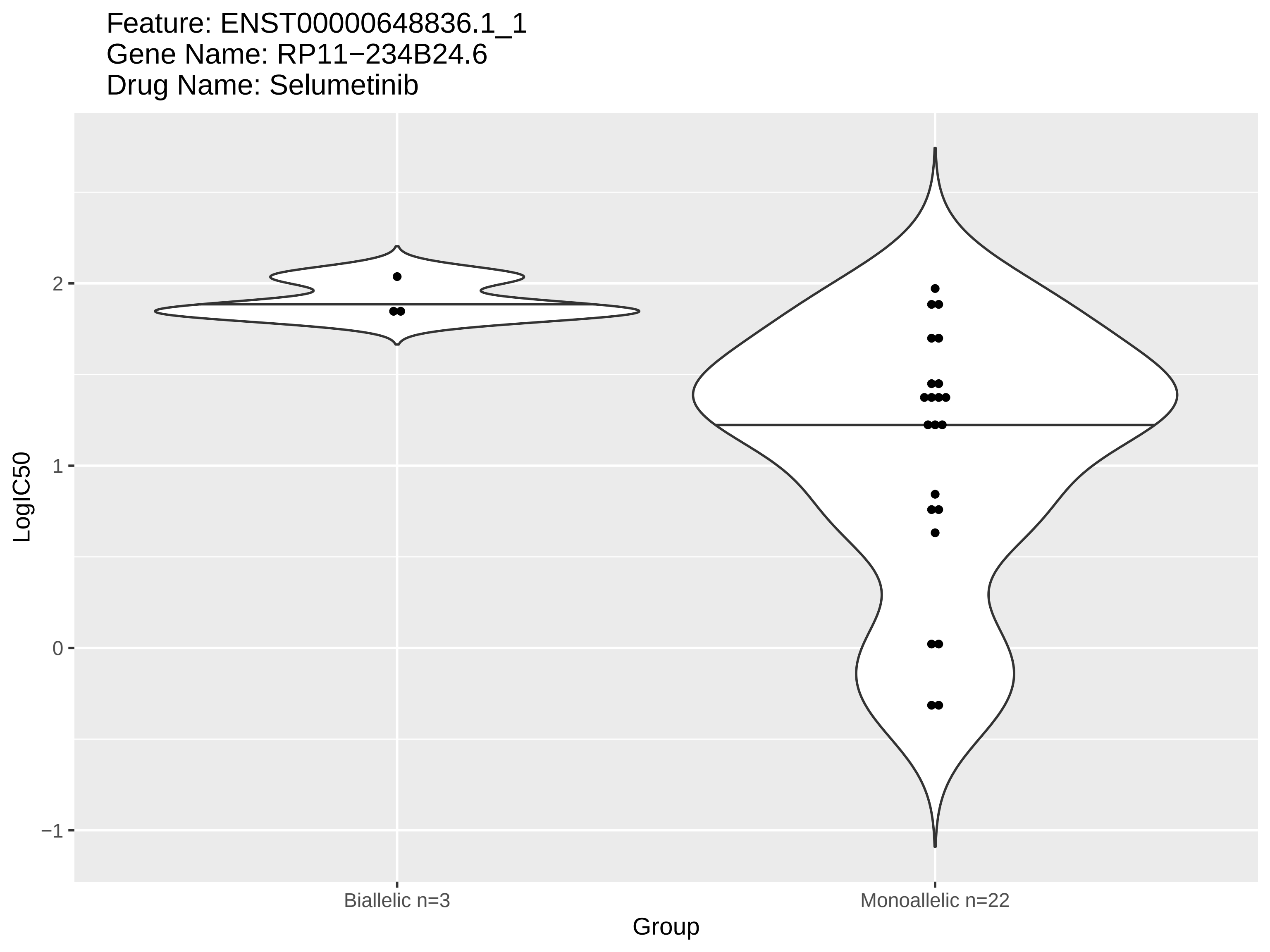
Feature: ENST00000648836.1_1
Gene Name: RP11−234B24.6
Drug Name: Selumetinib
LogIC50
2
1
0
−1
Biallelic n=3
Monoallelic n=22
Group

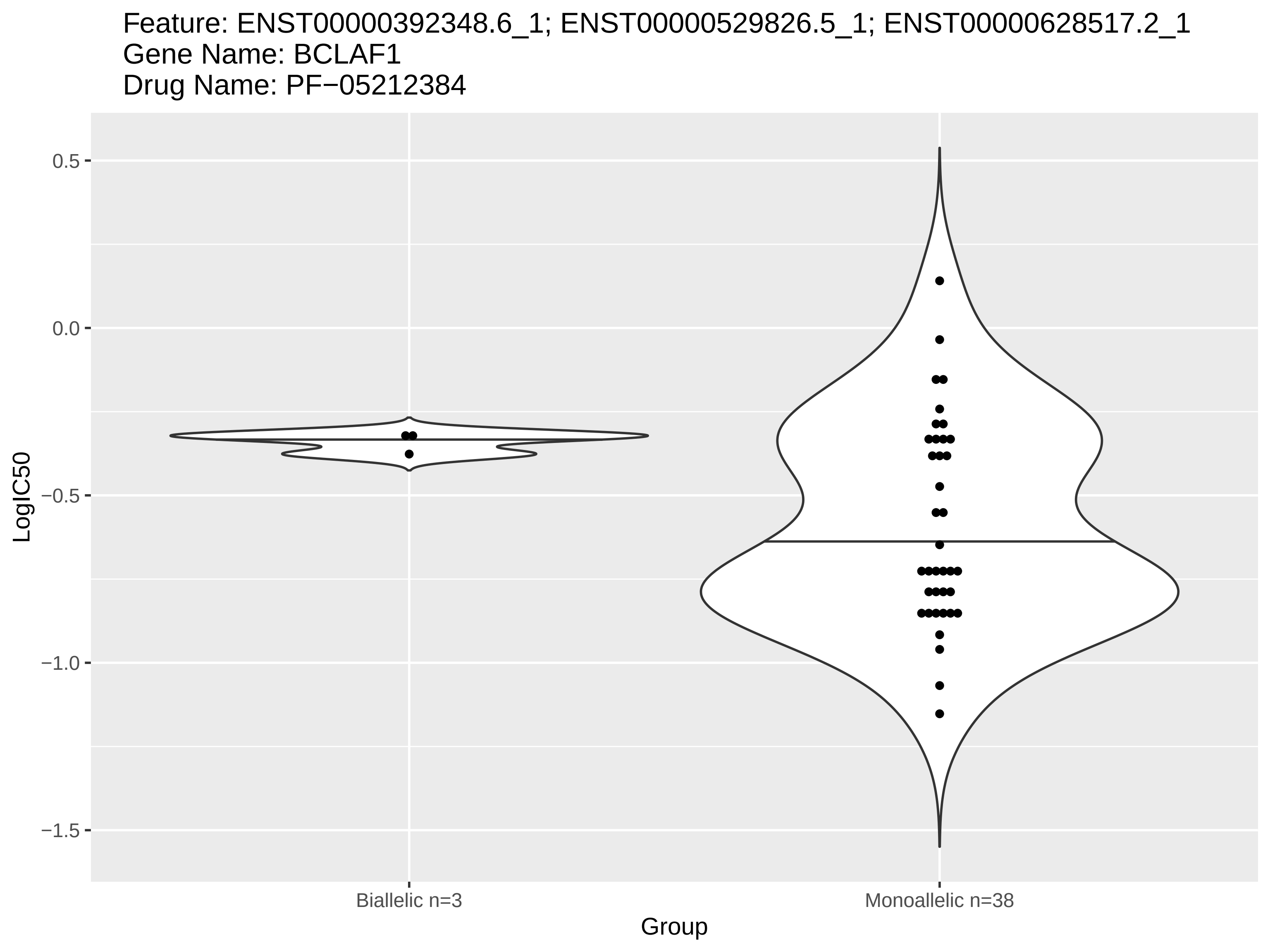

Feature: ENST00000392348.6_1; ENST00000529826.5_1; ENST00000628517.2_1
Gene Name: BCLAF1
Drug Name: PF−05212384
LogIC50
0.5
0.0
−0.5
−1.0
−1.5
Biallelic n=3
Monoallelic n=38
Group

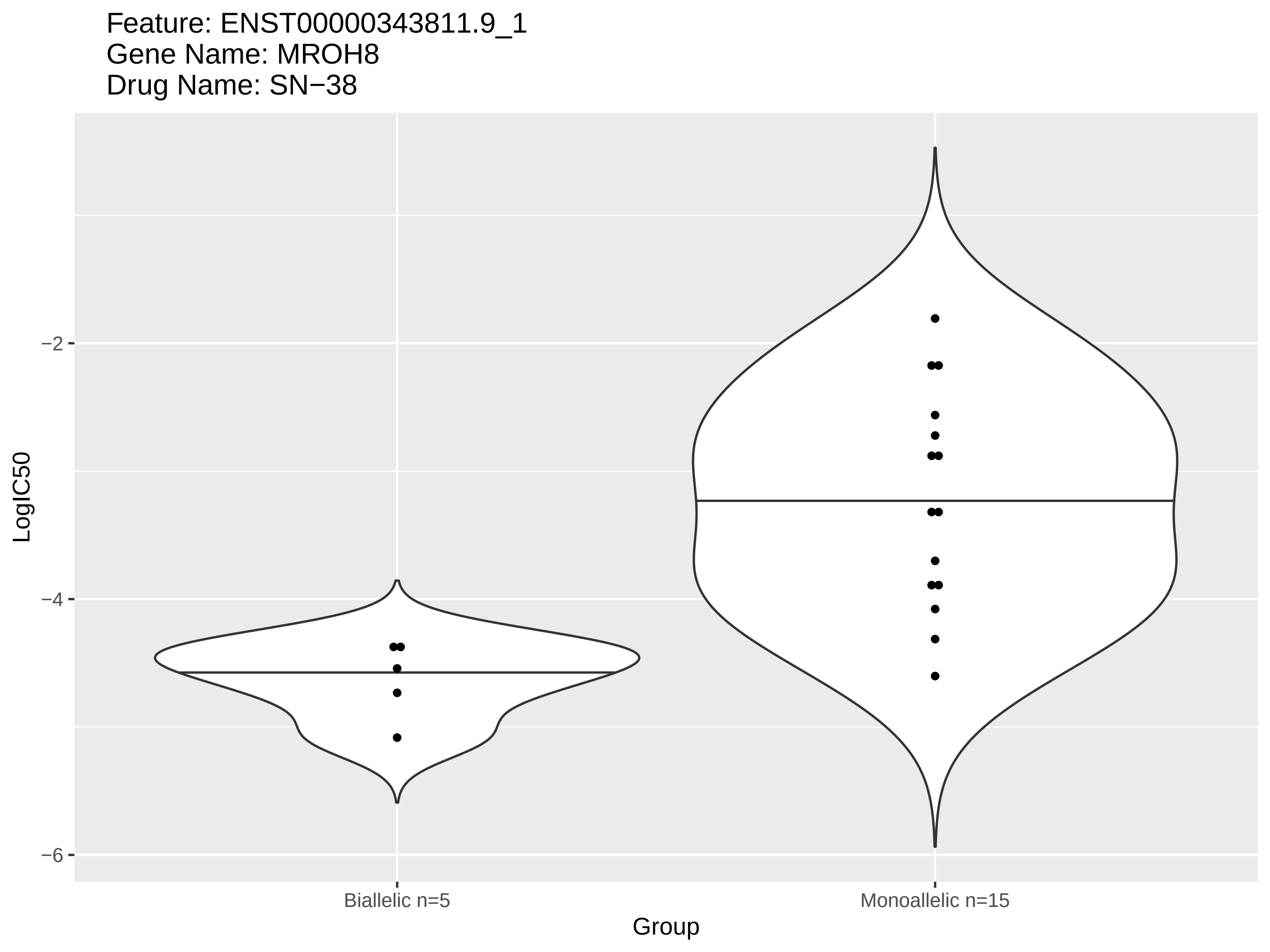
Feature: ENST00000343811.9_1
Gene Name: MROH8
Drug Name: SN−38
LogIC50
−2
−4
−6
Biallelic n=5
Monoallelic n=15
Group

Feature: ENST00000400440.6_1
Gene Name: MROH8
Drug Name: SN−38

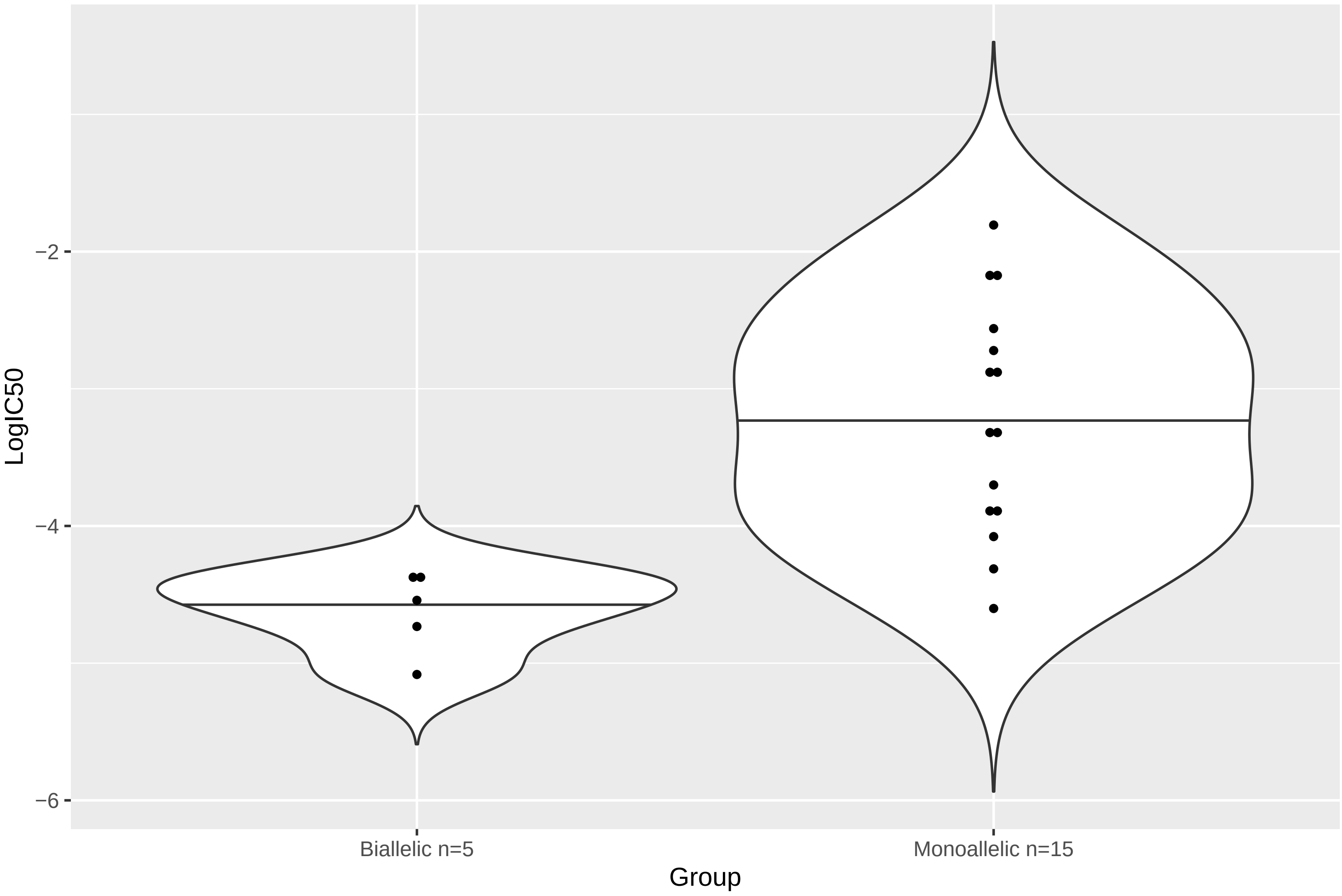

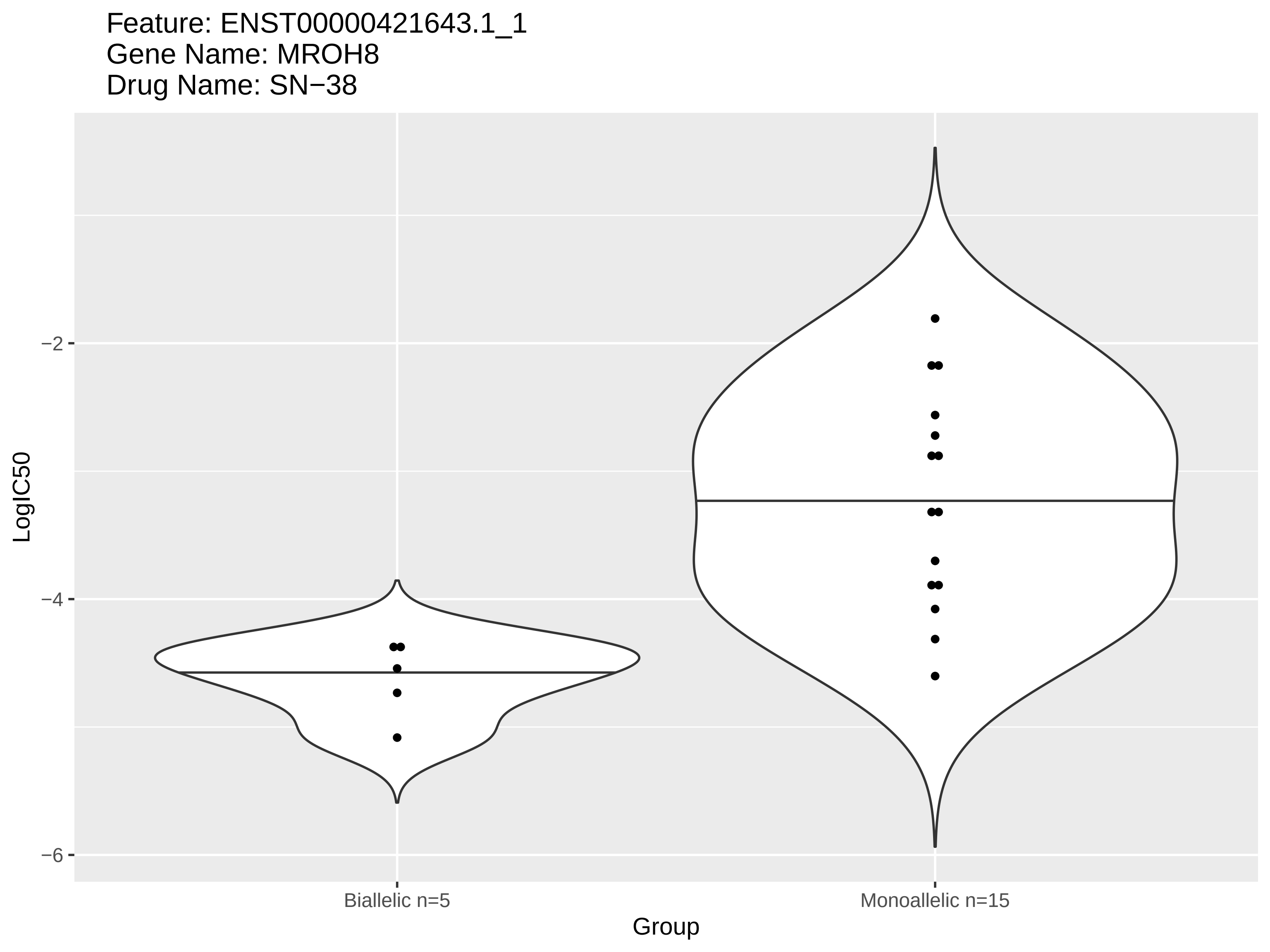
Feature: ENST00000421643.1_1
Gene Name: MROH8
Drug Name: SN−38
LogIC50
−2
−4
−6
Biallelic n=5
Monoallelic n=15
Group

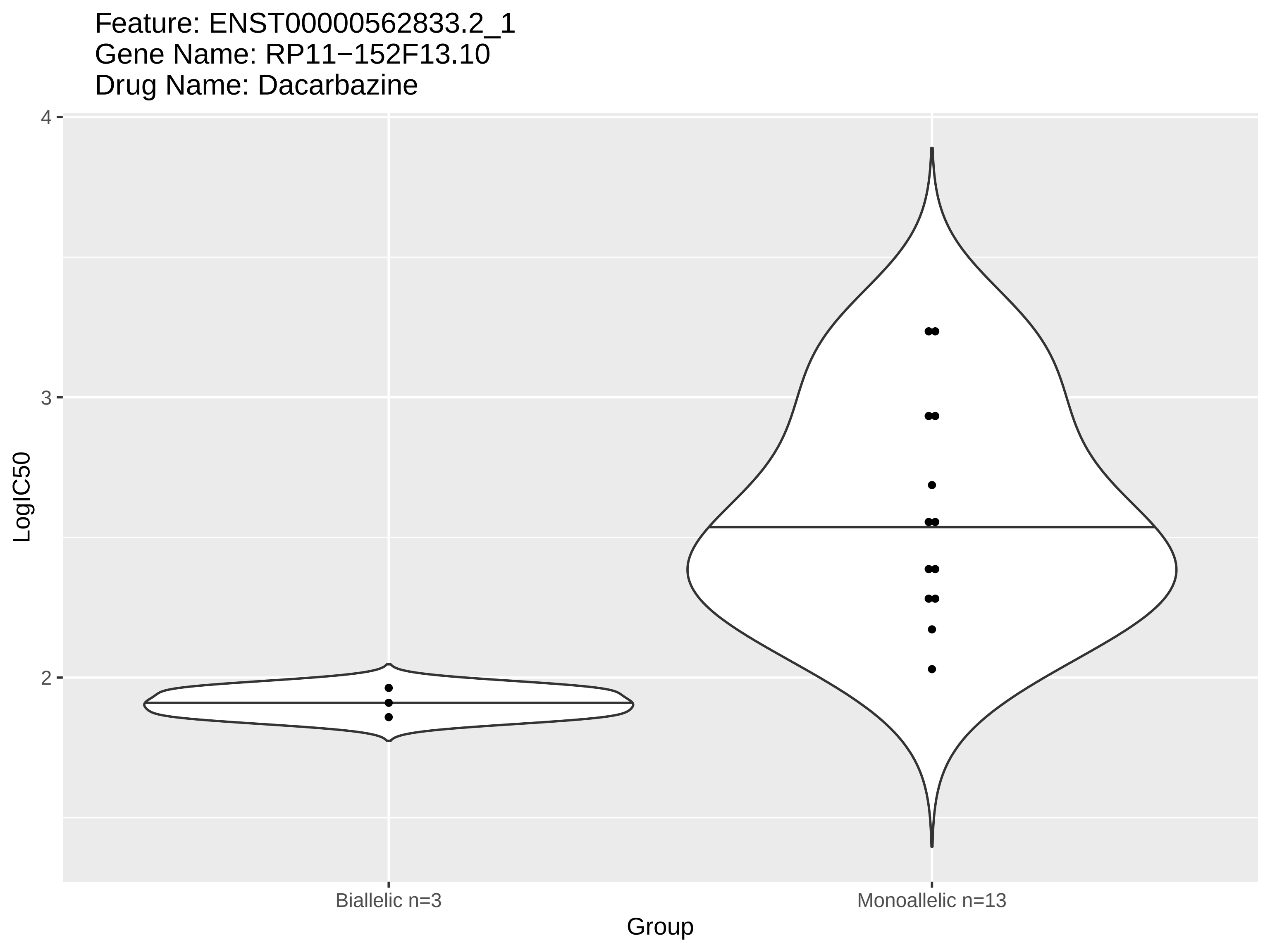
Feature: ENST00000562833.2_1
Gene Name: RP11−152F13.10
Drug Name: Dacarbazine
4
3
2
LogIC50
Biallelic n=3
Monoallelic n=13
Group

Feature: ENST00000470544.2_1
Gene Name: RPL7AP31
Drug Name: HG−5−113−01

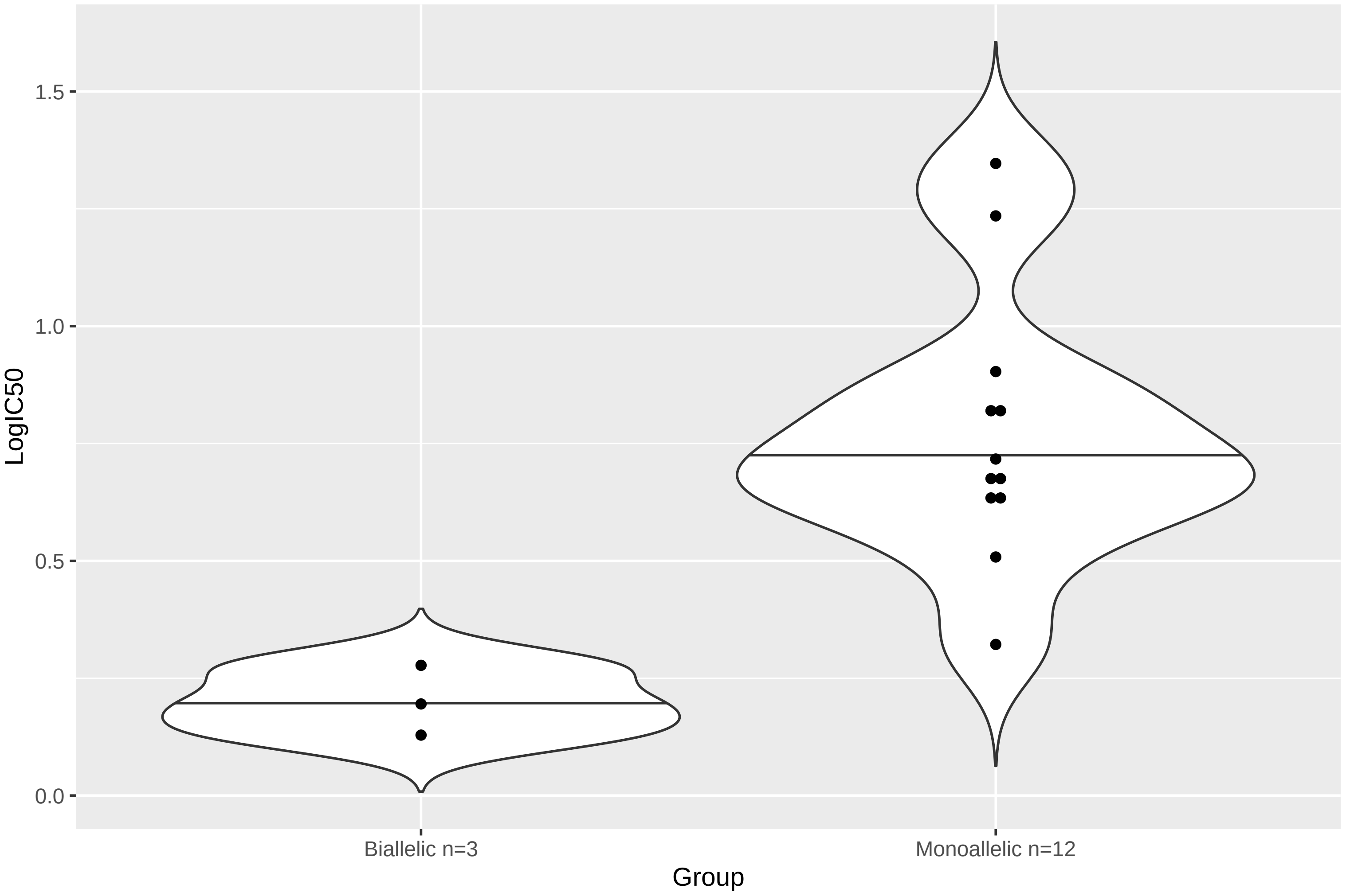

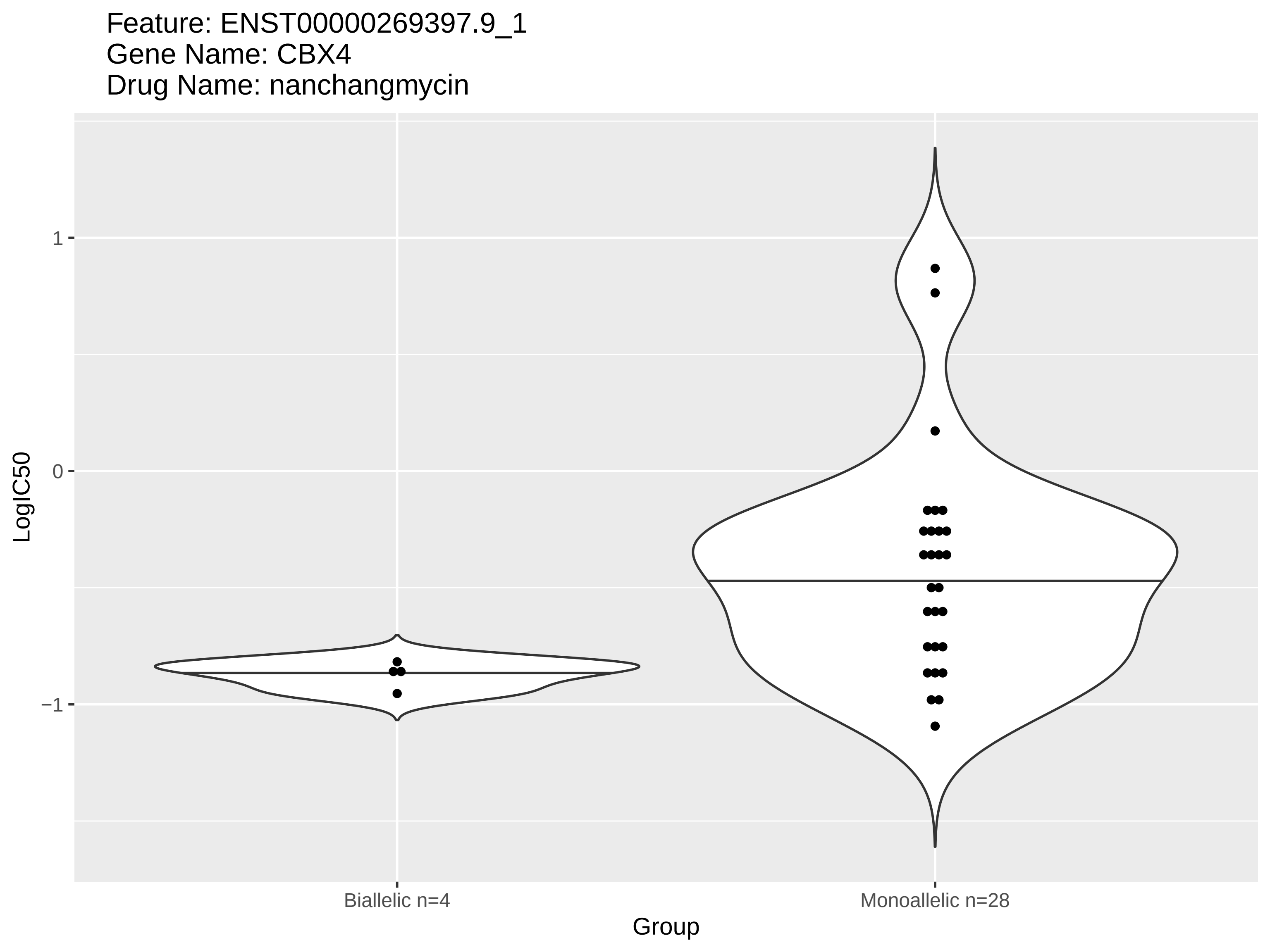

Feature: ENST00000269397.9_1
Gene Name: CBX4
Drug Name: nanchangmycin
1
0
−1
LogIC50
Biallelic n=4
Monoallelic n=28
Group

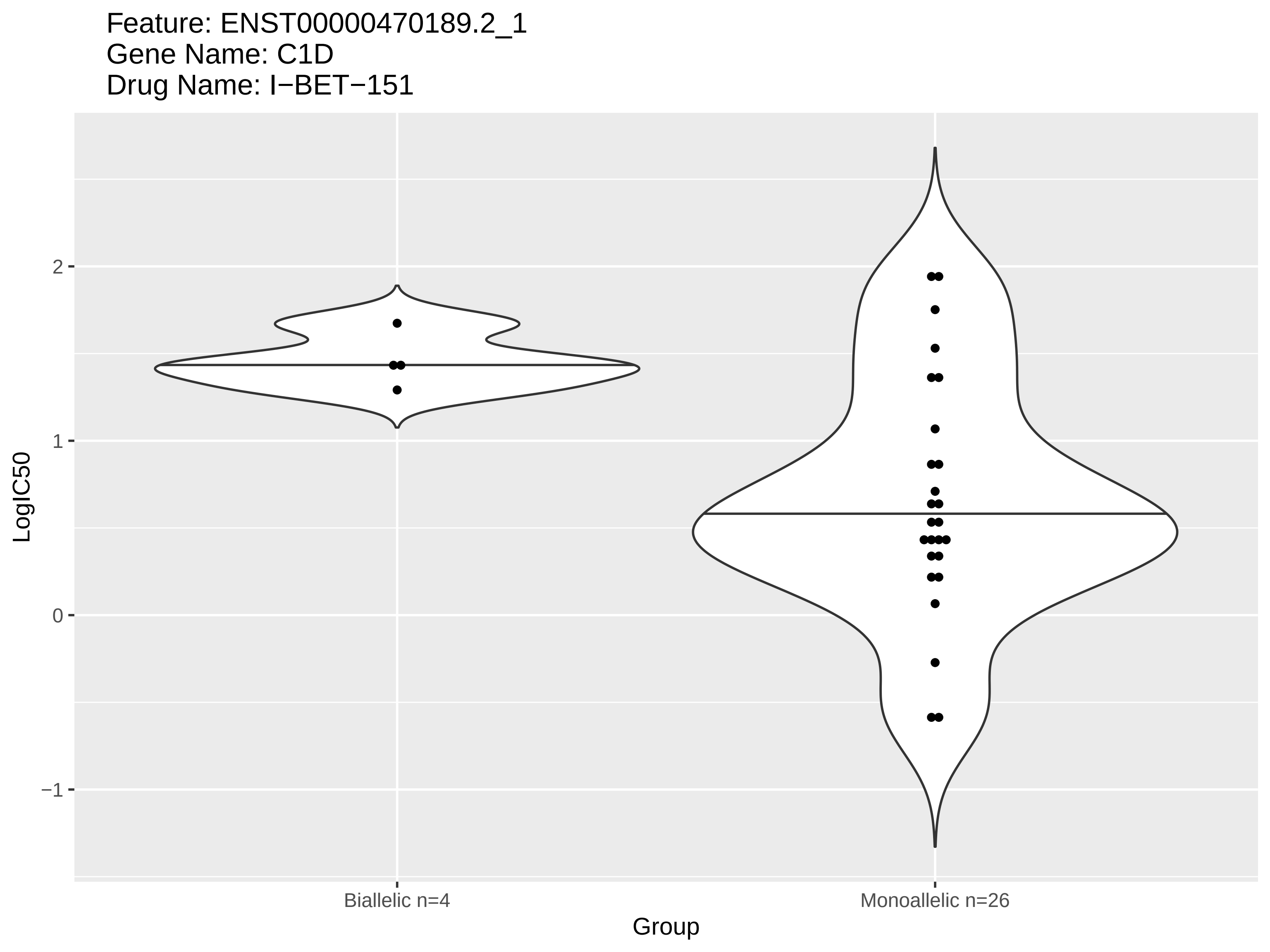
Feature: ENST00000470189.2_1
Gene Name: C1D
Drug Name: I−BET−151
2
1
0
−1
LogIC50
Biallelic n=4
Monoallelic n=26
Group

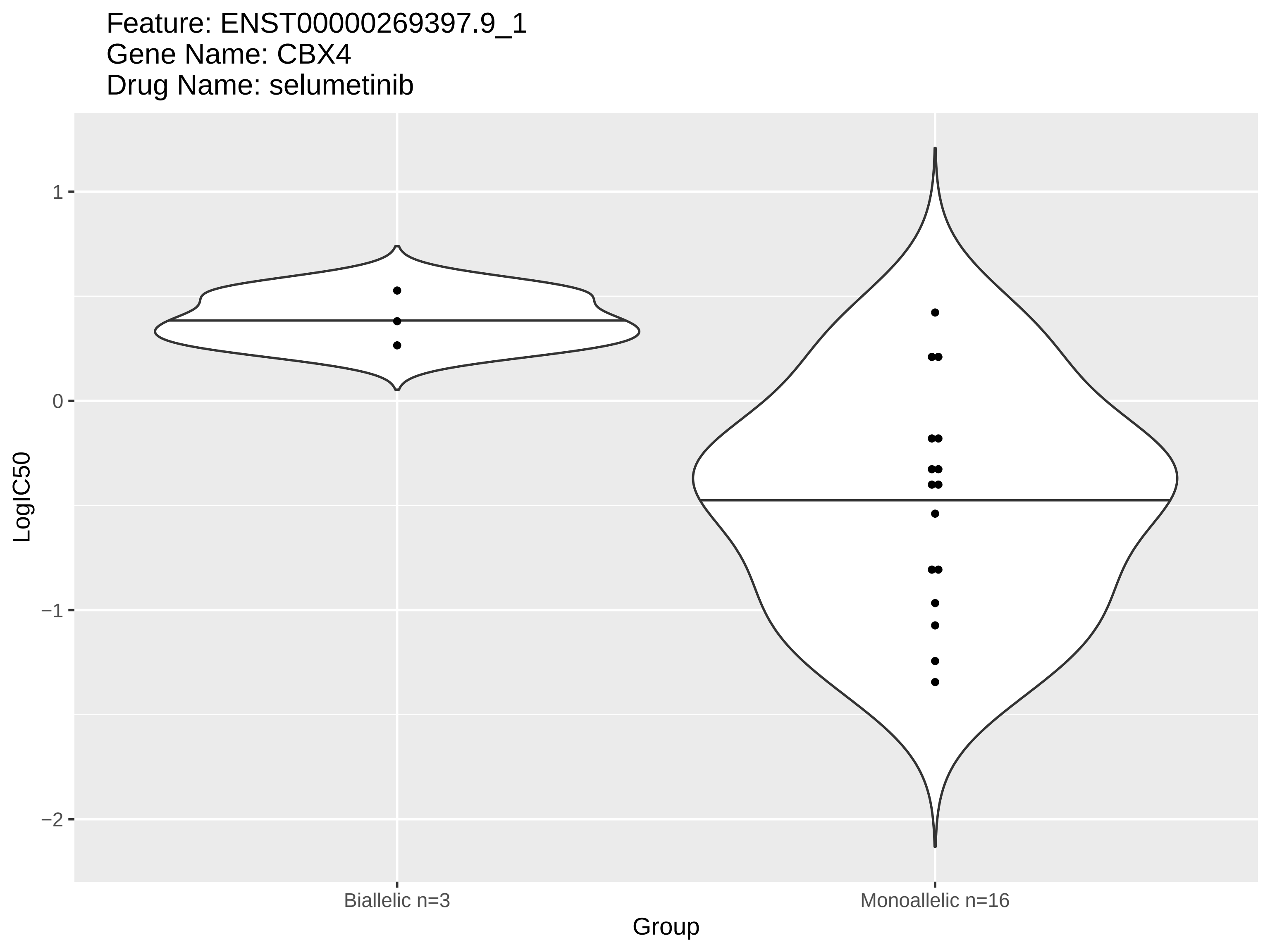
Feature: ENST00000269397.9_1
Gene Name: CBX4
Drug Name: selumetinib
LogIC50
1
0
−1
−2
Biallelic n=3
Monoallelic n=16
Group

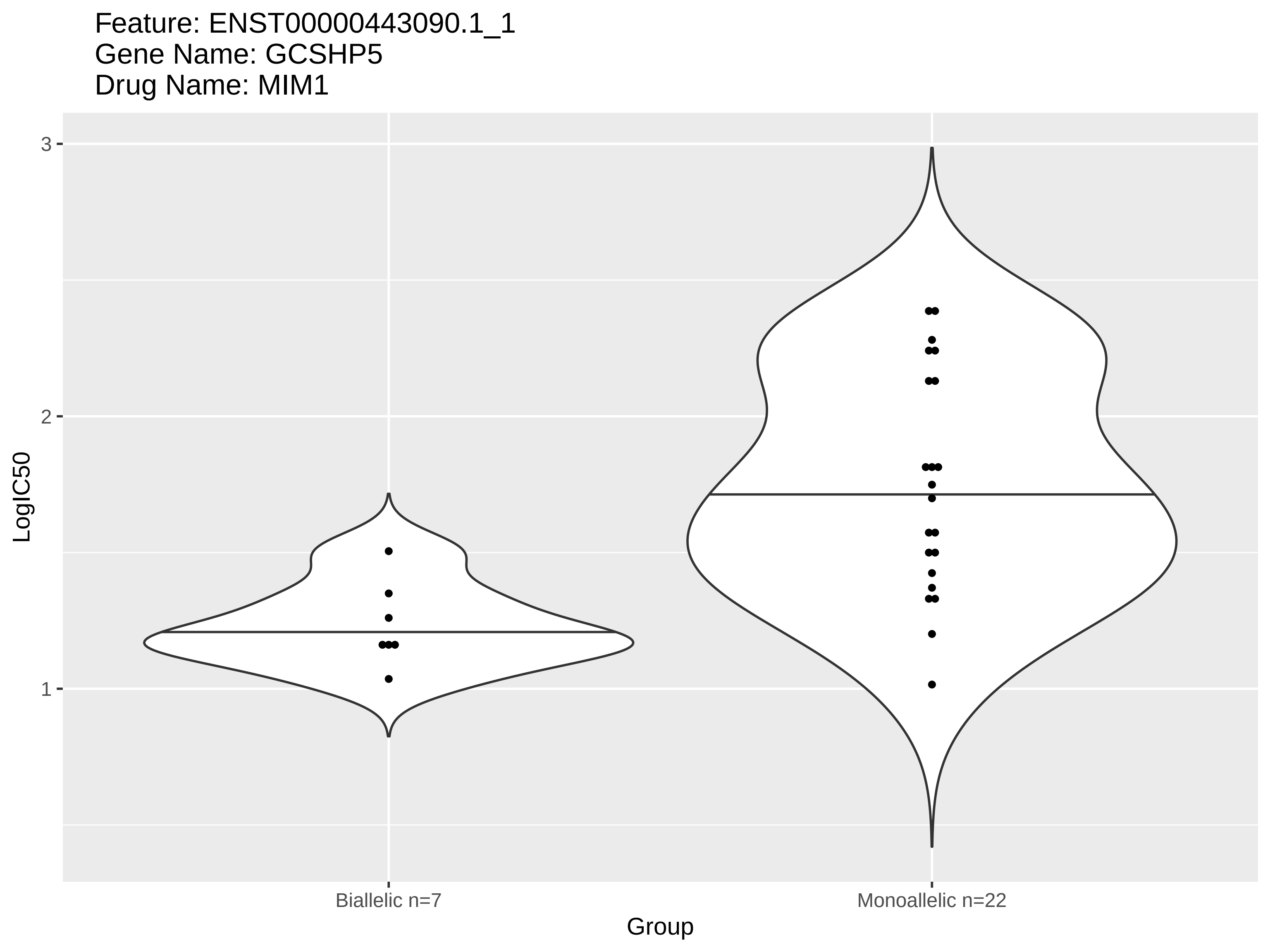
Feature: ENST00000443090.1_1
Gene Name: GCSHP5
Drug Name: MIM1
3
2
1
LogIC50
Biallelic n=7
Monoallelic n=22
Group

Feature: ENST00000307114.11_1; ENST00000476510.5_1
Gene Name: GTPBP2
Drug Name: QL−XI−92

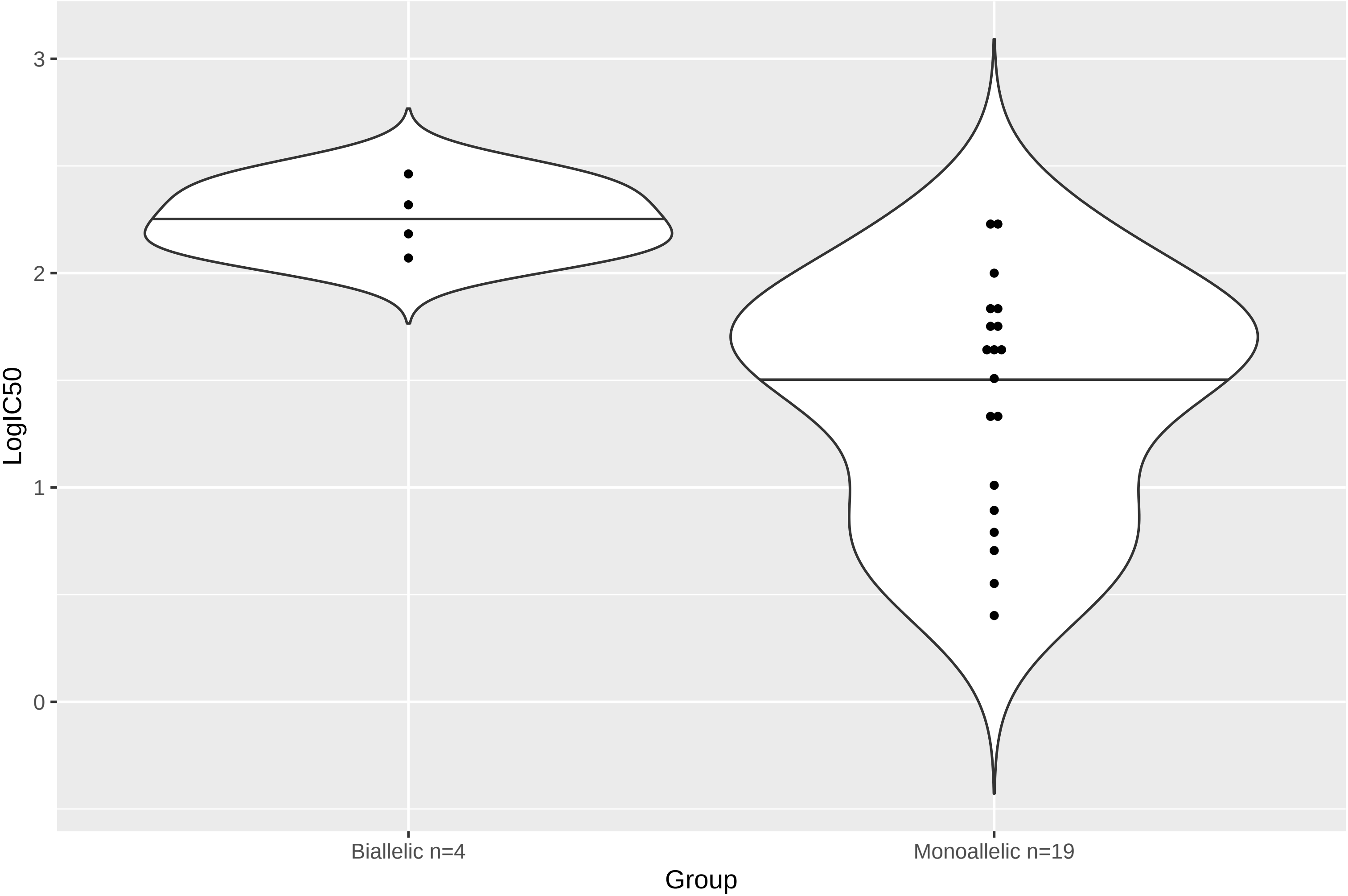

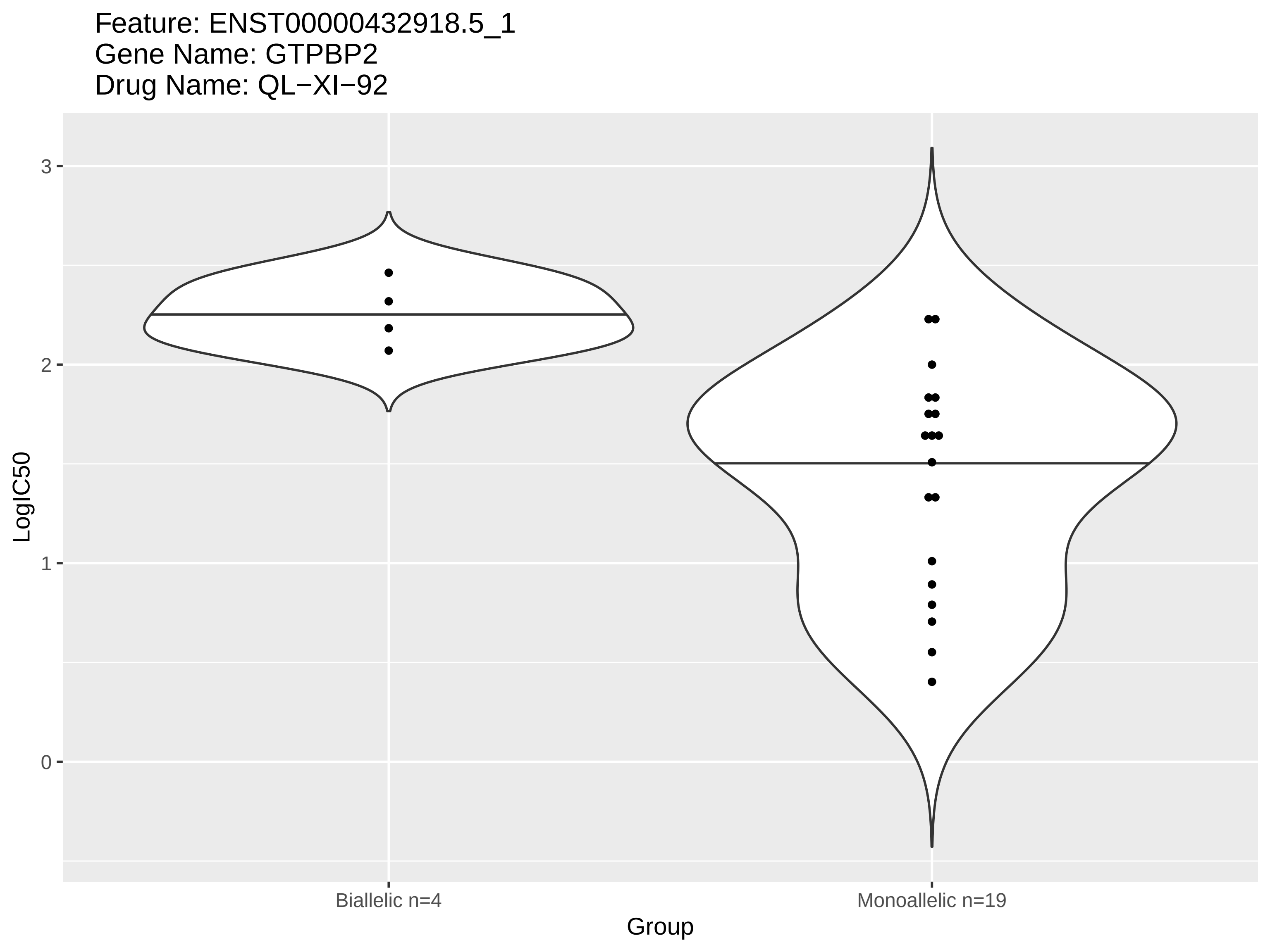
Feature: ENST00000432918.5_1
Gene Name: GTPBP2
Drug Name: QL−XI−92
LogIC50
3
2
1
0
Biallelic n=4
Monoallelic n=19
Group

Feature: ENST00000596415.1_1
Gene Name: NDUFV2P1
Drug Name: SGC0946

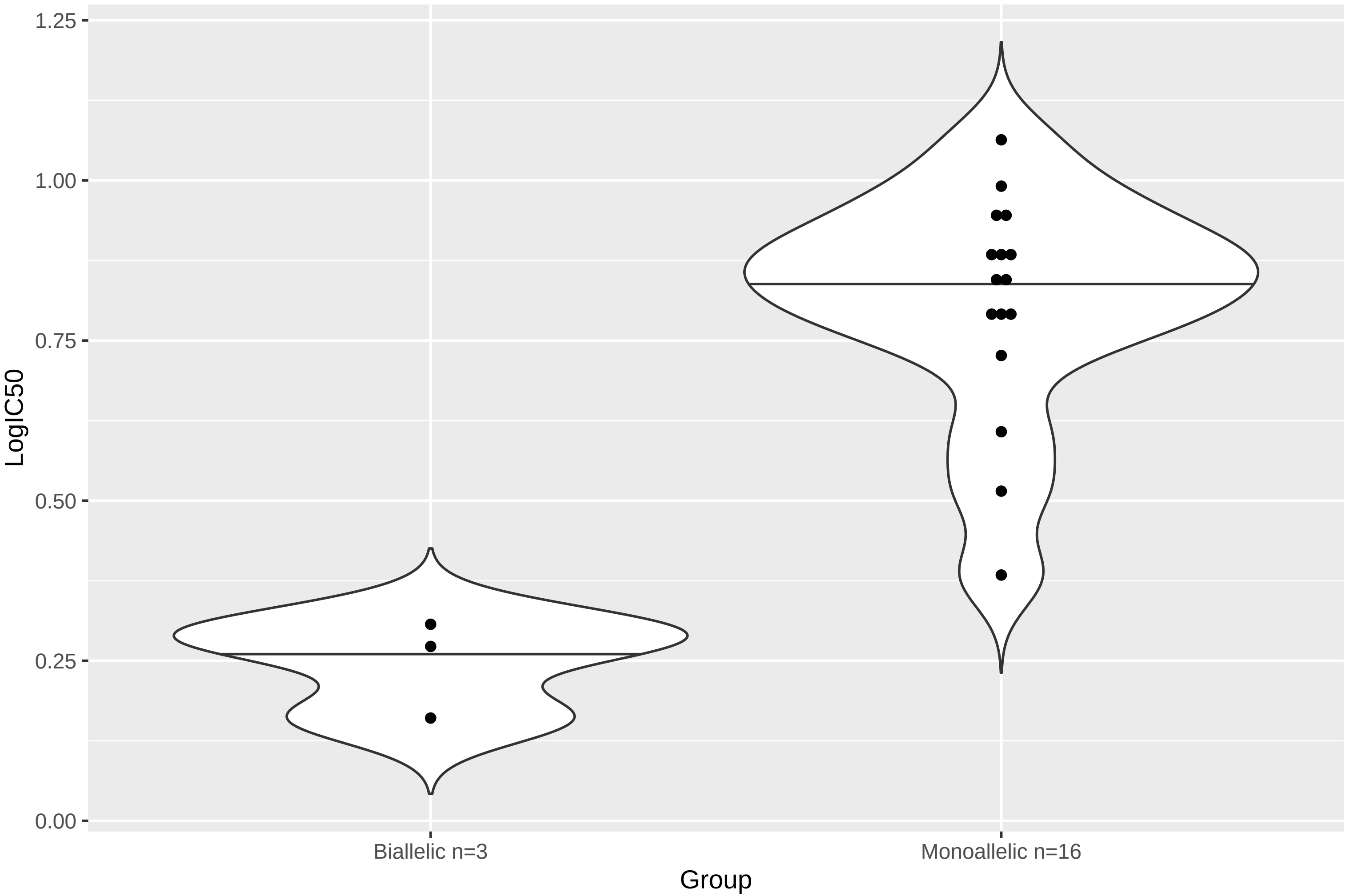

Feature: ENST00000637553.1_1
Gene Name: RP3−401D24.1
Drug Name: A−674563

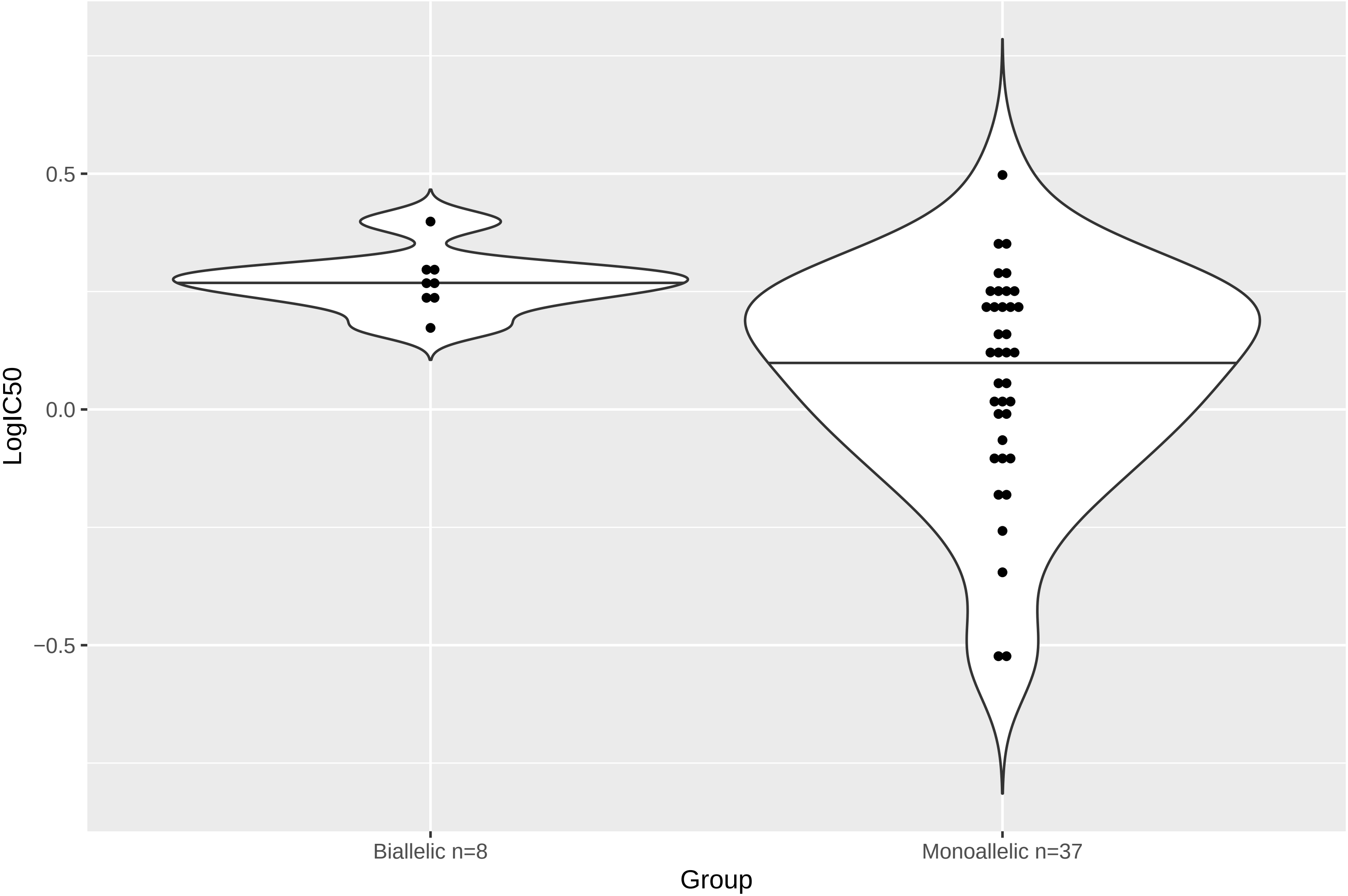

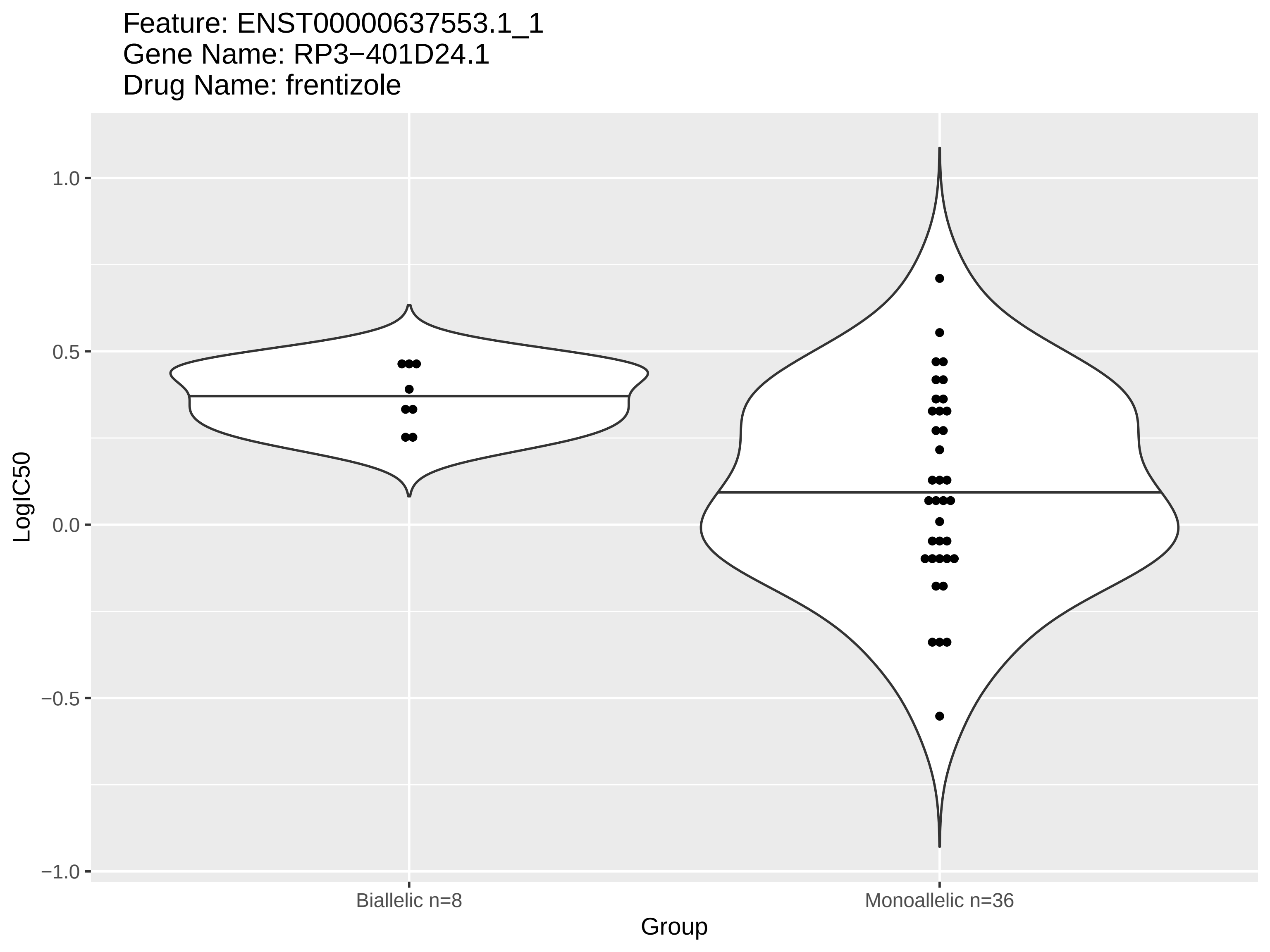

Feature: ENST00000637553.1_1
Gene Name: RP3−401D24.1
Drug Name: frentizole
LogIC50
1.0
0.5
0.0
−0.5
−1.0
Biallelic n=8
Monoallelic n=36
Group

Feature: ENST00000392348.6_1; ENST00000529826.5_1; ENST00000628517.2_1
Gene Name: BCLAF1
Drug Name: fluvastatin

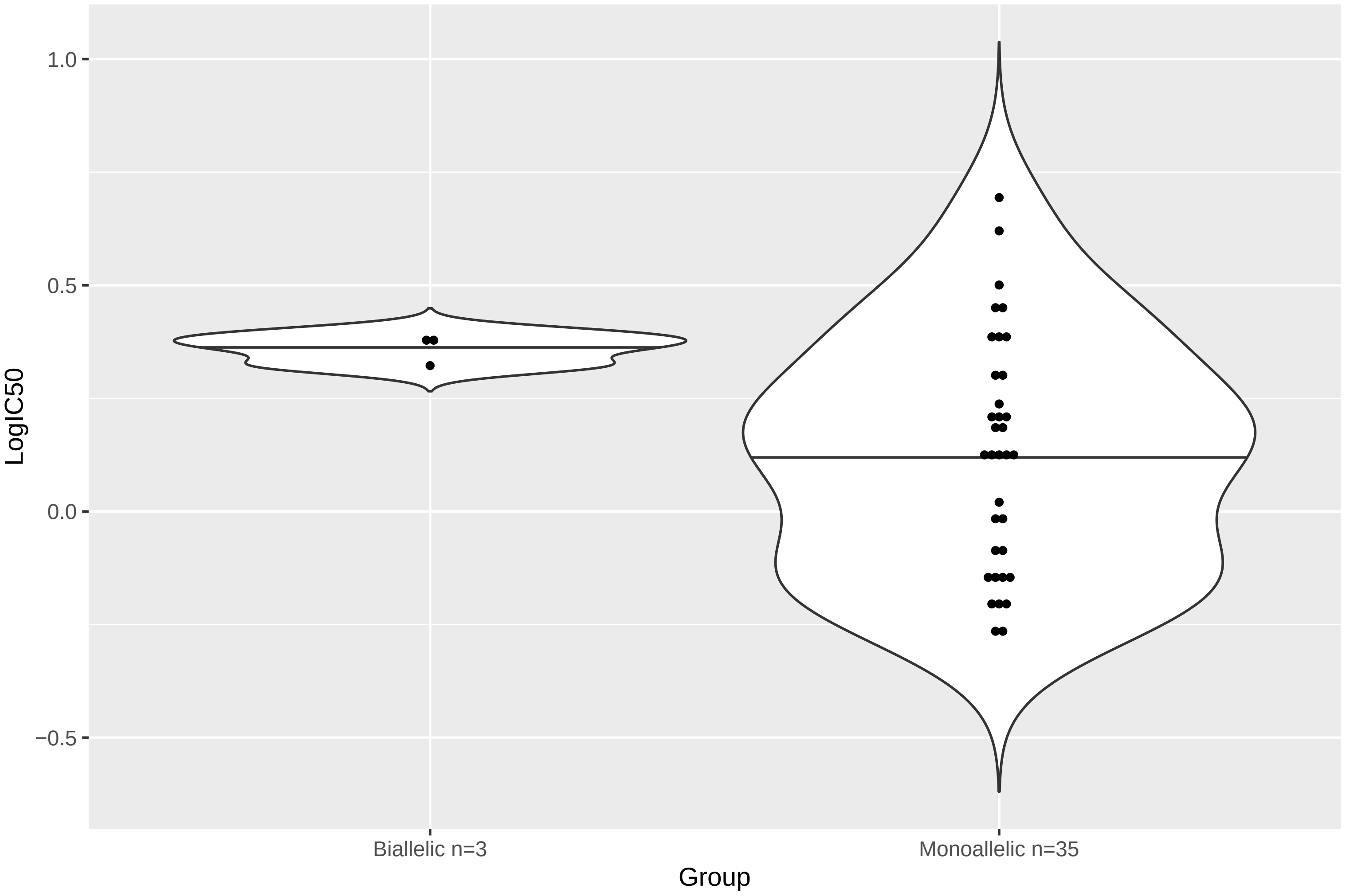

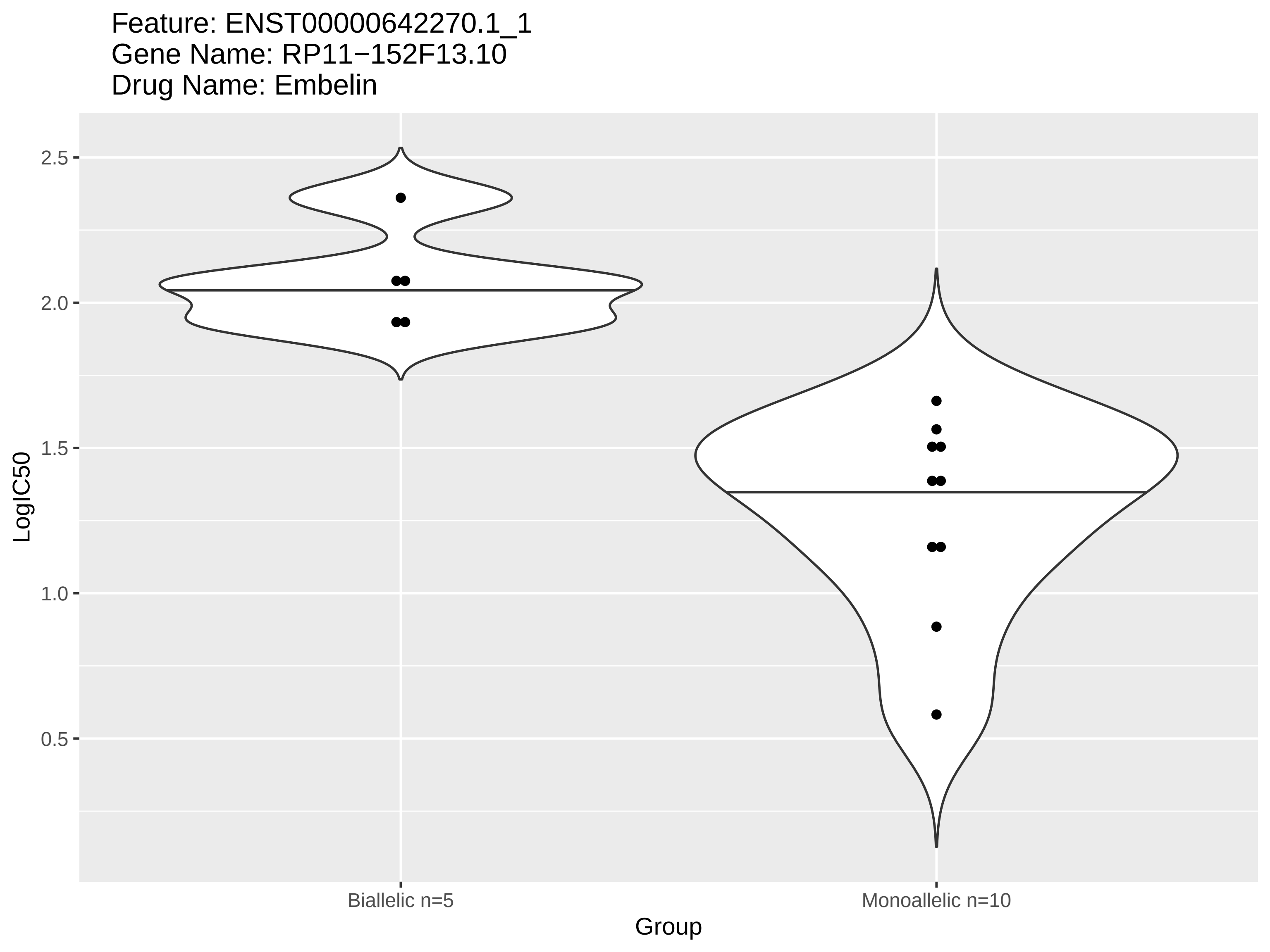

Feature: ENST00000642270.1_1
Gene Name: RP11−152F13.10
Drug Name: Embelin
LogIC50
2.5
2.0
1.5
1.0
0.5
Biallelic n=5
Monoallelic n=10
Group

Feature: ENST00000254900.10_1
Gene Name: BRD8
Drug Name: NVP−TAE684

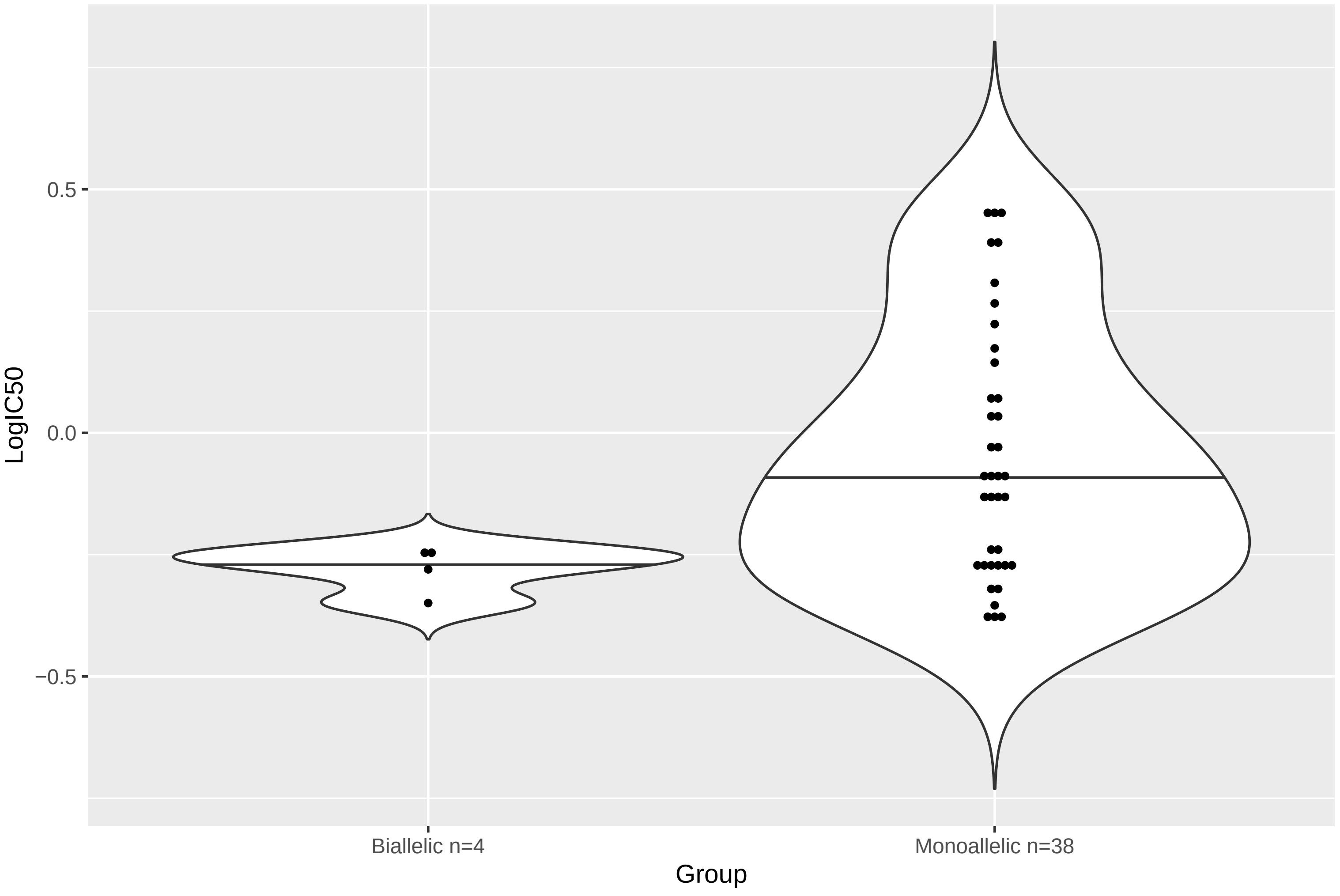

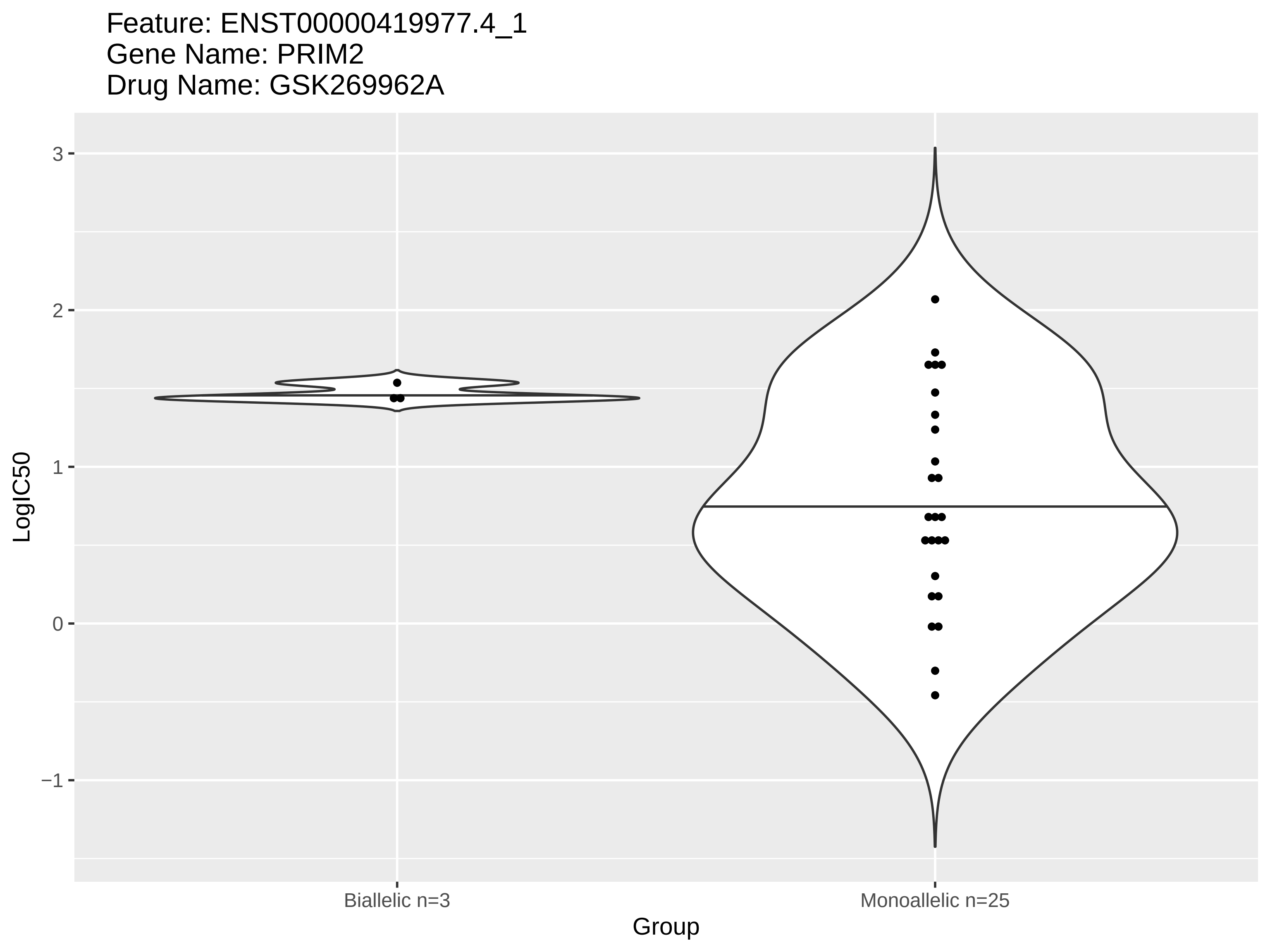

Feature: ENST00000419977.4_1
Gene Name: PRIM2
Drug Name: GSK269962A
LogIC50
3
2
1
0
−1
Biallelic n=3
Monoallelic n=25
Group

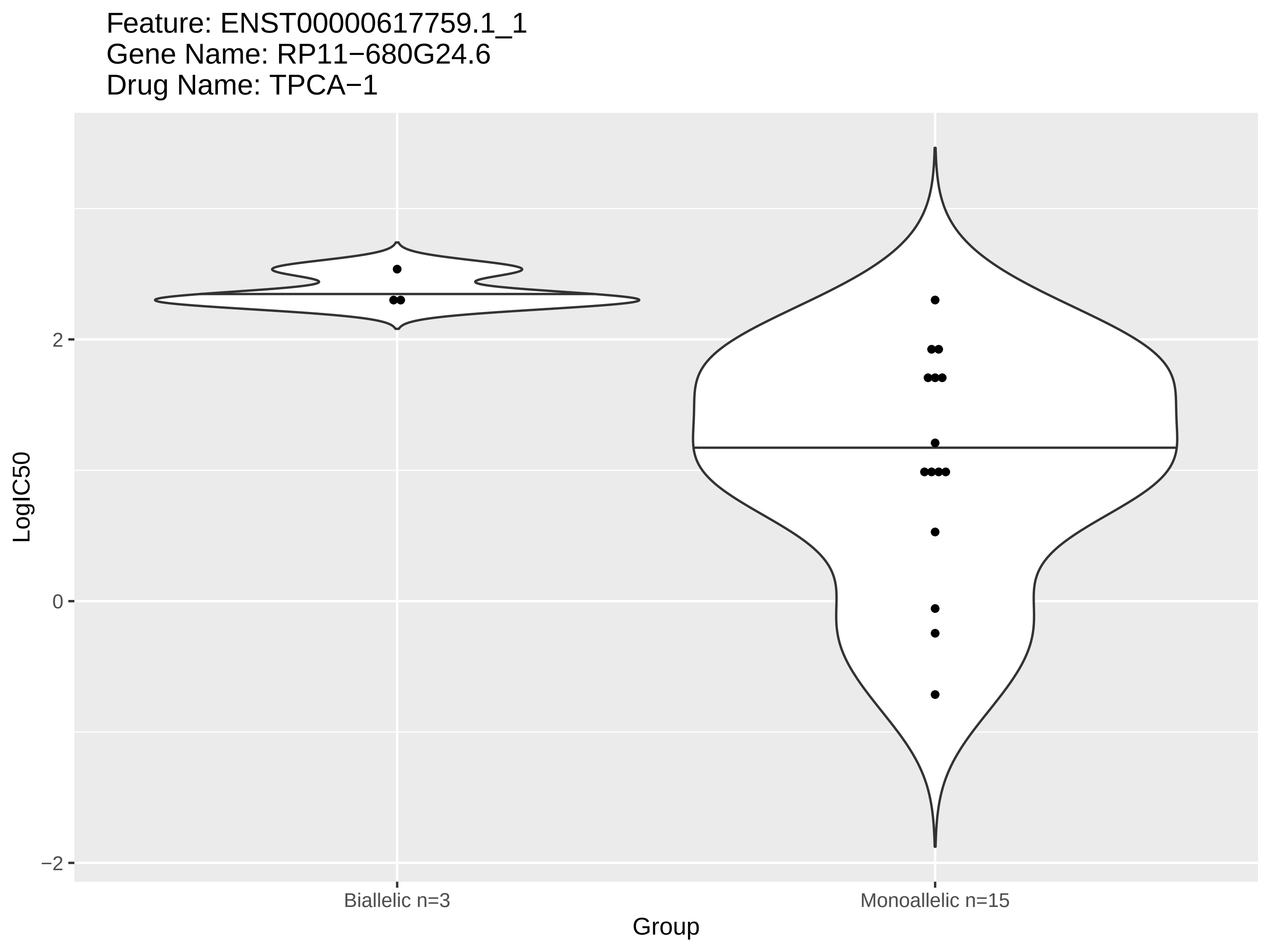

Feature: ENST00000617759.1_1
Gene Name: RP11−680G24.6
Drug Name: TPCA−1
LogIC50
2
0
−2
Biallelic n=3
Monoallelic n=15
Group

Feature: ENST00000527759.5_1
Gene Name: BCLAF1
Drug Name: cobimetinib

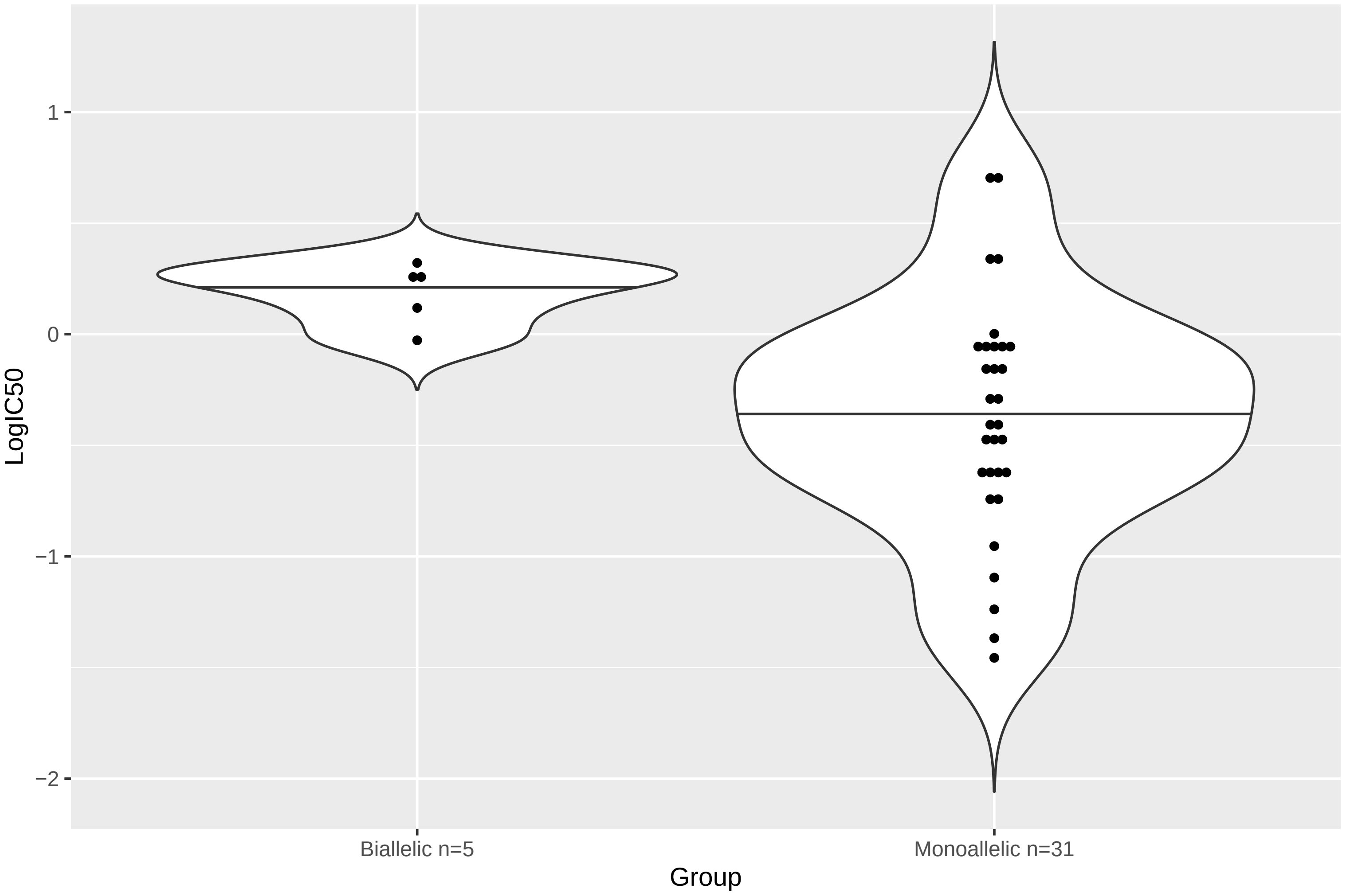

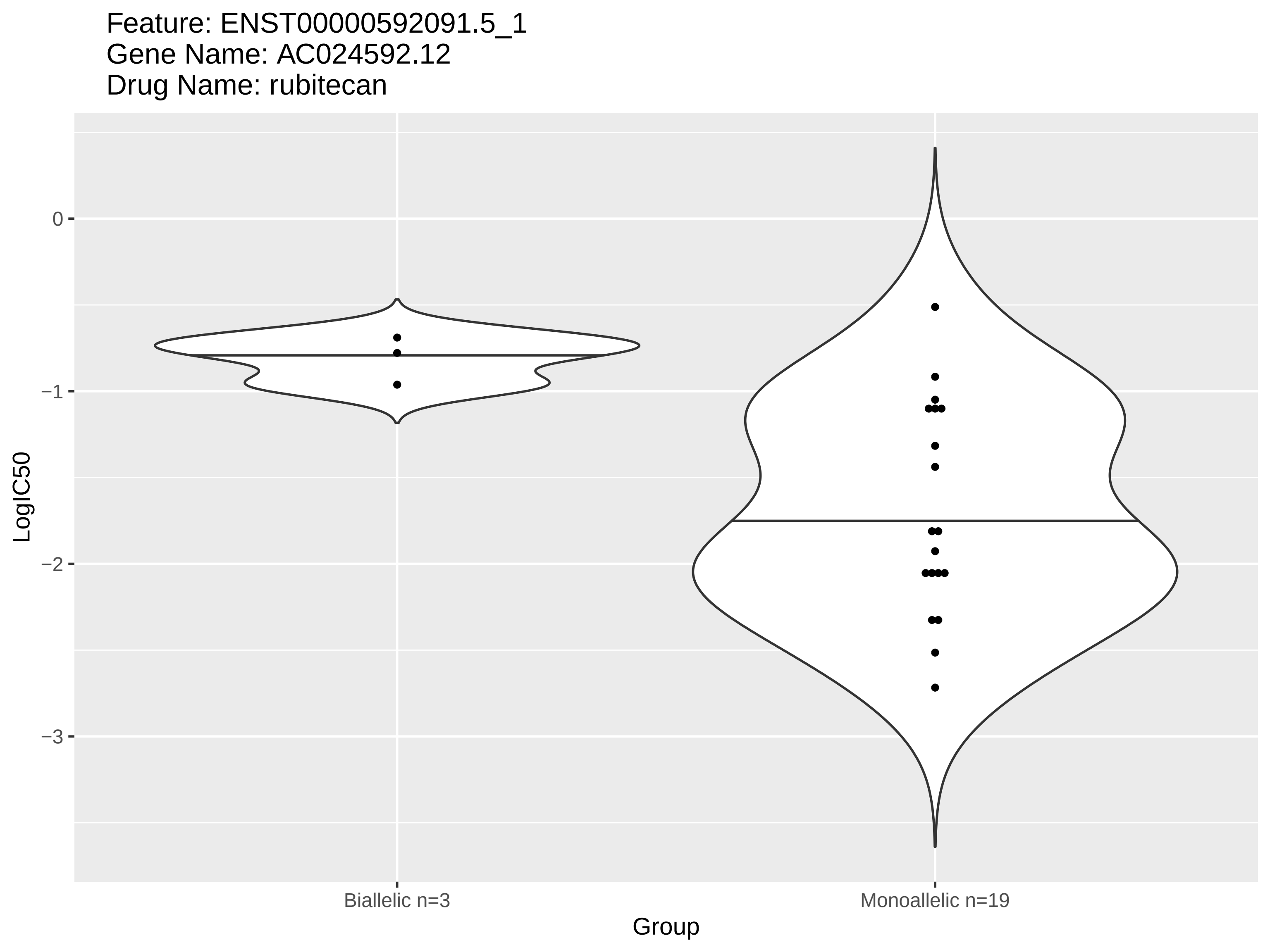
Feature: ENST00000592091.5_1
Gene Name: AC024592.12
Drug Name: rubitecan
0
−1
−2
−3
LogIC50
Biallelic n=3
Monoallelic n=19
Group

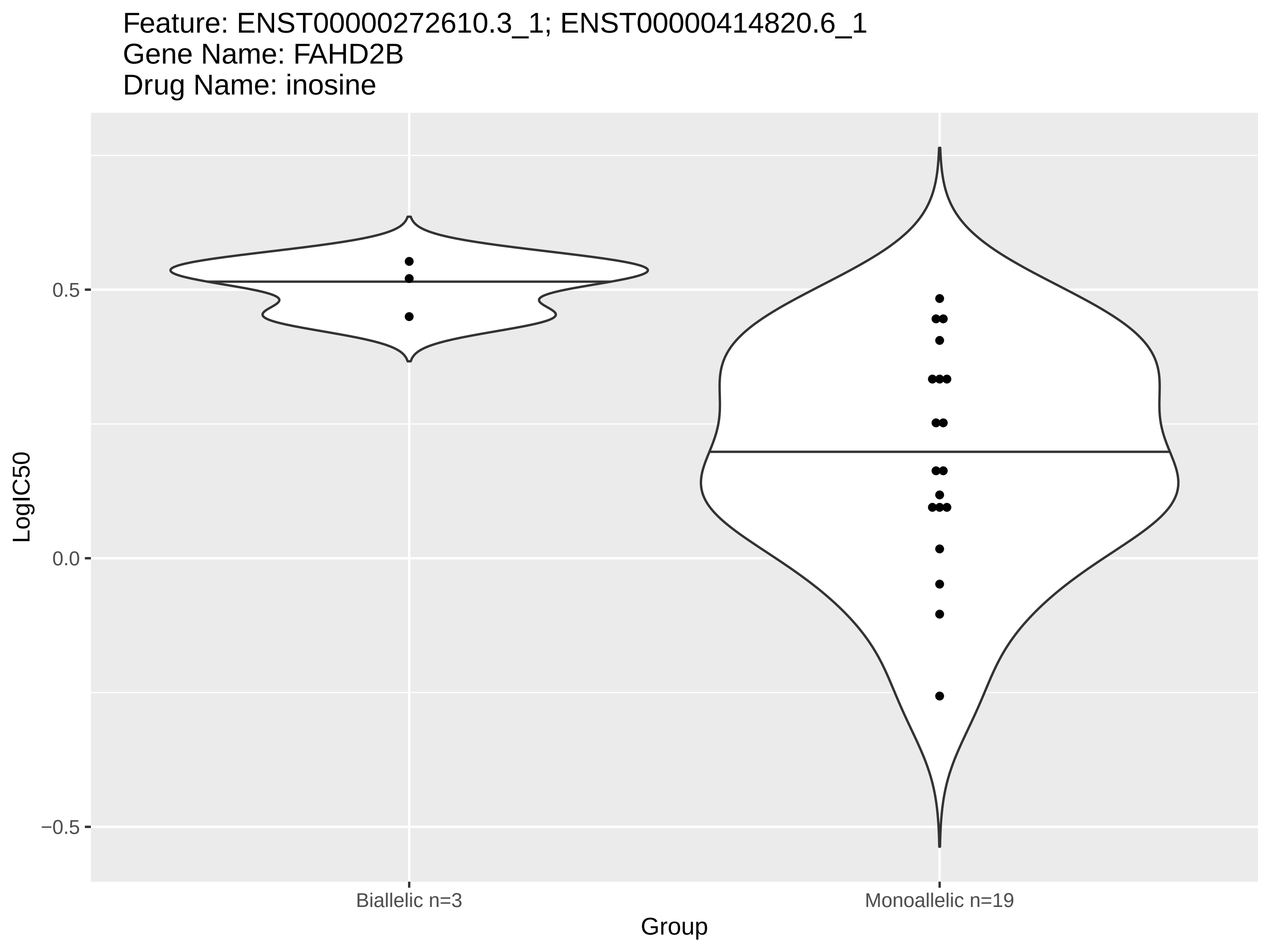
Feature: ENST00000272610.3_1; ENST00000414820.6_1
Gene Name: FAHD2B
Drug Name: inosine
LogIC50
0.5
0.0
−0.5
Biallelic n=3
Monoallelic n=19
Group

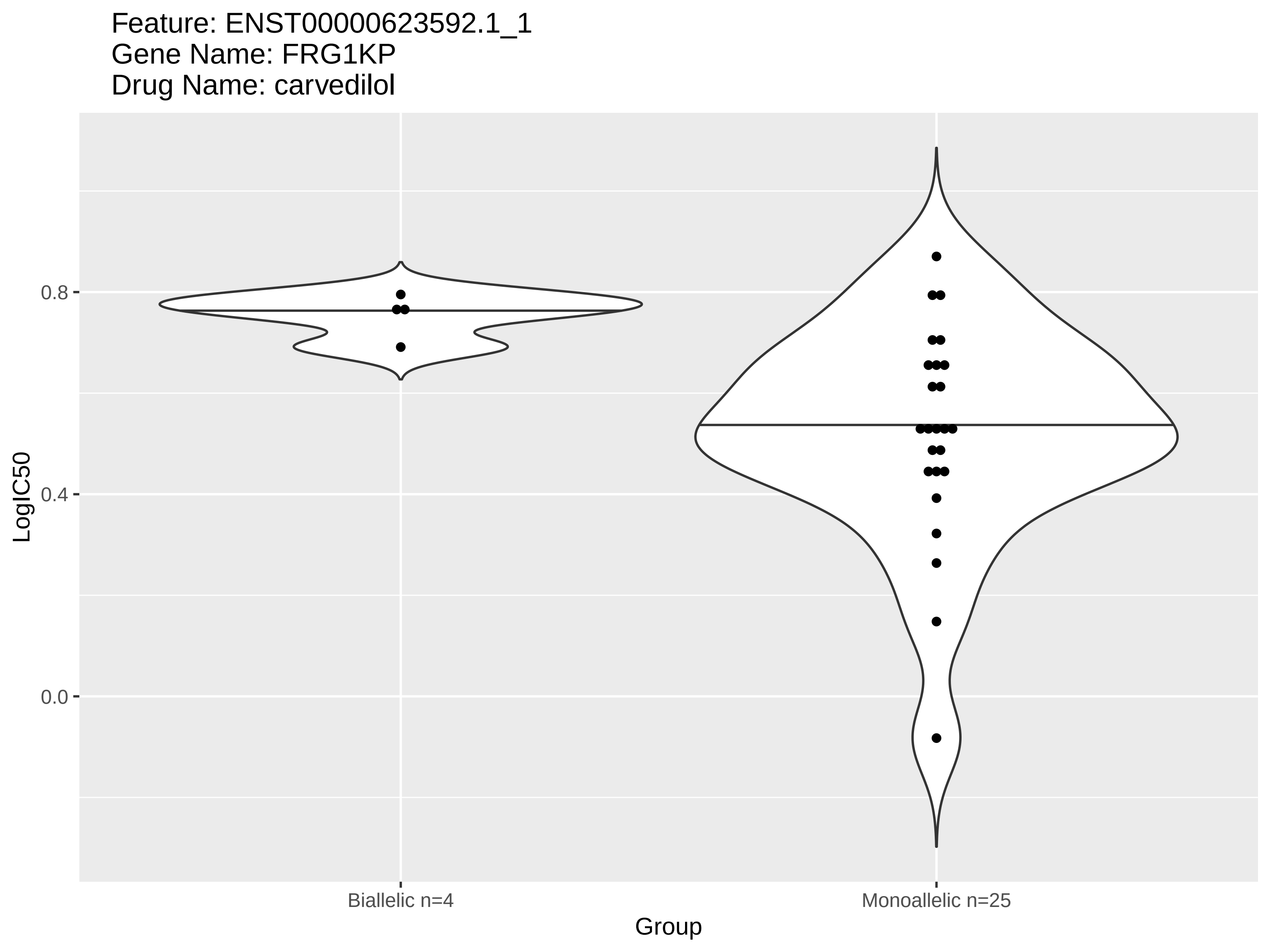

Feature: ENST00000623592.1_1
Gene Name: FRG1KP
Drug Name: carvedilol
LogIC50
0.8
0.4
0.0
Biallelic n=4
Monoallelic n=25
Group

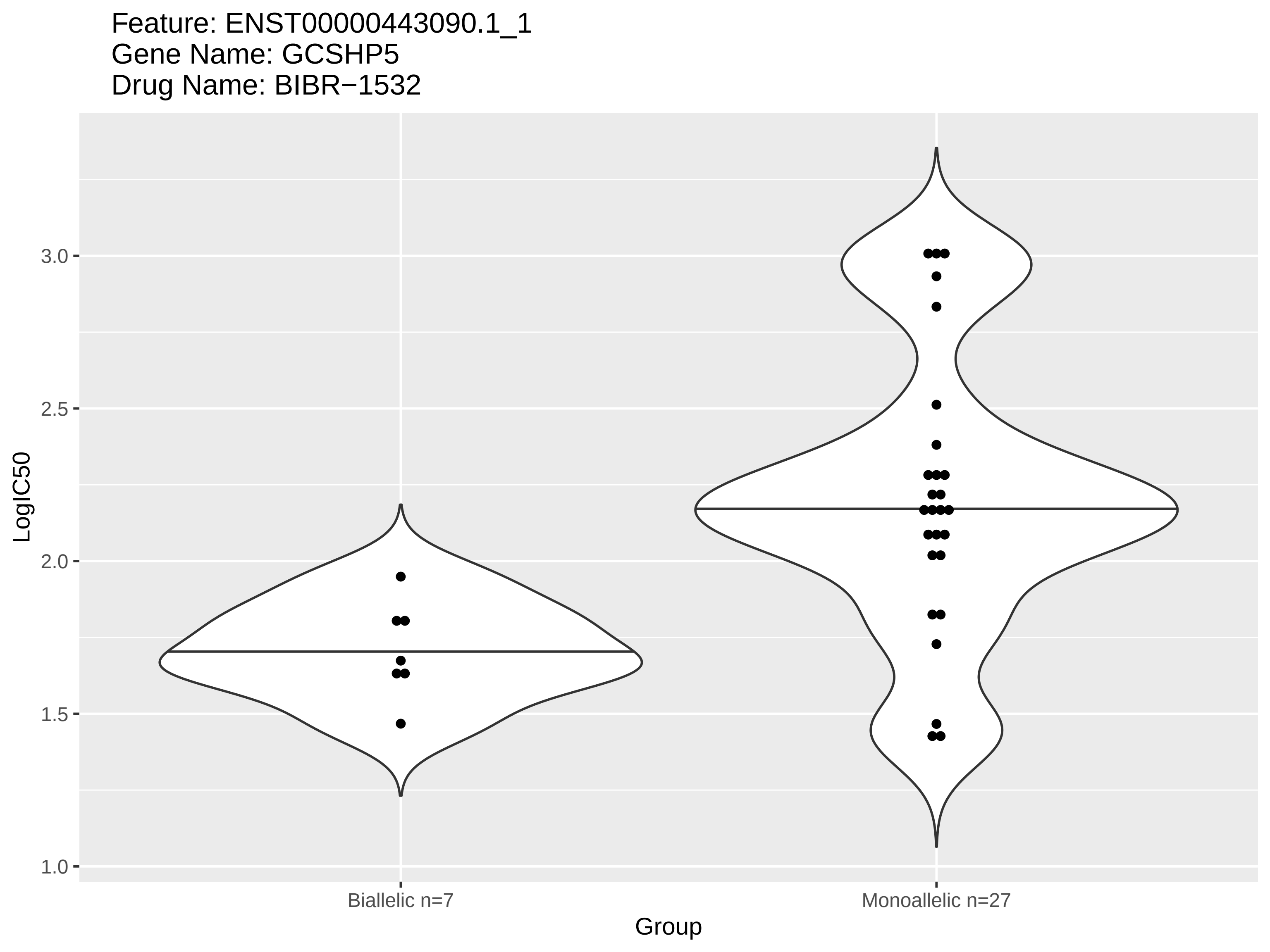
Feature: ENST00000443090.1_1
Gene Name: GCSHP5
Drug Name: BIBR−1532
LogIC50
3.0
2.5
2.0
1.5
1.0
Biallelic n=7
Monoallelic n=27
Group

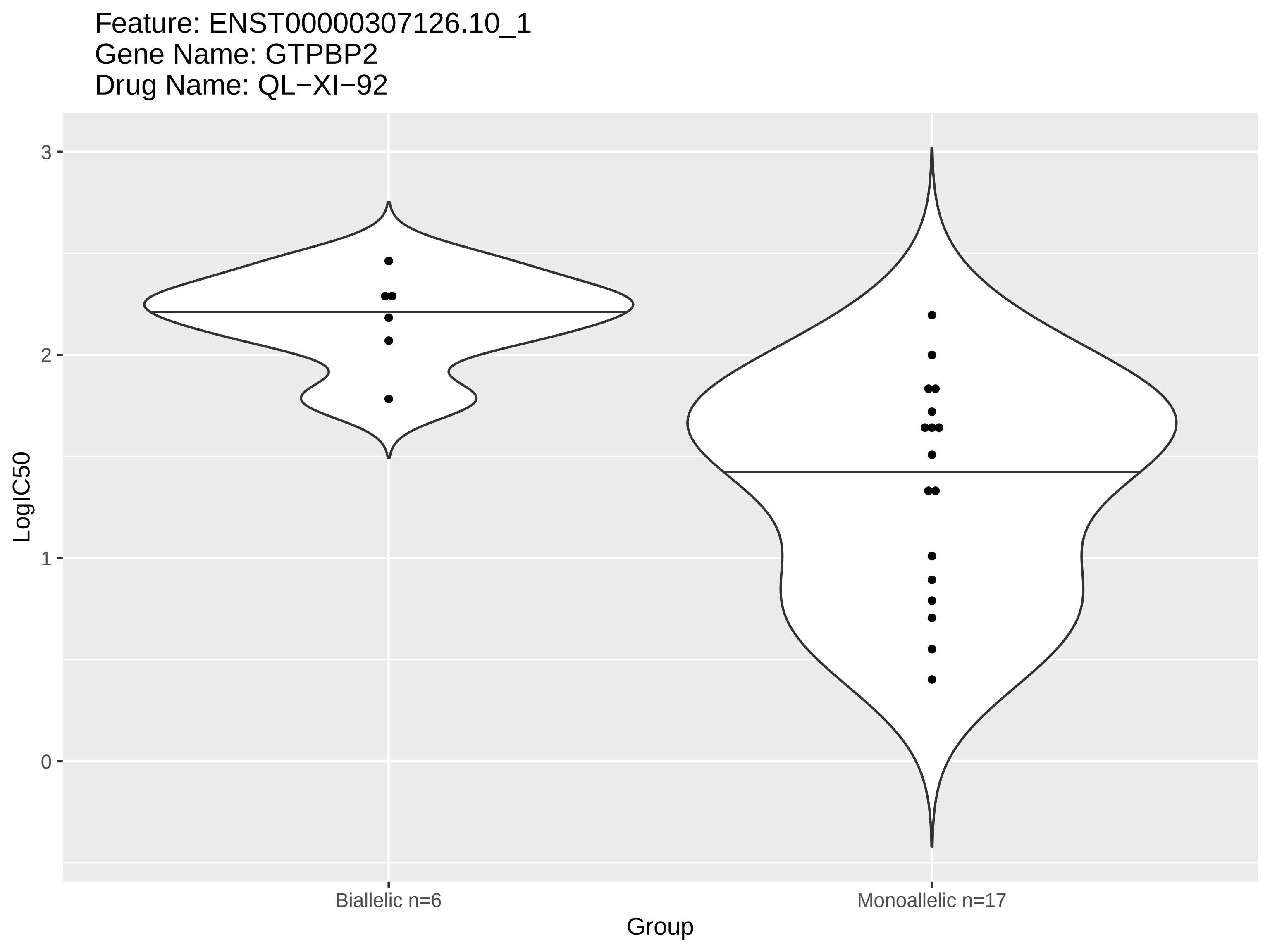
Feature: ENST00000307126.10_1
Gene Name: GTPBP2
Drug Name: QL−XI−92
LogIC50
3
2
1
0
Biallelic n=6
Monoallelic n=17
Group

Feature: ENST00000443090.1_1
Gene Name: GCSHP5
Drug Name: PD173074

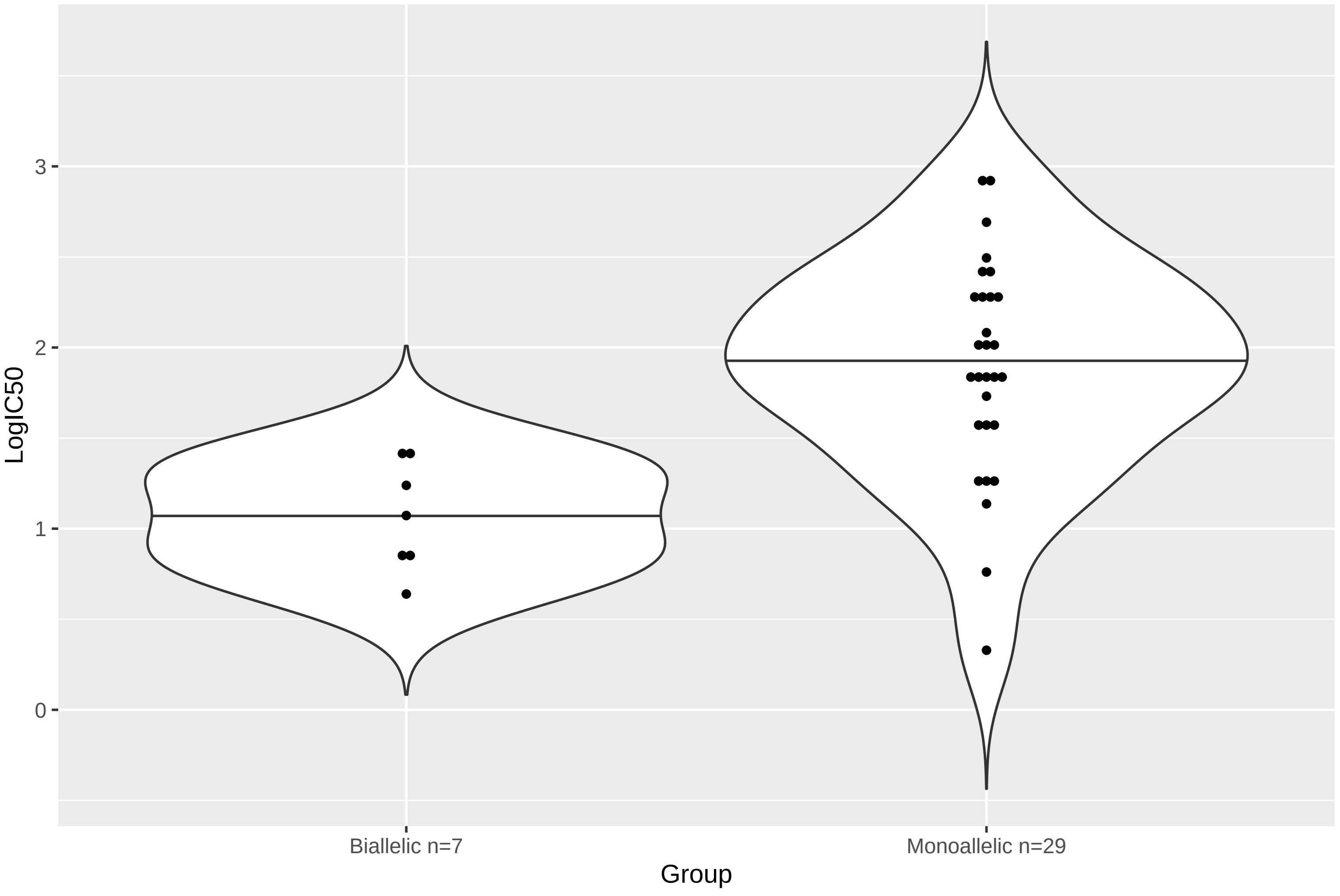

Feature: ENST00000470544.2_1
Gene Name: RPL7AP31
Drug Name: FGFR_3831

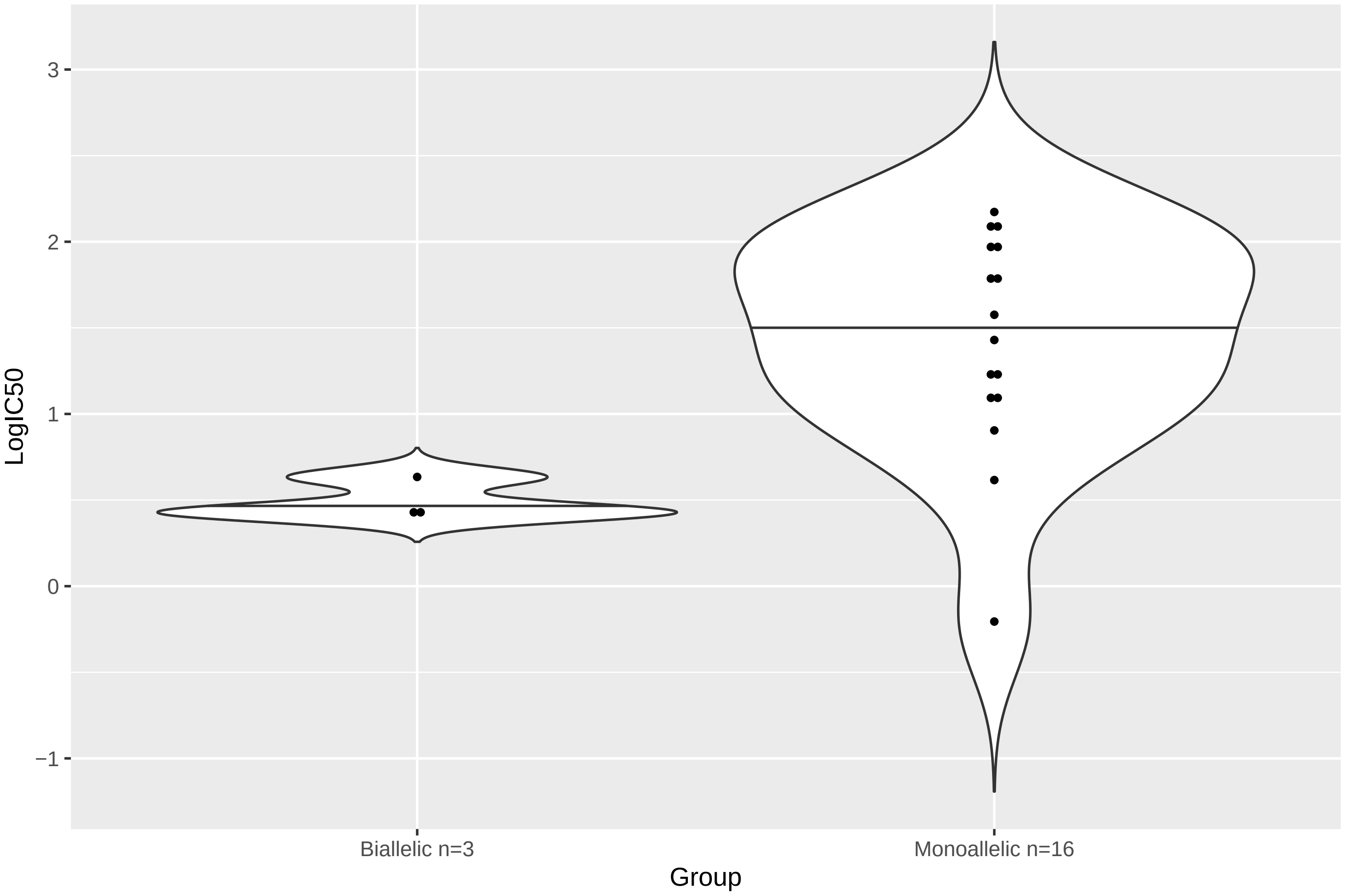

Feature: ENST00000456481.1_1
Gene Name: AC009245.3
Drug Name: LGK974

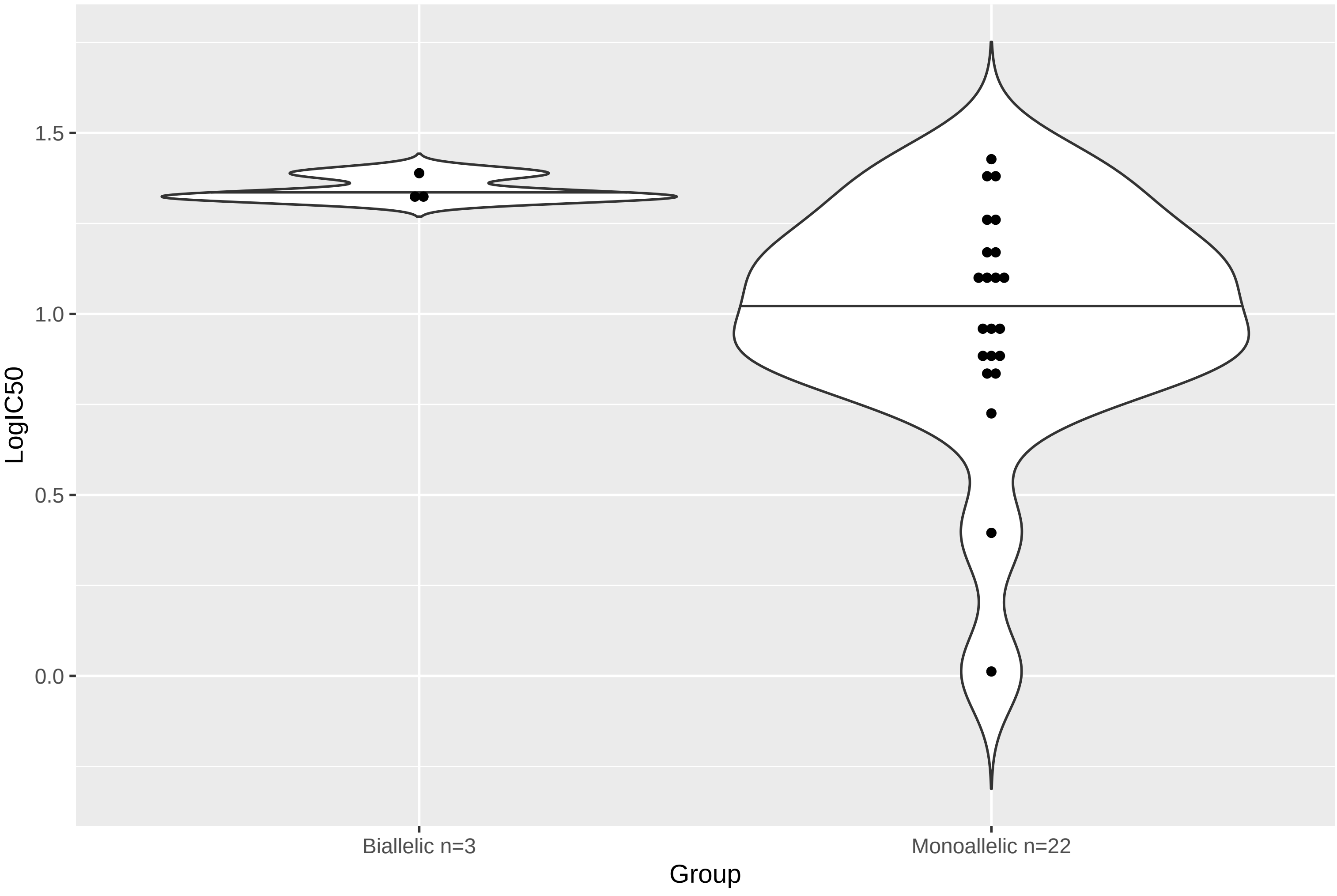

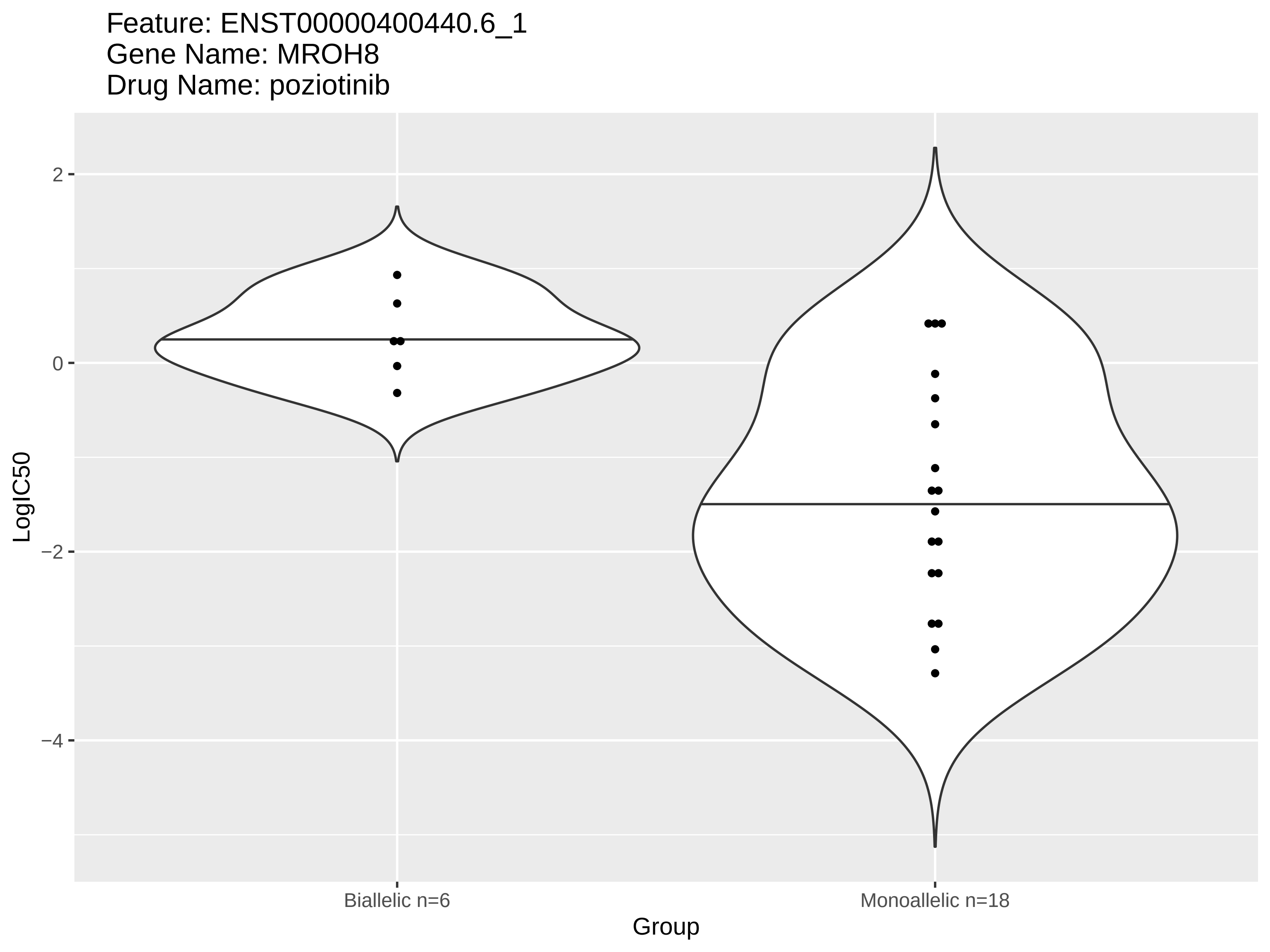
Feature: ENST00000400440.6_1
Gene Name: MROH8
Drug Name: poziotinib
LogIC50
2
0
−2
−4
Biallelic n=6
Monoallelic n=18
Group

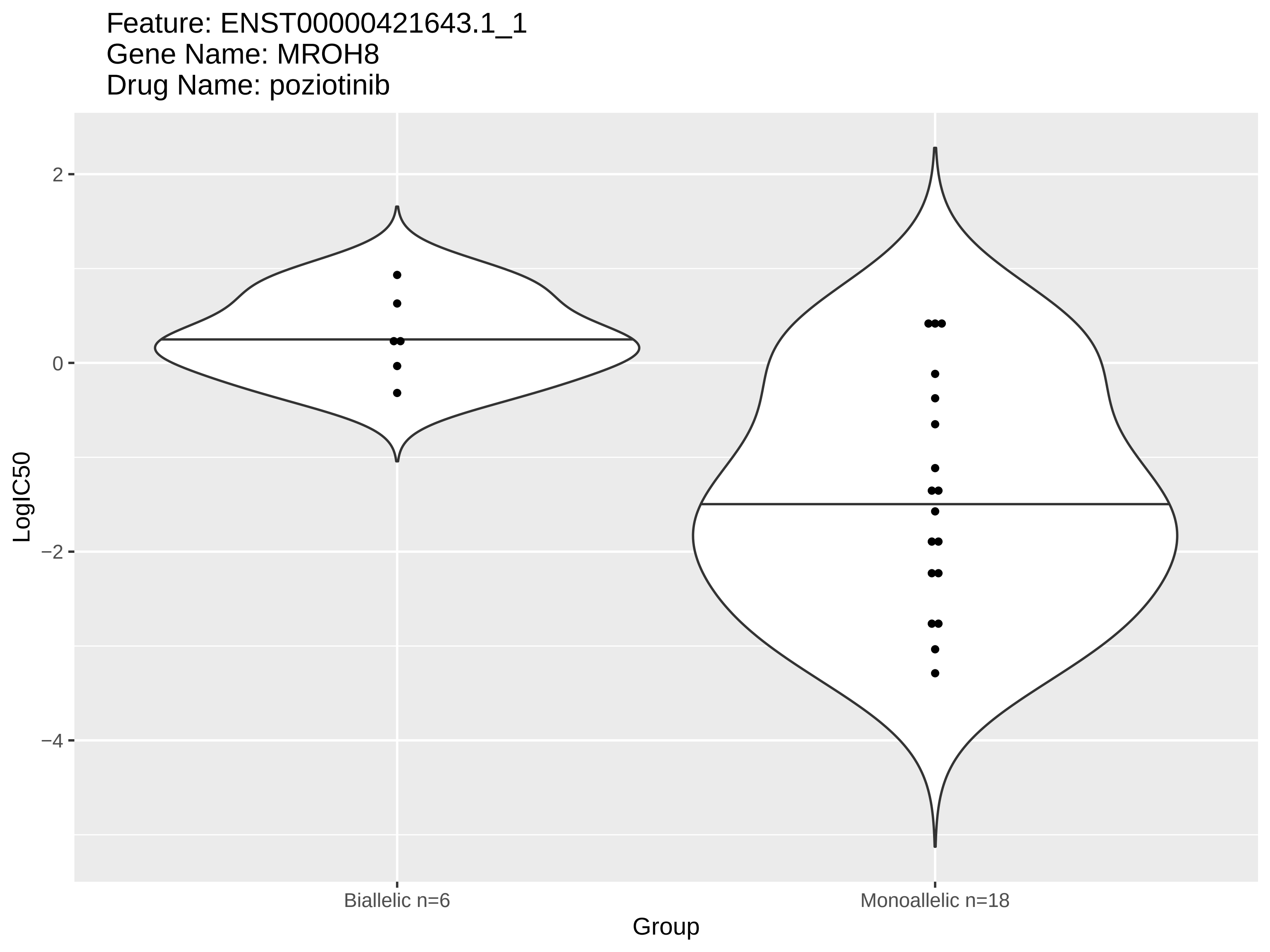
Feature: ENST00000421643.1_1
Gene Name: MROH8
Drug Name: poziotinib
LogIC50
2
0
−2
−4
Biallelic n=6
Monoallelic n=18
Group

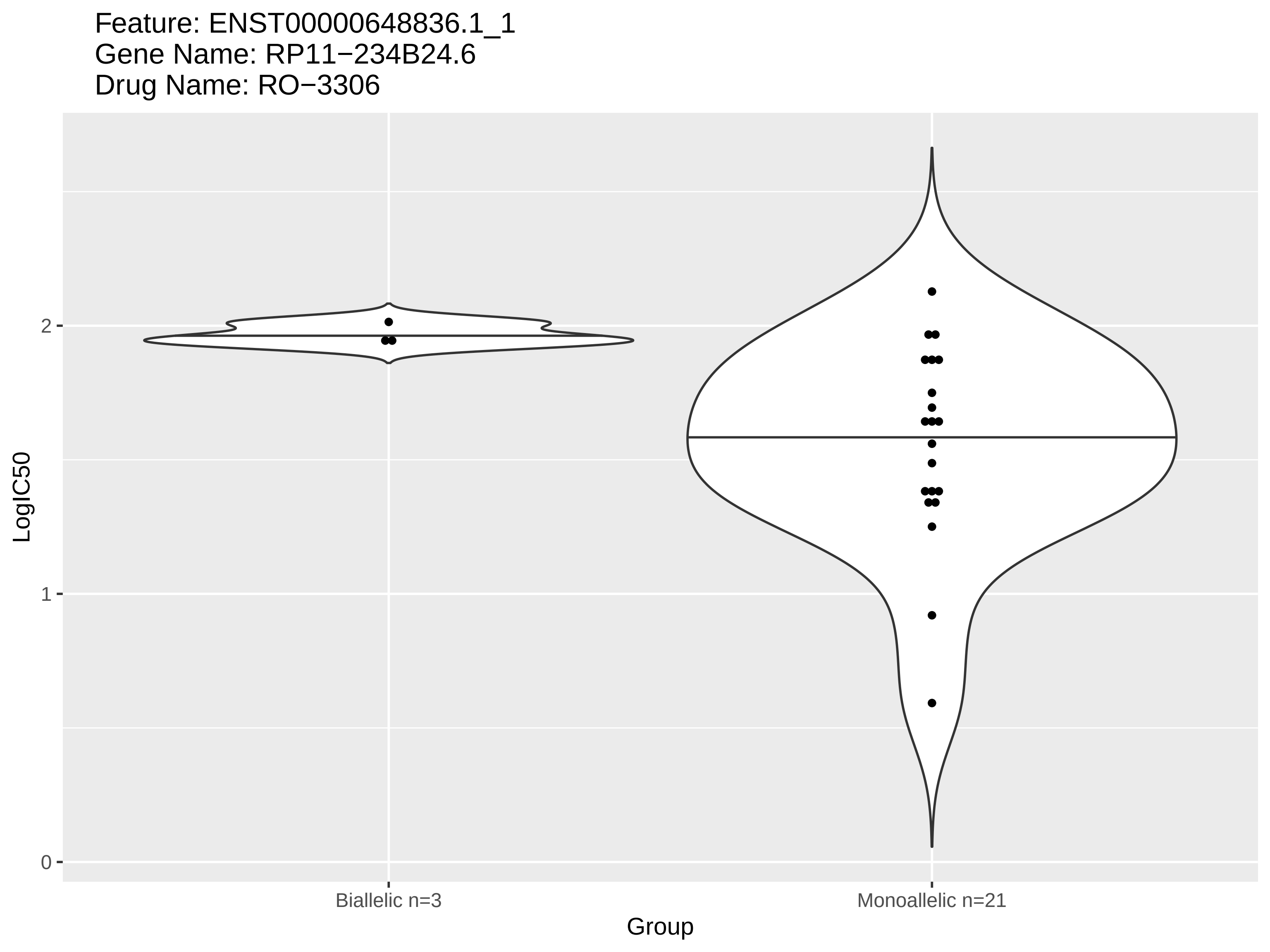
Feature: ENST00000648836.1_1
Gene Name: RP11−234B24.6
Drug Name: RO−3306
LogIC50
0
1
2
Biallelic n=3
Monoallelic n=21
Group

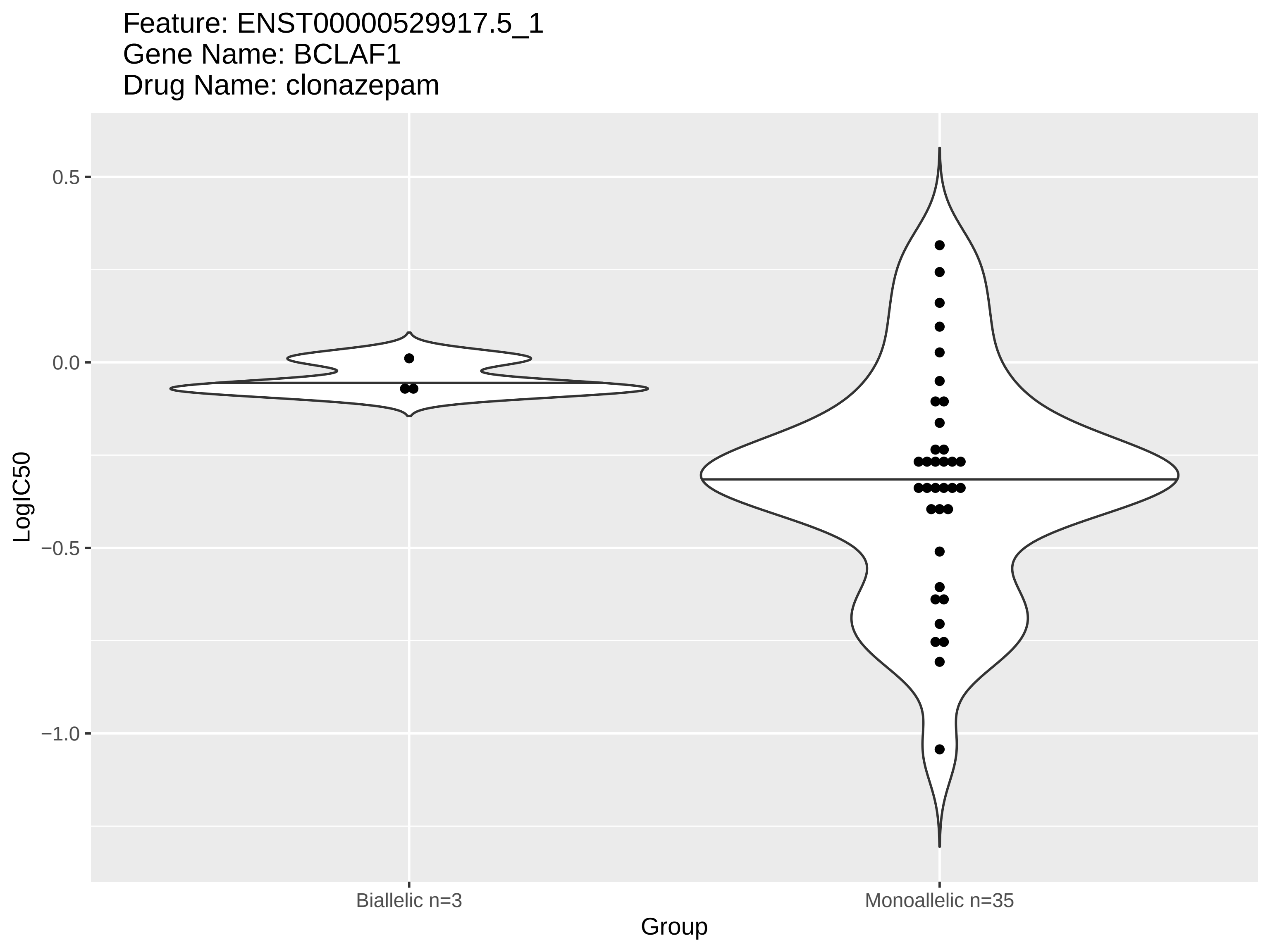
Feature: ENST00000529917.5_1
Gene Name: BCLAF1
Drug Name: clonazepam
0.5
0.0
−0.5
−1.0
LogIC50
Biallelic n=3
Monoallelic n=35
Group

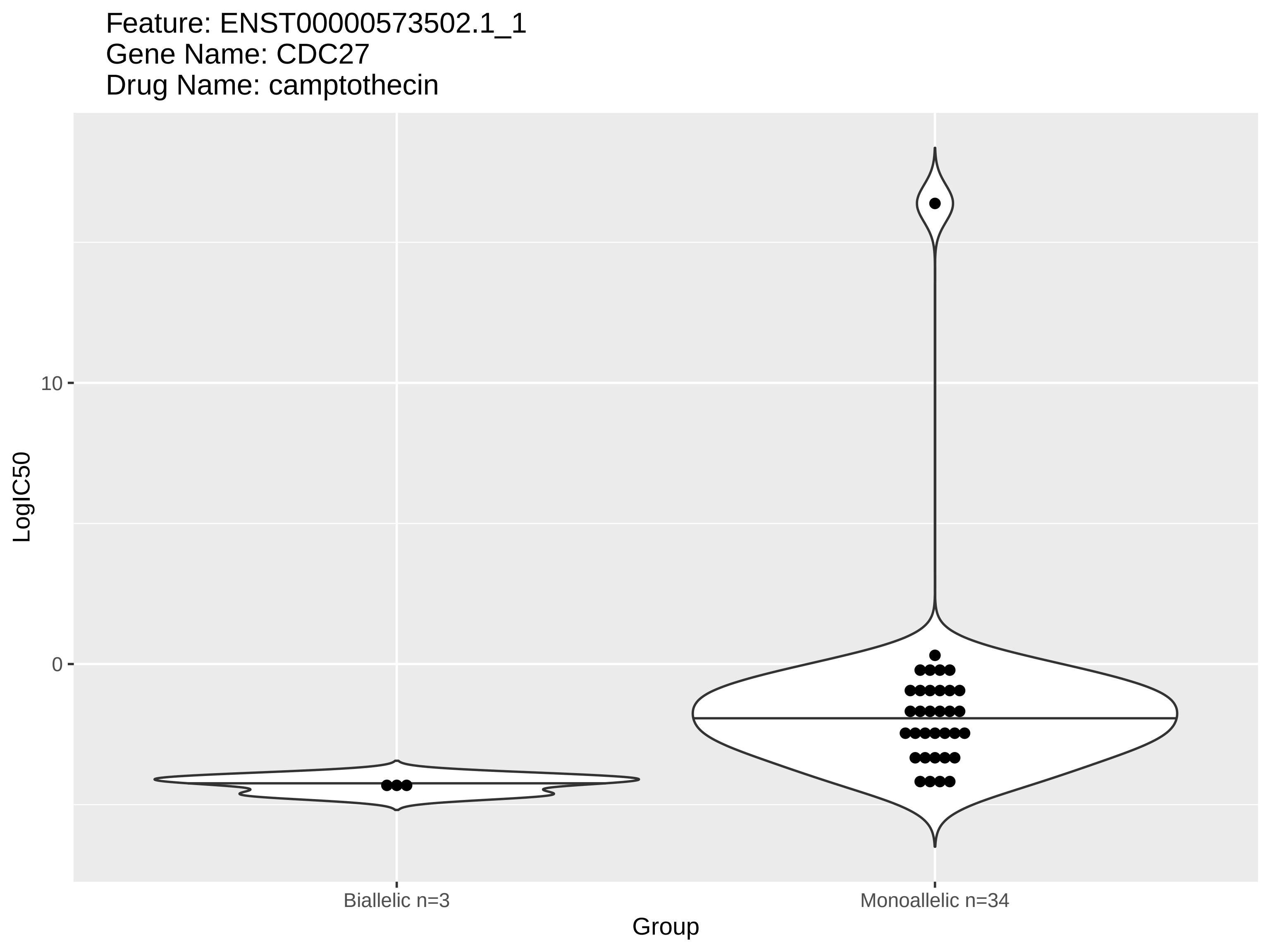

Feature: ENST00000573502.1_1
Gene Name: CDC27
Drug Name: camptothecin
10
0
LogIC50
Biallelic n=3
Monoallelic n=34
Group

Feature: ENST00000307114.11_1; ENST00000476510.5_1
Gene Name: GTPBP2
Drug Name: BMS−754807

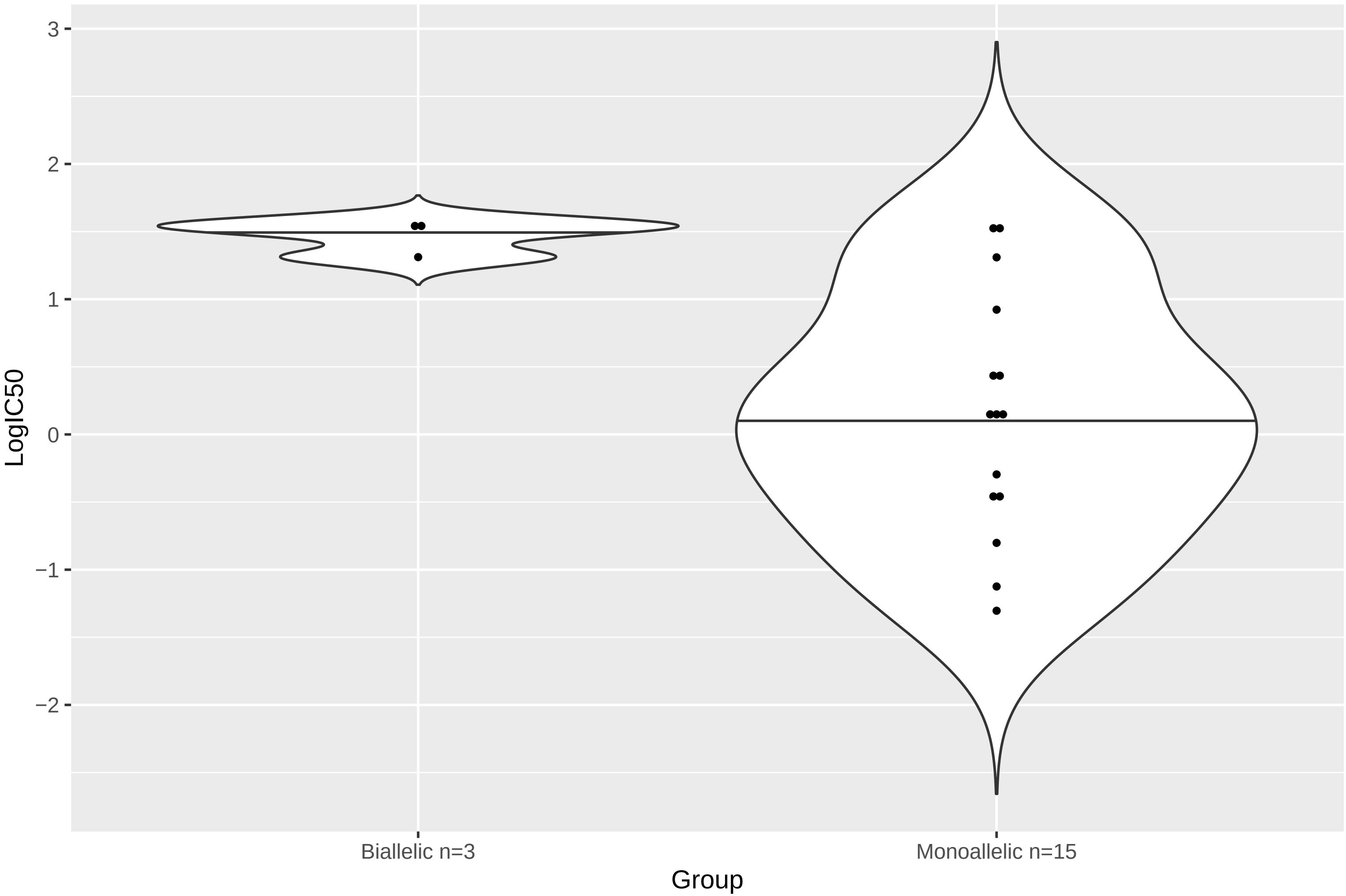

Feature: ENST00000432918.5_1
Gene Name: GTPBP2
Drug Name: BMS−754807

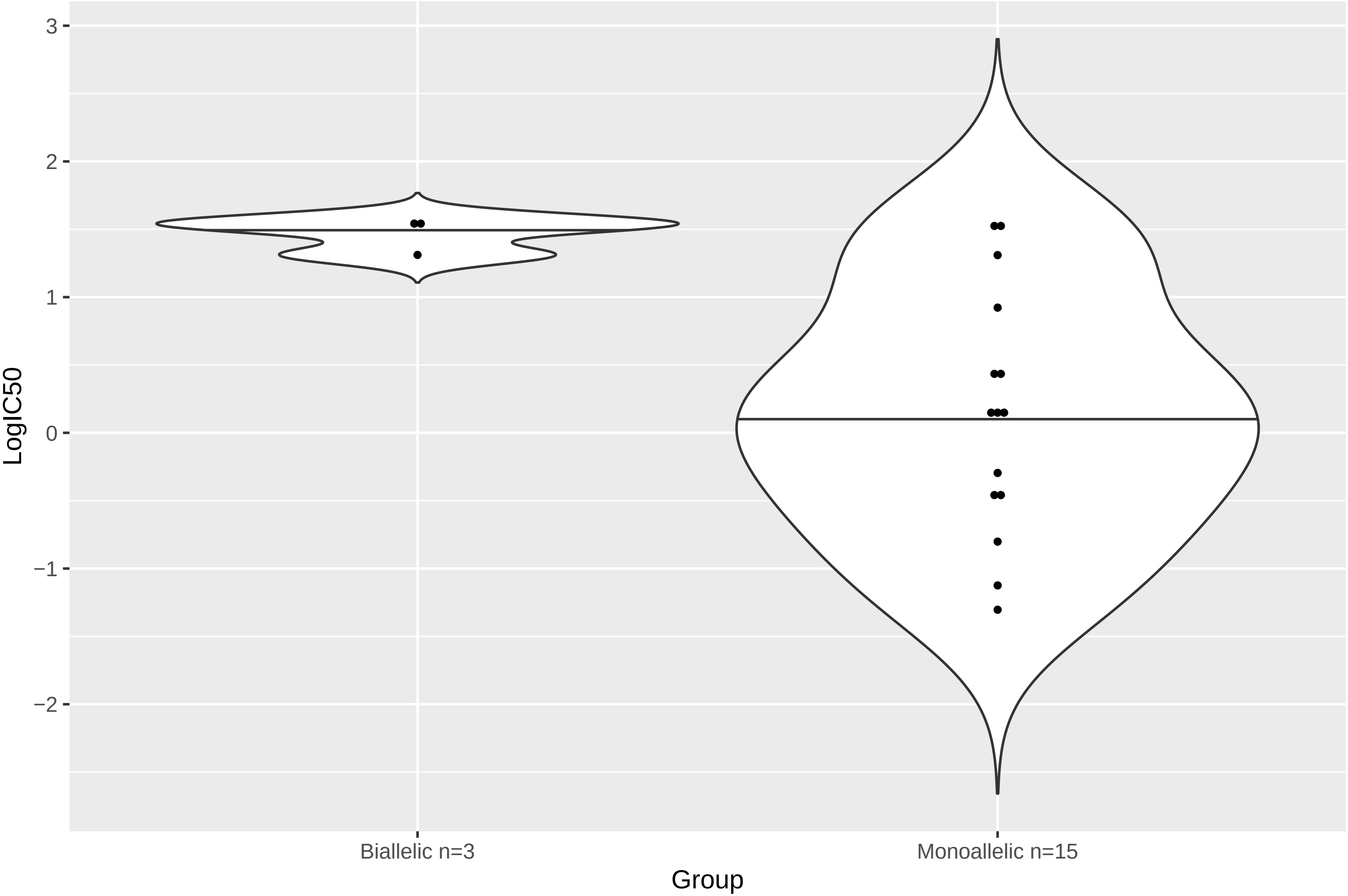

Feature: ENST00000307114.11_1; ENST00000476510.5_1
Gene Name: GTPBP2
Drug Name: C−75

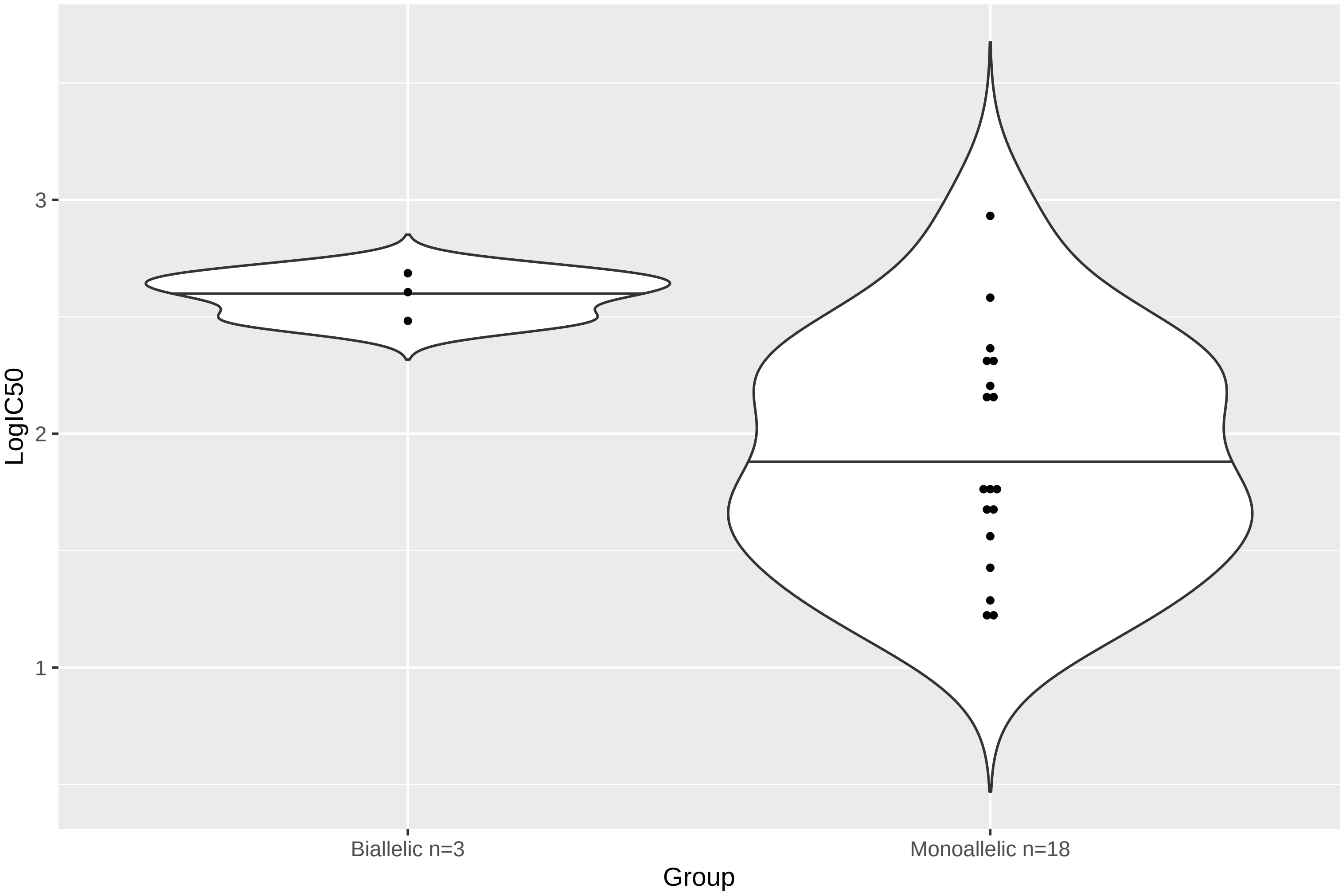

Feature: ENST00000432918.5_1
Gene Name: GTPBP2
Drug Name: C−75

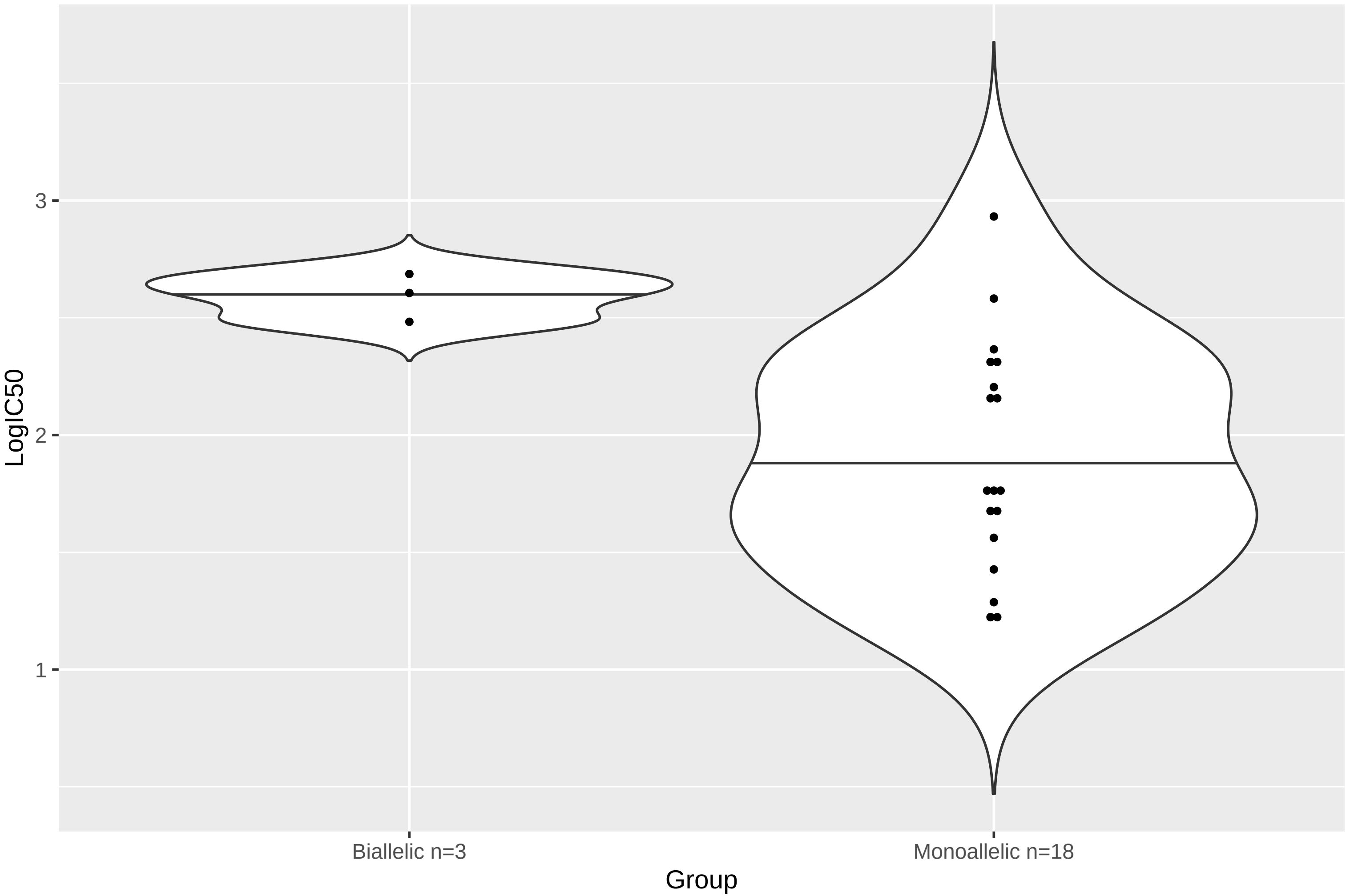

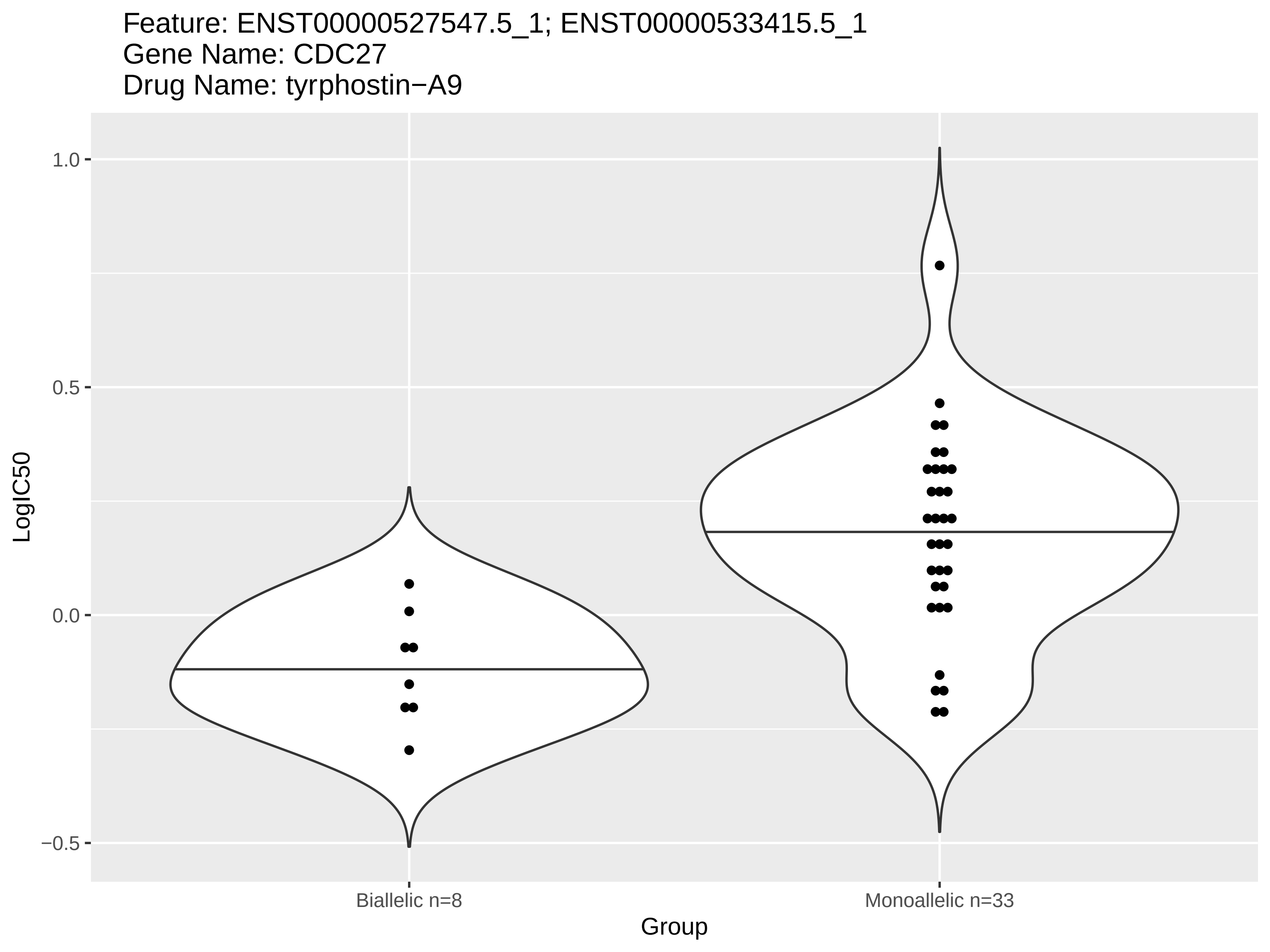

Feature: ENST00000527547.5_1; ENST00000533415.5_1
Gene Name: CDC27
Drug Name: tyrphostin−A9
LogIC50
1.0
0.5
0.0
−0.5
Biallelic n=8
Monoalleic n=33
Group

Feature: ENST00000392348.6_1; ENST00000529826.5_1; ENST00000628517.2_1
Gene Name: BCLAF1
Drug Name: cobimetinib

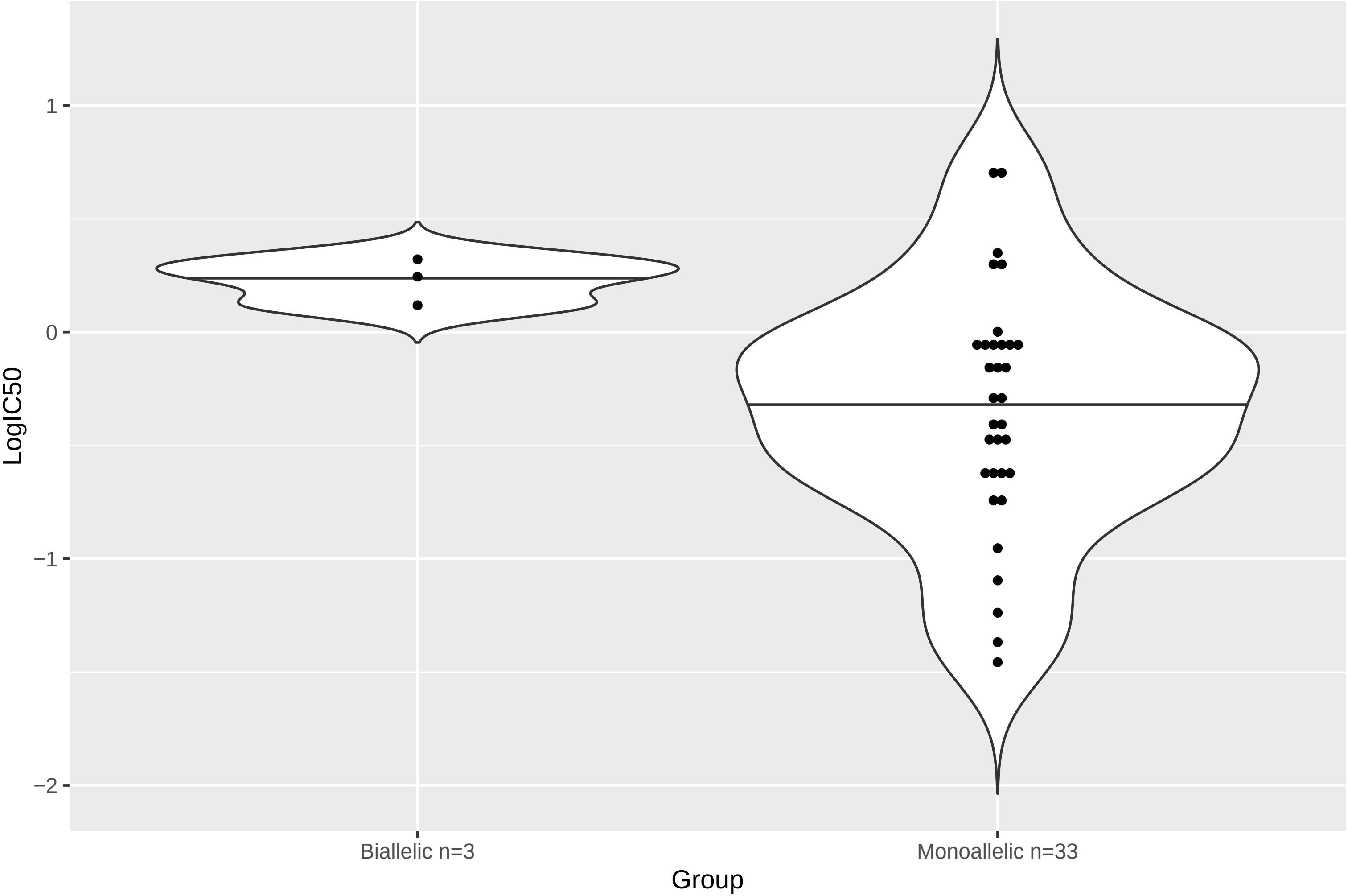

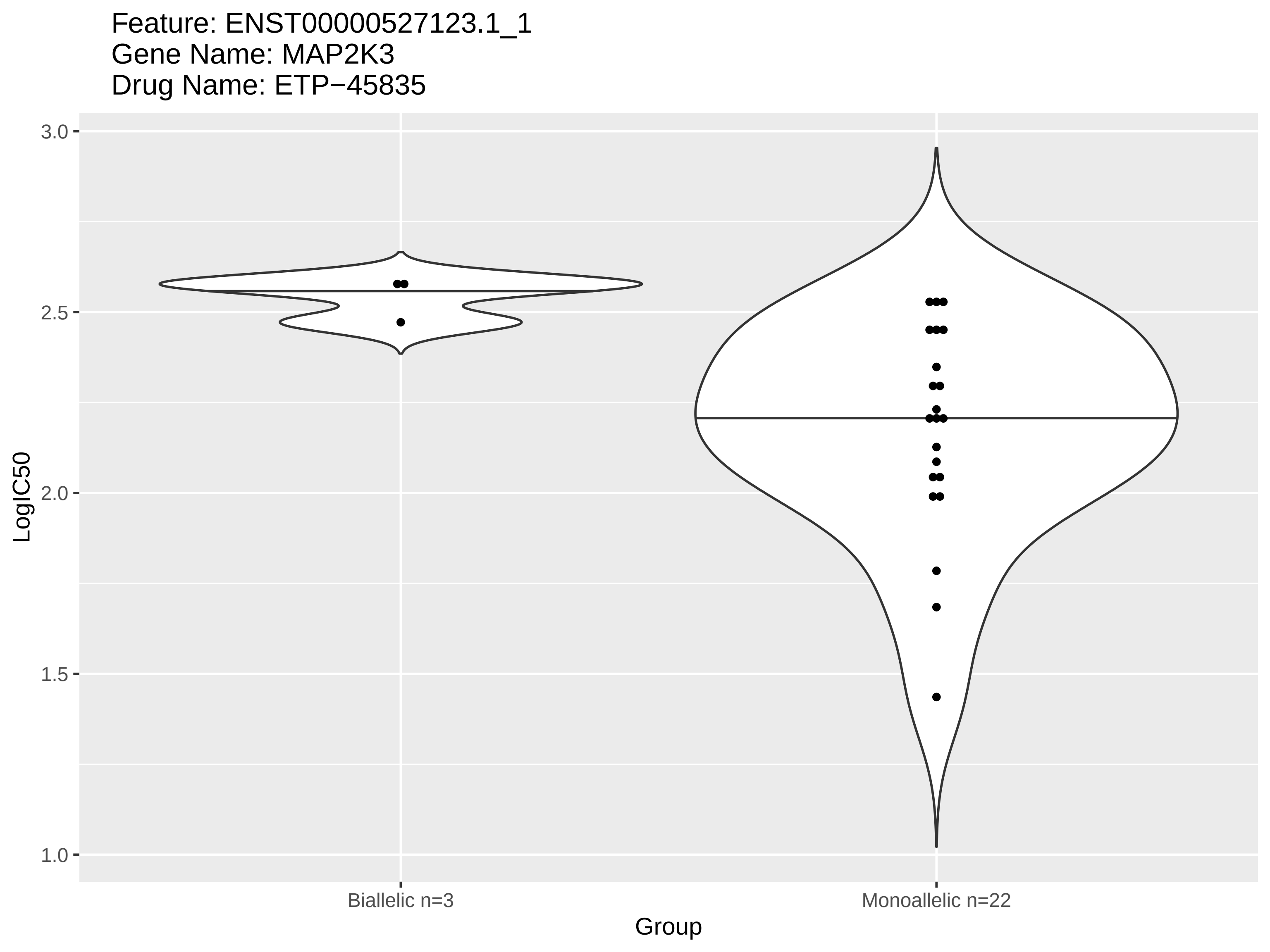

Feature: ENST00000527123.1_1
Gene Name: MAP2K3
Drug Name: ETP−45835
LogIC50
3.0
2.5
2.0
1.5
1.0
Biallelic n=3
Monoallelic n=22
Group

Feature: ENST00000573502.1_1
Gene Name: CDC27
Drug Name: NSC−697923

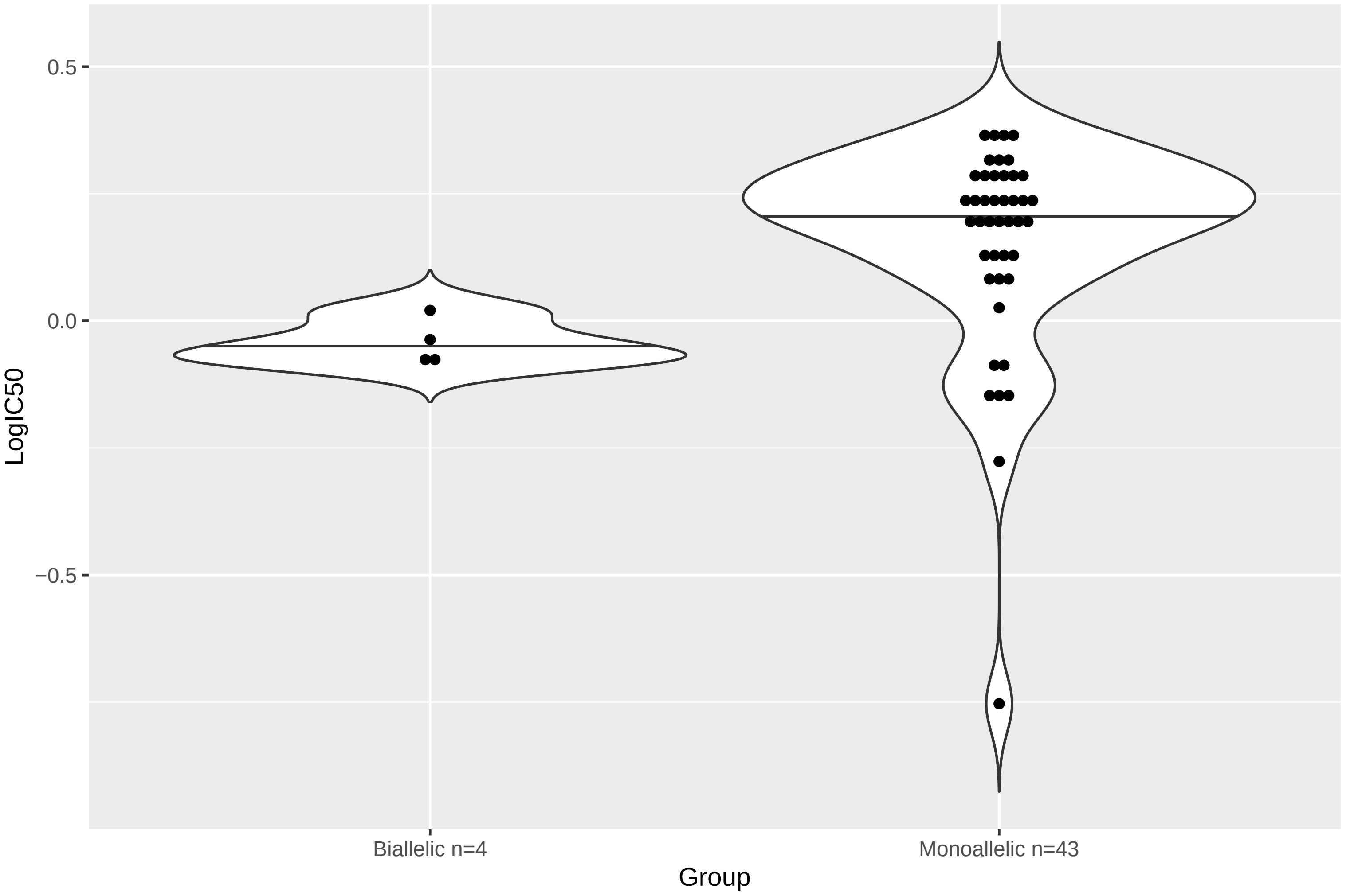

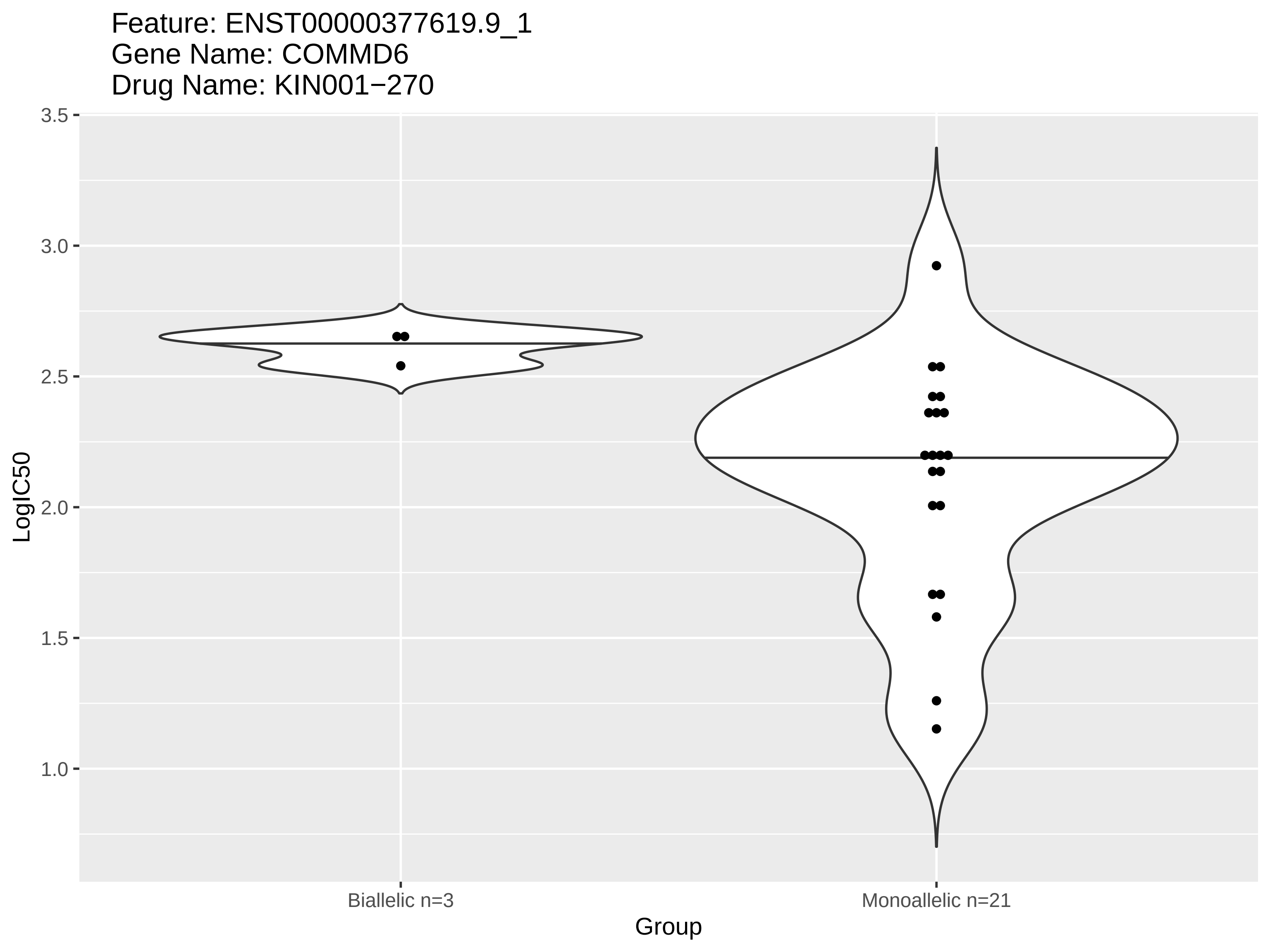
Feature: ENST00000377619.9_1
Gene Name: COMMD6
Drug Name: KIN001−270
3.5
3.0
2.5
2.0
1.5
1.0
LogIC50
Biallelic n=3
Monoallelic n=21
Group

Feature: ENST00000470638.3_1
Gene Name: PRIM2
Drug Name: dinaciclib

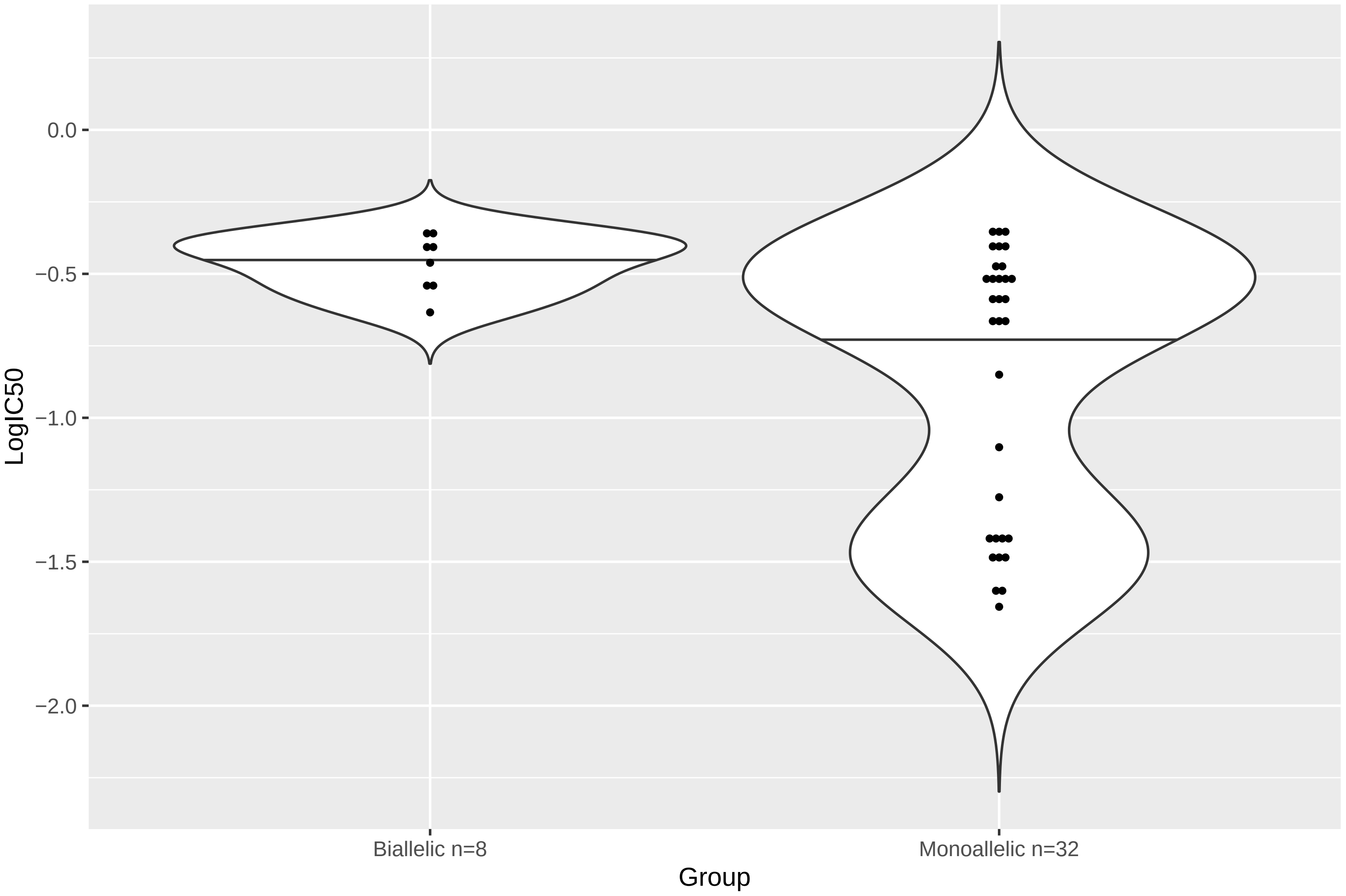

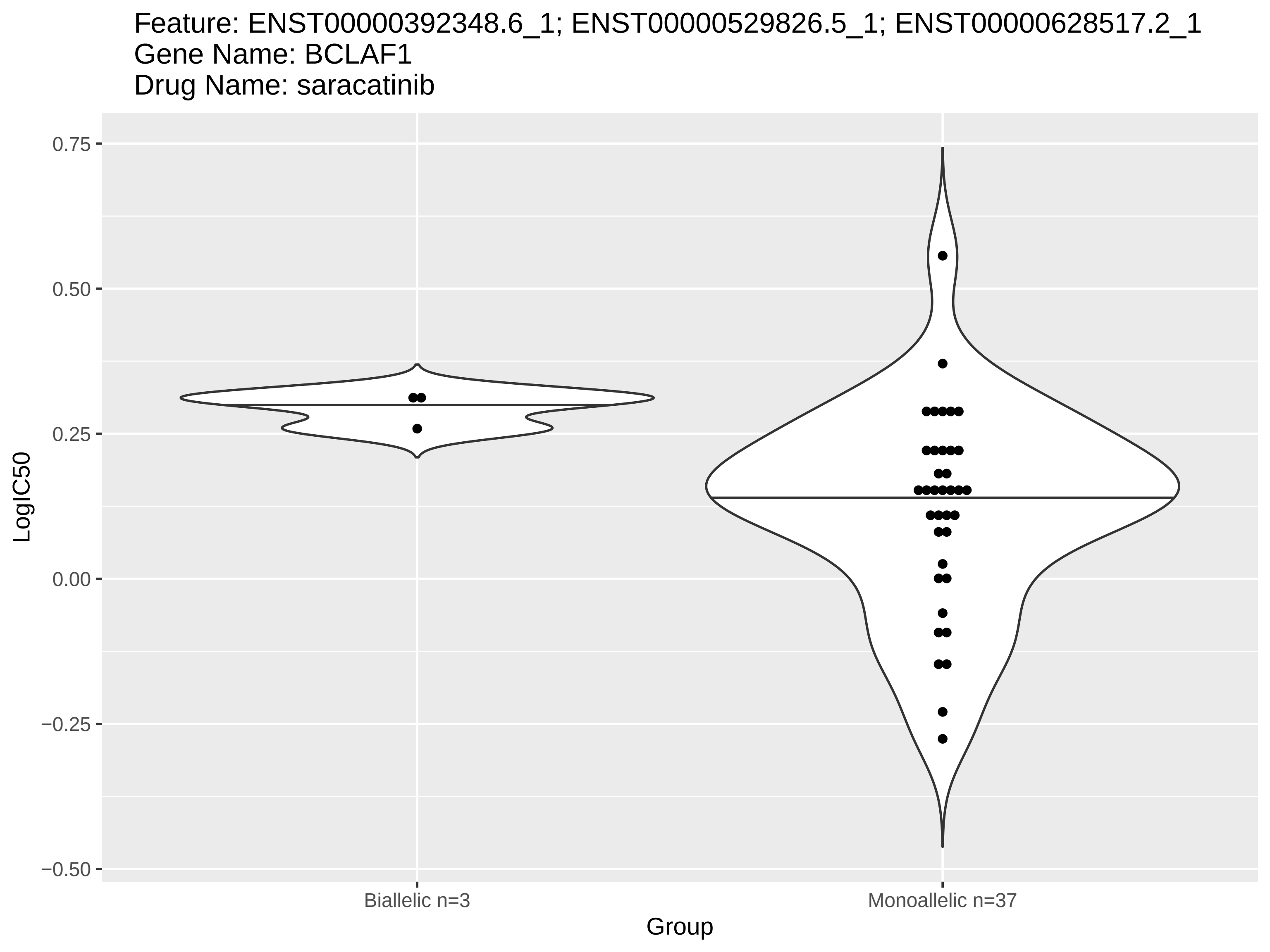

Feature: ENST00000392348.6_1; ENST00000529826.5_1; ENST00000628517.2_1
Gene Name: BCLAF1
Drug Name: saracatinib
LogIC50
0.75
0.50
0.25
0.00
−0.25
−0.50
Biallelic n=3
Monoallelic n=37
Group

Feature: ENST00000269397.9_1
Gene Name: CBX4
Drug Name: AZD3463

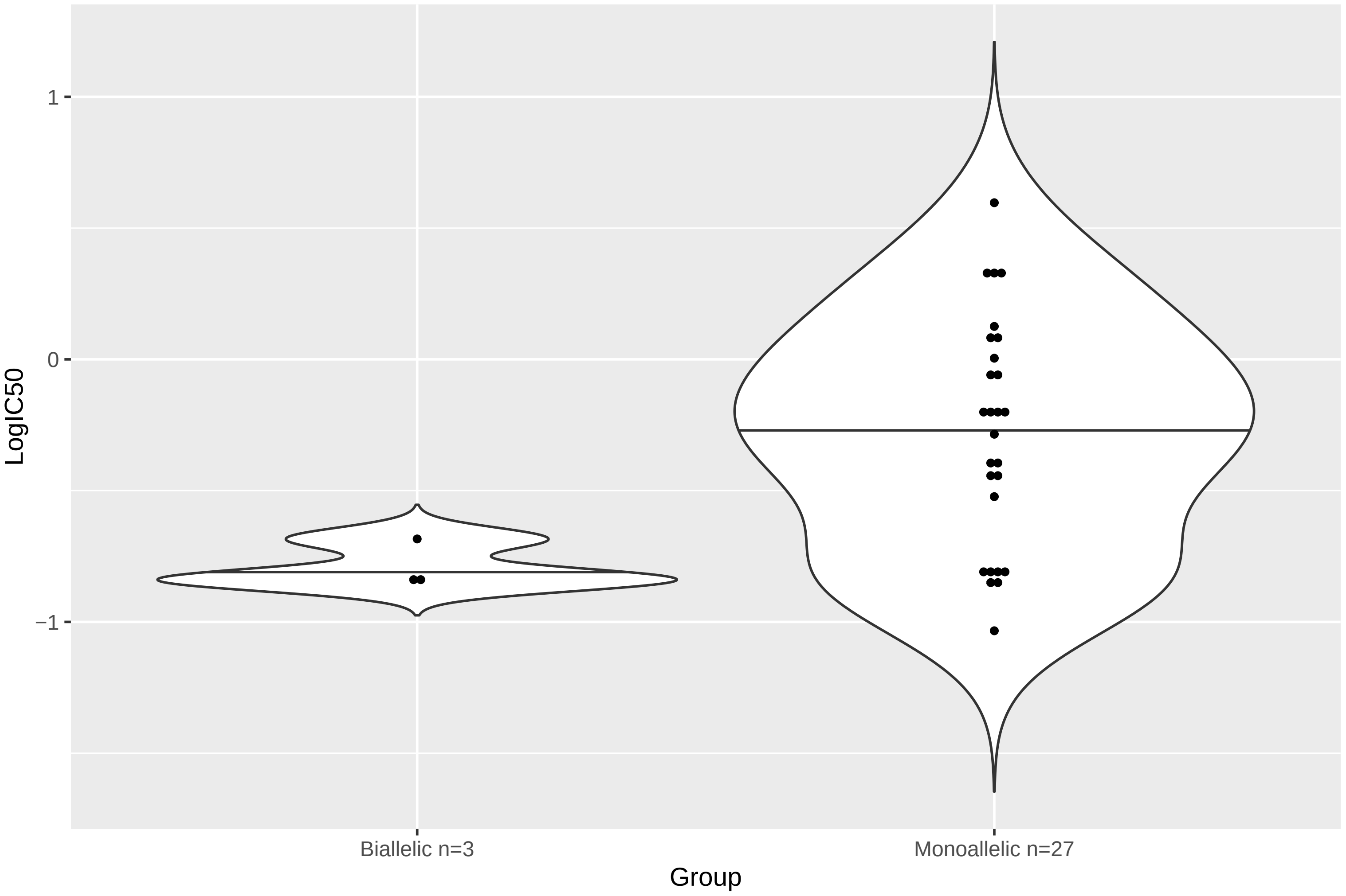

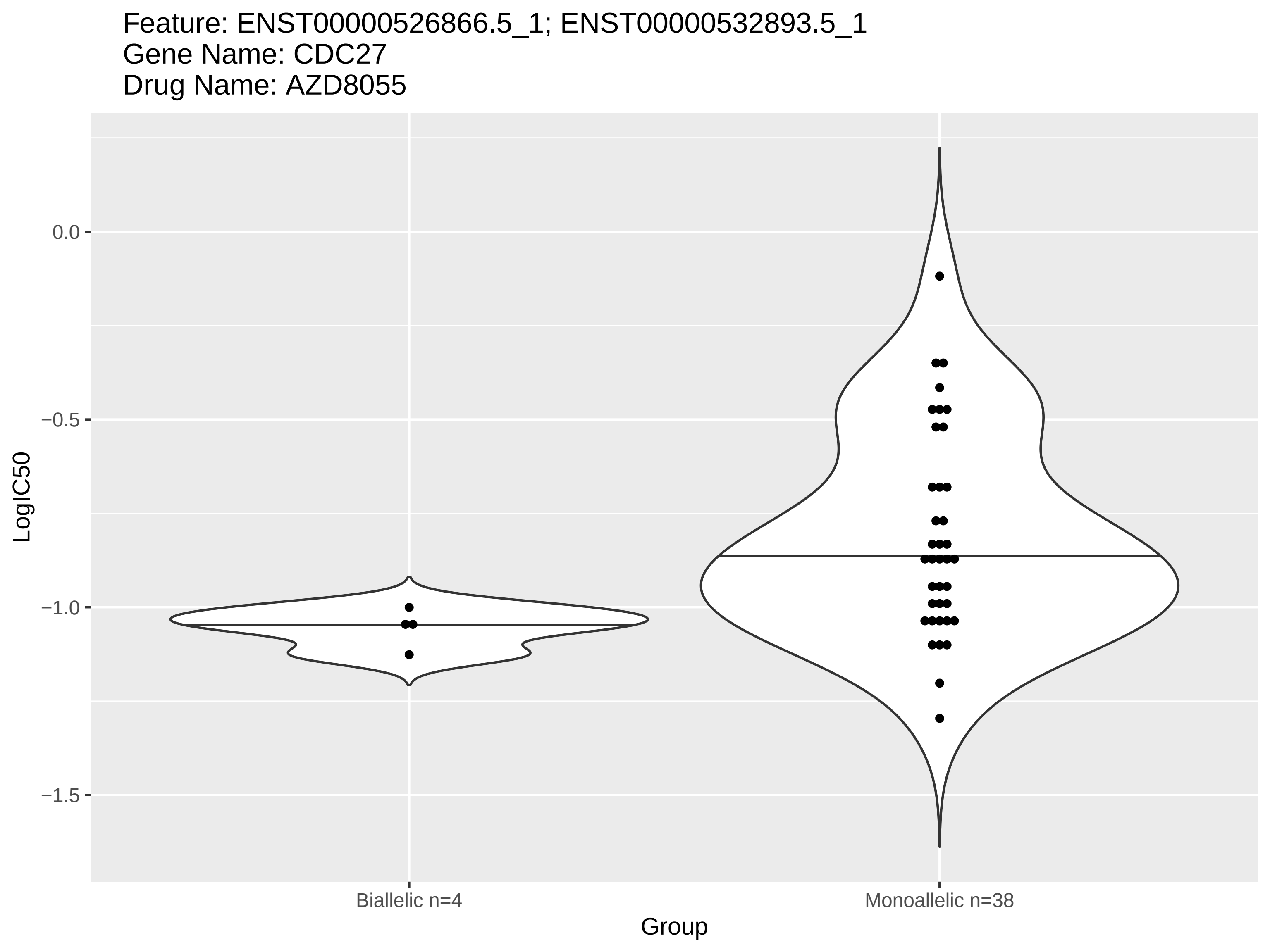

Feature: ENST00000526866.5_1; ENST00000532893.5_1
Gene Name: CDC27
Drug Name: AZD8055
LogIC50
0.0
−0.5
−1.0
−1.5
Biallelic n=4
Monoallelic n=38
Group

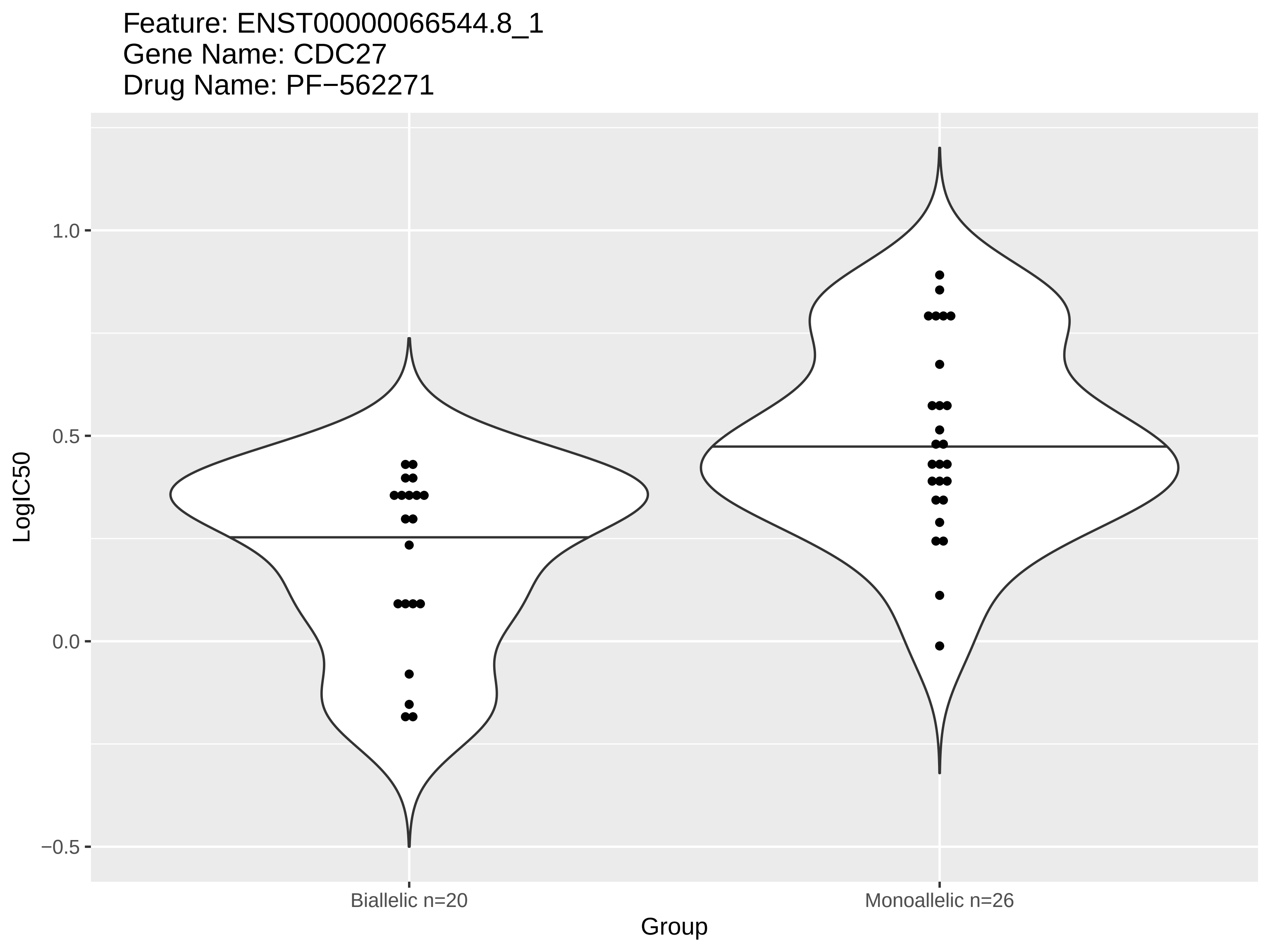

Feature: ENST00000066544.8_1
Gene Name: CDC27
Drug Name: PF−562271
LogIC50
1.0
0.5
0.0
−0.5
Biallelic n=20
Monoallelic n=26
Group

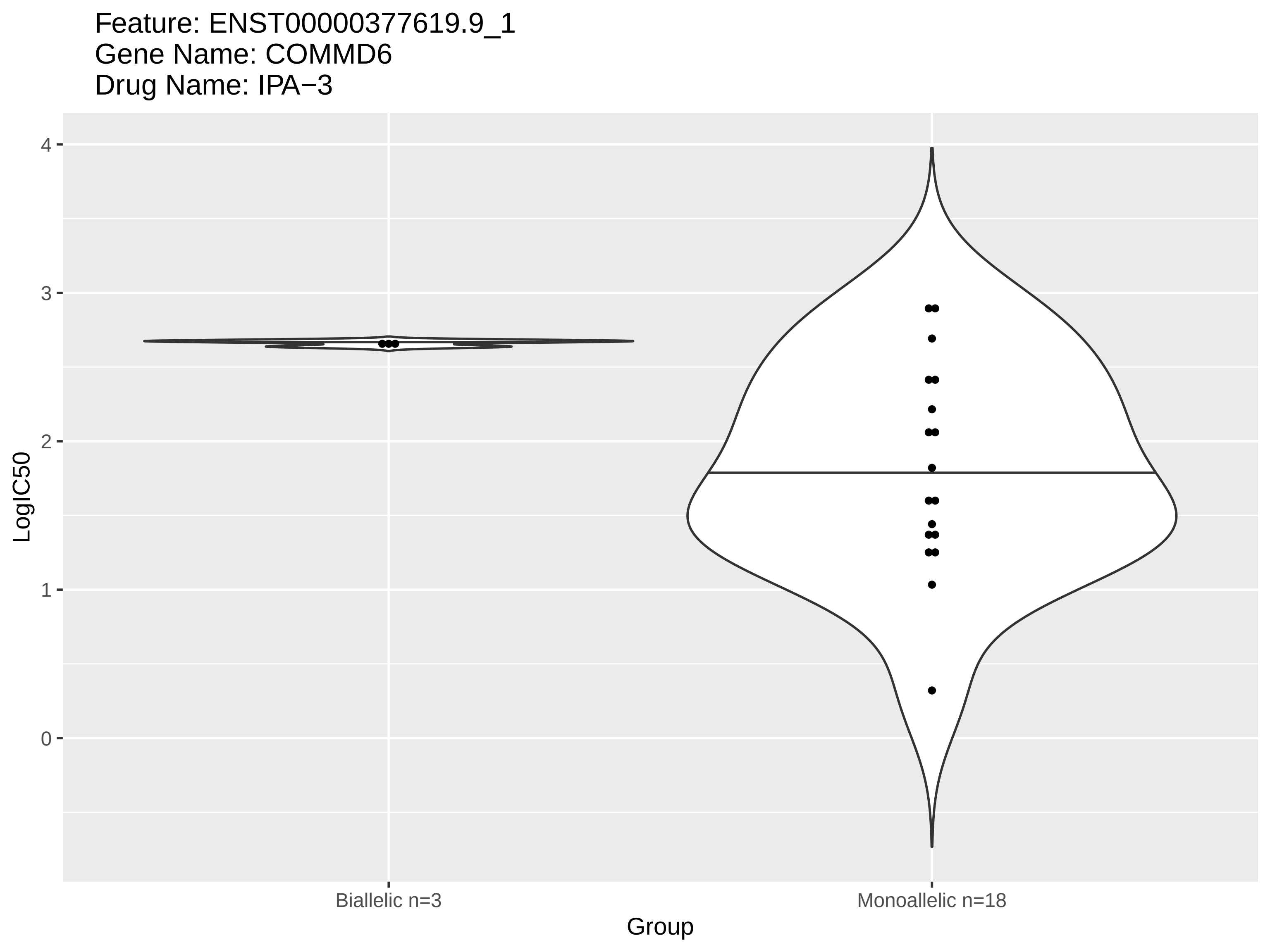
Feature: ENST00000377619.9_1
Gene Name: COMMD6
Drug Name: IPA−3
LogIC50
4
3
2
1
0
Biallelic n=3
Monoallelic n=18
Group

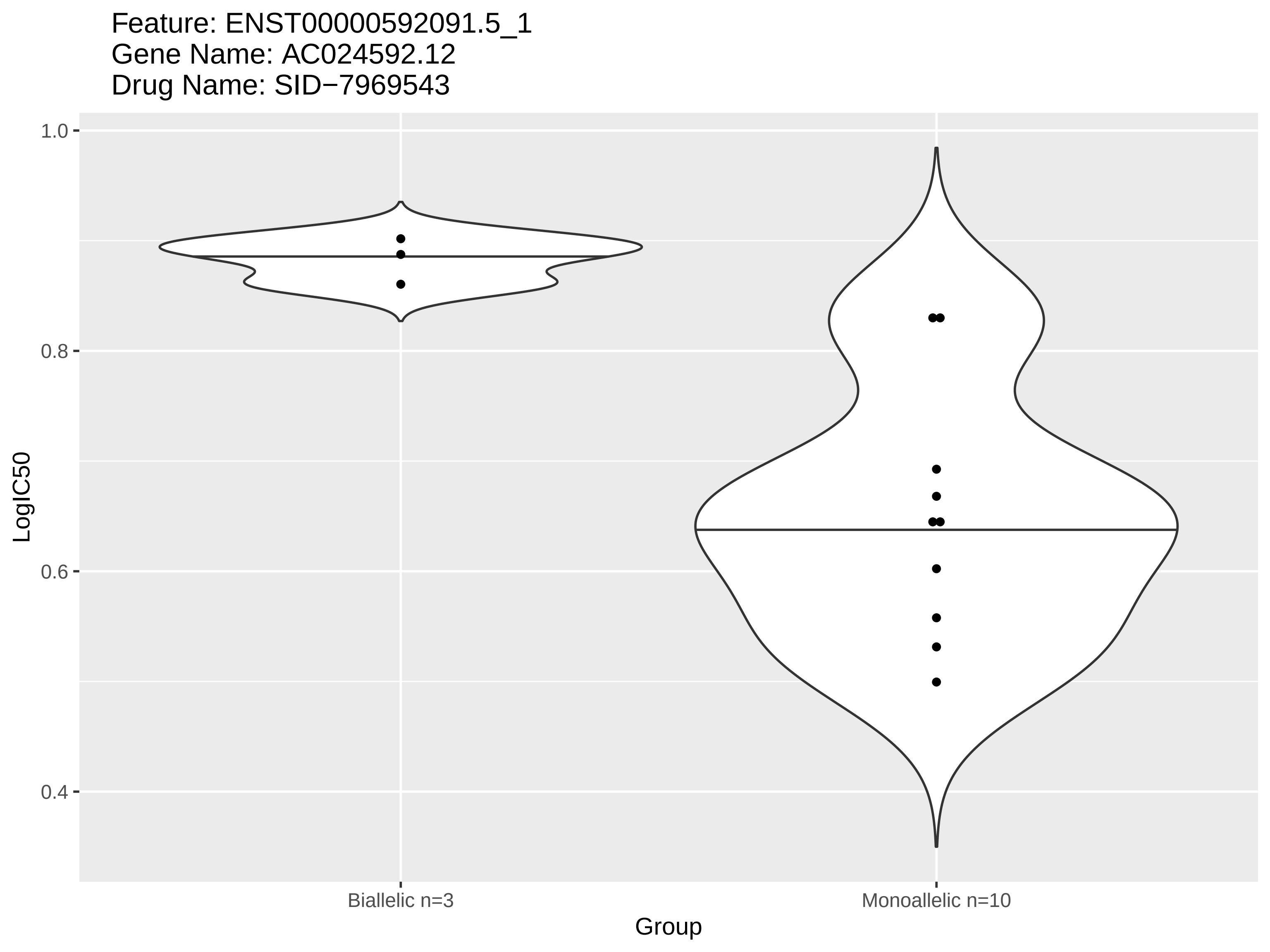
Feature: ENST00000592091.5_1
Gene Name: AC024592.12
Drug Name: SID−7969543
LogIC50
1.0
0.8
0.6
0.4
Biallelic n=3
Monoallelic n=10
Group

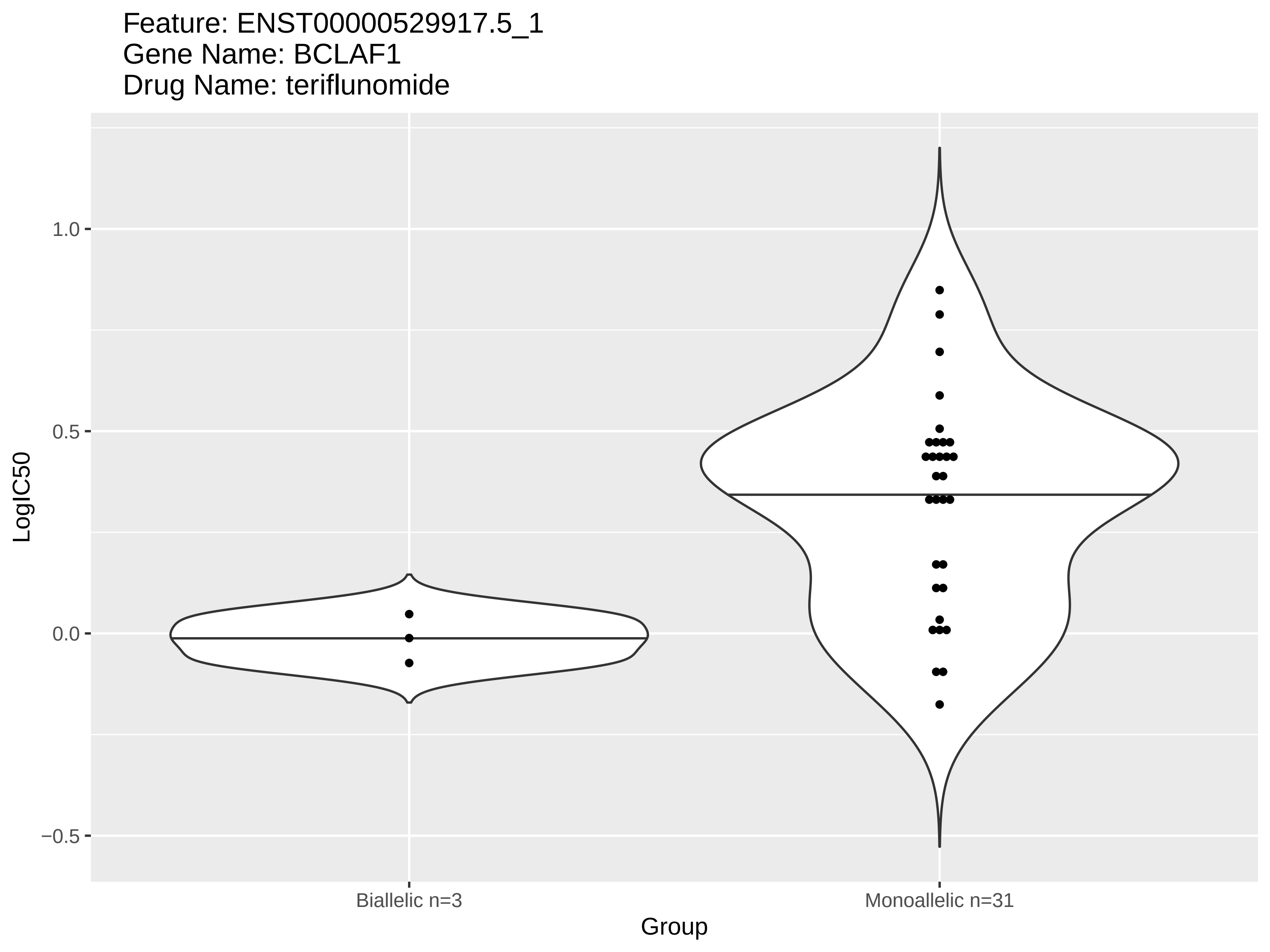
Feature: ENST00000529917.5_1
Gene Name: BCLAF1
Drug Name: teriflunomide
1.0
0.5
0.0
−0.5
LogIC50
Biallelic n=3
Monoallelic n=31
Group

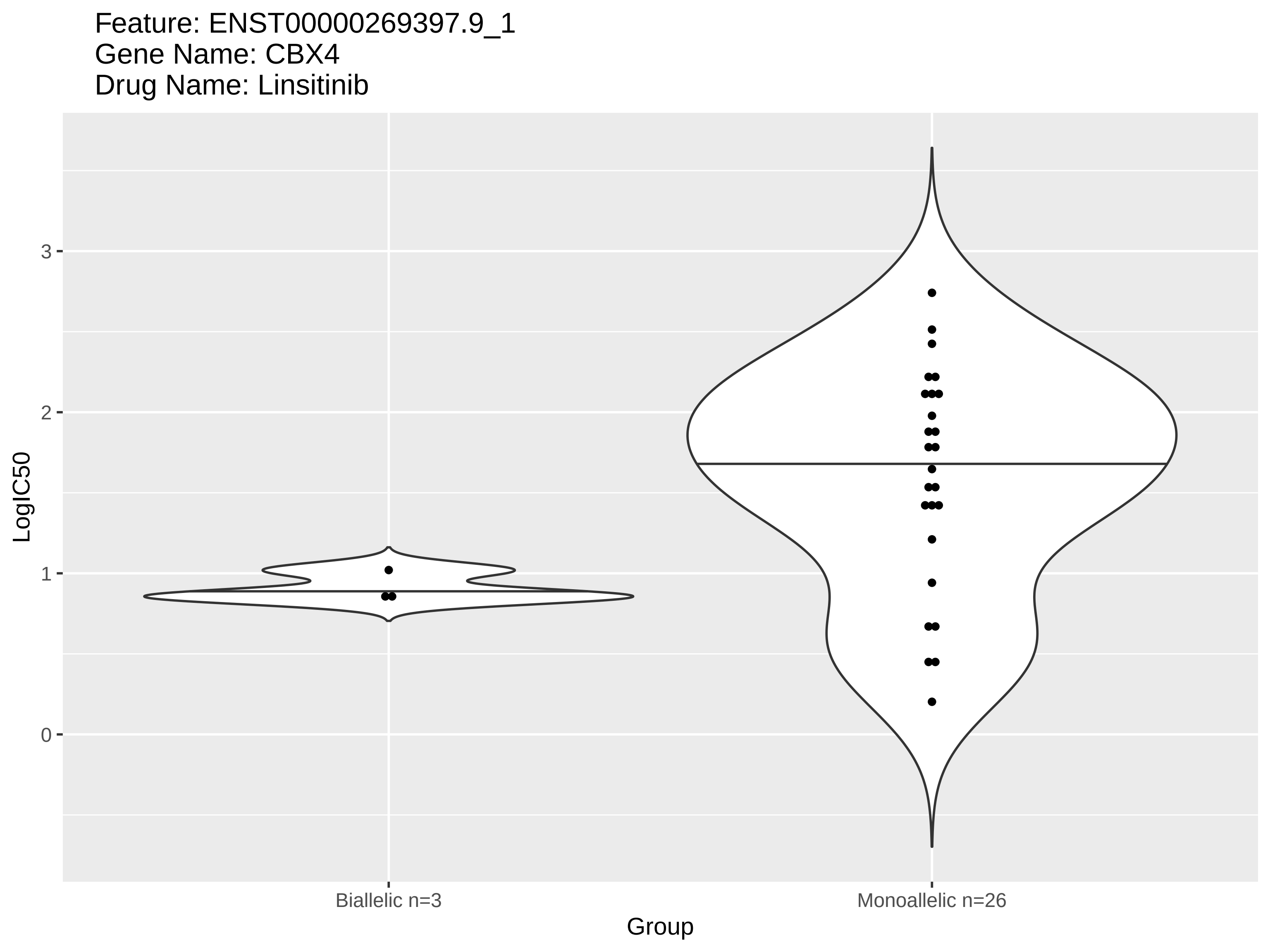

Feature: ENST00000269397.9_1
Gene Name: CBX4
Drug Name: Linsitinib
LogIC50
3
2
1
0
Biallelic n=3
Monoallelic n=26
Group

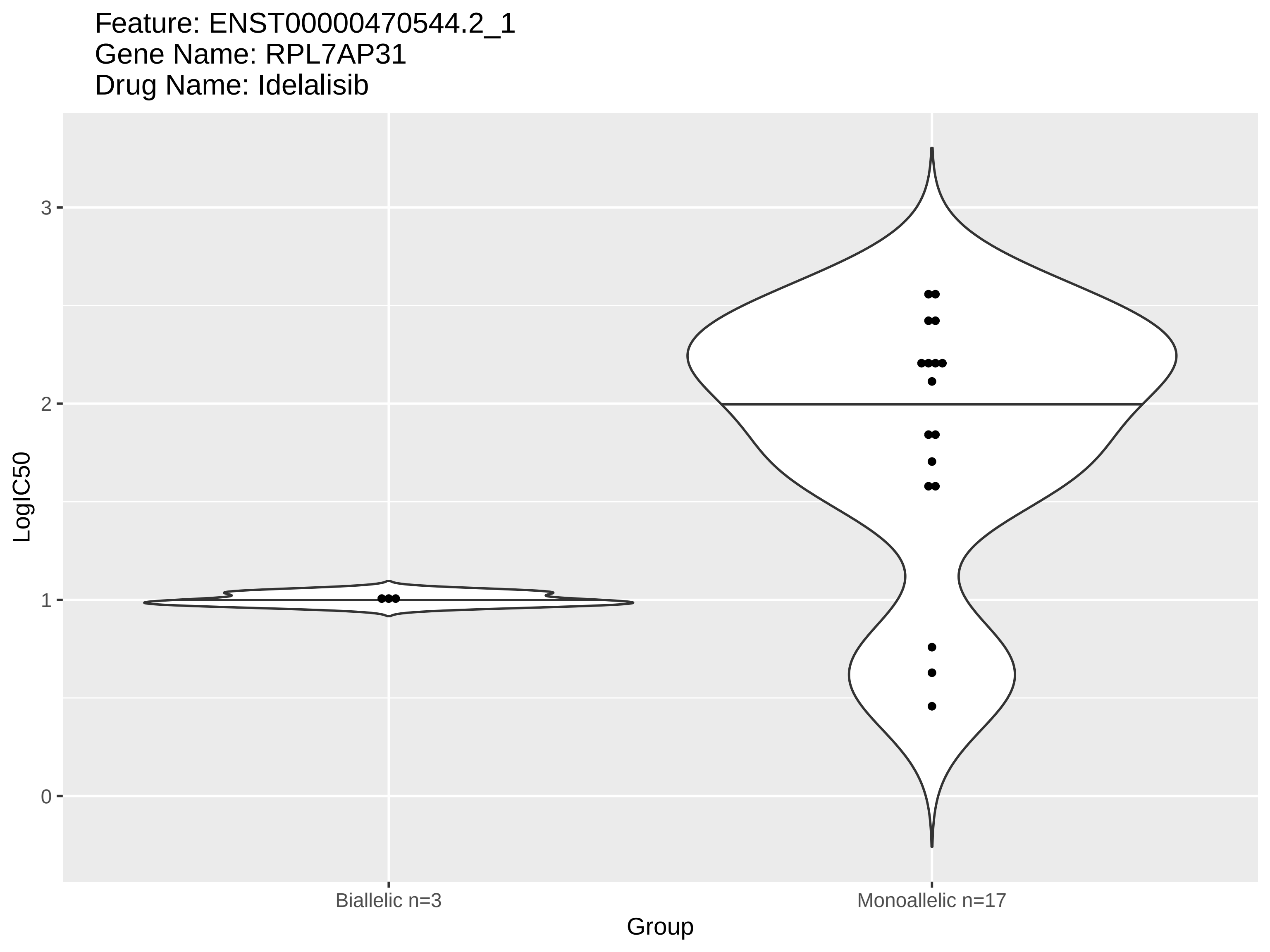
Feature: ENST00000470544.2_1
Gene Name: RPL7AP31
Drug Name: Idelalisib
LogIC50
3
2
1
0
Biallelic n=3
Monoallelic n=17
Group

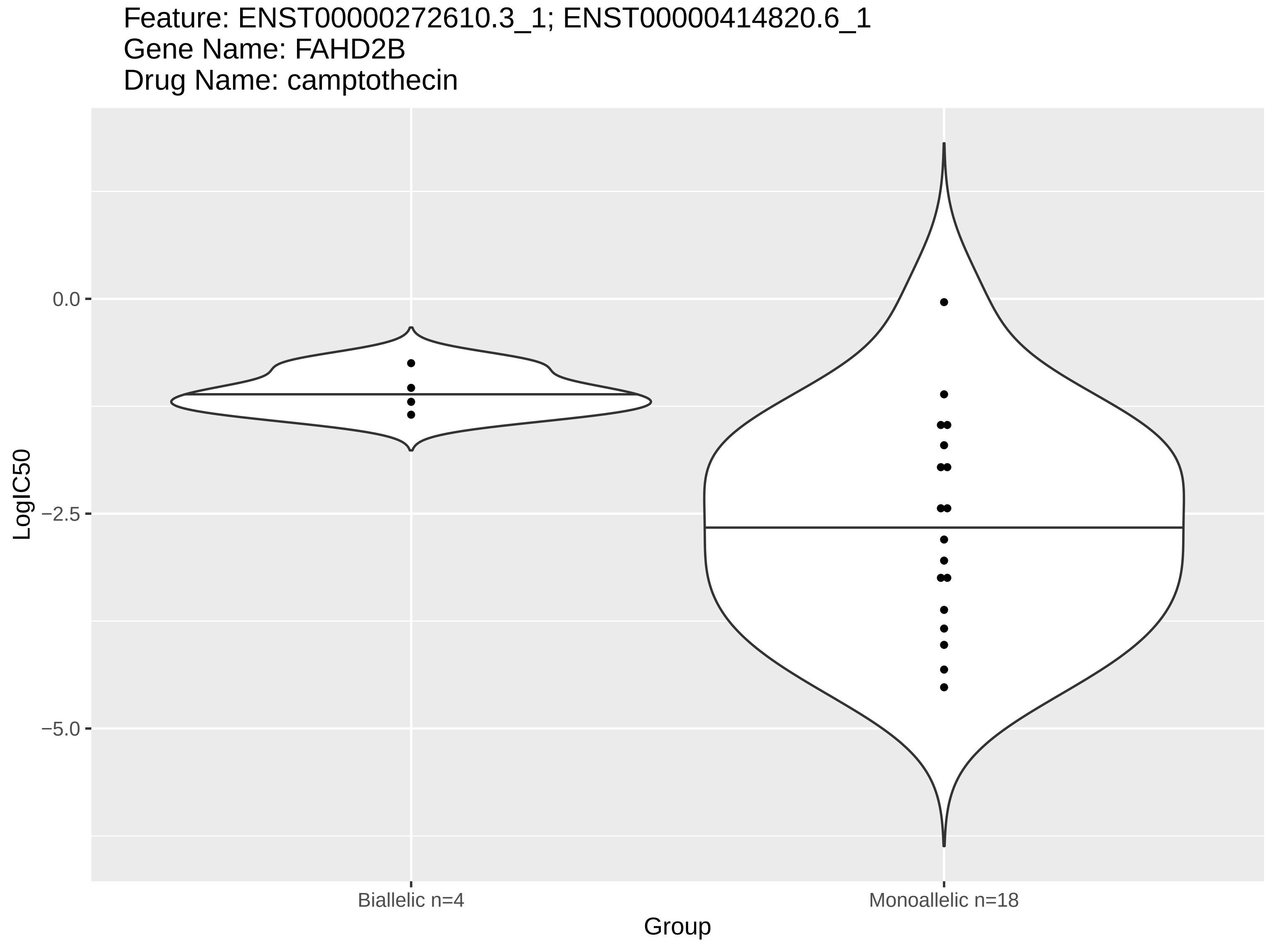

Feature: ENST00000272610.3_1; ENST00000414820.6_1
Gene Name: FAHD2B
Drug Name: camptothecin
0.0
−2.5
−5.0
LogIC50
Biallelic n=4
Monoallelic n=18
Group

Feature: ENST00000533422.5_1
Gene Name: BCLAF1
Drug Name: CX−5461

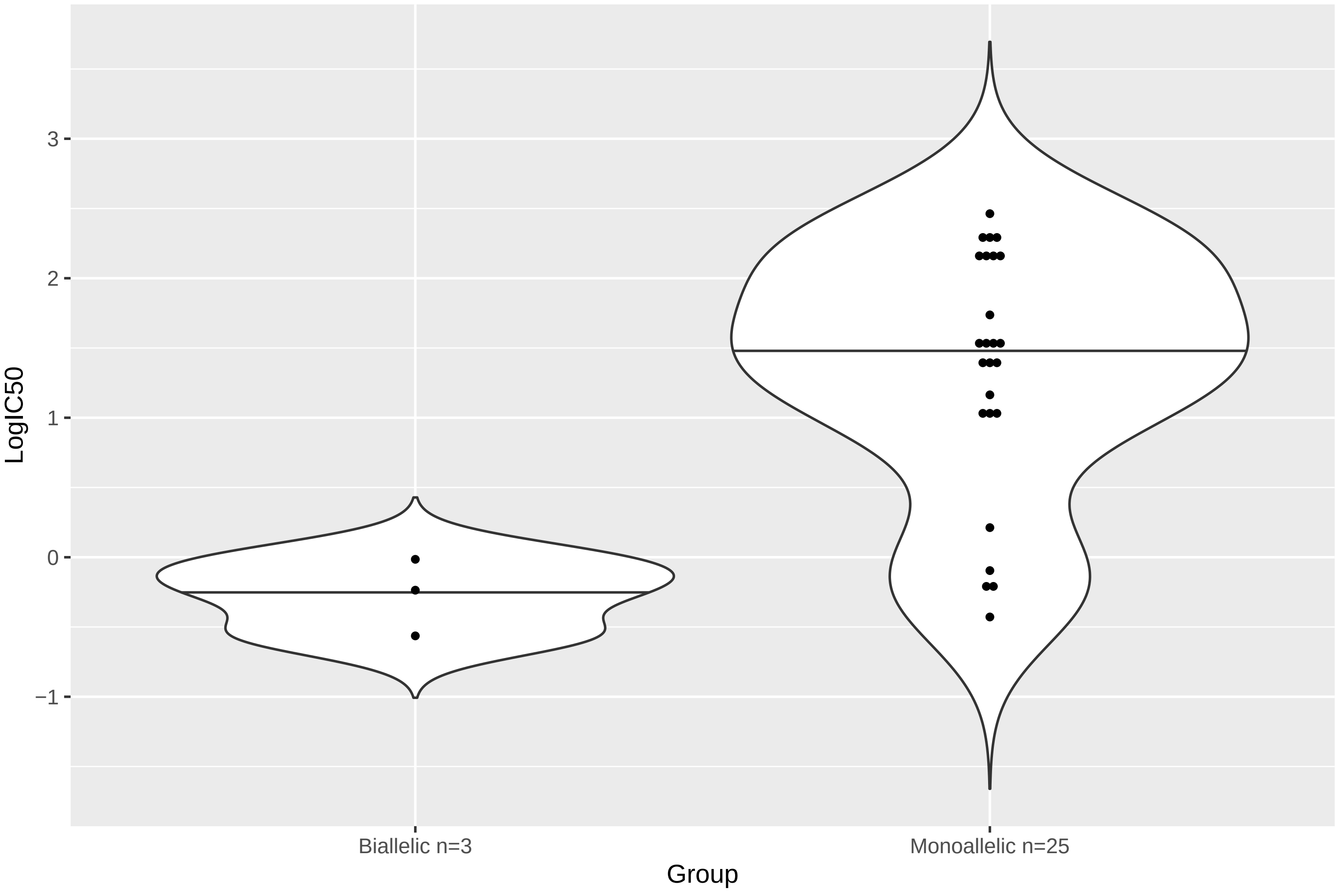

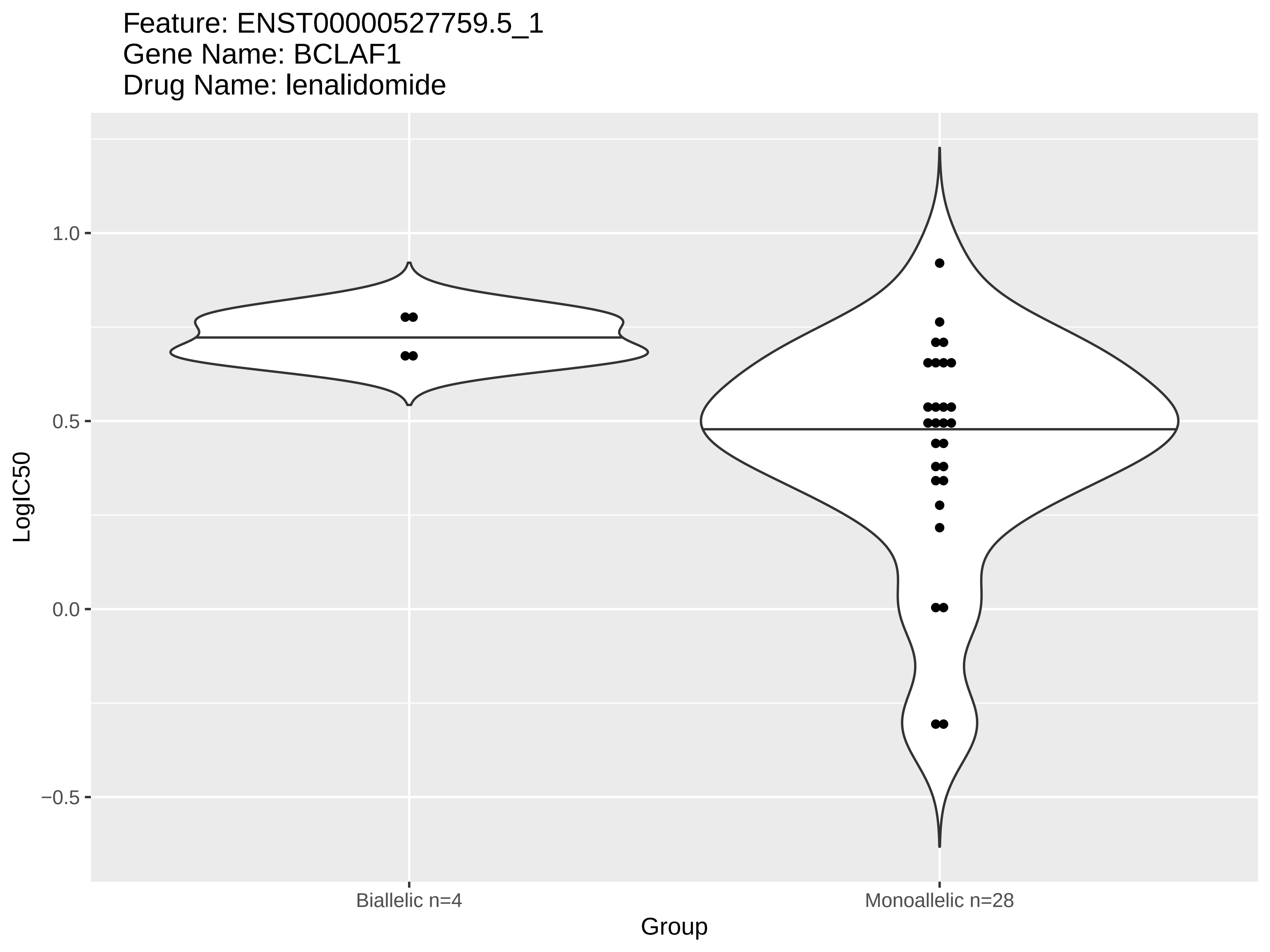
Feature: ENST00000527759.5_1
Gene Name: BCLAF1
Drug Name: lenalidomide
1.0
0.5
0.0
−0.5
LogIC50
Biallelic n=4
Monoallelic n=28
Group

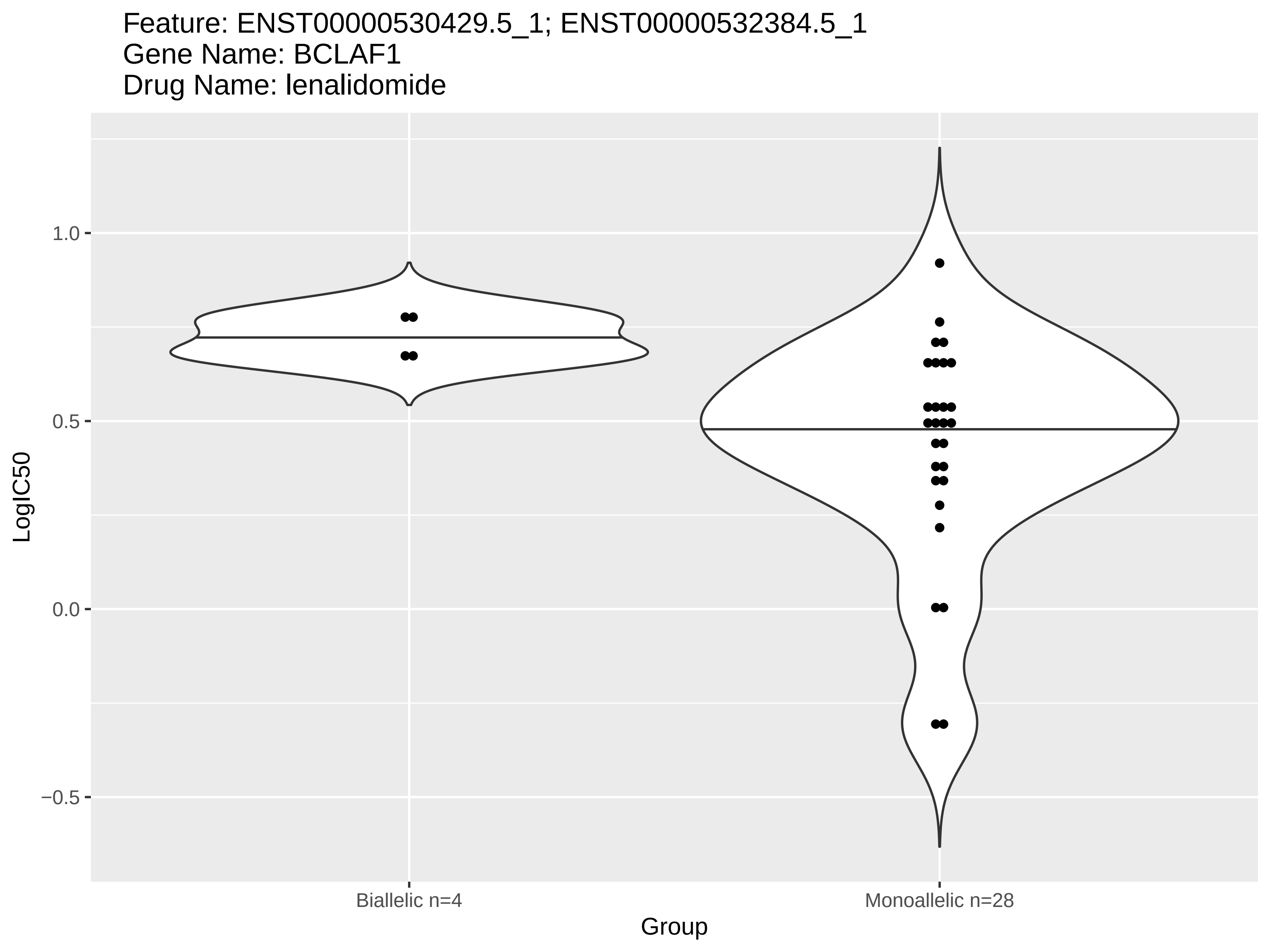
Feature: ENST00000530429.5_1; ENST00000532384.5_1
Gene Name: BCLAF1
Drug Name: lenalidomide
1.0
0.5
0.0
−0.5
LogIC50
Biallelic n=4
Monoallelic n=28
Group

Feature: ENST00000278882.8_1
Gene Name: FRG1BP
Drug Name: methscopolamine

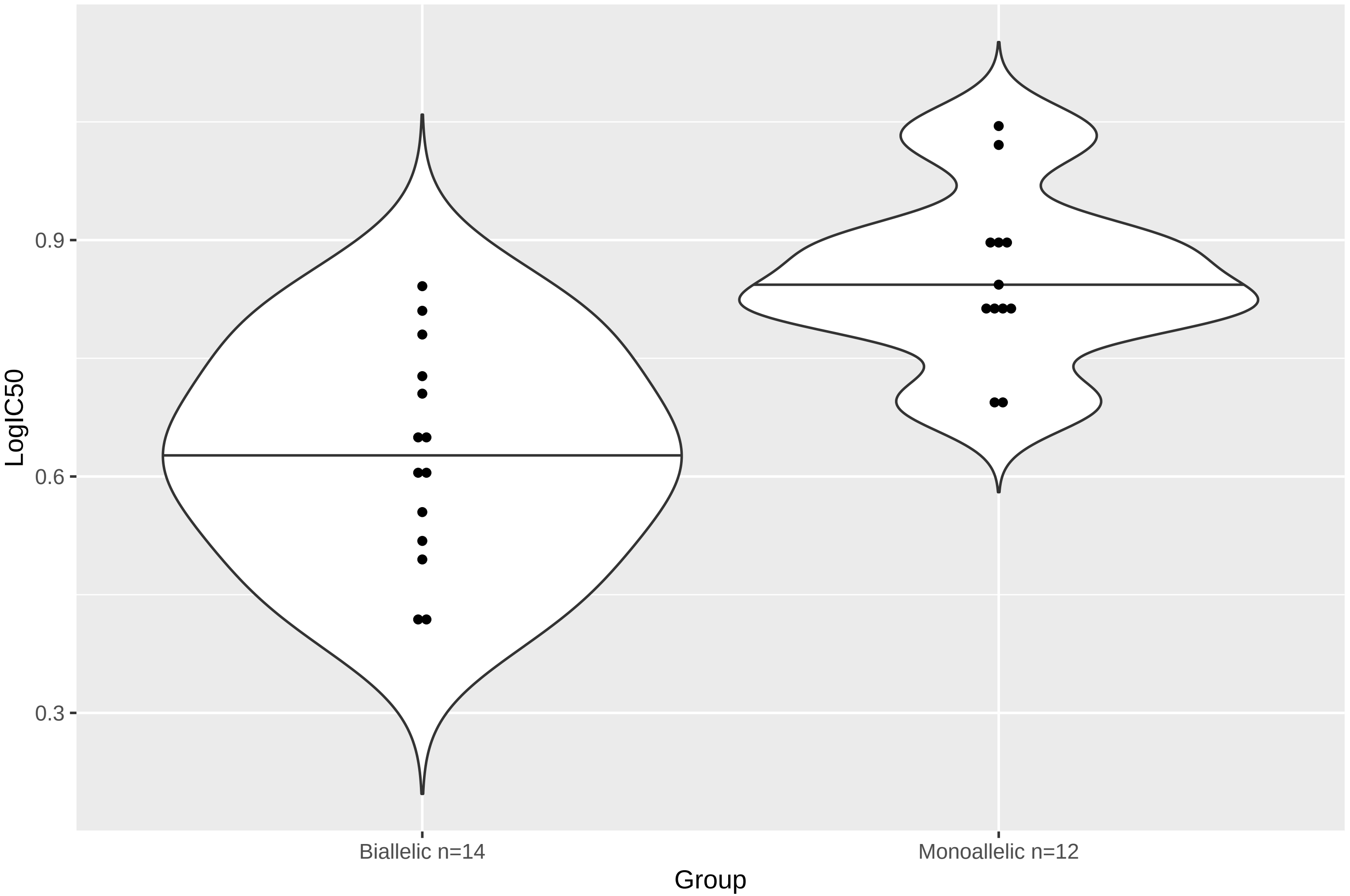

Feature: ENST00000615550.5_1; ENST00000672107.1_1
Gene Name: PRIM2
Drug Name: frentizole

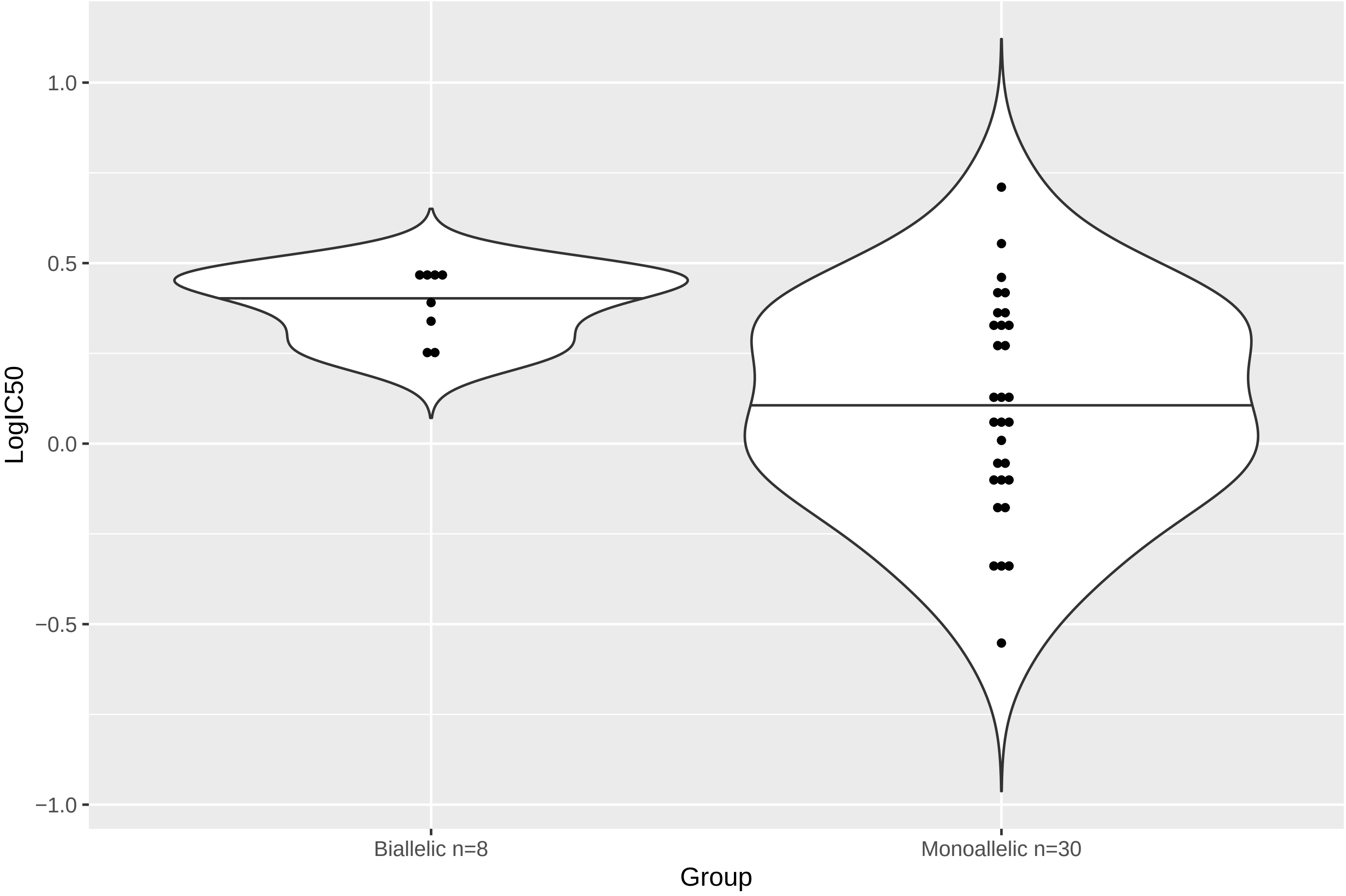

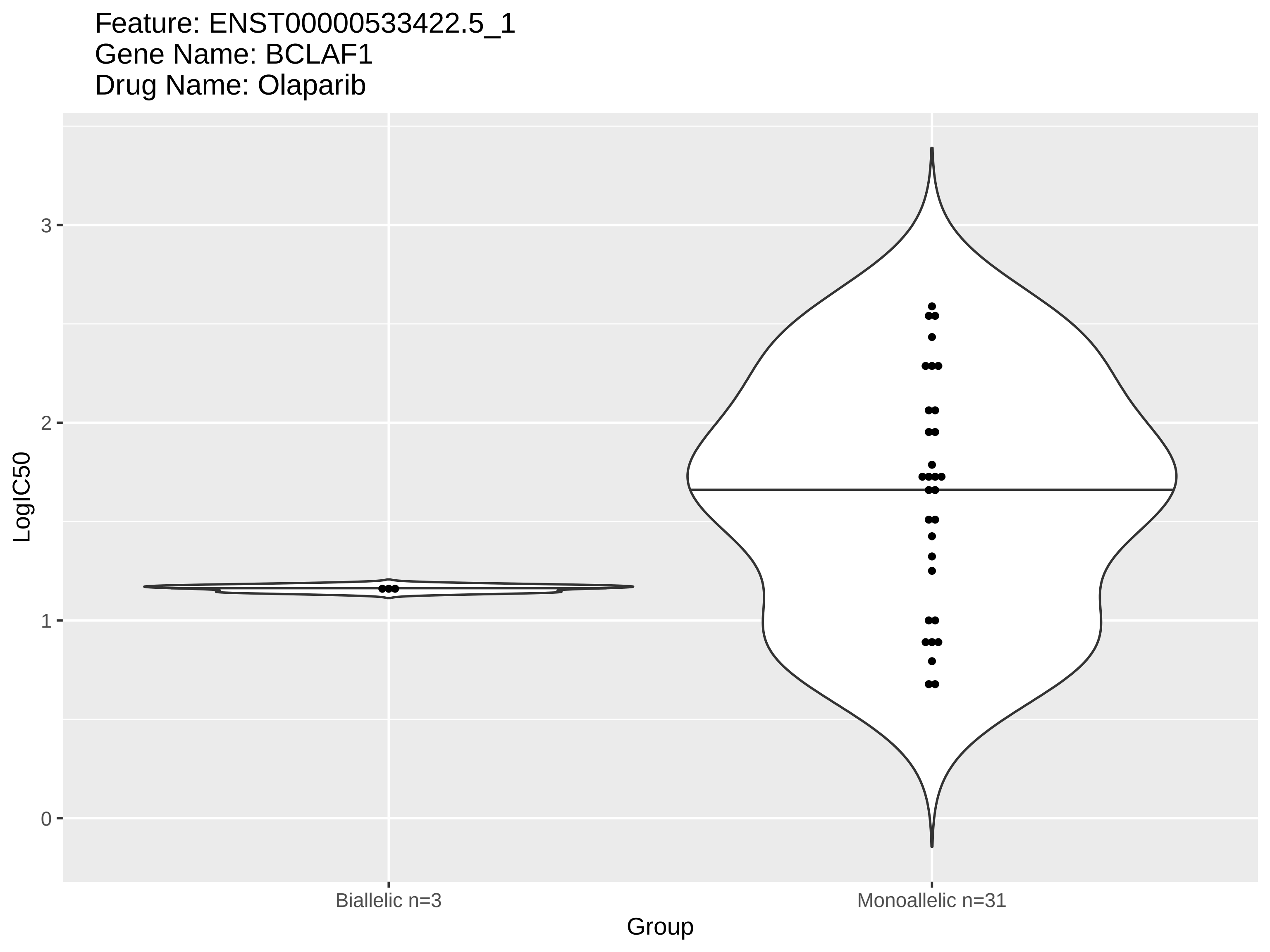
Feature: ENST00000533422.5_1
Gene Name: BCLAF1
Drug Name: Olaparib
LogIC50
3
2
1
0
Biallelic n=3
Monoallelic n=31
Group

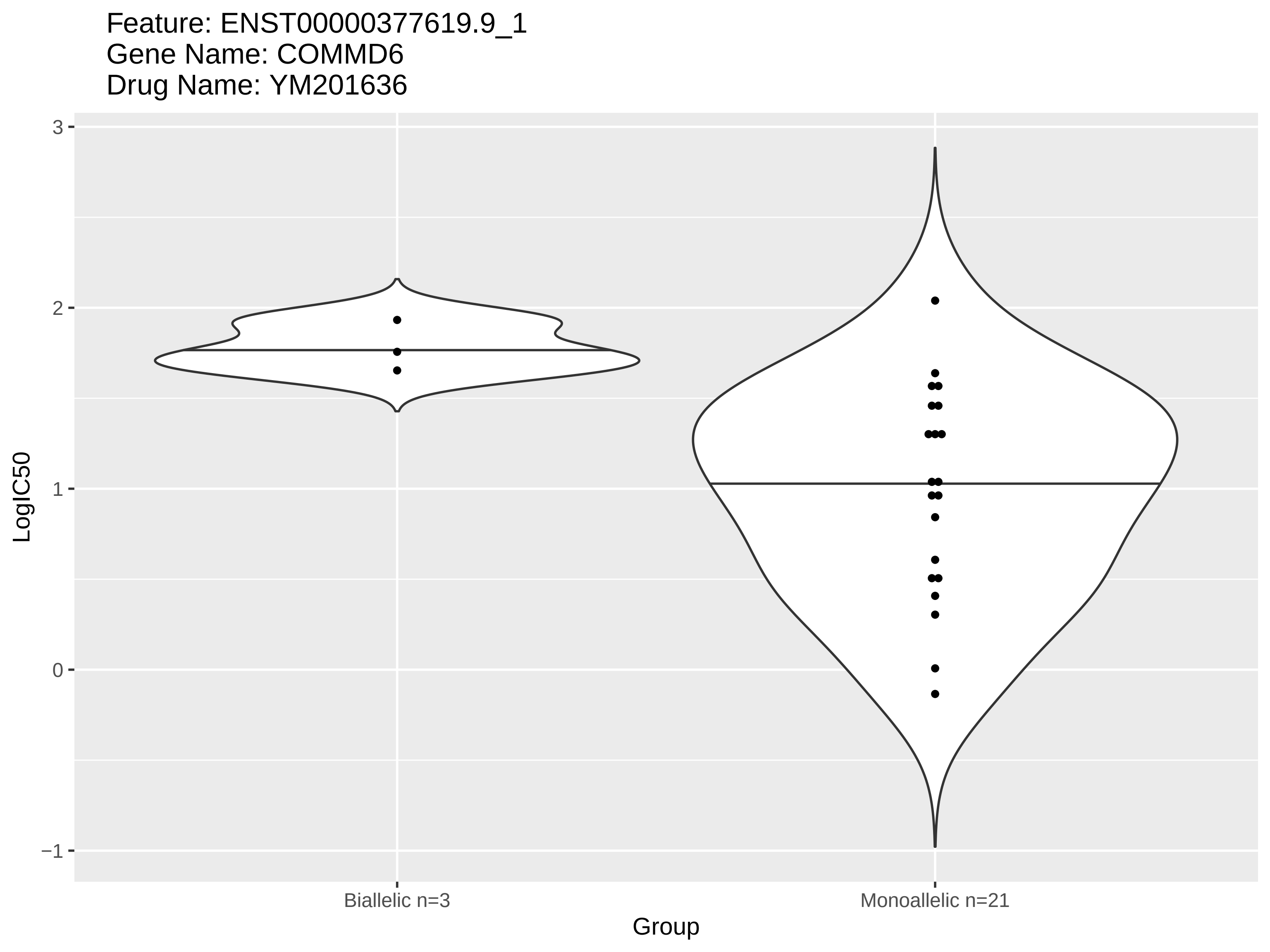

Feature: ENST00000377619.9_1
Gene Name: COMMD6
Drug Name: YM201636
3
2
1
0
−1
LogIC50
Biallelic n=3
Monoallelic n=21
Group

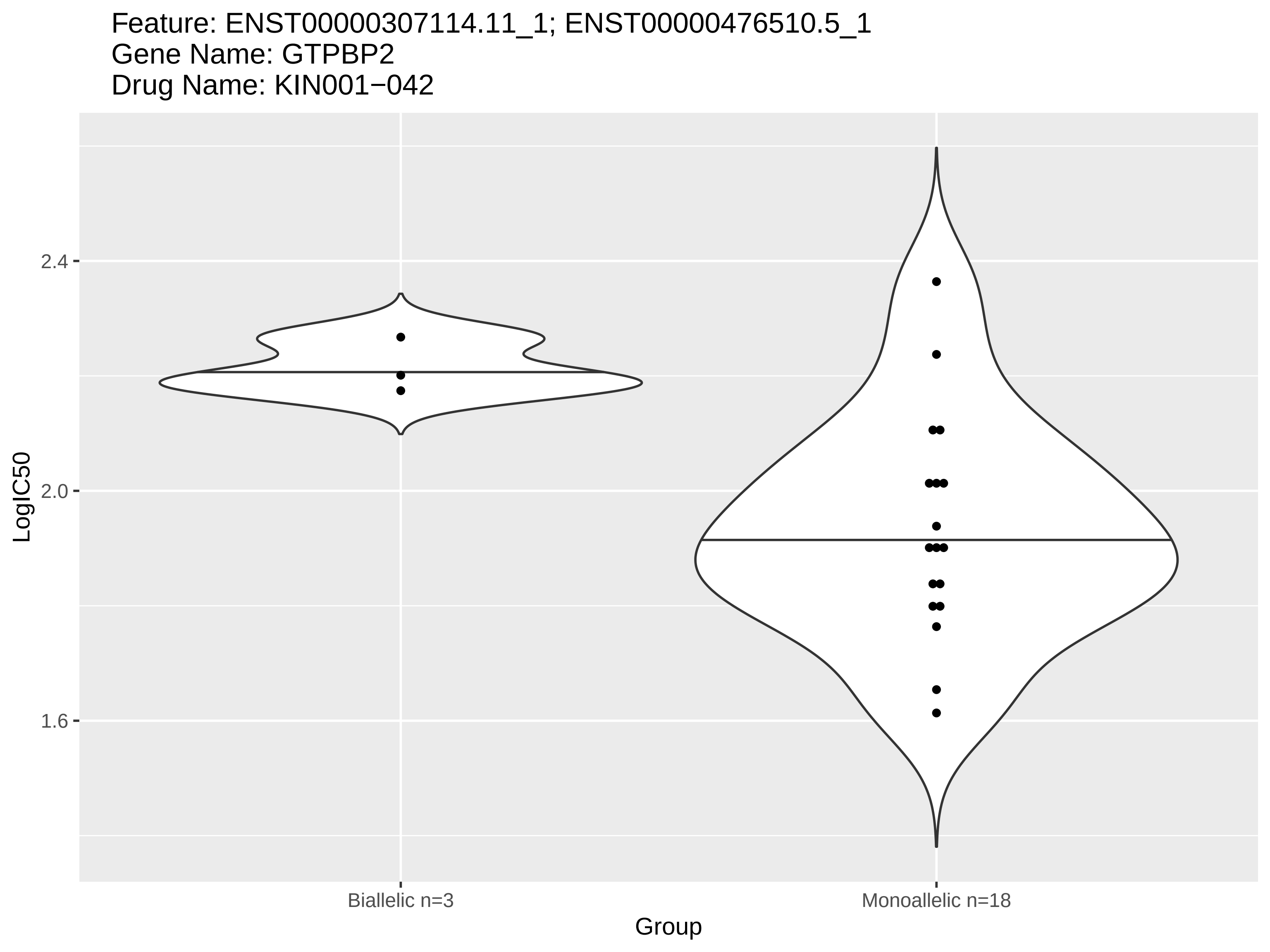

Feature: ENST00000307114.11_1; ENST00000476510.5_1
Gene Name: GTPBP2
Drug Name: KIN001−042
LogIC50
2.4
2.0
1.6
Biallelic n=3
Monoallelic n=18
Group

Feature: ENST00000432918.5_1
Gene Name: GTPBP2
Drug Name: KIN001−042

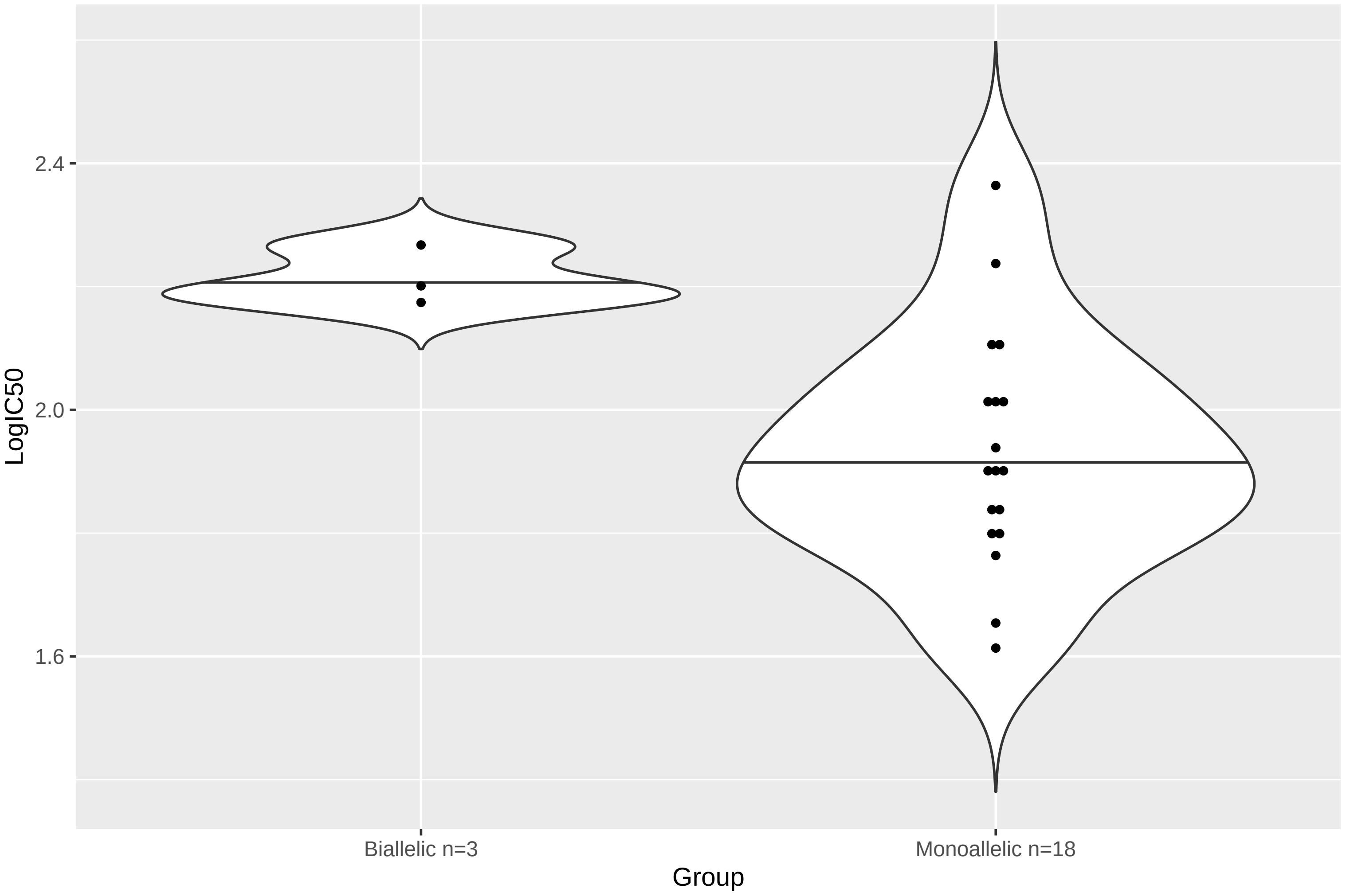

Feature: ENST00000443998.1_1
Gene Name: RP11−632C17__A.1
Drug Name: IAP_5620

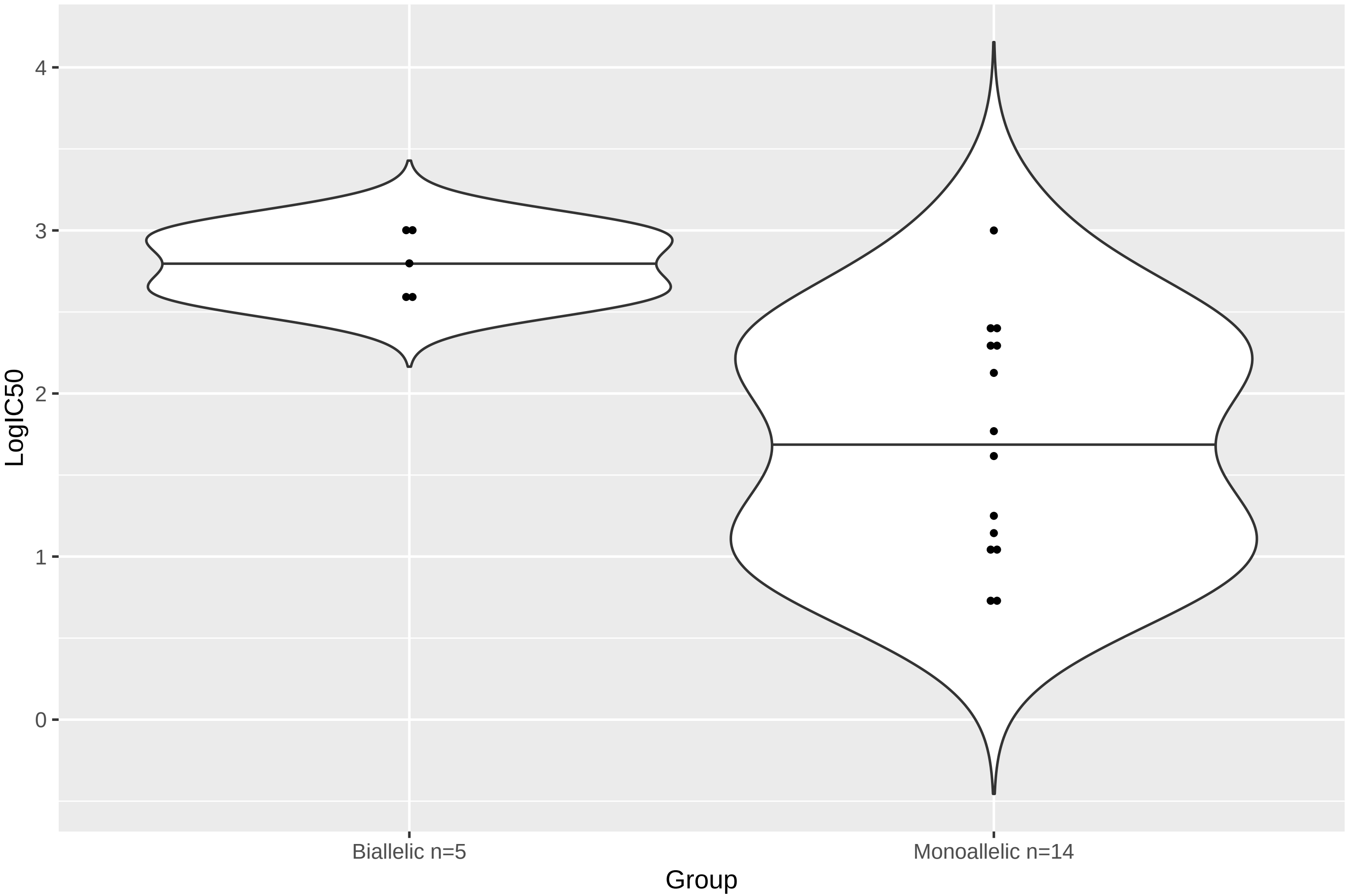

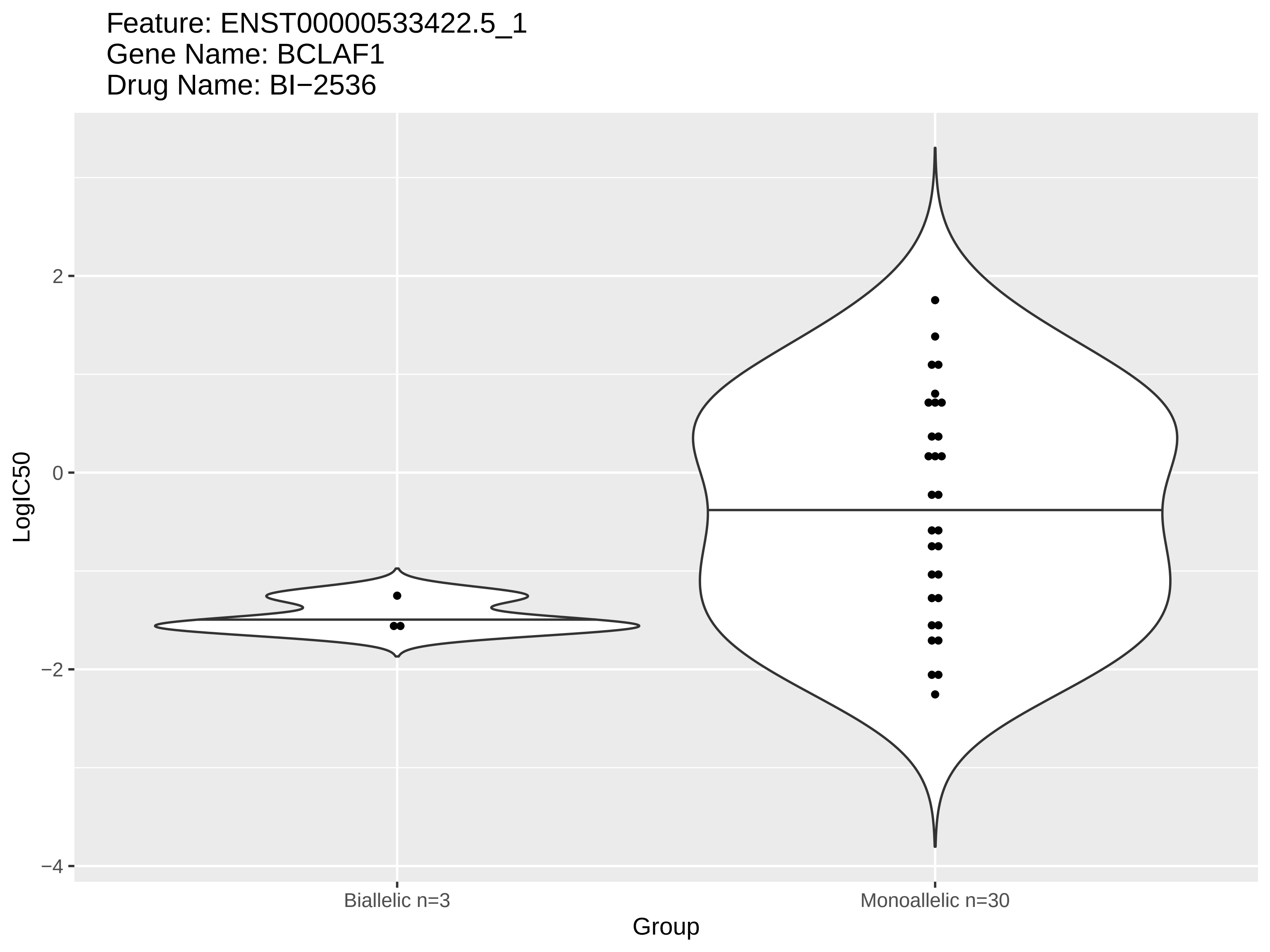

Feature: ENST00000533422.5_1
Gene Name: BCLAF1
Drug Name: BI−2536
LogIC50
2
0
−2
−4
Biallelic n=3
Monoallelic n=30
Group

Feature: ENST00000066544.8_1
Gene Name: CDC27
Drug Name: danusertib

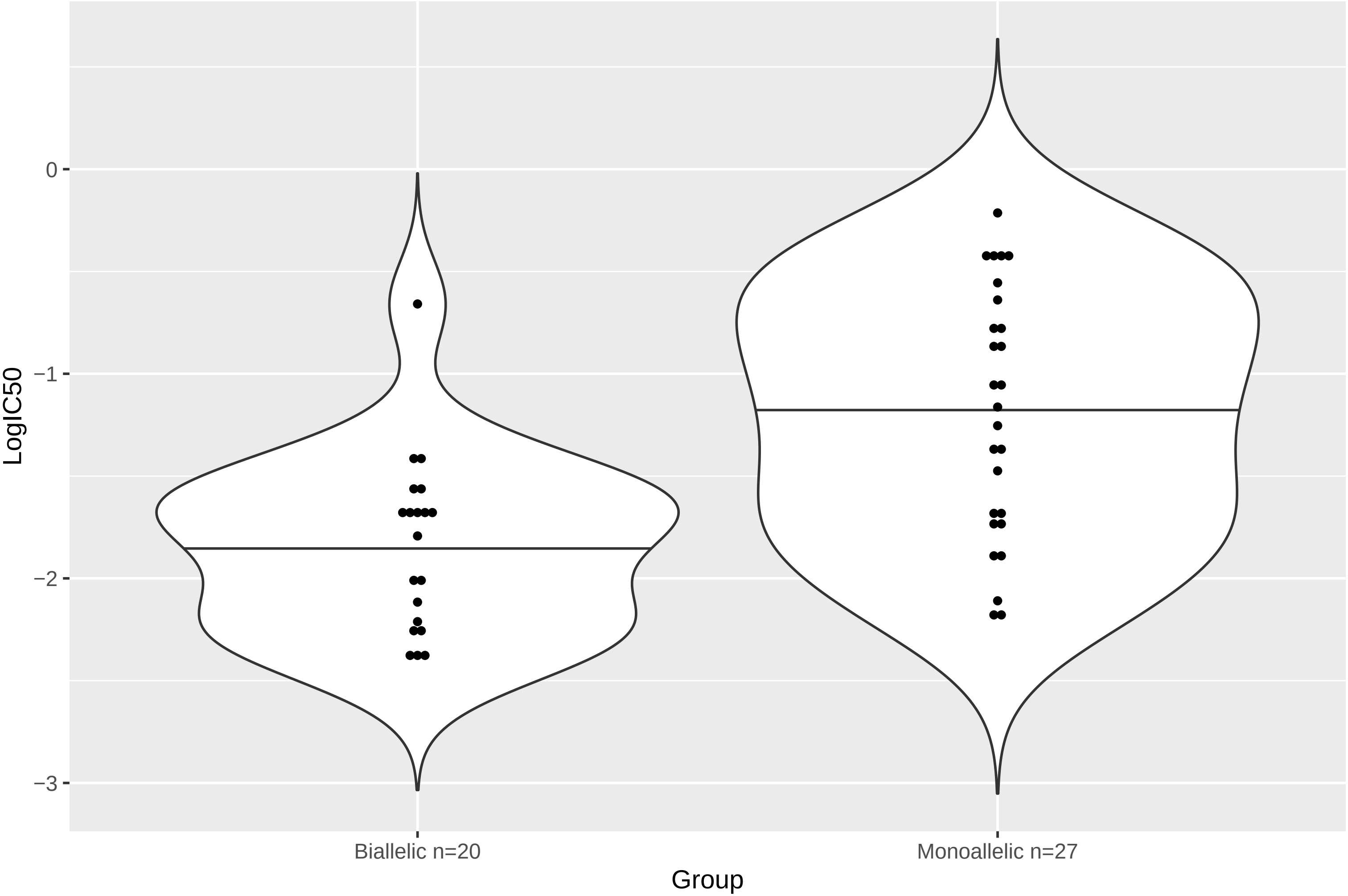

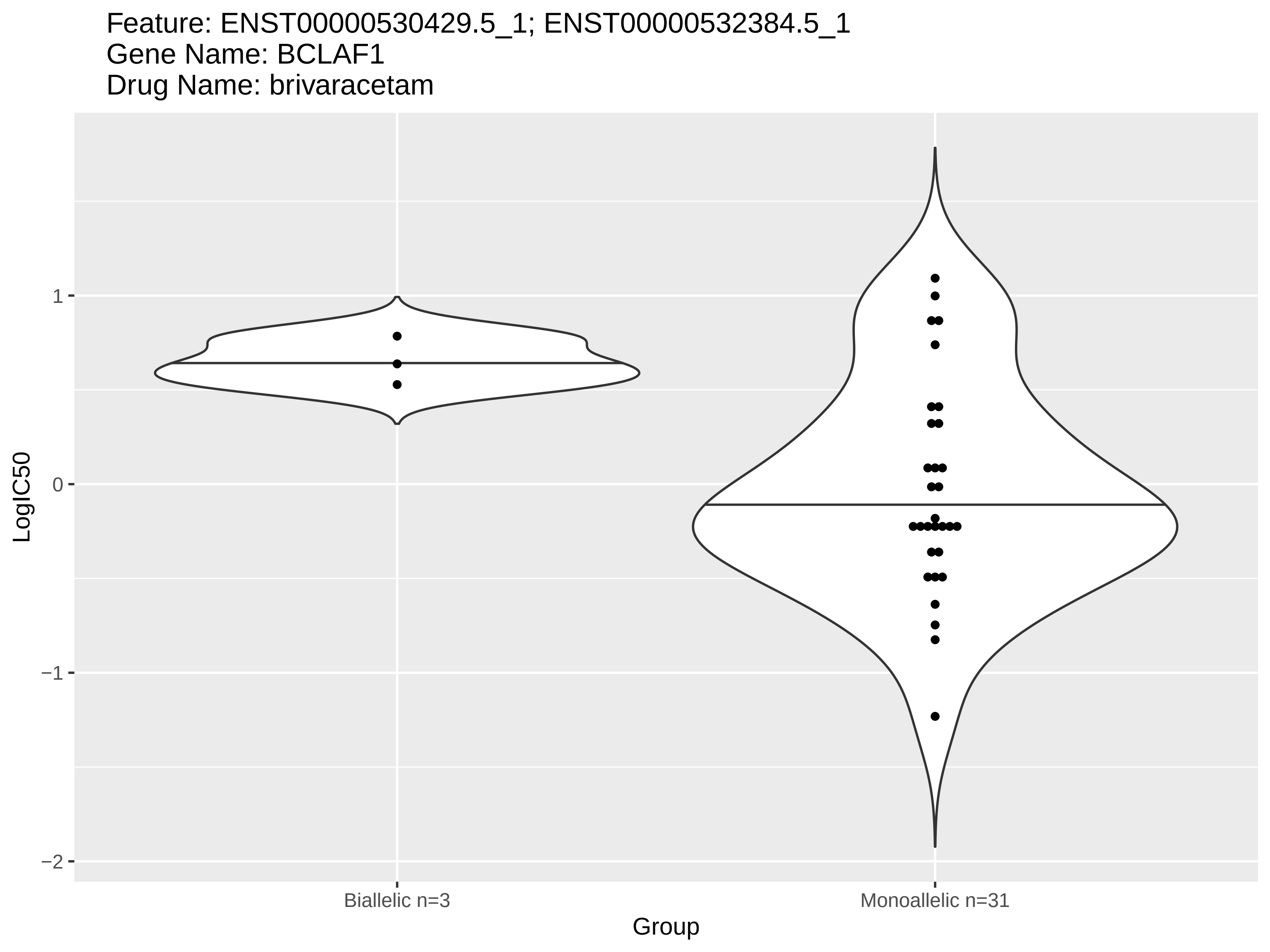
Feature: ENST00000530429.5_1; ENST00000532384.5_1
Gene Name: BCLAF1
Drug Name: brivaracetam
LogIC50
1
0
−1
−2
Biallelic n=3
Monoallelic n=31
Group

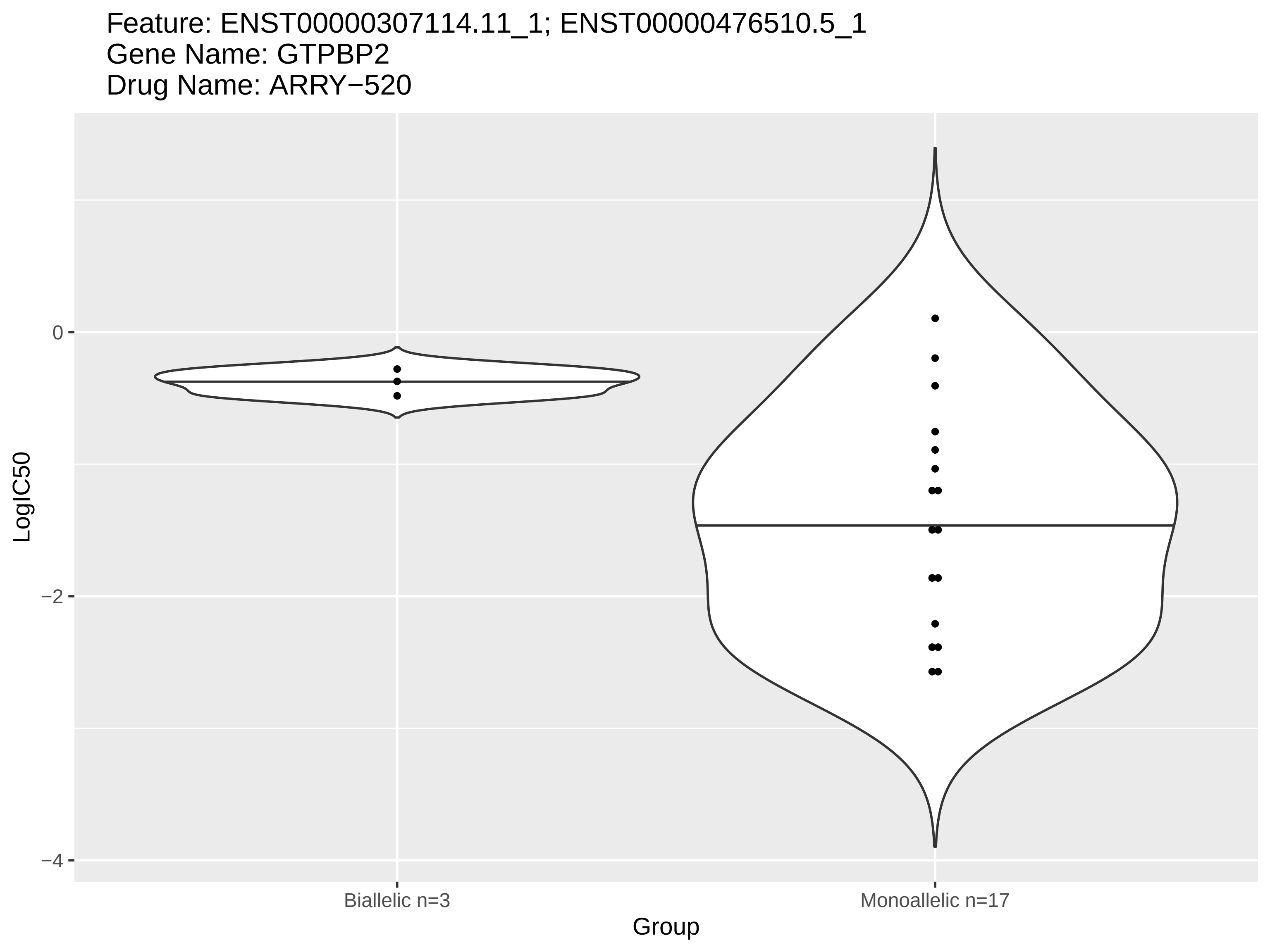
Feature: ENST00000307114.11_1; ENST00000476510.5_1
Gene Name: GTPBP2
Drug Name: ARRY−520
0
−2
−4
LogIC50
Biallelic n=3
Monoallelic n=17
Group

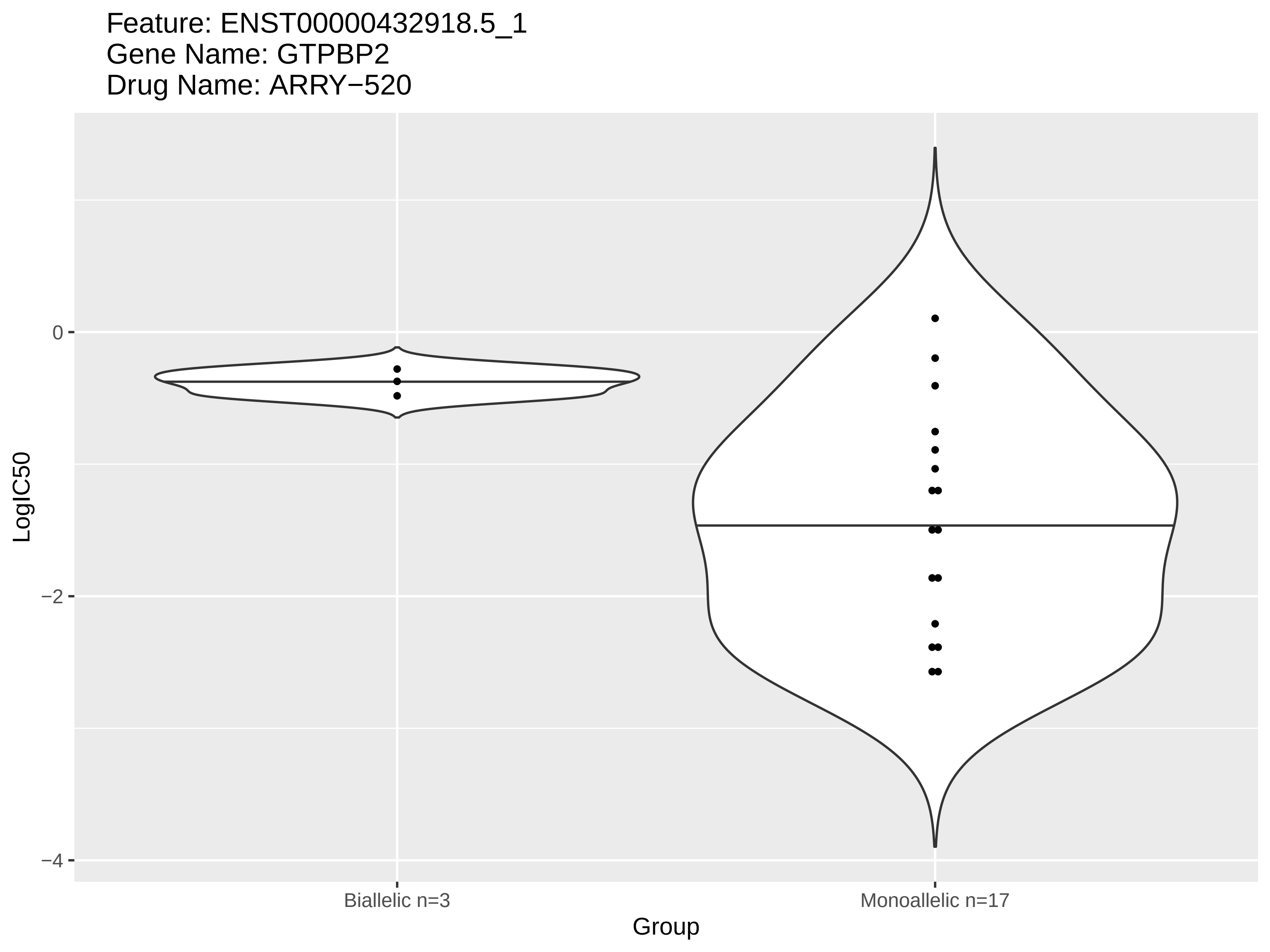

Feature: ENST00000432918.5_1
Gene Name: GTPBP2
Drug Name: ARRY−520
LogIC50
0
−2
−4
Biallelic n=3
Monoallelic n=17
Group

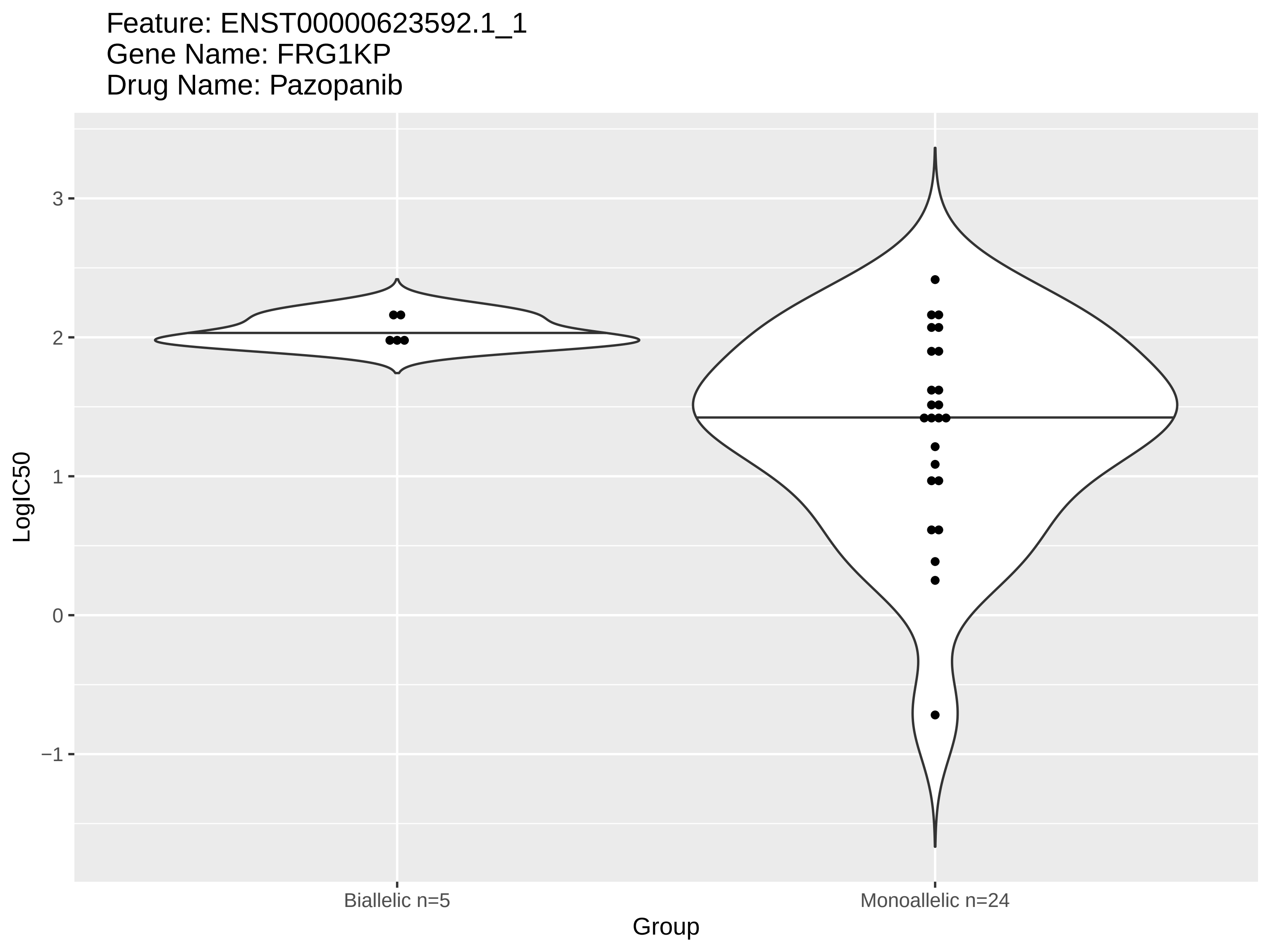
Feature: ENST00000623592.1_1
Gene Name: FRG1KP
Drug Name: Pazopanib
LogIC50
3
2
1
0
−1
Biallelic n=5
Monoallelic n=24
Group

Feature: ENST00000392348.6_1; ENST00000529826.5_1; ENST00000628517.2_1
Gene Name: BCLAF1
Drug Name: batimastat

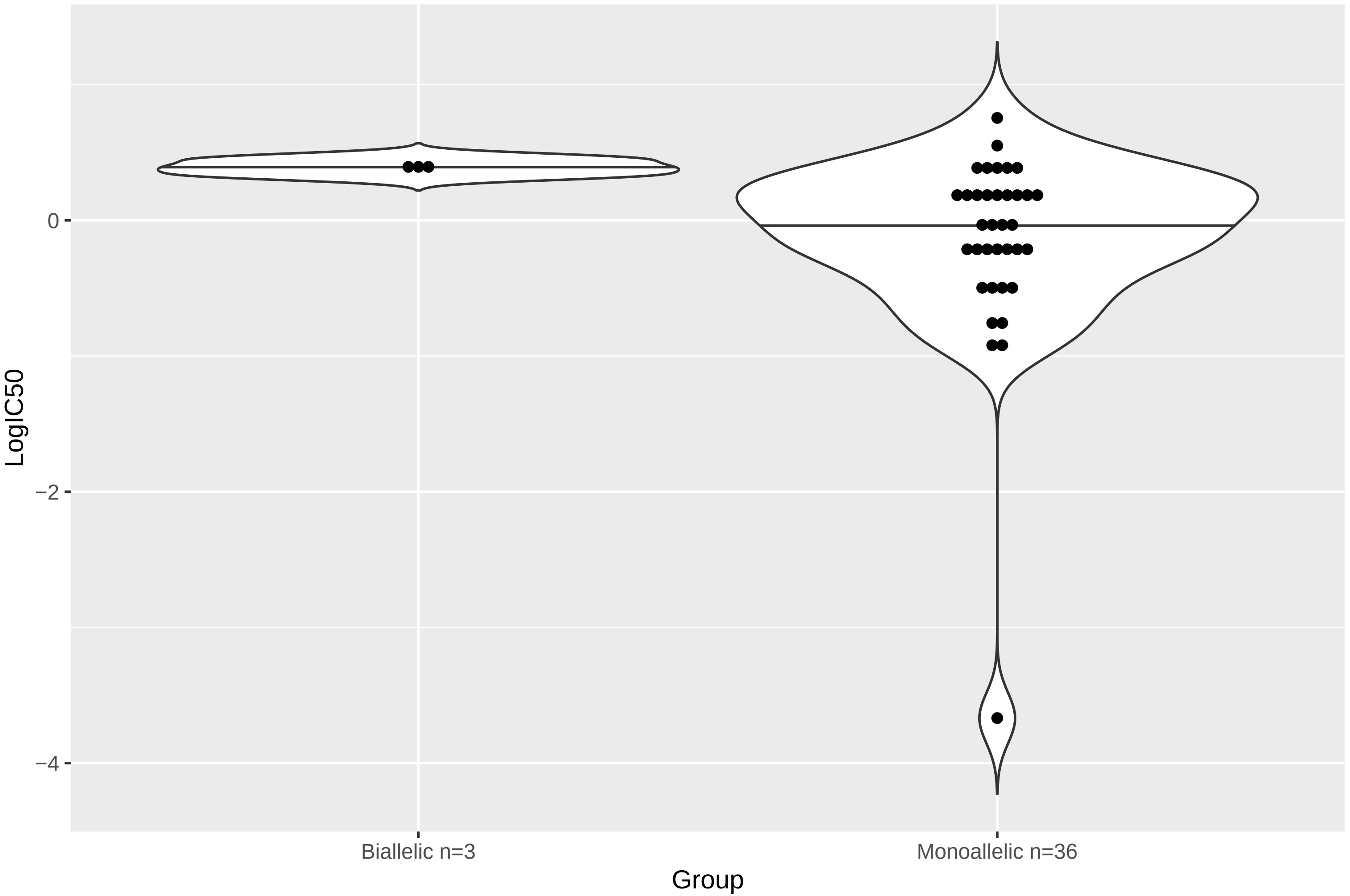

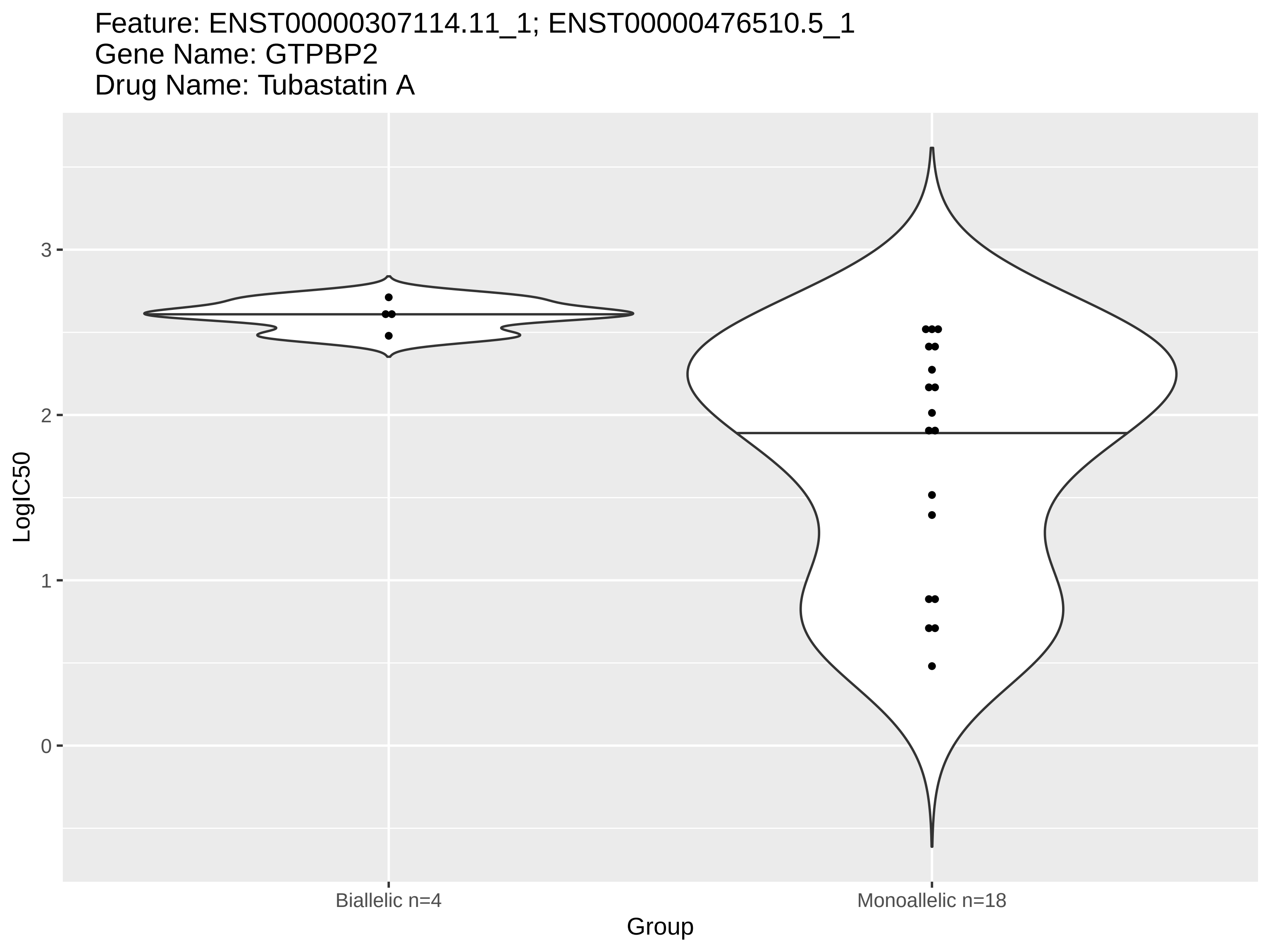

Feature: ENST00000307114.11_1; ENST00000476510.5_1
Gene Name: GTPBP2
Drug Name: Tubastatin A
LogIC50
3
2
1
0
Biallelic n=4
Monoallelic n=18
Group

Feature: ENST00000432918.5_1
Gene Name: GTPBP2
Drug Name: Tubastatin A

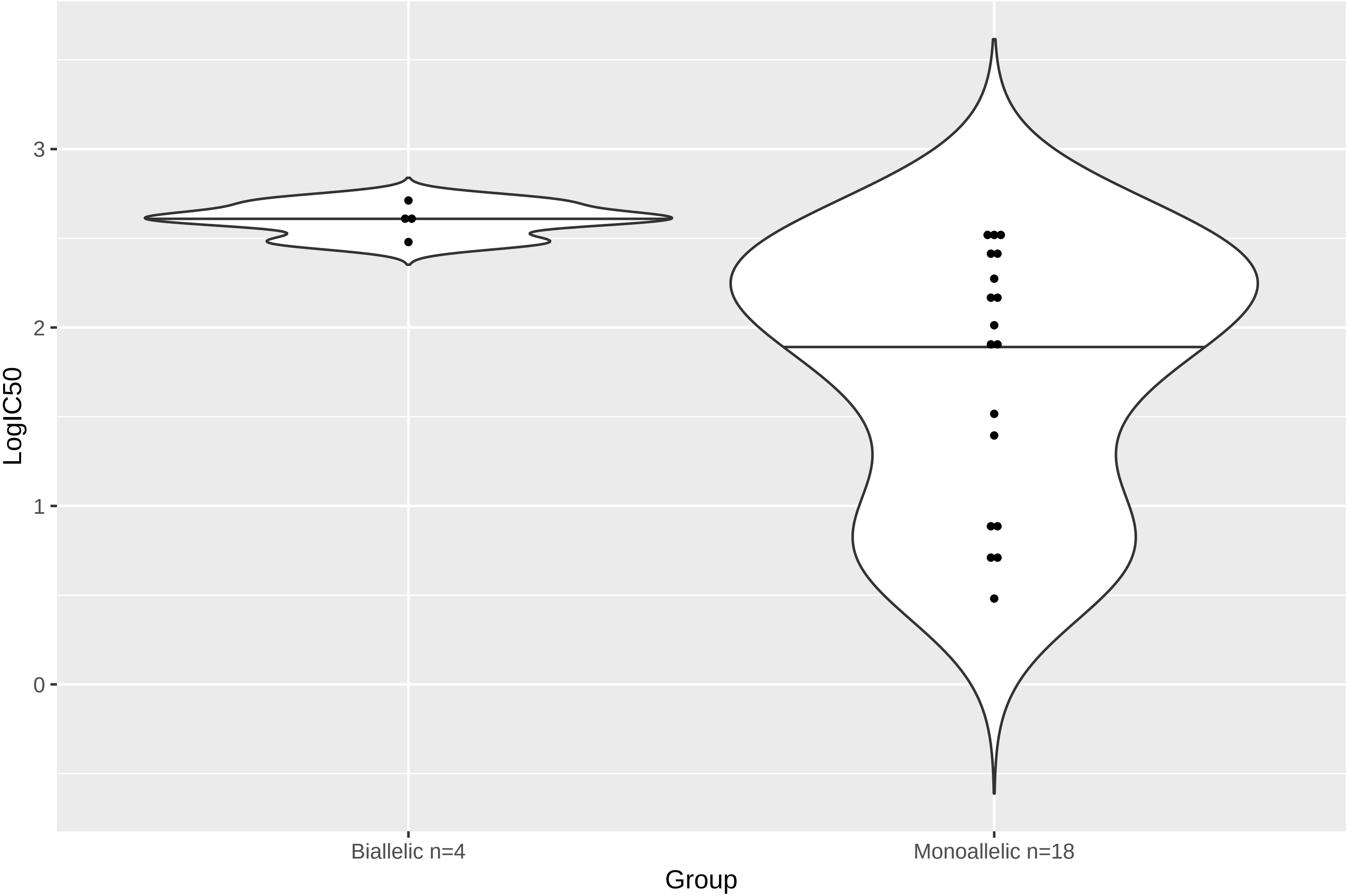

Feature: ENST00000571643.5_1
Gene Name: CDC27
Drug Name: FGFR_3831

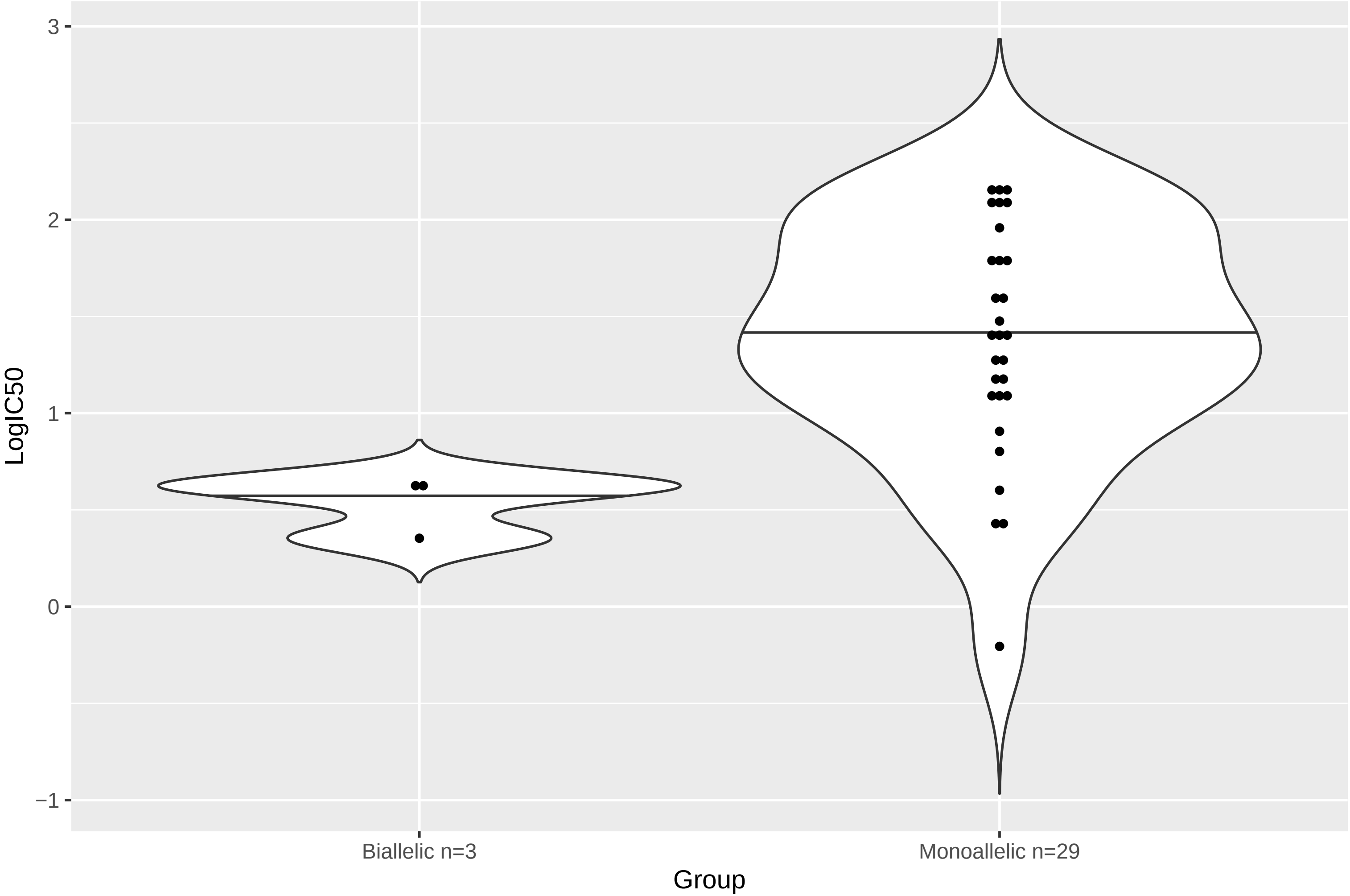

Feature: ENST00000533422.5_1
Gene Name: BCLAF1
Drug Name: Cediranib

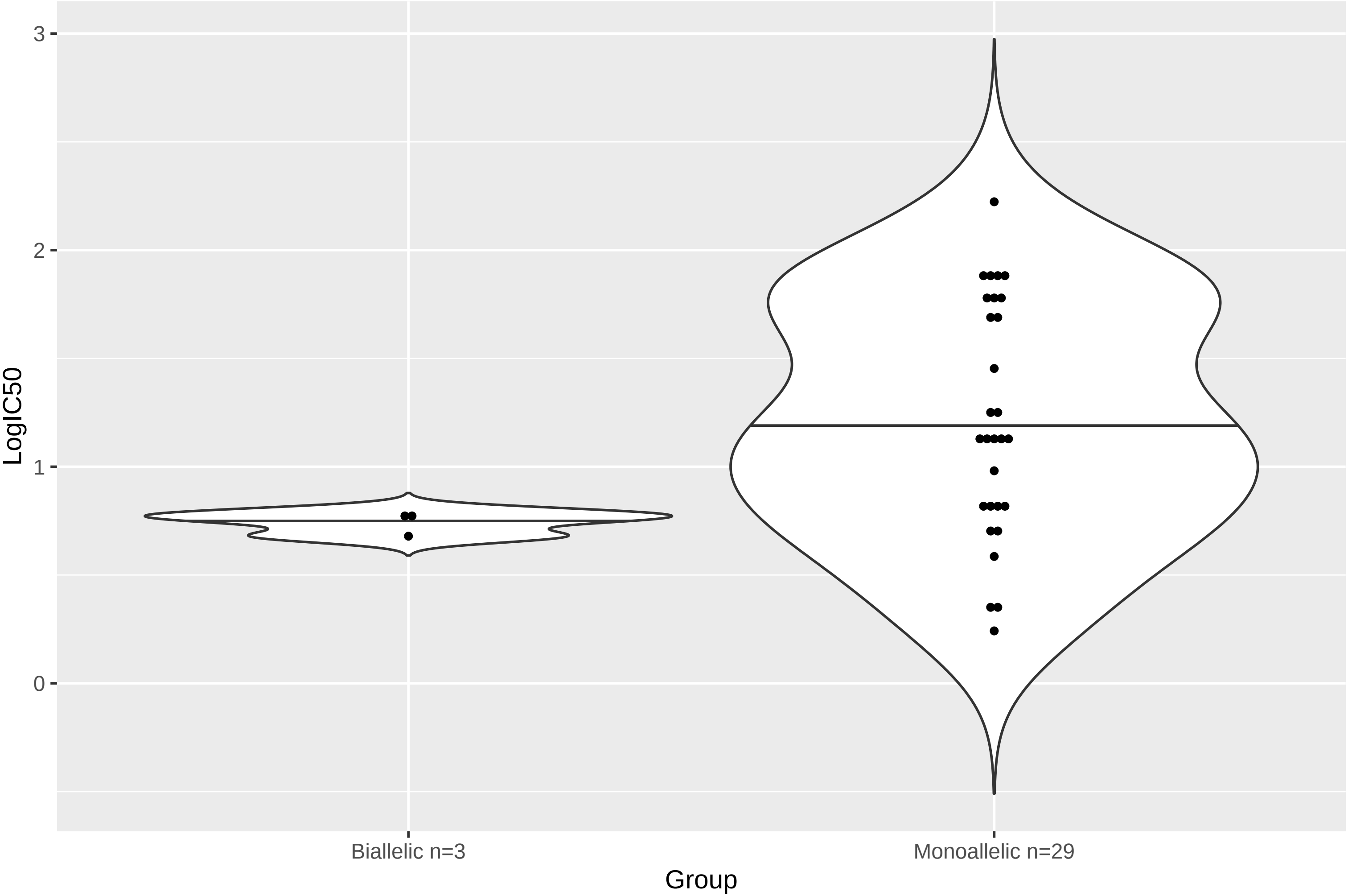

Feature: ENST00000307114.11_1; ENST00000476510.5_1
Gene Name: GTPBP2
Drug Name: WHI−P97

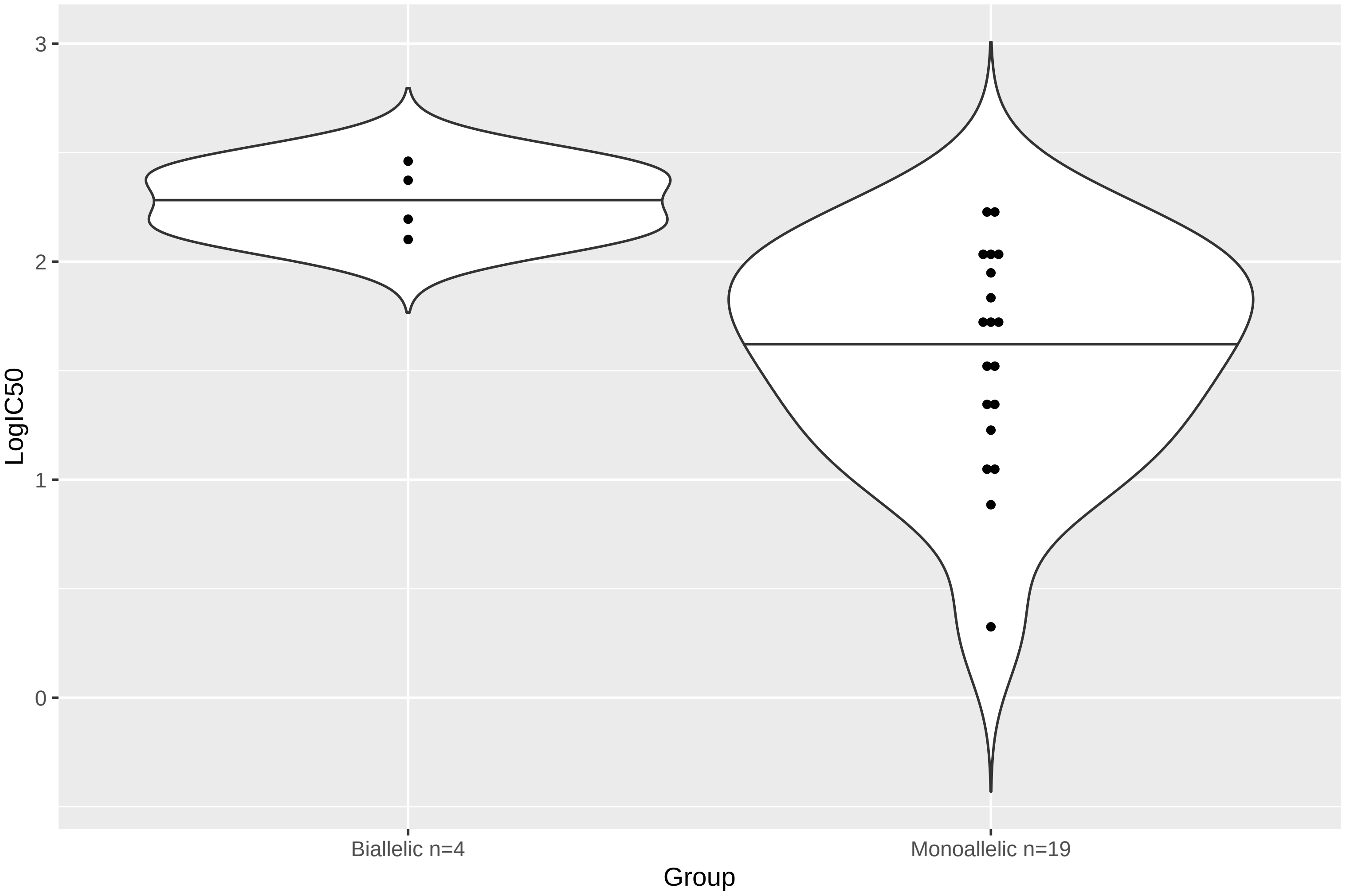

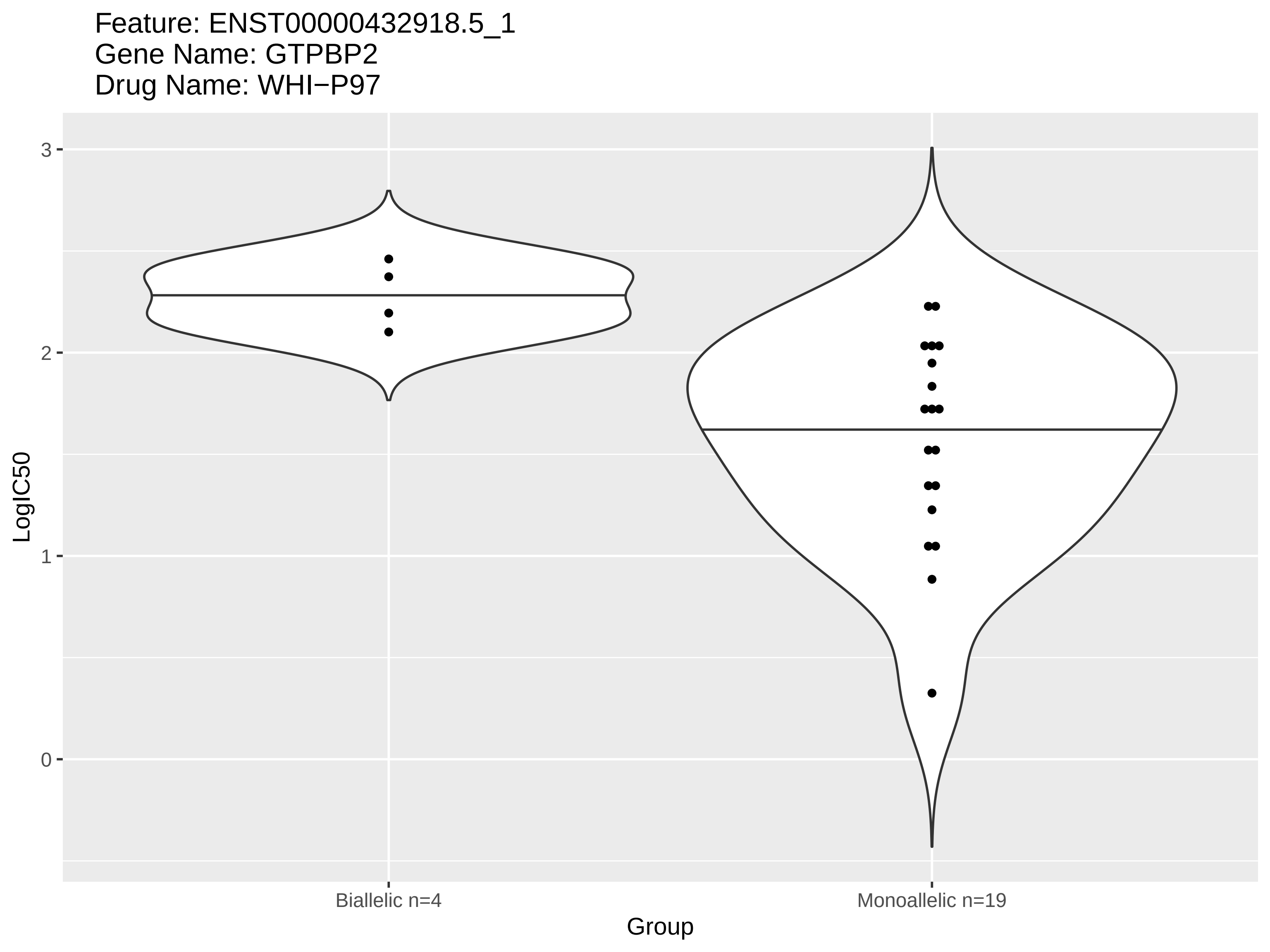

Feature: ENST00000432918.5_1
Gene Name: GTPBP2
Drug Name: WHI−P97
3
2
1
0
LogIC50
Biallelic n=4
Monoallelic n=19
Group

Feature: ENST00000527123.1_1
Gene Name: MAP2K3
Drug Name: TL−2−105

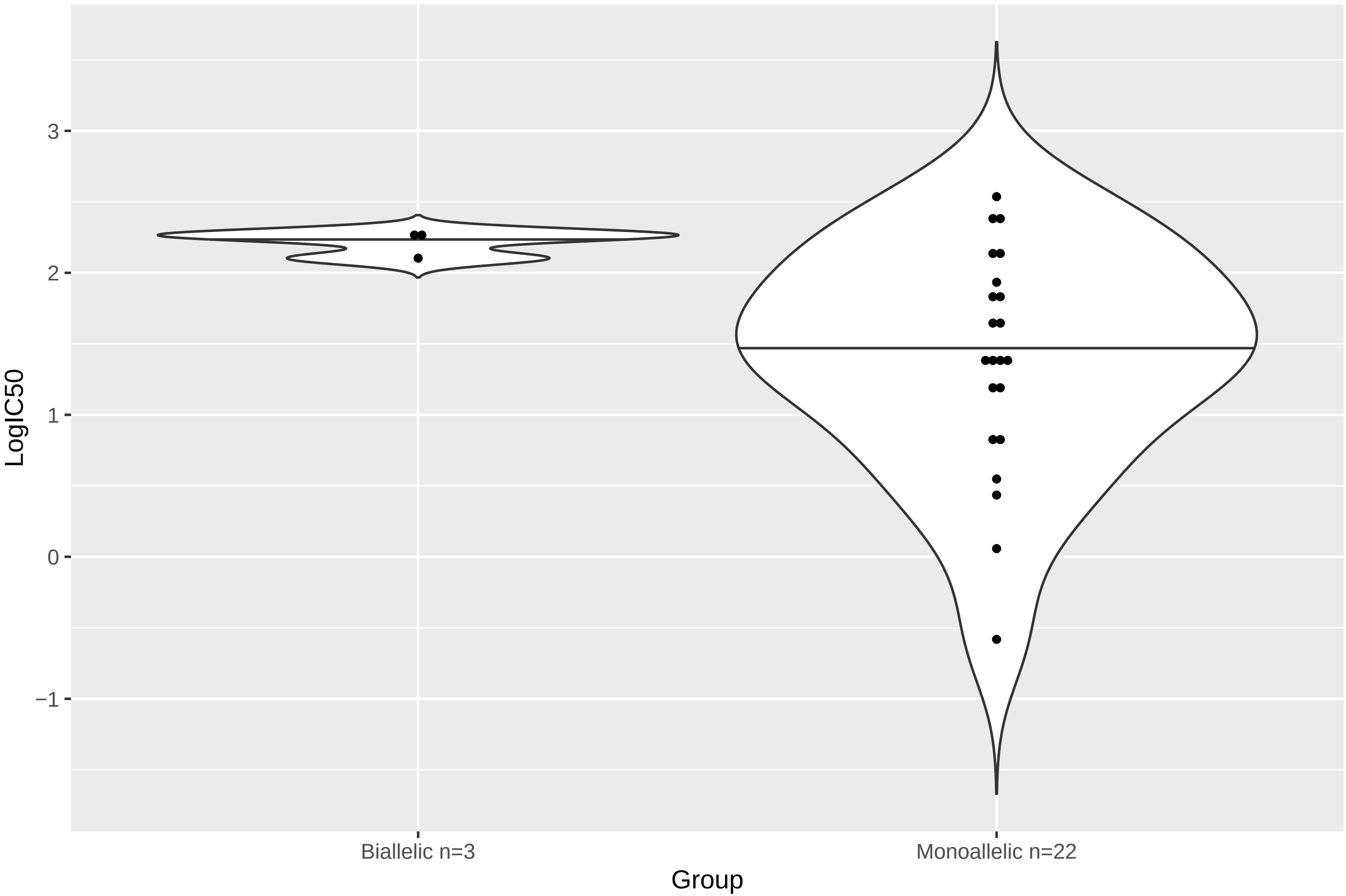

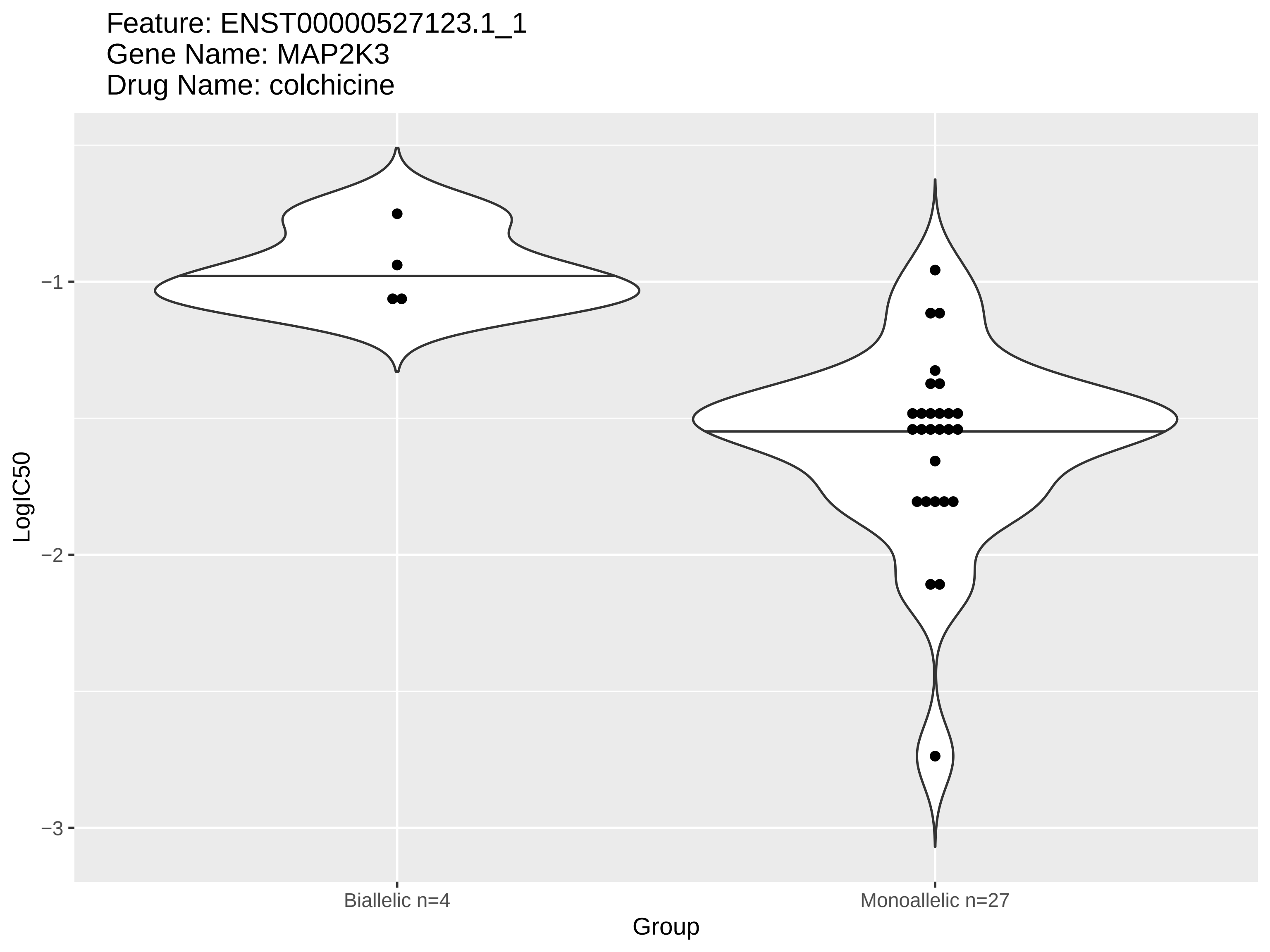

Feature: ENST00000527123.1_1
Gene Name: MAP2K3
Drug Name: colchicine
LogIC50
−1
−2
−3
Biallelic n=4
Monoallelic n=27
Group

Feature: ENST00000533422.5_1
Gene Name: BCLAF1
Drug Name: SB52334

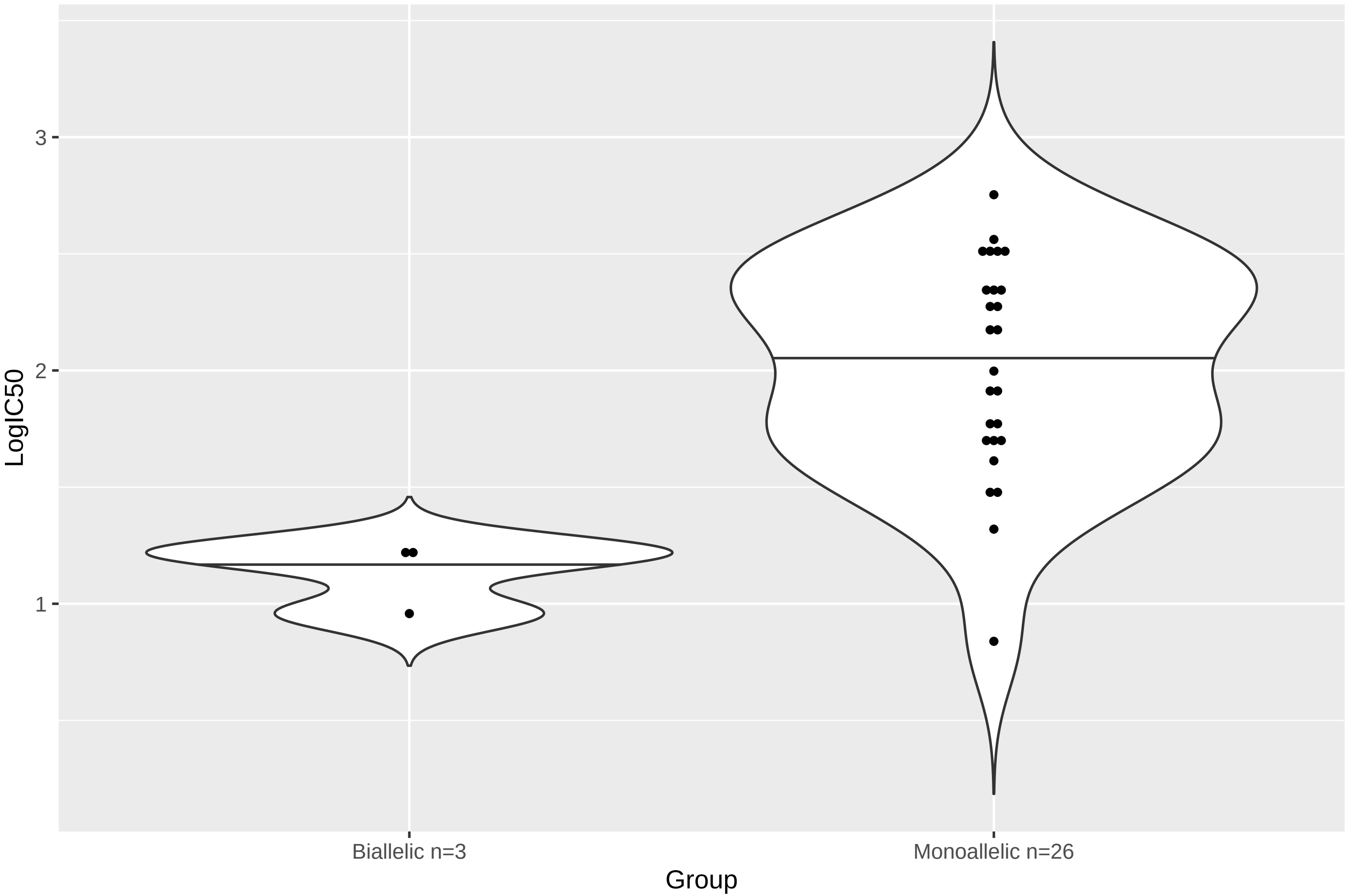

Feature: ENST00000456481.1_1
Gene Name: AC009245.3
Drug Name: tyrphostin−A9

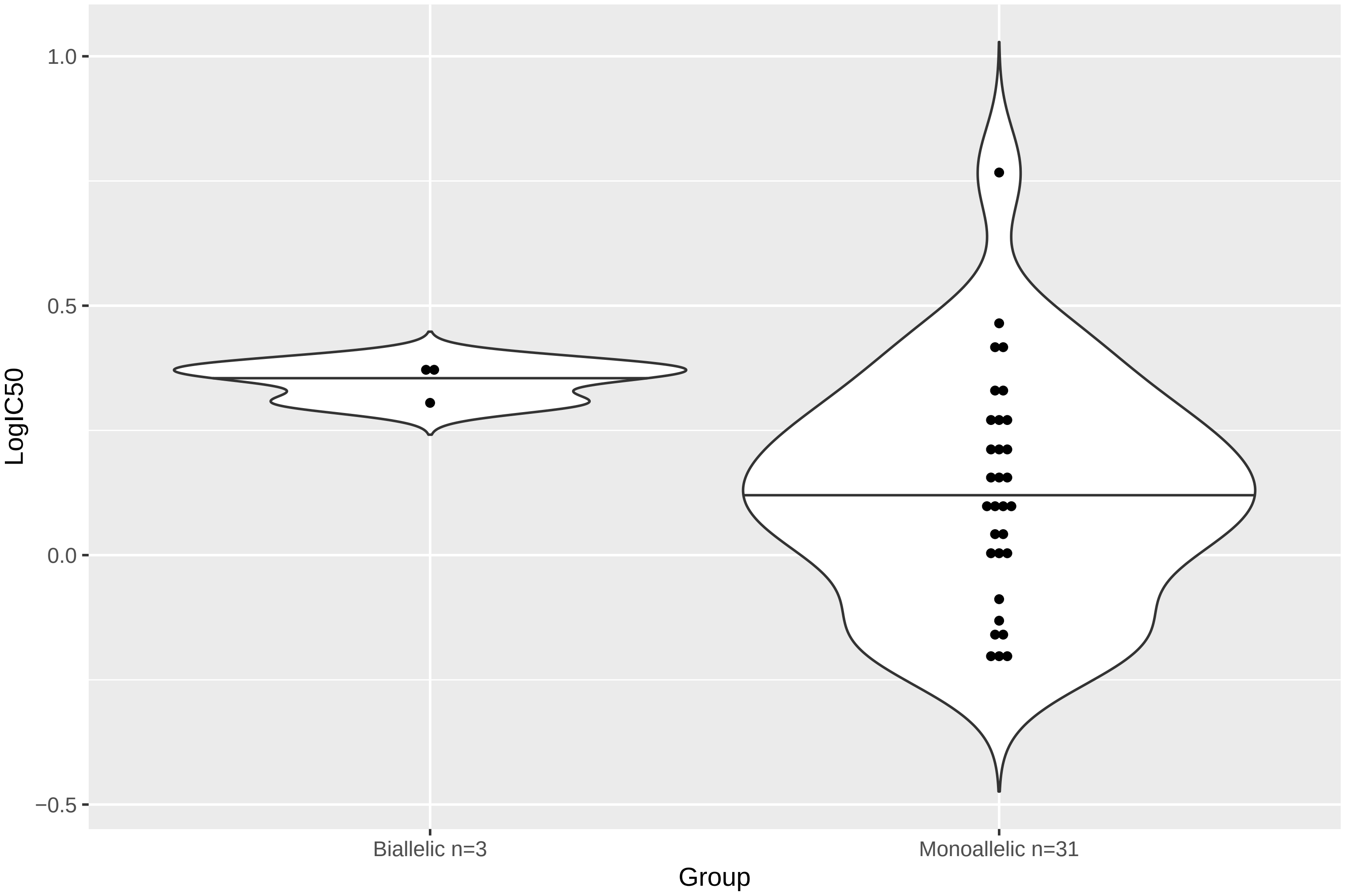

Feature: ENST00000377619.9_1
Gene Name: COMMD6
Drug Name: pardoprunox

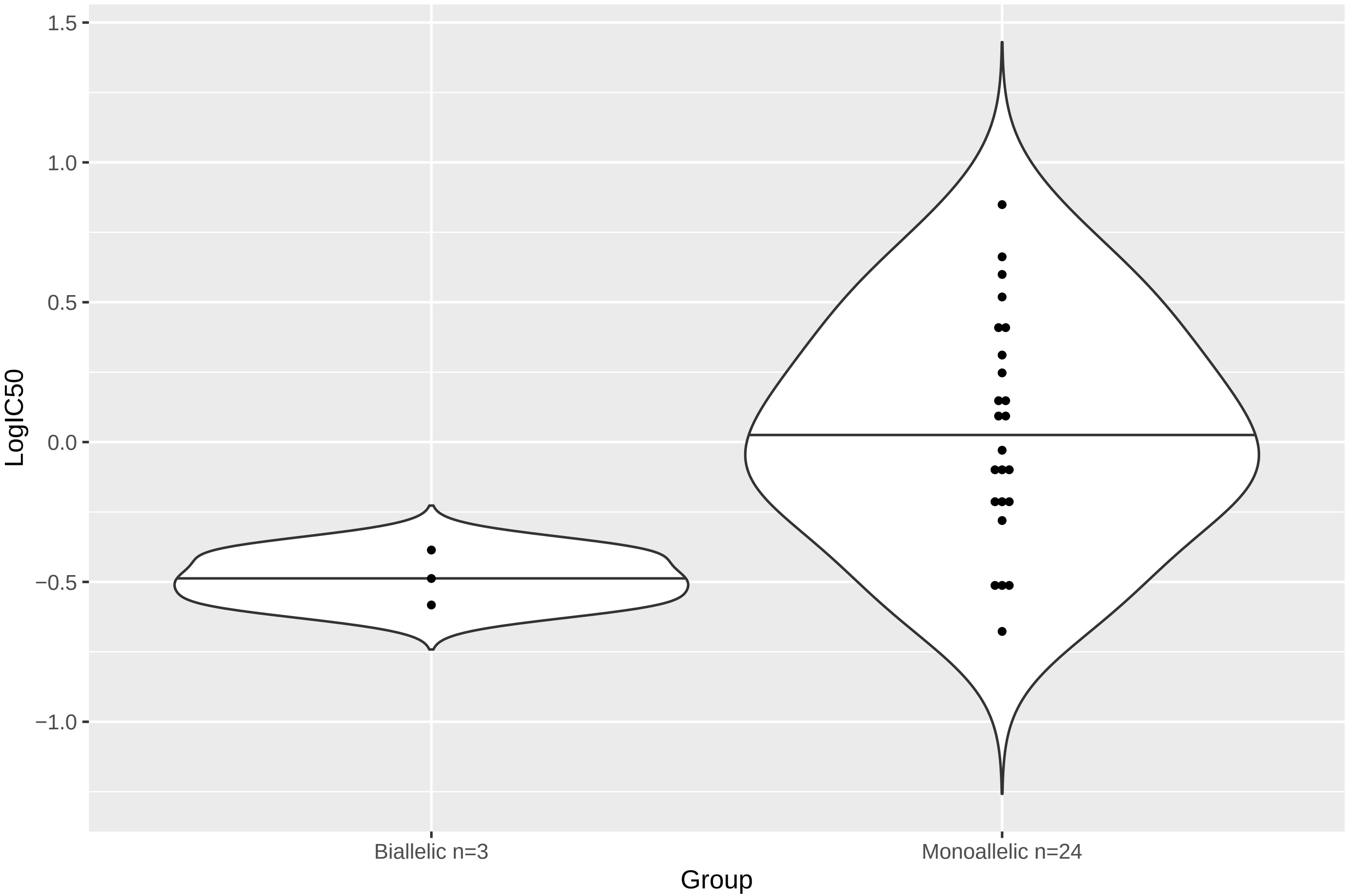

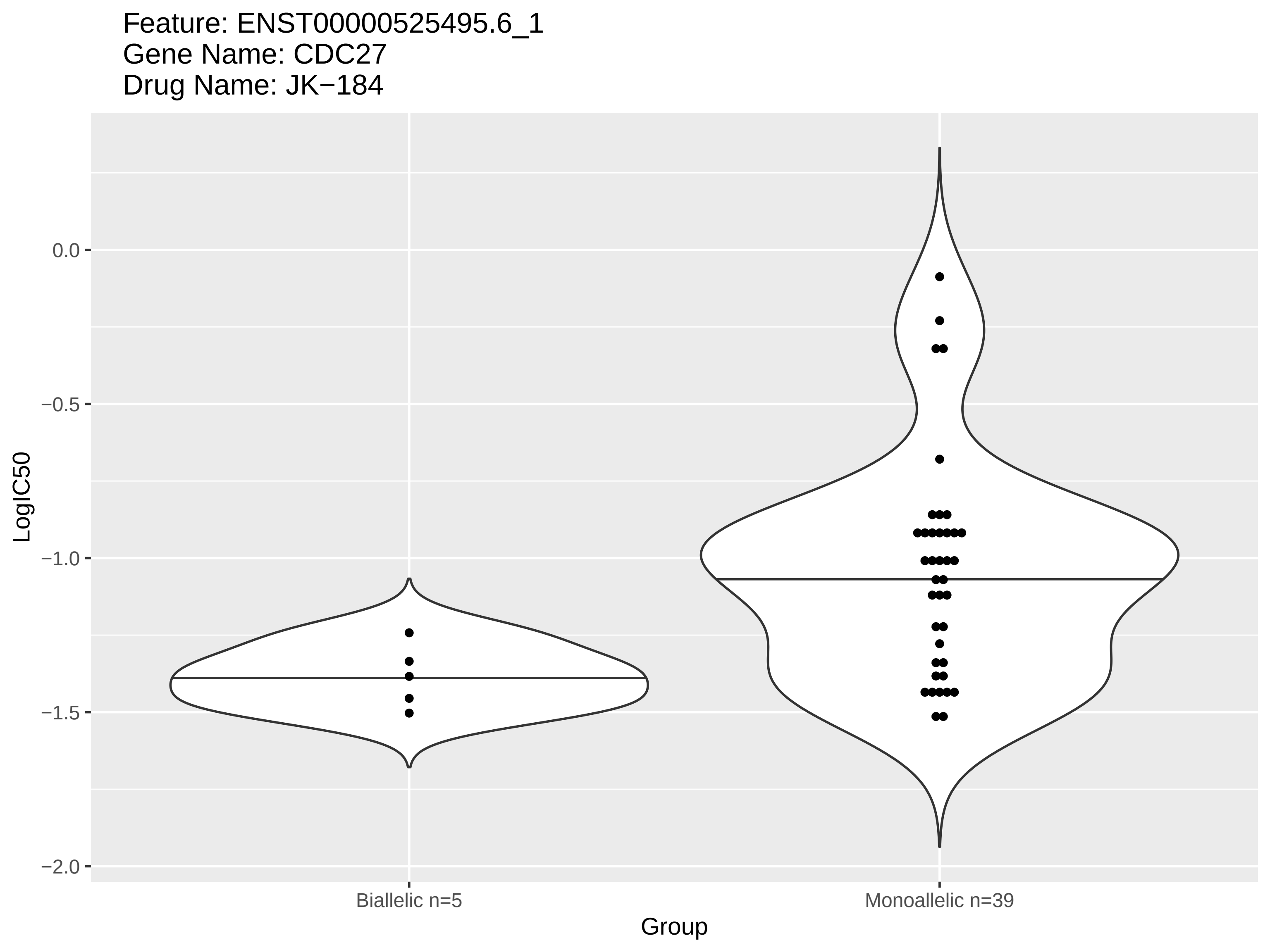
Feature: ENST00000525495.6_1
Gene Name: CDC27
Drug Name: JK−184
LogIC50
0.0
−0.5
−1.0
−1.5
−2.0
Biallelic n=5
Monoallelic n=39
Group

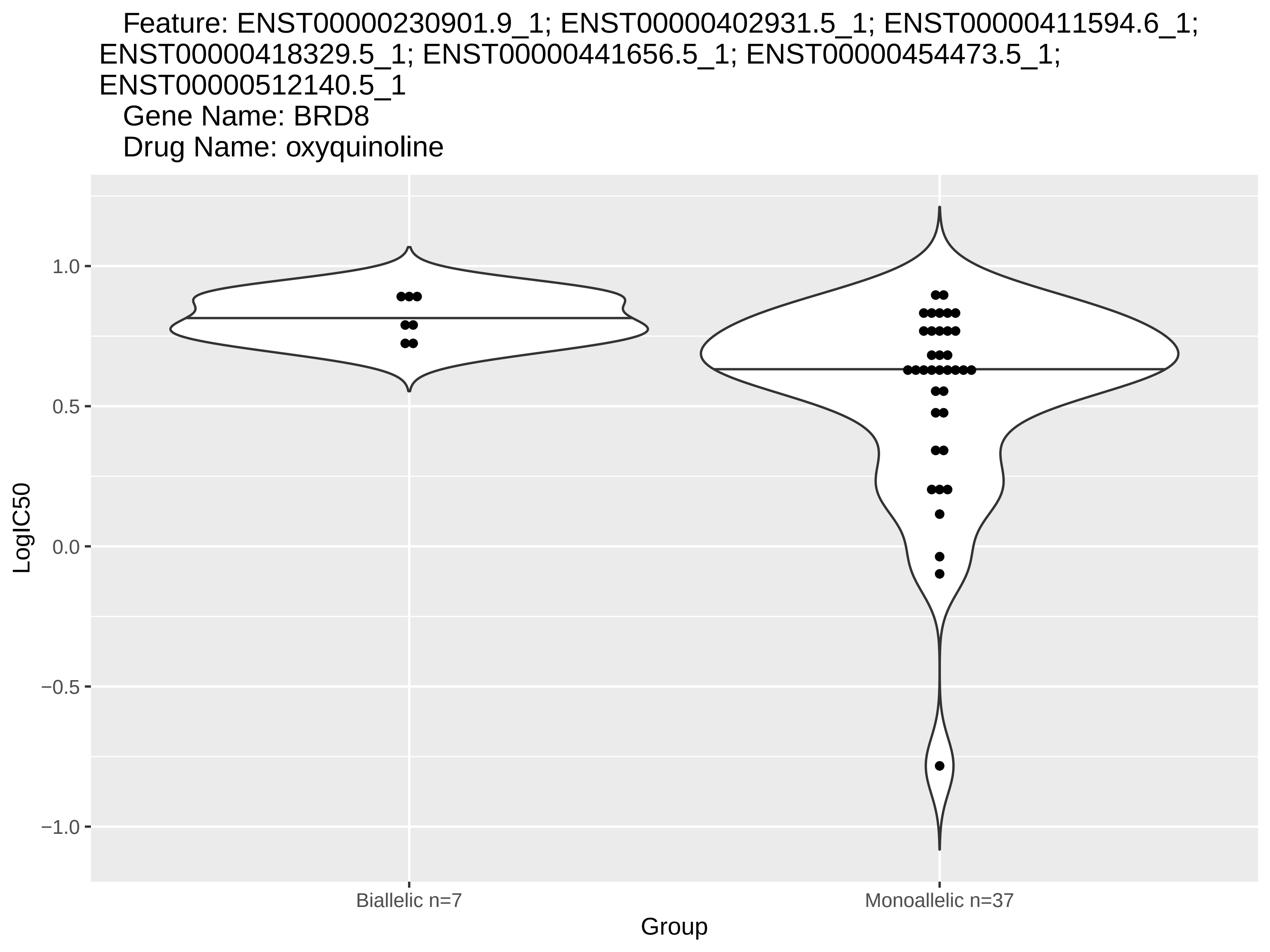

Feature: ENST00000230901.9_1; ENST00000402931.5_1; ENST00000411594.6_1; ENST00000418329.5_1; ENST00000441656.5_1; ENST00000454473.5_1; ENST00000512140.5_1
Gene Name: BRD8
Drug Name: oxyquinoline
LogIC50
1.0
0.5
0.0
−0.5
−1.0
Biallelic n=7
Monoallelic n=37
Group

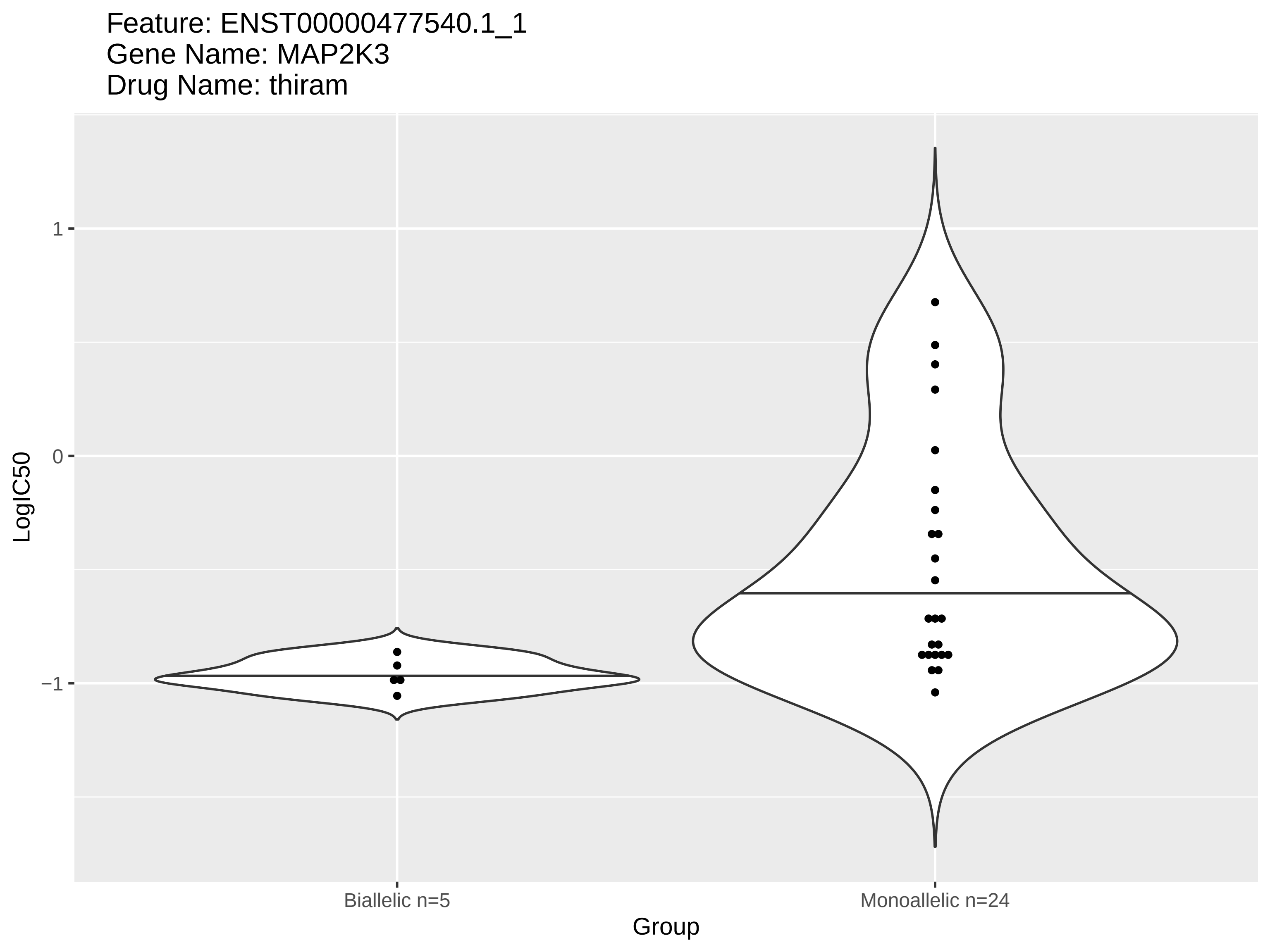

Feature: ENST00000477540.1_1
Gene Name: MAP2K3
Drug Name: thiram
1
0
−1
LogIC50
Biallelic n=5
Monoallelic n=24
Group

Feature: ENST00000575483.5_1
Gene Name: CDC27
Drug Name: LY456236

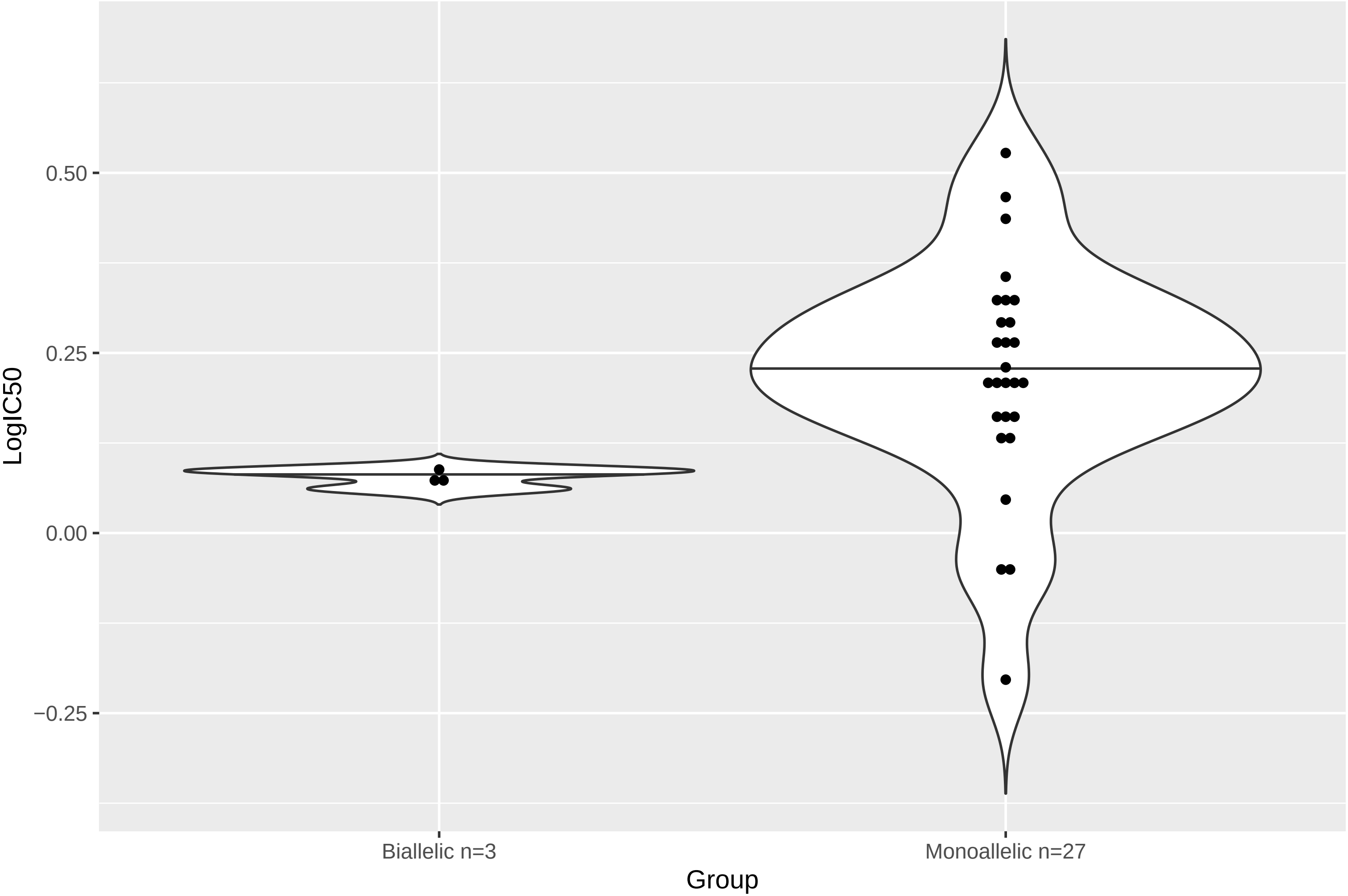

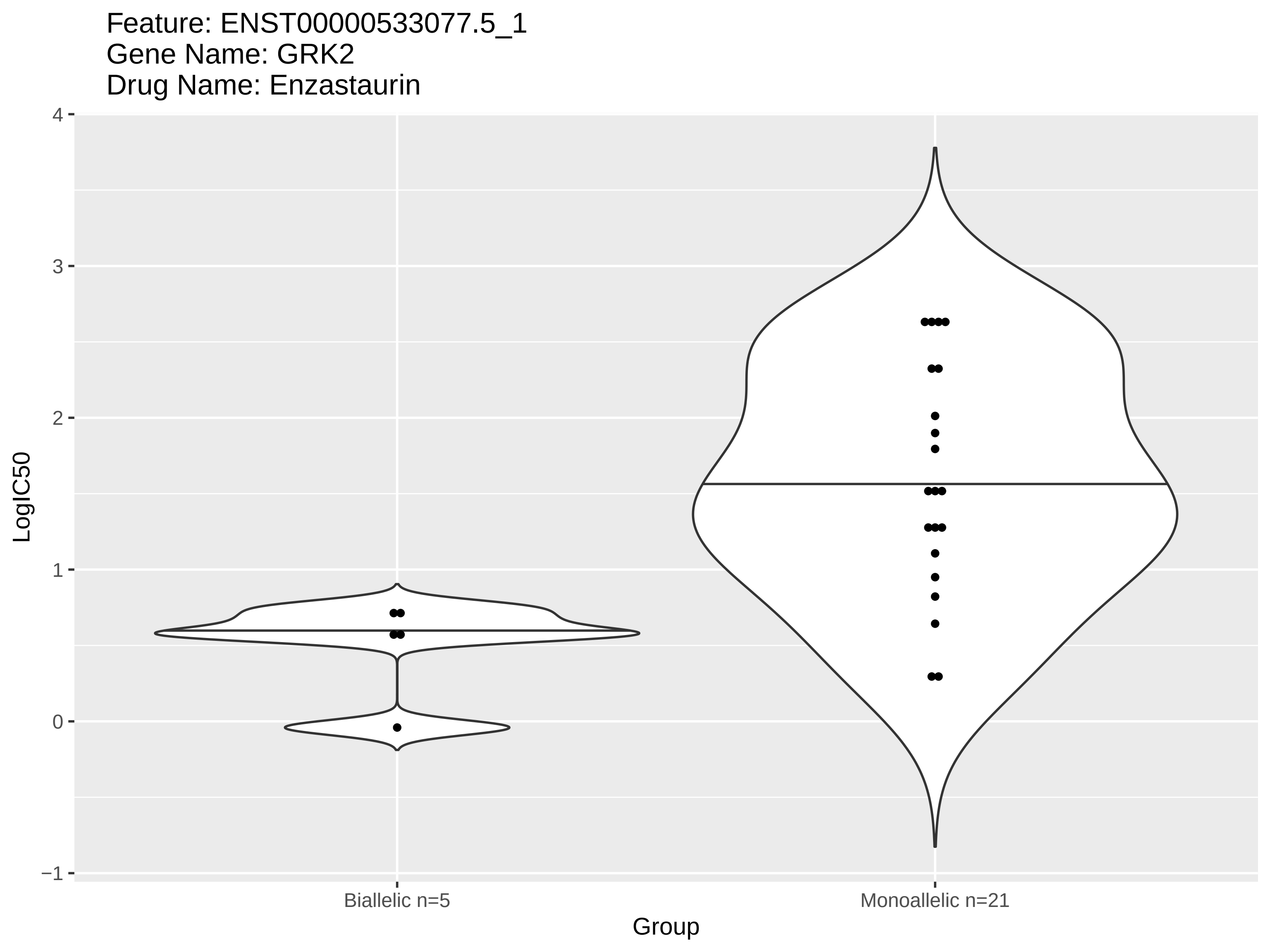
Feature: ENST00000533077.5_1
Gene Name: GRK2
Drug Name: Enzastaurin
LogIC50
4
3
2
1
0
−1
Biallelic n=5
Monoallelic n=21
Group

Feature: ENST00000377619.9_1
Gene Name: COMMD6
Drug Name: I−BET−762

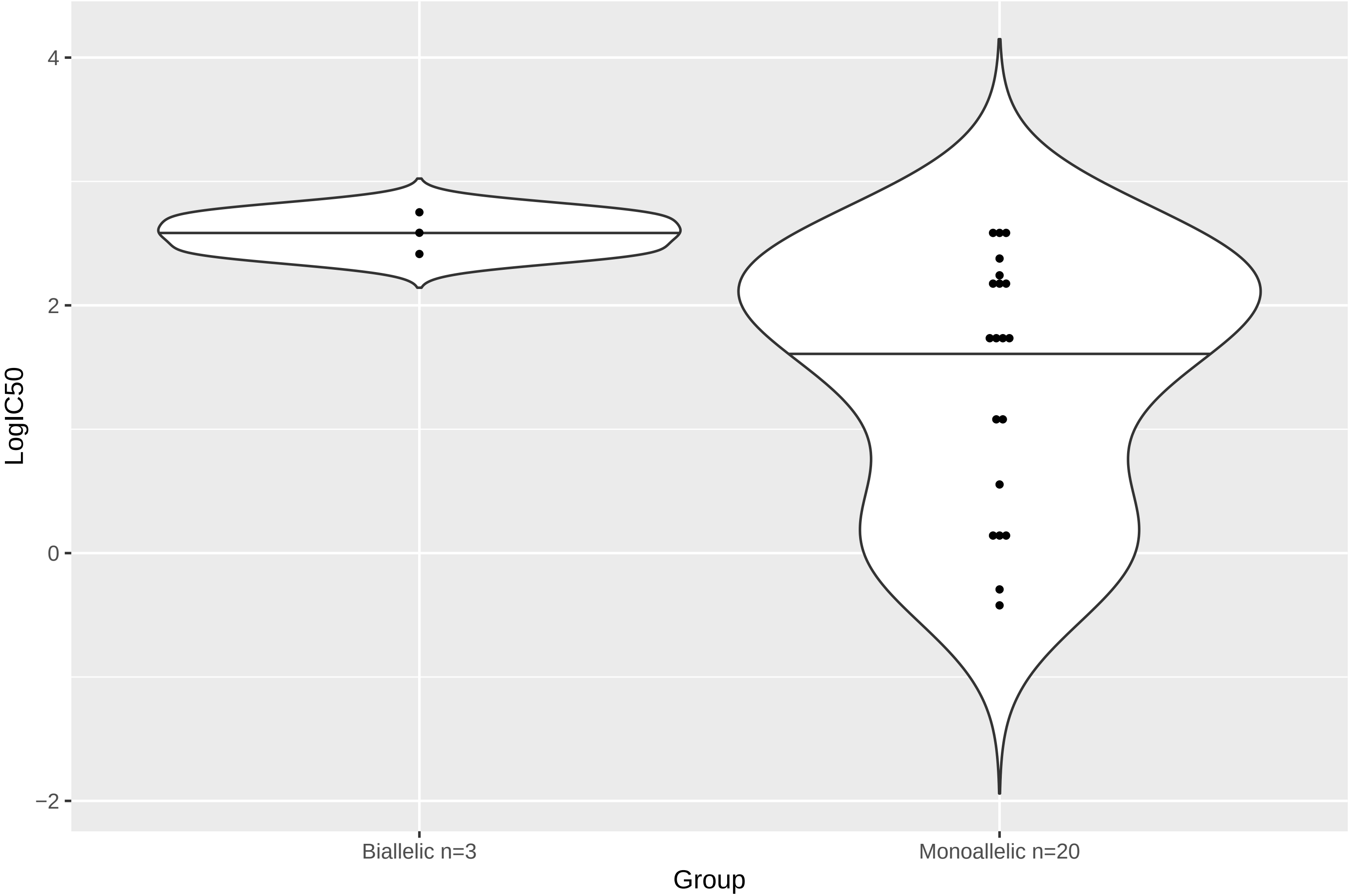

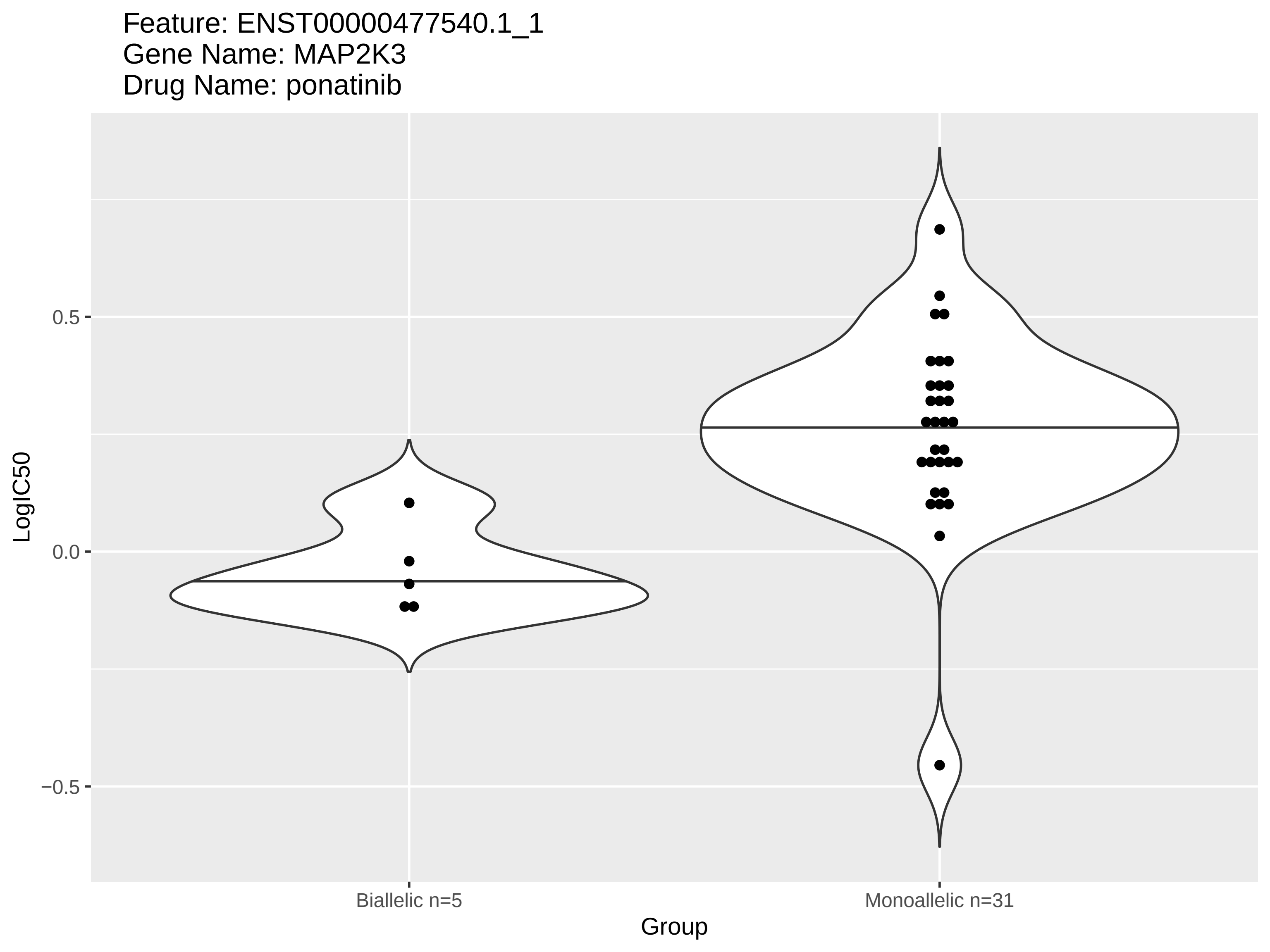
Feature: ENST00000477540.1_1
Gene Name: MAP2K3
Drug Name: ponatinib
0.5
0.0
−0.5
LogIC50
Biallelic n=5
Monoallelic n=31
Group

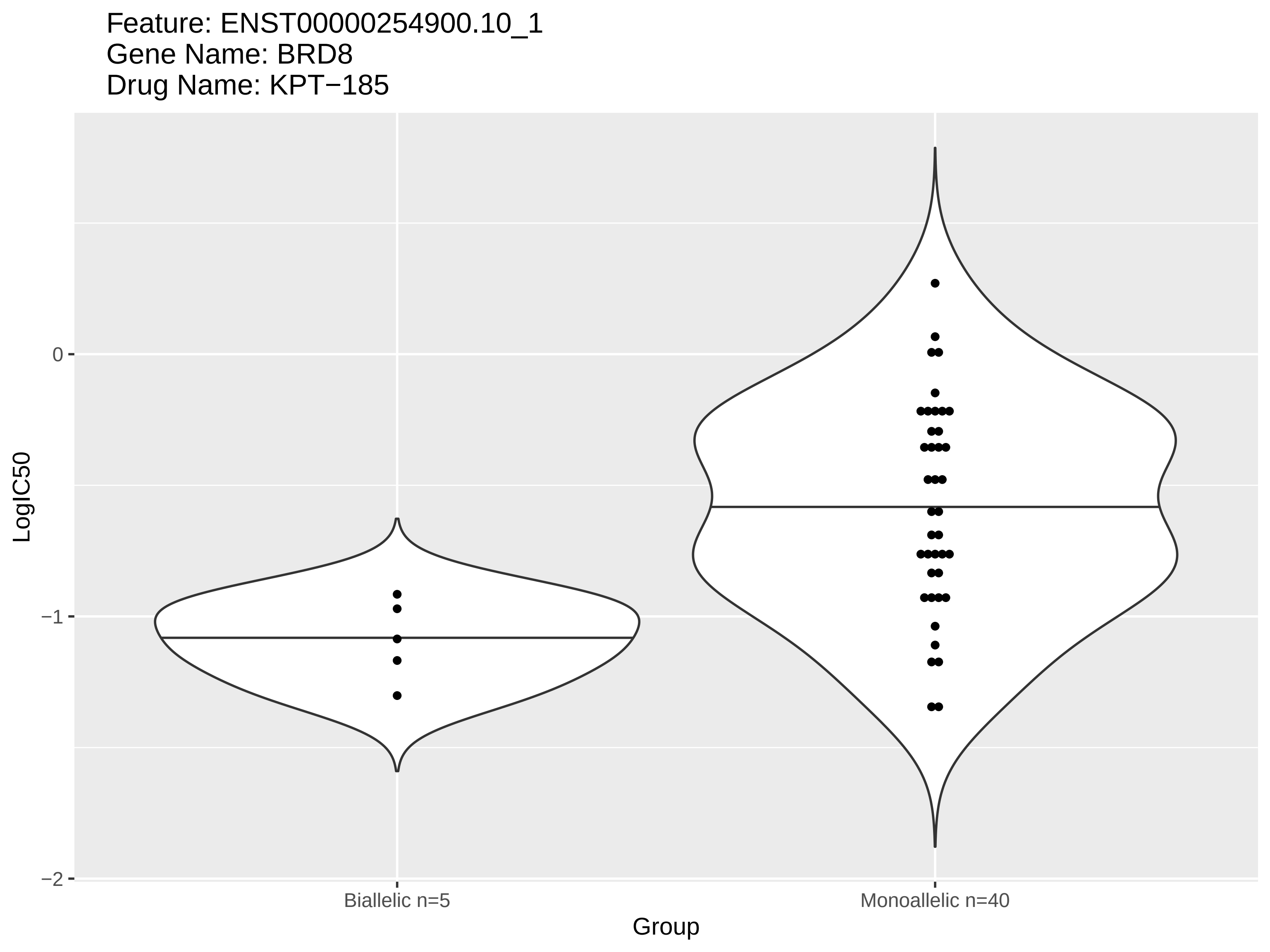
Feature: ENST00000254900.10_1
Gene Name: BRD8
Drug Name: KPT−185
LogIC50
0
−1
−2
Biallelic n=5
Monoallelic n=40
Group

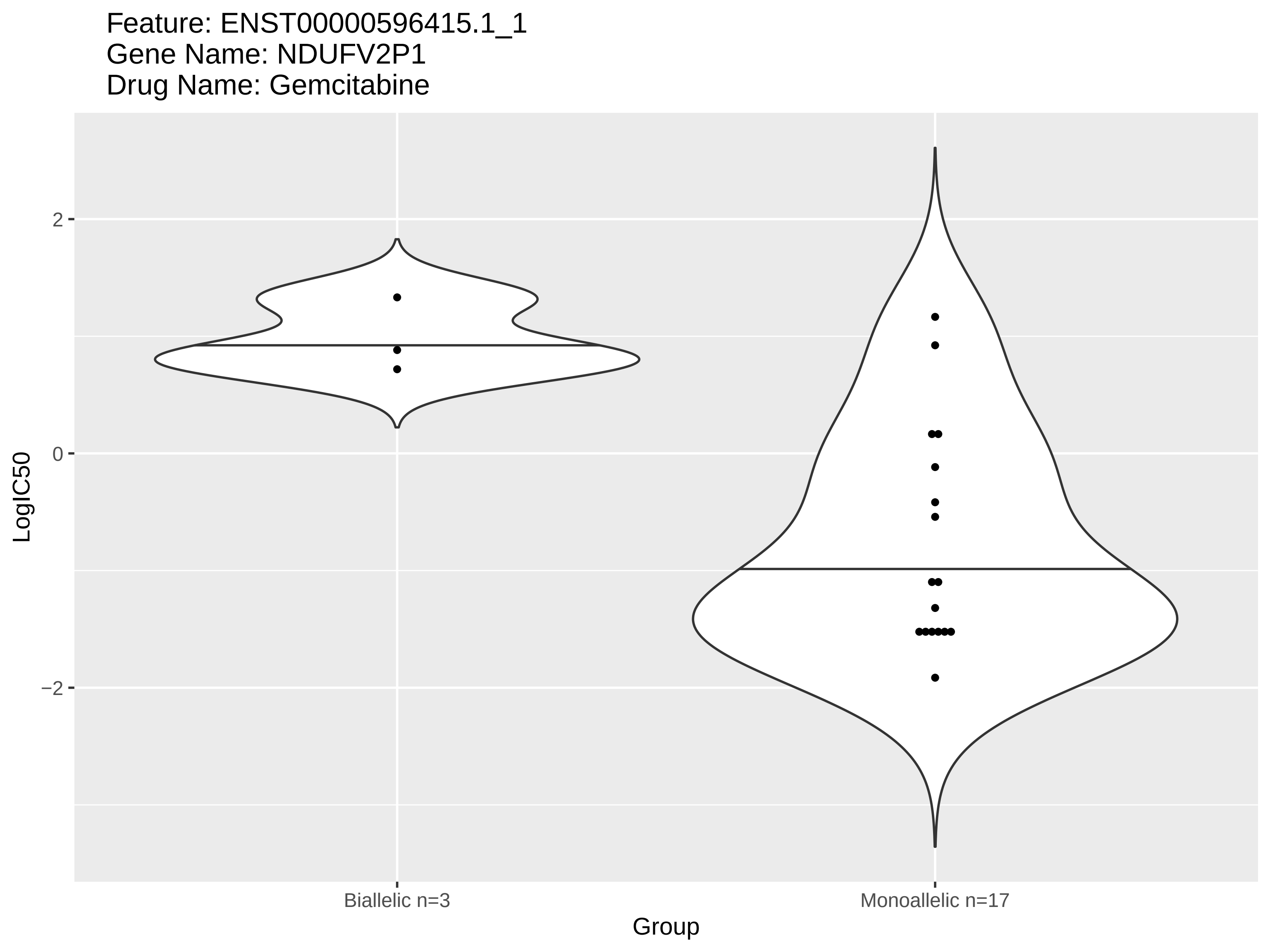
Feature: ENST00000596415.1_1
Gene Name: NDUFV2P1
Drug Name: Gemcitabine
2
0
−2
LogIC50
Biallelic n=3
Monoallelic n=17
Group

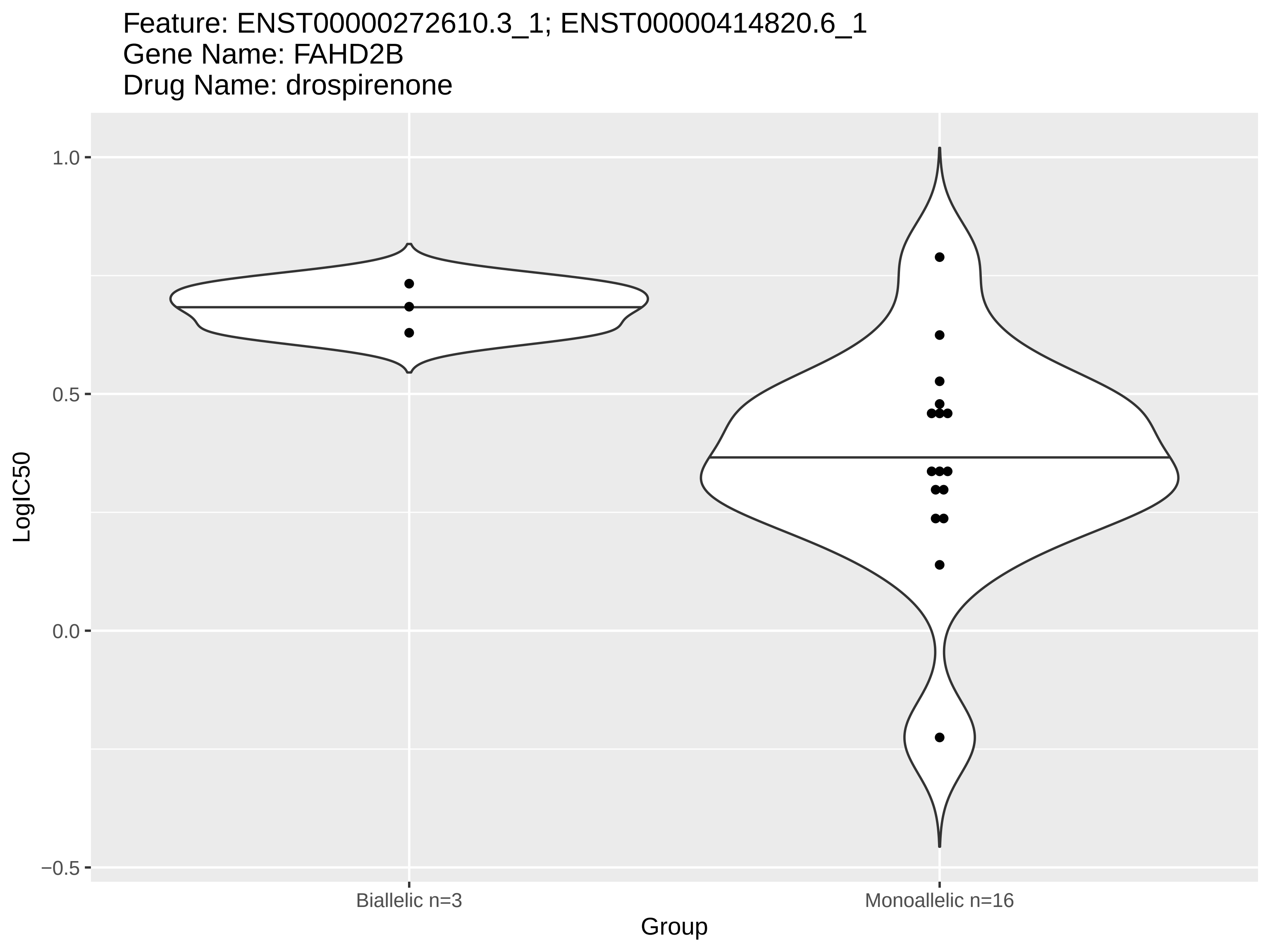
Feature: ENST00000272610.3_1; ENST00000414820.6_1
Gene Name: FAHD2B
Drug Name: drospirenone
1.0
0.5
0.0
−0.5
LogIC50
Biallelic n=3
Monoallelic n=16
Group

Feature: ENST00000534269.5_1
Gene Name: BCLAF1
Drug Name: azilsartan

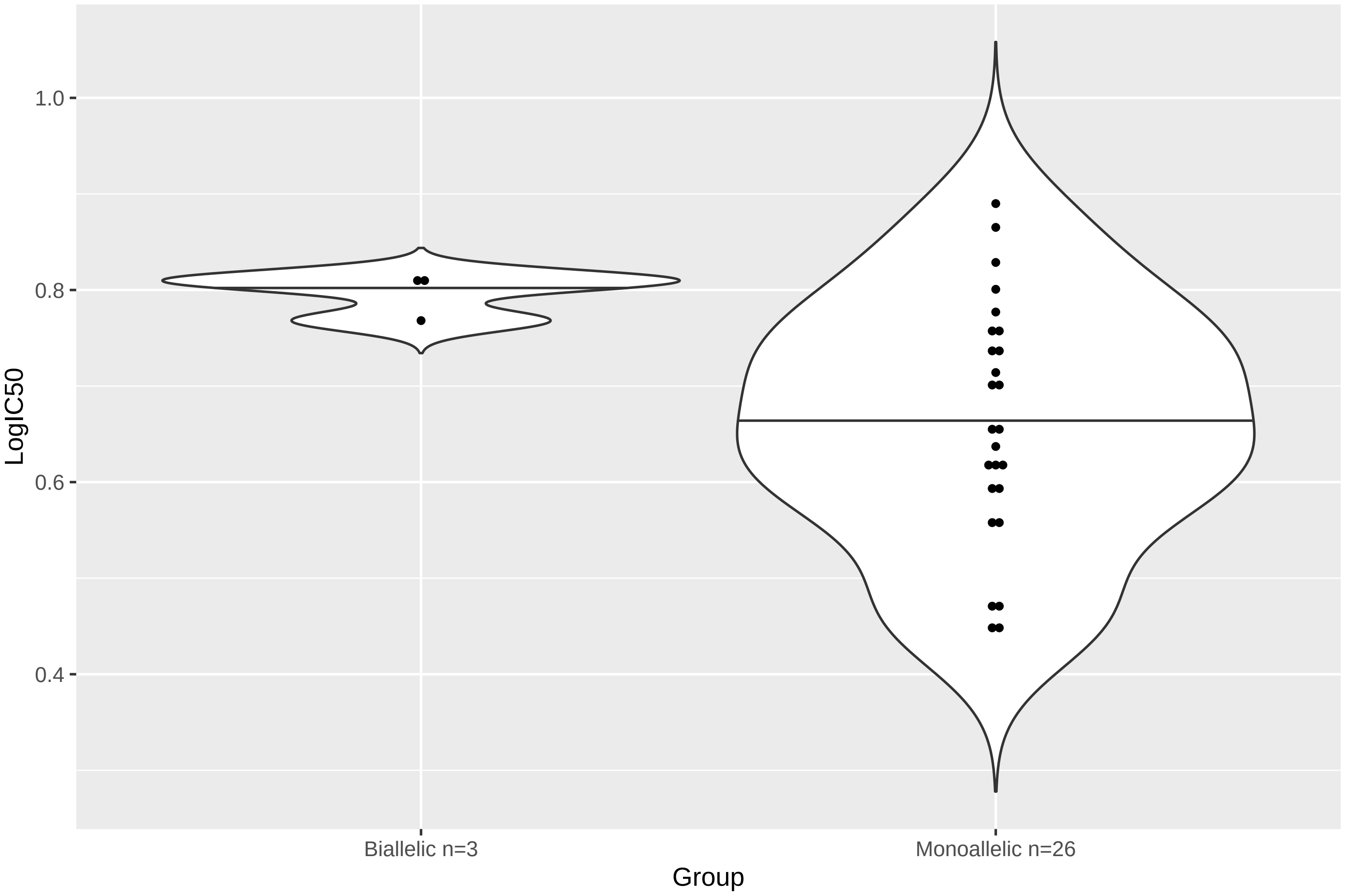

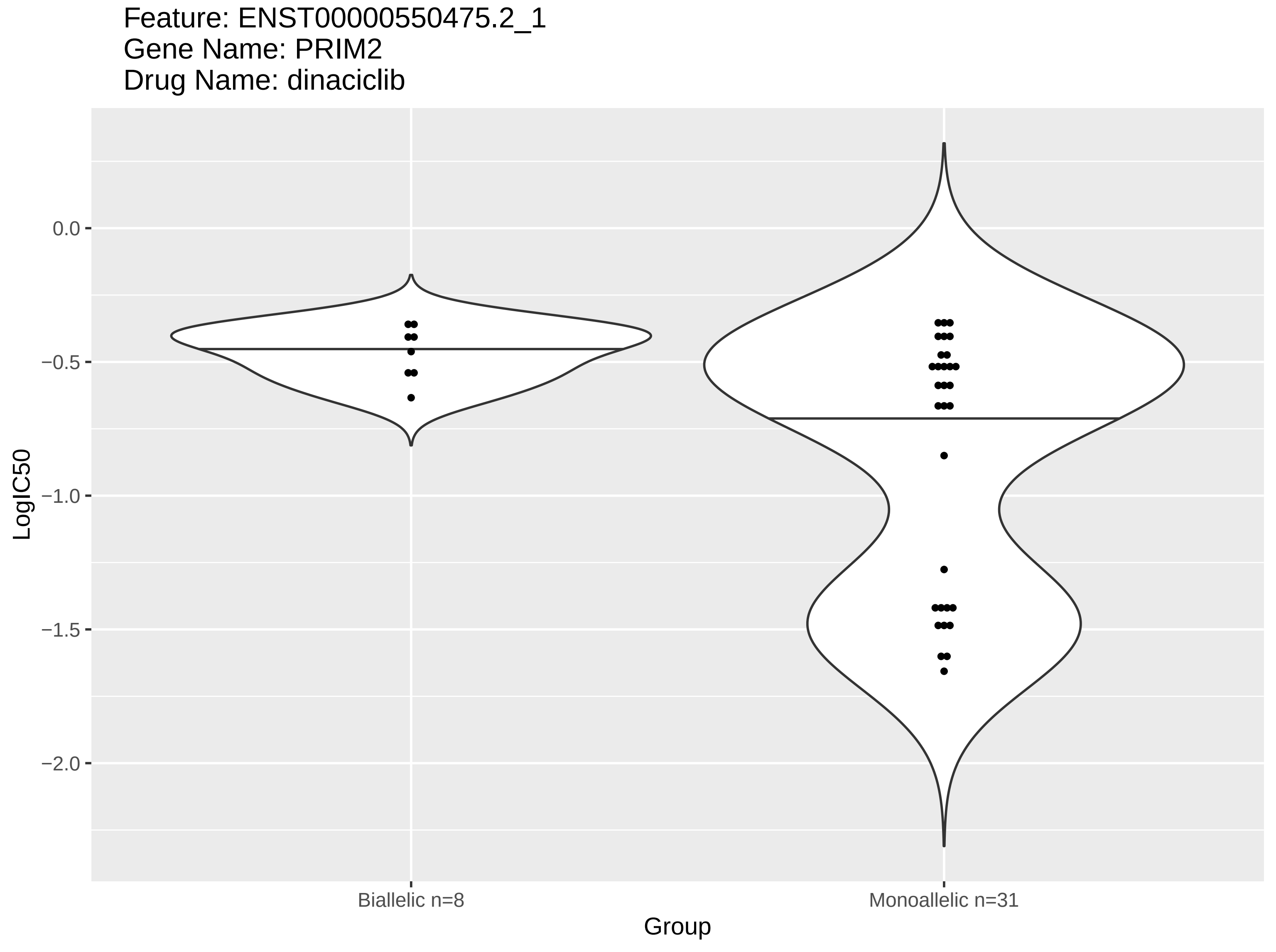
Feature: ENST00000550475.2_1
Gene Name: PRIM2
Drug Name: dinaciclib
0.0
−0.5
−1.0
−1.5
−2.0
LogIC50
Biallelic n=8
Monoallelic n=31
Group

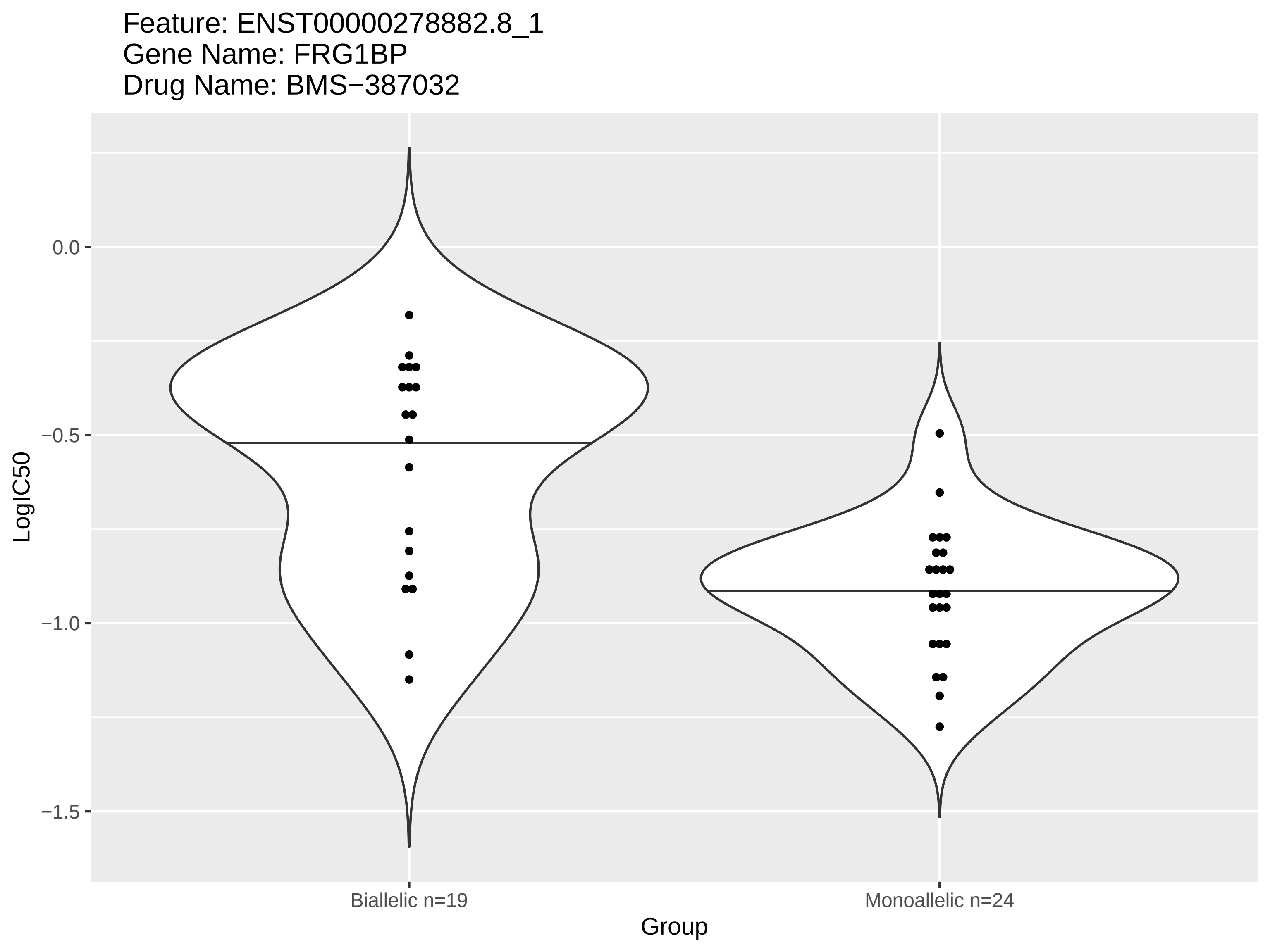
Feature: ENST00000278882.8_1
Gene Name: FRG1BP
Drug Name: BMS−387032
0.0
−0.5
−1.0
−1.5
LogIC50
Biallelic n=19
Monoallelic n=24
Group

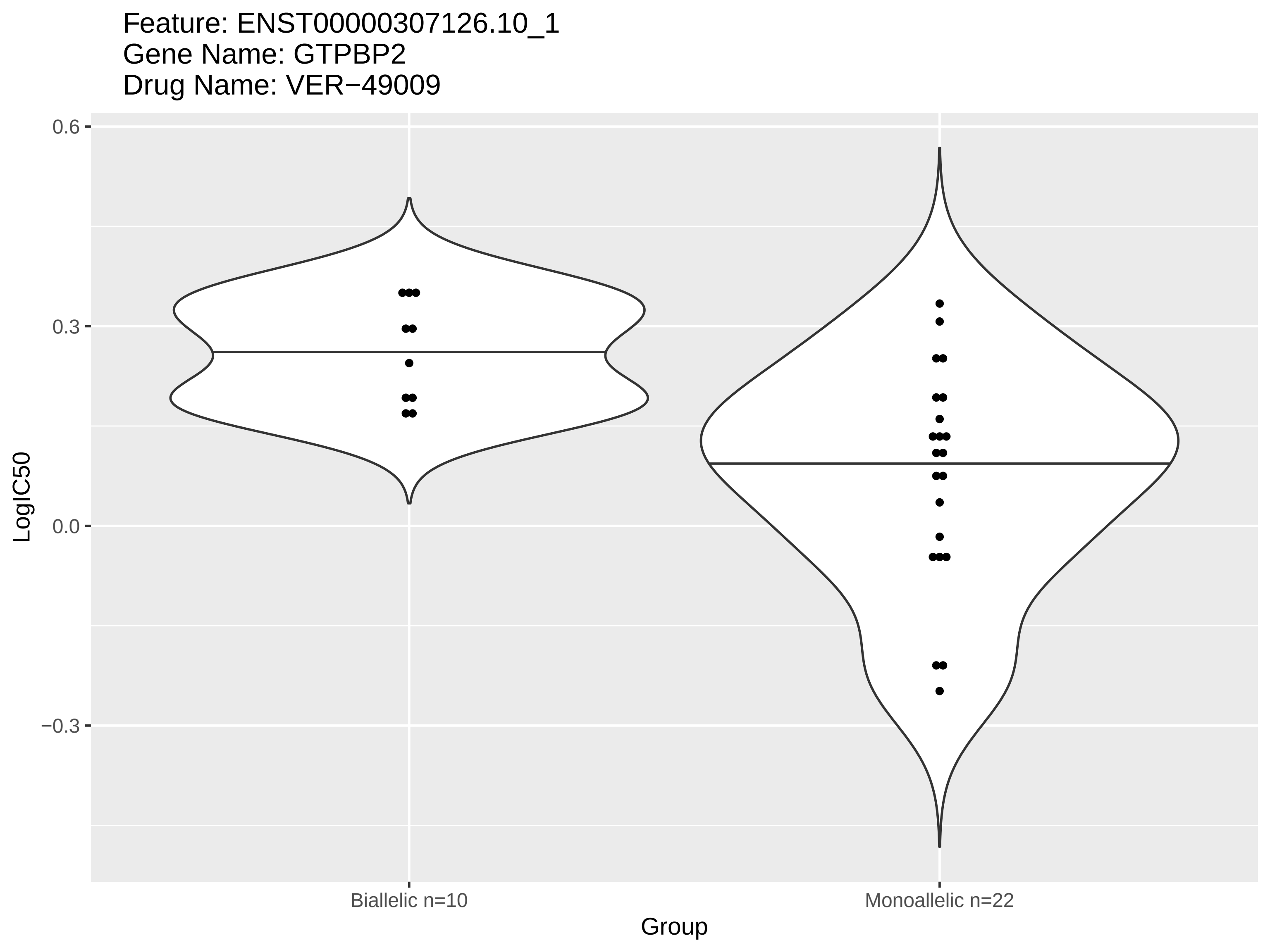
Feature: ENST00000307126.10_1
Gene Name: GTPBP2
Drug Name: VER−49009
0.6
0.3
0.0
−0.3
LogIC50
Biallelic n=10
Monoallelic n=22
Group

Feature: ENST00000443090.1_1
Gene Name: GCSHP5
Drug Name: PRIMA−1MET

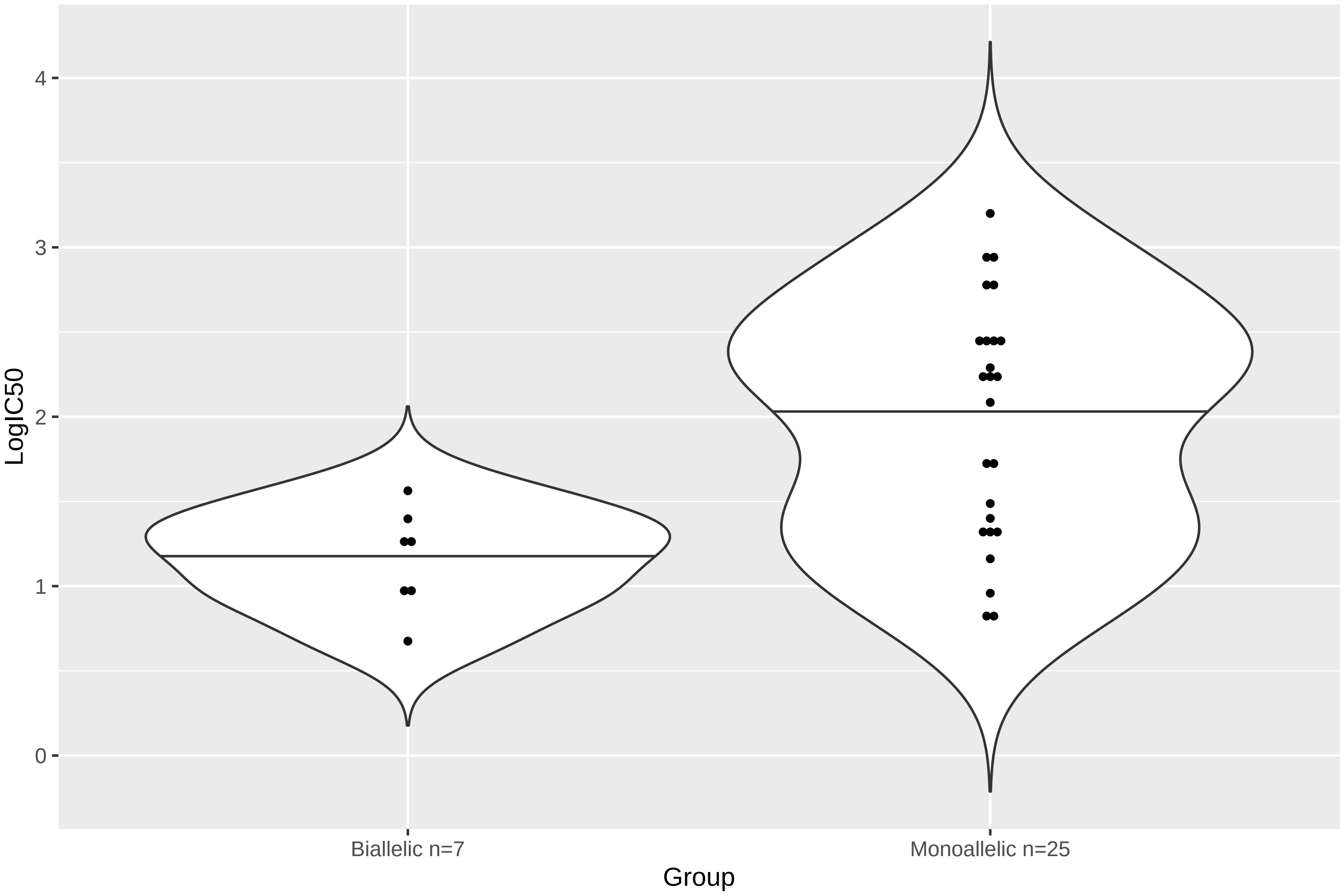

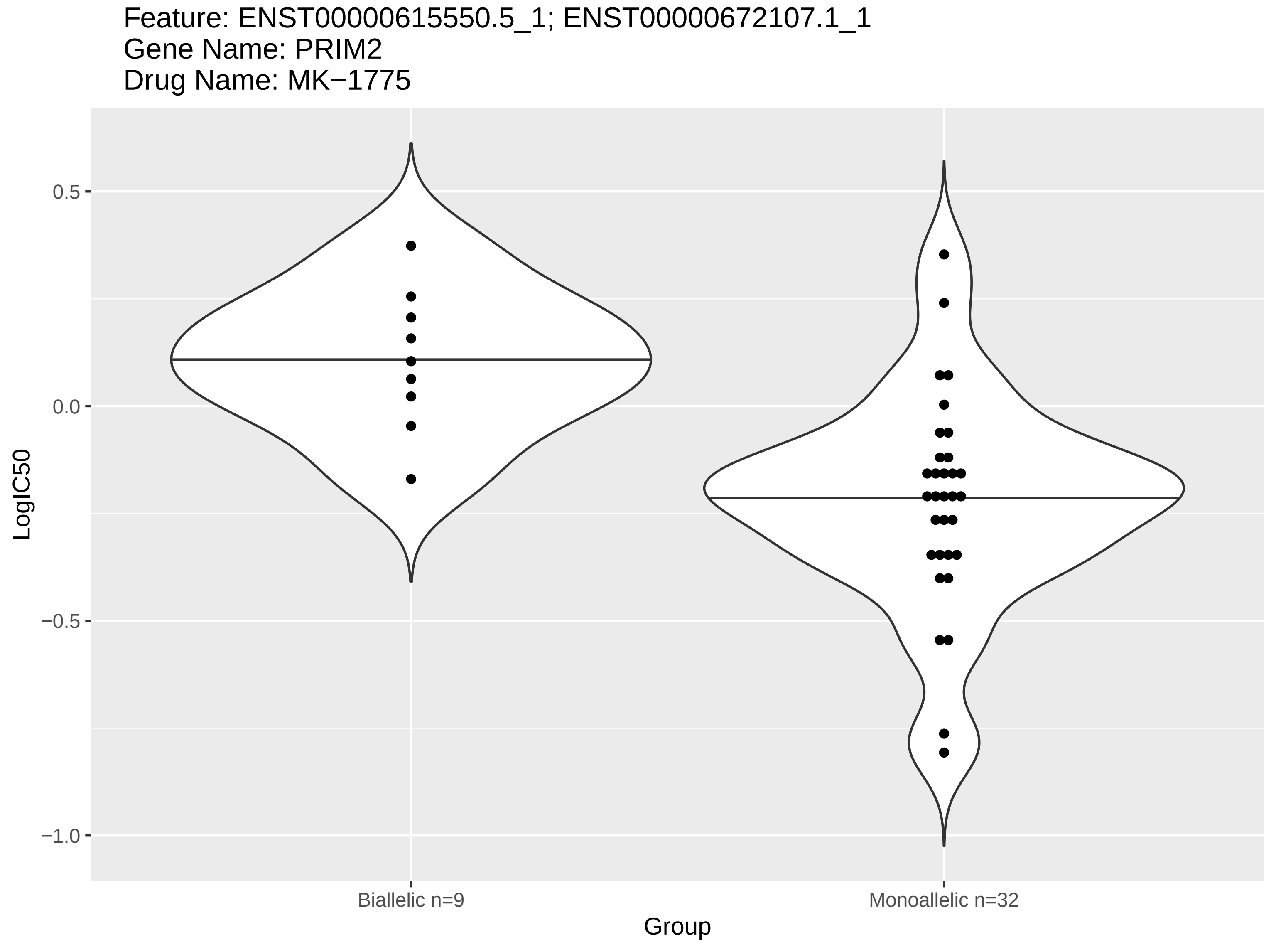

Feature: ENST00000615550.5_1; ENST00000672107.1_1
Gene Name: PRIM2
Drug Name: MK−1775
0.5
0.0
−0.5
−1.0
LogIC50
Biallelic n=9
Monoallelic n=32
Group

Feature: ENST00000530767.5_1
Gene Name: BCLAF1
Drug Name: Ro−4987655

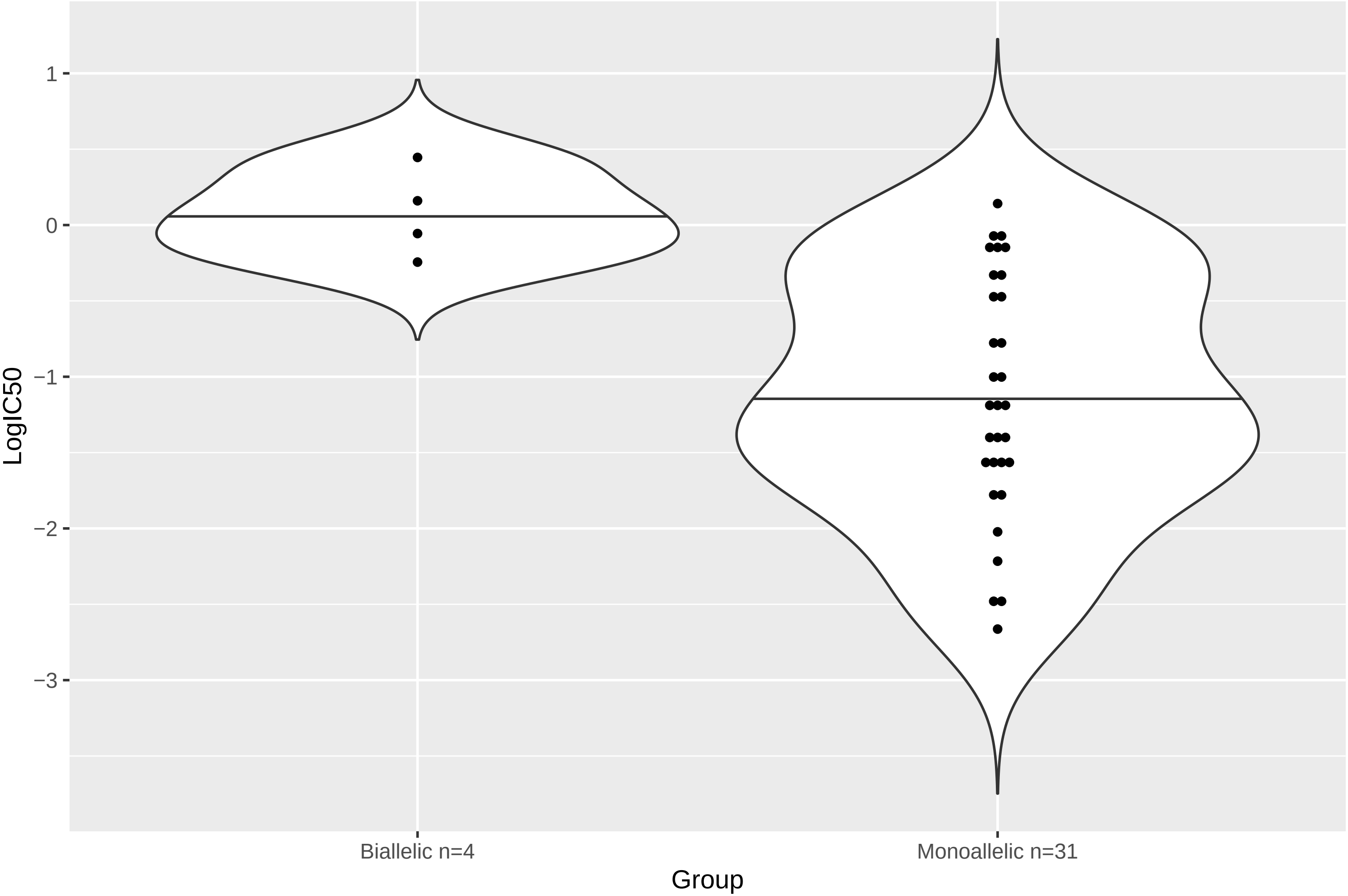

Feature: ENST00000534269.5_1
Gene Name: BCLAF1
Drug Name: Ro−4987655

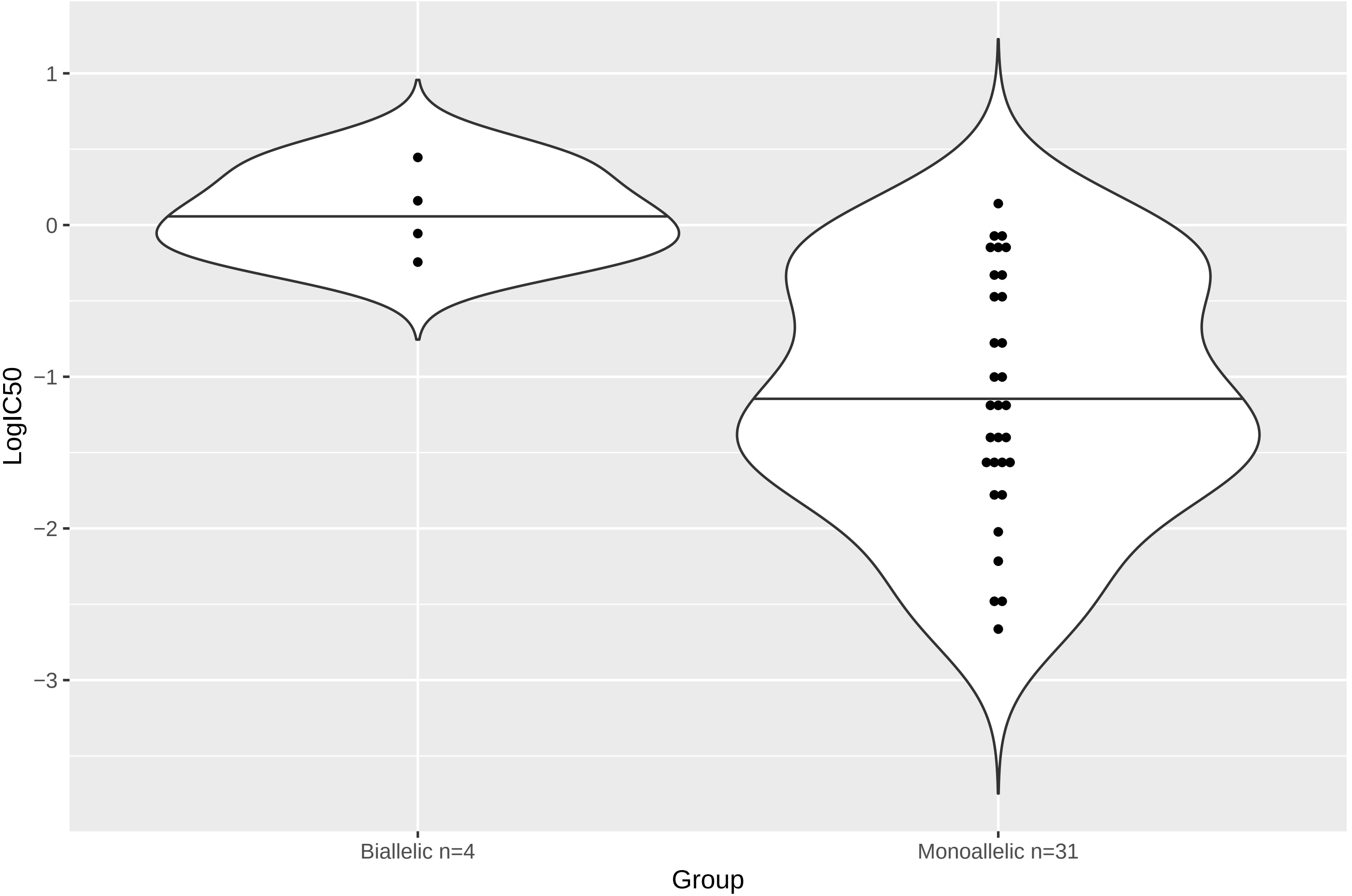

Feature: ENST00000377619.9_1
Gene Name: COMMD6
Drug Name: Y−39983

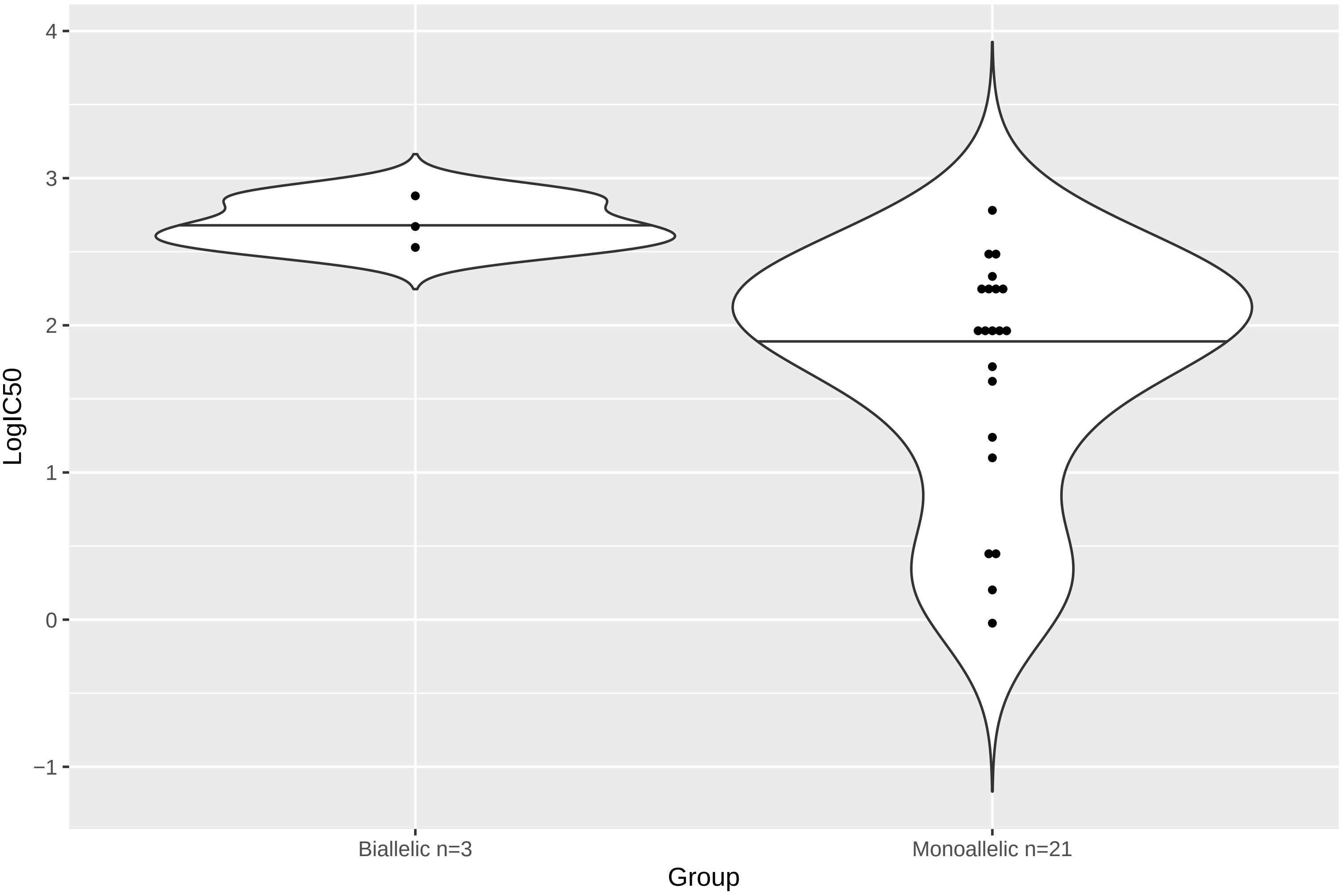

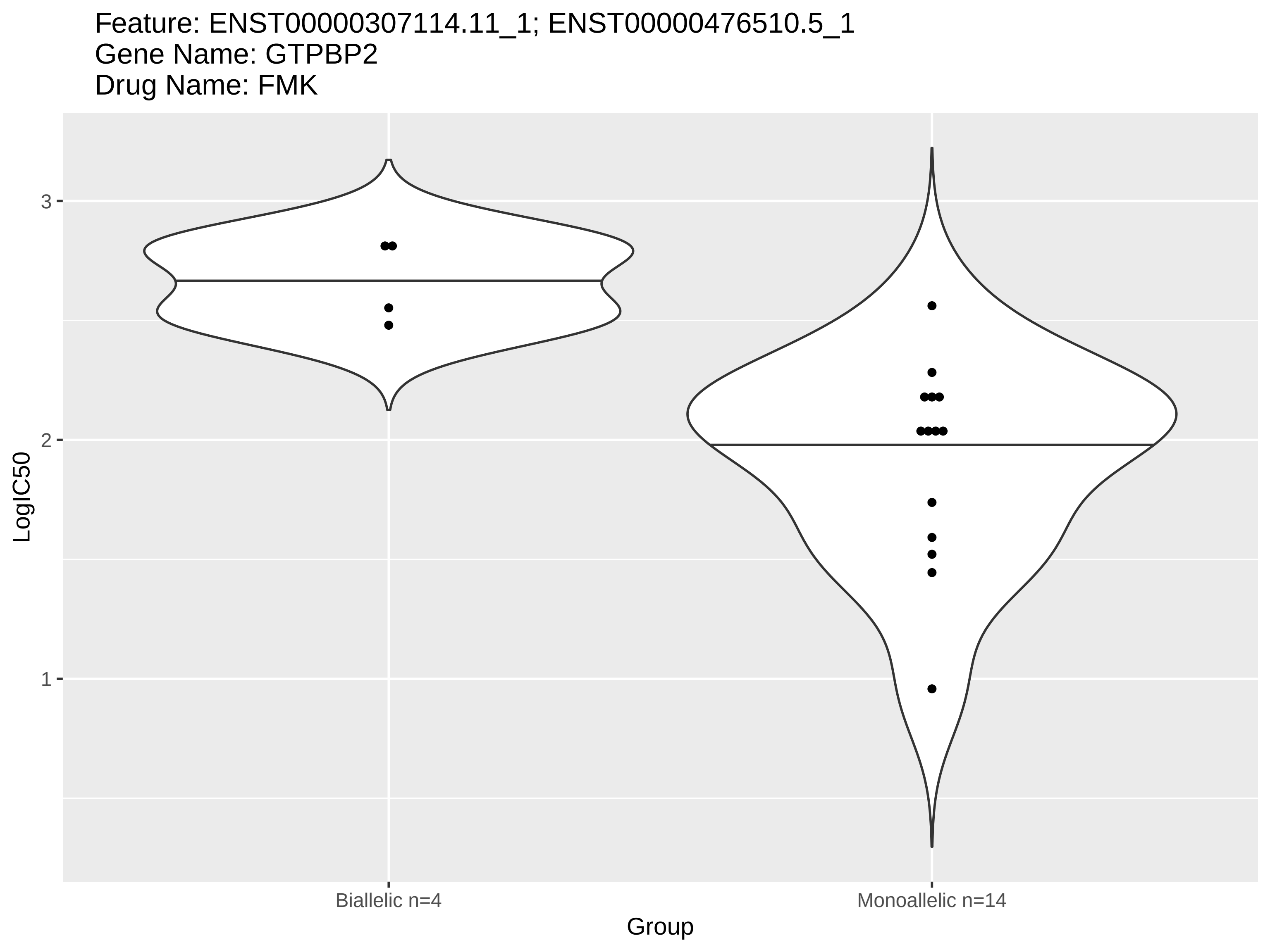

Feature: ENST00000307114.11_1; ENST00000476510.5_1
Gene Name: GTPBP2
Drug Name: FMK
3
2
1
LogIC50
Biallelic n=4
Monoallelic n=14
Group

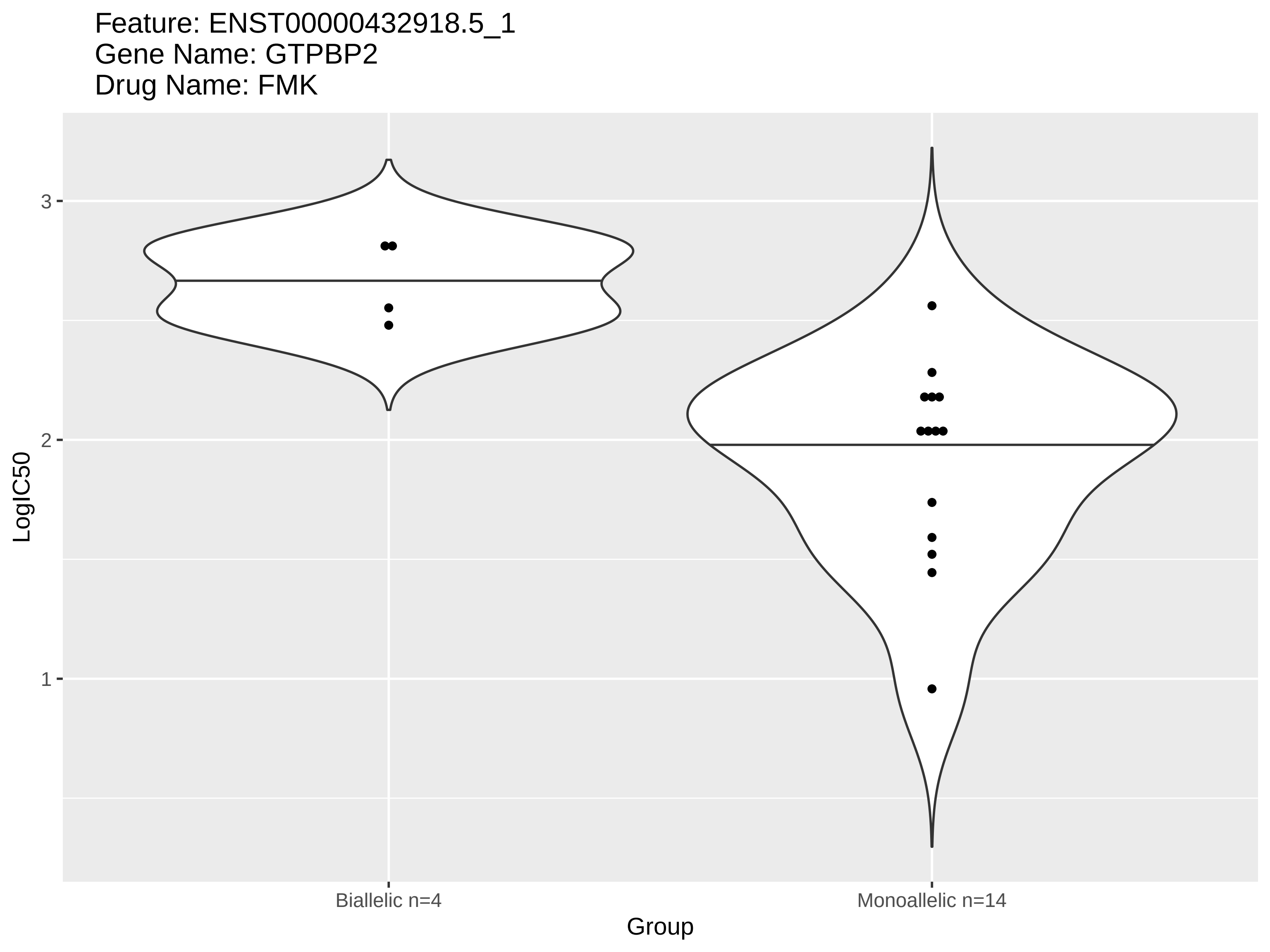
Feature: ENST00000432918.5_1
Gene Name: GTPBP2
Drug Name: FMK
LogIC50
3
2
1
Biallelic n=4
Monoallelic n=14
Group

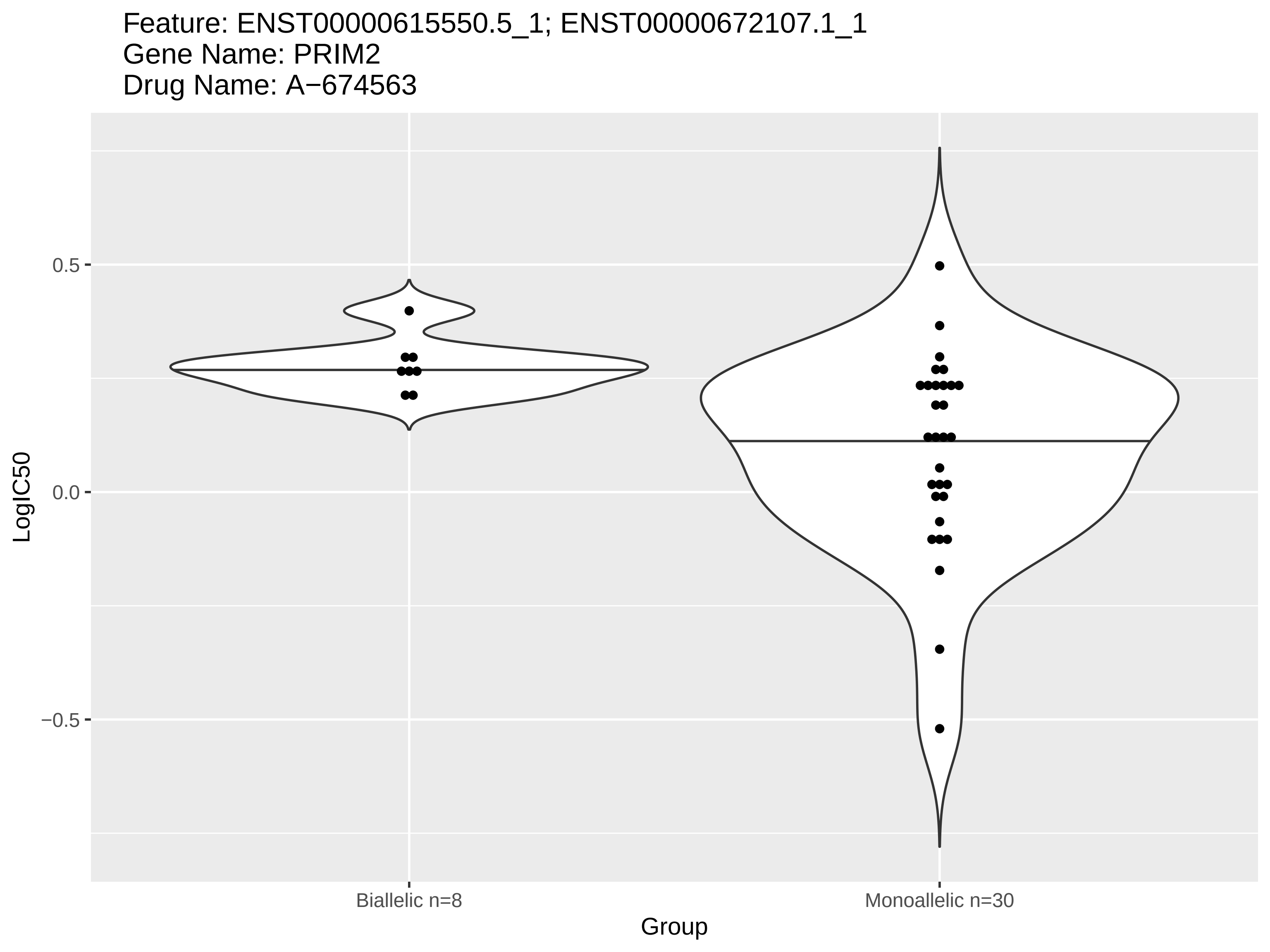

Feature: ENST00000615550.5_1; ENST00000672107.1_1
Gene Name: PRIM2
Drug Name: A−674563
0.5
0.0
−0.5
LogIC50
Biallelic n=8
Monoallelic n=30
Group

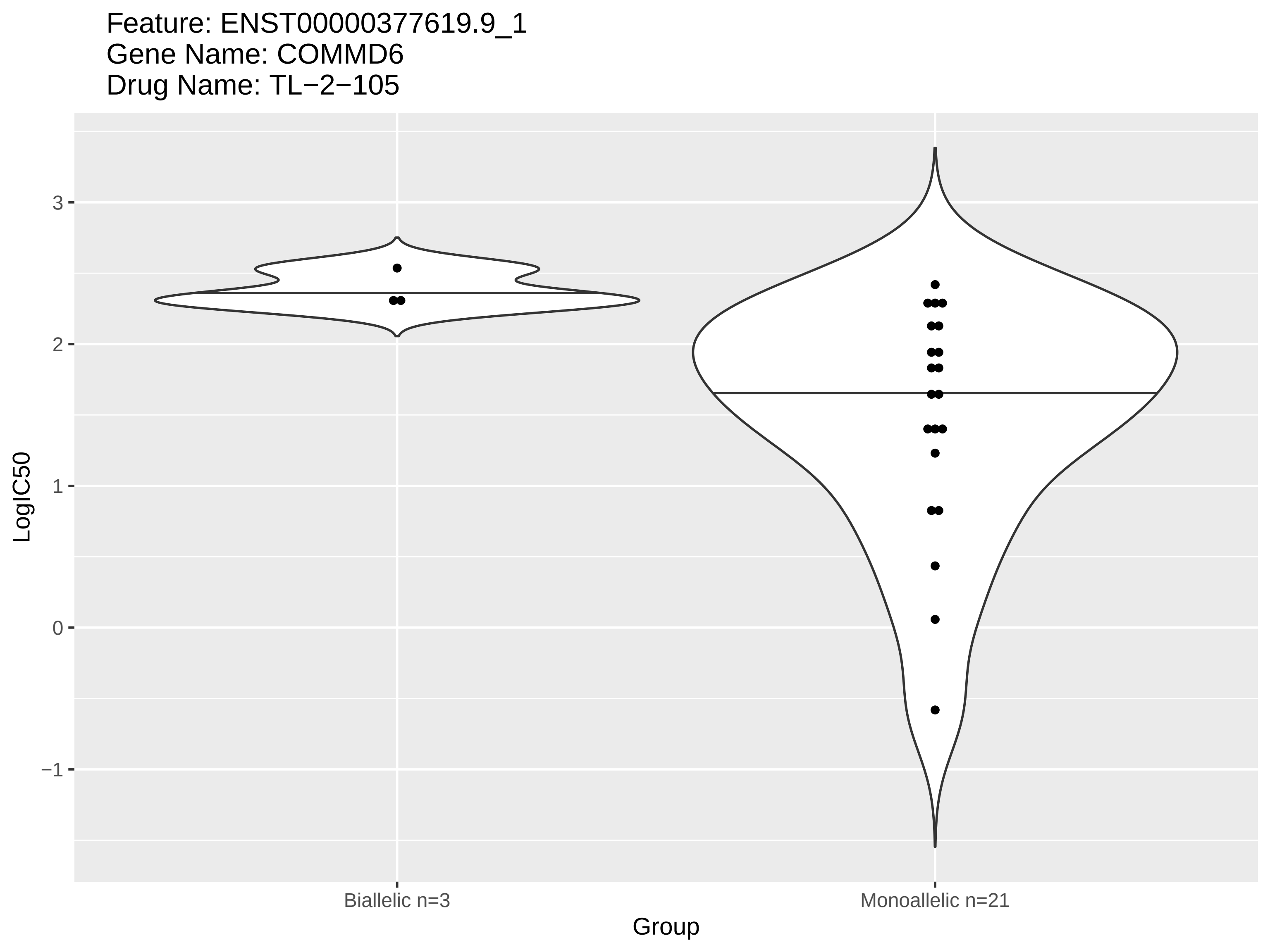
Feature: ENST00000377619.9_1
Gene Name: COMMD6
Drug Name: TL−2−105
LogIC50
3
2
1
0
−1
Biallelic n=3
Monoallelic n=21
Group

Feature: ENST00000533422.5_1
Gene Name: BCLAF1
Drug Name: AGI−5198

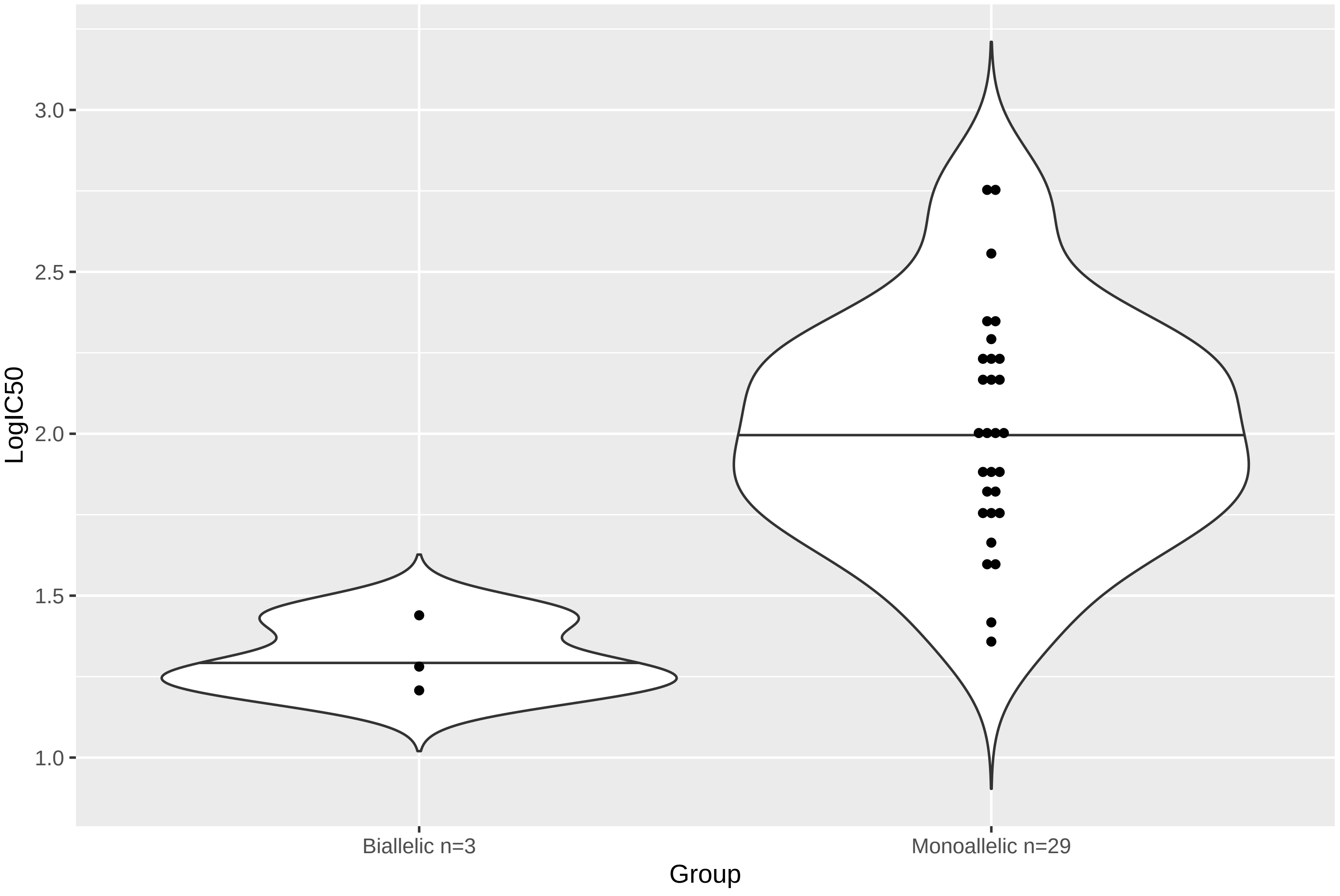

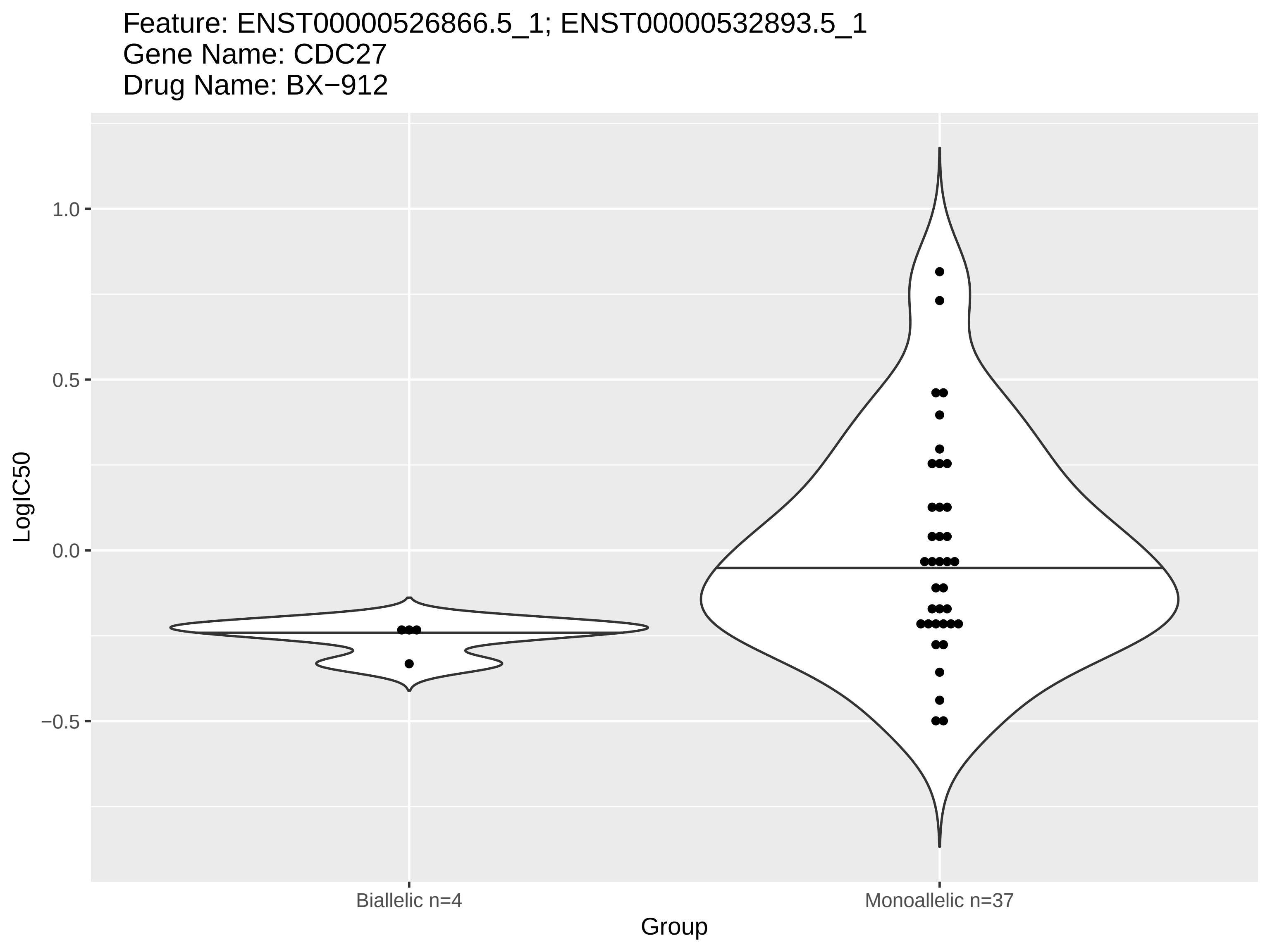

Feature: ENST00000526866.5_1; ENST00000532893.5_1
Gene Name: CDC27
Drug Name: BX−912
LogIC50
1.0
0.5
0.0
−0.5
Biallelic n=4
Monoallelic n=37
Group

Feature: ENST00000377619.9_1
Gene Name: COMMD6
Drug Name: WZ3105

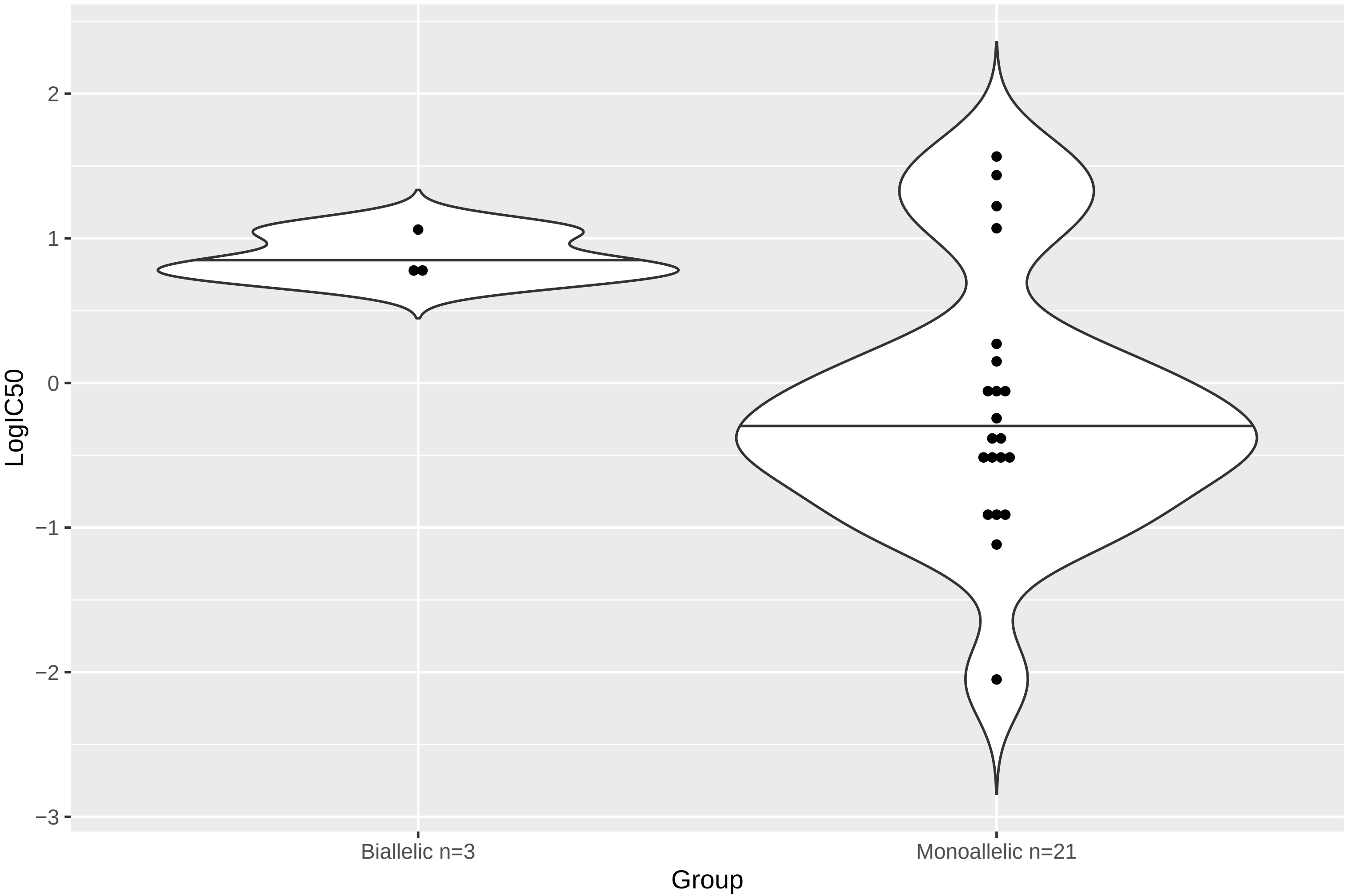

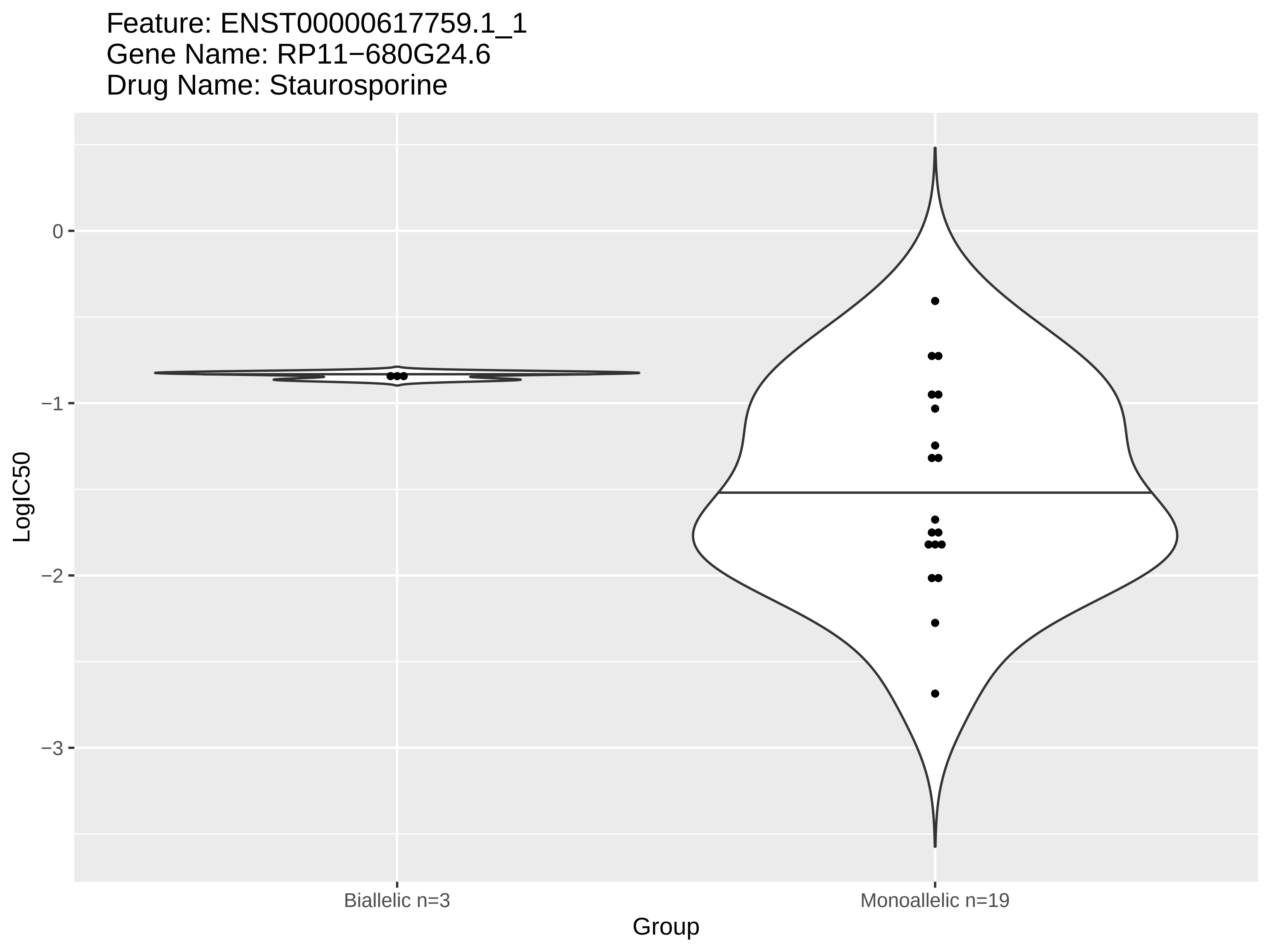
Feature: ENST00000617759.1_1
Gene Name: RP11−680G24.6
Drug Name: Staurosporine
0
−1
−2
−3
LogIC50
Biallelic n=3
Monoallelic n=19
Group

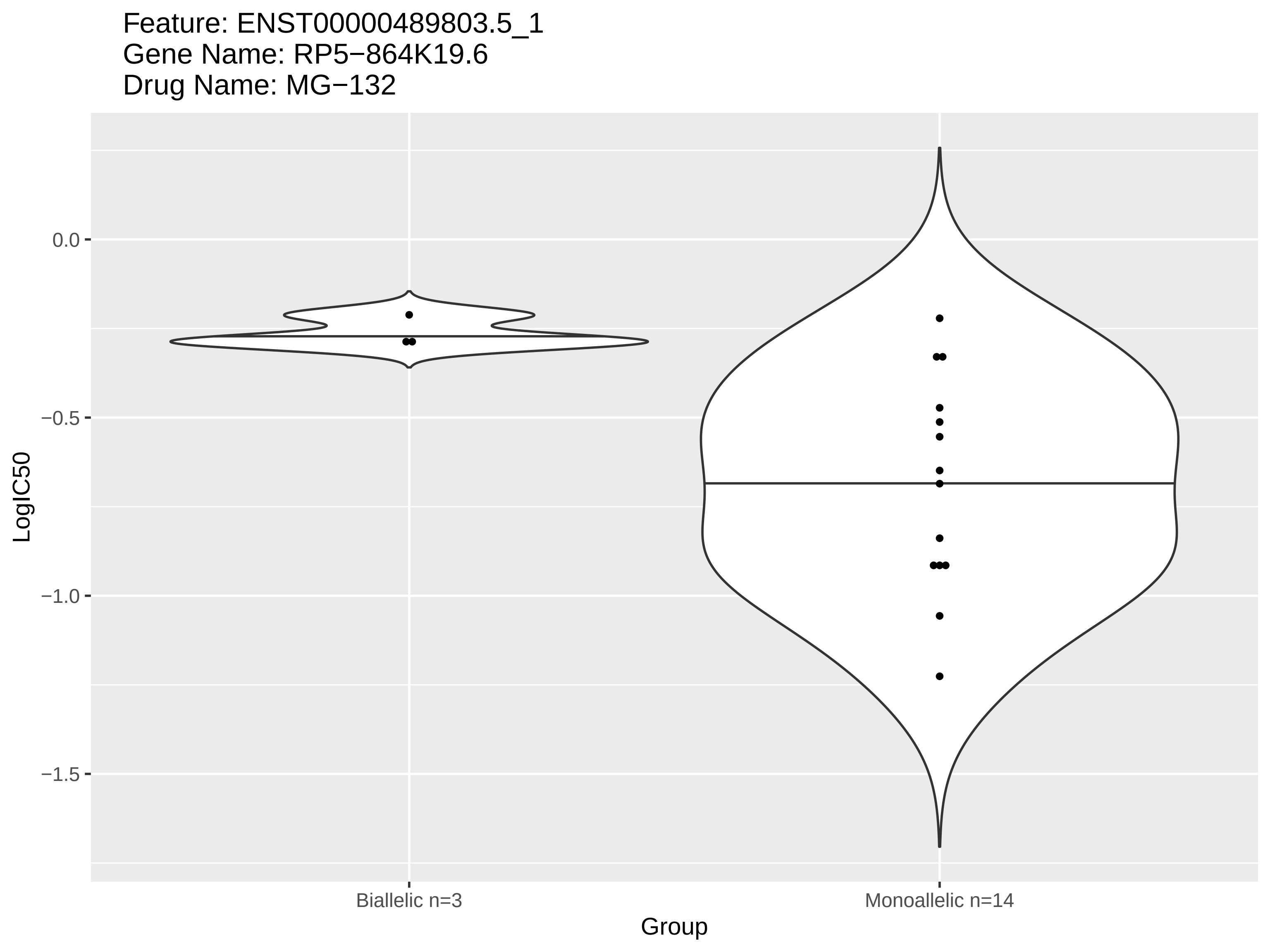

Feature: ENST00000489803.5_1
Gene Name: RP5−864K19.6
Drug Name: MG−132
0.0
−0.5
−1.0
−1.5
LogIC50
Biallelic n=3
Monoallelic n=14
Group

Feature: ENST00000533422.5_1
Gene Name: BCLAF1
Drug Name: MK−1775

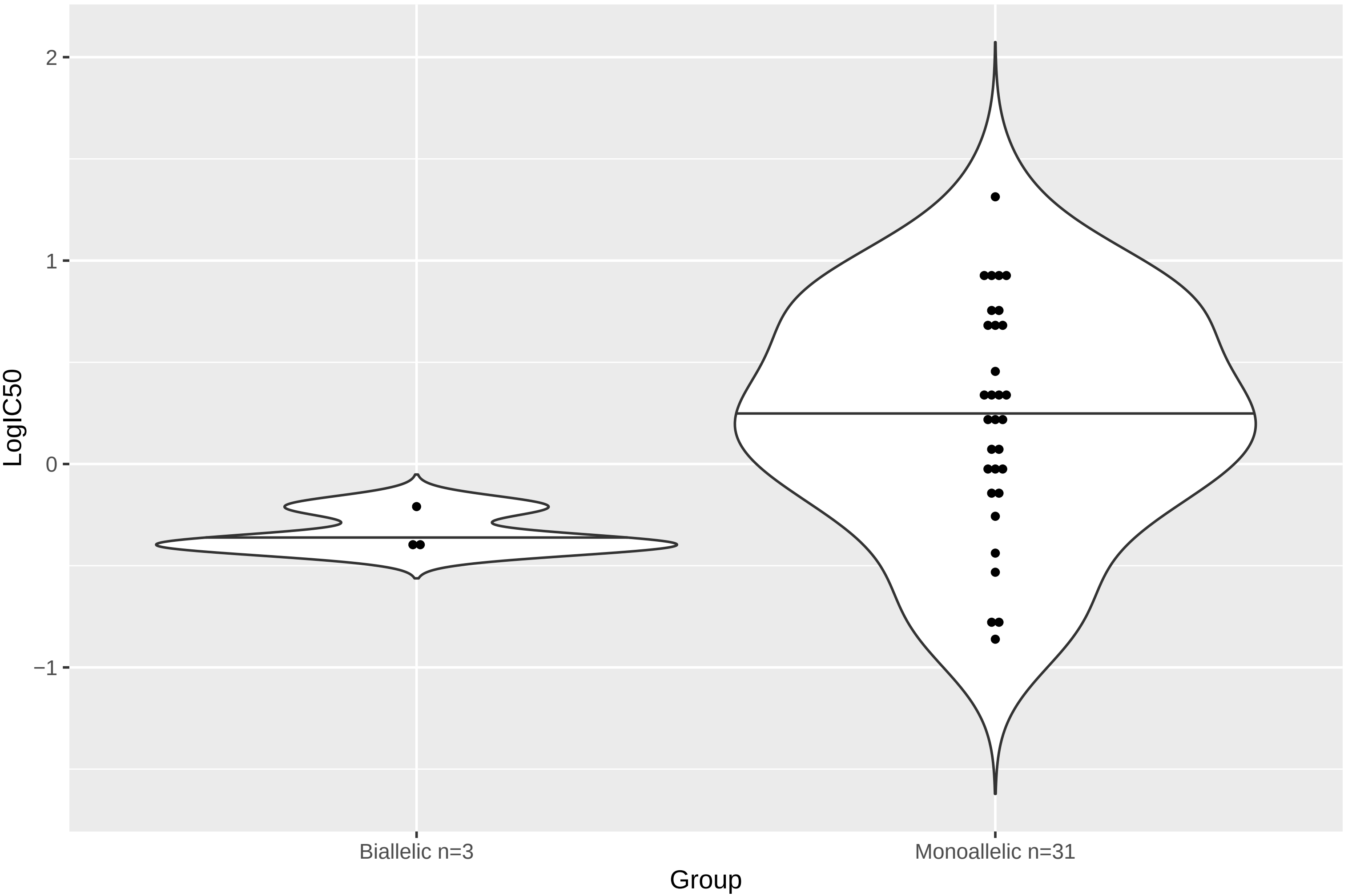

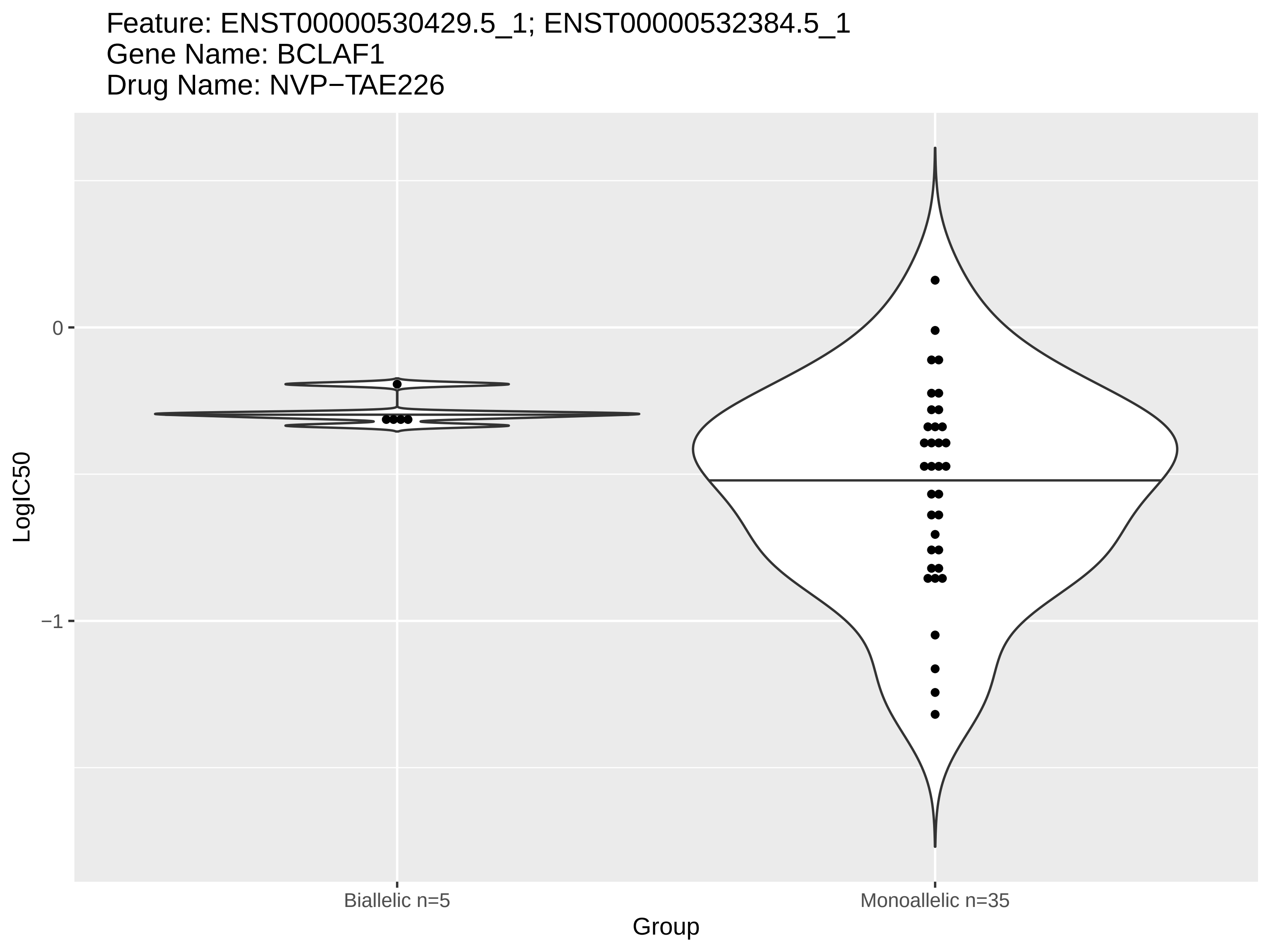

Feature: ENST00000530429.5_1; ENST00000532384.5_1
Gene Name: BCLAF1
Drug Name: NVP−TAE226
0
−1
LogIC50
Biallelic n=5
Monoallelic n=35
Group

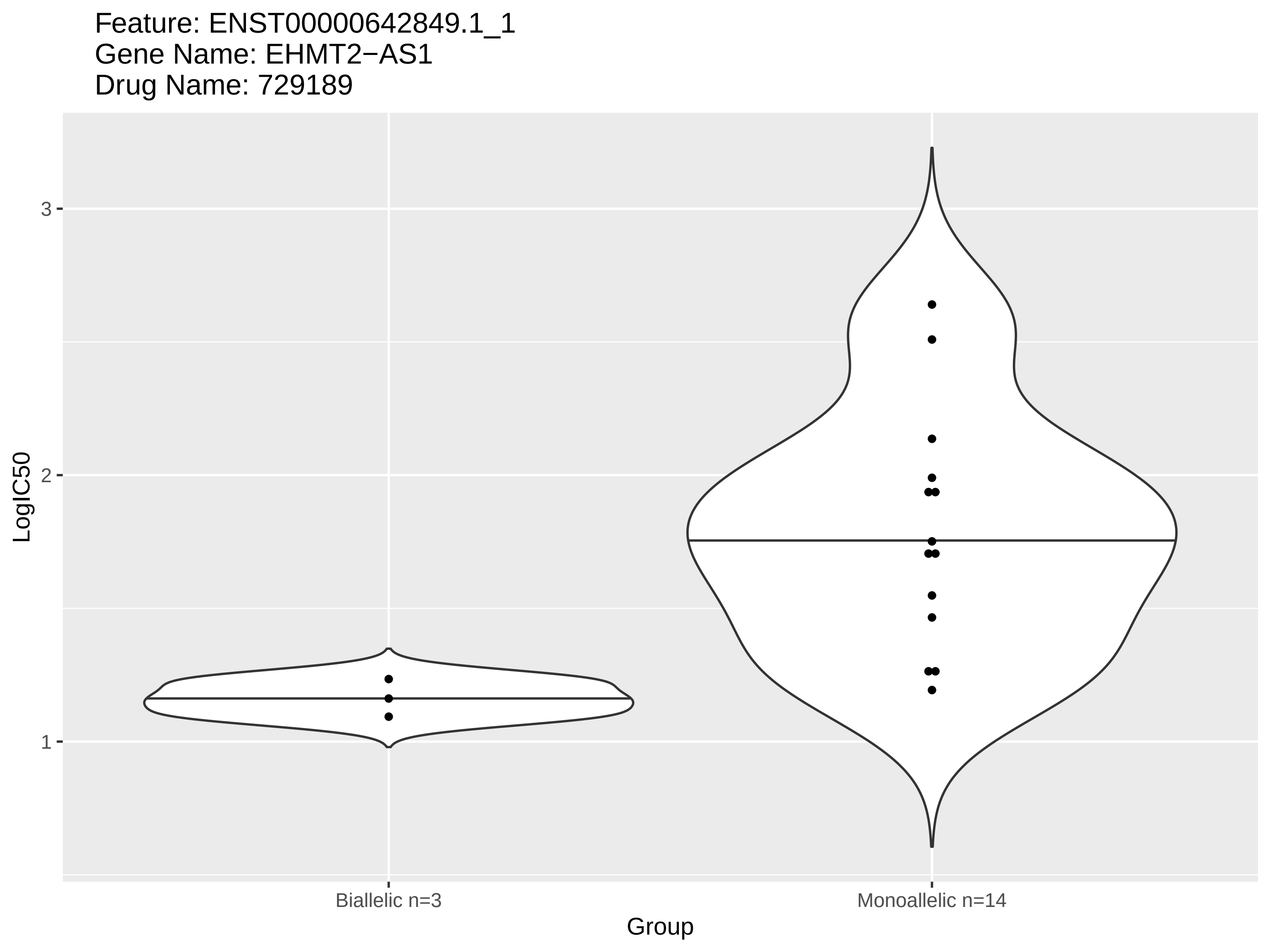
Feature: ENST00000642849.1_1
Gene Name: EHMT2−AS1
Drug Name: 729189
LogIC50
3
2
1
Biallelic n=3
Monoallelic n=14
Group

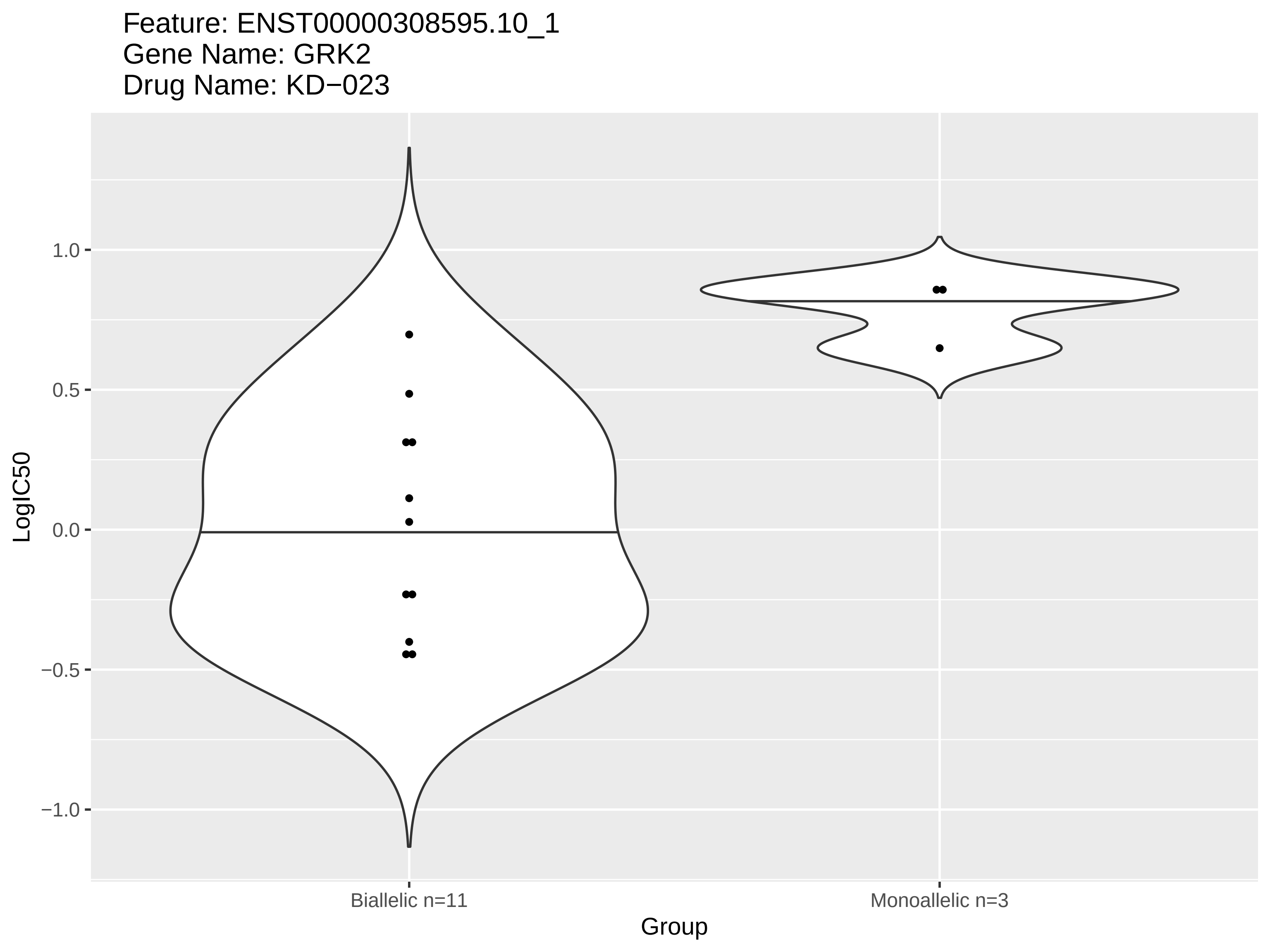
Feature: ENST00000308595.10_1
Gene Name: GRK2
Drug Name: KD−023
LogIC50
1.0
0.5
0.0
−0.5
−1.0
Biallelic n=11
Monoallelic n=3
Group

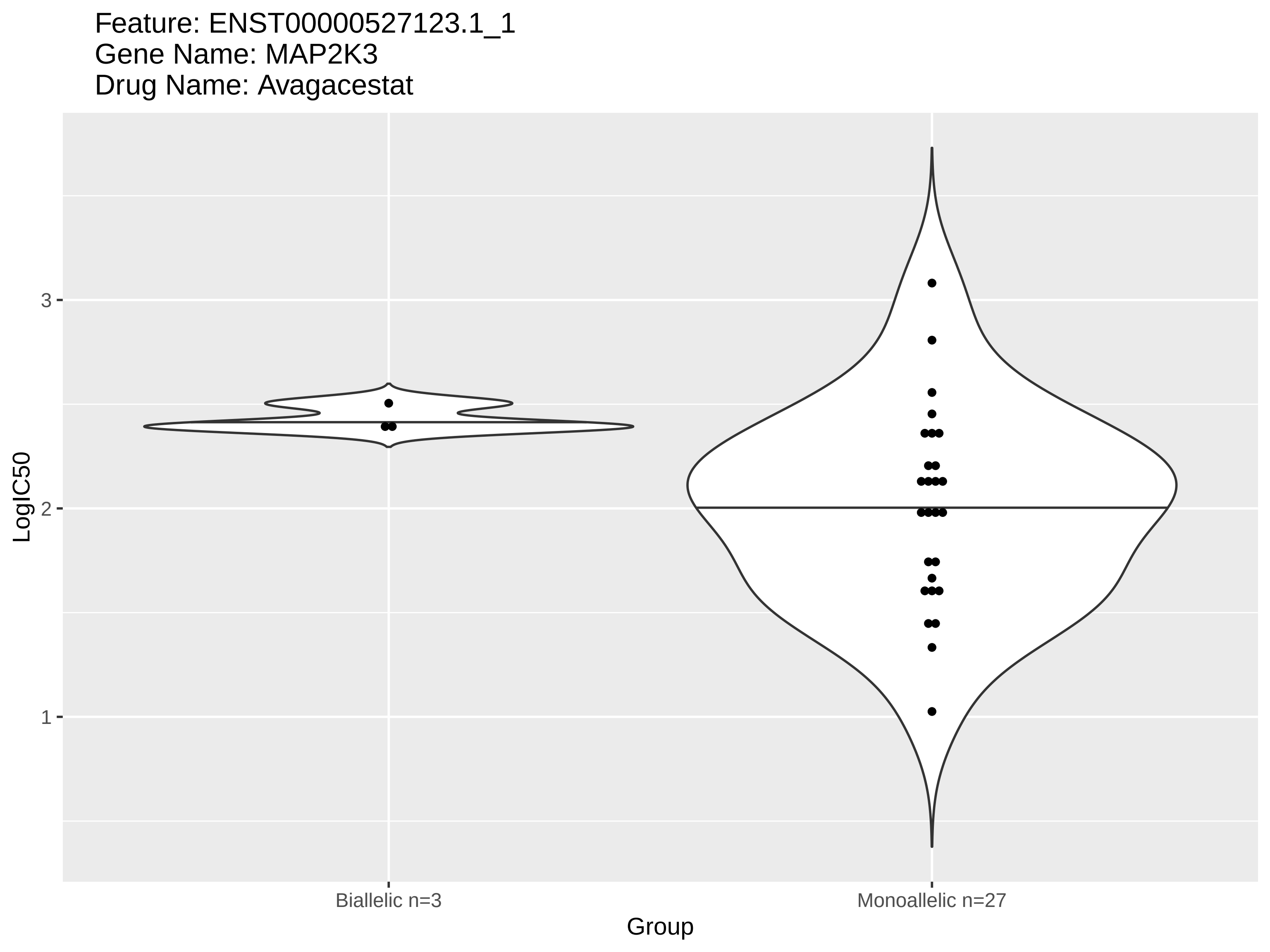
Feature: ENST00000527123.1_1
Gene Name: MAP2K3
Drug Name: Avagacestat
LogIC50
3
2
1
Biallelic n=3
Monoallelic n=27
Group

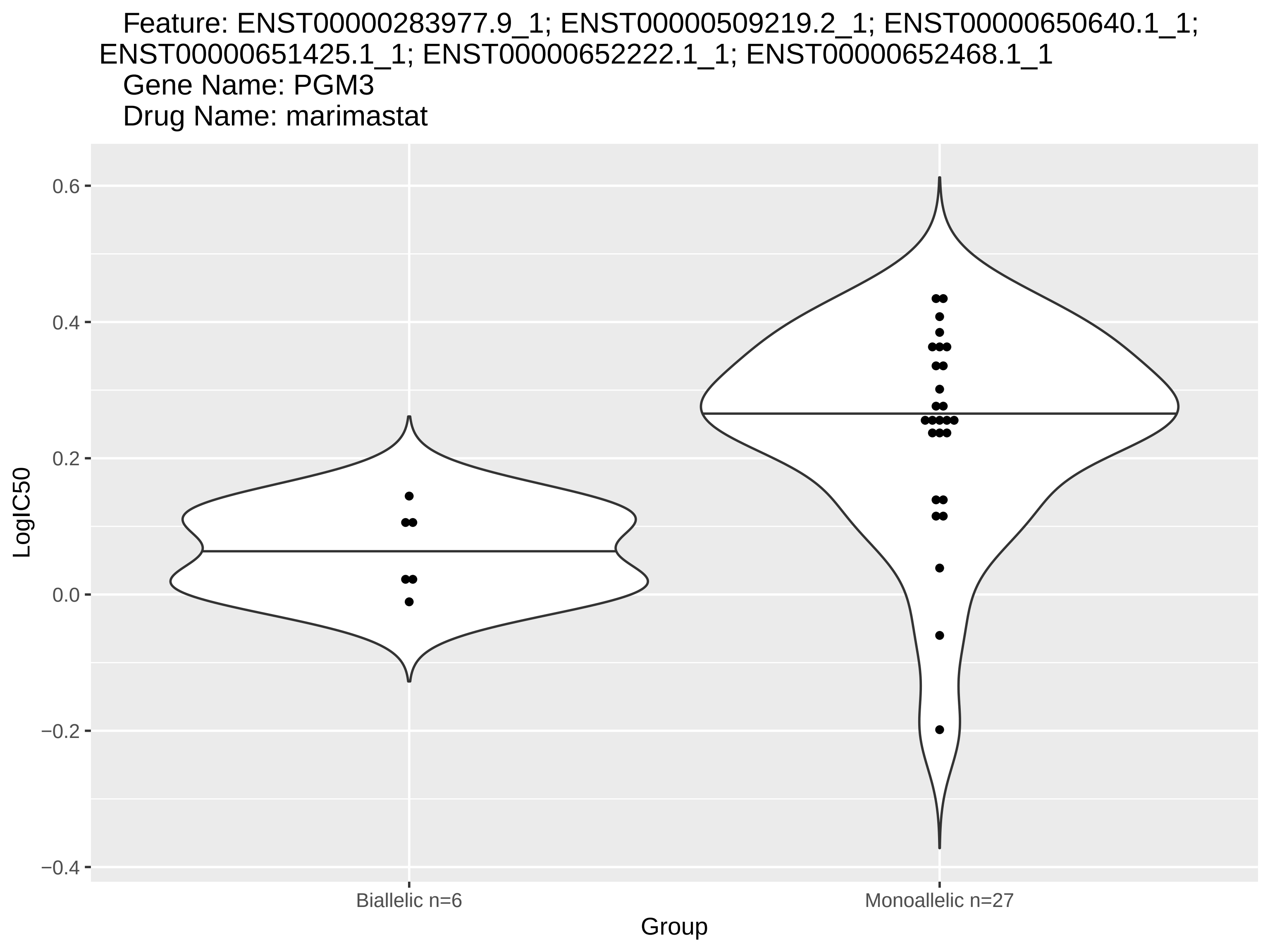
Feature: ENST00000283977.9_1; ENST00000509219.2_1; ENST00000650640.1_1;
ENST00000651425.1_1; ENST00000652222.1_1; ENST00000652468.1_1
Gene Name: PGM3
Drug Name: marimastat
LogIC50
0.6
0.4
0.2
0.0
−0.2
−0.4
Biallelic n=6
Monoallelic n=27
Group

Feature: ENST00000506587.5_1
Gene Name: PGM3
Drug Name: marimastat

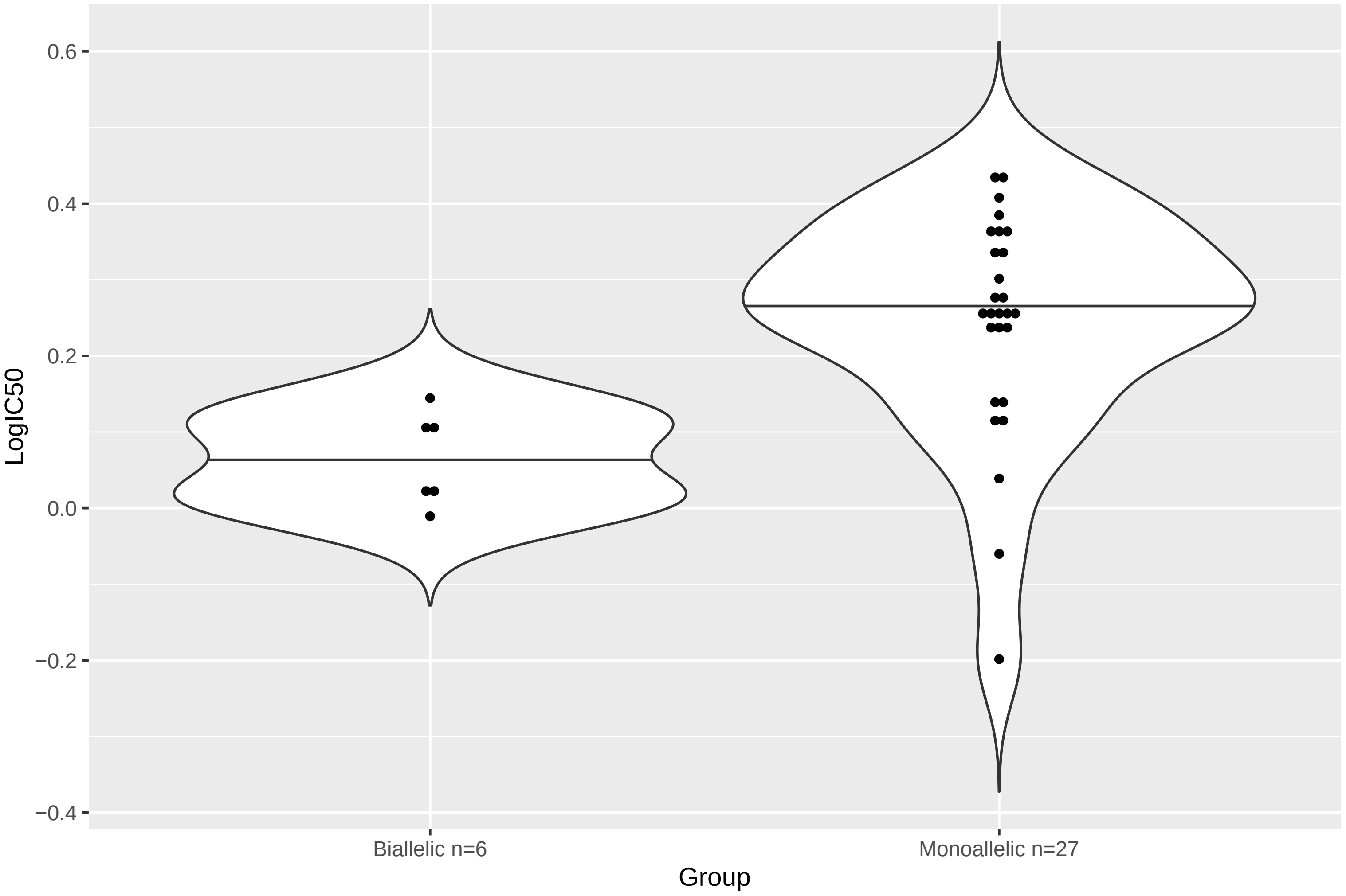

Feature: ENST00000443119.7_1
Gene Name: RP11−529K1.3
Drug Name: EHT−1864

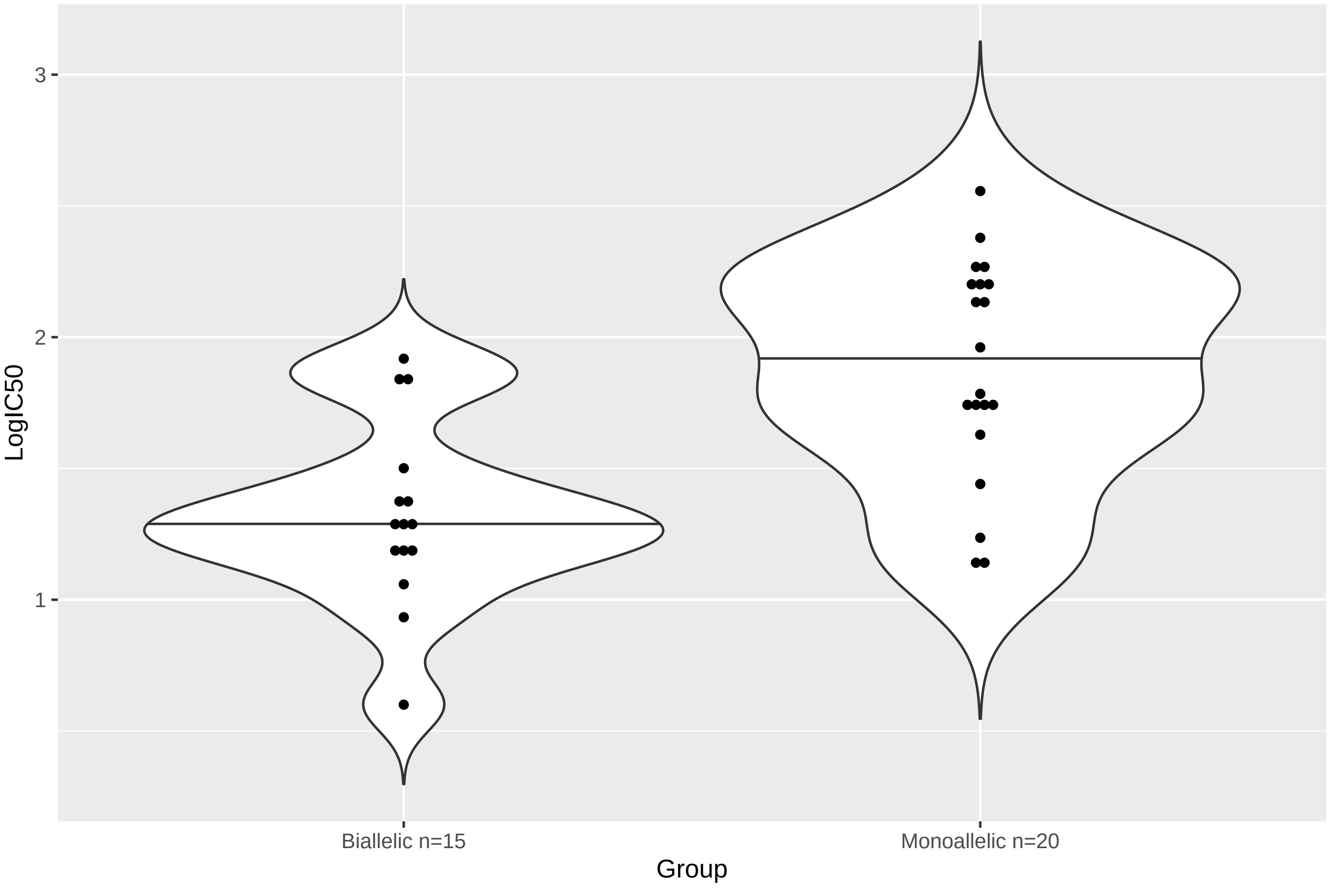

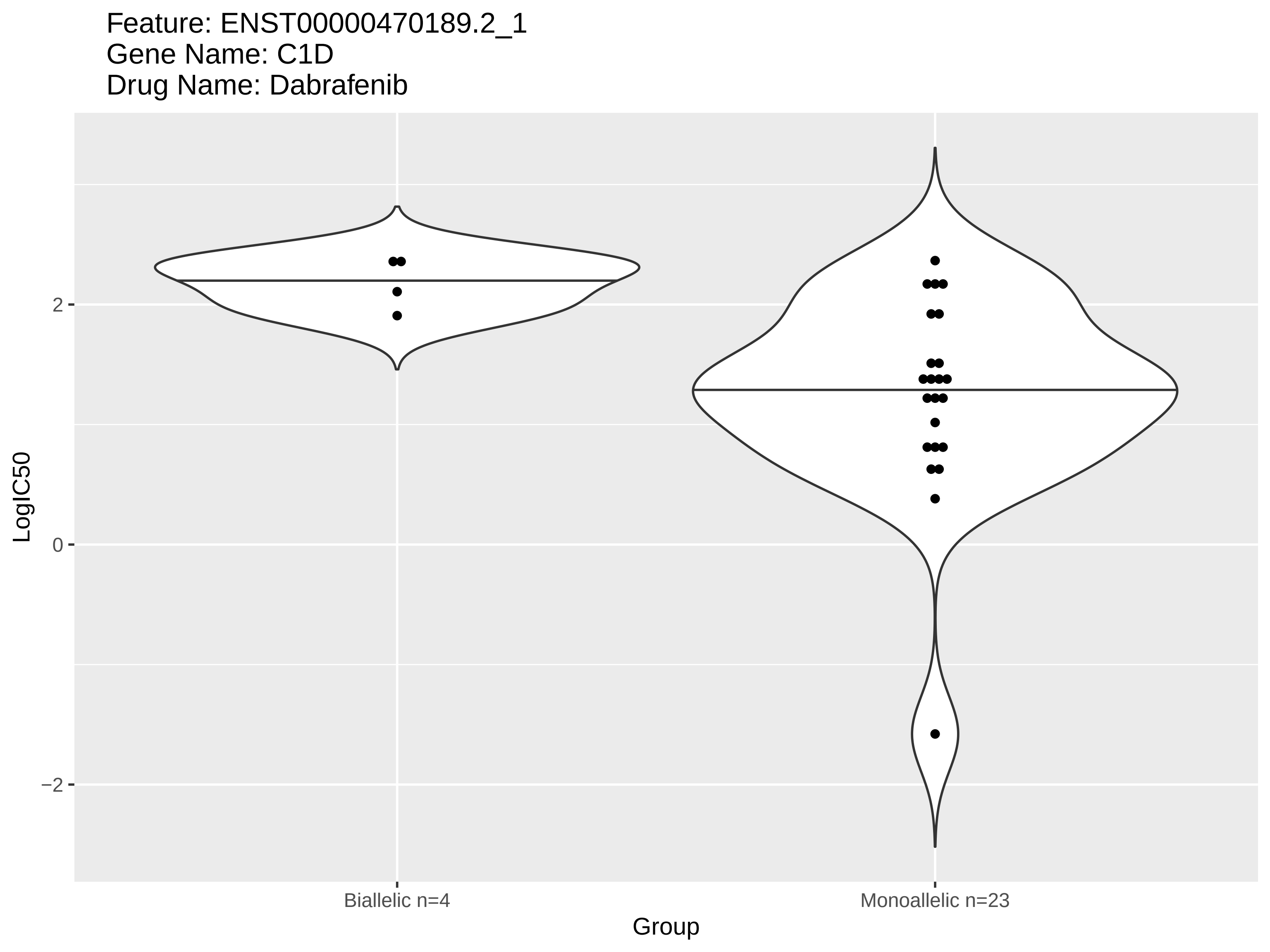

Feature: ENST00000470189.2_1
Gene Name: C1D
Drug Name: Dabrafenib
LogIC50
2
0
−2
Biallelic n=4
Monoallelic n=23
Group

Feature: ENST00000353331.8_1; ENST00000531224.6_1
Gene Name: BCLAF1
Drug Name: colchicine

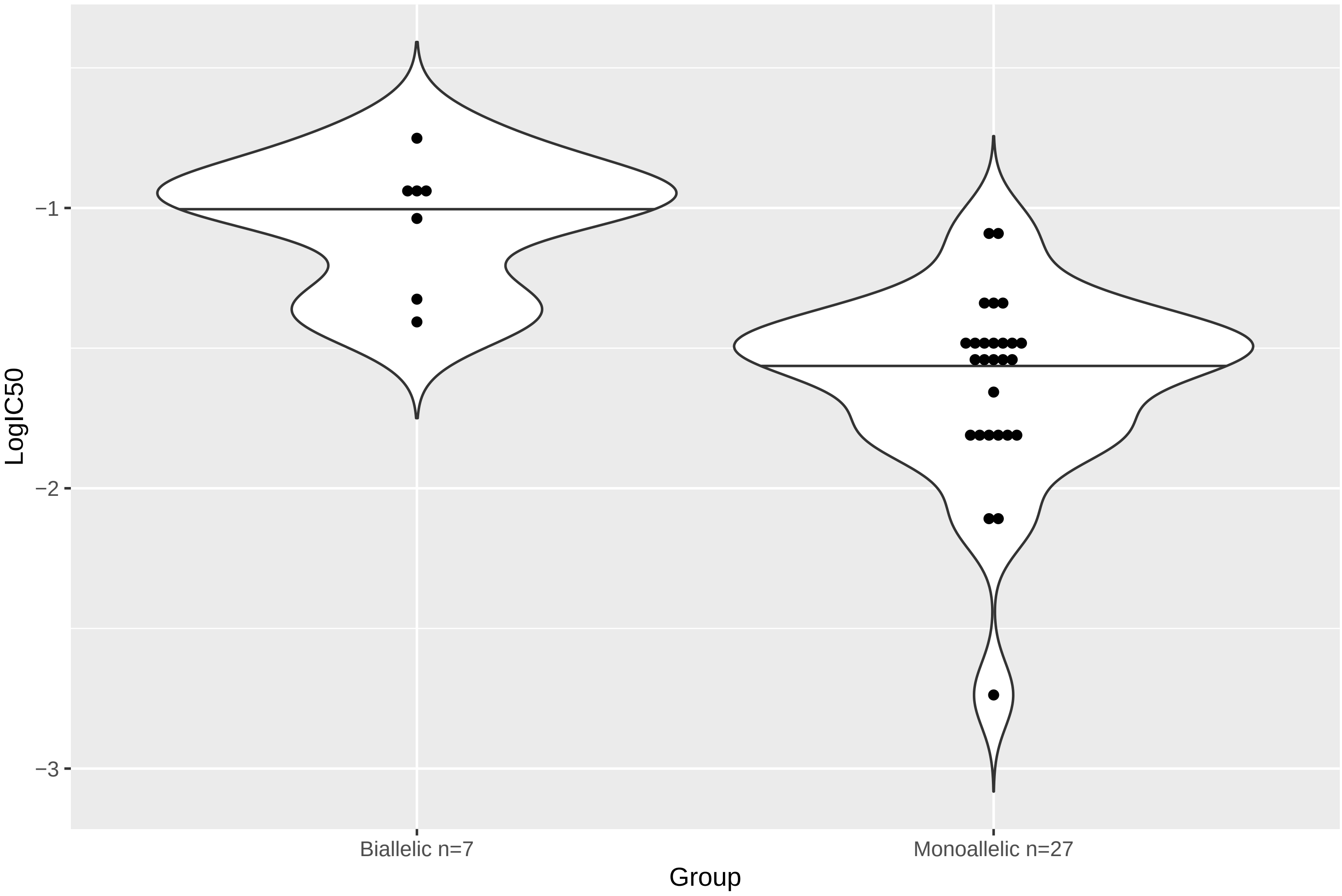

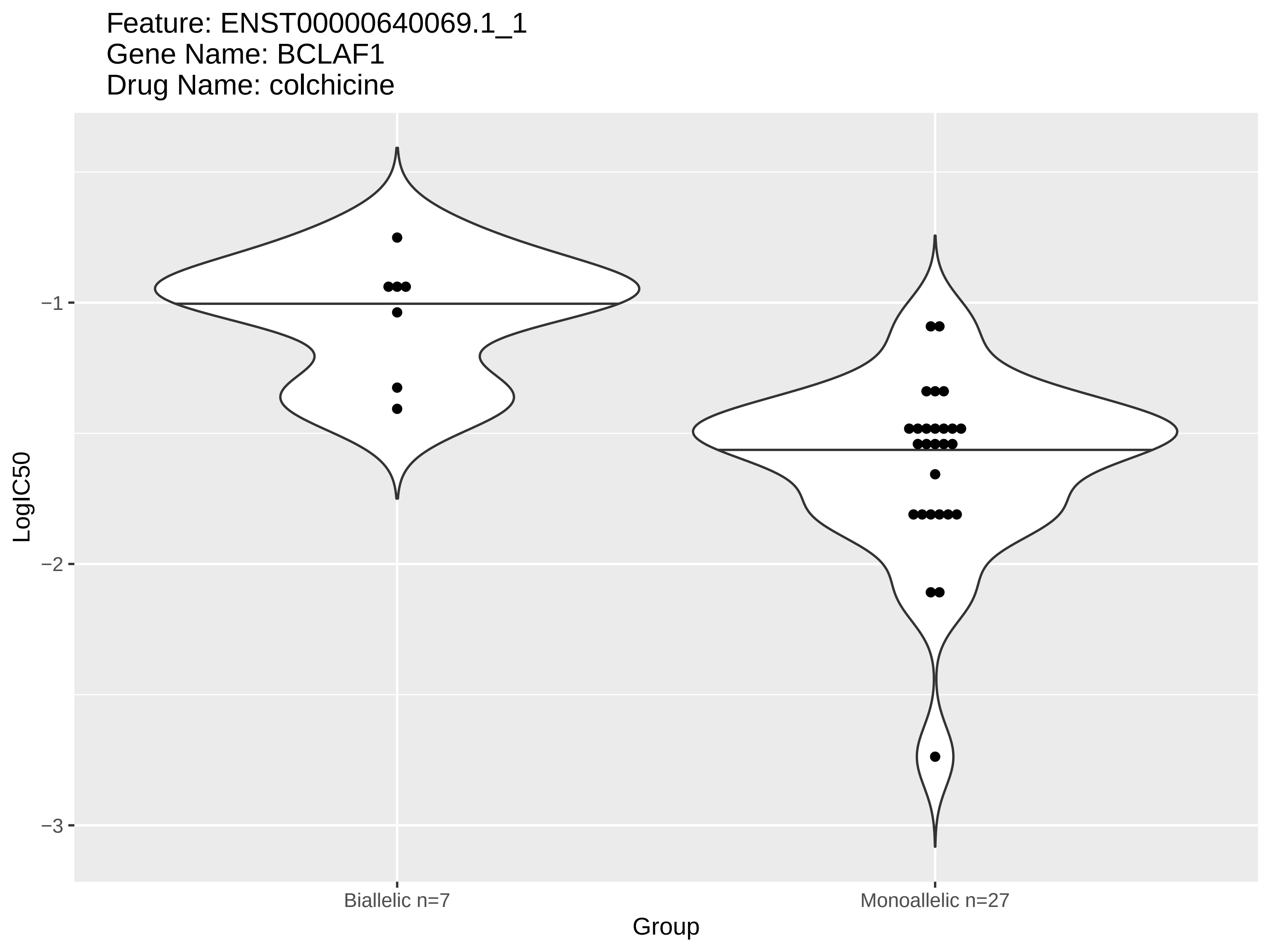

Feature: ENST00000640069.1_1
Gene Name: BCLAF1
Drug Name: colchicine
LogIC50
−1
−2
−3
Biallelic n=7
Monoallelic n=27
Group

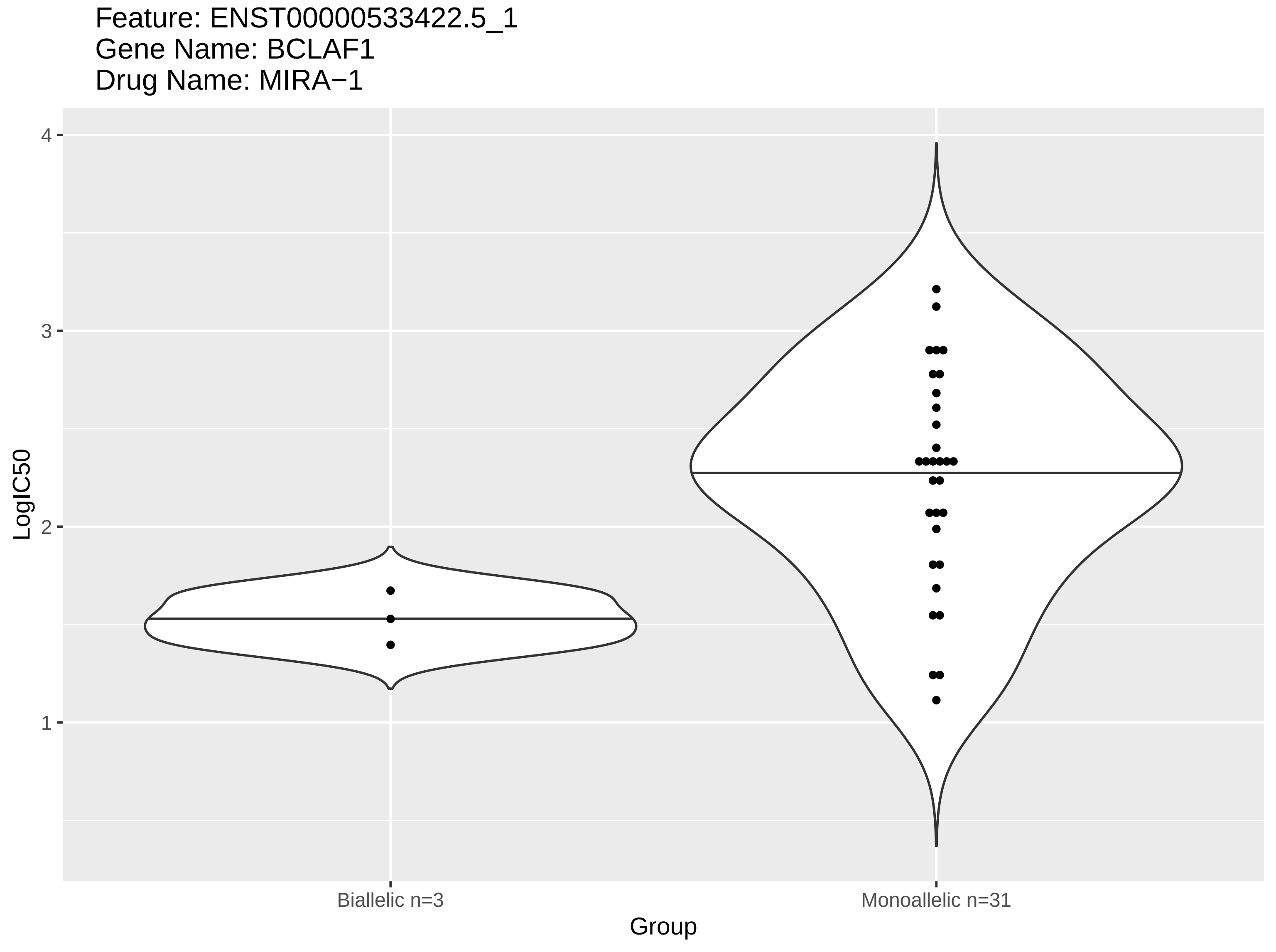

Feature: ENST00000533422.5_1
Gene Name: BCLAF1
Drug Name: MIRA−1
LogIC50
4
3
2
1
Biallelic n=3
Monoallelic n=31
Group

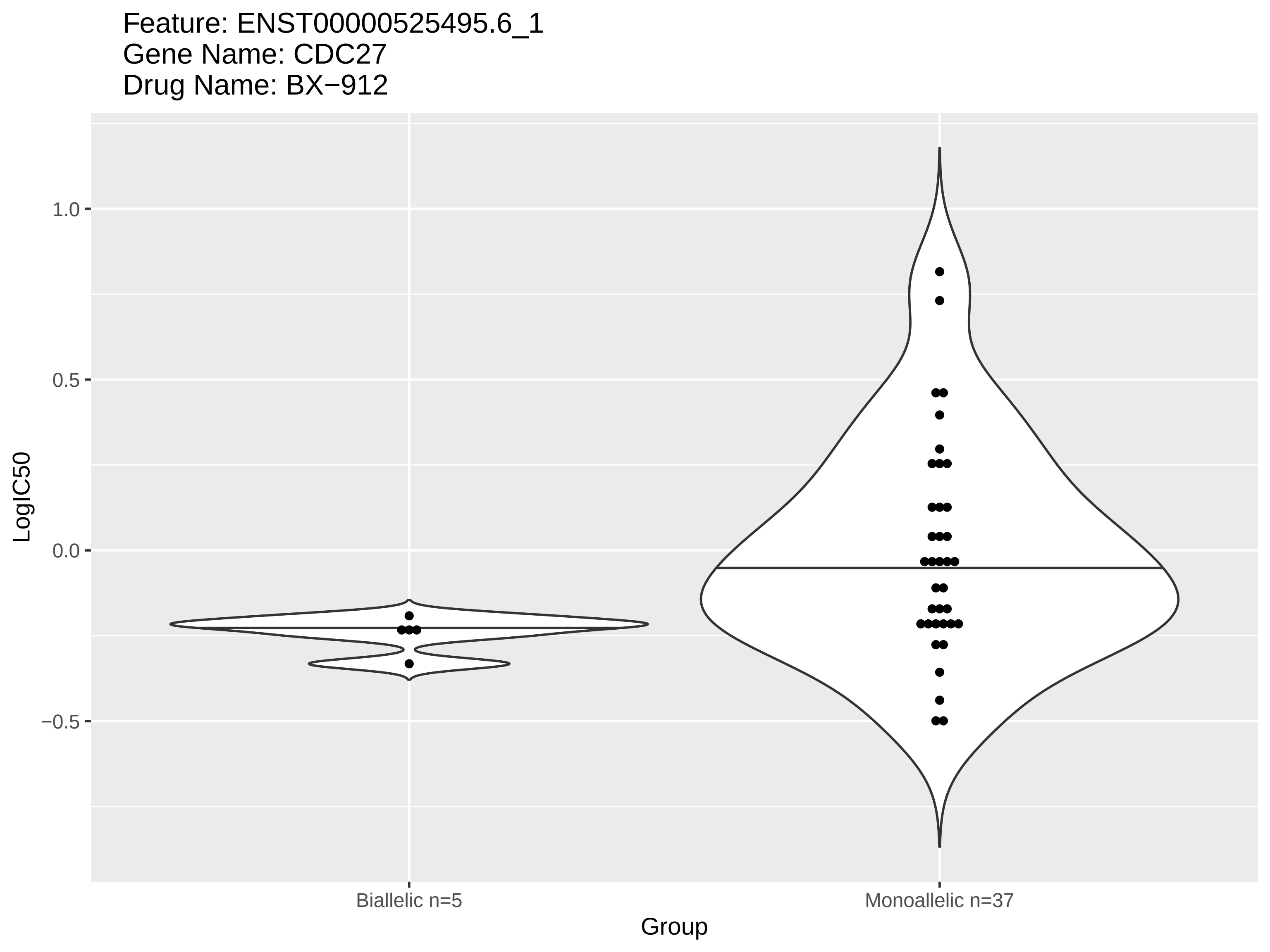

Feature: ENST00000525495.6_1
Gene Name: CDC27
Drug Name: BX−912
1.0
0.5
0.0
−0.5
LogIC50
Biallelic n=5
Monoallelic n=37
Group

Feature: ENST00000573502.1_1
Gene Name: CDC27
Drug Name: 10−hydroxycamptothecin

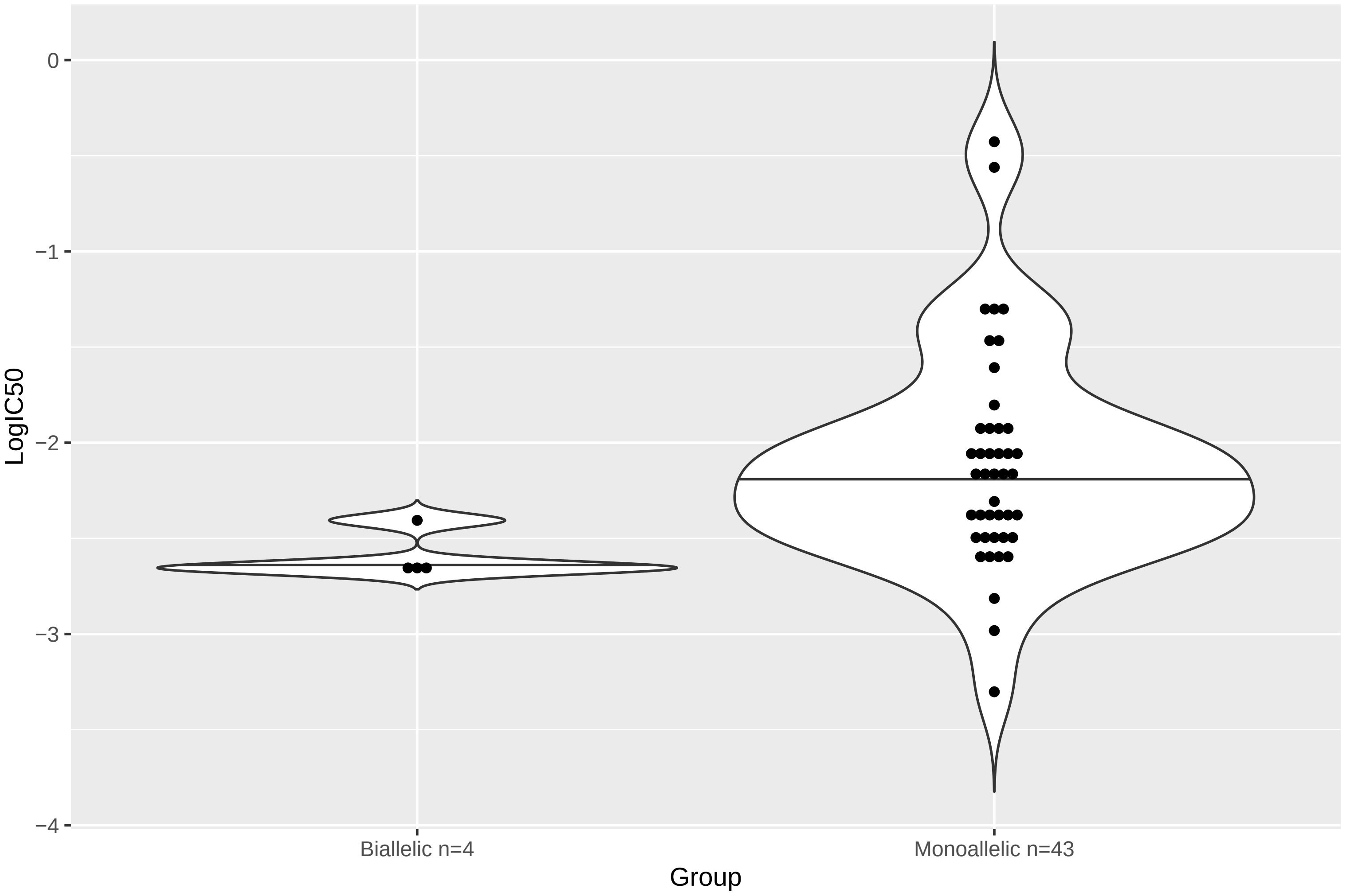

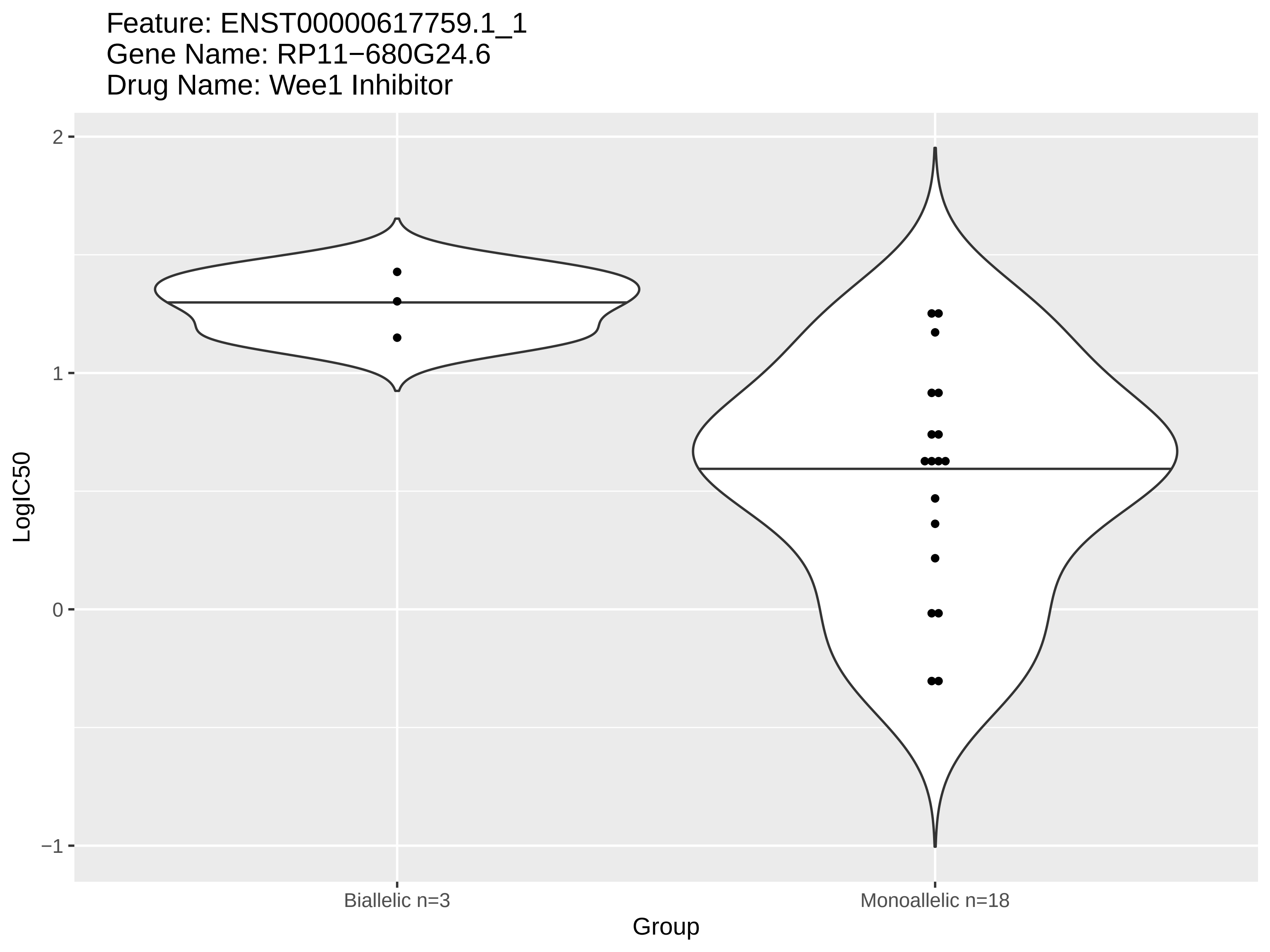
Feature: ENST00000617759.1_1
Gene Name: RP11−680G24.6
Drug Name: Wee1 Inhibitor
LogIC50
2
1
0
−1
Biallelic n=3
Monoallelic n=18
Group

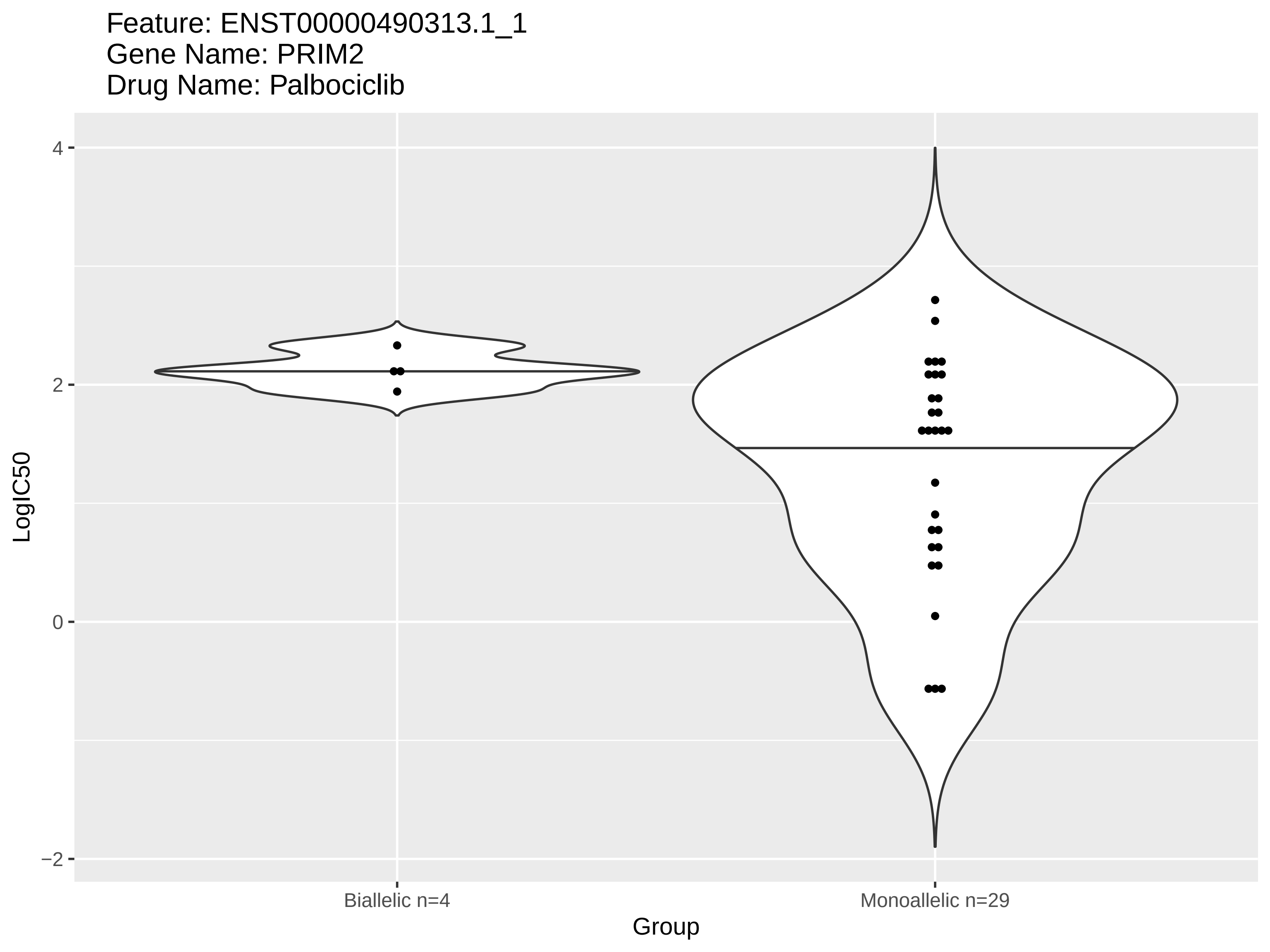
Feature: ENST00000490313.1_1
Gene Name: PRIM2
Drug Name: Palbociclib
4
2
0
−2
LogIC50
Biallelic n=4
Monoallelic n=29
Group

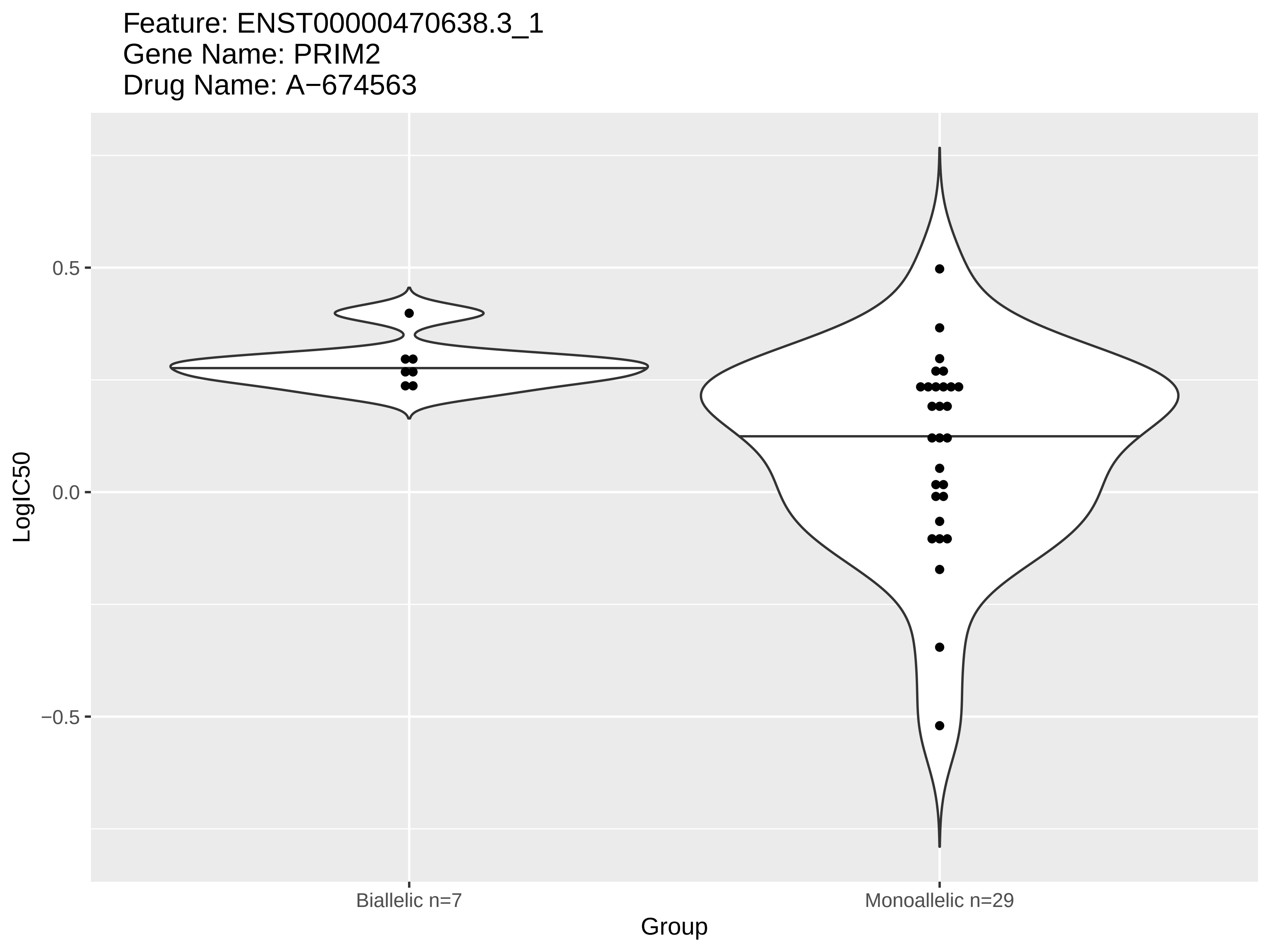
Feature: ENST00000470638.3_1
Gene Name: PRIM2
Drug Name: A−674563
0.5
0.0
−0.5
LogIC50
Biallelic n=7
Monoallelic n=29
Group

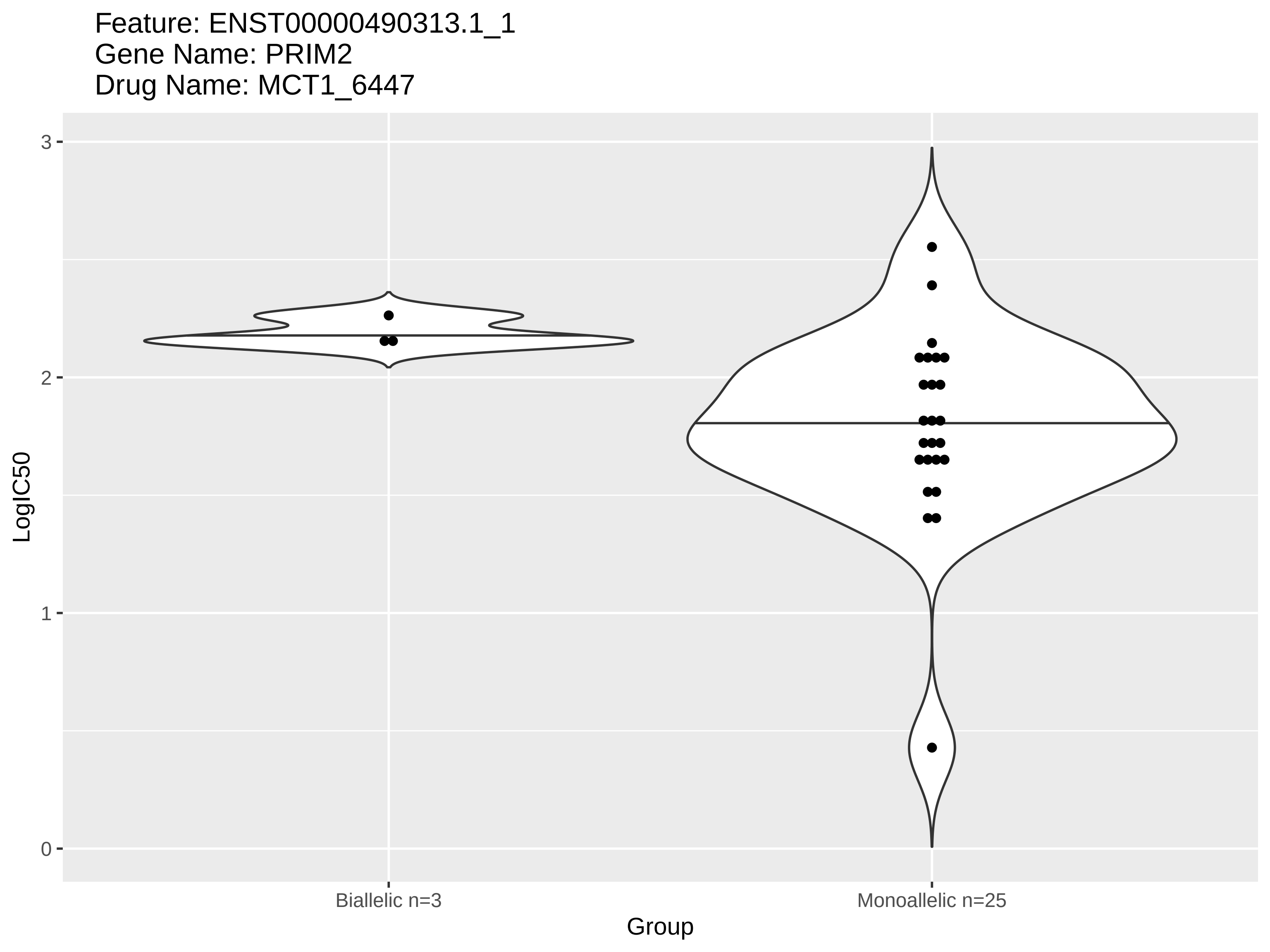
Feature: ENST00000490313.1_1
Gene Name: PRIM2
Drug Name: MCT1_6447
LogIC50
0
1
2
3
Biallelic n=3
Monoallelic n=25
Group

Feature: ENST00000615550.5_1; ENST00000672107.1_1
Gene Name: PRIM2
Drug Name: dinaciclib

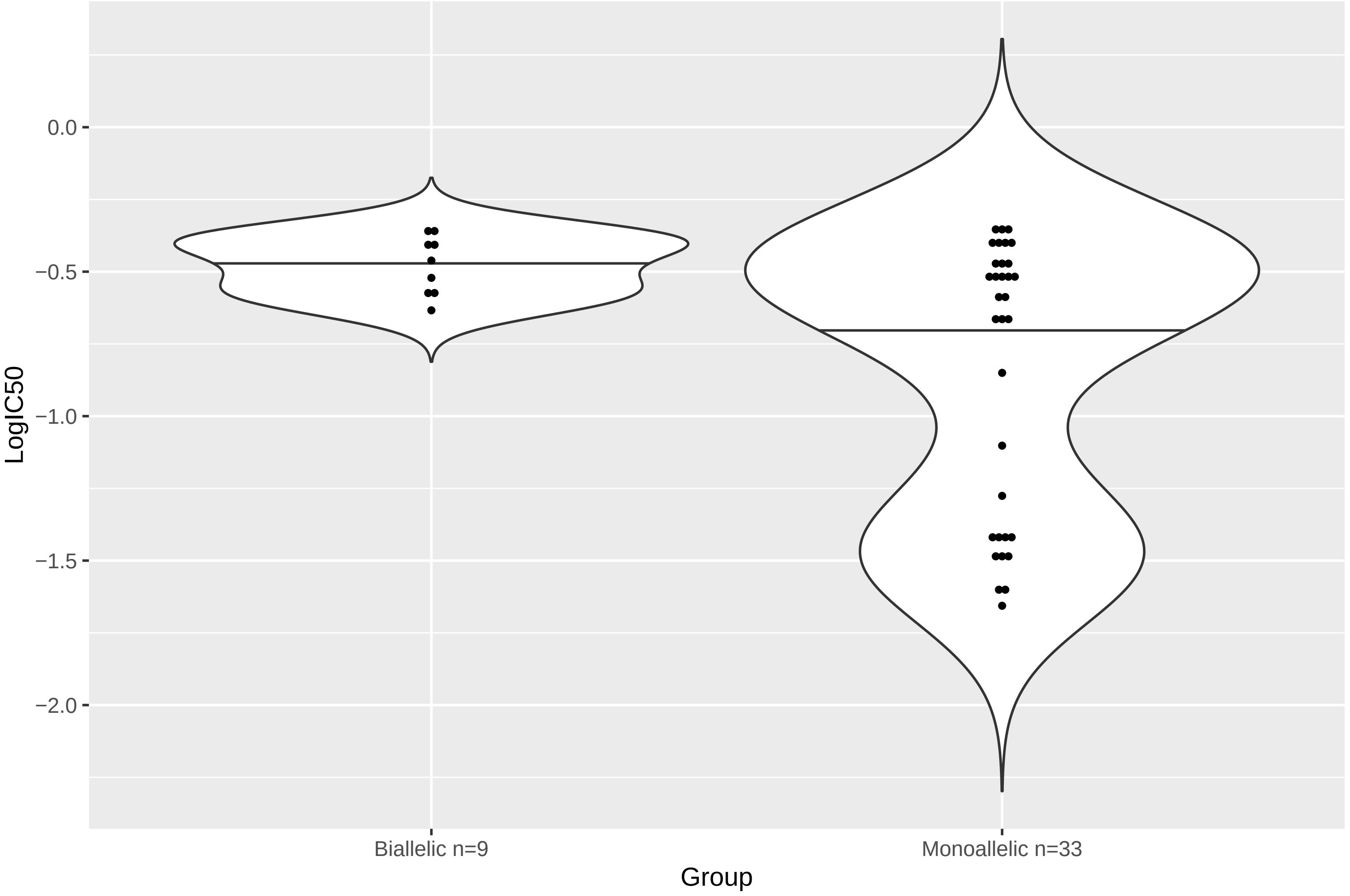

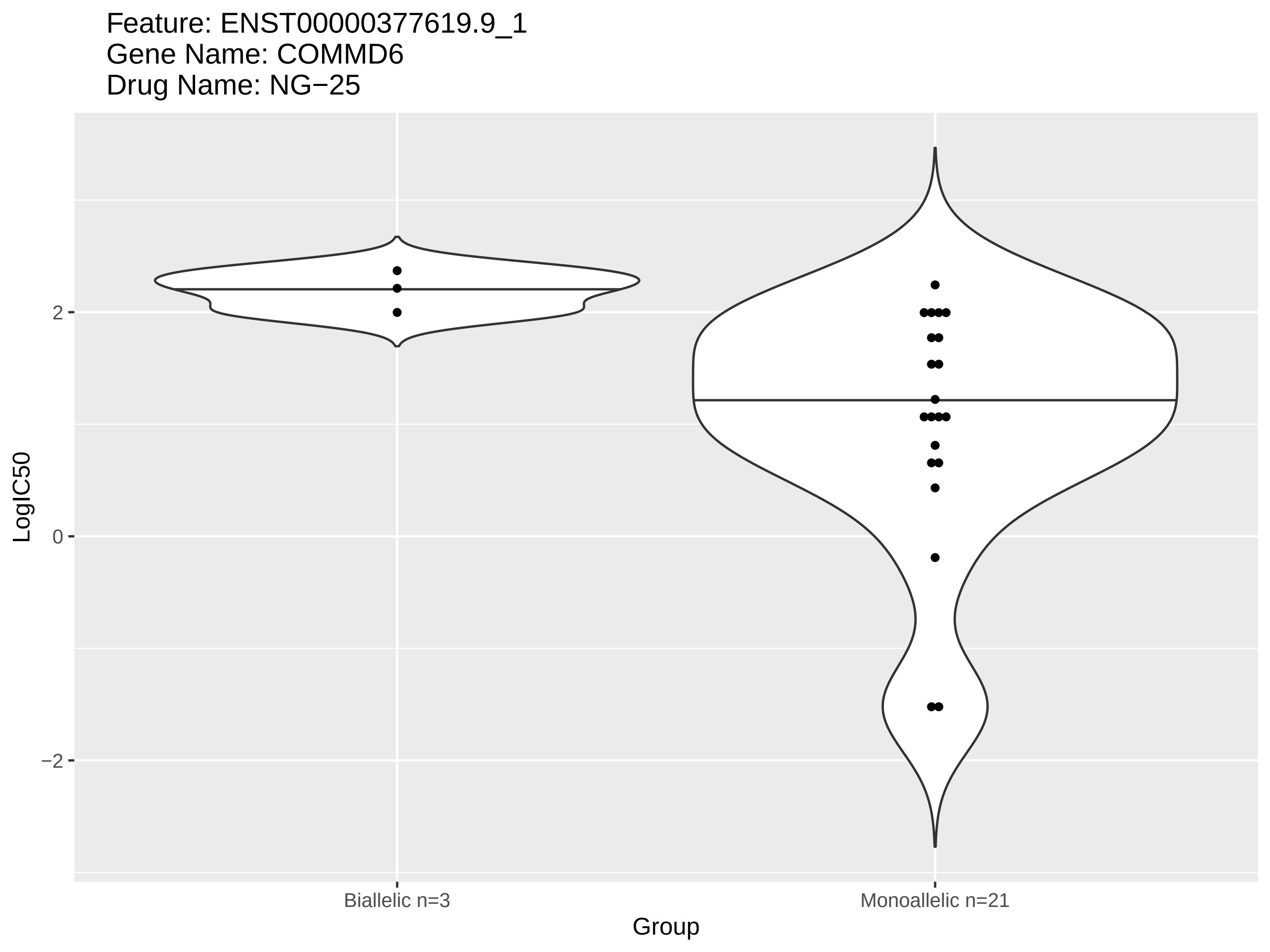
Feature: ENST00000377619.9_1
Gene Name: COMMD6
Drug Name: NG−25
LogIC50
2
0
−2
Biallelic n=3
Monoallelic n=21
Group

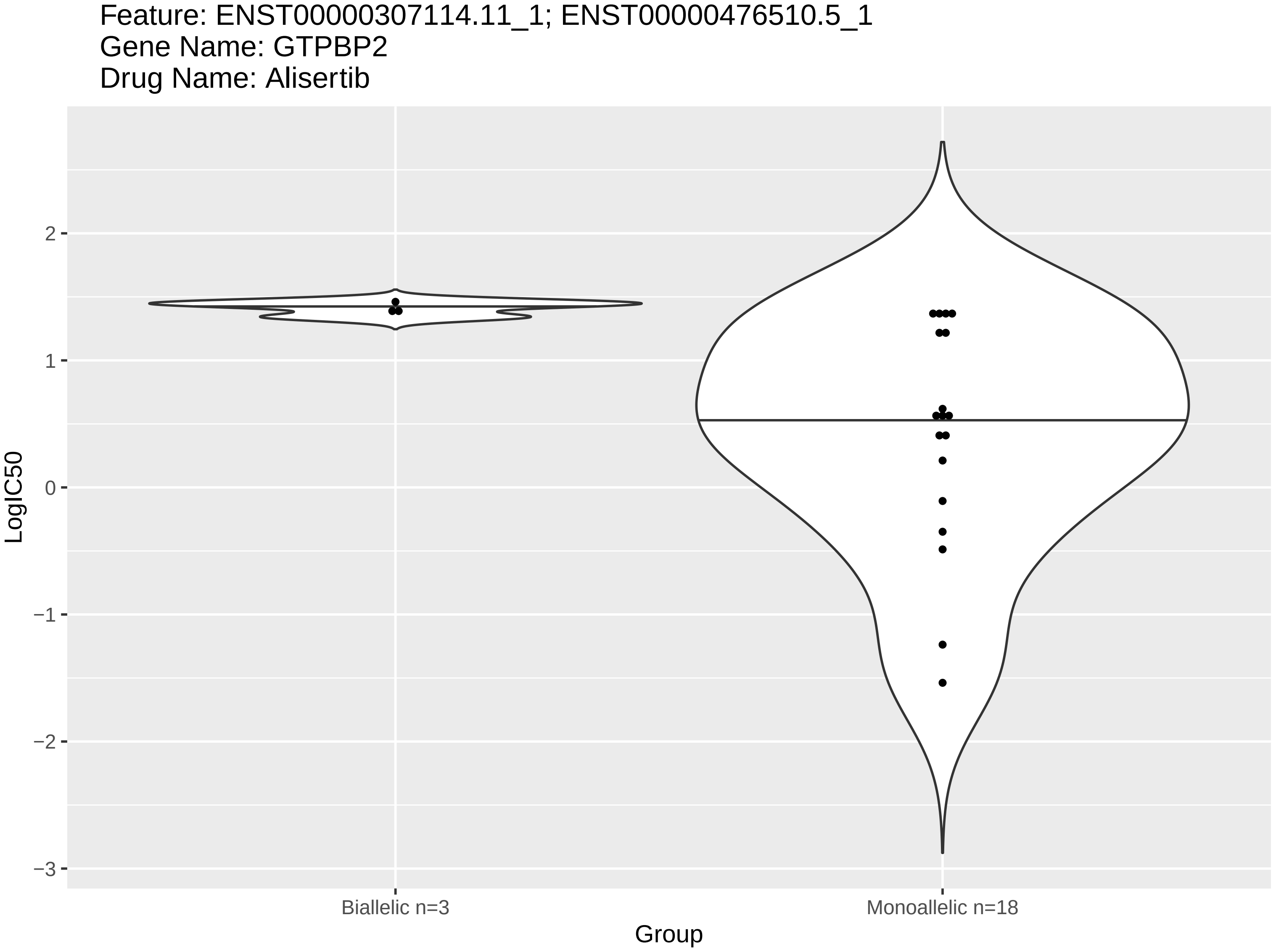
Feature: ENST00000307114.11_1; ENST00000476510.5_1
Gene Name: GTPBP2
Drug Name: Alisertib
LogIC50
2
1
0
−1
−2
−3
Biallelic n=3
Monoallelic n=18
Group

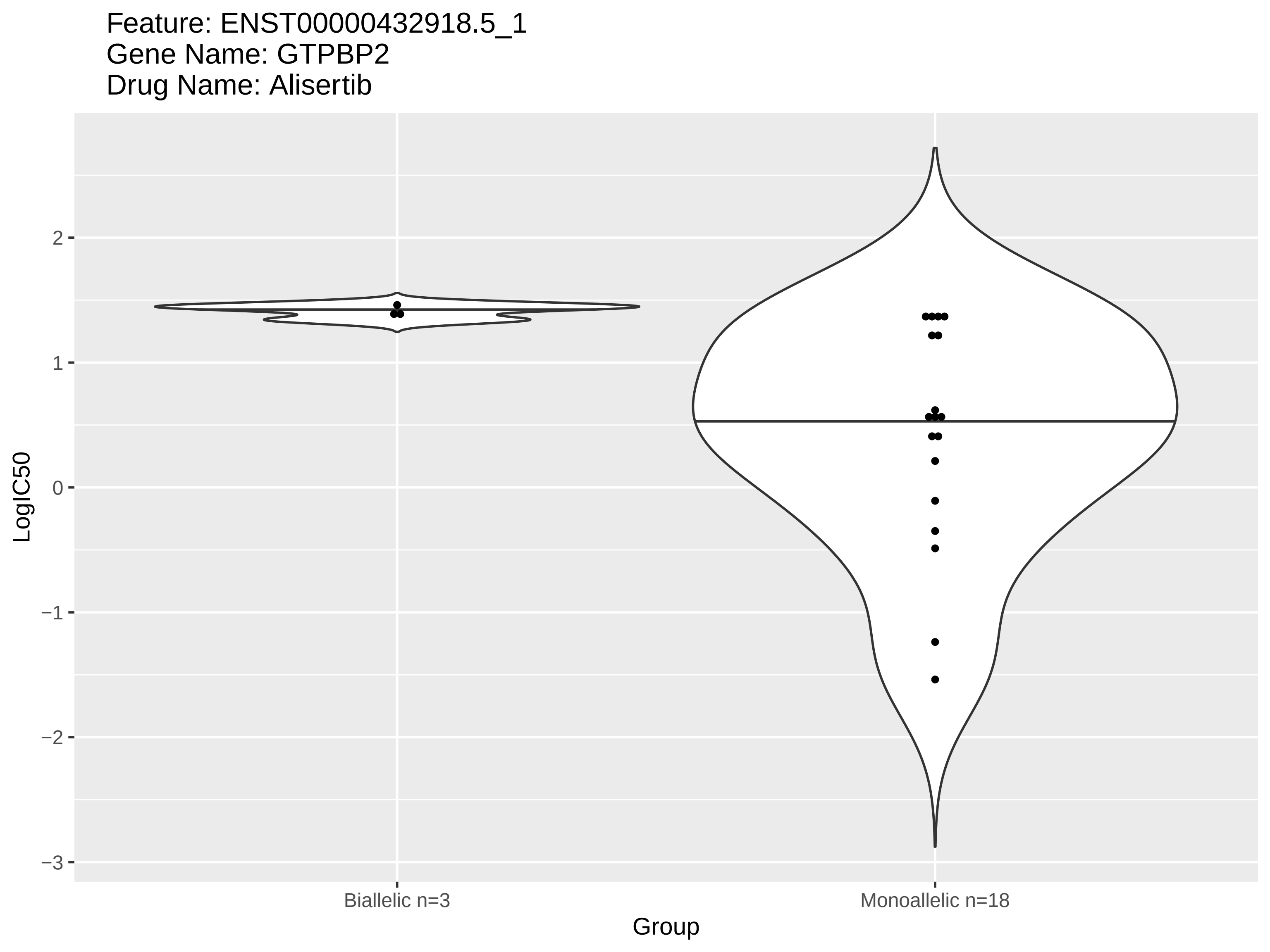

Feature: ENST00000432918.5_1
Gene Name: GTPBP2
Drug Name: Alisertib
2
1
0
−1
−2
−3
LogIC50
Biallelic n=3
Monoallelic n=18
Group

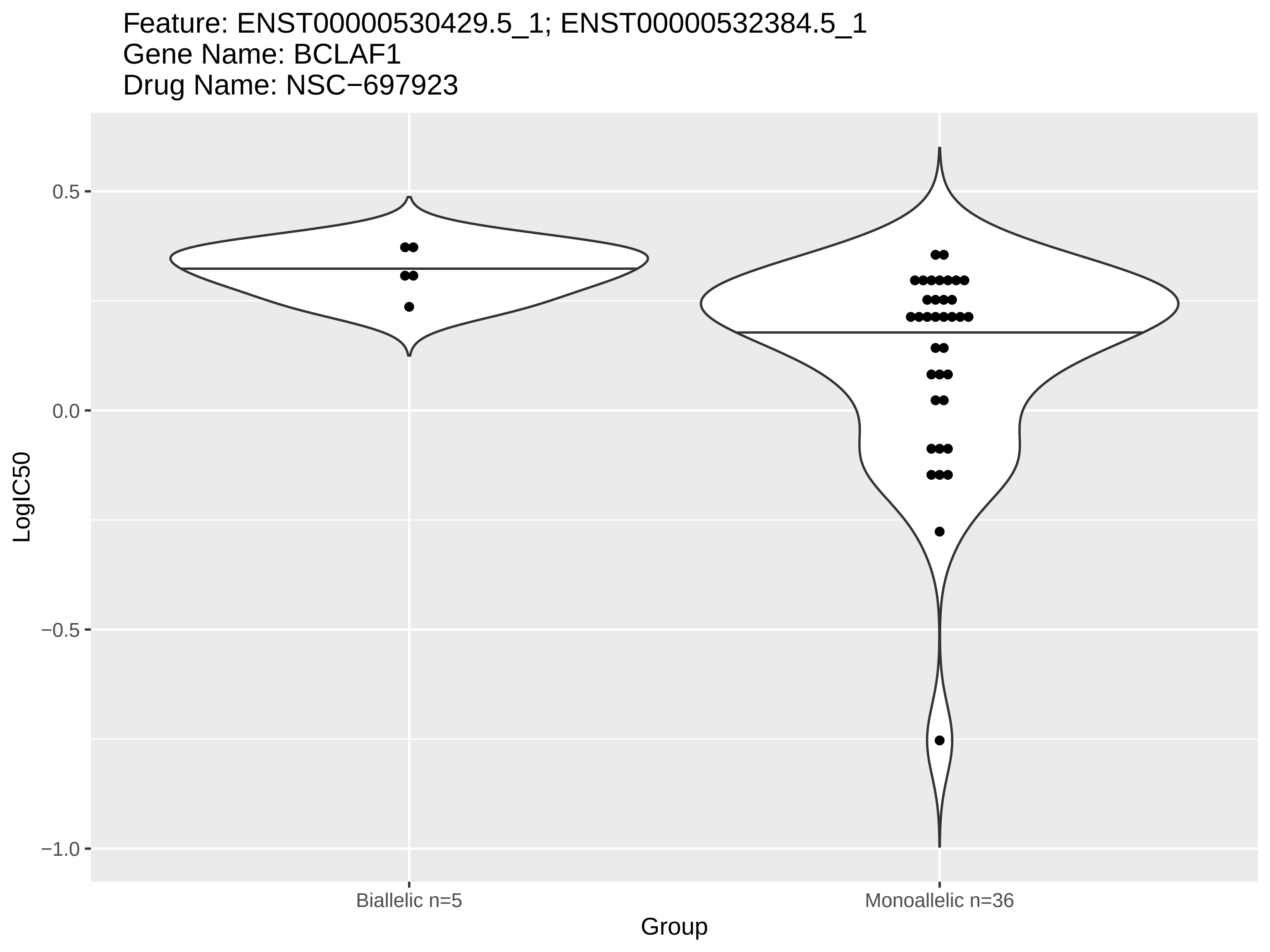
Feature: ENST00000530429.5_1; ENST00000532384.5_1
Gene Name: BCLAF1
Drug Name: NSC−697923
LogIC50
0.5
0.0
−0.5
−1.0
Biallelic n=5
Monoallelic n=36
Group

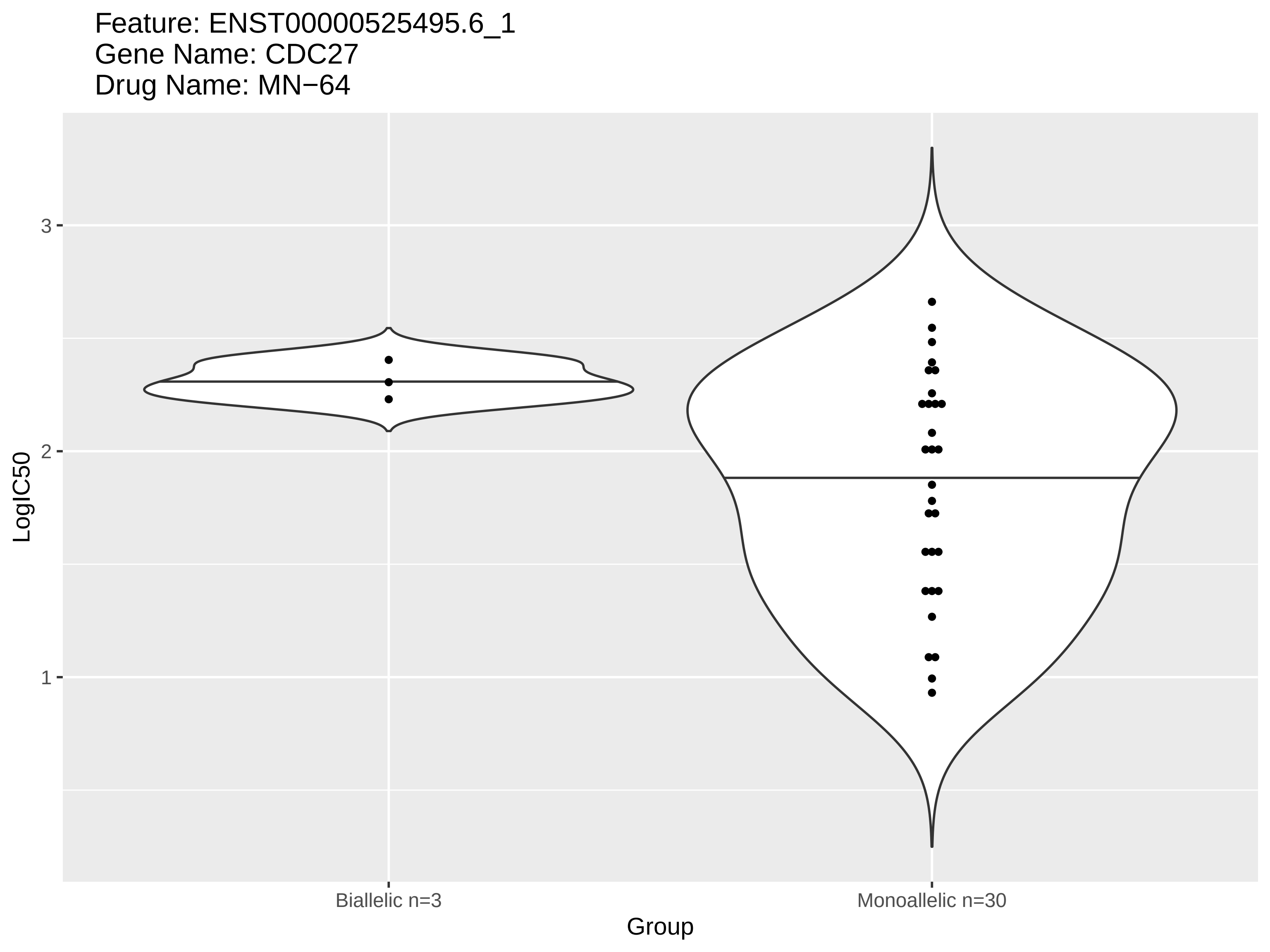

Feature: ENST00000525495.6_1
Gene Name: CDC27
Drug Name: MN−64
LogIC50
3
2
1
Biallelic n=3
Monoallelic n=30
Group

Feature: ENST00000377619.9_1
Gene Name: COMMD6
Drug Name: Camptothecin

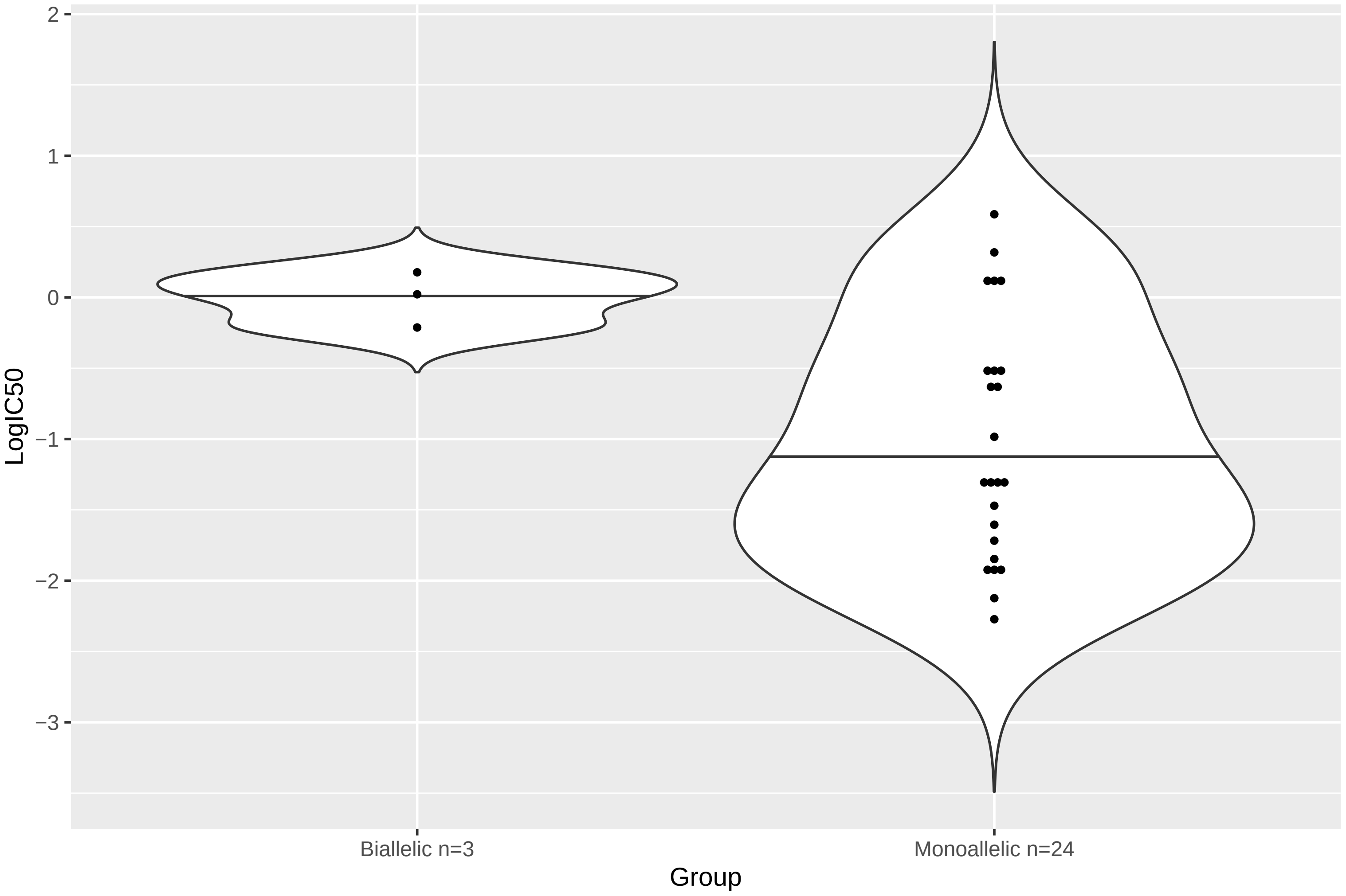

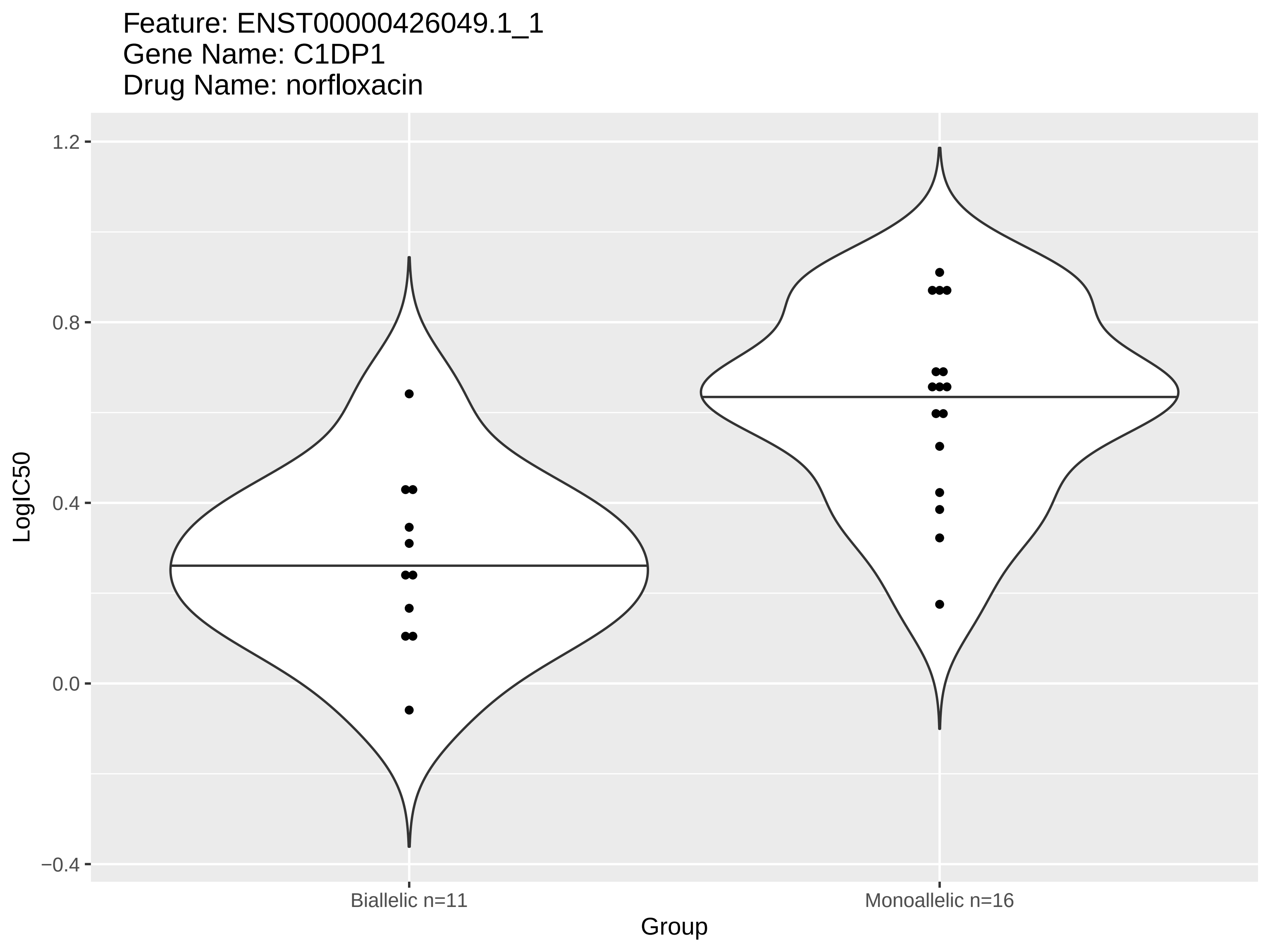

Feature: ENST00000426049.1_1
Gene Name: C1DP1
Drug Name: norfloxacin
1.2
0.8
0.4
0.0
−0.4
LogIC50
Biallelic n=11
Monoallelic n=16
Group

Feature: ENST00000648836.1_1
Gene Name: RP11−234B24.6
Drug Name: AICA Ribonucleotide

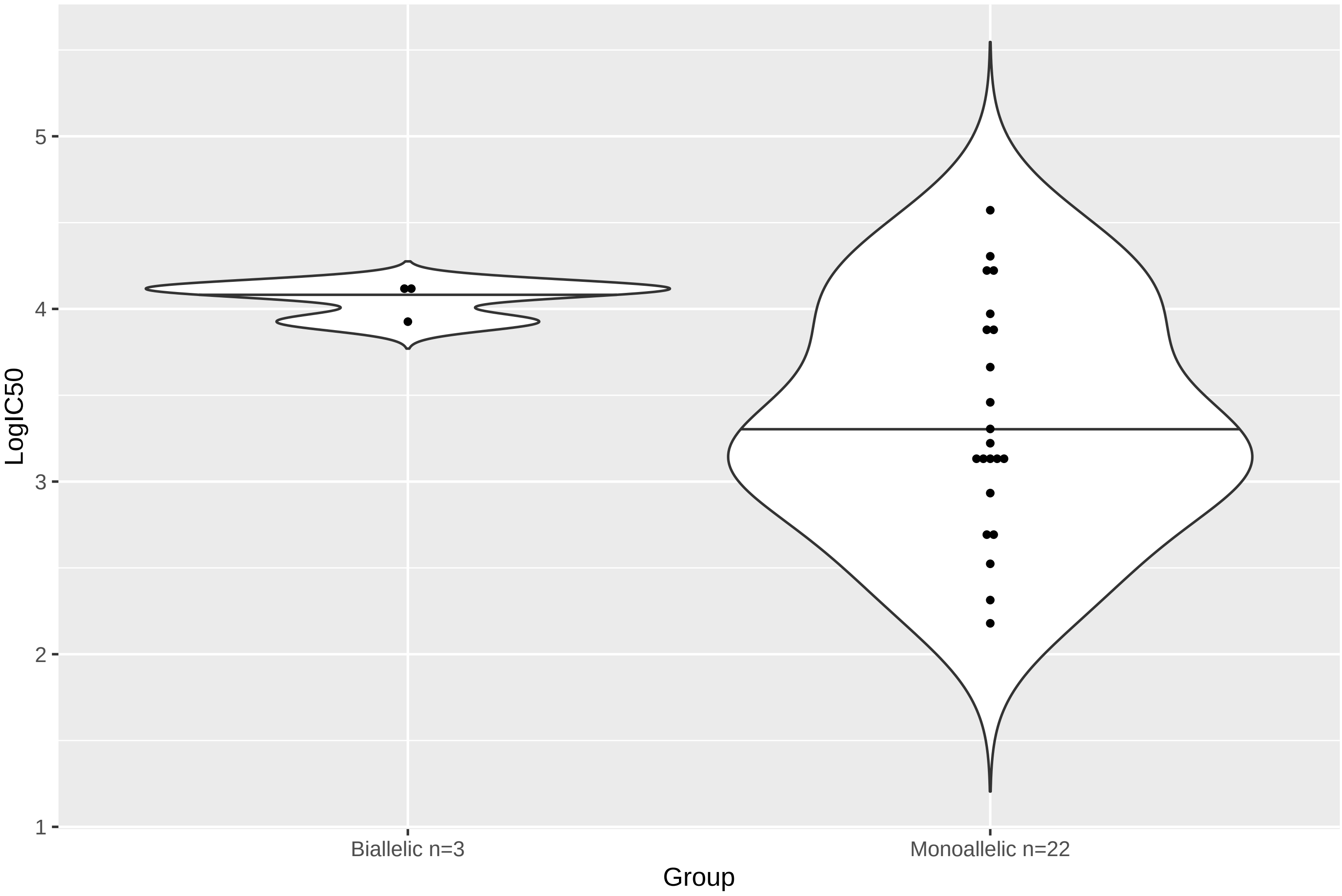

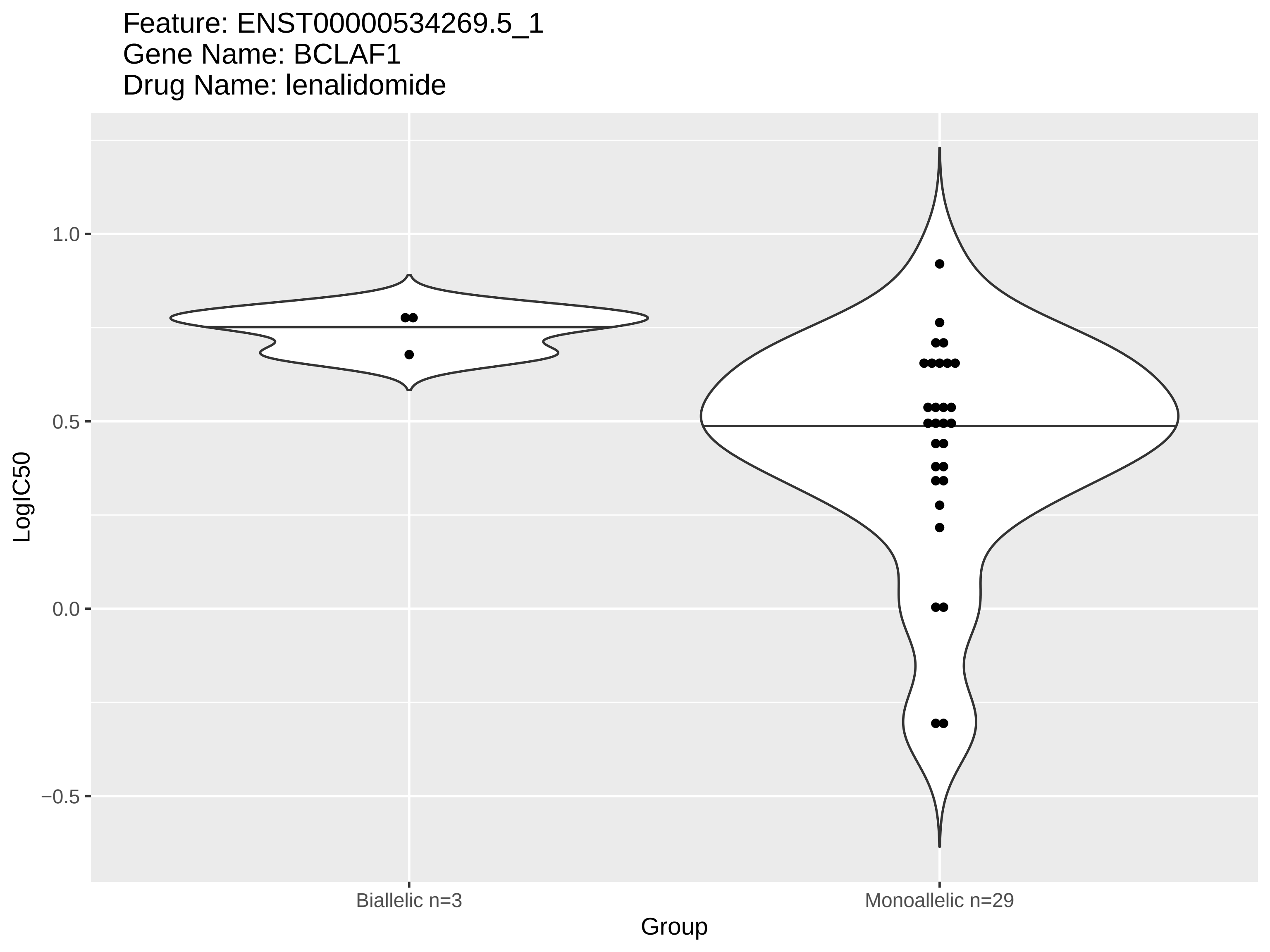
Feature: ENST00000534269.5_1
Gene Name: BCLAF1
Drug Name: lenalidomide
1.0
0.5
0.0
−0.5
LogIC50
Biallelic n=3
Monoallelic n=29
Group

Feature: ENST00000648836.1_1
Gene Name: RP11−234B24.6
Drug Name: fluvastatin

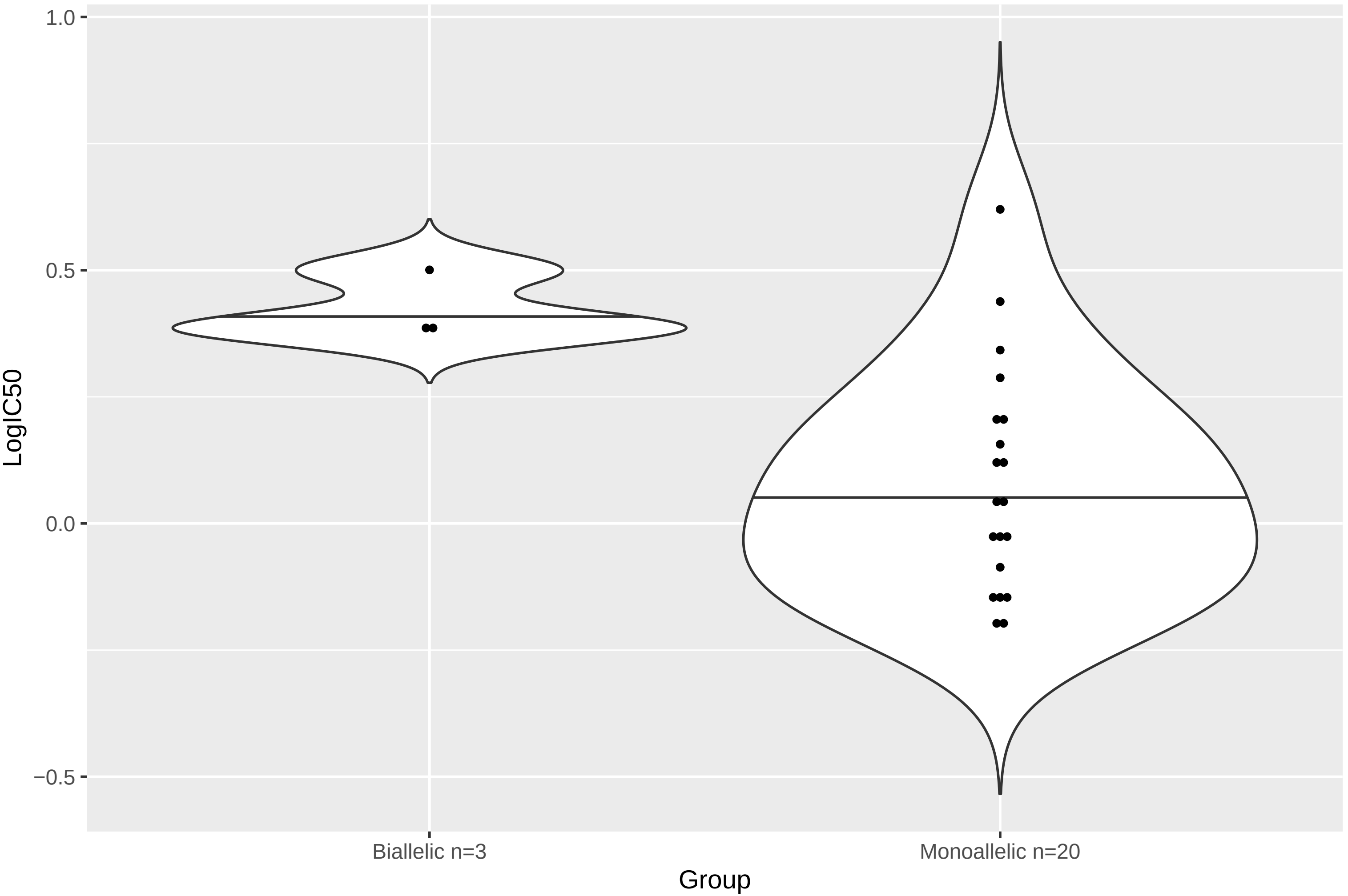

Feature: ENST00000529917.5_1
Gene Name: BCLAF1
Drug Name: tedizolid−phosphate

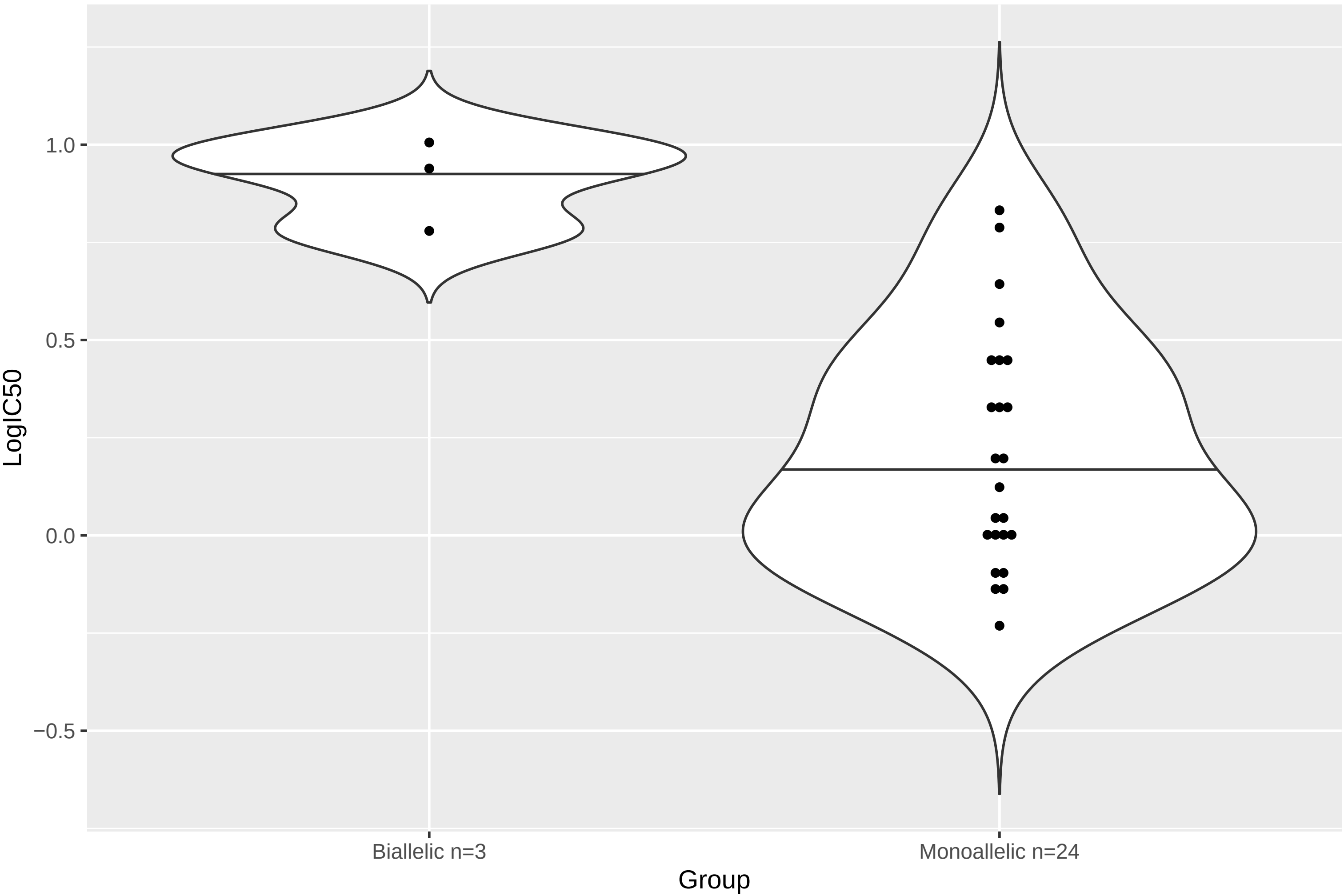

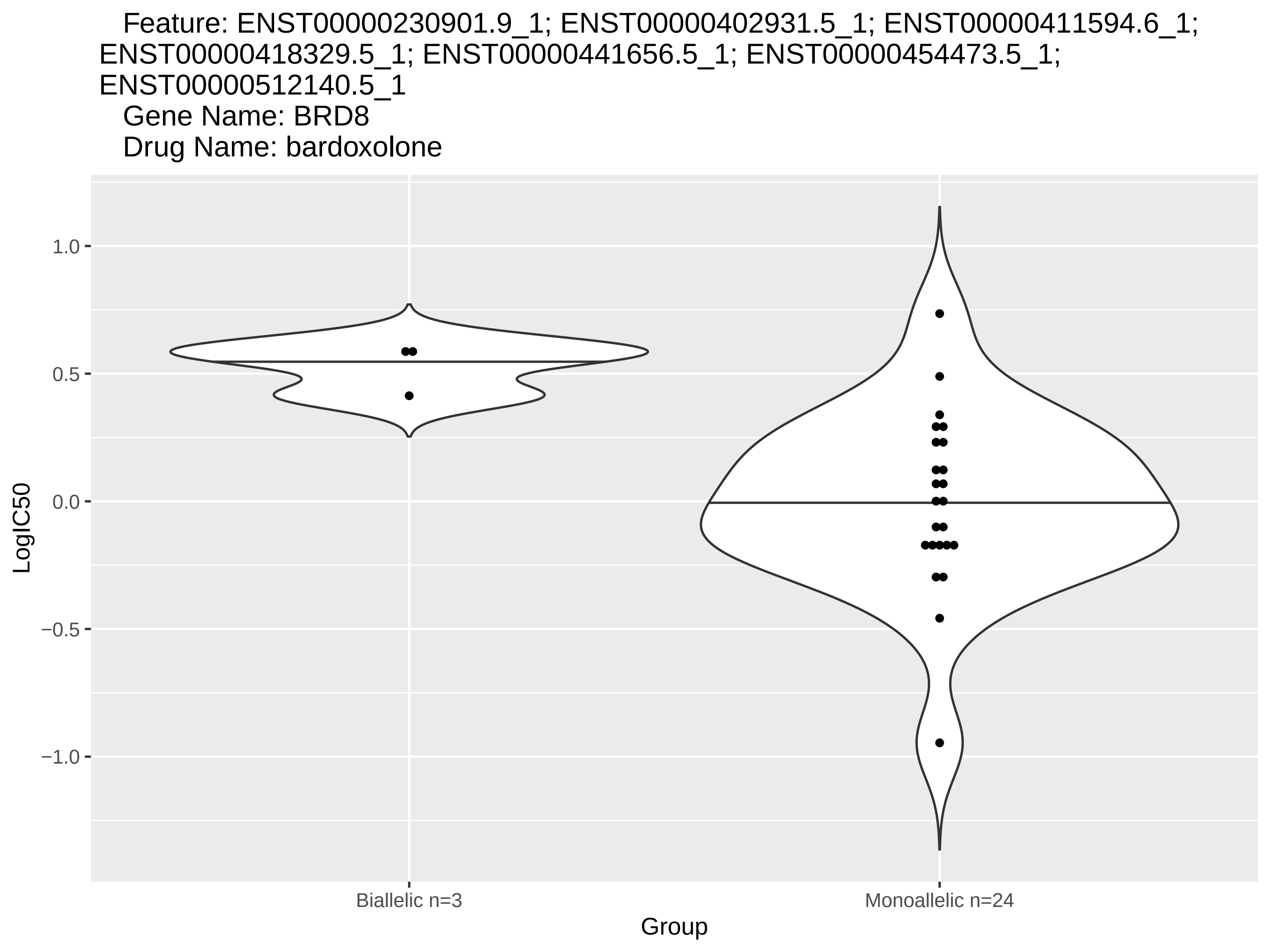
Feature: ENST00000230901.9_1; ENST00000402931.5_1; ENST00000411594.6_1; ENST00000418329.5_1; ENST00000441656.5_1; ENST00000454473.5_1; ENST00000512140.5_1
Gene Name: BRD8
Drug Name: bardoxolone
LogIC50
1.0
0.5
0.0
−0.5
−1.0
Biallelic n=3
Monoallelic n=24
Group
