# Supplementary material for: Allelic expression patterns of imprinted and non-imprinted genes in cancer cell lines from multiple histologies
Source: Clin Epigenetics. 2025 May 25;17:83. doi: 10.1186/s13148-025-01883-3 (PMC12105275; doi:10.1186/s13148-025-01883-3)
Supplement: Supplementary file 28 — Supplementary Material 28. Table S17. Distribution of statistically significant (p < 0.05) preference for monoallelic expression of a single base among SNVs in imprinted genes, as compared to SNVs in other predominantly monoallelically expressed genes. [file 13148_2025_1883_MOESM28_ESM.pdf]

Table S17. Distribution of statistically significant ($p < 0.05$) vs non-significant ($p > 0.05$) preference for monoallelic expression of a single base among SNVs in imprinted genes, as compared to SNVs in other predominantly monoallelically expressed genes

Shown are the summary results of the binomial test for preferential monoallelic expression among 474 SNVs which were expressed in > 5 cell lines. These SNVs belong to 70 previously reported imprinted genes and 60 additional genes with predominantly monoallelic expression. Statistical testing results for each of the 474 individual SNVs and their expression patterns are provided in Table S16A.

Significant base preference, SNVs with statistically significant ($p < 0.05$) preference for monoallelic expression of a particular base
Non-significant base preference, SNVs which did not reach statistical significance for expression of the most frequently monoallelically expressed variant ($p > 0.05$)

	Significant base preference	Non-significant base preference
**Imprinted genes**	16	32
**Other monoallelically expressed genes**	405	21

The difference between imprinted and other monoallelically expressed genes was highly statistically significant ($p < 10^{-15}$, $\chi^2 = 924.7$ using χ^2 test with 3 degrees of freedom).
