# Supplementary material for: Allelic expression patterns of imprinted and non-imprinted genes in cancer cell lines from multiple histologies
Source: Clin Epigenetics. 2025 May 25;17:83. doi: 10.1186/s13148-025-01883-3 (PMC12105275; doi:10.1186/s13148-025-01883-3)
Supplement: Supplementary file 29 — Supplementary Material 29. Supplementary figure and table legends. [file 13148_2025_1883_MOESM29_ESM.pdf]

Supplementary Figure and Table Legends

Supplementary Tables

Additional file 2:Table S1. The initial list 94 imprinted genes compiled from biomedical resources

Listed are the genes with available molecular data which had been reported to be imprinted in embryonic or adult somatic tissues, placenta, embryonic stem cells, or induced pluripotent stem cells (iPSCs).
[a] Chromosomal location is provided according to the data in the Catalog of Imprinted Genes [1-3], Bonaldi et al. [5], and additional publications listed among the references. For those imprinted genes that had minor discrepancies among their reported chromosomal location from different sources, their chromosomal location was reported according to GeneCards [26].
[b] The Geneimprint resource [6] was described in [27].

Gene category is provided according to the information reported by Bonaldi et al. [5] and/or GeneCards [26].
Overlapping exon bases provides the combined length of all isoforms in a given gene, some of which may be overlapping
Unique exon bases provides the total length of unique isoform bases in a given gene, with duplicate positions excluded
Gene and exon annotation is provided according to GENCODE, using lifted annotations from V38lift37 (Ensembl 104) mapped to hg19 (gencode.v38lift37).
The "**_1**" , which is a part of each **Gene ID**, is included in the GENCODE GTF/GFF feature annotations for all genes/transcripts/exons, corresponding to the mapping version "lift" from GRCh38 to GRCh37/hg19 [28].

Additional file 3:Table S2. Cell line data used in analyses of allelic expression status and associations with drug response

AML, acute myeloid leukemia; **SCLC,** small cell lung cancer; **PDAC,** pancreatic ductal adenocarcinoma

Additional file 4:Table S3. List of 108 CCLE cell lines with matching RNA-seq, WES, and copy number data which were included in the analysis of allelic expression patterns, and available drug response information for each cell line

AML, acute myeloid leukemia. **Pancreatic,** pancreatic ductal adenocarcinoma (PDAC)
PRISM compounds, GDSC1 compounds, and **GDSC2 compounds,** provide the numbers of compounds with IC50 drug response data for a given cell line in each of the three datasets.
An empty cell indicates the absence of IC50 drug response data for a given cell line in a given dataset.

Additional file 6:Table S4. Top 60 genes which were not included in the initial list of 94 imprinted genes and were found to have predominantly monoallelic expression in the pancancer analysis of 108 CCLE cell lines

Listed are the genes with > 50 monoallelic counts and ≥ 50% of monoallelic calls out of all calls 108 cell lines, for gene level data.
The genes are sorted by the counts (from highest to lowest) of cell lines with monoallelic expression.
The names of genes shown in red font are discussed in the text and in Table 2, based on previous reports of their potential imprinting status or monoallelic expression due to other causes.

Chr, chromosome
Gene and exon annotations are provided according to GENCODE, using lifted annotations from V38lift37 (Ensembl 104) mapped to hg19 (gencode.v38lift37).
The "**_1**" , which is a part of each Gene ID and Exon ID, is included in the GENCODE GTF/GFF feature annotations for all genes/transcripts/exons, corresponding to the mapping version "lift" from GRCh38 to GRCh37/hg19.
Exon IDs and **Exon ranges** provide identifiers and boundaries of all exons which are annotated to be a part of a given gene
Overlapping exon bases provides the combined length of all isoforms in a given gene, some of which may be overlapping
Unique exon bases provides the total length of unique isoform bases in a given gene, with duplicate positions excluded
Cell lines with copy number loss, Cell lines with no SNV data, Cell lines with no expression, Cell lines with biallelic expression, Cell lines with monoallelic expression, and **Cell lines with unresolved status** provide the number of cell lines with a particular expression pattern for a given gene. The algorithm for inference of expression patterns is shown in Fig. 1.
Discrepancy RNA VCF vs WES VCF indicates cases where only RNA-seq but not WES data indicate the presence of one or more heterozygous SNVs (see Methods)
Total monoallelic or biallelic, the combined number of cell lines with monoallelic and biallelic calls
Percentage of monoallelic calls, indicates the proportion (%) of monoallelic calls out of **Total monoallelic or biallelic** counts

Additional file 7:Table S5. Numbers of agents included in association analyses of *in vitro* drug response with allelic expression patterns and overall expression levels of isoforms

Association analysis with drug response included isoforms of 94 imprinted genes and 60 additional genes with predominantly monoallelic expression.
Shown are numbers of available agents included in comparisons that satisfied sample size requirements ($n \geq 3$ in both biallelic and monoallelic expression group in analysis of allelic expression patterns; $n \geq 10$ for Spearman correlation analysis of overall expression)

Additional file 12:Table S6. Gene level allelic expression patterns of the previously reported 94 imprinted genes among 108 CCLE cell lines

The genes are sorted by the counts (from highest to lowest) of cell lines with monoallelic expression. Allelic expression patterns of individual isoforms of these genes in each tumor category and across all cell lines are provided in Additional file 17:Table S9.

Chr, chromosome
Gene and exon annotations are provided according to GENCODE, using lifted annotations from V38lift37 (Ensembl 104) mapped to hg19 (gencode.v38lift37).
The "**_1**" , which is a part of each Gene ID and Exon ID, is included in the GENCODE GTF/GFF feature annotations for all genes/transcripts/exons, corresponding to the mapping version "lift" from GRCh38 to GRCh37/hg19.
Exon IDs and **Exon ranges** provide identifiers and boundaries of all exons which are annotated to be a part of a given gene
Overlapping exon bases provides the combined length of all isoforms in a given gene, some of which may be overlapping
Unique exon bases provides the total length of unique isoform bases in a given gene, with duplicate positions excluded
Cell lines with copy number loss, Cell lines with no SNV data, Cell lines with no expression, Cell lines with biallelic expression, Cell lines with monoallelic expression, and **Cell lines with unresolved status** provide the number of cell lines with a particular expression pattern for a given gene. The algorithm for inference of expression patterns is shown in Fig. 1.
Discrepancy RNA VCF vs WES VCF indicates cases where only RNA-seq but not WES data indicate the presence of one or more heterozygous SNVs (see Methods)
Total monoallelic or biallelic, the combined number of cell lines with monoallelic and biallelic calls
Percentage of monoallelic calls, indicates the proportion (%) of monoallelic calls out of **Total monoallelic or biallelic** counts

Additional file 14:Table S7. Average proportions (%) of transcriptome-wide allelic expression patterns of genes, isoforms, and individual exons with heterozygous SNVs in 108 CCLE cell lines

Shown is the percentage (%) of each pattern (**Copy number loss, Not expressed, Biallelically expressed,** and **Monoallelically expressed**), among other patterns, after excluding the features with no SNV data, computed among 108 CCLE cell lines from 9 cancer categories

Imprinted genes, indicates 94 imprinted genes listed in Table S1. **Other genes**, indicates 59,189 remaining protein-coding and noncoding gene transcripts

Additional file 15:Table S8. Gene level allelic expression patterns of non-imprinted control genes

Chr, chromosome

Gene and exon annotations are provided according to GENCODE, using lifted annotations from V38lift37 (Ensembl 104) mapped to hg19 (gencode.v38lift37).

The "**_1**" , which is a part of each Gene ID and Exon ID, is included in the GENCODE GTF/GFF feature annotations for all genes/transcripts/exons, corresponding to the mapping version "lift" from GRCh38 to GRCh37/hg19.

Exon IDs and **Exon ranges** provide identifiers and boundaries of all exons which are annotated to be a part of a given gene

Overlapping exon bases provides the combined length of all isoforms in a given gene, some of which may be overlapping

Unique exon bases provides the total length of unique isoform bases in a given gene, with duplicate positions excluded

Copy number loss, No SNV data, Not expressed, Biallelically expressed, Monoallelically expressed, or **Unresolved:** Number of cell lines with given expression pattern as defined by Fig. 1

Discrepancy RNA VCF vs WES VCF indicates cases where only RNA-seq but not WES data indicate the presence of one or more heterozygous SNVs (see Methods)

Total monoallelic or biallelic, the combined number of cell lines with monoallelic and biallelic calls

Percentage of monoallelic calls, indicates the proportion (%) of monoallelic calls out of **Total monoallelic or biallelic** counts

Additional file 17:Table S9. Allelic expression patterns of isoforms of the 94 imprinted genes

Explanation of column headers is provided in the legend to Table S6.

A. Summary of allelic patterns of isoform expression among 108 CCLE cancer cell lines (pancancer data)

B. Summary of allelic patterns of isoform expression among 17 AML cell lines

C. Summary of allelic patterns of isoform expression among 10 bladder cell lines

D. Summary of allelic patterns of isoform expression among 6 breast cell lines

E. Summary of allelic patterns of isoform expression among 13 colorectal cell lines

F. Summary of allelic patterns of isoform expression among 16 head and neck cell lines

G. Summary of allelic patterns of isoform expression among 4 neuroblastoma cell lines

H. Summary of allelic patterns of isoform expression among 14 ovarian cell lines

I. Summary of allelic patterns of isoform expression among 16 PDAC cell lines

J. Summary of allelic patterns of isoform expression among 12 SCLC cell lines

Additional file 19:Table S10. Numbers and proportions of monoallelically and biallelically expressed isoforms of PLAGL1 in different cancer categories

Shown are the numbers and proportions (%) of monoallelically and biallelically expressed isoforms among 28 *PLAGL1* isoforms according to the GENCODE annotation. The tissue-specific differences between monoallelic and biallelic expression patterns in the tissues where any isoforms had such patterns were highly significant ($p = 8.978 \times 10^{-10}$, $\chi^2 = 41.662$ using χ^2 test with 2 degrees of freedom).

AML, acute myeloid leukemia; **SCLC,** small cell lung cancer; **PDAC,** pancreatic ductal adenocarcinoma

Additional file 20:Table S11. Concordance among isoform level allelic expression of CCLE cell lines in our study with previously reported whole gene level results of Martin-Trujillo et al. (2017) in matching COSMIC cell lines

Shown are imprinted genes and cell lines which were profiled in both studies, based on the computational analysis of CCLE cell lines in our study, and on RT-PCR results of matching COSMIC cell lines in the study of Martin-Trujillo et al. *(Martin-Trujillo A, Vidal E, Monteagudo-Sanchez A, Sanchez-Delgado M, Moran S, Hernandez Mora JR et al. 2017. Copy number rather than epigenetic alterations are the major dictator of imprinted methylation in tumors. Nat Commun. 2017;8:467).* Allelic expression inference is shown for three CCLE breast cell lines (HCC1143, HCC1954, MDAMB436) and the colorectal cell line HT55, all of which were also profiled at the whole gene level in COSMIC by Martin-Trujillo et al. in COSMIC using qRT-PCR. Allelic expression inference based on CCLE data is provided for all isoforms of the genes which were profiled in COSMIC in at least one of the four cell lines, and the results for those cell lines which were also profiled in COSMIC are highlighted. The results which were concordant between CCLE and COSMIC findings are highlighted in yellow. The results which were could not be compared between the CCLE and COSMIC datasets due to the absence of heterozygous SNVs, copy number loss, or the lack of expression in one or both datasets are highlighted in green. The only isoform with a discrepancy in its allelic expression patterns between CCLE and COSMIC (ENST00000475188.1_1 of *MEST* in HT-55) is highlighted in purple. All other isoforms of that gene with available heterozygous SNV data were concordant between the two studies.

Chr, chromosome
Gene and exon annotations are provided according to GENCODE, using lifted annotations from V38lift37 (Ensembl 104) mapped to hg19 (gencode.v38lift37).
Transcript ID, isoform ID
The "**_1**" , which is a part of each Gene ID, Isoform ID, and Exon ID, is included in the GENCODE GTF/GFF feature annotations for all genes/transcripts/exons, corresponding to the mapping version "lift" from GRCh38 to GRCh37/hg19.
Exon IDs and **Exon ranges** provide identifiers and boundaries of all exons which are annotated to be a part of a given gene
Overlapping exon bases provides the combined length of all isoforms in a given gene, some of which may be overlapping
Unique exon bases provides the total length of unique isoform bases in a given gene, with duplicate positions excluded
The algorithm for inference of expression patterns **(Copy number loss, No SNV data, Not expressed, Biallelically expressed, Monoallelically expressed, or Unresolved)** is shown in Fig. 1.

Additional file 23:Table S12. Associations of allelic expression patterns of isoform groups of imprinted genes and other genes with predominantly monoallelic expression with in vitro drug response satisfying $p_{FDR} < 0.125$

Shown are association results for Student's t-test of isoform groups with log(IC50) satisfying $p_{FDR} < 0.125$. Isoforms from the same gene with identical monoallelic and biallelic expression patterns were grouped together. Analysis included only genes and agents for which drug response (log(IC50)) which was available for at least 3 cell lines with monoallelic expression and at least 3 cell lines with biallelic expression.
The *p*-values were adjusted for multiple testing using all isoform groups and agents satisfying sample size requirements.

p_0, *p*-value prior to FDR adjustment. **p_{FDR},** *p*-values after FDR adjustment. All associations results listed in the table had $p_0 < 1.98 \times 10^{-4}$. Association results satisfying $p_{FDR} < 0.05$ are highlighted in red.
Isoform group, provides the list of all isoforms from the same gene that had identical patterns of monoallelic and biallelic expression and were included in a given isoform group
Gene, gene name
Chr:positions, provides the chromosome and genome positions according to the human hg19 genome assembly.
Gene category, indicates whether a gene was included in the initial list of 94 imprinted genes (**Imprinted,** Tables S1 and S6); or if that gene was one of the top 60 additional genes with predominantly monoallelic expression (**Other monoallelic,** Table S4)
Drug dataset, dataset (GDSC1, GDSC2, or PRISM) from which the log(IC50) values were obtained
***t*,** *t*-statistic value from the Student's *t*-test comparing the mean log(IC50) values between monoallelically and biallelically expressed cell lines for a given isoform group
N_monoallelic, number cell lines with monoallelic expression of a given isoform group
N_biallelic, number cell lines with biallelic expression of a given isoform group
min log(IC50), minimal value of log(IC50) among all cell lines with monoallelic and biallelic expression of a given isoform group
max log(IC50), maximal value of log(IC50) among all cell lines with monoallelic and biallelic expression of a given isoform group
mean log(IC50) monoallelic, mean value of log(IC50) among the cell lines with monoallelic expression of a given isoform group
mean log(IC50) biallelic, mean value of log(IC50) among the cell lines with biallelic expression of a given isoform group

Additional file 24:Table S13. Spearman correlation of log(IC50) and mean allelic expression levels of isoform groups of imprinted genes and other genes with predominantly monoallelic expression with *in vitro* drug response satisfying p_{FDR} < 0.125

Shown are association results for Spearman correlation analysis of average expression of each isoform group with log(IC50) satisfying p_{FDR}< 0.125.
Analysis included isoform group-drug pairs for which with least 10 cell lines had data available.
Isoforms from the same gene with identical monoallelic and biallelic expression patterns were grouped together, and their mean expression values were used.
The *p*-values were adjusted for multiple testing using all isoform groups and agents satisfying sample size requirements.

p_0, *p*-value prior to FDR adjustment. **p_{FDR}**, *p*-value after FDR adjustment. **Spearman ρ,** Spearman correlation coefficient between expression and log(IC50).
All associations listed in the table also had $p_0 \leq 1.55 \times 10^{-5}$ and $0.6485 \leq |\rho| \leq 0.9648$.
Associations satisfying $p_{FDR} < 0.05$ are highlighted in red.
Isoform group, provides the list of all isoforms from the same gene that had identical patterns of monoallelic and biallelic expression and were included in a given isoform group
Gene, gene name
Chr:positions, provides the chromosome and genome positions according to the human hg19 genome assembly.
Gene category, indicates whether a gene was included in the initial list of 94 imprinted genes (**Imprinted,** Tables S1 and S6); or if that gene was one of the top 60 additional genes with predominantly monoallelic expression (**Other monoallelic**, Table S4)
Drug dataset, dataset (GDSC1, GDSC2, or PRISM) from which the log(IC50) values were obtained
Sample size, number of cell lines with available data used in correlation analysis
min log(IC50), minimal value of log(IC50) among the cell lines used in correlation analysis
max log(IC50), maximal value of log(IC50) among the cell lines used in correlation analysis
min log10(length normalized expression), minimal value of length normalized expression of a given isoform group among the cell lines used in correlation analysis
max log10(length normalized expression), maximal value of length normalized expression of a given isoform group among the cell lines used in correlation analysis

Additional file 25:Table S14. Associations of allelic expression patterns of isoform groups of imprinted genes and other genes with predominantly monoallelic expression with isoform expression satisfying $p_{FDR} <$ 0.125

Shown are association results for Student's *t*-test of isoform groups with log(IC50) satisfying $p_{FDR} < 0.125$. Isoforms from the same gene with identical monoallelic and biallelic expression patterns were grouped together. Only isoform groups with available data for at least 3 cell lines with monoallelic expression and at least 3 cell lines with biallelic expression were analyzed.
The *p*-values were adjusted for multiple testing using all isoform groups and agents satisfying sample size requirements.

p_0, *p*-value prior to FDR adjustment. **p_{FDR},** *p*-value after FDR adjustment
All associations listed in the table also had $p_0 < 0.006$.
Associations satisfying $p_{FDR} < 0.05$ are highlighted in red.
Isoform group, provides the list of all isoforms from the same gene that had identical patterns of monoallelic and biallelic expression and were included in a given isoform group
Gene, gene name
Gene category, indicates whether a gene was included in the initial list of 94 imprinted genes **(Imprinted**, Tables S1 and S6); or if that gene was one of the top 60 additional genes with predominantly monoallelic expression (**Other monoallelic**, Table S4)
***t*,** *t*-statistic value from the Student's *t*-test comparing the mean expression values between monoallelically and biallelically expressed cell lines for a given isoform group
N_monoallelic, number cell lines with monoallelic expression of a given isoform group
N_biallelic, number cell lines with biallelic expression of a given isoform group
min log(length normalized expression), minimal value of log_{10}(length normalized expression) among the cell lines used in the *t*-test analysis
max log(length normalized expression), maximal value of log_{10}(length normalized expression) among the cell lines used in the *t*-test analysis
mean log(length normalized expression) monoallelic, mean value of log_{10}(length normalized expression) among the cell lines with monoallelic expression of a given isoform group
mean log(length normalized expression) biallelic, mean value of log_{10}(length normalized expression) among the cell lines with biallelic expression of a given isoform group

Additional file 26:Table S15. Counts of the expressed allele in monoallelically expressed genes and isoforms and of both alleles in the genomic data for heterozygous SNVs located within 94 imprinted genes and 60 additional genes with predominantly monoallelic expression

Table S15A. Counts of the expressed allele in monoallelically expressed gene level data and of both alleles in the genomic data for heterozygous SNVs located within 94 imprinted genes and 60 additional genes with predominantly monoallelic expression
For each SNV, shown are counts of each expressed allele, and both genomic alleles across the cell lines where its gene was monoallelically expressed. The status of each allele in heterozygous SNVs was inferred from VCF files according to the algorithm presented in Fig. S3.
U in the RNA data is presented as its DNA analog, **T.**
Gene, gene name. **Gene category,** indicates whether a gene was included in the initial list of 94 imprinted genes (**Imprinted,** Tables S1 and S6); or if that gene was one of the top 60 additional genes with predominantly monoallelic expression (**Other monoallelic,** Table S4). **Chr_Pos_RefBase,** provides the chromosome, genome position, and the reference nucleotide of each SNV in the human hg19 genome assembly. **rsID,** SNV ID for SNVs reported in gnomad 2.1.1.
RNA_A, RNA_T, RNA_G, RNA_C, counts of each allele based on the RNA-seq data in the cell lines where the gene containing a given SNV was monoallelically expressed
WES_A, WES_T, WES_G, WES_C, counts of each allele based on the WES data in the cell lines where the gene containing a given SNV was monoallelically expressed
RNA_A_mean_LOG10_LENGTH_NORM_RNA_HTSEQ_COUNTS,
RNA_T_mean_LOG10_LENGTH_NORM_RNA_HTSEQ_COUNTS,
RNA_G_mean_LOG10_LENGTH_NORM_RNA_HTSEQ_COUNTS,
RNA_C_mean_LOG10_LENGTH_NORM_RNA_HTSEQ_COUNTS, mean expression of the gene containing a given allele across the cell lines where this gene was monoallelically expressed

Table S15B. Counts of the expressed allele in monoallelically expressed isoform level data and of both alleles in the genomic data for heterozygous SNVs located within 94 imprinted genes and 60 additional genes with predominantly monoallelic expression
For each SNV, shown are counts of each expressed allele, and both genomic alleles across the cell lines where its isoform was monoallelically expressed. The data are presented separately for each isoform with monoallelic expression, and the SNVs that occur within multiple isoforms are listed more than once. The status of each allele in heterozygous SNVs was inferred from VCF files according to the algorithm presented in Fig. S3.
U in the RNA data is presented as its DNA analog, **T.**
Transcript ID, isoform ID. **Gene,** gene name. **Gene category,** indicates whether a gene for a given SNV and isoform was included in the initial list of 94 imprinted genes (**Imprinted,** Tables S1 and S6), or if that gene was one of the top 60 additional genes with predominantly monoallelic expression (**Other monoallelic,** Table S4).
Chr_Pos_RefBase, provides the chromosome, genome position, and the reference nucleotide of each SNV in the human hg19 genome assembly. **rsID,** SNV ID for SNVs reported in gnomad 2.1.1.
RNA_A, RNA_T, RNA_G, RNA_C, counts of each allele based on the RNA-seq data in the cell lines where the isoform containing a given SNV was monoallelically expressed
WES_A, WES_T, WES_G, WES_C, counts of each allele based on the WES data in the cell lines where the isoform containing a given SNV was monoallelically expressed
RNA_A_mean_LOG10_LENGTH_NORM_RNA_HTSEQ_COUNTS,
RNA_T_mean_LOG10_LENGTH_NORM_RNA_HTSEQ_COUNTS,
RNA_G_mean_LOG10_LENGTH_NORM_RNA_HTSEQ_COUNTS,
RNA_C_mean_LOG10_LENGTH_NORM_RNA_HTSEQ_COUNTS, mean expression of the isoform containing a given allele across the cell lines where this gene was monoallelically expressed

Additional file 27:Table S16. Results of the two-sided binomial test for preferential monoallelic expression of sequence variants

Table S16A. Results of the two-sided binomial test to determine whether each of 474 SNVs showed a preference for monoallelic expression of the reference base vs any non-reference base

Shown are the results of the two-sided binomial test for predominant expression between reference base and any non-reference base in 474 SNVs. Each of the 474 SNVs had monoallelic expression in >5 cell lines. The 474 SNVs belong to 130 genes including 70 imprinted genes and 60 additional predominantly monoallelically expressed genes. Monoallelic expression status of each gene was inferred from multiple SNVs.

Reference indicates the hg19 reference nucleotide in a given position, whereas non-reference indicates a nucleotide other than the reference. **Gene,** gene name. **Gene category,** indicates whether a gene was included in the initial list of 94 imprinted genes (**Imprinted,** Tables S1 and S6), or if that gene was one of the top 60 additional genes with predominantly monoallelic expression (**Other monoallelic**, Table S4). **Chr_Pos_RefBase,** provides the chromosome, genome position, and the reference nucleotide of each SNV in the human hg19 genome assembly. **rsID,** SNV ID for SNVs reported in gnomad 2.1.1. ***p*-value,** the 2-sided binomial *p*-value for the proportion of reference allele under a null assumption of 50% reference vs 50% non-reference base. ***p* < 0.05,** indicator of whether the *p*-value was less than 0.05 (considered statistically significant and marked by *)
RNA ref count, WES ref count, RNA nonref count, and **WES nonref** count indicate the reference and non-reference cell line counts in the RNA-seq and WES data. **RNA ref count, WES ref count, RNA nonref count,** and **WES** indicate the number of cell lines that express the reference or non-reference base in the RNA-seq and WES data**. WES base types** indicates how many different nucleotide types were present in the WES data (usually 2 alleles)**. N monoallelically expressed,** total number of cell lines with monoallelic expression of a given gene. **Reference proportion,** proportion of cell lines that monoallelically express the reference nucleotide. **Mostly reference,** indicator of whether there are more reference than non-reference nucleotides in monoallelically expressed cell lines (i.e. whether the **Reference proportion** > 0.5). **All ref or nonref,** indicator of whether the cell lines monoallelically expressed either 100% reference or 100% non-reference base (i.e, the **Reference proportion** = 0 or 1). **RNA_A, RNA_T, RNA_G, RNA_C,** number of cell lines that monoallelically expressed a given base. **WES_A, WES_T, WES_G, WES_C,** number of those cell lines which monoallelically express the gene that have a given base represented in WES.
RNA_A_mean_LOG10_LENGTH_NORM_RNA_HTSEQ_COUNTS,
RNA_T_mean_LOG10_LENGTH_NORM_RNA_HTSEQ_COUNTS,
RNA_G_mean_LOG10_LENGTH_NORM_RNA_HTSEQ_COUNTS,
RNA_C_mean_LOG10_LENGTH_NORM_RNA_HTSEQ_COUNTS, mean expression of the isoform containing a given allele across the cell lines where this gene was monoallelically expressed

Table S16B. Gene level summary of the results of the two-sided binomial test for a preference for monoallelic expression of the reference base vs any non-reference base in individual SNVs in 130 genes

Gene, gene name; **Gene category,** indicates whether a gene was included in the initial list of previously reported imprinted genes (**Imprinted,** Tables S1 and S6), or if that gene was one of the top 60 additional genes with predominantly monoallelic expression (**Other monoallelic**, Table S4). **Monoallelic SNVs**, number of SNVs in a given gene that were present in the RNA-seq data of at least 1 cell line with monoallelic expression of that gene. **Monoallelic SNVs in >5 cell lines**, number of SNVs in a given gene that were present in the RNA-seq data of >5 cell lines with monoallelic expression of that gene. **Significant SNVs**, total number of SNVs in a given gene that have a significant binomial test (*p* < 0.05), as shown in Table S16A. **Significant SNVs biased to ref**, number of significant SNVs (*p* < 0.05) that showed bias to monoallelic expression of the hg19 reference allele. **Significant SNVs all ref or none ref**, the number of significant SNVs that either all had the monoallelic expression of the reference allele or none of them had the monoallelic expression of the reference allele. **Significant SNVs all ref,** the number of significant SNVs that all had the monoallelic expression of the reference allele

Additional file 28:Table S17. Distribution of statistically significant (*p* < 0.05) vs non-significant (*p* > 0.05) preference for monoallelic expression of a single base among SNVs in imprinted genes, as compared to SNVs in other predominantly monoallelically expressed genes

Shown are the summary results of the binomial test for preferential monoallelic expression among 474 SNVs which were expressed in > 5 cell lines. These SNVs belong to 70 previously reported imprinted genes and 60 additional genes with predominantly monoallelic expression. Statistical testing results for each of the 474 individual SNVs and their expression patterns are provided in Table S16A.
Significant base preference, SNVs with statistically significant ($p < 0.05$) preference for monoallelic expression of a particular base
Non-significant base preference, SNVs which did not reach statistical significance for expression of the most frequently monoallelically expressed variant ($p > 0.05$)
The difference between imprinted and other monoallelically expressed genes was highly statistically significant ($p < 10^{-15}$, $\chi^2 = 924.7$ using χ^2 test with 3 degrees of freedom).

Supplementary Figures

Additional file 1:Figure S1. An overview of the steps of the analysis of CCLE cancer cell line data. Detailed description of each step is provided in the Methods section. The algorithm for inference of allelic patterns of expression is provided in Fig. 1. **EBI,** European Bioinformatics institute; **UCSC,** University of California, Santa Cruz.

Additional file 5:Figure S2. Proportion of genes and isoforms with no detected heterozygous SNVs relative to $\log_{10}$ length normalized HTSEQ counts in RNA-seq and WES data. **(A)** Genes, RNA-seq expression data; **(B)** Genes, WES data; **(C)** isoforms, RNA-seq expression data; **(D)** isoforms, WES data. LOESS regression line is shown in green. Separate plots are provided for each of the 9 cancer categories and for the combined pancancer dataset.

Additional file 8:Figure S3. A flowchart of inference from VCF files of the expressed allele in monoallelically expressed isoforms and genes in 94 imprinted genes and 60 additional genes with predominantly monoallelic expression.
Features, genes or isoforms. An alternative base was reported in the VCF files relative to the human hg19 genome reference sequence.

Additional file 9:Figure S4. Number of RNA-seq and WES sequencing reads mapped to exons using HTSeq in each of the 9 tumor histologies of the 108 cell lines

Additional file 10:Figure S5. Comparison of the allelic expression patterns of the 94 imprinted vs 59,189 remaining genes at the **(A)** gene, **(B)** isoform, and **(C)** exon levels in each of the 9 cancer histologies of the 108 cell lines. Boxplots of the 94 **imprinted genes** listed in Additional file 2:Table S1 are represented by the lighter shades (left). Boxplots of the **other genes** representing the remaining 59,189 genes not included in the original list of 94 imprinted genes are shown by the darker shades of the same color for each tumor category (right).

Additional file 11:Figure S6. Comparison of the gene level allelic expression patterns of the 94 imprinted genes vs average expression patterns in 94 genes that were resampled, using 1000 replications, from 59,189 remaining genes. Boxplots of the 94 **imprinted genes** listed in Additional file 2:Table S1 are represented by the lighter shades (left). Boxplots of the 94 **subsampled genes** are shown by the darker shades of the same color for each tumor category (right).

Additional file 13:Figure S7. Heatmap of tumor histology-specific monoallelic expression among the 94 imprinted genes. For each gene and tumor category, shown is the proportion of monoallelically expressed genes among monoallelically or biallelically expressed genes, at the whole gene level. Dendrograms were inferred using Euclidian distances and complete linkage clustering. Biallelic only expression (values of 0 monoallelic and ≥ 1 biallelic counts) is presented by light blue color. **N/A**, cases with 0 monoallelic and 0 biallelic counts, are shown as grey color. Coloring of > 0 to 1 is on a 100 color gradient.

Additional file 16:Figure S8. Comparison of the allelic expression patterns of the 94 imprinted genes vs 60 additional predominantly monoallelically expressed genes at the **(A)** gene, **(B)** isoform, and **(C)** exon levels in each of the 9 cancer histologies of the 108 cell lines. Boxplots of the 94 imprinted genes listed in Additional file 2:Table S1 are represented by the lighter shades (left, marked as **Imprinted** in the legend). Boxplots of the 60 additional predominantly monoallelically expressed genes listed in Additional file 6:Table S4 are shown by the darker shades of the same color for each tumor category (right, marked as **Mono** in the legend).

Additional file 18:Figure S9. Tissue-specific allelic expression patterns of 28 *PLAGL1* isoforms.
Shown are proportions (%) of each allelic expression category within the cell lines from each of the 9 tumor categories

Additional file 21:Figure S10. Violin plots for associated agents and expression patterns of the isoform groups satisfying $p_{FDR} < 0.05$ for the 94 imprinted genes.

Additional file 22:Figure S11. Violin plots for associated agents and expression patterns of the isoform groups satisfying $p_{FDR} < 0.05$ for 60 predominantly monoallelic genes.
